# Supplementary material for: Evolution of the Dearomative Functionalization of Activated Quinolines and Isoquinolines: Expansion of the Electrophile Scope
Source: Angew Chem Int Ed Engl. 2022 May 13;61(27):e202204682. doi: 10.1002/anie.202204682 (PMC9321684; doi:10.1002/anie.202204682)
Supplement: Supplementary file 1 — Supporting Information [file ANIE-61-0-s001.pdf]

Angewandte Chemie
Eine Zeitschrift der Gesellschaft Deutscher Chemiker

Supporting Information

Evolution of the Dearomative Functionalization of Activated Quinolines and Isoquinolines: Expansion of the Electrophile Scope

*M. Kischkewitz, B. Marinic, N. Kratena, Y. Lai, H. B. Hepburn, M. Dow, K. E. Christensen, T. J. Donohoe**

Electronic Supplementary Information

Contents

General Experimental Techniques 2
Chemicals and solvents 2
Glassware and reaction conditions 2
Analytical techniques 2
Chromatography 3
Optimisation of reaction conditions 4
Unsuccessful electrophiles 5
Synthesis of quinolinium salts 6
General procedure A: Preparation of quinolinium salts 6
Synthesis of Tetrahydroquinolines 13
General Procedure B: Preparation of Tetrahydroquinolines 13
General Procedure C: Metal Catalysed Preparation of Tetrahydroquinolines 13
Synthesis of isoquinolinium salts 26
General Procedure D: Preparation of isoquinolinium salts 26
Synthesis of tetrahydroisoquinolines 33
General procedure E: Synthesis of tetrahydroisoquinolines 33
General procedure F: Rhodium catalysed synthesis of tetrehydroisoquinolines 33
Derivatisations of tetrahydroisoquinolines 53
Alkylation of isoquinolinium salts 55
General procedure G: Alkylation of isoquinolinium salts with aldehydes 55
Annulation reactions 64
Investigation into reaction kinetics 71
General 71

Analysis of kinetic data 71
Deuterium labelling experiments 72
Proposed mechanism for quinolinium and isoquinolinium salts 78
Crystallographic data 79
Spectra 83

General Experimental Techniques

Chemicals and solvents

Unless stated otherwise, all chemicals were purchased from commercial suppliers (Sigma-Aldrich, Fluorochem, Alfa Aesar, TCI) and used without further purification. Formic acid/triethylamine 5:2 complex was purchased from Sigma-Aldrich and used without further purification. Rh-catalyst stock solutions were prepared in a 25-mL volumetric flask by dissolving 2 mg $[RhCp^*Cl_2]_2$ in acetonitrile. All solvents used were HPLC grade or p.a. unless stated otherwise. Pentamethylphenyl vinyl ketone was prepared according to a published procedure.[1]

Glassware and reaction conditions

Reactions were carried out in flasks, oven-dried microwave vials, or fresh GC vials under an atmosphere of air unless otherwise stated.

Analytical techniques

^{1}H, and ^{13}C NMR spectra were recorded on a Bruker AVIII400 Spectrometer (^{1}H: 400 MHz and ^{13}C: 101 MHz), Bruker AVII500 (^{1}H: 500 MHz and ^{13}C: 126 MHz) or a Bruker AvanceIV600 (^{1}H: 600 MHz and ^{13}C: 151 MHz) in $CDCl_3$, DMSO-d_6 or C_6D_6 and referenced to residual solvent peaks. Chemical shifts δ are quoted in parts per million (ppm) to the nearest 0.01 for ^{1}H and 0.1 for ^{13}C, coupling constants *J* are quoted in Hz to the nearest 0.1 and splitting are recorded as singlet (s), doublet (d), triplet (t), quartet (q), pentet (p), hexet (h), heptet (hept), and multiplet (m). Assignments were based upon COSY, HSQC and HMBC experiments. Where unambiguous assignments could not be made the candidate positions are indicated by solidus "/". Low temperature[2] single crystal X-ray diffraction data were collected using a (Rigaku) Oxford Diffraction SuperNova diffractometer for **2m·HCl**, **8a·HCl** and **8e** and data were collected for **5m·HCl** at , Beamlime I19-1, Diamond Light Source.[3] Raw frame data were reduced using

[1] Keumi, T., Inagaki, T., Nakayama, N., Taniguchi, M., Morita, T., Kitajima, H., *J. Org. Chem.* **1989**, 17, 54, 4034 – 4038
[2] Cosier J.; Glazer, A. M. *J. Appl. Cryst.* **1986**, *19*, 105-107.
[3] Allan, D. R. et al. *Crystals*. **2017**, *7(11)*, 336-358.

CrysAlisPro and the structures were solved using 'Superflip'[4] before refinement with CRYSTALS.[5] The structures were then modified, improved and optimised by full-matrix least squares on F^2 as per the SI (CIF). Infrared spectra were recorded on a Bruker Tensor 27 FT-IR spectrometer fitted with an Attenuated Total Reflectance (ATR) sampling accessory. Absorption maxima are quoted in wavenumbers (cm^{-1}). High resolution mass spectra were recorded on a Bruker MicroTOF (resolution = 10000 FWHM). Melting points (m.p.) were obtained using a Lecia VMGT heated-stage microscope and are uncorrected.

Chromatography

Analytical thin layer chromatography was performed on pre-coated silica gel aluminium sheets from Merck (TLC Silica Gel 60 F_{254}). Spots were visualized either by the quenching of UV fluorescence or by staining with phosphomolybdic acid/cerium sulfate, potassium permanganate or vanillin solutions. Preparative flash column chromatography was carried out using Geduran Silica Gel 60 (40 µm – 63 µm) from Merck.

[4] Palatinus L.; Chapuis, G. *J. Appl. Cryst.* **2007**, *40*, 786-790.
[5] (a) Parois, P.; Cooper, R. I.; Thompson, A. L. *Chem. Cent. J.* **2015**, *9*, 30. (b) Cooper, R. I.; Thompson, A. L.; Watkin, D. J. *J. Appl. Cryst*. **2010**, *43*, 1100-1107.

Optimisation of reaction conditions

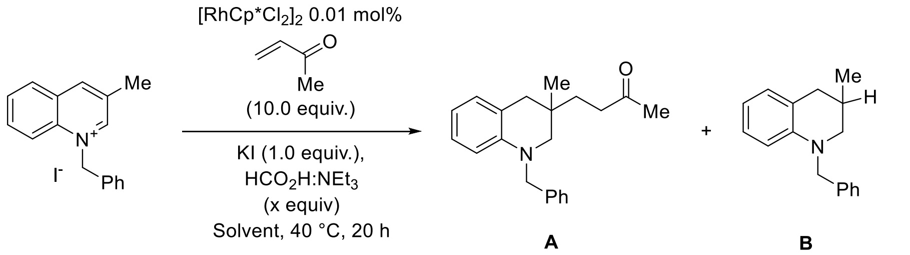

entry	solvent	conc.	equiv. of ”H$^-$ “	Yield A	Yield B
1	neat	0.13 M	100	59%	5%
2	CH_2Cl_2	0.1 M	10	66% (63%)	/
3	DCE	0.1 M	10	46%	/
4	CCl_3H	0.1 M	10	10%	/
5	THF	0.1 M	10	/	/
6	MeCN	0.1 M	10	41%	12%
7	MeOH	0.1 M	10	Complex mixtures	
8	*i*-PrOH	0.1 M	10	Messy	
9	TFE	0.1 M	10	Sm only	
10	HFIP	0.1 M	10	Sm only	
11	H_2O	0.1 M	10	Complex mixtures	
12	PhMe	0.1 M	10	7%	/

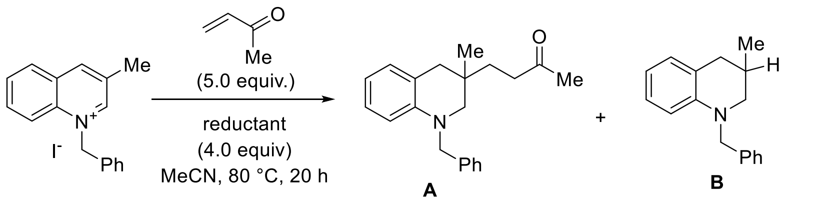

entry	Reductant	”H-“	Yield A	Yield B
1	$HCO_2H:NEt_3$ 5:2	4	86%	<5%
2	HCO_2H	4	0%	0%
3	$HCO_2H:NEt_3$ 1:1	4	86%	<5%
4	$HCO_2H:NEt_3$ 2:5	4	67%	24%
5	$NaHCO_2$	4	0%	0%
6	NH_4HCO_2	4	0%	0%

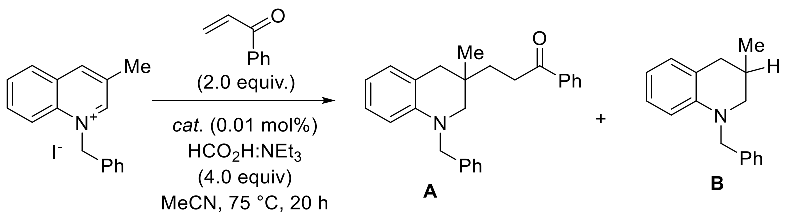

entry	Catalyst	Yield A	Yield B
1	$[RhCp^*Cl_2]_2$	83%	0%
2	[Ru(*p-cymene*)$Cl_2]_2$	85%	0%
3	$[IrCp^*Cl_2]_2$	83%	0%

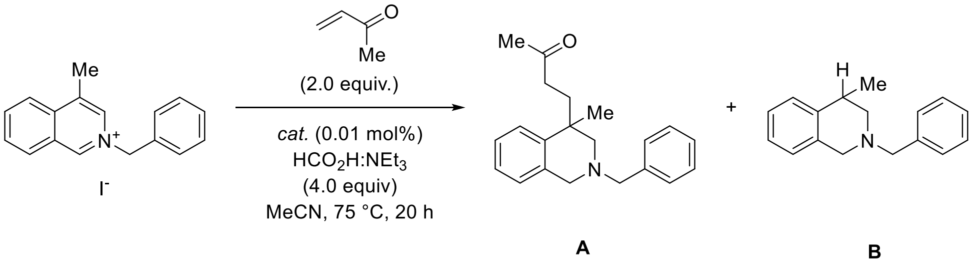

entry	Catalyst	Yield A	Yield B
1	$[RhCp^*Cl_2]_2$	94%	0%
2	[Ru(*p-cymene*)$Cl_2]_2$	77%	0%
3	$[IrCp^*Cl_2]_2$	80%	0%

Unsuccessful electrophiles

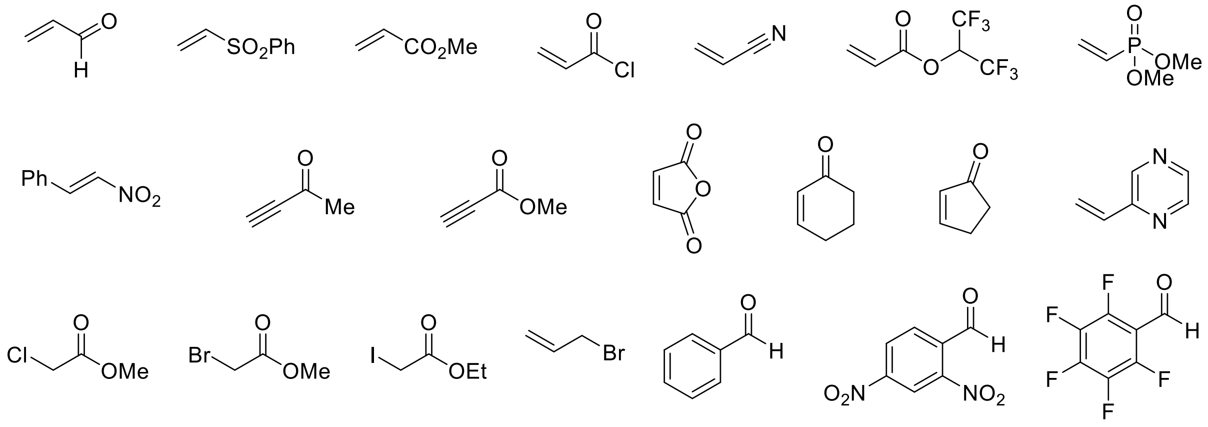

Figure 1: Electrophiles that were unsuccessful in the reaction with quinoline **1a**

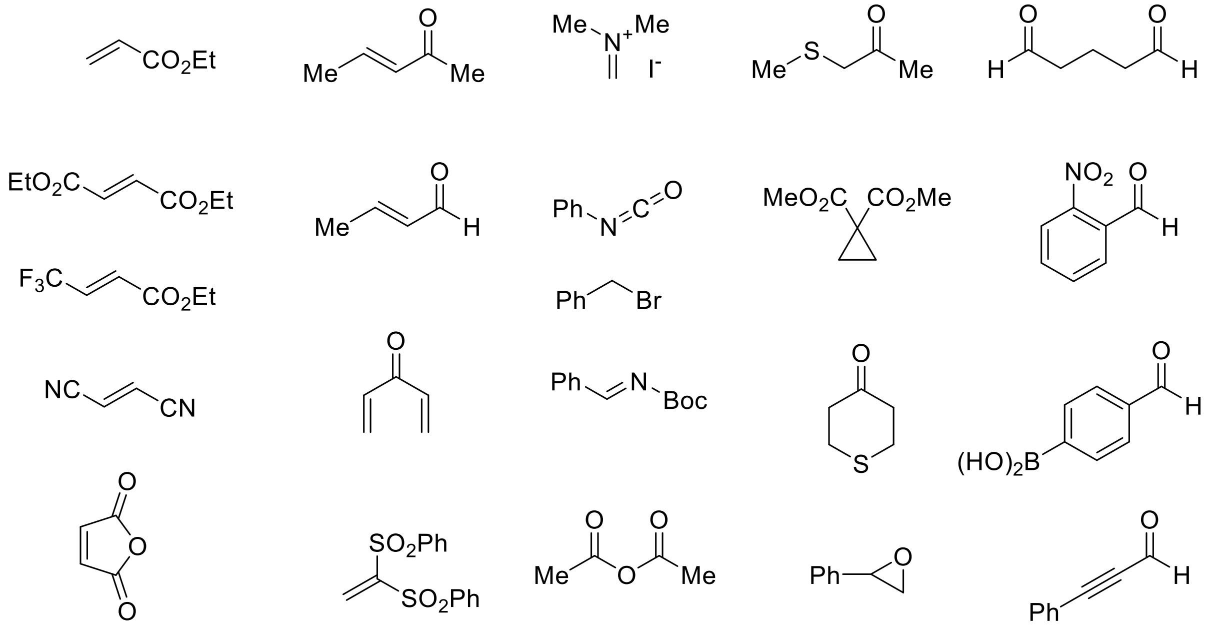

Figure 2: Electrophiles that were unsuccessful in the reaction with isoquinoline **4a**

Synthesis of quinolinium salts

General procedure A: Preparation of quinolinium salts

A mixture of the corresponding quinoline (1.0 equiv.) and benzyl iodide (2.0 equiv.) in acetone (0.5 M) was stirred in the dark at room temperature for 20 – 48 hours. Addition of diethyl ether (10 mL) resulted in precipitation and the resulting suspension was sonicated (15 min). The solids were collected by filtration, washed with diethyl ether and dried under vacuum to give the benzyl quinolinium iodide salts as crystalline solids.

N-Benzyl-3-methylquinolinium iodide (1a)

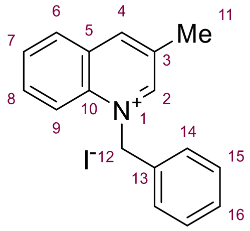

The title compound was prepared according to General Procedure **A** using 3-methylquinoline (716 mg, 5.00 mmol) and benzyl iodide (1.25 mL, 10.0 mmol) in acetone (10 mL) to give salt **1a** as a yellow solid (1.44 g, 80%). Spectroscopic data was consistent with that reported in the literature.[6]

HRMS (ESI): Exact mass calculated for $C_{17}H_{16}N$ $[M+H]^+$: 234.1277, found: 234.1279.

^{1}H NMR (400 MHz, DMSO-d_6) δ 9.77 (d, J = 1.9 Hz, 1H, C^2**H**), 9.20 (d, J = 2.1 Hz, 1H, C^4**H**), 8.43 (dd, J = 9.0, 1.0 Hz, 1H, C^9**H**), 8.38 (dd, J = 8.2, 1.5 Hz, 1H, C^6**H**), 8.12 (ddd, J = 8.8, 7.0, 1.5 Hz, 1H, C^8**H**), 7.97 (ddd, J = 8.0, 7.0, 0.9 Hz, 1H, C^7**H**), 7.45 – 7.30 (m, 5H, 2 x C^{14}**H** + 2 x C^{15}**H** + C^{16}**H**), 6.33 (s, 2H, C^{12}**H$_2$**), 2.71 (s, 3H, C^{11}**H$_3$**).

^{13}C NMR (101 MHz, DMSO-d_6) δ 151.5 (C^{2}H), 146.8 (C^{4}H), 135.9 (C^{10}), 134.6 (C^{8}H), 133.9 (C^{13}), 132.6 (C^3), 130.0 (C^{6}H), 129.9 (C^{7}H), 129.6 (C^5), 129.0 (2 x C^{14}H/C^{15}H), 128.7 (C^{16}H), 127.2 (2 x C^{14}H/C^{15}H), 119.0 (C^{9}H), 59.9 (C^{12}H$_2$), 18.1 (C^{11}H$_3$).

IR (neat) (cm^{-1}): 2981, 2160, 2034, 2009, 1978, 1523, 1362, 771, 753, 738.

N-Benzyl-3-isopropylquinolinium iodide (1b)

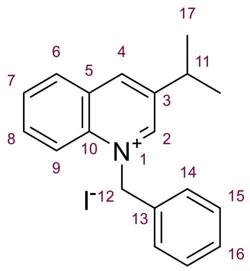

The title compound was prepared according to General Procedure **A** using 3-isopropylquinoline (514 mg, 3.00 mmol) and benzyl iodide (0.75 mL, 6.00 mmol) to give salt **1b** as a yellow solid (746 mg, 65%).

m.p. (acetone): 196 – 198 °C

HRMS (ESI): Exact mass calculated for $C_{19}H_{20}N$ $[M+H]^+$: 262.1590, found: 262.1591.

^{1}H NMR (400 MHz, DMSO-d_6) δ 9.85 (d, J = 2.0 Hz, 1H, C^2**H**), 9.30 (d, J = 2.0 Hz, 1H, C^4**H**), 8.43 (dd, J = 8.2, 1.6 Hz, 2H, C^{6}H + C^{9}H), 8.13 (ddd, J = 8.9, 7.0, 1.5 Hz, 1H, C^{7}H/C^{8}H), 7.98 (ddd, J = 7.9, 7.0, 0.9 Hz, 1H, C^{7}H/C^{8}H), 7.44 – 7.30 (m, 5H, 5 x C^{Ar}**H**), 6.37 (s, 2H, C^{12}**H$_2$**), 3.45 – 3.34 (m, 1H, C^{11}**H**), 1.46 (d, J = 6.9 Hz, 6H, 2 x C^{17}**H$_3$**).

^{13}C NMR (101 MHz, DMSO-d_6) δ 150.3 (C^{2}H), 144.6 (C^{4}H), 142.5 (C^{Ar}), 136.2 (C^{Ar}), 134.8 (C^{8}H), 134.0 (C^{Ar}), 130.3 (C^{6}H), 129.90 (C^{Ar}/C^{7}H), 129.88 (C^{Ar}/C^{7}H), 129.1 (2 x C^{14}H/C^{15}H), 128.7 (C^{16}H), 127.0 (2 x C^{14}H/C^{15}H), 119.0 (C^{9}H), 60.0 (C^{12}H$_2$), 31.3 (C^{11}H), 22.9 (2 x C^{17}H$_3$).

IR (neat) (cm^{-1}): 2981, 2528, 2160, 2031, 1977, 1602, 1509, 770, 751, 733.

[6] Grozavu, A.; Hepburn, H. B.; Smith, P. J.; Potukuchi, H. K.; Lindsay-Scott, P. J.; Donohoe, T. J.; *Nat. Chem.* **2019**, *11*, 242–247

***N*-Benzyl-3-benzylquinolinium iodide (1c)**

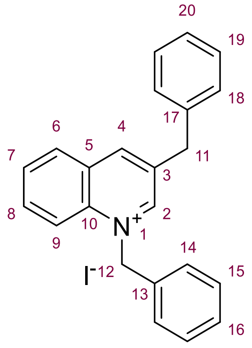

The title compound was prepared according to General Procedure **A** using 3-benzylquinoline (170 mg, 0.78 mmol) and benzyl iodide (0.19 mL, 1.55 mmol) in acetone (1 mL) to give salt **1c** as a yellow solid (275 mg, 81%). Spectroscopic data was consistent with that reported in the literature.[2]

^{1}H NMR (400 MHz, DMSO-d_6) δ 9.83 (s, 1H, C^{2}**H**), 9.22 (s, 1H, C^{4}**H**), 8.46 (d, *J* = 9.0 Hz, 1H, C^{9}**H**), 8.42 (dd, *J* = 8.3, 1.4 Hz, 1H, C^{6}**H**), 8.14 (ddd, *J* = 8.8, 7.0, 1.5 Hz, 1H, C^{8}**H**), 7.97 (ddd, *J* = 8.1, 7.0, 0.9 Hz, 1H, C^{7}**H**), 7.49 – 7.21 (m, 10H, 10 x C^{Ar}**H**), 6.35 (s, 2H, C^{12}**H₂**), 4.43 (s, 2H, C^{11}**H₂**).

^{13}C NMR (101 MHz, DMSO-d_6) δ 151.1 ($C^{2}H$), 146.8 ($C^{4}H$), 138.7 (C^{Ar}), 136.3 (C^{Ar}), 135.9 (C^{Ar}), 135.1 ($C^{8}H$), 133.9 (C^{Ar}), 130.4 ($C^{6}H$), 130.0 (C^{Ar}), 129.8 ($C^{7}H$), 129.03 (2 x $C^{Ar}H$), 128.99 (2 x $C^{Ar}H$), 128.9 (2 x $C^{Ar}H$), 128.7 ($C^{16}H/C^{20}H$), 127.2 (2 x $C^{Ar}H$), 126.9 ($C^{16}H/C^{20}H$), 119.1 ($C^{9}H$), 60.0 ($C^{12}H_2$), 37.5 ($C^{11}H_2$).

***N*-Benzyl-3-(3-methoxy-3-oxopropyl)quinolinium iodide (1d)**

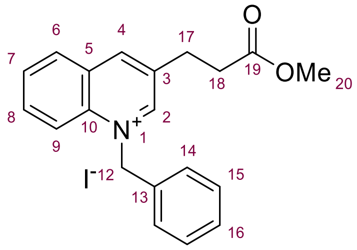

The title compound was prepared according to General Procedure **A** using 3-methylquinoline (538 mg, 2.50 mmol) and benzyl iodide (0.63 mL, 5.00 mmol) to give salt **1d** as a yellow solid (983 mg, 91%). Spectroscopic data was consistent with that reported in the literature.[2]

^{1}H NMR (400 MHz, DMSO-d_6) δ 9.82 (d, *J* = 1.9 Hz, 1H, C^{2}**H**), 9.27 (s, 1H, C^{4}**H**), 8.45 (dd, *J* = 9.0, 1.0 Hz, 1H, C^{9}**H**), 8.39 (dd, *J* = 8.2, 1.4 Hz, 1H, C^{6}**H**), 8.14 (ddd, *J* = 8.8, 7.0, 1.5 Hz, 1H, C^{8}**H**), 7.98 (ddd, *J* = 8.0, 7.0, 0.9 Hz, 1H, C^{7}**H**), 7.48 – 7.23 (m, 5H, 5 x C^{Ar}**H**), 6.34 (s, 2H, C^{12}**H₂**), 3.61 (s, 3H, C^{20}**H₃**), 3.28 (t, *J* = 7.3 Hz, 2H, C^{18}**H₂**), 2.98 (t, *J* = 7.3 Hz, 2H, C^{17}**H₂**).

^{13}C NMR (101 MHz, DMSO-d_6) δ 172.2 ($C^{19}O$), 151.3 ($C^{2}H$), 146.7 ($C^{4}H$), 136.1 (C^{Ar}), 135.3 (C^{Ar}), 135.0 ($C^{8}H$), 133.8 (C^{Ar}), 130.3 ($C^{6}H$), 130.0 ($C^{7}H$), 129.6 (C^{Ar}), 129.0 (2 x $C^{14}H/C^{15}H$), 128.8 ($C^{16}H$), 127.2 (2 x $C^{14}H/C^{15}H$), 119.0 ($C^{9}H$), 60.0 ($C^{12}H_2$), 51.6 ($C^{20}H_2$), 33.2 ($C^{18}H_2$), 27.1 ($C^{17}H_2$).

_N_-Benzyl-2-methylquinolinium iodide (1e)

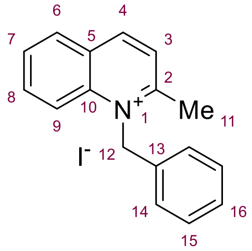

The title compound was prepared according to a modified General Procedure **A**. A solution of 3-methylquinoline (0.7 mL, 5.0 mmol) and benzyl iodide (1.25 mL, 10.0 mmol) in 1,4-dioxane (10 mL) was heated to 90 °C for 16°h. The reaction mixture was cooled to room temperature, addition of Et_2O (20 mL) resulted in formation of the precipitate which was filtered and washed with Et_2O to give salt **1e** as a brown solid (768 mg, 43%).

m.p. (EtOAc): 200 – 204 °C

HRMS (ESI): Exact mass calculated for $C_{17}H_{16}N$ $[M+H]^+$: 234.1277, found: 234.1279.

^{1}H NMR (400 MHz, DMSO-d_6) δ 9.24 (d, J = 8.5 Hz, 1H, C^{4}**H**), 8.46 (dd, J = 8.2, 1.5 Hz, 1H, C^{6}**H**), 8.39 (d, J = 9.0 Hz, 1H, C^{9}**H**), 8.25 (d, J = 8.6 Hz, 1H, C^{3}**H**), 8.15 (ddd, J = 8.9, 7.0, 1.6 Hz, 1H, C^{8}**H**), 7.98 (dd, J = 8.0, 7.0 Hz, 1H, C^{7}**H**), 7.42 – 7.29 (m, 3H, 3 x C^{Ar}**H**), 7.18 – 7.08 (m, 2H, 2 x C^{Ar}**H**), 6.33 (s, 2H, $C^{12}\mathbf{H_2}$), 3.08 (s, 3H, $C^{11}\mathbf{H_3}$).

^{13}C NMR (101 MHz, DMSO-d_6) δ 161.7 (C^{2}), 146.7 (C^{4}H), 138.9 (C^{Ar}), 135.5 (C^{8}H), 133.2 (C^{Ar}), 130.7 (C^{6}H), 129.22 (C^{7}H), 129.20 (2 x C^{15}H), 128.4 (C^{Ar}/C^{16}H), 128.2 (C^{Ar}/C^{16}H), 125.9 (2 x C^{14}H), 125.8 (C^{3}H), 119.2 (C^{9}H), 54.4 ($C^{12}H_2$), 22.8 ($C^{11}H_3$).

IR (neat) (cm^{-1}): 2961, 2530, 2160, 2029, 1977, 1602, 1523, 1350, 827, 771.

_N_-Benzyl-2,3-dimethylquinolinium iodide (1f)

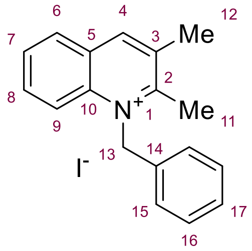

The title compound was prepared according to a modified General Procedure **A**. A solution of 3,4-dimethylquinoline (555 mg, 3.5 mmol) and benzyl iodide (0.88 mL, 7.0 mmol) in 1,4-dioxane (7.0 mL) was heated to 90 °C for 16°h. The reaction mixture was cooled to room temperature, addition of Et_2O (20 mL) resulted in formation of the precipitate which was filtered and washed with Et_2O to give salt **1f** as a yellow solid (433 mg, 33%).

m.p. (EtOAc): 200 – 202 °C

HRMS (ESI): Exact mass calculated for $C_{18}H_{18}N$ $[M+H]^+$:248.1434, found: 248.1436.

^{1}H NMR (400 MHz, DMSO-d_6) δ 9.10 (s, 1H, C^{4}**H**), 8.33 (dd, J = 8.2, 1.8 Hz, 2H, C^{6}**H** + C^{9}**H**), 8.07 (ddd, J = 8.9, 7.0, 1.6 Hz, 1H, C^{7}**H**/C^{8}**H**), 7.94 (dd, J = 7.7, 7.7 Hz, 1H, C^{7}**H**/C^{8}**H**), 7.43 – 7.29 (m, 3H), 7.15 (dd, J = 7.7, 1.7 Hz, 2H, 2 x C^{15}**H**), 6.37 (s, 2H, $C^{13}\mathbf{H_2}$), 2.97 (s, 3H, $C^{11}\mathbf{H_3}$), 2.68 (2, 3H, $C^{12}\mathbf{H_3}$).

^{13}C NMR (101 MHz, $CDCl_3$) δ 162.3 (C^{2}), 145.4 (C^{4}H), 137.8 (C^{Ar}), 134.4 (C^{7}H/C^{8}H), 133.7 (C^{Ar}), 133.3 (C^{Ar}), 129.7 (C^{7}H/C^{8}H), 129.2 (C^{4}H), 129.1 (2 x C^{16}H), 128.1 (C^{Ar}/C^{17}H), 127.8 (C^{Ar}/C^{17}H), 125.9 (2 x C^{15}H), 119.2 (C^{9}H), 55.0 ($C^{13}H_2$), 20.1 ($C^{11}H_3$), 19.7 ($C^{12}H_3$).

IR (neat) (cm^{-1}): 2956, 2529, 2160, 2032, 1977, 1509, 771, 751, 734, 692.

_N_-Benzyl-4-(methoxycarbonyl)quinolinium iodide (1g)

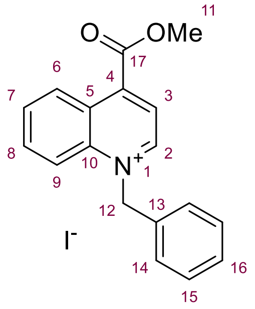

The title compound was prepared according to a modified General Procedure **A**. Methyl quinoline-4-carboxylate (710 mg, 3.79 mmol) and benzyl iodide (0.96 mL, 7.60 mmol) in acetone (9 mL) were stirred at rt in the dark for 24 hours to give salt **1g** as a red solid (650 mg, 42%). Spectroscopic data was consistent with that reported in the literature.[2]

^{1}H NMR (400 MHz, DMSO-d_6) δ 9.87 (d, *J* = 6.0 Hz, 1H, C^{2}**H**), 8.79 (dd, *J* = 8.6, 1.4 Hz, 1H, C^{6}**H**), 8.63 – 8.56 (m, 2H, C^{3}**H** + C^{9}**H**), 8.27 (ddd, *J* = 8.9, 7.0, 1.5 Hz, 1H, C^{8}**H**), 8.11 (ddd, *J* = 8.1, 7.0, 1.0 Hz, 1H, C^{7}**H**), 7.45 – 7.33 (m, 5H, 5 C^{Ar}**H**), 6.44 (s, 2H, C^{12}**H$_2$**), 4.11 (s, 3H, C^{11}**H$_3$**).

^{13}C NMR (101 MHz, DMSO-d_6) δ 164.1 (C^{17}O), 151.0 (C^{2}H), 145.0 (C^{Ar}), 138.3 (C^{Ar}), 135.7 (C^{8}H), 133.4 (C^{Ar}), 131.1 (C^{7}H), 129.1 (2 x C^{14}H/C^{15}H), 128.9 (C^{16}H), 127.8 (C^{3}H), 127.5 (2 x C^{14}H/C^{15}H), 126.5 (C^{Ar}), 122.8 (C^{3}H), 119.9 (C^{9}H), 60.7 (C^{12}H$_2$), 54.1 (C^{11}H$_3$).

1,3-Dimethylquinolinium iodide (1h)

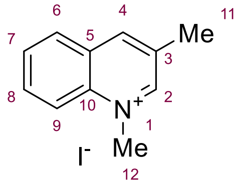

The title compound was prepared according to a modified General Procedure **A**. A mixture of 3-methylquinoline (573 mg, 4.00 mmol) and iodomethane (1.24 mL, 20.0 mmol) in 1,4-dioxane (0.4 M) was heated in a sealed pressure resistant flask at 90 °C for 16 hours. The mixture was allowed to cool to room temperature, the solid was collected by filtration, washed with diethyl ether and dried under vacuum for one hour to give title compound **1h** as a yellow solid (1.10 g, 97%).

m.p. (EtOAc): 203 – 205 °C

HRMS (ESI): Exact mass calculated for $C_{11}H_{12}N$ $[M+H]^+$: 158.0964, found 158.0965.

^{1}H NMR (400 MHz, $CDCl_3$) δ 9.50 (d, *J* = 1.9 Hz, 1H, C^{2}**H**), 9.09 (s, 1H, C^{4}**H**), 8.46 (dd, *J* = 8.9, 1.0 Hz, 1H, C^{9}**H**), 8.36 (dd, *J* = 8.3, 1.4 Hz, 1H, C^{6}**H**), 8.21 (ddd, *J* = 8.7, 7.0, 1.1 Hz, 1H, C^{8}**H**), 8.02 (ddd, *J* = 8.1, 7.0, 1.0 Hz, 1H, C^{7}**H**), 4.61 (s, 3H, C^{12}**H$_3$**), 2.64 (s, 3H, C^{11}**H**).

^{13}C NMR (101 MHz, $CDCl_3$) δ 151.3 (C^{2}H), 145.5 (C^{4}H), 136.7 (C^{Ar}), 134.3 (C^{8}H), 132.0 (C^{Ar}), 129.9 (C^{7}H), 129.5 (C^{6}H), 128.9 (C), 118.8 (C^{9}H), 45.2 (C^{12}H$_3$), 17.9 (C^{11}H$_3$).

IR (neat) (cm^{-1}): 2516, 2160, 2029, 1592, 1521, 1230, 868, 773, 748, 608.

N-(4-Methoxybenzyl)-3-methylquinolinium iodide (1i)

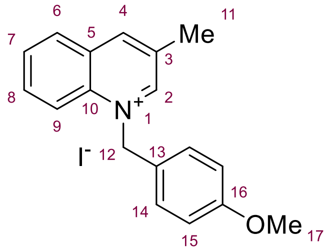

The title compound was prepared according to General Procedure **A** using 3-methylquinoline (0.27 mL, 2.0 mmol) and 4-methoxybenzyl iodide (975 mg, 3.9 mmol) in acetone (4 mL) to give salt **1i** as a yellow solid (782 mg, 99%).

m.p. (EtOAc): 190 – 192 °C

HRMS (ESI): Exact mass calculated for $C_{18}H_{18}NO$ $[M+H]^+$: 264.1383, found: 264.1385.

^{1}H NMR (400 MHz, DMSO-d_6) δ 9.72 (d, J = 1.9 Hz, 1H, C^2**H**), 9.16 (s, 1H, C^4**H**), 8.55 – 8.48 (m, 1H, C^9**H**), 8.36 (dd, J = 8.2, 1.5 Hz, 1H, C^6**H**), 8.13 (ddd, J = 8.8, 7.0, 1.5 Hz, 1H, C^8**H**), 7.97 (ddd, J = 8.0, 7.0, 0.9 Hz, 1H, C^7**H**), 7.43 (d, J = 8.8 Hz, 2H, 2 x C^{14}**H**), 6.94 (d, J = 8.8 Hz, 1H, 2 x C^{15}**H**), 6.23 (s, 2H, C^{12}**H$_2$**), 3.72 (s, 3H, C^{17}**H$_3$**), 2.70 (s, 3H, C^{11}**H$_2$**).

^{13}C NMR (101 MHz, DMSO-d_6) δ 159.5 (C^{16}), 151.0 (C^{2}H), 146.6 (C^{4}H), 135.8 (C^{Ar}), 134.5 (C^{8}H), 132.6 (C), 130.0 (C^{6}H), 129.9 (C^{7}H), 129.6 (C^{Ar}), 129.1 (2 x C^{14}H), 125.6 (C^{Ar}), 119.1 (C^{9}H), 114.4 (2 x C^{15}H), 59.6 (C^{17}H$_3$), 55.2 (C^{12}H$_2$), 18.1 (C^{11}H$_3$).

IR (neat) (cm^{-1}): 2956, 2160, 2030, 1977, 1516, 1243, 1180, 1019, 832, 805.

N-Benzyl-6-chloroquinolinium iodide (1k)

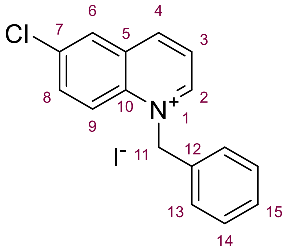

The title compound was prepared according to General Procedure **A** using 6-chloroquinoline (650 mg, 4.00 mmol) and benzyl iodide (1.00 mL, 8.00 mmol) to give salt **1l** as an orange solid (1.37 g, 90%). Spectroscopic data was consistent with that reported in the literature.[2]

^{1}H NMR (400 MHz, $CDCl_3$) δ 9.75 (dd, J = 5.8, 1.4 Hz, 1H, C^2**H**), 9.30 (dt, J = 8.5, 1.1 Hz, 1H, C^4**H**), 8.70 (d, J = 2.4 Hz, 1H, C^6**H**), 8.54 (d, J = 9.5 Hz, 1H, C^9**H**), 8.35 (dd, J = 8.5, 5.8 Hz, 1H, C^3**H**), 8.26 (dd, J = 9.4, 2.4 Hz, 1H, C^8**H**), 7.46 – 7.32 (m, 5H, 5 x C^{Ar}**H**), 6.39 (s, 2H, C^{11}**H$_2$**).

^{13}C NMR (101 MHz, $CDCl_3$) δ 150.8 (C^{2}H), 147.4 (C^{4}H), 136.3 (C^{Ar}), 135.6 (C^{8}H), 134.5 (C^{Ar}), 133.6 (C^{Ar}), 130.8 (C^{Ar}), 129.3 (C^{6}H), 129.1 (2 x C^{14}H), 128.8 (C^{15}H), 127.3 (2 x C^{13}H), 123.7 (C^{3}H), 121.6 (C^{9}H), 60.2 (C^{11}H$_2$).

N-Benzyl-7-methylquinolinium iodide (1l)

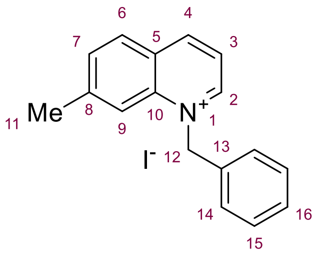

The title compound was prepared according to General Procedure **A** using 7-methylquinoline (573 mg, 4.00 mmol) and benzyl iodide (1.00 mL, 8.00 mmol) to give salt **1m** as a yellow solid (1.29 g, 91%). Spectroscopic data was consistent with that reported in the literature.[2]

^{1}H NMR (400 MHz, $CDCl_3$) δ 9.64 (dd, J = 5.9, 1.5 Hz, 1H, C^{2}**H**), 9.31 (dt, J = 8.3, 1.2 Hz, 1H, C^{4}**H**), 8.44 – 8.36 (m, 2H, C^{6}**H** + C^{9}**H**), 8.20 (dd, J = 8.3, 5.9 Hz, 1H, C^{3}**H**), 7.88 (dd, J = 8.4, 1.4 Hz, 1H, C^{7}**H**), 7.46 – 7.32 (m, 5H, 5 x C^{Ar}**H**), 6.33 (s, 2H, C^{12}**H_2**), 2.63 (d, J = 0.9 Hz, 3H, C^{11}**H_3**).

^{13}C NMR (101 MHz, $CDCl_3$) δ 149.7 (C^{2}H), 147.6 (C^{4}H), 137.9 (C^{Ar}), 133.8 (C^{Ar}), 132.0 (C^{7}H), 130.4 (C^{6}H), 129.1 (2 x C^{15}H), 128.8 (C^{Ar}/C^{16}H), 128.3 (C^{Ar}/C^{16}H), 127.4 (2 x C^{14}H), 121.4 (C^{3}H), 118.0 (C^{9}H), 59.5 ($C^{12}H_2$), 22.3 ($C^{11}H_3$).

N-Benzyl-5-nitroquinolinium iodide (1m)

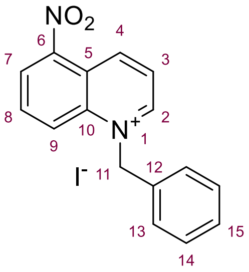

The title compound was prepared according to a modified General Procedure **A**. 5-Nitroquinoline (522 mg, 3.00 mmol) and benzyl iodide (0.75 mL, 6.00 mmol) in acetone (6.00 mL) were stirred at rt in the dark for 2 days to give salt **1k** as an orange solid (471 mg, 40%).

m.p. (acetone): 189 – 191 °C

HRMS (ESI): Exact mass calculated for $C_{16}H_{13}N_2O_2$ $[M+H]^+$: 265.0972, found: 265.0973.

^{1}H NMR (400 MHz, $CDCl_3$) δ 9.90 (dd, J = 5.8, 1.3 Hz, 1H, C^{2}**H**), 9.61 (dt, J = 9.0, 1.2 Hz, 1H, C^{4}**H**), 8.90 (dt, J = 9.2, 1.0 Hz, 1H, C^{7}**H**), 8.74 (dd, J = 7.8, 0.8 Hz, 1H, C^{9}**H**), 8.50 (dd, J = 9.0, 5.8 Hz, 1H, C^{3}**H**), 8.37 (dd, J = 9.1, 7.8 Hz, 1H, C^{8}**H**), 7.45 – 7.35 (m, 5H, 5 x C^{Ar}**H**), 6.48 (s, 2H, C^{11}**H_2**).

^{13}C NMR (101 MHz, $CDCl_3$) δ 152.0 (C^{2}H), 146.6 (C^{4}H), 137.6 (C^{Ar}), 134.2 (C^{Ar}), 133.4 (C^{8}H), 129.1 (2 x C^{14}H), 128.9 (C^{15}H), 127.4 (2 x C^{13}H), 127.1 (C^{9}H), 125.2 (C^{7}H), 125.0 (C^{3}H), 122.6 (C^{Ar}), 61.1 ($C^{11}H_2$), C^{6} missing.

IR (neat) (cm^{-1}): 2981, 2523, 2160, 2030, 1977, 1525, 1385, 1365, 1244, 746.

Synthesis of Tetrahydroquinolines

General Procedure B: Preparation of Tetrahydroquinolines

Quinolinium salt (0.125 mmol, 1.00 equiv.) and electrophile (1.00 to 5.00 equiv.) were dissolved in MeCN (0.10 mL, 1.25 M). Upon addition of HCO_2H:NEt_3 5:2 complex (42 µL, 4.00 equiv.) the reaction mixture was heated to 80 ˚C for 16 hours. The reaction was diluted with CH_2Cl_2 (10 mL) and quenched with an aqueous solution of K_2CO_3 (10 mL, 0.1 M). The phases were separated, and the aqueous layer was extracted with CH_2Cl_2 (3 x 10 mL). The organic layers were combined, dried ($MgSO_4$), filtered, and concentrated *in vacuo*. The crude material was purified by flash column chromatography to furnish the corresponding amines.

General Procedure C: Metal Catalysed Preparation of Tetrahydroquinolines

Quinolinium salt (1.00 equiv.), and $[RhCp^*Cl_2]_2$ (0.01 mol%) were added to a microwave vial. MeCN (1.25 M), electrophile (1.0 – 5.0 equiv.) and HCO_2H:NEt_3 (5:2, 4.0 equiv.) were added and the solution heated at 80 °C for 20 hours. The reaction was diluted with CH_2Cl_2 (10 mL) and quenched with an aqueous solution of K_2CO_3 (10 mL, 0.1 M). The phases were separated, and the aqueous layer was extracted with CH_2Cl_2 (3 x 10 mL). The organic layers were combined, dried ($MgSO_4$), filtered, and concentrated *in vacuo*. The crude material was purified by flash column chromatography to furnish the corresponding amines.

_N_-Benzyl-3-methyl-1,2,3,4-tetrahydroquinoline (3a)

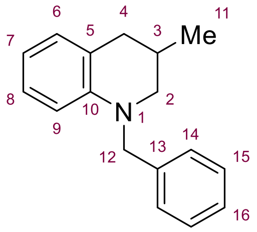

The title compound was prepared according to General Procedure **B** using salt **1a** (45 mg, 0.125 mmol) in the absence of electrophile. Purification by column chromatography (0%-2% EtOAc in pentane) gave *amine* **3a** (23 mg, 77%) as a clear oil. Spectroscopic data was consistent with that reported in the literature.[7]

HRMS (ESI): Exact mass calculated for $C_{17}H_{20}N$ $[M+H]^+$: 238.1590, found: 238.1589.

^{1}H NMR (400 MHz, $CDCl_3$) δ 7.38 – 7.22 (m, 5H, 2 x C^{14}**H** + 2 x C^{15}**H** + C^{16}**H**), 7.05 – 6.96 (m, 2H, C^6**H** + C^8**H**), 6.61 (td, *J* = 7.3, 1.1 Hz, 1H, C^7**H**), 6.57 – 6.47 (m, 1H, C^9**H**), 4.51 (s, 2H, C^{12}**H_2**), 3.31 (ddd, *J* = 11.2, 4.0, 2.2 Hz, 1H, C^2**H_2**), 3.06 (dd, *J* = 11.2, 9.6 Hz, 1H, C^2**H_2**), 2.85 (ddd, *J* = 15.6, 4.6, 2.2 Hz, 1H, C^4**H_2**), 2.53 (dd, *J* = 15.6, 10.5 Hz, 1H, C^4**H_2**), 2.28 – 2.13 (m, 1H, C^3**H**), 1.08 (d, *J* = 6.6 Hz, 3H, C^{11}**H_3**).

---

[7]Kaga, A.; Hayashi, H; Hakamata, H; Oi, M.; Uchiyama, M.; Takita, R.; Chiba, S.; *Angew.Chem. Int.Ed.* **2017**, *56*,11807 –11811

^{13}**C NMR** (101 MHz, $CDCl_3$) δ 145.3 (C^{10}), 139.2 (C^{13}), 129.3 (C^6H/C^8H), 128.7 (2 x $C^{14}H/C^{15}H$), 127.3 (C^6H/C^8H), 126.9 ($C^{16}H$), 126.7 (2 x $C^{14}H/C^{15}H$), 122.0 (C^5), 116.0 (C^7H), 110.9 (C^9H), 57.0 (C^2H_2), 55.3 ($C^{12}H_2$), 36.6 (C^4H_2), 27.5 (C^3H), 19.2 ($C^{11}H_3$).

IR (neat) (cm^{-1}): 2953, 2922, 1602, 1506, 1494, 1452, 1359, 1349, 1283, 1245.

4-(*N*-Benzyl-3-methyl-1,2,3,4-tetrahydroquinolin-3-yl)butan-2-one (2a)

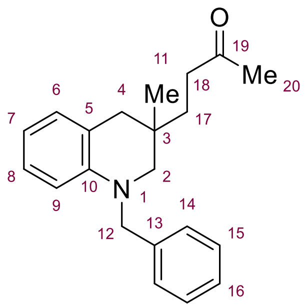

The title compound was prepared according to General Procedure **B** using salt **1a** (45 mg, 0.125 mmol) and methyl vinyl ketone (50 µL, 0.63 mmol) as electrophile. Purification by column chromatography (0%-5% EtOAc in pentane) gave *amine* **2a** (30 mg, 78%) as a clear oil.

HRMS (ESI): Exact mass calculated for $C_{21}H_{26}NO$ $[M+H]^+$: 308.2009, found: 308.2009.

^{1}**H NMR** (400 MHz, $CDCl_3$) δ 7.36 – 7.20 (m, 5H, 2 x C^{14}**H** + 2 x C^{15}**H** + C^{16}**H**), 7.04 – 6.93 (m, 2H, C^6**H** + C^8**H**), 6.61 (t, *J* = 7.3 Hz, 1H, C^7**H**), 6.56 (d, *J* = 8.2 Hz, 1H, C^9**H**), 4.48 (d, *J* = 3.3 Hz, 2H, C^2**H₂**), 3.08 – 2.96 (m, 2H, C^4**H₂**), 2.59 (s, 2H, C^{12}**H₂**), 2.50 – 2.31 (m, 2H, C^{18}**H₂**), 2.11 (s, 3H, C^{20}**H₃**), 1.71 – 1.53 (m, 2H, C^{17}**H₂**), 0.97 (s, 3H, C^{11}**H₃**).

^{13}**C NMR** (101 MHz, $CDCl_3$) δ 208.9 (C^{19}), 144.7 (C^{10}), 139.2 (C^{13}), 129.9 (C^6H/C^8H), 128.7 (2 x $C^{14}H/C^{15}H$), 127.3 (C^6H/C^8H), 127.0 ($C^{16}H$), 126.8 (2 x $C^{14}H/C^{15}H$), 120.6 (C^5), 116.5 (C^7H), 110.9 (C^9H), 59.8 (C^2H_2), 55.5 ($C^{12}H_2$), 40.7 (C^4H_2), 38.4 ($C^{18}H_2$), 32.5 ($C^{17}H_2$), 30.8 (C^3), 30.1 ($C^{20}H_3$), 23.5 ($C^{11}H_3$).

IR (neat) (cm^{-1}): 2919, 2834, 1714, 1602, 1504, 1497, 1451, 1352, 1282, 1249.

3-(*N* -Benzyl-3-methyl-1,2,3,4-tetrahydroquinolin-3-yl)-1-phenylpropan-1-one (2b)

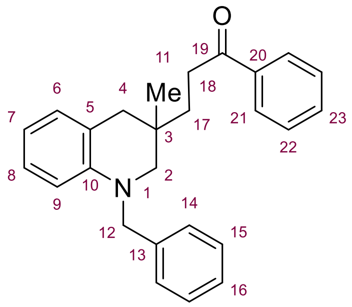

The title compound was prepared according to General Procedure **B** using salt **1a** (45 mg, 0.125 mmol) and phenyl vinyl ketone (33 mg, 0.25 mmol) as electrophile. Purification by column chromatography (0%-5% EtOAc in pentane) gave *amine* **2b** (37 mg, 77%) as a clear oil.

HRMS (ESI): Exact mass calculated for $C_{26}H_{28}NO$ $[M+H]^+$: 370.2165, found: 360.2168.

^{1}**H NMR** (400 MHz, $CDCl_3$) δ 7.92 – 7.82 (m, 2H, 2 x C^{21}**H**), 7.60 – 7.48 (m, 1H, C^{23}**H**), 7.42 (dd, *J* = 8.4, 7.1 Hz, 2H, 2 x C^{22}**H**), 7.32 – 7.16 (m, 5H, 2 x C^{14}**H** + 2 x C^{15}**H** + C^{16}**H**), 7.04 – 6.96 (m, 2H, C^6**H** + C^8**H**), 6.61 (t, *J* = 1.1 Hz, 1H, C^7**H**), 6.56 (d, *J* = 8.1 Hz, 1H, C^9**H**), 4.55 – 4.42 (m, 2H, C^{12}**H₂**), 3.15 – 3.03 (m, 2H, C^2**H₂**), 3.03 – 2.82 (m, 2H, C^{18}**H₂**), 2.73 – 2.57 (m, 2H, C^4**H₂**), 1.79 (t, *J* = 8.2 Hz, 2H, C^{17}**H₂**), 1.05 (s, 3H, C^{11}**H₃**).

^{13}C NMR (101 MHz, $CDCl_3$) δ 200.5 (C^{19}), 144.8 (C^{10}), 139.2 (C^{13}), 137.1 (C^{20}), 133.1 (C^{23}H), 129.9 (C^6H/C^8H), 128.72 (2 x C^{15}H/C^{22}H), 128.69 (2 x C^{15}H/C^{22}H), 128.2 (2 x C^{21}H), 127.4 (C^6H/C^8H), 127.0 (C^{16}), 126.9 (2 x C^{14}H), 120.7 (C^5), 116.5 (C^7H), 111.0 (C^9H), 60.0 (C^2H_3), 55.6 ($C^{12}H_3$), 40.7 (C^4H_3), 33.33 ($C^{17}H_2$/$C^{18}H_2$), 33.31 ($C^{17}H_2$/$C^{18}H_2$), 31.1 (C^3), 23.6 ($C^{11}H_3$).

IR (neat) (cm^{-1}): 2981, 2895, 1683, 1601, 1505, 1450, 1283, 1249, 743, 692.

3-(*N*-Benzyl-3-methyl-1,2,3,4-tetrahydroquinolin-3-yl)-1-(2,3,4,5,6-pentamethylphenyl)propan-1-one (2c)

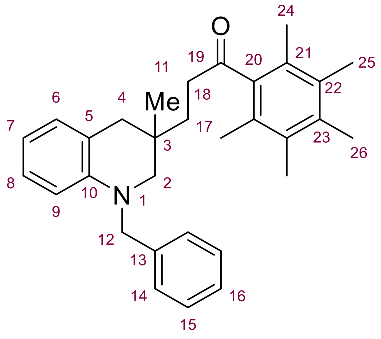

The title compound was prepared according to General Procedure **B** using salt **1a** (45 mg, 0.125 mmol) and 2,3,4,5,6-pentamethylphenyl vinyl ketone (51 mg, 0.25 mmol) as electrophile. Purification by column chromatography (0%-5% EtOAc in pentane) gave *amine* **2c** (35 mg, 65%) as a white solid.

m.p. (EtOAc): 112 – 114 °C

HRMS (ESI): Exact mass calculated for $C_{31}H_{38}NO$ $[M+H]^+$: 440.2948, found: 440.2942.

^{1}H NMR (400 MHz, $CDCl_3$) δ 7.35 – 7.21 (m, 5H, 2 x C^{14}**H** + 2 x C^{15}**H** + C^{16}**H**), 7.05 – 6.98 (m, 1H, C^8**H**), 6.96 (dd, *J* = 7.4, 1.6 Hz, 1H, C^6**H**), 6.61 (td, *J* = 7.3, 1.1 Hz, 1H, C^7**H**), 6.55 (dd, *J* = 8.2, 1.1 Hz, 1H, C^9**H**), 4.58 – 4.37 (m, 2H, C^{12}**H₂**), 3.13 – 2.99 (m, 2H, C^2**H₂**), 2.76 – 2.55 (m, 4H, C^4**H₂** + C^{18}**H₂**), 2.24 (s, 3H, C^{26}**H₃**), 2.19 (s, 6H, 2 x C^{24}**H₃**/C^{25}**H₃**), 2.07 (s, 6H, 2 x C^{24}**H₃**/C^{25}**H₃**), 1.88 – 1.74 (m, 2H, C^{17}**H₂**), 1.01 (s, 3H, C^{11}**H₃**).

^{13}C NMR (101 MHz, $CDCl_3$) δ 212.1 (C^{19}), 144.8 (C^{10}), 141.1 (C^{Ar}), 139.2 (C^{Ar}), 135.6 (C^{Ar}), 133.2 (2 x C^{Ar}), 129.8 (C^6H), 128.7 (2 x C^{14}H/C^{15}H), 127.4 (C^8H), 127.3 (2 x C^{Ar}), 127.0 (C^{16}H), 126.7 (2 x C^{14}H/C^{15}H), 120.6 (C^5), 116.5 (C^7H), 111.0 (C^9H), 60.2 (C^2H_2), 55.7 ($C^{12}H_2$), 40.6 (C^4H_2/$C^{18}H_2$), 40.3 (C^4H_2/$C^{18}H_2$), 32.4 ($C^{17}H_2$), 30.9 (C^3), 23.3 ($C^{11}H_3$), 17.4 (2 x $C^{24}H_3$/$C^{25}H_3$), 16.8 ($C^{26}H_3$), 16.1 (2 x $C^{24}H_3$/$C^{25}H_3$).

IR (neat) (cm^{-1}): 2922, 1700, 1603, 1506, 1497, 1452, 1357, 1317, 1283, 1249, 745.

Dimethyl 2-((*N*-benzyl-3-methyl-1,2,3,4-tetrahydroquinolin-3-yl)methyl)malonate (2d)

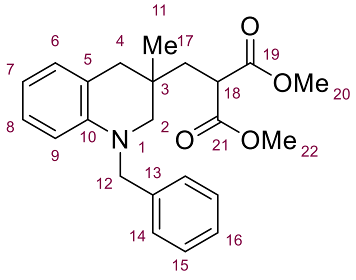

The title compound was prepared according to a modified General Procedure **B** using salt **1a** (45 mg, 0.125 mmol) and dimethyl 2-methylenemalonate (90 mg, 0.625 mmol) as electrophile. Purification by column chromatography (0%-5% EtOAc in pentane) gave *amine* **2d** (15 mg, 32%) as a clear oil.

HRMS (ESI): Exact mass calculated for $C_{23}H_{28}NO_4$ $[M+H]^+$: 382.2013, found: 382.2013.

^{1}H NMR (400 MHz, $CDCl_3$) δ 7.30 – 7.09 (m, 5H, 5 x C^{Ar}**H**), 6.92 (ddd, *J* = 8.2, 7.3, 1.7 Hz, 1H, C^{8}**H**), 6.87 (dd, *J* = 7.4, 1.6 Hz, 1H, C^{6}**H**), 6.52 (ddd, *J* = 7.3, 7.3, 1.1 Hz, 1H, C^{7}**H**), 6.48 (dd, *J* = 8.2, 1.1 Hz, 1H, C^{9}**H**), 4.40 (dd, *J* = 16.9, 16.9 Hz, 2H, C^{12}**H$_2$**), 3.63 (s, 6H, C^{20}**H$_3$** + C^{22}**H$_3$**), 3.40 (t, *J* = 6.5 Hz, 1H, C^{18}**H**), 3.08 – 2.82 (m, 2H, C^{4}**H$_2$**), 2.66 – 2.39 (m, 2H, C^{2}**H$_2$**), 2.07 – 1.93 (m, 2H, C^{17}**H$_2$**), 0.89 (s, 3H, C^{11}**H$_3$**).

^{13}C NMR (101 MHz, $CDCl_3$) δ 170.49 (C^{19}O/C^{21}O), 170.45 (C^{19}O/C^{21}O), 144.5 (C^{Ar}), 139.0 (C^{Ar}), 129.9 (C^{6}H), 128.7 (2 x C^{14}H/C^{15}H), 127.4 (C^{8}H), 127.0 (C^{16}H), 126.8 (2 x C^{14}H/C^{15}H), 120.2 (C^{Ar}), 116.6 (C^{7}H), 111.1 (C^{9}H), 59.8 (C^{2}H$_2$), 55.6 (C^{12}H$_2$), 52.82 (C^{20}H$_3$/C^{22}H$_3$), 52.81 (C^{20}H$_3$/C^{22}H$_3$), 47.8 (C^{18}H), 40.3 (C^{4}H$_2$), 37.8 (C^{17}H), 31.3 (C^{3}), 23.1 (C^{11}H$_3$).

IR (neat) (cm^{-1}): 2953, 1753, 1735, 1603, 1576, 1502, 1452, 1436, 1379, 1352.

***N*-Benzyl-3-(2,2-bis(phenylsulfonyl)ethyl)-3-methyl-1,2,3,4-tetrahydroquinoline (2e)**

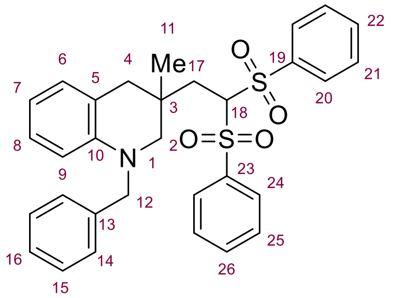

The title compound was prepared according to a modified General Procedure **B** using salt **1a** (45 mg, 0.125 mmol) and 1,1-bis(phenylsulfonyl)ethylene (154 mg, 0.5 mmol) as electrophile and HCO_2H:NEt_3 5:2 (84 µL, 1.0 mmol) as the terminal reductant. Purification by column chromatography (0%-5% EtOAc in pentane) gave *amine* **2e** (42 mg, 62%) as a white solid.

m.p. (EtOAc): 169 – 171 °C

HRMS (ESI): Exact mass calculated for $C_{31}H_{32}NO_4S_2$ $[M+H]^+$: 546.1767, found: 546.1768.

^{1}H NMR (400 MHz, $CDCl_3$) δ 7.95 – 7.87 (m, 2H, 2 x C^{Ar}**H**), 7.74 – 7.59 (m, 4H, 4 x C^{Ar}**H**), 7.58 – 7.51 (m, 2H, 2 x C^{Ar}**H**), 7.49 – 7.39 (m, 2H, 2 x C^{Ar}**H**), 7.30 – 7.19 (m, 3H, 3 x C^{Ar}**H**), 7.16 – 6.99 (m, 4H, 2 x C^{Ar}**H** + C^{6}**H** + C^{8}**H**), 6.69 (td, *J* = 7.4, 1.1 Hz, 1H, C^{7}**H**), 6.59 (dd, *J* = 8.3, 1.1 Hz, 1H, C^{9}**H**), 4.59 (t, *J* = 4.0 Hz, 1H, C^{18}**H**), 4.46 (q, *J* = 17.0 Hz, 2H, C^{12}**H$_2$**), 3.14 (dd, *J* = 11.7, 1.2 Hz, 1H, C^{2}**H$_2$**), 3.03 (dd, *J* = 11.6, 2.1 Hz, 1H, C^{2}**H$_2$**), 2.81 (dd, *J* = 16.1, 2.1 Hz, 1H, C^{4}**H$_2$**), 2.57 (d, *J* = 16.1 Hz, 1H, C^{4}**H$_2$**), 2.35 – 2.18 (m, 2H, C^{17}**H$_2$**), 1.12 (s, 3H, C^{11}**H$_3$**).

^{13}C NMR (101 MHz, $CDCl_3$) δ 144.3 (C^{10}), 138.8 (C^{Ar}), 138.3 (C^{Ar}), 136.7 (C^{Ar}), 134.6 (C^{Ar}H), 134.5 (C^{Ar}H), 130.8 (C^{Ar}H), 130.5 (2 x C^{Ar}H), 129.9 (2 x C^{Ar}H), 129.2 (2 x C^{Ar}H), 129.0 (2 x C^{Ar}H), 128.7 (2 x C^{Ar}H), 127.5 (C^{Ar}H), 127.0 (C^{Ar}H), 126.7 (2 x C^{Ar}H), 119.9 (C^{Ar}), 116.9 (C^{7}H), 111.1 (C^{9}H), 81.1 (C^{18}H), 60.3 (C^{2}H$_2$), 55.3 (C^{12}H$_2$), 39.2 (C^{4}H$_2$), 32.2 (C^{3}), 31.8 (C^{17}H$_2$), 24.2 (C^{11}H$_3$).

IR (neat) (cm^{-1}): 3063, 2924, 1669, 1602, 1500, 1448, 1329, 1287, 1249, 1194.

3-(*N*-Benzyl-3-methyl-1,2,3,4-tetrahydroquinolin-3-yl)-*N*-methylpyrrolidine-2,5-dione (2f)

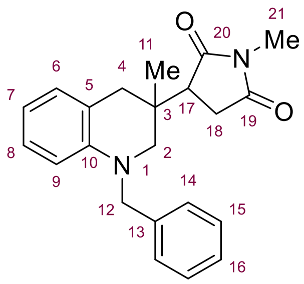

The title compound was prepared according to General Procedure **B** using salt **1a** (45 mg, 0.125 mmol) and *N*-methylmaleimide (28 mg, 0.25 mmol) as electrophile. Purification by column chromatography (0%-5% EtOAc in pentane) gave *amine* **2f** as an inseparable 2:1 diastereomeric mixture (36 mg, 82%) as a clear oil.

HRMS (ESI): Exact mass calculated for $C_{22}H_{25}N_2O_2$ [M+H]$^+$: 349.1911, found: 349.1912

^{1}**H NMR** (400 MHz, $CDCl_3$) δ 7.41 – 7.15 (m, 10H, 5 x C^{Ar}**H**, *A* + *B*), 7.09 –6.82 (m, 4H, C^{8}**H**, *A+B*, C^{8}**H**, *A+B*), 6.72 – 6.53 (m, 4H, C^{9}**H**, *A+B*, C^{7}**H**, *A+B*), 4.75 – 4.62 (m, 1H, C^{12}**H$_2$**, *B*), 4.57 – 4.47 (m, 2H, C^{12}**H$_2$**, *A+B*), 4.39 (d, *J* = 16.6 Hz, 1H, C^{12}**H$_2$**, *A*), 3.86 (dd, *J* = 11.9, 2.2 Hz, 1H, C^{2}**H$_2$**, *B*), 3.54 – 3.42 (m, 2H, C^{4}**H$_2$**, *A*, C^{2}**H$_2$**, *B*), 3.12 – 2.99 (m, 3H, C^{17}**H**, *B*, C^{2}**H$_2$**, *A*), 3.00 – 2.91 (m, 7H, C^{21}**H$_3$**, *A+B*, C^{17}**H**, *A*), 2.84 (d, *J* = 16.1 Hz, 1H, C^{4}**H$_2$**, *A*), 2.82 – 2.59 (m, 2H, C^{4}H$_2$, *B*), 2.58 – 2.48 (m, 2H, C^{18}**H$_2$**, *A+B*), 2.45 – 2.30 (m, 2H, C^{18}**H$_2$**, *A+B*), 0.95 (s, 3H, C^{11}**H$_3$**, *B*), 0.94 (s, 3H, C^{11}**H$_3$,** *A*).

^{13}**C NMR** (101 MHz, $CDCl_3$) δ 178.8 (C^{19}O/C^{20}O, *B*), 178.6 (C^{19}O/C^{20}O, *A*), 176.5 (C^{19}O/C^{20}O, *B*), 176.4 (C^{19}O/C^{20}O, *A*), 145.7 (C^{10}, *B*), 144.8 (C^{10}, *A*), 139.0 (C^{13}, *B*), 138.7 (C^{13}, *A*), 130.5 (C^{8}H, *A*), 129.8 (C^{8}H, *B*), 128.9 (2 x C^{Ar}H, *A/B*), 128.8 (C^{Ar}H, *A/B*), 128.7 (C^{Ar}H, *A/B*), 128.4 (C^{Ar}H, *A/B*), 127.9 (C^{Ar}H, *A/B*), 127.6 (C^{Ar}H, *A/B*), 127.4 (C^{Ar}H, *A/B*), 127.0 (2 x C^{Ar}H, *A/B*), 120.5 (C^{5}, *A*), 120.2 (C^{8}H, *B*), 118.7 (C^{5}, *B*), 117.5 (C^{7}H, *A*), 116.6 (C^{7}H, *B*), 114.8 (C^{8}H, *B*), 111.2 (C^{9}H, *B*), 111.1 (C^{9}H, *A*), 64.3, 58.7 (C^{2}H$_2$, *A*), 58.3 (C^{2}H$_2$, *B*), 55.6 (C^{12}H$_2$, *B*), 55.3 (C^{12}H$_2$, *A*), 44.0 (C^{17}H, *A*), 43.8 (C^{17}H, *B*), 40.3 (C^{4}H$_2$, *B*), 38.8 (C^{4}H$_2$, *A*), 34.3 (C^{3}, *A*), 33.9 (C^{3}, *B*), 30.9 (C^{18}H$_2$, *A*), 30.7 (C^{18}H$_2$, *B*), 24.83 (C^{21}H$_3$, *B*), 24.80 (C^{21}H$_3$, *A*), 19.5 (C^{11}H$_3$, *A*), 19.2 (C^{11}H$_3$, *B*).

IR (neat) (cm^{-1}): 2956, 1771, 1694, 1602, 1498, 1435, 1382, 1355, 1281, 1123.

3-(*N*-Benzyl-3-methyl-1,2,3,4-tetrahydroquinolin-3-yl)-*N*-phenylpyrrolidine-2,5-dione (2g)

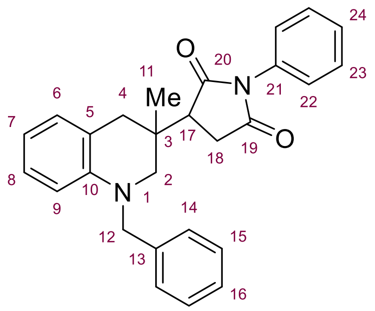

The title compound was prepared according to General Procedure **B** using salt **1a** (45 mg, 0.125 mmol) and *N*-phenylmaleimide (43 mg, 0.25 mmol) as electrophile. Purification by column chromatography (0%-5% EtOAc in pentane) gave *amine* **2g** as an inseparable 1.5:1 diastereomeric mixture (38 mg, 73%) as a white wax.

HRMS (ESI): Exact mass calculated for $C_{27}H_{27}N_2O_2$ [M+H]$^+$: 411.2067, found: 411.2067.

^{1}**H NMR** (400 MHz, $CDCl_3$, *A* = major diastereomer) δ 7.54 – 7.20 (m, 20H, 20 x C^{Ar}**H**, *A* + *B*), 7.13 – 7.01 (m, 3H, C^{6}**H** + C^{8}**H**, *A*, C^{6}**H**/C^{8}**H**, *B*), 6.98 (dd, *J* = 7.4, 1.6 Hz, 1H, C^{6}**H**/C^{8}**H**, *B*), 6.70 (t, *J* = 7.2 Hz, 2H, C^{7}**H**,

A + *B*), 6.66 – 6.58 (m, 2H, $C^{9}H$, *A* + *B*), 4.74 (d, *J* = 17.1 Hz, 1H, $C^{12}H_2$, *B*), 4.61 – 4.36 (m, 3H, $C^{12}H_2$, *A* + *B*), 3.92 (dd, *J* = 12.0, 2.3 Hz, 1H, $C^{2}H_2$, *B*), 3.57 (dd, *J* = 16.2, 2.3 Hz, 1H, $C^{4}H_2$, *A*), 3.50 (d, *J* = 11.9 Hz, 1H, $C^{2}H_2$, *B*), 3.22 (dd, *J* = 8.9, 5.8 Hz, 1H, $C^{17}H$, *B*), 3.18 – 3.06 (m, 3H, $C^{2}H_2$, *A*, $C^{17}H$, *A*), 2.94 – 2.63 (m, 6H, $C^{4}H_2$, *A* + *B*, $C^{18}H_2$, *A*+*B*), 2.61 – 2.52 (m, 1H, $C^{18}H_2$, *A*), 1.09 (s, 3H, $C^{11}H_3$, *B*), 1.07 (s, 3H, $C^{11}H_3$, *A*).

^{13}C NMR (101 MHz, $CDCl_3$) δ 177.8 (C^{19}/C^{20}, *B*), 177.5 (C^{19}/C^{20}, *A*), 175.4 (C^{19}/C^{20}, *B*), 175.4 (C^{19}/C^{20}, *A*), 144.9 (C^{10}, *B*), 144.8 (C^{10}, *A*), 139.0 (C^{13}, *B*), 138.7 (C^{13}, *A*), 134.3 (C^{21}, *B*), 132.0 (C^{21}, *A*), 130.6 ($C^{6}H/C^{8}H$, *A*), 129.8 ($C^{6}H/C^{8}H$, *B*), 129.31 ($C^{Ar}H$, *A/B*), 129.26 ($C^{Ar}H$, *A/B*), 128.9 ($C^{Ar}H$, *A/B*), 128.8 ($C^{Ar}H$, *A/B*), 128.74 ($C^{Ar}H$, *A/B*), 128.69 ($C^{Ar}H$, *A/B*), 128.0 ($C^{Ar}H$, *A/B*), 127.6 ($C^{Ar}H$, *A/B*), 127.4 ($C^{Ar}H$, *A/B*), 127.0 ($C^{Ar}H$, *A/B*), 126.9 ($C^{Ar}H$, *A/B*), 126.8 ($C^{Ar}H$, *A/B*), 126.6 ($C^{Ar}H$, *A/B*), 120.2 (C^{5}, *A*), 118.6 (C^{5}, *B*), 117.53 ($C^{7}H$, *A*), 116.65 ($C^{7}H$, *B*), 111.2 ($C^{9}H$, *A*), 111.1 ($C^{9}H$, *B*), 58.8 ($C^{2}H_2$, *A*), 58.2 ($C^{2}H_2$, *B*), 55.4 ($C^{12}H_2$, *B*), 55.3 ($C^{12}H_2$, *A*), 43.7 ($C^{17}H$, *A*), 43.5 ($C^{17}H$, *B*), 40.4 ($C^{4}H_2$, *B*), 38.8 ($C^{4}H_2$, *A*), 34.7(C^{3}, *A*), 34.2 (C^{3}, *B*), 31.1 ($C^{18}H_2$, *A*), 31.0 ($C^{18}H_2$, *B*), 19.5 ($C^{11}H_3$, *A*), 19.2 ($C^{11}H_3$, *B*).

IR (neat) (cm^{-1}): 2929, 1774, 1707, 1600, 1499. 1453, 1381, 1286, 1180, 1028.

3-(*N*-Benzyl-3-methyl-1,2,3,4-tetrahydroquinolin-3-yl)pyrrolidine-2,5-dione (2h)

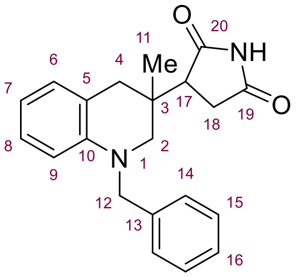

The title compound was prepared according to General Procedure **B** using salt **1a** (45 mg, 0.125 mmol) and maleimide (24 mg, 0.25 mmol) as electrophile. Purification by column chromatography (0%-5% EtOAc in pentane) gave *amine* **2h** as an inseparable 1.7:1 diastereomeric mixture (28 mg, 67%) as a clear oil.

HRMS (ESI): Exact mass calculated for $C_{21}H_{23}N_2O_2$ $[M+H]^+$: 335.1754, found: 335.1754.

^{1}H NMR (400 MHz, $CDCl_3$) δ 8.44 – 8.14 (m, 2H, NH, *A* + *B*), 7.34 – 7.07 (m, 10H, 10 x $C^{Ar}H$, *A* + *B*), 7.04 – 6.74 (m, 4H, $C^{6}H$ + $C^{8}H$, *A* + *B*), 6.63 – 6.45 (m, 4H, $C^{7}H$ + $C^{9}H$, *A* + *B*), 4.68 – 4.23 (m, 4H, $C^{12}H_2$, *A* + *B*), 3.75 (dd, *J* = 11.9, 2.3 Hz, 1H, $C^{4}H_2$, *B*), 3.43 – 3.31 (m, 2H, $C^{2}H_2$, *A*, $C^{4}H_2$, *B*), 3.04 – 2.98 (m, 1H, $C^{17}H$,*B*), 2.98 – 2.89 (m, 3H, $C^{4}H_2$ + $C^{17}H$, *A*), 2.75 (d, *J* = 16.2 Hz, 1H, $C^{2}H_2$, *A*), 2.70 – 2.41 (m, 4H, $C^{2}H_2$, *B*, $C^{18}H_2$, *A* + *B*), 2.39 – 2.29 (m, 1H, $C^{18}H_2$, *A*), 0.95 (s, 3H, $C^{11}H_3$, *B*), 0.93 (s, 3H, $C^{11}H_3$, *A*).

Major diastereoisomer A: **^{13}C NMR** (101 MHz, $CDCl_3$) δ 178.9 ($C^{19}O$/ $C^{20}O$), 176.45 ($C^{19}O$/ $C^{20}O$), 144.79 (C^{10}), 138.6 (C^{13}), 130.6 ($C^{8}H$), 128.9 (2 x $C^{14}H/C^{15}H$), 127.6 ($C^{6}H$), 127.4 ($C^{16}H$), 127.02 (2 x $C^{14}H/C^{15}H$), 120.2 (C^{5}), 117.5 ($C^{7}H$), 111.17 ($C^{9}H$), 58.6 ($C^{2}H_2$), 55.3 ($C^{12}H_2$), 45.2 ($C^{17}H$), 38.7 ($C^{4}H_2$), 34.3 (C^{3}), 32.0 ($C^{18}H_2$), 19.5 ($C^{11}H_3$).

Minor diastereoisomer B: **^{13}C NMR** (101 MHz, $CDCl_3$) δ 179.0 ($C^{19}O$/ $C^{20}O$), 176.50 ($C^{19}O$/ $C^{20}O$), 144.83 (C^{10}), 139.0 (C^{13}), 129.8 ($C^{8}H$), 128.7 (2 x $C^{14}H/C^{15}H$), 127.9 ($C^{6}H$), 126.95 ($C^{16}H$), 126.8 (2 x $C^{14}H/C^{15}H$),

118.6 (C^{5}), 116.7 (C^{7}H), 111.21 (C^{9}H), 58.1 (C^{2}H$_2$), 55.5 (C^{12}H), 44.9 (C^{17}H), 40.2 (C^{4}H$_2$), 33.9 (C^{3}), 31.9 (C^{18}H$_2$), 19.3 (C^{11}H$_3$).

IR (neat) (cm^{-1}): 3221, 1776, 1701, 1600, 1497, 1453, 1350, 1179, 908, 727.

4-(*N*-Benzyl-3-isopropyl-1,2,3,4-tetrahydroquinolin-3-yl)butan-2-one (2i)

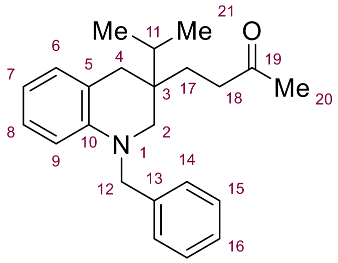

The title compound was prepared according to General Procedure **B** using salt **1b** (45 mg, 0.125 mmol) and methyl vinyl ketone (50 µL, 0.63 mmol) as electrophile. Purification by column chromatography (0%-5% EtOAc in pentane) gave *amine* **2i** (25 mg, 60%) as a yellow oil.

HRMS (ESI): Exact mass calculated for $C_{23}H_{30}NO$ $[M+H]^+$: 336.2322, found: 336.2320.

^{1}**H NMR** (400 MHz, CDCl$_3$) δ 7.36 – 7.20 (m, 5H, 2 x C^{14}**H** + 2 x C^{15}**H** + C^{16}**H**), 7.04 – 6.96 (m, 2H, C^{6}**H** + C^{8}**H**), 6.61 (td, *J* = 7.3, 1.1 Hz, 1H, C^{7}**H**), 6.57 (dd, *J* = 8.7, 1.1 Hz, 1H, C^{9}**H**), 4.52 (d, *J* = 16.8 Hz, 1H, C^{12}**H$_2$**), 4.42 (d, *J* = 16.8 Hz, 1H, C^{12}**H$_2$**), 3.19 (dd, *J* = 11.6, 1.0 Hz, 1H, C^{2}**H$_2$**), 2.93 (dd, *J* = 11.6, 2.1 Hz, 1H, C^{11}**H$_2$**), 2.79 (d, *J* = 15.9 Hz, 1H, C^{4}**H$_2$**), 2.48 (dd, *J* = 16.0, 2.1 Hz, 1H, C^{4}**H$_2$**), 2.42 – 2.22 (m, 2H, C^{18}**H$_2$**), 2.05 (s, 3H, C^{20}**H$_3$**), 1.80 – 1.53 (m, 3H, C^{11}**H** + C^{17}**H$_2$**), 0.90 (dd, *J* = 6.9, 2.0 Hz, 6H, 2 x C^{21}**H$_3$**).

^{13}**C NMR** (101 MHz, CDCl$_3$) δ 209.0 (C^{19}), 145.2 (C^{10}), 139.2 (C^{13}), 130.0 (C^{6}H/C^{8}H), 128.7 (2 x C^{14}H/C^{15}H), 127.1 (C^{6}H/C^{8}H/C^{15}H), 127.01 (C^{6}H/C^{8}H/C^{15}H), 126.97 (2 x C^{14}H/C^{15}H), 121.2 (C^{5}), 116.7 (C^{7}H), 111.0 (C^{9}H), 55.7 (C^{2}H$_2$/C^{12}H$_2$), 55.6 (C^{2}H$_2$/C^{12}H$_2$), 38.4 (C^{4}H$_2$), 35.4 (C^{3} + C^{18}H$_2$), 35.2 (C^{3} + C^{18}H$_2$), 31.4 (C^{11}H), 30.0 (C^{20}H$_3$), 26.2 (C^{17}H$_2$), 17.3 (C^{21}H$_3$), 17.0 (C^{21}H$_3$).

IR (neat) (cm^{-1}): 3027, 2959, 1715, 1602, 1576, 1506, 1452, 1387, 1354, 1280.

4-(*N*-Benzyl-3-benzyl-1,2,3,4-tetrahydroquinolin-3-yl)butan-2-one (2j)

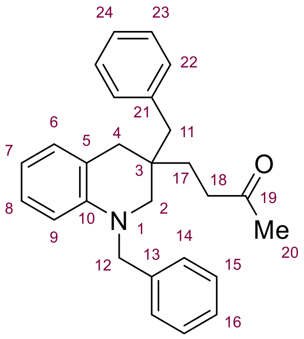

The title compound was prepared according to General Procedure **B** using salt **1c** (45 mg, 0.125 mmol) and methyl vinyl ketone (50 µL, 0.63 mmol) as electrophile. Purification by column chromatography (0%-5% EtOAc in pentane) gave *amine* **2j** (43 mg, 90%) as a yellow oil.

HRMS (ESI): Exact mass calculated for $C_{27}H_{30}NO$ $[M+H]^+$: 384.2322, found: 384.2322.

^{1}**H NMR** (400 MHz, CDCl$_3$) δ 7.38 – 7.18 (m, 8H, 8 x C^{Ar}**H**), 7.12 – 6.96 (m, 4H, 4 x C^{Ar}**H**), 6.68 – 6.59 (m, 2H, C^{7}**H** + C^{9}**H**), 4.60 (d, *J* = 16.9 Hz, 1H, C^{12}**H$_2$**), 4.42 (d, *J* = 16.9 Hz, 1H, C^{12}**H$_2$**), 3.07 (brs, *J* = 1.5 Hz, 2H, C^{2}**H$_2$**), 2.74 – 2.54 (m, 4H, C^{4}**H$_2$+** C^{11}**H$_2$**), 2.54 – 2.32 (m, 2H, C^{18}**H$_2$**), 2.09 (s, 3H, C^{20}**H$_3$**), 1.73 – 1.48 (m, 2H, C^{17}**H$_2$**).

^{13}**C NMR** (101 MHz, CDCl$_3$) δ 208.6 (C^{19}), 144.6 (C^{10}), 138.9 (C^{13}), 137.7 (C^{Ar}), 130.6 (2 x C^{Ar}H), 130.1 (C^{Ar}H), 128.7 (2 x C^{Ar}H), 128.2 (2 x C^{Ar}H), 127.4 (C^{Ar}H), 127.04 (C^{Ar}H), 126.99 (2 x C^{Ar}H), 126.5 (C^{Ar}H),

120.3 (C^5), 116.7 (C^7H/C^9H), 111.1 (C^7H/C^9H), 57.8 (C^2H_2), 55.5 ($C^{12}H_2$), 41.7 ($C^4H_2/C^{11}H_2$), 38.3 ($C^4H_2/C^{11}H_2$), 38.1 ($C^{18}H_2$), 34.6 (C^3), 30.1 ($C^{20}H_3$), 29.0 ($C^{17}H_2$).

IR (neat) (cm^{-1}): 3061, 3027, 2920, 1714, 1602, 1576, 1499, 1452, 1354, 1286.

Methyl 3-(*N*-benzyl-3-(3-oxobutyl)-1,2,3,4-tetrahydroquinolin-3-yl)propanoate (2k)

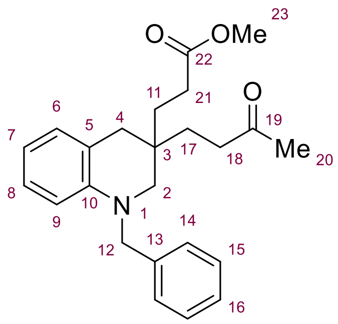

The title compound was prepared according to General Procedure **B** using salt **1d** (45 mg, 0.125 mmol) and methyl vinyl ketone (50 µL, 0.63 mmol) as electrophile. Purification by column chromatography (0%-5% EtOAc in pentane) gave *amine* **2k** (39 mg, 82%) as a clear oil.

HRMS (ESI): Exact mass calculated for $C_{24}H_{30}NO_3$ $[M+H]^+$: 380.2220, found: 380.2219.

^{1}H NMR (400 MHz, $CDCl_3$) δ 7.36 – 7.20 (m, 5H, 2 x $C^{14}\mathbf{H}$ + 2 x $C^{15}\mathbf{H}$ + $C^{16}\mathbf{H}$), 7.05 – 6.93 (m, 2H, $C^6\mathbf{H}$ + $C^8\mathbf{H}$), 6.66 – 6.56 (m, 2H, $C^7\mathbf{H}$ + $C^9\mathbf{H}$), 4.57 – 4.34 (m, 2H, $C^{12}\mathbf{H_2}$), 3.65 (s, 3H, $C^{23}\mathbf{H_3}$), 3.01 (s, 2H, $C^4\mathbf{H_2}$), 2.62 (s, 2H, $C^4\mathbf{H_2}$), 2.48 – 2.15 (m, 4H, $C^{18}\mathbf{H_2}$ + $C^{21}\mathbf{H_2}$), 2.10 (s, 3H, $C^{20}\mathbf{H_3}$), 1.79 – 1.47 (m, 4H, $C^{11}\mathbf{H_2}$ + $C^{17}\mathbf{H_2}$).

^{13}C NMR (101 MHz, $CDCl_3$) δ 208.4 ($C^{19}O$), 174.2 ($C^{20}O$), 144.8 (C^{10}), 138.9 (C^5), 130.0 (C^6H/C^8H), 128.7 (2 x $C^{14}H/C^{15}H$), 127.4 (C^6H/C^8H), 127.1 ($C^{16}H$), 126.9 (2 x $C^{14}H/C^{15}H$), 120.0 (C^{13}), 116.8 (C^7H/C^9H), 111.1 (C^7H/C^9H), 57.6 (C^2H_2), 55.4 ($C^{12}H_2$), 51.8 ($C^{23}H_3$), 39.0 (C^4H_2), 37.6 ($C^{18}H_2/C^{21}H_2$), 32.9 (C^3), 30.1 ($C^{20}H_3$), 29.9 ($C^{11}H_2/C^{17}H_2$), 28.4 ($C^{18}H_2/C^{21}H_2$), 28.3 ($C^{11}H_2/C^{17}H_2$).

IR (neat) (cm^{-1}): 3027, 2925, 1734, 1715, 1602, 1502, 1452, 1436, 1354, 1320.

3-(*N*-Benzyl-2,3-dimethyl-1,2,3,4-tetrahydroquinolin-3-yl)-1-(2,3,4,5,6-pentamethylphenyl)propan-1-one (2l)

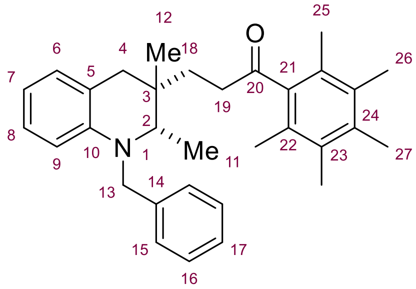

The title compound was prepared according to General Procedure **C** using salt **1f** (45 mg, 0.125 mmol) and 2,3,4,5,6-pentamethylphenyl vinyl ketone (51 mg, 0.25 mmol) as electrophile. Purification by column chromatography (0%-5% EtOAc in pentane) gave *amine* **2l** (22 mg, 32%) as an inseparable 2:1 diastereomeric mixture as a white wax.

The title compound could not be prepared according to General procedure **B** and only starting material was recovered from the reaction.

HRMS (ESI): Exact mass calculated for $C_{32}H_4NO$ $[M+H]^+$: 545.3104, found: 545.3102.

^{1}H NMR (400 MHz, CDCl3) δ 7.35 – 7.17 (m, 10H, 2 x $C^{15}\mathbf{H}$ + 2 x $C^{16}\mathbf{H}$ + $C^{17}\mathbf{H}$, *A* + *B*), 7.04 – 6.91 (m, 3H, $C^6\mathbf{H}$ + $C^8\mathbf{H}$, *A* + *B*), 6.66 – 6.55 (m, 2H, $C^7\mathbf{H}$, *A* + *B*), 6.46 (dd, J = 8.3, 1.1 Hz, 1H, $C^9\mathbf{H}$, *A*), 6.40 (d, J = 8.1 Hz, 1H, $C^9\mathbf{H}$, *B*), 4.73 (d, J = 16.7 Hz, 1H, $C^{13}\mathbf{H_2}$, *B*), 4.58 – 4.34 (m, 3H, $C^{13}\mathbf{H_2}$, *A* + *B*), 3.27 – 3.20 (m, 1H,

C^{2}**H**, *B*), 3.12 – 2.99 (m, 1H, C^{2}**H$_2$**, *A*), 2.84 (dd, J = 16.4, 3.7 Hz, 2H, C^{4}**H$_2$**, *A* + *B*), 2.80 – 2.53 (m, 4H, C^{19}**H$_2$**, *A* + *B*), 2.51 – 2.37 (m, 2H, C^{4}**H$_2$**, *A* + C^{13}**H$_2$**, *B*), 2.29 – 1.94 (m, 30H, 2 x C^{25}**H$_3$** + 2 x C^{26}**H$_3$** + C^{27}**H$_3$**, *A* + *B*), 1.94 – 1.67 (m, 4H, C^{18}**H$_2$**, *A* + *B*), 1.12 – 1.04 (m, 6H, C^{11}**H$_3$**, *A* + *B*), 1.03 (s, 3H, C^{12}**H$_3$**, *A*), 0.99 (s, 3H, C^{12}**H$_3$**, *B*).

Major diastereoisomer *A*: **^{13}C NMR** (151 MHz, CDCl3) δ 212.0 (C^{20}O), 143.5 (C^{10}), 141.0 (C^{21}), 139.8 (C^{14}), 135.6 (C^{Ar}), 133.3 (2 x C^{Ar}), 129.8 (C^{6}H/C^{8}H), 128.6 (2 x C^{15}H/C^{16}H), 127.3 (2 x C^{Ar} + C^{6}H/C^{8}H), 127.14 (C^{17}H), 126.8 (2 x C^{15}H/C^{16}H), 120.0 (C^{5}), 116.2 (C^{7}H), 111.9 (C^{9}H), 62.0 (C^{2}H), 54.8 (C^{12}H$_2$), 40.0 (C^{19}H$_2$), 36.74 (C^{4}H$_2$), 33.7 (C^{3}), 33.1 (C^{18}H$_2$), 23.83 (C^{12}H$_3$), 17.4 (2 x C^{25}H$_3$/C^{26}H$_3$), 16.81 (C^{27}H$_3$), 16.09 (2 x C^{25}H$_3$/C^{26}H$_3$), 13.9 (C^{11}H$_3$).

Minor diastereoisomer *B*: **^{13}C NMR** (151 MHz, CDCl3) δ 212.1 (C^{20}O), 143.6 (C^{10}), 141.1 (C^{21}), 139.7 (C^{14}), 135.4 (C^{Ar}), 133.1 (2 x C^{Ar}), 129.5 (C^{6}H/C^{8}H), 128.7 (2 x C^{15}H/C^{16}H), 127.4 (2 x C^{Ar} + C^{6}H/C^{8}H), 127.11 (C^{17}H), 126.9 (2 x C^{15}H/C^{16}H), 120.2 (C^{5}), 116.4 (C^{7}H), 112.2 (C^{9}H), 61.6 (C^{2}H), 55.0 (C^{13}H$_2$), 40.3 (C^{19}H$_2$), 36.69 (C^{4}H$_2$), 33.8 (C^{3}), 31.8 (C^{18}H$_2$), 23.77 (C^{12}H$_3$), 17.3 (2 x C^{25}H$_3$/C^{26}H$_3$), 16.78 (C^{27}H$_3$), 16.07 (2 x C^{25}H$_3$/C^{26}H$_3$), 14.3 (C^{11}H$_3$).

IR (neat) (cm^{-1}): 3029, 2923, 1698, 1602, 1497, 1451, 1315, 1116, 1027, 909.

3-(*N*-Benzyl-2-methyl-1,2,3,4-tetrahydroquinolin-3-yl)-1-(2,3,4,5,6-pentamethylphenyl)propan-1-one (2m)

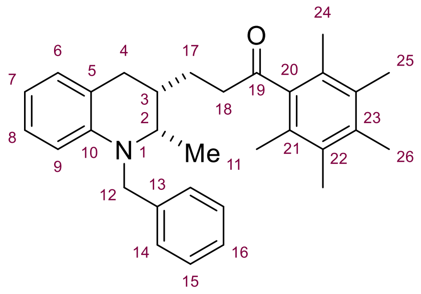

The title compound was prepared according to General Procedure **C** using salt **1e** (45 mg, 0.125 mmol) and 2,3,4,5,6-pentamethylphenyl vinyl ketone (37 mg, 0.19 mmol) as electrophile. Purification by column chromatography (0%-5% EtOAc in pentane) gave *amine* **2m** (18 mg, 33%) as an inseparable 5:1 diastereomeric ratio as a white wax.

The title compound could not be prepared according to General procedure **B** and only starting material was recovered from the reaction.

HRMS (ESI): Exact mass calculated for $C_{31}H_{38}NO$ $[M+H]^+$: 440.2948, found: 440.2946.

^{1}H NMR (400 MHz, $CDCl_3$) δ 7.37 – 7.18 (m, 10H, 5 x C^{Ar}**H**, *A* + *B*), 7.05 – 6.89 (m, 4H, C^{6}**H**, *A* + *B*, C^{8}**H**, *A* + *B*), 6.64 – 6.54 (m, 2H, C^{7}**H** /C^{9}**H**, *A* + *B*), 6.45 – 6.37 (m, 2H, C^{7}**H** /C^{9}**H**, *A* + *B*), 4.58 – 4.39 (m, 4H, C^{12}**H$_2$**, *A* + *B*), 3.51 – 3.41 (m, 1H, C^{2}**H**, *A*), 3.39 – 3.29 (q, 1H, C^{2}**H**, *B*), 2.94 – 2.61 (m, 8H, C^{4}**H$_2$**, *A* + *B*, C^{18}**H$_2$**, *A* + *B*), 2.28 – 2.00 (m, 32H, C^{3}**H**, *A* + *B, 2 x* C^{23}**H$_3$**, *A* + *B*, 2 x C^{24}**H$_3$**, *A* + *BI*, C^{26}**H$_3$**, *A* + *B*), 1.87 – 1.73 (m, 4H, C^{17}**H$_2$**, *A* + *B*), 1.19 (d, *J* = 6.5 Hz, 3H, C^{11}**H$_3$**, *B*) 1.07 (d, *J* = 6.5 Hz, 3H, C^{11}**H$_3$**, *A*).

^{13}C NMR (151 MHz, $CDCl_3$, only major diastereomer *A* reported) δ 211.6 (C^{19}O), 144.1 (C^{10}), 140.8 (C^{20}), 139.4 (C^{13}), 135.6 (C^{23}), 133.3 (2 x C^{21}/C^{22}), 129.2 (C^{6}H/C^{8}H), 128.7 (2 x C^{14}H/C^{15}H), 127.4 (C^{6}H/C^{8}H/2 x

C^{Ar}), 127.3 ($C^6H/C^8H/2$ x C^{Ar}), 126.9 ($C^{16}H$), 126.5 (2 x $C^{14}H/C^{15}H$), 120.9 (C^5), 115.8 (C^7H), 111.7 (C^9H), 57.1 (C^2H), 54.0 ($C^{12}H_2$), 43.5 (C^4H_2), 36.0 (C^3H), 29.9 ($C^{18}H_2$), 26.6 ($C^{17}H_2$), 17.4 (2 x $C^{24}H_3/C^{25}H_3$), 16.8 ($C^{26}H_3$), 16.1 (2 x $C^{24}H_3/C^{25}H_3$), 12.2 ($C^{11}H_3$).

IR (neat) (cm^{-1}): 2924, 1699. 1602, 1499, 1451, 1377, 1353, 1251. 743, 697.

3-(*N*-Benzyl-2-methyl-1,2,3,4-tetrahydroquinolin-3-yl)-1-(2,3,4,5,6-pentamethylphenyl)propan-1-one (2m·HCl)

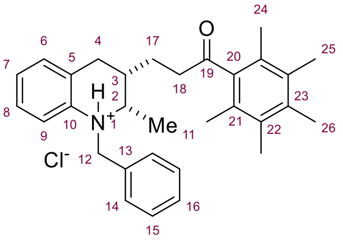

In a 25 mL round bottom flask under argon was charged quinoline product **2m** (35 mg, 0.08 mmol, 1.0 eq.) in dry diethyl ether (1.5 mL). The solution was chilled to 0 °C and 2M HCl in diethyl ether (0.06 mL, 0.12 mmol, 1.5 eq.) was introduced dropwise. After 15 minutes the solids were collected via filtration to give the title compound **2m·HCl** (38 mg, 99%) as a white solid. A single crystal for X-ray diffraction was obtained by slow evaporation from the CH_2Cl_2/heptane solvent system.

^{1}H NMR (400 MHz, $CDCl_3$, major diastereomer) δ 7.92 (dd, *J* = 8.1, 1.4 Hz, 1H), 7.78 – 7.74 (m, 2H), 7.42 – 7.38 (m, 2H), 7.34 (dd, *J* = 7.5, 1.4 Hz, 1H), 7.30 (dd, *J* = 7.8, 1.7 Hz, 1H), 7.26 – 7.21 (m, 2H), 4.80 (d, *J* = 13.0 Hz, 1H), 4.35 (d, *J* = 13.1 Hz, 1H), 3.67 (dd, *J* = 6.8, 3.0 Hz, 1H), 3.02 (dd, *J* = 16.6, 4.9 Hz, 1H), 2.94 (t, *J* = 7.5 Hz, 1H), 2.73 – 2.49 (m, 2H), 2.25 (s, 3H), 2.20 (s, 6H), 2.14 (d, J = 7.1 Hz, 1H), 2.07 (s, 6H), 2.00 (s, 1H), 1.85 (d, *J* = 6.6 Hz, 1H), 1.79 – 1.67 (m, 1H), 1.41 (d, *J* = 6.8 Hz, 3H).

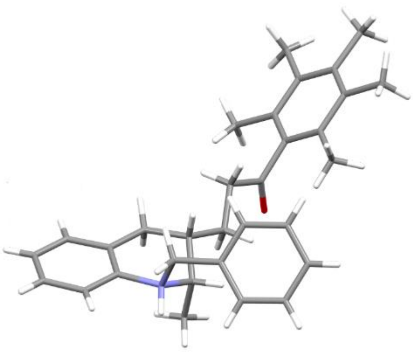

Crystal data $C_{31}H_{38}ClNO$ = 476.10, triclinic, a = 9.2443(4), b = 16.6064(5), c = 18.4379(7) Å, β = 97.470(3)°, Z = 4, T = 300 K, space group *P-1*, 28854 reflections measured, 11191 unique (R_{int} = 0.049), which were used in all calculations.

4-(1,3-Dimethyl-1,2,3,4-tetrahydroquinolin-3-yl)butan-2-one (2n)

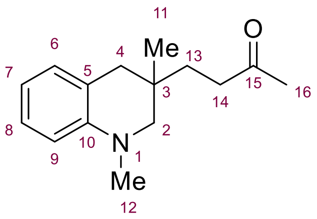

The title compound was prepared according to General Procedure **B** using quinolinium salt **1h** (36 mg, 0.125 mmol) and methyl vinyl ketone (50 µL, 0.63 mmol) as electrophile. Purification by column chromatography (0-10% EtOAc in pentane) furnished *amine* **2n** (23 mg, 78%) as a light-yellow oil.

HRMS (ESI): Exact mass calculated for $C_{15}H_{22}NO$ $[M+H]^+$: 232.1696, found: 232.1698.

^{1}H NMR (400 MHz, $CDCl_3$) δ 7.13 – 7.04 (m, 1H, C^8**H**), 6.93 (d, *J* = 1.1 Hz, 1H, C^6**H**), 6.66 – 6.56 (m, 2H, C^7**H** + C^9**H**), 2.95 – 2.83 (m, 5H, C^2**H₂** + C^{12}**H₃**), 2.60 – 2.51 (m, 2H, C^4**H₂**), 2.51 – 2.43 (m, 2H, C^{14}**H₂**), 2.14 (s, 3H, C^{16}**H₃**), 1.69 – 1.52 (m, 2H, C^{13}**H₂**), 0.95 (s, 3H, C^{11}**H₃**).

^{13}C NMR (101 MHz, $CDCl_3$) δ 209.1 (C^{15}), 145.6 (C^{10}), 129.6 (C^{6}H), 127.2 (C^{8}H), 121.1 (C^{5}), 116.5 (C^{7}H), 110.8 (C^{9}H), 61.4 (C^{2}H$_2$), 40.3 (C^{4}H$_2$), 39.3 (C^{12}H$_3$), 38.6 (C^{14}H$_2$), 32.7 (C^{13}H$_2$), 30.9 (C^{3}), 30.0 (C^{16}H$_3$), 23.6 (C^{11}H$_3$).

IR (neat) (cm^{-1}): 3657, 2980, 1714, 1603, 1503, 1461, 1375, 1278, 1160, 747.

4-(*N*-(4-Methoxybenzyl)-3-methyl-1,2,3,4-tetrahydroquinolin-3-yl)butan-2-one (2o)

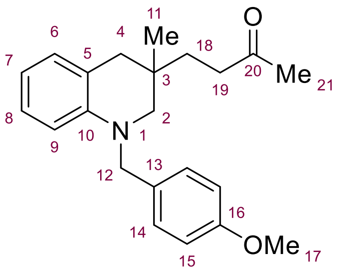

The title compound was prepared according to General Procedure **B** using quinolinium salt **1i** (49 mg, 0.125 mmol) and methyl vinyl ketone (50 µL, 0.63 mmol) as electrophile. Purification by column chromatography (0-10% EtOAc in pentane) furnished *amine* **2o** (33 mg, 77%) as a pale yellow oil.

HRMS (ESI): Exact mass calculated for $C_{22}H_{28}NO_2$ $[M+H]^+$: 338.2115, found: 338.2112.

^{1}H NMR (400 MHz, $CDCl_3$) δ 7.19 (d, *J* = 8.9 Hz, 2H, 2 x C^{14}**H**), 7.01 (ddd, *J* = 8.2, 7.3, 1.7 Hz, 1H, C^{8}**H**), 7.00 – 6.92 (m, 1H, C^{6}**H**), 6.86 (d, *J* = 8.7 Hz, 2H, 2 x C^{15}**H**), 6.64 – 6.55 (m, 2H, C^{7}**H** + C^{9}**H**), 4.48 – 4.33 (m, 2H, C^{12}**H$_2$**), 3.79 (s, 3H, C^{17}**H$_3$**), 3.05 – 2.93 (m, 2H, C^{4}**H$_2$**), 2.58 (s, 2H, C^{2}**H$_2$**), 2.50 – 2.30 (m, 2H, C^{19}**H$_2$**), 2.11 (s, 3H, C^{21}**H$_3$**), 1.70 – 1.52 (m, 2H, C^{18}**H$_2$**), 0.96 (s, 3H, C^{8}**H$_3$**).

^{13}C NMR (101 MHz, $CDCl_3$) δ 209.0 (C^{20}O), 158.7 (C^{16}), 144.8 (C^{10}), 131.1 (C^{Ar}), 129.9 (C^{6}H), 128.0 (2 x C^{14}H), 127.3 (C^{8}H), 120.6 (C^{Ar}), 116.4 (C^{7}H/C^{9}H), 114.1 (2 x C^{15}H), 110.9 (C^{7}H/C^{9}H), 59.5 (C^{4}H$_2$), 55.4 (C^{17}H$_3$), 54.8 (C^{12}H$_2$), 40.7 (C^{2}H$_2$), 38.4 (C^{19}H$_2$), 32.5 (C^{18}H$_2$), 30.8 (C^{3}), 30.0 (C^{21}H$_2$), 23.5 (C^{11}H$_3$).

IR (neat) (cm^{-1}): 2980, 1714, 1603, 1509, 1457, 1352, 1282, 1244, 1170, 1034.

3,3'-(*N*-Benzyl-6-chloro-1,2,3,4-tetrahydroquinoline-3,3-diyl)bis(1-phenylpropan-1-one) (2p)

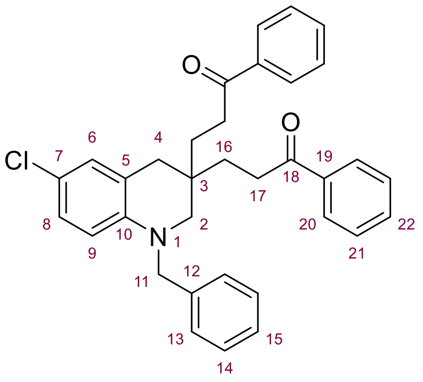

The title compound was prepared according to General Procedure **B** using quinolinium salt **1k** (48 mg, 0.125 mmol) and phenyl vinyl ketone (66 mg, 0.50 mmol) as electrophile. Purification by column chromatography (5-20% EtOAc in pentane) gave *amine* **2p** (38 mg, 58%) as a pale yellow solid.

m.p. (EtOAc): 148 – 150 °C.

HRMS (ESI): Exact mass calculated for $C_{34}H_{32}NO_2Cl$ $[M+H]^+$: 522.2205, found: 522.2196.

^{1}H NMR (400 MHz, $CDCl_3$) δ 7.93 – 7.80 (m, 4H, 4 x C^{20}**H**), 7.60 – 7.51 (m, 2H, 2 x C^{22}**H**), 7.46 – 7.37 (m, 4H, 4 x C^{21}**H**), 7.31 – 7.12 (m, 5H, 5 x C^{Ar}H), 7.02 – 6.90 (m, 2H, C^{7}**H** + C^{8}**H**), 6.51 (d, *J* = 8.7 Hz, 1H, C^{9}**H**), 4.45 (s, 2H, C^{11}**H$_2$**), 3.13 (s, 2H, C^{4}**H$_2$**), 3.06 – 2.78 (m, 4H, 2 x C^{17}**H$_2$**), 2.71 (s, 2H, C^{2}**H$_2$**), 1.92 – 1.74 (m, 4H, 2 x C^{16}**H$_2$**).

^{13}C NMR (101 MHz, $CDCl_3$) δ 199.6 (2 x $C^{18}O$), 151.6 (C^7Cl), 146.0 (C^{Ar}), 137.6 (2 x C^{Ar}), 136.8 (C^{Ar}), 133.3 (2 x $C^{22}H$), 129.0 (2 x $C^{13}H/C^{14}H$), 128.8 (4 x $C^{21}H$), 128.1 (4 x $C^{20}H$), 127.5 (C^6H + C^8H + $C^{15}H$), 126.7 (2 x $C^{14}H/C^{13}H$), 114.8 (C^9H), 113.8 (C^{Ar}), 112.2 (C^6H), 57.5 (C^4H_2), 56.1 ($C^{11}H_2$), 35.3 (C^2H_2), 32.6 (C^3), 32.4 (2 x $C^{17}H_2$), 29.0 (2 x $C^{16}H_2$).

IR (neat) (cm^{-1}): 3059, 2159, 2028, 1686, 1596, 1503, 1447, 1298, 1278, 1222.

3,3'-(*N*-Benzyl-7-methyl-1,2,3,4-tetrahydroquinoline-3,3-diyl)bis(1-phenylpropan-1-one) (2q)

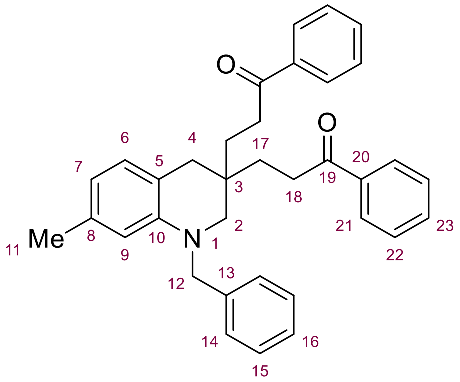

The title compound was prepared according to General Procedure **C** using quinolinium salt **1l** (45 mg, 0.125 mmol) and phenyl vinyl ketone (66 mg, 0.50 mmol) as electrophile. Purification by column chromatography (5-20% EtOAc in pentane) gave *amine* **2q** (27 mg, 44%) as a pale brown solid. General procedure **B** afforded the title compound in a significantly reduced yield of 29%.

m.p. (EtOAc): 123 – 125 °C

HRMS (ESI): Exact mass calculated for $C_{35}H_{35}NO_2$ $[M+H]^+$: 502.2741, found: 502.2741.

^{1}H NMR (400 MHz, $CDCl_3$) δ 7.87 – 7.78 (m, 4H, 4 x $C^{21}\mathbf{H}$), 7.55 – 7.45 (m, 2H, 2 x $C^{23}\mathbf{H}$), 7.43 – 7.34 (m, 4H, 4 x $C^{22}\mathbf{H}$), 7.29 – 7.08 (m, 5H, 5 x $C^{Ar}H$), 6.88 (d, *J* = 7.8 Hz, 1H, $C^6\mathbf{H}$), 6.48 – 6.41 (m, 2H, $C^7\mathbf{H}$ + $C^9\mathbf{H}$), 4.44 (s, 2H, $C^{12}\mathbf{H_2}$), 3.07 (s, 2H, $C^4\mathbf{H_2}$), 3.00 – 2.78 (m, 4H, 2 x $C^{18}\mathbf{H_2}$), 2.69 (s, 2H, $C^2\mathbf{H_2}$), 2.19 (s, 3H, $C^{11}\mathbf{H_3}$), 1.90 – 1.71 (m, 4H, 2 x $C^{17}\mathbf{H_2}$).

^{13}C NMR (101 MHz, $CDCl_3$) δ 200.1 (2 x $C^{19}O$), 144.9 (C^{10}), 139.1 (C^{13}), 137.1 (C^8), 137.0 (2 x C^{20}), 133.1 (2 x $C^{23}H$), 129.9 (C^6H), 128.73 (2 x $C^{14}H$), 128.67 (4 x $C^{22}H$), 128.2 (4 x $C^{21}H$), 127.2 (2 x $C^{15}H$), 127.1 ($C^{16}H$), 117.8 (C^7H), 117.3 (C^5), 111.7 (C^9H), 57.7 (C^4H_2), 55.3 ($C^{12}H_2$), 39.1 (C^2H_2), 33.3 (C^3), 32.7 (2 x $C^{18}H_2$), 29.2 (2 x $C^{17}H_2$), 21.7 ($C^{11}H_3$).

IR (neat) (cm^{-1}): 3657, 2980, 2921, 1682, 1512, 1449, 1379, 1251, 1158, 741.

3,3'-(*N*-Benzyl-5-nitro-1,2,3,4-tetrahydroquinoline-3,3-diyl)bis(1-phenylpropan-1-one) (2r)

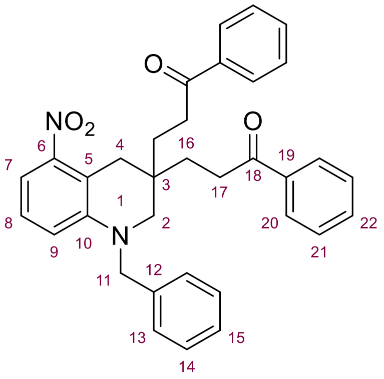

The title compound was prepared according to General Procedure **B** using quinolinium salt **1m** (49 mg, 0.125 mmol) and phenyl vinyl ketone (66 mg, 0.50 mmol) as electrophile. Purification by column chromatography (5-20% EtOAc in pentane) gave *amine* **2r** (22 mg, 33%) as an orange solid.

m.p. (EtOAc): 168 – 170 °C

HRMS (ESI): Exact mass calculated for $C_{34}H_{32}N_2O_4$ $[M+H]^+$: 533.446, found: 533.2434.

^{1}H NMR (400 MHz, $CDCl_3$) δ 7.84 – 7.77 (m, 4H, 4 x C^{20}**H**), 7.51 – 7.42 (m, 2H, 2 x C^{22}**H**), 7.35 (dd, *J* = 8.4, 7.0 Hz, 4H, 4 x C^{21}**H**), 7.22 – 7.05 (m, 5H, 5 x C^{Ar}H), 7.04 – 6.95 (m, 2H, C^7**H** + C^8**H**), 6.69 (dd, *J* = 6.4, 3.2 Hz, 1H, C^9**H**), 4.47 (s, 2H, C^{11}**H₂**), 3.15 (s, 2H, C^4**H₂**), 3.01 – 2.88 (m, 2H, 2 x C^{17}**H₂**), 2.84 – 2.72 (m, 4H, C^2**H₂** + 2 x C^{17}**H₂**), 1.86 – 1.65 (m, 4H, 2 x C^{16}**H₂**).

^{13}C NMR (101 MHz, $CDCl_3$) δ 199.6 (2 x C^{18}O), 151.6 (C^6), 146.0 (C^{10}), 137.6 (2 x C^{Ar}), 136.8 (C^{Ar}), 133.3 (2 x C^{22}H), 129.0 (2 x C^{13}H/C^{14}H), 128.8 (4 x C^{20}H/C^{21}H), 128.1 (4 x C^{20}H/C^{21}H), 127.5 (C^8H + C^{15}), 126.7 (2 x C^{13}H/C^{14}H), 114.8 (C^9H), 113.8 (C^{Ar}), 112.2 (C^7H), 57.5 (C^4H_2), 56.1 ($C^{11}H_2$), 35.3 (C^2H_2), 32.6 (C^3), 32.4 (2 x $C^{17}H_2$), 29.0 (2 x $C^{16}H_2$).

IR (neat) (cm^{-1}): 3061, 2922, 1680, 1600, 1521, 1496, 1449, 1350, 1288, 1213.

Methyl 1-benzyl-3,3-bis(3-oxobutyl)-1,2,3,4-tetrahydroquinoline-4-carboxylate (2s)

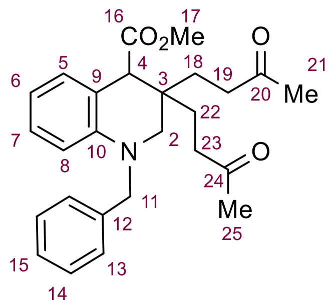

The title compound was prepared according to General Procedure **B** using quinolinium salt **1g** (50 mg, 0.12 mmol) and methyl vinyl ketone (33 mg, 0.43 mmol) as electrophile. Purification by column chromatography (3-25% EtOAc in pentane) furnished *amine* **2s** (36 mg, 69%) as a light-yellow oil.

HRMS (ESI): Exact mass calculated for $C_{26}H_{32}NO_4$ $[M+H]^+$: 422.2326 found: 422.2325;

^{1}H NMR (400 MHz, $CDCl_3$) δ 7.36 – 7.28 (m, 2H, C^{13}**H**), 7.27 – 7.20 (m, 3H, C^{14}**H** + C^{15}**H**), 7.11 – 6.98 (m, 2H, C^5**H** + C^6**H**), 6.65 (dd, *J* = 8.4, 1.1 Hz, 1H, C^8**H**), 6.60 (td, *J* = 7.3, 1.1 Hz, 1H, C^7**H**), 4.60 (d, J = 16.9 Hz, 1H, C^{11}**H$_a$**), 4.47 (d, *J* = 16.9 Hz, 1H, C^{11}**H$_b$**), 3.66 (s, 3H, C^{17}**H₃**), 3.63 – 3.70 (d, *J* = 12.0, 1H, C^2**H$_a$**) 3.50 (d, *J* = 2.1 Hz, 1H, C^4**H**), 2.78 (dd, *J* = 12.0, 2.1 Hz, 1H, C^2**H$_b$**), 2.62 (ddd, *J* = 17.4, 10.9, 5.0 Hz, 1H, C^{19}**H$_a$**), 2.41 (ddd, *J* = 17.1, 10.9, 5.5 Hz, 1H, C^{19}**H$_b$**), 2.27 – 2.10 (m, 5H, C^{23}**H₂** + C^{21}**H₃**), 2.00 (s, 3H, C^{25}**H₃**), 1.69 – 1.37 (m, 4H, C^{18}**H₂** + C^{22}**H₂**).

^{13}C NMR (151 MHz, $CDCl_3$) δ 208.0 (C^{20}O), 207.8 (C^{24}O), 173.6 (C^{16}O), 144.1 (C^{10}), 138.6 (C^{12}), 130.3 (C^5H), 129.0 (C^7H), 128.8 (2 x C^{14}H), 127.1 (C^{15}H), 126.9 (2 x C^{13}H), 116.5 (C^9), 116.4 (C^6H), 111.4 (C^8H), 54.8 ($C^{11}H_2$), 52.8 (C^2H_2), 52.3 (C^4H), 52.1 ($C^{17}H_3$), 37.2 ($C^{19}H_2$), 36.9 ($C^{23}H_2$), 34.6 (C^3), 30.22 ($C^{21}H_3$), 30.17 ($C^{25}H_3$), 26.6 ($C^{18}H_2$), 26.4 ($C^{22}H_2$).

IR (neat) (cm^{-1}): 2947, 2925, 1713, 1602, 1507, 1355, 1244, 1159, 746.

Synthesis of isoquinolinium salts

General Procedure D: Preparation of isoquinolinium salts

A mixture of the corresponding isoquinoline (1.00 equiv.) and benzyl iodide (2.00 equiv.) in acetone (0.5 M) was stirred in the dark at room temperature for 20 hours. Addition of diethyl ether (10 mL) resulted in precipitation and the resulting suspension was sonicated (15 min). The solids were collected by filtration, washed with diethyl ether, and dried under vacuum to give the benzyl quinolinium iodide salts as crystalline solids. Where necessary additional purification by column chromatography was performed.

_N_-Benzyl-4-methylisoquinolinium iodide (**4a**)

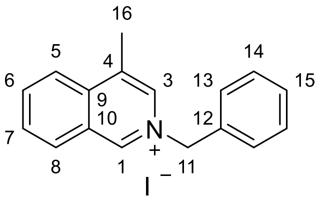

The title compound was prepared according to the General Procedure **D** using 4-methylisoquinoline (858 mg, 6.00 mmol, 1.0 equiv.) and benzyl iodide (1.13 mL, 9.00 mmol) in acetone (0.5 M). The resultant solid was washed with diethyl ether and dried under vacuum. Further drying under high vacuum at 70 °C for 10 hours gave the salt **4a** (2.09 g, 97%) as a yellow solid.

m.p. (acetone): 126 – 128 °C

HRMS (ESI): Exact mass calculated for $C_{17}H_{16}N$ $[M]^+$: 234.1277, found 234.1278

^{1}H NMR (400 MHz, DMSO-d_6) δ 10.20 (s, 1H, C^{1}**H**), 8.80 (s, 1H, C^{3}**H**), 8.54 (dd, *J* = 8.3, 1.1 Hz, 1H, C$^{5/8}$**H**), 8.36 (dd, *J* = 8.6, 1.1 Hz, 1H, C$^{5/8}$**H**), 8.30 (dd, *J* = 6.9, 1.4 Hz, 1H, C$^{6/7}$**H**), 8.09 (ddd, *J* = 8.1, 6.8, 1.2 Hz, 1H, C$^{6/7}$**H**), 7.66 – 7.57 (m, 2H, 2 x C^{14}**H**), 7.50 – 7.37 (m, 3H, 2 x C^{13}**H** + C^{15}**H**), 5.94 (s, 2H, C^{11}**H$_2$**), 2.76 (s, 3H, C^{16}**H$_3$**)

^{13}C NMR (101 MHz, DMSO-d_6) δ 148.3 (C^{1}H), 137.0 (C^{Ar}H), 136.6 (C^{Q}), 135.2 (C^{Q}), 134.2 (C^{Q}), 133.3 (C^{3}H), 131.1 (2 x C^{Ar}H), 129.3 (C^{15}H), 129.2 (2 x C^{13}H), 128.8 (2 x C^{14}H), 126.9 (C^{Q}), 124.4 (C^{Ar}), 63.2 (C^{11}H$_2$), 15.8 (C^{16}H$_3$)

IR (neat) (cm^{-1}): 3672, 2999, 2886, 1474, 1460, 1380, 1252, 1152, 1072, 954.

_N_-Benzyl-4-methylisoquinolinium bromide (4a')

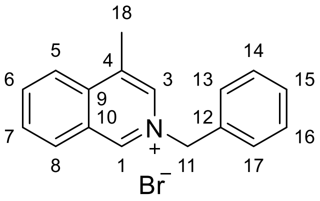

The title compound was prepared according to a modified General Procedure **D**. A mixture of 4-methylisoquinoline (500 mg, 3.49 mmol, 1.0 eq.) and benzyl bromide (0.50 mL, 4.19 mmol, 1.2 eq.) in dioxane (10 mL) was stirred at 90 °C for 24 h. The reaction mixture was filtered. The resultant solid was sonicated in Et_2O (10 mL) for 5 min and washed with ether (50 mL) then dried under vacuum to give isoquinolinium bromide **4a'** (1.01 g, 92%) as an off-white solid.

m.p. (acetone): 197 °C

HRMS (ESI): Exact mass calculated for $C_{17}H_{16}N$ $[M]^+$: 234.1277, found: 234.1279;

^{1}H NMR (400 MHz, DMSO-d_6) δ 10.24 (s, 1H, C^{1}**H**), 8.82 (t, *J* = 1.3 Hz, 1H, C^{3}**H**), 8.54 (dt, *J* = 8.2, 1.1 Hz, 1H, $C^{5/8}$**H**), 8.37 (dd, *J* = 8.6, 1.1 Hz, 1H, $C^{5/8}$**H**), 8.30 (ddd, *J* = 8.5, 6.9, 1.3 Hz, 1H, $C^{6/7}$**H**), 8.09 (ddd, *J* = 8.2, 6.9, 1.2 Hz, 1H, $C^{6/7}$**H**), 7.64 – 7.59 (m, 2H, 2 x C^{Ar}**H**), 7.48 – 7.41 (m, 3H, 3 x C^{Ar}**H**), 5.94 (s, 2H, C^{11}**H_2**), 2 2.76 (s, 1H, C^{18}**H_3**);

^{13}C NMR (101 MHz, DMSO-d_6) δ 148.4 (C^{1}H), 137.0 ($C^{5/8}$H), 136.6 (C^{Ar}), 135.2 (C^{Ar}), 134.3 (C^{Ar}), 133.3 (C^{3}H), 131.2 (C^{Ar}H), 131.1 C^{Ar}H), 129.3 (C^{Ar}H), 129.2 (2 x C^{Ar}H), 128.8 (2 x C^{Ar}H), 127.0 (C^{Ar}), 124.4 ($C^{5/8}$H), 63.2 ($C^{11}H_2$), 15.7 ($C^{18}H_3$);

IR (neat) (cm^{-1}): 3418, 3006, 2164, 2032, 1978, 1642, 1399, 1170, 1116, 871,744, 699.

***N*-Benzyl-4-butylisoquinolinium iodide** (**4b**)

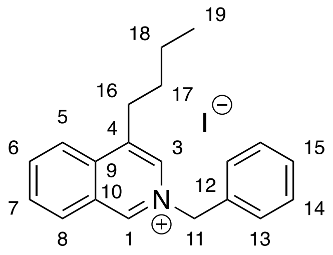

The title compound was prepared according to the General Procedure **D** using 4-butylisoquinoline (930 mg, 5.00 mmol) and benzyl iodide (1.20 mL, 10.0 mmol) in acetone (0.5 M) The resultant solid was washed with diethyl ether and dried under vacuum to give salt **4b** (1.95 g, 97%) as a yellow solid. Spectroscopic data was consistent with that reported in the literature.[8]

^{1}H NMR (400 MHz, DMSO-d_6) δ 10.16 (s, 1H, C^{1}**H**), 8.78 (s, 1H, C^{3}**H**), 8.53 (d, *J* = 7.8 Hz, 1H, $C^{5/8}$**H**), 8.45 (dd, *J* = 8.6, 1.0 Hz, 1H, $C^{5/8}$**H**), 8.29 (ddd, *J* = 8.4, 7.0, 1.3 Hz, 1H, $C^{6/7}$**H**), 8.08 (ddd, *J* = 8.1, 7.0, 1.0 Hz, 1H, $C^{6/7}$**H**), 7.63 – 7.55 (m, 2H, 2 x C^{14}**H**), 7.50 – 7.37 (m, 3H, 2 x C^{13}**H** + C^{15}**H**), 5.94 (s, 2H, C^{11}**H_2**), 3.22 – 3.14 (m, 2H, C^{16}**H_2**), 1.73 – 1.61 (m, 2H, C^{17}**H_2**), 1.38 (m, 2H, C^{18}**H_2**), 0.91 (t, *J* = 7.3 Hz, 3H, C^{19}**H_3**)

^{13}C NMR (101 MHz, DMSO-d_6) δ 148.4 (C^{1}H), 139.2 (C^{Q}), 137.1 ($C^{6/7}$H), 136.1 (C^{Q}), 134.4 (C^{Q}), 133.3 (C^{3}H), 131.4 ($C^{5/8}$H), 131.1 ($C^{6/7}$H), 129.3 (C^{15}H), 129.2 (2 x C^{13}H), 128.7 (2 x C^{14}H), 127.4 (C^{Q}), 124.2 ($C^{5/8}$H), 63.3 ($C^{11}H_2$), 31.7 ($C^{17}H_2$), 28.8 ($C^{16}H_2$), 21.9 ($C^{18}H_2$), 13.7 ($C^{19}H_3$).

[8] Reeves, B. M.; Hepburn, H. B.; Grozavu, A.; Lindsay-Scott, P. J.; Donohoe, T. J.; *Angew. Chem*. **2019**, *58*, 15697–15701.

N-Benzyl-4-isobutylisoquinolinium iodide (4c)

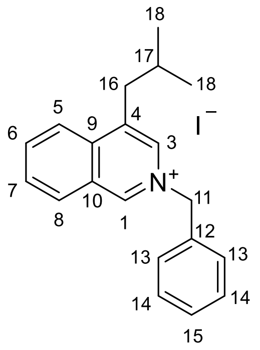

The title compound was prepared according to a modified General Procedure **D**. A mixture of 4-isobutylisoquinoline (758 mg, 4.09 mmol, 1.0 eq.) and benzyl iodide (0.50 mL, 4.91 mmol, 1.2 eq.) in acetone (5 mL) was stirred for 24 hours. The resultant precipitate was dried under vacuum to give isoquinolinium iodide **4c** (1.21 g, 99%) as a yellow solid. Spectroscopic data was consistent with that reported in the literature.[4]

^{1}H NMR (400 MHz, $CDCl_3$) δ 10.93 (d, *J* = 1.4 Hz, 1H, C^{1}**H**), 8.67 (d, *J* = 8.3 Hz, 1H, C^{5}**H**), 8.60 (d, *J* = 1.5 Hz, 1H, C^{3}**H**), 8.15 – 8.05 (m, 2H, C^{8}**H** + C^{7}**H**), 7.88 (ddd, *J* = 8.1, 6.4, 1.6 Hz, 1H, C^{6}**H**), 7.75 – 7.67 (m, 2H C^{13}**H**), 7.31 (dd, *J* = 5.0, 1.9 Hz, 3H, C^{14}**H** + C^{15}**H**), 6.28 (s, 2H, C^{11}**H$_2$**), 2.98 (d, *J* = 7.2 Hz, 2H, C^{16}**H$_2$**), 2.01 (hept, *J* = 6.7 Hz, 1H, C^{17}**H**), 0.90 (d, *J* = 6.6 Hz, 6H, C^{18}**H$_3$**).

^{13}C NMR (101 MHz, $CDCl_3$) δ 147.8 (C^{1}H), 138.7 (C^{12}), 137.3 (C^{9}), 136.9 (C^{7}H), 133.3 (C^{Ar}), 133.2 (C^{3}H), 132.0 (C^{5}H), 130.9 (C^{6}H), 129.9 (C^{15}H), 129.6 (2 x C^{13}H), 129.5 (2 x C^{14}H), 127.9 (C^{10}), 123.8 (C^{8}H), 63.4 (C^{11}H$_2$), 39.0 (C^{16}H$_2$), 29.5 (C^{17}H), 22.6 (2 x C^{18}H$_3$).

N-Benzyl-4-toluylisoquinolinium iodide (4d)

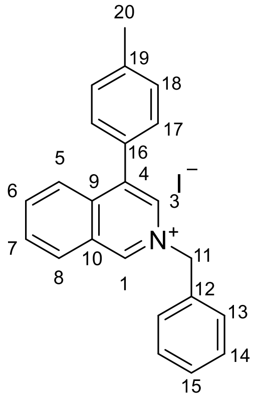

The title compound was prepared according to General Procedure **D** using 4-(*p*-tolyl)isoquinoline (1.05 g, 4.79 mmol) and benzyl iodide (0.72 mL, 5.75 mmol) to give salt **4d** (1.60 g, 77 %) as a brown solid. Spectroscopic data was consistent with that reported in the literature.[4]

^{1}H NMR (400 MHz, $CDCl_3$) δ 11.15 (s, 1H, C^{1}**H**), 8.79 (d, *J* = 8.3 Hz, 1H, C^{5}**H**), 8.33 (d, *J* = 1.5 Hz, 1H, C^{3}**H**), 8.11 – 7.99 (m, 2H, C^{8}**H** + C^{7}**H**), 7.94 (ddd, *J* = 8.2, 6.5, 1.6 Hz, 1H, C^{6}**H**), 7.71 – 7.65 (m, 2H, C^{13}**H**), 7.47 – 7.40 (m, 2H, C^{17}**H**), 7.39 – 7.32 (m, 5H, C^{14}**H** + C^{15}**H** + C^{18}**H**), 6.35 (s, 2H, C^{11}**H$_2$**), 2.45 (s, 3H, C^{20}**H$_3$**).

^{13}C NMR (101 MHz, $CDCl_3$) δ 148.6 (C^{1}H), 140.4 (C^{9}), 140.0 (C^{16}), 137.3 (C^{7}H), 136.7 (C^{12}), 132.8 (C^{4}), 132.4 (C^{3}H), 131.9 (C^{5}H), 131.3 (C^{6}H), 130.16 (C^{Ar}), 130.06 (C^{Ar}), 129.9 (C^{Ar}), 129.8 (C^{Ar}), 129.7 (4x C^{Ar}), 128.2 (C^{Ar}), 125.7 (C^{8}H), 64.2 (C^{11}H$_2$), 21.5 (C^{20}H$_3$).

N-Benzyl-1-butyl-4-methylisoquinolinium iodide (4e)

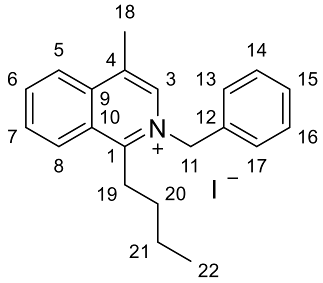

The title compound was prepared according to General Procedure **D** using 1-butyl-4-methylisoquinoline (199 mg, 1.0 mmol, 1.0 eq.) and benzyl iodide (0.19 mL, 1.5 mmol 1.5 eq.) in acetone (2.5 mL) to give salt **4e** (402 mg, 96%) as a cream-colored solid.

m.p. (acetone): 234 °C

HRMS (ESI): Exact mass calculated for $C_{21}H_{24}N$ $[M]^+$: 290.1903, found: 290.1905;

^{1}H NMR (400 MHz, DMSO-d_6) δ 8.81 (d, *J* = 1.1 Hz, 1H, C^{3}**H**), 8.72 (dt, *J* = 8.7, 0.9 Hz, 1H, C5$^{/8}$**H**), 8.40 (dd, *J* = 8.4, 1.2 Hz, 1H, C$^{5/8}$**H**), 8.30 (ddd, *J* = 8.3, 7.0, 1.1 Hz, 1H, C$^{6/7}$**H**), 8.09 (ddd, *J* = 8.3, 6.9, 1.2 Hz, 1H, C$^{6/7}$**H**), 7.48 – 7.38 (m, 3H, 3 x C^{Ar}**H**), 7.33 (dd, *J* = 7.9, 1.8 Hz, 2H, 2 x C^{Ar}**H**), 6.07 (s, 2H, C^{11}**H$_2$**), 3.66 – 3.53 (m, 2H, C^{19}**H**), 2.77 (s, 3H, C^{18}**H$_3$**), 1.56 – 1.32 (m, 4H, C^{20}**H$_2$** + C^{21}**H$_2$**), 0.83 (t, *J* = 7.1 Hz, 3H, C^{22}**H$_3$**);

^{13}C NMR (101 MHz, DMSO-d_6) δ 161.1 (C^{1}), 136.8 (C^{Ar}), 136.2 (C$^{6/7}$H), 135.4 (C^{9}H), 134.5 (C^{Ar}), 132.7 (C^{Ar}), 131.1 (C$^{6/7}$H), 129.1 (2 x C^{Ar}H), 129.1 (C$^{5/8}$H), 128.6 (C^{Ar}), 127.0 (2 x C^{Ar}H), 126.7 (C^{A}), 125.0 (C$^{5/8}$H), 60.8 (C^{11}H$_2$), 31.6 (C^{21}H$_2$), 29.1 (C^{19}H$_2$), 22.2 (C^{20}H$_2$), 15.7 (C^{18}H$_3$), 13.5 (C^{22}H$_3$);

IR (neat) (cm^{-1}): 2961, 2938, 2161, 2033, 2034, 1978, 1493, 1413, 782, 741, 725, 699, 686.

2-Benzyl-3-methylisoquinolin-2-ium iodide (4f)

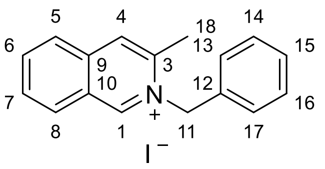

The title compound was prepared according to General Procedure **D** using 3-methylisoquinoline (430 mg, 3.00 mmol, 1.0 eq.) and benzyl iodide (0.56 mL, 4.5 mmol 1.5 eq.) in acetone (6 mL) to give salt **4f** (907 mg, 84%) as a yellow solid.

m.p. (acetone): 201 °C

HRMS (ESI): Exact mass calculated for $C_{17}H_{16}N$ $[M]^+$: 234.1277, found: 234.1277;

^{1}H NMR (400 MHz, DMSO-d_6) δ 10.29 (s, 1H, C^{1}**H**), 8.54 (s, 1H, C^{4}**H**), 8.50 (d, *J* = 8.4 Hz, 1H, C$^{5/8}$**H**), 8.28 – 8.22 (m, 2H, C$^{6/7}$**H** + C$^{5/8}$**H**), 8.03 (dt, *J* = 8.2, 4.0 Hz, 1H, C$^{6/7}$**H**), 7.48 – 7.38 (m, 3H, 3 x C^{Ar}**H**), 7.36 – 7.30 (m, 2H, 2 x C^{Ar}**H**), 6.09 (s, 2H, C^{11}**H$_2$**), 2.76 (s, 3H, C^{18}**H$_3$**);

^{13}C NMR (101 MHz, DMSO-d_6) δ 151.5 (C^{1}H), 144.3 (C^{Ar}), 138.0 (C^{Ar}), 137.2 (C^{8}), 133.3 (C^{Ar}), 130.5 (C$^{6/7}$), 130.2 (C$^{5/8}$H), 129.2 (2 x C^{Ar}H), 128.7 (C^{Ar}H), 127.5 (2 x C^{Ar}H), 126.6 (C^{4}H), 126.3 (C$^{6/7}$H), 126.1 (C^{Ar}), 60.6 (C^{11}H$_2$), 19.2 (C^{18}H$_3$);

IR (neat) (cm^{-1}): 2982, 2162, 1645, 1439, 1400, 1344, 913, 747, 740, 706.

***N*-Benzyl-4-methyl-3-phenylisoquinolinium iodide (4g)**

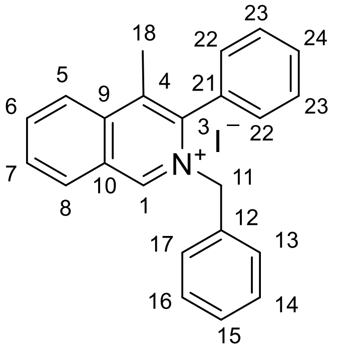

The title compound was prepared according to General Procedure **D** using 3-phenyl-4-butylisoquinoline (318 mg, 1.45 mmol) and benzyl iodide (0.26 mL, 2.00 mmol). Purification by flash column chromatography (0-6% MeOH in CH_2Cl_2) gave salt **4g** (511 mg, 81%) as a yellow solid. Spectroscopic data was consistent with that reported in the literature.[4]

^{1}H NMR (400 MHz, $CDCl_3$) δ 10.99 (s, 1H, C^{1}**H**), 8.83 – 8.75 (d, *J* = 8.2, 1H, C^{5}**H**), 8.27 – 8.10 (m, 2H, C^{8}**H** + C^{7}**H**), 7.95 (ddd, *J* = 8.2, 6.5, 1.6 Hz, 1H, C^{6}**H**), 7.63 – 7.54 (m, 1H,

C^{15}**H**), 7.54 – 7.45 (m, 2H, C^{23}**H**), 7.32 – 7.15 (m, 5H, C^{24}**H** + C^{22}**H** + C^{14}**H** + C^{16}**H**), 7.00 – 6.88 (m, 2H, C^{13}**H** + C^{17}**H**), 5.93 (s, 2H, C^{11}**H$_2$**), 2.44 (s, 3H, C^{18}**H$_3$**).

^{13}C NMR (101 MHz, $CDCl_3$) δ 150.1 (C^{1}H), 143.8 (C^{9}), 138.0 (C^{4}), 137.6 (C^{7}H), 134.3 (C^{12}), 133.2 (C^{21}), 132.4 (C^{5}H+ C^{Ar}), 131.0 (C^{Ar}H), 130.9 (C^{7}H), 130.2 (2 x C^{Ar}H), 129.6 (2 x C^{23}H), 129.3 (2 x C^{Ar}H), 129.2 (C^{Ar}), 128.3 (C^{13} + C^{17}), 126.7 (C^{10}), 124.3 (C^{8}H), 62.9 (C^{11}H$_2$), 16.5 (C^{18}H$_3$).

N-Benzyl-1,2,3,4-tetrahydrophenanthridinium iodide (4h)

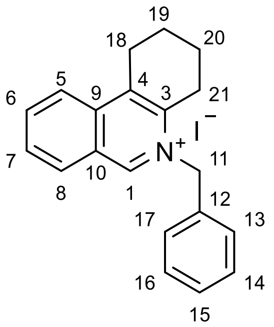

The title compound was prepared according to General Procedure **D** using 1,2,3,4-tetrahydrophenanthridine[9] (110 mg, 0.6 mmol, 1.0 eq.), benzyl iodide (0.13 mL, 1.0 mmol, 1.7 eq.) and acetone (2 mL). After precipitation with diethyl ether the product **4h** was obtained as light-yellow powder (179 mg, 74% yield).

m.p. (acetone): 231 °C

HRMS (ESI): Exact mass calculated for $C_{20}H_{20}N$ $[M]^+$: 274.1590, found: 274.1591.

^{1}H NMR (400 MHz, DMSO-*d$_6$*) δ 10.13 (s, 1H, C^{1}**H**), 8.48 (d, *J* = 8.2 Hz, 1H, C^{5}**H**), 8.36 (d, *J* = 8.6 Hz, 1H, C^{8}**H**), 8.28 (ddd, *J* = 8.4, 6.8, 1.3 Hz, 1H, C^{7}**H**), 8.04 (t, *J* = 7.3 Hz, 1H, C^{6}**H**), 7.47 – 7.37 (m, 3H, C^{13}**H** + C^{15}**H** + C^{17}**H**), 7.34 – 7.21 (m, 2H, C^{14}**H** + C^{16}**H**), 6.05 (s, 2H, C^{11}**H$_2$**), 3.30 – 3.23 (m, 2H, C^{21}**H$_2$**), 3.09 – 2.93 (m, 2H, C^{18}**H$_2$**), 1.99 – 1.78 (m, 4H, C^{19}**H$_2$** + C^{20}**H$_2$**).

^{13}C NMR (101 MHz, DMSO-*d$_6$*) δ 150.1 (C^{3}), 142.6 (C^{1}H), 137.2 (C^{7}H), 136.9 (C^{12}), 133.8 (C^{9}), 133.6 (C^{4}), 131.1 (C^{5}H), 130.3 (C^{6}H), 129.3 (C^{13} + C^{17}), 128.7 (C^{15}H), 127.2 (C^{14} + C^{16}), 125.2 (C^{10}), 123.2 (C^{8}H), 60.3 (C^{11}H$_2$), 26.2 (C^{18}H$_2$), 24.9 (C^{21}H$_2$), 21.1 (C^{19}H$_2$), 20.3 (C^{20}H$_2$).

IR (neat) (cm^{-1}): 3026, 2943, 2869, 1634, 1601, 1508, 1455, 1424, 1367, 1030.

N-Methyl-4-butylisoquinolin-ium iodide (4i)

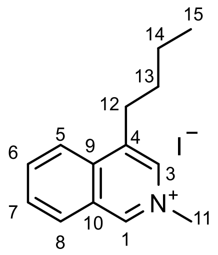

The title compound was prepared according to General Procedure **D** using 4-butylisoquinoline (556 mg, 3.00 mmol) to give salt **4i** (1.09 g, 99 %) as a yellow solid. Spectroscopic data was consistent with that reported in the literature.[4]

^{1}H NMR (400 MHz, $CDCl_3$) δ 10.65 (s, 1H, C^{1}**H**), 8.65 (d, *J* = 8.3 Hz, 1H, C^{5}**H**), 8.54 – 8.48 (m, 1H, C^{3}**H**), 8.19 (dd, *J* = 8.5, 1.1 Hz, 1H, C^{8}**H**), 8.12 (ddd, *J* = 8.5, 6.9, 1.3 Hz, 1H, C^{7}**H**), 7.93 (ddd, *J* = 8.1, 6.8, 1.2 Hz, 1H, C^{6}**H**), 4.76 (s, 3H, C^{11}**H$_3$**), 3.29 – 3.01 (m, 2H, C^{12}**H$_2$**), 1.83 – 1.72 (m, 2H, C^{13}**H$_2$**), 1.48 (h, *J* = 7.3 Hz, 2H, C^{14}**H$_2$**), 0.97 (t, *J* = 7.3 Hz, 3H, C^{15}**H$_3$**).

[9]Prepared according to: Pandey, G., Balakrishnan, M., *J. Org. Chem.* **2008**, 73, 20, 8128 – 8131.

^{13}C NMR (101 MHz, $CDCl_3$) δ 148.6 ($C^{1}H$), 139.7 (C^{9}), 136.87 (C^{4}), 136.84 ($C^{7}H$), 133.7 ($C^{3}H$), 131.8 ($C^{5}H$), 131.1 ($C^{6}H$), 127.8 (C^{10}), 123.6 ($C^{8}H$), 48.8 ($C^{11}H_3$), 32.2 ($C^{13}H_2$), 29.9 ($C^{12}H_2$), 22.8 ($C^{14}H_4$), 13.9 ($C^{15}H_2$).

***N*-(4-Methoxybenzyl)-4-butylisoquinolin-2-ium iodide (4j)**

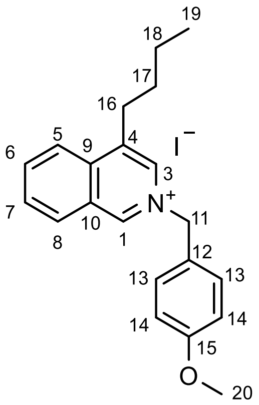

The title compound was prepared according to General Procedure **D** using 4-butylisoquinoline (371 mg, 2.00 mmol) and 4-methoxybenzyl iodide (0.34 mL, 2.40 mmol) to give salt **4j** (986 mg, 99 %) as a white solid. Spectroscopic data was consistent with that reported in the literature.[4]

^{1}H NMR (400 MHz, $CDCl_3$) δ 10.86 (s, 1H, $C^{1}\mathbf{H}$), 8.67 (d, *J* = 8.2 Hz, 1H, $C^{5}\mathbf{H}$), 8.52 – 8.39 (m, 1H, $C^{3}\mathbf{H}$), 8.18 – 8.02 (m, 2H, $C^{8}\mathbf{H}$ + $C^{7}\mathbf{H}$), 7.90 (ddd, *J* = 8.1, 6.5, 1.4 Hz, 1H, $C^{6}\mathbf{H}$), 7.66 (d, *J* = 8.7 Hz, 2H, $C^{13}\mathbf{H_2}$), 6.86 (d, *J* = 8.7 Hz, 2H, $C^{14}\mathbf{H_2}$), 6.17 (s, 2H, $C^{11}\mathbf{H_2}$), 3.76 (s, 3H, $C^{20}\mathbf{H_3}$), 3.25 – 2.96 (m, 2H, $C^{16}\mathbf{H_2}$), 1.79 – 1.68 (m, 2H, $C^{18}\mathbf{H_2}$), 1.43 (h, *J* = 7.4 Hz, 2H, $C^{17}\mathbf{H_2}$), 0.95 (t, *J* = 7.3 Hz, 3H, $C^{19}\mathbf{H_3}$).

^{13}C NMR (101 MHz, $CDCl_3$) δ 160.8 (C^{15}), 147.8 ($C^{1}H$), 139.8 (C^{9}), 137.0 (C^{4}), 136.9 ($C^{7}H$), 132.2 ($C^{5}H$ + $C^{3}H$), 131.4 (2 x $C^{13}H$), 131.0 ($C^{6}H$), 127.9 (C^{10}), 124.9 (C^{12}), 123.6 ($C^{8}H$), 115.0 (2 x $C^{14}H$), 63.4 ($C^{11}H_2$), 55.5 ($C^{20}H_3$), 32.1 ($C^{16}H_2$), 30.1 ($C^{17}H_2$), 22.8 ($C^{18}H_2$), 13.9 ($C^{19}H_3$).

***N*-Benzylisoquinolinium iodide (4k)**

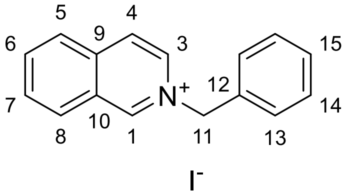

The title compound was prepared according to General Procedure **D** using isoquinoline (1.00 g, 7.74 mmol) and benzyl iodide (1.9 mL, 15.48 mmol) to give salt **4k** (2.6 g, 97%) as a light yellow solid. Spectroscopic data was consistent with that reported in the literature.[4]

^{1}H NMR (400 MHz, DMSO-d_6) δ 10.28 (s, 1H, $C^{1}\mathbf{H}$), 8.83 (dd, *J* = 6.8, 1.5 Hz, 1H $C^{3}\mathbf{H}$), 8.61 (d, *J* = 6.8 Hz, 1H, $C^{4}\mathbf{H}$), 8.54 (d, *J* = 8.47 Hz, 1H, $C^{5}\mathbf{H}$), 8.36 (dd, *J* = 8.3, 1.1 Hz, 1H, $C^{8}\mathbf{H}$), 8.28 (ddd, *J* = 8.3, 7.0, 1.2 Hz, 1H, $C^{7}\mathbf{H}$), 8.10 (ddd, *J* = 8.2, 6.9, 1.2 Hz, 1H, $C^{6}\mathbf{H}$), 7.62 – 7.55 (m, 2H, 2 x $C^{Ar}\mathbf{H}$), 7.50 – 7.38 (m, 3H, 3 x $C^{Ar}\mathbf{H}$), 5.98 (s, 2H, $C^{11}\mathbf{H_2}$).

^{13}C NMR (101 MHz, DMSO-d_6) δ 150.2 ($C^{1}H$), 137.2 ($C^{7}H$), 137.1 (C^{Ar}), 134.8 ($C^{3}H$), 134.3 (C^{Ar}), 131.4 ($C^{6}H$), 130.6 ($C^{5}H$), 129.3 ($C^{15}H$), 129.2 (2 x $C^{Ar}H$), 128.8 (2 x $C^{Ar}H$), 127.4 ($C^{8}H$), 127.3 (C^{Ar}), 126.3 ($C^{4}H$), 63.4 ($C^{11}H_2$).

8-Bromo-*N*-methylisoquinolinium iodide (4l)

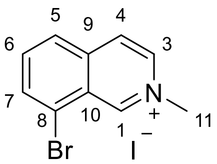

A mixture of the 8-Bromisoquinoline (624 mg, 3.0 mmol, 1.0 eq.) and methyl iodide (0.37 mL, 6.0 mmol, 2.0 eq.) in CH_2Cl_2 (7.5 mL) was stirred at 40 °C for 12 h. The reaction mixture was filtered under reduced pressure. The resulting solid was

washed with ether (50 mL) then dried under vacuum to give isoquinolinium iodide **4l** (439 mg, 42%) as a yellow solid.

m.p. (CH_2Cl_2): 296 °C

HRMS (ESI): Exact mass calculated for $C_{10}H_9BrN+$ $[M]^+$: 221.9913, found: 290.1915.

^{1}H NMR (400 MHz, DMSO-d_6): 9.96 (s, 1H, C^1**H**), 8.81 (dd, *J* = 6.7, 1.3 Hz, 1H, C^3**H**), 8.64 (d, *J* = 6.7 Hz, 1H, C^4**H**), 8.40 (dd, *J* = 7.6, 0.9 Hz, 1H, C^7**H**), 8.36 (d, *J* = 8.4, 1.0 Hz, 1H, C^5**H**), 8.12 (dd, *J* = 8.3, 7.5 Hz, 1H, C^6**H**), 4.57 (s, 3H, C^{11}**H$_3$**);

^{13}C NMR (101 MHz, DMSO-d_6) δ 150.0 (C^{1}H), 138.8 (C^{Ar}), 137.2 (C^{3}H + C^{6}H), 135.3 (C^{7}H), 127.3 (C^{5}H), 126.4 (C^{Ar}), 126.0 (C^{4}H), 123.2 (C^{Ar}), 48.4 (C^{11}H$_3$);

IR (neat) (cm^{-1}): 3043, 3002, 1646, 1558, 1359, 1337, 1185, 851, 790, 745, 671.

_N_-Methyl-5-((4-tosyl-1,4-diazepan-1-yl)sulfonyl)isoquinolinium iodide (4m)

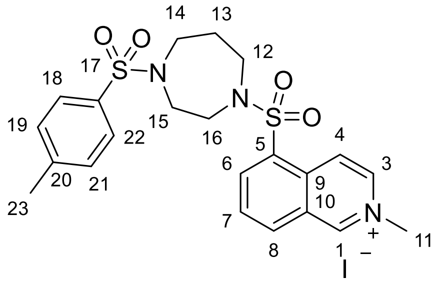

A mixture of Fasudil (560 mg, 1.9 mmol, 1.0 eq.), *N*,*N*-diisopropylethylamine (0.1 mL, 3.8 mmol, 2.0 eq.) and *p*-toluenesulfonyl chloride (544 mg, 2.85 mmol, 1.5 eq.) in CH_2Cl_2 (20 mL) was stirred at room temperature overnight. After the addition of H_2O (10 mL) the aqueous phase was extracted with CH_2Cl_2 (3 x 10 mL). Removal of the solvent and flash column chromatography (10% EtOAc in pentane → 100% EtOAc) delivered the tosylated isoquinoline **4m'** (450 mg, 53%) as a white solid. Subsequently **4m'** (446 mg, 1.0 mmol, 1.0 eq.) was directly used in General Procedure **D** with methyl iodide (0.12 mL, 2.0 mmol, 2.0 eq.) in CH_2Cl_2 (2.0 mL) to give salt **4m** (394 mg, 67%) as a yellow solid.

m.p. (CH_2Cl_2): 210 °C

HRMS (ESI): Exact mass calculated for $C_{22}H_{26}N_3O_4S_2$ $[M]^+$: 460.1359, found: 460.1356.

^{1}H NMR (400 MHz, DMSO-d_6) δ 10.20 (s, 1H, C^1**H**), 8.92 (d, *J* = 7.1 Hz, 1H, C^3**H**), 8.84 (dd, *J* = 7.2, 1.5 Hz, 1H, C^4**H**), 8.76 (d, *J* = 8.4 Hz, 1H, C$^{6/8}$**H**), 8.71 (dd, *J* = 7.5, 1.2 Hz, 1H, C$^{6/8}$**H**), 8.19 (t, *J* = 8.4, 1H, C^7**H**), 7.66 (d, *J* = 8.3 Hz, 2H, C^{18}**H** + C^{22}**H**), 7.42 (d, *J* = 7.7 Hz, 2H, C^{19}**H** + C^{21}**H**), 4.52 (s, 3H, C^{11}**H$_3$**), 3.61 – 3.55 (m, 2H, C$^{12/14/15/16}$**H$_2$**), 3.50 (t, *J* = 5.9 Hz, 2H, C$^{12/14/15/16}$**H$_2$**), 3.25 (t, *J* = 5.9 Hz, 2H, C$^{12/14/15/16}$**H$_2$**), 2.40 (s, 3H, C^{23}**H$_3$**), 1.88 – 1.77 (m, 2H (C^{13}**H$_2$**).; **N.B**. one C$^{12/14/15/16}$**H$_2$** under the DMSO signal.

^{13}C NMR (101 MHz, DMSO-d_6) δ 151.7 (C^{1}H), 143.4 (C^{Ar}), 138.0 (C^{4}H), 137.3 (C$^{6/8}$H), 135.7 (C$^{6/8}$H), 135.5 (C^{Ar}), 134.8 (C^{Ar}), 132.4 (C^{Ar}), 130.5 (C^{7}H), 130.0 (C^{19}H + C^{21}H), 128.5 (C^{Ar}), 126.7 (C^{18}H + C^{22}H), 122.2 (C^{10}H), 49.2 (C$^{12/14/15/16}$H$_2$), 49.1 (C$^{12/14/15/16}$H$_2$), 48.1 (C^{11}H$_3$), 46.8 (C$^{12/14/15/16}$H$_2$), 46.8 (C$^{12/14/15/16}$H$_2$), 29.3 (C^{13}H$_2$), 21.0 (C^{23}H$_3$);

IR (neat) (cm^{-1}): 2930, 2154, 2033, 1315, 1148, 1131, 978, 905, 813, 807, 701.

Synthesis of tetrahydroisoquinolines

General procedure E: Synthesis of tetrahydroisoquinolines

Isoquinolinium salt (0.125 mmol, 1.00 equiv.) and electrophile (1.00 to 5.00 equiv.) were dissolved in MeCN (0.10 mL, 1.25 M). Upon addition of $HCO_2H:NEt_3$ 5:2 complex (42 µL, 4.00 equiv.) the reaction mixture was heated to 80 ˚C for 18 hours. The reaction was diluted with CH_2Cl_2 (10 mL) and quenched with an aqueous solution of K_2CO_3 (10 mL, 0.1 M). The solution was separated, and the aqueous layer was extracted with CH_2Cl_2 (3 x 10 mL). The organic layers were combined, dried ($MgSO_4$), filtered, and concentrated *in vacuo*. The crude material was purified by flash column chromatography to furnish the respective amines

General procedure F: Rhodium catalysed synthesis of tetrehydroisoquinolines

Isoquinolinium salt (1.00 equiv.), and $[RhCp^*Cl_2]_2$ (0.01 mol%) was added to a microwave vial. MeCN (1.25 M), electrophile (1.0-2.0 equiv.) and $HCO_2H:NEt_3$ (5:2, 4.0 equiv.) were added and the solution heated at 75 °C for 18 hours. The reaction was diluted with CH_2Cl_2 (10 mL) and quenched with an aqueous solution of K_2CO_3 (10 mL, 0.1 M). The solution was separated, and the aqueous layer was extracted with CH_2Cl_2 (3 x 10 mL). The organic layers were combined, dried ($MgSO_4$), filtered, and concentrated *in vacuo*. The crude material was purified by flash column chromatography to furnish the respective amines.

***N*-Benzyl-4-(butan-2-one)-4-methyl-1,2,3,4-tetrahydroisoquinoline** (**5a**)

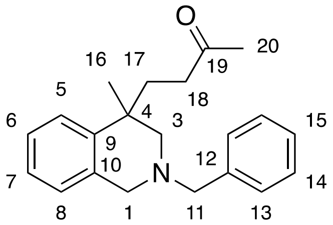

The title compound was prepared by General Procedure **E** using *N*-benzyl-4-methylisoquinolinium iodide **4a** (45 mg, 0.125 mmol) and methyl vinyl ketone (21 µL, 0.250 mmol). Purification by column chromatography (5% EtOAc in pentane) gave amine **5a** (33 mg, 86%) as a yellow oil.

HRMS (ESI): Exact mass calculated for $C_{21}H_{26}ON$ $[M+H]^+$: 308.2009, found 308.2011.

^{1}H NMR (400 MHz, $CDCl_3$) δ 7.41 – 7.21 (m, 6H, 6 x C^{Ar}**H**), 7.21 – 7.14 (m, 1H, C^{Ar}**H**), 7.10 (td, *J* = 7.3, 1.5 Hz, 1H, C^{Ar}**H**), 6.99 (ddd, *J* = 7.6, 1.6, 0.7 Hz, 1H, C^{Ar}**H**), 3.74 – 3.49 (m, 2H, $C^{1/11}$**H**$_2$), 3.61 (q, *J* = 13.0 Hz, 2H, $C^{1/11}$**H**$_2$), 2.57 (dd, *J* = 11.5, 1.3 Hz, 1H, C^3**H**$_2$), 2.45 (ddd, *J* = 16.1, 11.7, 4.9 Hz, 1H, C^{18}**H**$_2$), 2.30 (dd, *J* = 11.4, 0.8 Hz, 1H, C^3**H**$_2$), 2.12 – 1.99 (m, 2H, C^{17}**H**$_2$+ C^{18}**H**$_2$), 2.02 (s, 3H, C^{20}**H**$_3$), 1.80 (ddd, *J* = 15.1, 11.8, 4.9 Hz, 1H, C^{17}**H**$_2$), 1.24 (s, 3H, C^{16}**H**$_3$)

^{13}C NMR (101 MHz, $CDCl_3$) δ 209.3 ($C^{19}O$), 142.2 (C^Q), 138.7 (C^Q), 134.9 (C^Q), 129.2 (2 x C^{Ar}), 128.4 (2 x C^{Ar}), 127.3 (C^{Ar}), 126.7 (C^{Ar}), 126.5 (C^{Ar}), 126.2 (C^{Ar}), 125.8 (C^{Ar}), 63.0 ($C^{1/11}H_2$), 61.8 (C^3H_2), 57.6 ($C^{1/11}H_2$), 39.7 ($C^{18}H_2$), 37.8 (C^4), 36.1 ($C^{17}H_2$), 30.1 ($C^{20}H_3$), 27.8 ($C^{16}H_3$)

IR (neat) (cm^{-1}): 2927, 2799, 1714, 1493, 1452, 1162, 1096, 914, 760, 700.

***N*-Benzyl--4-methyl-4-(pentan-3-one)-1,2,3,4-tetrahydroisoquinoline (5b)**

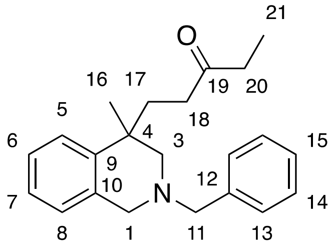

The title compound was prepared by General Procedure **E** using *N*-benzyl-4-methylisoquinolinium iodide **4a** (45 mg, 0.125 mmol) and ethyl vinyl ketone (25 µL, 0.250 mmol). Purification by flash column chromatography (5% EtOAc in pentane) gave amine **5b** (37 mg, 92%) as a yellow oil.

HRMS (ESI): Exact mass calculated for $C_{22}H_{28}ON$ $[M+H]^+$: 322.2165, found 322.2165.

^{1}H NMR (500 MHz, $CDCl_3$) δ 7.45 – 7.40 (m, 2H, 2 x C^{Ar}**H**), 7.40 – 7.33 (m, 2H, 2 x C^{Ar}**H**), 7.33 – 7.24 (m, 2H, 2 x C^{Ar}**H**), 7.21 (td, *J* = 7.5, 1.5 Hz, 1H, C^{Ar}**H**), 7.14 (td, *J* = 7.4, 1.4 Hz, 1H, C^{Ar}**H**), 7.02 (dd, *J* = 7.6, 1.4 Hz, 1H, C^{Ar}**H**), 3.80 – 3.49 (m, 4H, $C^1\mathbf{H}_2$ + $C^{11}\mathbf{H}_2$), 2.61 (dd, *J* = 11.4, 1.2 Hz, 1H, $C^3\mathbf{H}_2$), 2.45 (ddd, *J* = 16.3, 12.3, 4.9 Hz, 1H, $C^{18}\mathbf{H}_2$), 2.38 – 2.27 (m, 3H, $C^3\mathbf{H}_2$ + $C^{20}\mathbf{H}_2$), 2.16 – 2.02 (m, 2H, $C^{18}\mathbf{H}_2$ + $C^{17}\mathbf{H}_2$), 1.84 (ddd, *J* = 15.9, 12.2, 4.9 Hz, 1H, $C^{17}\mathbf{H}_2$), 1.28 (s, 3H, $C^{16}\mathbf{H}_3$), 1.02 (t, *J* = 7.3 Hz, 3H, $C^{21}\mathbf{H}_3$)

^{13}C NMR (126 MHz, $CDCl_3$) δ 211.9 ($C^{19}O$), 142.2 (C^Q), 138.7 (C^Q), 134.9 (C^Q), 129.2 (2 x C^{Ar}), 128.4 (2 x C^{Ar}), 127.2 (C^{Ar}), 126.6 (C^{Ar}), 126.5 (C^{Ar}), 126.2 (C^{Ar}), 125.7 (C^{Ar}), 63.0 ($C^{1/11}H_2$), 61.8 (C^3H_2), 57.6 ($C^{1/11}H_2$), 38.3 ($C^{18}H_2$), 37.8 (C^4), 36.1 ($C^{17}H_2$), 35.9 ($C^{20}H_2$), 27.9 ($C^{16}H_3$), 8.0 ($C^{21}H_3$)

IR (neat) (cm^{-1}): 2932, 2798, 1713, 1493, 1453, 1368, 1346, 1097, 759, 700.

***N*-Benzyl-4-methyl-4-(1-phenylpropan-1-one)-1,2,3,4-tetra-hydroisoquinoline (5c)**

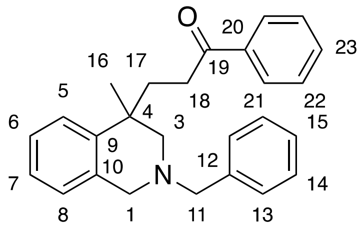

The title compound was prepared by General Procedure **E** using *N*-benzyl-4-methylisoquinolinium iodide **4a** (45 mg, 0.125 mmol) and phenyl vinyl ketone (33 mg, 0.250 mmol). Purification by flash column chromatography (2.5% EtOAc in pentane) gave amine **5c** (40 mg, 87%) as a yellow oil.

HRMS (ESI): Exact mass calculated for $C_{26}H_{28}ON$ $[M+H]^+$: 370.2165, found 370.2165.

^{1}H NMR (400 MHz, $CDCl_3$) δ 7.78 – 7.69 (m, 2H, 2 x C^{Ar}**H**), 7.48 – 7.39 (m, 1H, C^{Ar}**H**), 7.37 – 7.24 (m, 4H, 4 x C^{Ar}**H**), 7.24 – 7.13 (m, 4H, 4 x C^{Ar}**H**), 7.13 – 7.07 (m, 1H, C^{Ar}**H**), 7.03 (td, *J* = 7.4, 1.4 Hz, 1H, C^{Ar}**H**), 6.94 – 6.88 (m, 1H, C^{Ar}**H**), 3.64 (d, *J* = 14.6 Hz, 1H, $C^1\mathbf{H}_2$), 3.53 (s, 2H, $C^{11}\mathbf{H}_2$), 3.43 (d, *J* = 14.6 Hz, 1H, $C^1\mathbf{H}_2$), 2.98 (ddd, *J* = 16.4, 11.3, 4.9 Hz, 1H, $C^{18}\mathbf{H}_2$), 2.63 (d, *J* = 11.3, 1H, $C^3\mathbf{H}_2$), 2.52 (ddd, *J* = 16.8, 11.2, 4.8 Hz, 1H, $C^{18}\mathbf{H}_2$), 2.29 (d, *J* = 11.5 Hz, 1H, $C^3\mathbf{H}_2$), 2.16 (ddd, *J* = 14.1, 11.3, 4.8 Hz, 1H, $C^{17}\mathbf{H}_2$), 2.01 – 1.85 (m, 1H, $C^{17}\mathbf{H}_2$), 1.23 (s, 3H, $C^{16}\mathbf{H}_3$)

^{13}C NMR (101 MHz, $CDCl_3$) δ 200.8 ($C^{19}O$), 142.2 (C^Q), 138.6 (C^Q), 137.2 (C^Q), 135.0 (C^Q), 132.9 (C^{Ar}), 129.2 (2 x C^{Ar}), 128.6 (2 x C^{Ar}), 128.4 (2 x C^{Ar}), 128.1 (2 x C^{Ar}), 127.3 (C^{Ar}), 126.7 (C^{Ar}), 126.6 (C^{Ar}), 126.2 (C^{Ar}), 125.8 (C^{Ar}), 63.1 ($C^{11}H_2$), 62.3 (C^3H_2), 57.5 (C^1H_2), 38.0 (C^4), 37.0 ($C^{17}H_2$), 34.8 ($C^{18}H_2$), 28.1 ($C^{16}H_3$)

IR (neat) (cm^{-1}): 2925, 2854, 1681, 1597, 1449, 1273, 1240, 1113, 760, 700.

N-Benzyl-4-methyl-4-(1-pentamethylphenylpropan-1-one)-1,2,3,4-tetra-hydroisoquinoline (5d)

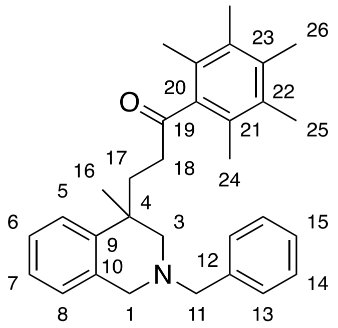

The title compound was prepared by General Procedure **E** using N-benzyl-4-methylisoquinolinium iodide **4a** (45 mg, 0.125 mmol) and pentamethylphenyl vinyl ketone (51 mg, 0.250 mmol). Purification by flash column chromatography (CH_2Cl_2) gave amine **5d** (51 mg, 92%) as a yellow oil.

HRMS (ESI): Exact mass calculated for $C_{31}H_{38}ON$ $[M+H]^+$: 440.2948, found 440.2952.

^{1}H NMR (400 MHz, $CDCl_3$) δ 7.30 – 7.14 (m, 6H, 6 x C^{Ar}**H**), 7.08 (td, J = 7.6, 1.5 Hz, 1H, C^{Ar}**H**), 6.99 (td, J = 7.4, 1.3 Hz, 1H, C^{Ar}**H**), 6.86 (dd, J = 7.6, 1.4 Hz, 1H, C^{Ar}**H**), 3.58 – 3.39 (m, 4H, C^1**H**$_2$ + C^{11}**H**$_2$), 2.76 – 2.63 (m, 1H, C^{18}**H**$_2$), 2.46 (d, J = 11.3 Hz, 1H, C^3**H**$_2$), 2.32 (d, J = 11.3 Hz, 1H, C^3**H**$_2$), 2.35 – 2.06 (m, 2H, C^{17}**H**$_2$ + C^{18}**H**$_2$), 2.12 (s, 3H, C^{26}**H**$_3$), 2.06 (s, 6H, 2 x C^{24}**H**$_3$), 1.90 (s, 6H, 2 x C^{25}**H**$_3$), 1.99 – 1.77 (m, 1H, C^{17}**H**$_2$), 1.24 (s, 3H, C^{16}**H**$_3$)

^{13}C NMR (101 MHz, $CDCl_3$) δ 212.4 (C^{19}), 141.8 (C^Q), 141.2 (C^Q), 138.6 (C^Q), 135.4 (C^Q), 135.0 (C^Q), 133.1 (2 x C^Q), 129.0 (2 x C^{Ar}), 128.4 (2 x C^{Ar}), 127.4 (2 x C^Q), 127.2 (C^{Ar}), 126.7 (C^{Ar}), 126.6 (C^{Ar}), 126.1 (C^{Ar}), 125.8 (C^{Ar}), 63.3 ($C^{11}H_2$), 62.6 (C^3H_2), 57.5 (C^1H_2), 41.9 ($C^{18}H_2$), 37.9 (C^4), 35.5 ($C^{17}H_2$), 28.9 ($C^{16}H_3$), 17.3 (2 x $C^{24/25}H_3$), 16.8 ($C^{26}H_3$), 16.0 (2 x $C^{24/25}H_3$)

IR (neat) (cm^{-1}): 2926, 2800, 1698, 1493, 1452, 1311, 910, 759, 730, 700.

N-Benzyl-4-methyl-4-propanal-1,2,3,4-tetra-hydroisoquinoline (**5e**)

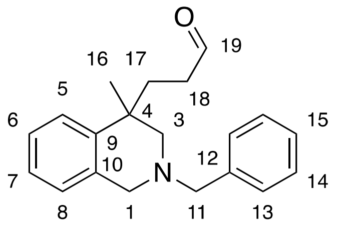

The title compound was prepared by General Procedure **F** using N-benzyl-4-methylisoquinolinium iodide **4a** (45 mg, 0.125 mmol), acrolein (17 µL, 0.250 mmol), HCO_2H:NEt_3 (5:2) (42 µL, 0.500 mmol), and 0.1 mL of the catalyst stock solution (corresponding to 0.01 mol% of $[RhCp^*Cl_2]_2$) was added to a sealed vial and the solution was heated at 47 °C for 20 hours. The reaction mixture was diluted with dichloromethane (10 mL) and quenched with potassium carbonate (10 mL, 0.1 M). The phases were separated, and the aqueous layer was extracted with dichloromethane (3 x 10 mL). The combined organic layers were concentrated *in vacuo*. The crude material was purified by flash column chromatography (15-20% EtOAc in pentane) to give *amine* **5e** (29 mg, 79%) as a yellow oil.

The title compound could not be prepared according to General procedure **E** and only starting material was recovered from the reaction.

HRMS (ESI): Exact mass calculated for $C_{20}H_{24}ON$ $[M+H]^+$: 294.1852, found 294.1853.

^{1}H NMR (400 MHz, $CDCl_3$) δ 9.53 (t, J = 1.6 Hz, 1H, C^{19}**H**), 7.33 – 7.13 (m, 6H, 6 x C^{Ar}**H**), 7.10 (dddd, J = 8.5, 7.8, 1.5, 0.7 Hz, 1H, C^{Ar}**H**), 7.03 (td, J = 7.3, 1.5 Hz, 1H, C^{Ar}**H**), 6.91 (ddt, J = 7.5, 1.5, 0.7 Hz, 1H, C^{Ar}**H**), 3.65 (d, J = 14.7 Hz, 1H, C^{1}**H**$_a$), 3.52 (s, 2H, C^{11}**H**$_2$), 3.42 (d, J = 14.7 Hz, 1H, C^{1}**H**$_b$), 2.53 (d, J = 11.5, 1H, C^{3}**H**$_a$), 2.45 – 2.31 (m, 1H, C^{18}**H**$_2$), 2.23 (d, J = 11.5 Hz, 1H, C^{3}**H**$_b$), 2.09 – 1.94 (m, 2H, C^{17}**H**$_a$ + C^{18}**H**$_2$), 1.88 – 1.76 (m, 1H, C^{17}**H**$_b$), 1.17 (s, 3H, C^{16}**H**$_3$)

^{13}C NMR (101 MHz, $CDCl_3$) δ 202.2 (C^{19}O), 141.8 (C^{Q}), 138.4 (C^{Q}), 134.9 (C^{Q}), 129.3 (2 x C^{Ar}), 128.4 (2 x C^{Ar}), 127.3 (C^{Ar}), 126.7 (C^{Ar}), 126.6 (C^{Ar}), 126.1 (C^{Ar}), 126.0 (C^{Ar}), 63.0 (C^{11}H$_2$), 62.0 (C^{3}H$_2$), 57.3 (C^{1}H$_2$), 40.1 (C^{18}H$_2$), 37.7 (C^{4}), 34.6 (C^{17}H$_2$), 27.5 (C^{16}H$_3$)

IR (neat) (cm^{-1}): 2926, 1721, 1683, 1493, 1453, 1369, 910, 760, 731, 700.

***N*-Benzyl-4-methyl-4-(dimethyl methylmalonate)- 1,2,3,4-tetra-hydroisoquinoline** (**5f**)

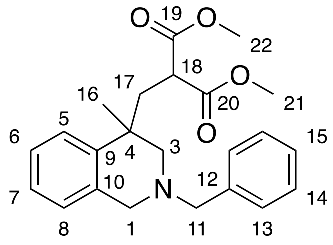

The title compound was prepared by General Procedure **E** using *N*-benzyl-4-methylisoquinolinium iodide **4a** (45 mg, 0.125 mmol) and dimethyl 2-methylenemalonate (36 mg, 0.25 mmol). Purification by column chromatography (4-6% EtOAc in pentane) gave *amine* **5f** (15 mg, 34%) as a yellow oil. General procedure **E** afforded the title compound in a significantly reduced yield of 11%.

HRMS (ESI): Exact mass calculated for $C_{23}H_{28}O_4N$ $[M+H]^+$: 382.2013, found 382.2011.

^{1}H NMR (500 MHz, $CDCl_3$) δ 7.42 – 7.26 (m, 6H, 6 x C^{Ar}**H**), 7.23 – 7.16 (m, 1H, C^{Ar}**H**), 7.11 (td, J = 7.4, 1.3 Hz, 1H, C^{Ar}**H**), 7.00 – 6.95 (m, 1H, C^{Ar}**H**), 3.87 (t, J = 6.1 Hz, 1H, C^{18}**H**), 3.82 – 3.72 (m, 1H, C$^{1/11}$**H**$_2$), 3.69 (s, 3H, C$^{21/22}$**H**$_3$), 3.72 – 3.64 (m, 1H, C$^{1/11}$**H**$_2$), 3.62 – 3.53 (m, 1H, C$^{1/11}$**H**$_2$), 3.48 (s, 3H, C$^{21/22}$**H**$_3$), 3.40 (d, J = 14.7 Hz, 1H, C$^{1/11}$**H**$_2$), 2.79 – 2.72 (m, 1H, C^{3}**H**$_2$), 2.49 (dd, J = 14.5, 6.2 Hz, 1H, C^{17}**H**$_2$), 2.42 – 2.33 (m, 2H, C^{3}**H**$_2$ + C^{17}**H**$_2$), 1.27 (s, 3H, C^{16}**H**$_3$)

^{13}C NMR (126 MHz, $CDCl_3$) δ 170.68 (C^{19}O/C^{20}O), 170.65 (C^{19}O/C^{20}O), 140.4 (C^{Q}), 138.2 (C^{Q}), 135.2 (C^{Q}), 129.1 (2 x C^{Ar}), 128.4 (2 x C^{Ar}), 127.2 (C^{Q}), 126.8 (C^{Ar}), 126.6 (C^{Ar}), 126.5 (C^{Ar}), 126.1 (C^{Ar}), 62.97 (C^{3}H$_2$/C$^{1/11}$H$_2$), 62.95 (C^{3}H$_2$/C$^{1/11}$H$_2$), 56.9 (C$^{1/11}$H$_2$), 52.7 (C$^{21/22}$H$_3$), 52.5 (C$^{21/22}$H$_3$), 48.7 (C^{18}H), 41.7 (C^{17}H$_2$), 37.9 (C^{4}), 28.1 (C^{16}H$_3$)

IR (neat) (cm^{-1}): 2952, 2925, 1735, 1494, 1435, 1266, 1150, 1028, 762, 701.

N-Benzyl-4-methyl-4-(2,5-dioxopyrrolidin-3-yl)-1,2,3,4-tetrahydroisoquinoline (5g)

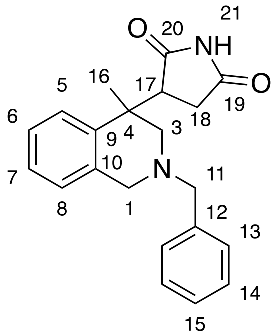

The title compound was prepared by General Procedure **E** using *N*-benzyl-4-methylisoquinolinium iodide **4a** (45 mg, 0.125 mmol) and maleimide (24 mg, 0.25 mmol). Purification by column chromatography (10-33% EtOAc in pentane) gave *amine* **5g** (25 mg, 59%) as an inseparable 2:1 diastereomeric mixture as a yellow oil.

HRMS (ESI): Exact mass calculated for $C_{21}H_{23}O_2N_2$ $[M+H]^+$: 335.1754 found 335.1753.

Major diastereomer: ^{1}**H NMR** (600 MHz, $CDCl_3$) δ 7.91 (d, *J* = 7.4 Hz, 1H, N^{21}**H**), 7.42 – 6.98 (m, 9H, 9 x C^{Ar}**H**), 3.79 (d, *J* = 14.7 Hz, 1H, C$^{1/11}$**H**$_2$), 3.61 (d, *J* = 12.6 Hz, 1H, C$^{1/11}$**H**$_2$), 3.48 (d, *J* = 7.2 Hz, 1H, C$^{1/11}$**H**$_2$), 3.46 (d, *J* = 5.0 Hz, 1H, C$^{1/11}$**H**$_2$), 3.20 (dd, *J* = 9.6, 4.9 Hz, 1H, C^{17}**H**), 3.03 – 2.96 (m, 1H, C^{18}**H**$_2$), 2.93 (d, *J* = 12.2 Hz, 1H, C^3**H**$_2$), 2.40 (d, *J* = 13.5 Hz, 1H, C^3**H**$_2$), 2.30 – 2.23 (m, 1H, C^{18}**H**$_2$), 1.64 (s, 3H, C^{16}**H**$_3$)

^{13}**C NMR** (151 MHz, $CDCl_3$) δ 178.5 (C$^{19/20}$O), 176.9 (C$^{19/20}$O), 140.2 (C^Q), 137.7 (C^Q), 134.8 (C^Q), 129.6 (2 x C^{Ar}), 128.5 (2 x C^{Ar}), 127.5 (C^{Ar}), 127.3 (C^{Ar}), 126.9 (C^{Ar}), 126.7 (C^{Ar}), 125.8 (C^{Ar}), 63.2 (C$^{1/11}$H$_2$), 59.7 (C^{3}H$_2$), 57.4 (C$^{1/11}$H$_2$), 51.8 (C^{17}H), 40.0 (C^4), 35.4 (C^{18}H$_2$), 27.0 (C^{16}H$_3$).

Minor diastereomer: ^{1}**H NMR** (600 MHz, $CDCl_3$) δ 8.22 (s, 1H, N^{21}**H**), 7.42 – 6.98 (m, 9H, 9 x C^{Ar}**H**), 3.87 (d, *J* = 15.0 Hz, 1H, C$^{1/11}$**H**$_2$), 3.76 (d, *J* = 12.9 Hz, 1H, C$^{1/11}$**H**$_2$), 3.60 (d, *J* = 12.9 Hz, 1H, C$^{1/11}$**H**$_2$), 3.45 – 3.39 (m, 2H, C$^{1/11}$**H**$_2$ + C^{17}**H**), 3.26 (d, *J* = 11.7 Hz, 1H, C^3**H**$_2$), 2.90 – 2.83 (m, 1H, C^{18}**H**$_2$), 2.63 – 2.55 (m, 1H, C^{18}**H**$_2$), 2.40 (d, *J* = 11.7 Hz, 1H, C^3**H**$_2$), 1.45 (s, 3H, C^{16}**H**$_3$)

^{13}**C NMR** (151 MHz, $CDCl_3$) δ 178.5 (C$^{19/20}$O), 176.8 (C$^{19/20}$O), 138.6 (C^Q), 138.0 (C^Q), 135.4 (C^Q), 129.3 (C^{Ar}), 129.2 (C^{Ar}), 128.4 (C^{Ar}), 127.4 (C^{Ar}), 127.0 (C^{Ar}), 126.9 (C^{Ar}), 126.6 (C^{Ar}), 126.2 (C^{Ar}), 125.4 (C^{Ar}), 63.0 (C$^{1/11}$H$_2$), 61.8 (C^{3}H$_2$), 56.7 (C$^{1/11}$H$_2$), 50.0 (C^{17}H), 40.8 (C^4), 33.6 (C^{18}H$_2$), 22.9 (C^{16}H$_3$).

IR (neat) (cm^{-1}): 3213, 2805, 1778, 1701, 1494, 1349, 1182, 910, 730, 700.

N-Benzyl-4-methyl-4-(N-phenyl-2,5-dioxopyrrolidin-3-yl)-1,2,3,4-tetrahydroisoquinoline (5h)

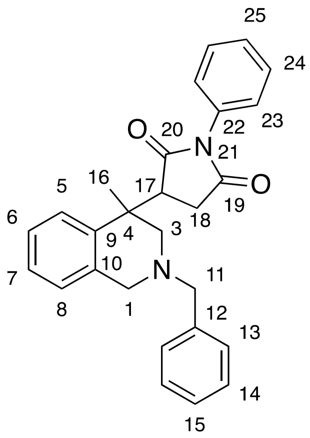

The title compound was prepared by General Procedure **E** using *N*-benzyl-4-methylisoquinolinium iodide **4a** (45 mg, 0.125 mmol) and *N*-phenylmaleimide (43 mg, 0.25 mmol). Purification by column chromatography (CH_2Cl_2) gave *amine* **5h** (31 mg, 60%) as an inseparable 2:1 diastereomeric mixture as a yellow oil.

HRMS (ESI): Exact mass calculated for $C_{27}H_{27}O_2N_2$ $[M+H]^+$: 411.2067, found 411.2066.

Major diastereomer: ^{1}**H NMR** (600 MHz, $CDCl_3$) δ 7.53 – 7.00 (m, 14H, 14 x C^{Ar}**H**), 3.76 (t, *J* = 14.1 Hz, 1H, C$^{1/11}$**H**$_2$), 3.67 (d, *J* = 12.9 Hz, 1H, C$^{1/11}$**H**$_2$),

3.55 (d, *J* = 12.8 Hz, 1H, C$^{1/11}$**H**$_2$), 3.45 (d, *J* = 14.7 Hz, 1H, C$^{1/11}$**H**$_2$), 3.39 (dd, *J* = 9.7, 4.7 Hz, 1H, C^{17}**H**), 3.19 – 3.11 (m, 1H, C^{18}**H**$_2$), 2.97 (d, *J* = 12.1, 1H, C^{3}**H**$_2$), 2.54 (d, *J* = 12.1 Hz, 1H, C^{3}**H**$_2$), 2.51 – 2.43 (m, 1H, C^{18}**H**$_2$), 1.72 (s, 3H, C^{16}**H**$_3$)

^{13}C NMR (101 MHz, CDCl$_3$) δ 177.4 (C$^{19/20}$O), 175.9 (C$^{19/20}$O), 140.2 (C^{Q}), 137.6 (C^{Q}), 134.8 (C^{Q}), 132.1 (C^{Q}), 129.2 (2 x C^{Ar}), 129.2 (2 x C^{Ar}), 128.7 (2 x C^{Ar}), 128.6 (C^{Ar}), 128.5 (C^{Ar}), 127.6 (C^{Ar}), 127.0 (C^{Ar}), 126.7 (C^{Ar}), 126.6 (2 x C^{Ar}), 125.9 (C^{Ar}), 63.6 (C$^{1/11}$H$_2$), 60.3 (C^{3}H$_2$), 56.9 (C$^{1/11}$H$_2$), 50.4 (C^{17}H), 40.6 (C^{4}), 34.3 (C^{18}H$_2$), 27.2 (C^{16}H$_3$).

Minor diastereomer: **^{1}H NMR** (600 MHz, CDCl$_3$) δ 7.53 – 7.00 (m, 14H, 14 x C^{Ar}**H**), 3.90 (d, *J* = 14.9 Hz, 1H, C$^{1/11}$**H**$_2$), 3.76 (t, *J* = 14.1 Hz, 1H, C$^{1/11}$**H**$_2$), 3.67 (d, *J* = 12.9 Hz, 1H, C$^{1/11}$**H**$_2$), 3.46 (dd, *J* = 15.1, 6.6 Hz, 2H, C$^{1/11}$**H**$_2$ + C^{17}**H**), 3.31 – 3.24 (m, 1H, C^{3}**H**$_2$), 3.19 – 3.11 (m, 1H, C^{18}**H**$_2$), 2.79 – 2.71 (m, 1H, C^{18}**H**$_2$), 2.51 – 2.43 (m, 1H, C^{3}**H**$_2$), 1.54 (s, 3H, C^{16}**H**$_3$)

^{13}C NMR (101 MHz, CDCl$_3$) δ 177.4 (C$^{19/20}$O), 175.9 (C$^{19/20}$O), 138.3 (C^{Q}), 137.9 (C^{Q}), 135.6 (C^{Q}), 132.2 (C^{Q}), 129.4 (2 x C^{Ar}), 129.2 (4 x C^{Ar}), 128.6 (C^{Ar}), 127.5 (C^{Ar}), 127.3 (2 x C^{Ar}), 127.1 (2 x C^{Ar}), 126.6 (C^{Ar}), 126.3 (C^{Ar}), 63.0 (C$^{1/11}$H$_2$), 62.7 (C^{3}H$_2$), 56.7 (C$^{1/11}$H$_2$), 49.1 (C^{17}H), 41.1 (C^{4}), 32.7 (C^{18}H$_2$), 23.3 (C^{16}H$_3$).

IR (neat) (cm^{-1}): 2926, 1774, 1707, 1499, 1380, 1180, 911, 733, 698, 623.

***N*-Benzyl-4-(butan-2-one)-4-butyl-1,2,3,4-tetrahydroisoquinoline** (**5i**)

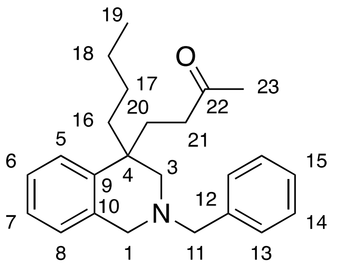

The title compound was prepared by General Procedure **E** using *N*-benzyl-4-butylisoquinolinium iodide **4b** (50 mg, 0.125 mmol) and methyl vinyl ketone (21 µL, 0.25 mmol). Purification by column chromatography (5% EtOAc in pentane) gave *amine* **5i** (33 mg, 76%) as a yellow oil.

HRMS (ESI): Exact mass calculated for $C_{24}H_{32}ON$ $[M+H]^{+}$: 350.2478, found 350.2478.

^{1}H NMR (500 MHz, CDCl$_3$) δ 7.45 – 7.33 (m, 4H, 4 x C^{Ar}**H**), 7.33 – 7.26 (m, 1H, C^{Ar}**H**), 7.23 – 7.16 (m, 2H, 2 x C^{Ar}**H**), 7.16 – 7.08 (m, 1H, C^{Ar}**H**), 7.01 (d, *J* = 7.6 Hz, 1H, C^{Ar}**H**), 3.75 – 3.45 (m, 4H, C^{1}**H**$_2$ + C^{11}**H**$_2$), 2.58 – 2.46 (m, 3H, C^{3}**H**$_2$ + C^{21}**H**$_2$), 2.22 – 1.99 (m, 2H, C^{20}**H**$_2$ + C^{21}**H**$_2$), 2.03 (s, 3H, C^{23}**H**$_3$), 1.83 (ddd, *J* = 14.0, 11.7, 4.9 Hz, 1H, C^{20}**H**$_2$), 1.80 – 1.70 (m, 1H, C^{16}**H**$_2$), 1.53 (ddd, *J* = 13.8, 12.2, 3.8 Hz, 1H, C^{16}**H**$_2$), 1.27 – 1.18 (m, 3H, C^{17}**H**$_2$ + C^{18}**H**$_2$), 1.00 - 0.89 (m, 1H, C^{17}**H**$_2$), 0.85 (t, *J* = 7.1 Hz, 3H, C^{19}**H**$_3$)

^{13}C NMR (126 MHz, CDCl$_3$) δ 209.5 (C^{22}O), 140.9 (C^{Q}), 138.7 (C^{Q}), 135.7 (C^{Q}), 129.3 (2 x C^{Ar}), 128.4 (2 x C^{Ar}), 127.3 (C^{Ar}), 126.6 (C^{Ar}), 126.6 (C^{Ar}), 126.4 (C^{Ar}), 125.6 (C^{Ar}), 63.2 (C$^{1/11}$H$_2$), 59.4 (C^{3}H$_2$), 57.6 (C$^{1/11}$H$_2$), 41.4 (C^{16}H$_2$), 40.7 (C^{4}), 39.8 (C^{21}H$_2$), 35.0 (C^{20}H$_2$), 30.1 (C^{23}H$_3$), 26.5 (C^{17}H$_2$), 23.6 (C^{18}H$_2$), 14.2 (C^{19}H$_3$)

IR (neat) (cm^{-1}): 2930, 1715, 1493, 1453, 1352, 1161, 1093, 919, 757, 700.

N-Benzyl-4-(butan-2-one)-4-isobutyl-1,2,3,4-tetrahydroisoquinoline (5j)

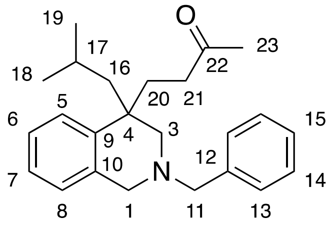

The title compound was prepared by General Procedure **E** using *N*-benzyl-4-isobutylisoquinolinium iodide **4c** (50 mg, 0.125 mmol) and methyl vinyl ketone (21 µL, 0.25 mmol). Purification by column chromatography (3% EtOAc in pentane) gave *amine* **5j** (26.5 mg, 61%) as a yellow oil.

HRMS (ESI): Exact mass calculated for $C_{24}H_{32}ON$ $[M+H]^+$: 350.2478, found 350.2484.

^{1}H NMR (400 MHz, $CDCl_3$) δ 7.32 – 7.15 (m, 5H, 5 x C^{Ar}**H**), 7.15 – 7.04 (m, 2H, 2 x C^{Ar}**H**), 7.00 (td, *J* = 7.3, 1.6 Hz, 1H, C^{Ar}**H**), 6.88 (dd, *J* = 7.5, 1.4 Hz, 1H, C^{Ar}**H**), 3.67 – 3.35 (m, 4H, C^{1}**H$_2$** + C^{11}**H$_2$**), 2.57 – 2.40 (m, 2H, C^{3}**H$_2$**), 2.48 – 2.34 (m, 1H, C^{21}**H$_2$**), 2.05 – 1.93 (m, 1H, C^{21}**H$_2$**), 1.92 (s, 3H, C^{23}**H$_3$**), 1.97 – 1.86 (m, 1H, C^{20}**H$_2$**), 1.72 (ddd, *J* = 13.8, 11.7, 4.9 Hz, 1H, C^{20}**H$_2$**), 1.65 (dd, *J* = 13.8, 5.7 Hz, 1H, C^{16}**H$_2$**), 1.62 – 1.53 (m, 1H, C^{17}**H**), 1.38 (dd, *J* = 13.8, 4.7 Hz, 1H, C^{16}**H$_2$**), 0.78 (d, *J* = 6.5 Hz, 3H, $C^{18/19}$**H$_3$**), 0.56 (d, *J* = 6.5 Hz, 3H, $C^{18/19}$**H$_3$**)

^{13}C NMR (101 MHz, $CDCl_3$) δ 209.5 (C^{22}), 140.7 (C^{Q}), 138.6 (C^{Q}), 135.7, (C^{Q}) 129.3 (2 x C^{Ar}), 128.4 (2 x C^{Ar}), 127.3 (C^{Ar}), 126.7 (C^{Ar}), 126.6 (C^{Ar}), 126.4 (C^{Ar}), 125.6 (C^{Ar}), 63.4 ($C^{1/11}H_2$), 60.1 ($C^{3}H_2$), 57.4 ($C^{1/11}H_2$), 50.4 ($C^{16}H_2$), 41.4 (C^{4}), 39.8 ($C^{21}H_2$), 36.4 ($C^{20}H_2$), 30.1 ($C^{23}H_3$), 25.9 ($C^{18/19}H_3$), 24.9 ($C^{18/19}H_3$), 24.6 ($C^{17}H$)

IR (neat) (cm^{-1}): 3028, 2953, 2807, 1715, 1453, 1365, 1162, 918, 756, 700.

4-(N-Benzyl-4-(p-tolyl)-1,2,3,4-tetrahydroisoquinolin-4-yl)butan-2-one (5k)

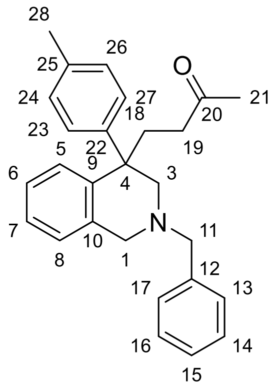

The title compound was prepared according to General Procedure **E** using isoquinolinium salt **4d** (55 mg, 0.125 mmol), methyl vinyl ketone (20 µL, 0.250 mmol), 5:2 HCO_2H:Et_3N (42 µL, 0.500 mmol) in MeCN (0.1 mL) and was purified by column chromatography (4% EtOAc in pentane) to furnish tetrahydroisoquinoline **5k** (24 mg, 50%) as a colourless oil.

HRMS (ESI): Exact mass calculated for $C_{27}H_{30}NO$ $[M+H]^+$: 384.2322, found: 384.2303.

^{1}H NMR (400 MHz, $CDCl_3$) δ 7.22 – 7.14 (m, 5H, 5 x C^{Ar}**H**), 7.09 – 6.96 (m, 7H, 7 x C^{Ar}**H**), 6.80 (dd, *J* = 7.7, 1.5 Hz, 1H, C^{Ar}**H**), 3.72 (d, *J* = 14.7 Hz, 1H, C^{1}**H$_2$**), 3.56 – 3.48 (m, 3H, C^{1}**H$_2$** + C^{11}**H$_2$**) 2.69 (dd, *J* = 11.6, 1.3 Hz, 1H, C^{3}**H$_2$**), 2.60 (d, *J* = 11.8 Hz, 1H, C^{3}**H$_2$**), 2.57 – 2.43 (m, 2H, C^{19}**H$_2$** + C^{18}**H$_2$**), 2.35 – 2.27 (m, 1H, C^{18}**H$_2$**), 2.23 (s, 3H, C^{21}**H$_3$**), 2.18 – 2.09 (m, 1H, C^{19}**H$_2$**), 1.95 (s, 3H, C^{28}**H$_3$**)

^{13}C NMR (101 MHz, $CDCl_3$) δ 209.1 (C^{20}), 144.1 (C^{Ar}), 140.6 (C^{Ar}), 138.3 (C^{Ar}), 135.9 (C^{Ar}), 135.7 ($C^{Ar}H$), 129.1 (2 x $C^{Ar}H$), 129.1 ($C^{Ar}H$), 128.8 (2 x $C^{Ar}H$), 128.3 (2 x $C^{Ar}H$), 128.0 (2 x $C^{Ar}H$), 127.2 ($C^{Ar}H$), 126.6 ($C^{Ar}H$), 126.4 ($C^{Ar}H$), 126.1 ($C^{Ar}H$), 65.1 ($C^{3}H_2$), 62.8 ($C^{11}H_2$), 57.3 ($C^{1}H_2$), 46.6 (C^{4}), 40.3 ($C^{19}H_2$), 33.5 ($C^{18}H_2$), 30.7 ($C^{28}H_3$), 21.0 ($C^{21}H_3$)

IR (neat) (cm^{-1}): 2925, 2797, 1712, 1602, 1854, 1493, 1239, 1159, 1095, 1071.

4-(2-Benzyl-1-butyl-4-methyl-1,2,3,4-tetrahydroisoquinolin-4-yl)butan-2-one (5l)

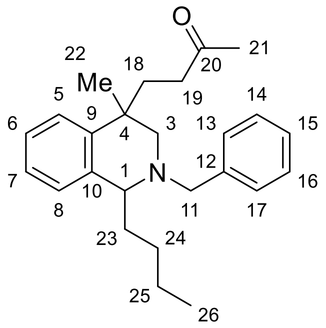

The title compound was prepared according to General Procedure **F** using isoquinolinium salt **4e** (52 mg, 0.125 mmol), methyl vinyl ketone (20 µL, 0.250 mmol), in MeCN (0.2 mL) at 55 °C and was purified by column chromatography (3% EtOAc in pentane) to furnish tetrahydroisoquinoline **5l** (25 mg, 55%) as an inseparable 4.5:1 diastereomeric mixture as a colourless oil.

The title compound could not be prepared according to General procedure **E** and only starting material was recovered from the reaction.

Data for major **both diastereomers A and B** (from the mixture):

HRMS (ESI): Exact mass calculated for $C_{25}H_{34}NO$ $[M+H]^+$: 364.2635, found: 364.2632.

^{1}H NMR (400 MHz, $CDCl_3$) δ 7.32 – 7.21 (m, 4H, C^{Ar}**H**), 7.20 – 7.03 (m, 5H, C^{Ar}**H**), 4.04 (d, *J* = 13.3 Hz, 0.2H, $C^{11}\mathbf{H_{2B}}$), 3.87 (d, *J* = 13.4 Hz, 0.8H, $C^{11}\mathbf{H_{2A}}$), 3.72 – 3.64 (m, 1H, C^{1}**H**), 3.43 (d, *J* = 13.3 Hz, 0.8H, $C^{11}\mathbf{H_{2A}}$), 3.19 (d, *J* = 13.3 Hz, 0.2H, $C^{11}\mathbf{H_{2B}}$), 2.60 (d, *J* = 11.9 Hz, 0.2H, $C^{3}\mathbf{H_{2B}}$), 2.54 (d, *J* = 11.8 Hz, 0.8H, $C^{3}\mathbf{H_{2B}}$), 2.27 – 2.06 (m, 2H, $C^{3}\mathbf{H_2}$, $C^{19}\mathbf{H_2}$), 2.00 – 1.71 (m, 7H, $C^{19}\mathbf{H_2}$, $C^{18}\mathbf{H_2}$, $C^{21}\mathbf{H_3}$, $C^{23}\mathbf{H_2}$), 1.55 (ddd, *J* = 13.9, 11.4, 4.7 Hz, 1H, $C^{18}\mathbf{H_2}$), 1.49 – 1.35 (m,1H, $C^{24}\mathbf{H_2}$), 1.25 – 1.01 (m, 6H, $C^{22}\mathbf{H_3}$, $C^{24}\mathbf{H_2}$, $C^{25}\mathbf{H_2}$,)1.03 (s, 3H, $C^{26}\mathbf{H_3}$)

^{13}C NMR (101 MHz, $CDCl_3$) δ 209.2 ($C^{20}{}_B$), 209.2 ($C^{20}{}_A$), 143.7 ($C^{Ar}{}_B$), 142.5 ($C^{Ar}{}_A$), 139.8 ($C^{Ar}{}_B$), 139.7 ($C^{Ar}{}_A$), 139.1 ($C^{Ar}{}_B$), 138.2 ($C^{Ar}{}_A$), 129.1 (2 x $C^{Ar}H_B$), 129.0 (2 x $C^{Ar}H_A$), 128.2 (2 x $C^{Ar}H_{A+B}$), 127.0 ($C^{Ar}H_B$), 126.9 ($C^{Ar}H_A$), 126.7 ($C^{Ar}H_A$), 126.5 ($C^{Ar}H_B$), 126.2 ($C^{Ar}H_A$), 125.9($C^{Ar}H_B$), 125.8 ($C^{Ar}H_B$), 125.8 ($C^{Ar}H_A$), 125.7 ($C^{Ar}H_B$), 125.3 ($C^{Ar}H_A$), 63.0 ($C^{1}H_B$), 62.8 ($C^{1}H_A$), 59.0 ($C^{11}H_{2B}$), 58.9 ($C^{11}H_{2A}$), 57.5 ($C^{3}H_{2B}$), 56.1 ($C^{3}H_{2A}$), 39.3 ($C^{19}H_{2B}$), 39.2 ($C^{19}H_{2A}$), 36.9 ($C^{4}{}_A$), 36.7 ($C^{4}{}_B$), 35.6 ($C^{18}H_{2A}$), 34.9 ($C^{18}H_{2A}$), 32.6 ($C^{23}H_{2B}$), 30.9 ($C^{23}H_{2A}$), 29.9 ($C^{21}H_{3A}$), 29.8 ($C^{21}H_{3B}$), 28.4 ($C^{22}H_{3A}$), 27.8 ($C^{24}H_{2A}$), 26.8 ($C^{24}H_{2B}$), 25.8 ($C^{22}H_{3B}$), 23.2 ($C^{25}H_{2A}$), 23.1 ($C^{25}H_{2B}$), 14.1 ($C^{26}H_{3A+B}$)

IR (neat) (cm^{-1}):2955, 2932, 1714, 1491, 1453, 1356, 1162, 910, 731, 699.

3-(*N*-Benzyl-3-methyl-1,2,3,4-tetrahydroisoquinolin-4-yl)-1-(2,3,4,5,6-pentamethylphenyl)propan-1-one (5m)

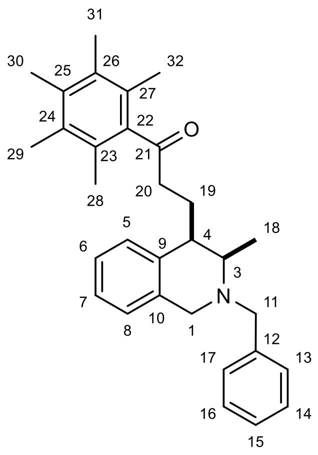

The title compound was prepared according to General Procedure **F** using isoquinolinium salt **4f** (43 mg, 0.125 mmol), 1-(2,3,4,5,6-pentamethylphenyl)prop-2-en-1-one (28 mg, 0.125 mmol), 5:2 $HCO_2H:Et_3N$ (42 µL, 0.50 mmol) in MeCN (0.1 mL) and was purified by column chromatography (3-5% EtOAc in pentane) to furnish tetrahydroisoquinoline **5m** (50 mg, 91%) as an inseparable 12:1 diastereomeric mixture as a colourless oil. The major diastereomer was found to be *cis*-**5o** (see crystal structure of the corresponding HCl salt below; NOESY inconclusive).

The title compound could not be prepared according to General procedure **E**.

HRMS (ESI): Exact mass calculated for $C_{31}H_{38}NO$ $[M+H]^+$: 440.2948, found: 440.2950.

Major diastereomer: **^{1}H NMR** (500 MHz, C_6D_6) δ 7.39 – 7.35 (m, 2H, 2 x C^{Ar}**H**), 7.31 (d, *J* = 7.7 Hz, 1H, C^{Ar}**H**), 7.24 – 7.15 (m, 2H, 2 x C^{Ar}**H**), 7.12 – 7.05 (m, 2H, 2 x C^{Ar}**H**), 7.02 – 6.96 (m, 1H, C^{Ar}**H**), 6.70 (dd, *J* = 7.7, 1.4 Hz, 1H, C^{Ar}**H**), 3.72 (d, *J* = 13.4 Hz, 1H, C^1**H₂**), 3.64 (d, *J* = 15.7 Hz, 1H, $C^{11}\mathbf{H_2}$), 3.47 (d, *J* = 15.7 Hz, 1H, $C^{11}\mathbf{H_2}$), 3.35 (d, *J* = 13.3 Hz, 1H, $C^1\mathbf{H_2}$), 3.14 – 3.05 (m, 1H, C^4**H**), 2.96 (dt, *J* = 6.6, 3.3 Hz, 1H, C^3**H**), 2.87 – 2.79 (m, 1H, $C^{20}\mathbf{H_2}$), 2.75 – 2.60 (m, 2H, $C^{20}\mathbf{H_2}$ + $C^{19}\mathbf{H_2}$), 2.07 – 2.02 (m, 1H, $C^{19}\mathbf{H_2}$) 2.01 (s, 3H, $C^{30}\mathbf{H_3}$), 1.99 (s, 6H, $C^{28}\mathbf{H_3}$ + $C^{32}\mathbf{H_3}$) 1.93 (s, 6H, $C^{29}\mathbf{H_3}$ + $C^{31}\mathbf{H_3}$), 0.88 (d, *J* = 6.6 Hz, 3H, $C^{18}\mathbf{H_3}$)

^{13}C NMR (126 MHz, C_6D_6) δ 210.4 (C^{21}), 141.9 (C^{Ar}), 140.1 (C^{Ar}), 137.9 (C^{Ar}), 135.3 (C^{Ar}), 134.8 (C^{Ar}), 132.9 (2 x C^{Ar}), 128.8 (2 x C^{Ar}H), 128.7 (2 x C^{Ar}H), 127.5 (2 x C^{Ar}), 127.4 (C^{Ar}H), 127.2 (C^{Ar}H), 126.6 (C^{Ar}H), 126.5 (C^{Ar}H), 126.0 (C^{Ar}H), 59.4 (C^1H_2), 55.6 (C^3H), 52.7 ($C^{11}H_2$), 43.4 ($C^{20}H_2$), 42.4 (C^4H), 24.2 ($C^{19}H_2$), 17.3 ($C^{29}H_3$ + $C^{31}H_3$), 16.6 ($C^{30}H_3$), 15.9 ($C^{28}H_3$ + $C^{32}H_3$), 10.2 ($C^{18}H_3$)

IR (neat) (cm^{-1}): 2964, 1698, 1452, 1377, 1315, 1262, 1116, 1027, 909, 799.

Relative stereochemistry could not be unambiguously assigned by NOESY correlations:

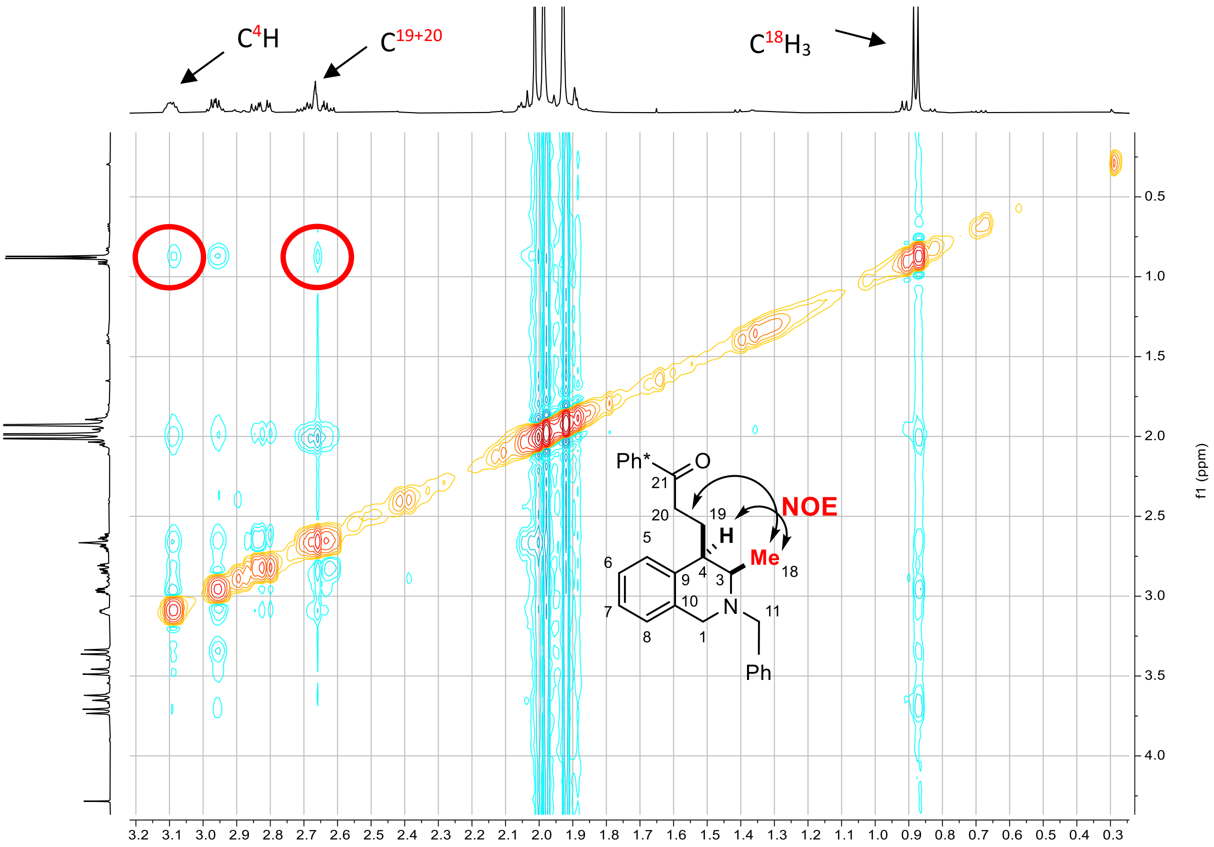

Crystal structure of the corresponding HCl salt:

***N*-Benzyl-3-methyl-4-(3-oxo-3-(2,3,4,5,6-pentamethylphenyl)propyl)-1,2,3,4-tetrahydroisoquinolinium chloride (5m·HCl)**

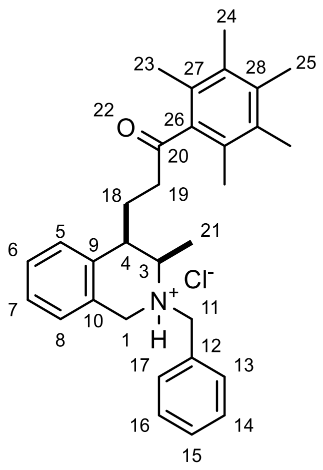

In a 25 mL round bottom flask under argon was charged isoquinoline product **5m** (140 mg, 0.32 mmol) in dry diethyl ether (5 mL). The solution was chilled to 0 °C and 2M HCl in diethyl ether (0.19 mL, 0.38 mmol) was introduced dropwise. After 15 minutes the solids were collected via filtration to give the title compound **5m·HCl** (148 mg, 98%, two diastereomers ca. 55:45) as a white solid. A single crystal for X-ray diffraction was obtained from slow evaporation from the CH_2Cl_2/hexane/MTBE solvent system.

m.p. (Et_2O): 186 – 188 °C

^{1}H NMR (400 MHz, $CDCl_3$) δ 12.90 (s, 1H), 12.81 (s, 1H), 7.89 (d, *J* = 5.4 Hz, 2H), 7.82 – 7.72 (m, 2H), 7.52 (d, *J* = 7.8 Hz, 1H), 7.48 – 7.27 (m, 9H), 7.20 (t, *J* = 8.4 Hz, 1H), 7.01 (d, *J* = 7.3 Hz, 1H), 4.73 (d, *J* = 15.8 Hz, 1H), 4.45 (d, *J* = 10.8 Hz, 2H), 4.25 (s, 1H), 4.18 – 4.05 (m, 1H), 4.00 (d, *J* = 16.3 Hz, 1H), 3.91 (s, 0H), 3.68 (s, 1H), 3.60 (s, 1H), 3.38 (s, 1H), 2.93 (ddd, *J* = 15.0, 11.0, 3.8 Hz, 1H), 2.78 – 2.67 (m, 1H), 2.67 – 2.56 (m, 3H), 2.56 – 2.40 (m, 1H), 2.23 (s, 6H), 2.18 (s, 12H), 2.07 (s, 6H), 2.02 (s, 6H), 1.88 – 1.76 (m, 2H), 1.70 (s, 2H), 1.42 (d, *J* = 6.5 Hz, 3H), 1.16 (d, *J* = 6.2 Hz, 2H).

Crystal data $C_{31}H_{38}ClNO$, M = 476.10, monoclinic, a = 20.6290(8), b = 7.2107(3), c = 17.2803(6) Å, β = 91.754°, Z = 4, T = 35 K, space group *Pc*, 24211 reflections measured, 11639 unique (R_{int} = 0.120), which were used in all calculations.

4-((4a*R*,10b*R*)-5-Benzyl-2,3,4,4a,5,6-hexahydrophenanthridin-10b(1H)-yl)butan-2-one (5n)

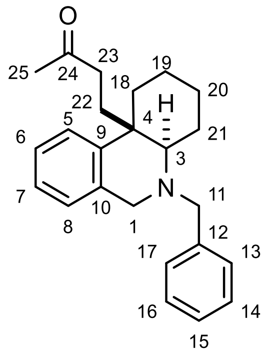

The title compound was prepared according to General Procedure **F** using isoquinolinium salt **4h** (48 mg, 0.12 mmol), methyl vinyl ketone (20 µL, 0.24 mmol), 5:2 $HCO_2H:Et_3N$ (50 µL, 0.6 mmol), $[RhCp*Cl]_2$ (7 µg, 0.005 mmol, 0.01 mol%) in MeCN (0.15 mL) and was purified by column chromatography (2.5-5% EtOAc in pentane) to furnish tetrahydroisoquinoline **5n** (28 mg, 67%, d.r. > 25:1) as a colourless oil.

HRMS (ESI): Exact mass calculated for $C_{24}H_{30}NO$ $[M+H]^+$: 348.2322, found: 348.2322.

^{1}H NMR (400 MHz, $CDCl_3$) δ 7.39 – 7.29 (m, 4H, C^{Ar}**H**), 7.27 – 7.23 (m, 1H, C^{Ar}**H**), 7.18 (dd, *J* = 7.7, 1.6 Hz, 1H, C^5**H**), 7.13 (td, *J* = 7.5, 1.5 Hz, 1H, C^6**H**), 7.08 (td, *J* = 7.3, 1.6 Hz, 1H, C^7**H**), 6.89 (dd, *J* = 7.4, 1.5 Hz, 1H, C^8**H**), 4.25 (d, *J* = 12.9 Hz, 1H, C^{11}**H$_a$**), 3.82 (d, *J* = 15.6 Hz, 1H, C^1**H$_a$**), 3.34 (d, *J* = 15.6 Hz, 1H, C^1**H$_b$**), 3.13 (d, *J* = 12.9 Hz, 1H, C^{11}**H$_b$**), 2.63 – 2.47 (m, 2H, C^{23}**H$_a$** + C^3**H**), 2.38 (ddd, *J* = 14.1, 11.9, 4.7 Hz, 1H, C^{22}**H$_a$**), 2.28 (m, 1H, C^{18}**H$_a$**), 2.18 (ddd, *J* = 16.5, 12.0, 4.4 Hz, 1H, C^{23}**H$_b$**), 2.02 (s, 3H, C^{25}**H$_3$**), 1.99 – 2.05 (m, 1H, C^{21}**H$_a$**), 1.92 (m, 2H, C^{19}**H$_a$** + C^{22}**H$_b$**), 1.58 (m, 3H, C^{20}**H$_2$** + C^{21}**H$_b$**), 1.42 – 1.27 (m, 2H, C^{19}**H$_b$** + C^{18}**H$_b$**)

^{13}C NMR (101 MHz, $CDCl_3$) δ 210.1 (C^{24}), 142.8 (C^9), 140.5 (C^{12}), 134.9 (C^{10}), 128.8 (C^{13} + C^{17}), 128.4 (C^{14} + C^{16}), 126.9 (C^{15}), 126.6 (C^8), 125.8 (C^7), 125.6 (C^6), 125.5 (C^5), 67.2 (C^3), 57.3 (C^{11}), 57.1 (C^1), 40.8 (C^4), 40.0 (C^{23}), 34.2 (C^{18}), 30.1 (C^{25}), 27.6 (C^{22}), 25.6 (C^{19}), 25.1 (C^{21}), 20.9 (C^{20})

IR (neat) (cm^{-1}): 2928, 2855, 2782, 1711, 1492, 1451, 1355, 1160, 1116, 760.

Relative stereochemistry was assigned by the following observed NOESY correlations:

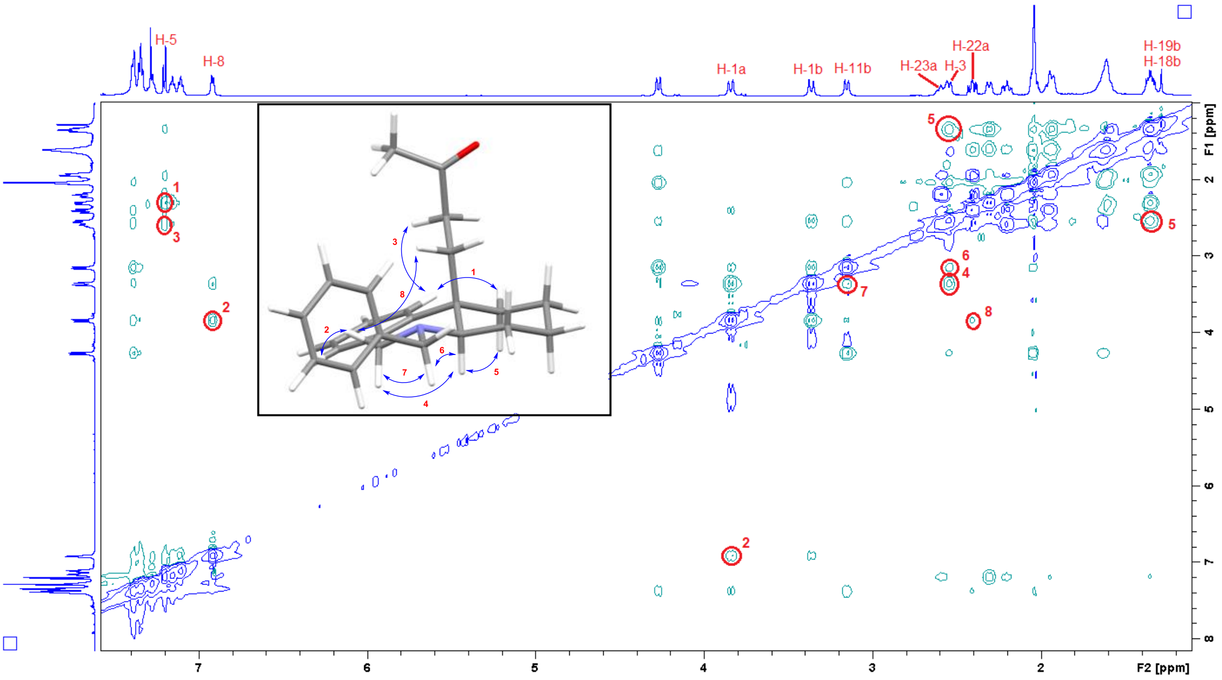

4-(*N*-Benzyl-4-methyl-3-phenyl-1,2,3,4-tetrahydroisoquinolin-4-yl)butan-2-one (5o)

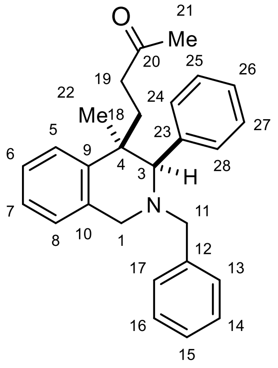

The title compound was prepared according to General Procedure **E** using isoquinolinium salt **4g** (55 mg, 0.125 mmol), methyl vinyl ketone (20 µL, 0.250 mmol), 5:2 $HCO_2H:Et_3N$ (42 µL, 0.50 mmol) in MeCN (0.1 mL) and was purified by column chromatography (10% EtOAc in pentane) to furnish tetrahydroisoquinoline **5o** (46 mg, 96%, dr > 25:1) as a colourless oil. The relative stereochemistry was assigned by NOESY. General procedure **E** gave the title compound in a significantly reduced yield of 72%.

HRMS (ESI): Exact mass calculated for $C_{27}H_{30}NO$ $[M+H]^+$: 384.2322, found: 384.2303.

Data for major diastereomer **5o** from the mixture.

^{1}H NMR (500 MHz, $CDCl_3$) δ 7.46 – 7.36 (m, 5H, 5 x C^{Ar}**H**), 7.34 – 7.26 (m, 5H, 5 x C^{Ar}**H**), 7.23 – 7.19 (m, 1H, C^{Ar}**H**), 7.06 – 7.01 (m, 3H, 3 x C^{Ar}**H**), 3.77 (d, *J* = 16.0 Hz, 1H, C^1**H_2**), 3.73 (s, 1H, C^3**H**), 3.61 (d, *J* = 16.0 Hz, 1H, C^1**H_2**), 3.54 – 3.45 (m, 2H, C^{11}**H_2**), 2.46 (ddd, *J* = 16.1, 11.9, 4.2 Hz, 1H, C^{19}**H_2**), 2.30 (ddd, *J* = 13.8, 11.7, 4.2 Hz, 1H, C^{18}**H_2**), 2.16 (ddd, *J* = 16.2, 11.8, 4.4 Hz, 1H, C^{19}**H_2**), 1.87 (s, 3H, C^{21}**H_3**), 1.71 (ddd, *J* = 13.8, 11.9, 4.5 Hz, 1H, C^{18}**H_2**), 1.53 (s, 3H, C^{22}**H_3**)

^{13}C NMR (126 MHz, $CDCl_3$) δ 209.2 (C^{20}), 143.0 (C^{Ar}), 139.3 (C^{Ar}), 135.1 (C^{Ar}), 134.4 (C^{Ar}), 130.1 (2 x C^{Ar}H), 128.8 (2 x C^{Ar}H), 128.4 (2 x C^{Ar}H), 128.0 (2 x C^{Ar}H), 127.5 (C^{Ar}H), 127.1 (C^{Ar}H), 126.8 (C^{Ar}H), 126.3 (C^{Ar}H), 125.8 (C^{Ar}H), 125.7 (C^{Ar}H), 70.7 (C^3H), 59.7 ($C^{11}H_2$), 52.4 (C^1H_2), 40.6 (C^4), 38.8 ($C^{19}H_2$), 31.4 ($C^{18}H_2$), 30.2 ($C^{21}H_3$), 29.7 ($C^{22}H_3$)

IR (neat) (cm^{-1}): 2959, 1714, 1492, 1451, 1355, 1279, 1240, 1162, 1072, 1028, 909.

Relative stereochemistry was assigned by the following observed NOESY correlations:

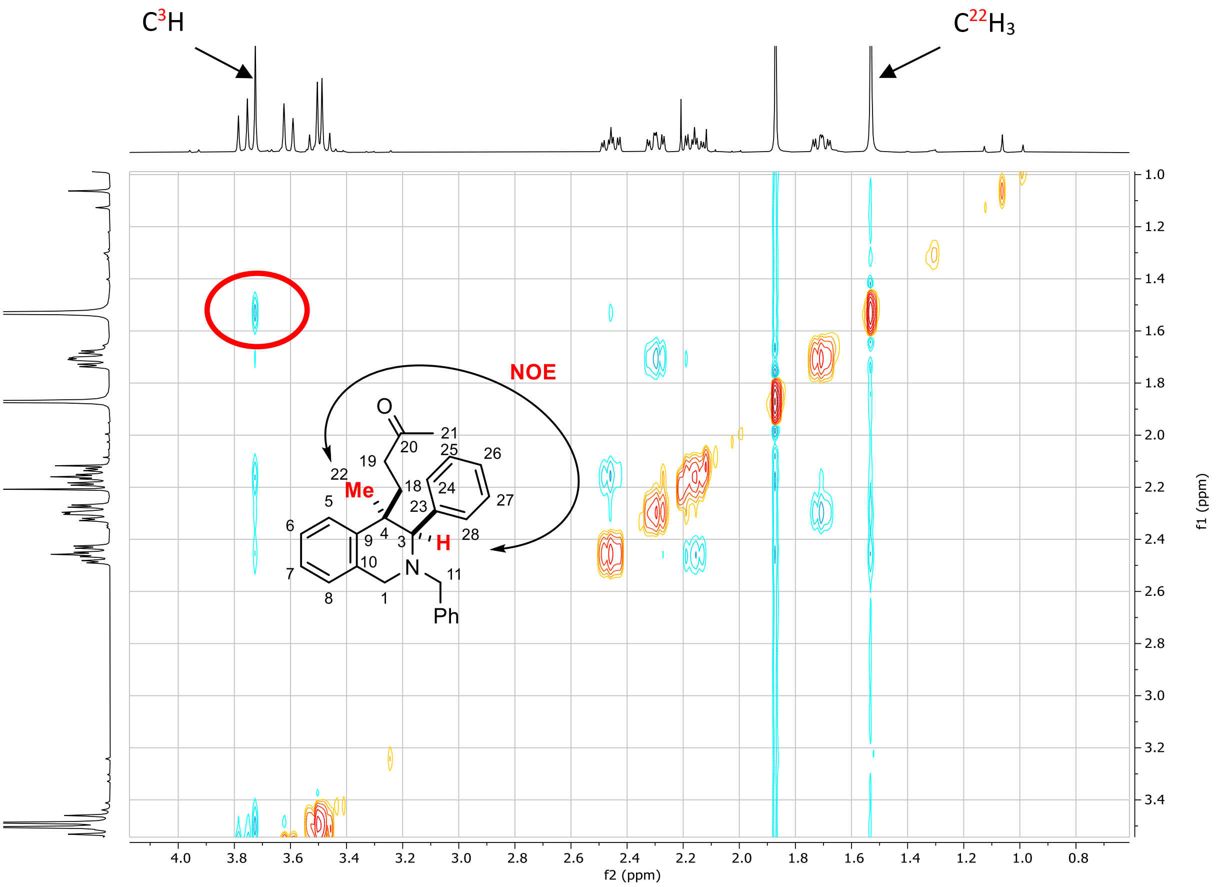

N-Methyl-4-(butan-2-one)-4-butyl-1,2,3,4-tetrahydroisoquinoline (5p)

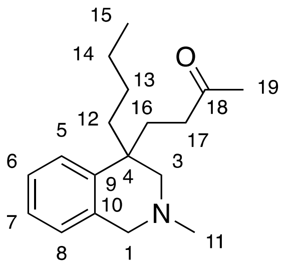

The title compound was prepared by General Procedure **E** using N-methyl-4-butylisoquinolinium iodide **4i** (41 mg, 0.125 mmol) and methyl vinyl ketone (21 µL, 0.25 mmol). Purification by column chromatography (20% EtOAc in pentane) gave *amine* **5p** (21 mg, 62%) as a yellow oil.

HRMS (ESI): Exact mass calculated for $C_{18}H_{28}ON$ $[M+H]^+$: 274.2165, found 274.2165.

^{1}H NMR (400 MHz, $CDCl_3$) δ 7.10 (s, 2H, 2 x C^{Ar}**H**), 7.09 – 7.00 (m, 1H, C^{Ar}**H**), 6.94 (dt, J = 7.5, 1.0 Hz, 1H, C^{Ar}**H**), 3.49 (d, J = 14.6 Hz, 1H, C^1**H_2**), 3.28 (d, J = 14.6 Hz, 1H, C^1**H_2**), 2.50 – 2.35 (m, 1H, C^{17}**H_2**), 2.40 (dd, J = 11.5, 1H, C^3**H_2**), 2.27 (d, J = 11.6 Hz, 1H, C^3**H_2**), 2.20 (s, 3H, C^{11}**H_3**), 2.08 – 1.90 (m, 2H, C^{16}**H_2** + C^{17}**H_2**), 1.93 (s, 3H, C^{19}**H_3**), 1.85 – 1.72 (m, 1H, C^{16}**H_2**), 1.61 (ddd, J = 13.8, 11.9, 4.4 Hz, 1H, C^{12}**H_2**), 1.67 – 1.55 (m, 1H, C^{12}**H_2**), 1.27 – 1.08 (m, 3H, C^{13}**H_2** + C^{14}**H_2**), 1.02 – 0.89 (m, 1H, C^{13}**H_2**), 0.77 (t, J = 7.1 Hz, 3H, C^{15}**H_3**)

^{13}C NMR (101 MHz, $CDCl_3$) δ 208.7 ($C^{18}O$), 140.1 (C^Q), 135.9 (C^Q), 126.6 (C^{Ar}), 126.3 (2 x C^{Ar}), 125.6 (C^{Ar}), 62.5 (C^3H_2), 59.1 (C^1H_2), 46.0 ($C^{11}H_3$), 41.7 ($C^{12}H_2$), 40.7 (C^4), 39.9 ($C^{17}H_2$), 35.9 ($C^{16}H_2$), 30.1 ($C^{19}H_3$), 26.5 ($C^{13}H_2$), 23.6 ($C^{14}H_2$), 14.2 ($C^{15}H_3$)

IR (neat) (cm^{-1}): 2931, 2860, 1714, 1492, 1464, 1450, 1253, 1161, 908, 763.

N-(4-Methoxybenzyl)-4-(butan-2-one)-4-butyl-1,2,3,4-tetrahydroisoquinoline (**5q**)

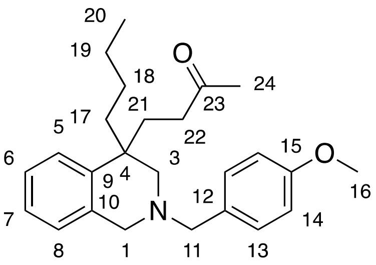

The title compound was prepared by General Procedure **E** using *N*-(4-methoxybenzyl)-4-butylisoquinolinium iodide **4j** (54 mg, 0.125 mmol) and methyl vinyl ketone (21 µL, 0.25 mmol). Purification by flash column chromatography (6% EtOAc in pentane) gave *amine* **5q** (27 mg, 57%) as a yellow oil.

HRMS (ESI): Exact mass calculated for $C_{25}H_{34}O_2N$ $[M+H]^+$: 380.2584, found 380.2584.

^{1}H NMR (400 MHz, $CDCl_3$) δ 7.26 – 7.15 (m, 2H, 2 x C^{Ar}**H**), 7.13 – 7.04 (m, 2H, 2 x C^{Ar}**H**), 7.07 – 6.96 (m, 1H, C^{Ar}**H**), 6.90 (dt, *J* = 7.6, 1.0 Hz, 1H, C^{Ar}**H**), 6.83 – 6.75 (m, 2H, 2 x C^{Ar}**H**), 3.73 (s, 3H, C^{16}**H**$_3$), 3.56 – 3.37 (m, 4H, C^1**H**$_2$ + C^{11}**H**$_2$), 2.48 – 2.32 (m, 1H, C^{22}**H**$_2$), 2.38 (d, *J* = 5.5 Hz, 2H, C^3**H**$_2$), 2.06 – 1.85 (m, 2H, C^{21}**H**$_2$ + C^{22}**H**$_2$), 1.93 (s, 3H, C^{24}**H**$_3$), 1.79 – 1.66 (m, 1H, C^{21}**H**$_2$), 1.70 – 1.59 (m, 1H, C^{17}**H**$_2$), 1.48 – 1.36 (m, 1H, C^{17}**H**$_2$), 1.12 (m, 3H, C^{18}**H**$_2$ + C^{19}**H**$_2$), 0.84 (m, 1H, C^{18}**H**$_2$), 0.74 (t, *J* = 7.1 Hz, 3H, C^{20}**H**$_3$)

^{13}C NMR (101 MHz, $CDCl_3$) δ 209.5 (C^{23}O), 158.9 (C^Q), 140.9 (C^Q), 135.8 (C^Q), 130.7 (C^Q), 130.4 (2 x C^{Ar}), 126.6 (2 x C^{Ar}), 126.5 (C^{Ar}), 126.4 (C^{Ar}), 125.6 (C^{Ar}), 113.7 (C^{Ar}), 62.5 (C$^{1/11}$H$_2$), 59.2 (C^{3}H$_2$), 57.5 (C$^{1/11}$H$_2$), 55.4 (C^{16}H$_3$), 41.4 (C^{17}H$_2$), 40.7 (C^4), 39.9 (C^{22}H$_2$), 35.0 (C^{21}H$_2$), 30.1 (C^{24}H$_3$), 26.5 (C^{18}H$_2$), 23.6 (C^{19}H$_2$), 14.2 (C^{20}H$_3$)

IR (neat) (cm^{-1}): 2931, 1714, 1612, 1512, 1302, 1246, 1171, 1036, 830, 762.

4-(N-Benzyl-1,2,3,4-tetrahydroisoquinolin-4-yl)butan-2-one (5r)

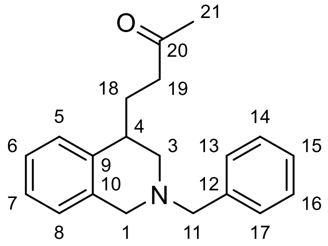

The title compound was prepared according to General Procedure **E** using isoquinolinium salt **4k** (43 mg, 0.125 mmol), methyl vinyl ketone (10 µL, 0.125 mmol), 5:2 HCO_2H:Et_3N (42 µL, 0.50 mmol) in MeCN (0.1 mL) and was purified by flash column chromatography (6-10% EtOAc in pentane) to furnish tetrahydroisoquinoline **5r** (23 mg, 63%) as a colourless oil.

HRMS (ESI): Exact mass calculated for $C_{20}H_{24}NO$ $[M+H]^+$: 294.1852, found: 294.1850.

^{1}H NMR (400 MHz, $CDCl_3$) δ 7.34 – 7.16 (m, 5H, 5 x C^{Ar}**H**), 7.13 – 7.01 (m, 3H, 3 x C^{Ar}**H**), 6.92 (d, *J* = 6.7 Hz, 1H, C^{Ar}**H**), 3.73 (d, *J* = 14.8 Hz, 1H, C^1**H**$_2$), 3.67 (d, *J* = 13.0 Hz, 1H, C^{11}**H**$_2$), 3.47 (d, *J* = 13.0 Hz, 1H, C^{11}**H**$_2$), 3.40 (d, *J* = 14.8 Hz, 1H, C^1**H**$_2$), 2.76 – 2.66 (m, 1H, C^4**H**), 2.61 (ddd, *J* = 11.6, 3.6, 1.2 Hz, 1H, C^3**H**$_2$), 2.45 (dd, *J* = 11.6, 4.3 Hz, 1H, C^3**H**$_2$), 2.31 (ddd, *J* = 16.2, 9.2, 6.6 Hz, 1H, C^{19}**H**$_2$), 2.19 (ddd, *J* = 16.8, 9.4, 5.6 Hz, 1H, C^{19}**H**$_2$), 1.99 (s, 3H, C^{21}**H**$_3$), 1.98 – 1.87 (m, 2H, 2 x C^{18}**H**$_2$)

^{13}C NMR (101 MHz, $CDCl_3$) δ 209.1 (C^{20}O), 138.8 (C^{Ar}), 138.4 (C^{Ar}), 135.2 (C^{Ar}), 129.2 (2 x C^{Ar}H), 128.5 (2 x C^{Ar}H), 128.4 (C^{Ar}H), 127.3 (C^{Ar}H), 126.6 (C^{Ar}H), 126.3 (C^{Ar}H), 126.0 (C^{Ar}H), 62.9 (C^{11}H$_2$), 56.9 (C^{1}H$_2$), 53.9 (C^{3}H$_2$), 41.4 (C^{19}H$_2$), 38.0 (C^{4}H), 30.0 (C^{21}H$_3$), 30.0 (C^{18}H$_2$)

IR (neat) (cm^{-1}): 2925, 2797, 1712, 1493, 1452, 1366, 1159, 1095, 740, 700.

4-(6-Bromo-*N*-methyl-1,2,3,4-tetrahydroisoquinolin-4-yl)butan-2-one (5s)

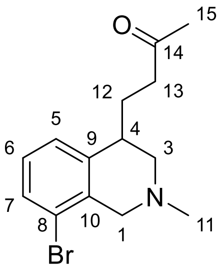

The title compound was prepared according to General Procedure **E** using isoquinolinium salt **4l** (44 mg, 0.125 mmol), methyl vinyl ketone (10 μL, 0.125 mmol), 5:2 $HCO_2H:Et_3N$ (42 μL, 0.50 mmol) in MeCN (0.1 mL) and was purified by flash column chromatography (6-10% EtOAc in pentane) to furnish tetrahydroisoquinoline **5s** (16 mg, 43%) as a colourless oil.

HRMS (ESI): Exact mass calculated for $C_{14}H_{19}BrNO$ [M+H]$^+$: 296.0645, found: 296.0645.

^{1}H NMR (400 MHz, $CDCl_3$) δ 7.38 (dd, *J* = 7.8, 1.2 Hz, 1H, C^{7}**H**), 7.19 – 7.14 (m, 1H, C^{5}**H**), 7.09 – 7.01 (m, 1H, C^{6}**H**), 3.74 (d, *J* = 16.0 Hz, 1H, C^{1}**H$_2$**), 3.27 (d, *J* = 16.0 Hz, 1H, C^{1}**H$_2$**), 2.86 (dt, *J* = 8.5, 4.2 Hz, 1H, C^{4}**H**), 2.61 – 2.54 (m, 1H, C^{3}**H$_2$**), 2.54 – 2.48 (m, 2H, C^{3}**H$_2$** + C^{13}**H$_2$**), 2.45 – 2.36 (m, 4H, C^{11}**H$_3$** + C^{13}**H$_2$**), 2.13 (s, 3H, C^{15}**H$_3$**), 2.11 – 2.05 (m, 1H, C^{12}**H$_2$**), 1.95 (dtd, *J* = 14.1, 8.2, 5.9 Hz, 1H, C^{12}**H$_2$**);

^{13}C NMR (101 MHz, $CDCl_3$) δ 208.5 (C^{14}O), 140.5 (C$^{9/10}$), 134.6 (C$^{9/10}$), 130.1 (C^{7}H), 127.6 (C$^{5/6}$H), 127.6 (C$^{5/6}$H), 122.7 (C^{8}), 58.9 (C^{1}H$_2$), 56.4 (C^{3}H$_2$), 46.1 (C^{11}H$_3$), 41.0 (C^{13}H$_2$), 38.2 (C^{4}H), 30.1 (C^{15}H$_3$), 29.8 (C^{12}H$_2$)

IR (neat) (cm^{-1}): 2938, 2780, 1713, 1564, 1461, 1445, 1377, 1367, 1356, 1161, 1113, 776, 715.

3-(*N*-Benzyl-1,2,3,4-tetrahydroisoquinolin-4-yl)pyrrolidine-2,5-dione (5t)

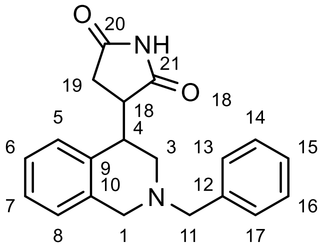

The title compound was prepared according to General Procedure **E** using isoquinolinium salt **4k** (43 mg, 0.125 mmol), methyl vinyl ketone (12 mg, 0.125 mmol), 5:2 $HCO_2H:Et_3N$ (42 μL, 0.50 mmol) in MeCN (0.1 mL) and was purified by flash column chromatography (6-10% EtOAc in pentane) to furnish tetrahydroisoquinoline **5t** (26 mg, 65%) as an inseparable 2:1 diastereomeric mixture as a colourless oil.

Data for **both diastereomers A and B** (from the mixture):

HRMS (ESI): Exact mass calculated for $C_{20}H_{21}N_2O_2$ [M+H]$^+$: 321.1598, found: 321.1593.

^{1}H NMR (400 MHz, $CDCl_3$) δ 8.20 (s, 0.48H, NH$_A$), 8.09 – 7.91 (m, 0.45H, NH$_B$), 7.34 – 7.17 (m, 5H, C^{Ar}**H**$_{A/B}$), 7.15 – 6.92 (m, 4H, C^{Ar}**H**$_{A/B}$), 3.88 – 3.76 (m, 1H, C^{1}**H$_{2A+B}$**), 3.69 – 3.52 (m, 2H, C^{18}**H**$_{A/B}$ + C^{11}**H$_{2A+B}$** + C^{19}**H$_{2A/B}$**), 3.52 – 3.44 (m, 1H, C^{18}**H**$_{A/B}$), 3.37 – 3.22 (m, 2H, C^{1}**H$_{2A+B}$** + C^{4}**H**$_{A+B}$ + C^{11}**H$_{2A+B}$**), 2.98 (ddd, *J* = 8.7, 5.8, 2.4 Hz, 0.52H, C^{4}**H**$_{A/B}$), 2.89 – 2.77 (m, 1H, C^{1}**H$_{2A+B}$** + C^{3}**H$_{2A+B}$**), 2.71 (dd, *J* = 19.0, 9.2 Hz, 0.57H,

C^{19}**H**$_{A/B}$), 2.62 (dd, *J* = 11.6, 3.9 Hz, 0.52H, C^{1}**H**$_{\mathbf{2A/B}}$), 2.58 – 2.43 (m, 1H, C^{3}**H**$_{\mathbf{2A+B}}$), 2.23 (dd, *J* = 18.7, 9.5 Hz, 0.49H, C^{19}**H**$_{\mathbf{2A/B}}$)

^{13}C NMR (101 MHz, $CDCl_3$) δ 180.4 (C$^{21}_{A}$), 179.4 (C$^{21}_{B}$), 177.7 (C$^{20}_{A}$), 177.5 (C$^{20}_{B}$), 138.2(C$^{Ar}_{B}$), 137.6 (C$^{Ar}_{A}$), 136.1 (C$^{Ar}_{B}$), 135.7 (C$^{Ar}_{A}$), 134.5 (C$^{Ar}_{B}$), 132.7 (C$^{Ar}_{A}$), 129.6 (2 x C^{Ar}H$_{B}$), 129.1(2 x C^{Ar}H$_{A}$), 128.6 (2 x C^{Ar}H$_{B}$), 128.6 (2 x C^{Ar}H$_{A}$), 128.2 (C^{Ar}H$_{A}$), 127.9 (C^{Ar}H$_{B}$), 127.5 (C^{Ar}H$_{B}$), 127.5 (C^{Ar}H$_{A}$), 127.4 (C^{Ar}H$_{B}$), 127.0 (4 x C^{Ar}H$_{A/B}$), 126.8 (C^{Ar}H$_{A}$), 63.2 (C^{11}H$_{2B}$), 63.1 (C^{11}H$_{2A}$), 56.9 (C^{1}H$_{2B}$), 56.5 (C^{1}H$_{2A}$ + C^{3}H$_{2A/B}$), 52.3 (C^{3}H$_{2A+B}$), 49.0 (C^{4}H$_{A}$), 48.4 (C^{4}H$_{B}$), 38.0 (C^{18}H$_{A}$), 36.5 (C^{18}H$_{A}$), 33.8 (C^{19}H$_{2A}$), 33.2(C^{19}H$_{2A}$)

IR (neat) (cm^{-1}): 3204, 3064, 2804, 2762, 1700, 1348, 1268, 1180, 1092, 910.

_N_-Benzyl-4-(2-nitro-1-phenylethyl)-1,2,3,4-tetrahydroisoquinoline (5u)

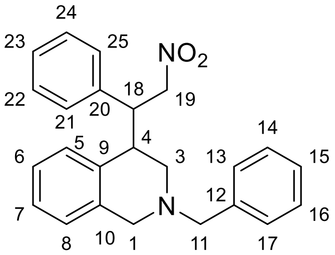

The title compound was prepared according to General Procedure **E** using isoquinolinium salt **4k** (43 mg, 0.125 mmol), (*E*)-(2-nitrovinyl)benzene (19 mg, 0.125 mmol), 5:2 HCO_2H:Et_3N (42 μL, 0.50 mmol) in MeCN (0.1 mL) and was purified by column chromatography (4% EtOAc in pentane) to furnish the diastereomeric tetrahydroisoquinolines **5u*A*** (19 mg, 41%) and **5u*B*** (9 mg, 19%) as colourless oils.

Data for **major diastereomer 5u*A*:**

HRMS (ESI): Exact mass calculated for $C_{24}H_{25}N_2O_2$ [M+H]$^+$: 373.1911, found: 373.1901.

^{1}H NMR (400 MHz, $CDCl_3$) δ 7.34 – 7.24 (m, 5H, 5 x C^{Ar}**H**), 7.21 – 7.04 (m, 7H, 7 x C^{Ar}**H**), 6.98 (dd, *J* = 7.6, 1.5 Hz, 1H, C^{Ar}**H**), 6.90 (dd, *J* = 7.6, 1.5 Hz, 1H, C^{Ar}**H**), 4.64 – 4.59 (m, 2H, C^{19}**H**$_{\mathbf{2}}$), 4.03 (td, *J* = 9.5, 6.8 Hz, 1H, C^{18}**H**), 3.94 (d, *J* = 15.4 Hz, 1H, C^{1}**H**$_{\mathbf{2}}$), 3.47 (s, 2H, C^{11}**H**$_{\mathbf{2}}$), 3.26 (d, *J* = 15.5 Hz, 1H, C^{1}**H**$_{\mathbf{2}}$), 2.93 (dt, *J* = 9.3, 2.5 Hz, 1H, C^{4}**H**), 2.67 (dt, *J* = 11.5, 1.6 Hz, 1H, C^{3}**H**$_{\mathbf{2}}$), 2.32 (dd, *J* = 11.6, 3.2 Hz, 1H, C^{3}**H**$_{\mathbf{2}}$)

^{13}C NMR (101 MHz, $CDCl_3$) δ 139.4 (C^{Ar}), 138.1 (C^{Ar}), 135.8 (C^{Ar}), 134.2 (C^{Ar}), 129.7 (C^{Ar}H), 129.3 (2 x C^{Ar}H), 128.8 (2 x C^{Ar}H), 128.5 (2 x C^{Ar}H), 128.1 (2 x C^{Ar}H), 127.4 (C^{Ar}H), 127.3 (C^{Ar}H), 127.3 (C^{Ar}H), 127.1 (C^{Ar}H), 125.7 (C^{Ar}H), 79.9 (C^{19}H), 63.0 (C^{11}H$_2$), 55.9 (C^{1}H$_2$), 53.4 (C^{3}H$_2$), 48.3 (C^{18}H), 43.8 (C^{4}H)

IR (neat) (cm^{-1}): 3029, 2921, 2805, 1551, 1495, 1454, 1378, 1089, 911, 757, 735, 701.

Data for **minor diastereomer 5u*B*:**

HRMS (ESI): Exact mass calculated for $C_{24}H_{25}N_2O_2$ [M+H]$^+$: 373.1911, found: 373.1904.

^{1}H NMR (400 MHz, $CDCl_3$) δ 7.51 – 7.40 (m, 5H, 5 x C^{Ar}**H**), 7.37 – 7.30 (m, 1H, C^{Ar}**H**), 7.26 – 7.06 (m, 6H, 6 x C^{Ar}**H**), 6.81 – 6.74 (m, 2H, 2 x C^{Ar}**H**), 5.19 (dd, *J* = 14.1, 12.2 Hz, 1H, C^{19}**H**$_{\mathbf{2}}$), 4.18 (dd, *J* = 14.1, 3.8 Hz, 1H, C^{19}**H**$_{\mathbf{2}}$), 4.08 – 3.96 (m, 2H, C^{18}**H** + C^{1}**H**$_{\mathbf{2}}$), 3.83 (d, *J* = 12.2 Hz, 1H, C^{11}**H**$_{\mathbf{2}}$), 3.41 (d, *J* = 14.9 Hz, 1H, C^{1}**H**$_{\mathbf{2}}$), 3.31 (d, *J* = 12.3 Hz, 1H, C^{11}**H**$_{\mathbf{2}}$), 3.01 (t, *J* = 4.6 Hz, 1H, C^{4}**H**), 2.91 (d, *J* = 12.3 Hz, 1H, C^{3}**H**$_{\mathbf{2}}$), 2.07 (dd, *J* = 12.4, 4.3 Hz, 1H, C^{3}**H**$_{\mathbf{2}}$)

^{13}C NMR (101 MHz, $CDCl_3$) δ 138.9 (C^{Ar}), 138.0 (C^{Ar}), 136.5 (C^{Ar}), 135.1 (C^{Ar}), 130.3 (2 x $C^{Ar}H$), 128.9 (2 x $C^{Ar}H$), 128.8 (2 x $C^{Ar}H$), 128.6 ($C^{Ar}H$), 127.8 (2 x $C^{Ar}H$), 127.8 ($C^{Ar}H$), 127.2 ($C^{Ar}H$), 127.1 ($C^{Ar}H$), 127.0 ($C^{Ar}H$), 126.7 ($C^{Ar}H$), 75.4 ($C^{19}H_2$), 63.1 ($C^{11}H_2$), 57.4 ($C^{1}H_2$), 50.4 ($C^{18}H$), 49.2 ($C^{3}H_2$), 43.8 ($C^{4}H$)

IR (neat) (cm^{-1}): 3029, 2804, 1547,1495, 1453, 1380, 1088, 756, 738, 700.

3-(*N*-Benzyl-1,2,3,4-tetrahydroisoquinolin-4-yl)cyclohexan-1-one (5w)

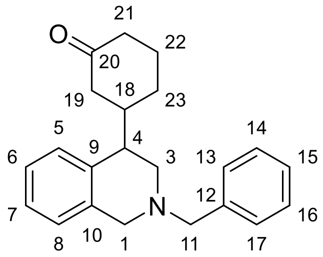

The title compound was prepared according to General Procedure **E** using isoquinolinium salt **4k** (43 mg, 0.125 mmol), cyclohex-2-en-1-one (12 mg, 0.125 mmol), 5:2 HCO_2H:Et_3N (42 μL, 0.50 mmol) in MeCN (0.1 mL) and was purified by flash column chromatography (6-10% EtOAc in pentane) to furnish tetrahydroisoquinoline **5w** (12 mg, 30%) as an inseparable 1:1 diastereomeric mixture as a colourless oil.

Data for **both diastereomers A and B** (from the mixture):

HRMS (ESI): Exact mass calculated for $C_{22}H_{26}NO$ $[M+H]^+$: 320.2009, found: 320.2009.

^{1}H NMR (400 MHz, $CDCl_3$) δ 7.42 – 7.24 (m, 5H, 5 x $C^{Ar}\mathbf{H}_{\mathbf{A+B}}$), 7.18 – 7.07 (m, 3H, 3 x $C^{Ar}\mathbf{H}_{\mathbf{A+B}}$), 7.04 – 6.96 (m, 1H, $C^{Ar}\mathbf{H}_{\mathbf{A+B}}$), 7.04 – 6.96 (m, 1H, $C^{1}\mathbf{H}_{\mathbf{2}A+B}$), 3.76 – 3.66 (m, 1H, $C^{11}\mathbf{H}_{\mathbf{2}A+B}$), 3.59 – 3.51 (m, 1H, $C^{11}\mathbf{H}_{\mathbf{2}A+B}$), 3.47 – 3.34 (m, 1H, $C^{1}\mathbf{H}_{\mathbf{2}A+B}$), 3.47 – 3.34 (m, 1H, $C^{3}\mathbf{H}_{\mathbf{2}A+B}$), 2.79 (q, *J* = 4.2 Hz, 0.5H, $C^{4}\mathbf{H}_{A/B}$), 2.66 (q, *J* = 4.0 Hz, 0.5H, $C^{4}\mathbf{H}_{A/B}$), 2.54 – 2.44 (m, 1H, $C^{3}\mathbf{H}_{\mathbf{2}A+B}$), 2.42 – 1.93 (m, 6H, $C^{18}\mathbf{H}_{A+B}$ + $C^{19}\mathbf{H}_{\mathbf{2}A+B}$ + $C^{21}\mathbf{H}_{\mathbf{2}A+B}$ + $C^{22}\mathbf{H}_{\mathbf{2}A/B}$), 1.87 – 1.79 (m, 0.5H, $C^{23}\mathbf{H}_{A/B}$), 1.65 – 1.42 (m, 2H, $C^{23}\mathbf{H}_{\mathbf{2}A/B}$ + $C^{22}\mathbf{H}_{\mathbf{2}A/B}$), 1.41 – 1.29 (m, 0.5H, $C^{23}\mathbf{H}_{\mathbf{2}A/B}$)

^{13}C NMR (151 MHz, $CDCl_3$) δ 212.6 ($C^{20}{}_{A/B}$), 212.3 ($C^{20}{}_{A/B}$), 138.6 ($C^{Ar}{}_{A/B}$), 138.4 ($C^{Ar}{}_{A/B}$), 136.2 ($C^{Ar}{}_{A/B}$), 136.2 ($C^{Ar}{}_{A/B}$), 135.7 ($C^{Ar}{}_{A/B}$), 135.7 ($C^{Ar}{}_{A/B}$), 129.4 (2 x $C^{Ar}H_{A/B}$), 129.3 (2 x $C^{Ar}H_{A/B}$), 128.9 ($C^{Ar}H_{A/B}$), 128.6 ($C^{Ar}H_{A/B}$), 128.5 (2 x $C^{Ar}H_{A/B}$), 128.4 (2 x $C^{Ar}H_{A/B}$), 127.4 ($C^{Ar}H_{A/B}$), 127.4 ($C^{Ar}H_{A/B}$), 126.7 ($C^{Ar}H_{A/B}$), 126.6 ($C^{Ar}H_{A/B}$), 126.2 ($C^{Ar}H_{A/B}$), 126.2 (2 x $C^{Ar}H_{A/B}$), 126.0 ($C^{Ar}H_{A/B}$), 63.2 ($C^{11}H_{2A/B}$), 63.0 ($C^{11}H_{2A/B}$), 56.7 ($C^{1}H_{2A/B}$), 56.6 ($C^{1}H_{2A/B}$), 52.0 ($C^{3}H_{2A/B}$), 51.7 ($C^{3}H_{2A/B}$), 47.2 ($C^{19}H_{2A/B}$), 45.3 ($C^{19}H_{2A/B}$), 44.1 ($C^{4}H_{A/B}$), 44.0 ($C^{4}H_{A/B}$), 43.4 ($C^{18}H_{A/B}$), 42.9 ($C^{18}H_{A/B}$), 41.6 ($C^{21}H_{A/B}$), 41.6 ($C^{21}H_{A/B}$), 30.1 ($C^{23}H_{2A/B}$), 28.7 ($C^{23}H_{2A/B}$), 25.8 ($C^{22}H_{2A/B}$), 25.6 ($C^{22}H_{2A/B}$)

IR (neat) (cm^{-1}): 3027, 2926, 2864, 2799, 2757, 1708, 1494, 1345, 1312, 1093.

4,4'-(2-Benzyl-1,2,3,4-tetrahydroisoquinoline-4,4-diyl)bis(butan-2-one) (5x)

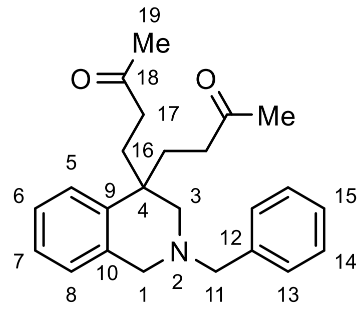

The title compound was prepared according to modified General Procedure **F** using isoquinolinium salt **4k** (43 mg, 0.125 mmol), methyl vinyl ketone (50 µL, 0.61 mmol), 5:2 HCO_2H:Et_3N (42 µL, 0.50 mmol) in MeCN (0.1 mL) and was purified by flash column chromatography (2% MeCN in 1:1 pentane:CH_2Cl_2) to furnish tetrahydroisoquinoline **5x** (20 mg, 44%) as a colourless oil.

General procedure **E** gave the title compound in comparable yield of 45%, however this procedure resulted in formation of an impurity that made purification significantly more challenging and a clean sample of 5x could not be obtained without significant losses in yield.

HRMS (ESI): Exact mass calculated for $C_{24}H_{30}NO_2$ $[M+H]^+$: 364.2271, found: 364.2274.

^{1}H NMR (400 MHz, $CDCl_3$) δ 7.39 – 7.24 (m, 5H, 5 x C^{Ar}**H**), 7.24 – 7.08 (m, 3H, 3 x C^{Ar}**H**), 7.00 (dd, J = 7.5, 1.4 Hz, 1H, C^{Ar}**H**), 3.60 – 3.53 (m, 4H, C^1**H_2** + C^{11}**H_2**), 2.51 – 2.37 (m, 4H, C^3**H_2** + C^{17}**H_2**), 2.12 – 1.96 (m, 10H, C^{16}**H_2** + C^{17}**H_2** + 2 x C^9**H_3**), 1.84 – 1.74 (m, 2H, C^{16}**H_2**)

^{13}C NMR (101 MHz, $CDCl_3$) δ 208.9 (C^{18}), 139.5 (C^{Ar}), 138.3 (C^{Ar}), 135.9 (C^{Ar}), 129.4 (2 x C^{13}H/C^{14}H), 128.4 (2 x C^{13}H/C^{14}H), 127.4 (C^{Ar}H), 126.9 (C^{Ar}H), 126.7 (C^{Ar}H), 126.3 (C^{Ar}H), 126.0 (C^{Ar}H), 63.1 ($C^{11}H_2$), 59.4 (C^1H_2), 57.4 (C^3H_2), 40.2 ($C^{17}H_2$), 39.5 (C^4H), 35.0 ($C^{19}H_3$), 30.1 ($C^{16}H_2$)

IR (neat) (cm^{-1}): 2925, 2797, 1712, 1493, 1452, 1366, 1159, 1095, 740, 700.

4-(9,10-Dimethoxy-5,8,13,13a-tetrahydro-6H-[1,3]dioxolo[4,5-g]isoquinolino[3,2-a]isoquinolin-13-yl)butan-2-one (5y)

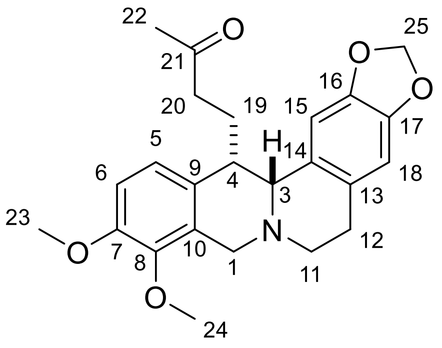

The title compound was prepared according to General Procedure **E** using berberine chloride (47 mg, 0.125 mmol), methyl vinyl ketone (10 µL, 0.125 mmol), 5:2 HCO_2H:Et_3N (42 µL, 0.50 mmol) in MeCN (0.3 mL) and was purified by flash column chromatography (0.5% MeOH in CH_2Cl_2) to furnish tetrahydroisoquinoline **5y** (44 mg, 86%, d.r. > 25:1) as a colourless oil.

HRMS (ESI): Exact mass calculated for $C_{24}H_{28}NO_5$ $[M+H]^+$: 410.1962, found: 410.1963.

^{1}H NMR (400 MHz, $CDCl_3$) δ 6.84 (d, *J* = 8.4 Hz, 1H, $C^{5/6}$**H**), 6.79 (d, *J* = 8.4 Hz, 1H, $C^{5/6}$**H**), 6.67 (s, 1H, $C^{15/18}$**H**), 6.57 (s, 1H, $C^{15/18}$**H**), 5.92 (d, *J* = 1.4 Hz, 1H, C^{25}**H_2**), 5.90 (d, *J* = 1.4 Hz, 1H, C^{25}**H_2**), 4.21 (d, *J* = 16.1 Hz, 1H, C^1**H_2**), 3.86 (s, 3H, $C^{23/24}$**H_3**), 3.86 (s, 3H, $C^{23/24}$**H_3**), 3.68 (d, *J* = 2.9 Hz, 1H, C^3**H**), 3.48 (d, *J* = 16.1 Hz, 1H, C^1**H_2**), 3.16 – 2.99 (m, 3H, C^4**H** + C^{11}**H_2**, C^{12}**H_2**), 2.63 – 2.49 (m, 2H, C^{11}**H_2**, C^{12}**H_2**), 2.49 – 2.34 (m, 1H, C^{20}**H_2**), 2.26 – 2.13 (m, 1H, C^{20}**H_2**), 1.95 (s, 3H, C^{22}**H_2**), 1.76 – 1.60 (m, 2H, C^{19}**H_2**)

^{13}C NMR (101 MHz, $CDCl_3$) δ 209.7 (C^{21}), 149.6 (C^{Ar}), 146.4 (C^{Ar}), 145.8 (C^{Ar}), 145.1 (C^{Ar}), 132.2 (C^{Ar}), 129.4 (C^{Ar}), 129.2 (C^{Ar}), 128.9 (C^{Ar}), 124.5 ($C^{5/6}H$), 110.3 ($C^{5/6}H$), 108.4 ($C^{15/18}H$), 105.6 ($C^{15/18}H$), 100.8 ($C^{25}H_2$), 63.4 ($C^{3}H$), 60.1 ($C^{23/24}H_3$), 55.8 ($C^{23/24}H_3$), 54.2 ($C^{1}H_2$), 50.5 ($C^{11}H_2$), 43.3 ($C^{4}H$), 41.7 ($C^{20}H_2$), 29.8 ($C^{12}H_2$), 29.7 ($C^{22}H_3$), 26.1 ($C^{19}H_2$)

IR (neat) (cm^{-1}): 2905, 1709, 1484, 1278, 1245, 1221, 1163, 1087, 1070, 1037, 859, 808, 730.

Relative stereochemistry was assigned based on the J_{H3-H4} coupling constant (2.9 Hz observed, calculated[10] for *cis*: 3.6 Hz)

4-(2,3,9,10-Tetramethoxy-5,8,13,13a-tetrahydro-6H-isoquinolino[3,2-a]isoquinolin-13-yl)butan-2-one (5z)

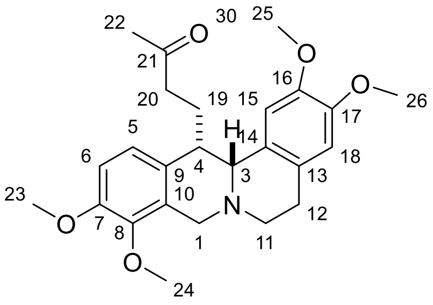

The title compound was prepared according to General Procedure **E** using palmatine chloride (48 mg, 0.125 mmol), methyl vinyl ketone (10 µL, 0.125 mmol), 5:2 $HCO_2H:Et_3N$ (42 µL, 0.50 mmol) in MeCN (0.2 mL) and was purified by column chromatography (0.4–0.5% MeOH in CH_2Cl_2) to furnish tetrahydroisoquinoline **5y** (40 mg, 75%, d.r. > 25:1) as a colourless oil.

HRMS (ESI): Exact mass calculated for $C_{25}H_{32}NO_5$ $[M+H]^+$: 426.2280, found: 426.2279.

^{1}H NMR (400 MHz, $CDCl_3$) δ 6.85 (d, J = 8.4 Hz, 1H, $C^{5/6}\mathbf{H}$), 6.79 (d, J = 8.4 Hz, 1H, $C^{5/6}\mathbf{H}$), 6.70 (s, 1H, $C^{15/18}\mathbf{H}$), 6.60 (s, 1H, $C^{15/18}\mathbf{H}$), 4.21 (d, J = 16.1 Hz, 1H, $C^{1}\mathbf{H_2}$), 3.89 (s, 3H, $C^{23/24/25/26}\mathbf{H_3}$), 3.87 (s, 3H, $C^{23/24/25/26}\mathbf{H_3}$), 3.86 (s, 3H, $C^{23/24/25/26}\mathbf{H_3}$), 3.86 (s, 4H, $C^{23/24/25/26}\mathbf{H_3}$), 3.74 – 3.66 (d, J = 3.0 Hz, 1H, $C^{3}\mathbf{H}$), 3.49 (d, J = 16.1 Hz, 1H, $C^{1}\mathbf{H_2}$), 3.20 – 3.04 (m, 3H, $C^{4}\mathbf{H}$ + $C^{11}\mathbf{H_2}$, $C^{12}\mathbf{H_2}$), 2.64 – 2.52 (m, 2H, $C^{11}\mathbf{H_2}$, $C^{12}\mathbf{H_2}$), 2.42 – 2.31 (m, 1H, $C^{20}\mathbf{H_2}$), 2.28 – 2.16 (m, 1H, $C^{20}\mathbf{H_2}$), 1.93 (s, 3H, $C^{22}\mathbf{H_3}$), 1.72 – 1.63 (m, 2H, $C^{19}\mathbf{H_2}$)

^{13}C NMR (101 MHz, $CDCl_3$) δ 209.9 (C^{21}), 150.6 (C^{Ar}), 147.7 (C^{Ar}), 147.4 (C^{Ar}), 145.3 (C^{Ar}), 132.4 (C^{Ar}), 129.0 (C^{Ar}), 128.4 (C^{Ar}), 128.2 (C^{Ar}), 125.1 ($C^{5/6}H$), 111.4 ($C^{15/18}H$), 110.4 ($C^{5/6}H$), 108.8 ($C^{15/18}H$), 63.1 ($C^{3}H$), 60.2 ($C^{23/24/25/26}H_3$), 56.1 ($C^{23/24/25/26}H_3$), 55.9 ($C^{23/24/25/26}H_3$), 55.9 ($C^{23/24/25/26}H_3$), 54.4 ($C^{1}H_2$), 51.5 ($C^{11}H_2$), 42.0 ($C^{4}H$), 41.6 ($C^{20}H_2$), 29.8 ($C^{20}H_3$), 29.4 ($C^{12}H_2$), 26.4 ($C^{19}H_2$)

IR (neat) (cm^{-1}): 2937, 2834, 1710, 1610, 1517, 1456, 1279, 1231, 1144, 1110, 913.

Relative stereochemistry was assigned based on the J_{H3-H4} coupling constant (3.0 Hz observed, calculated[11] for *cis*: 3.6 Hz)

[10] https://www.nmrdb.org/ (accessed 07.10.2021)

[11] https://www.nmrdb.org/ (accessed 07.10.2021)

4-(2-Methyl-5-((4-tosyl-1,4-diazepan-1-yl)sulfonyl)-1,2,3,4-tetrahydroisoquinolin-4-yl)butan-2-one (5aa)

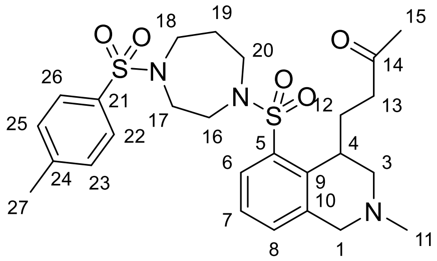

The title compound was prepared according to General Procedure **E** using isoquinolinium salt **4m** (83 mg, 0.141 mmol), methyl vinyl ketone (12 µL, 0.141 mmol), 5:2 $HCO_2H:Et_3N$ (42 µL, 0.50 mmol) in MeCN (0.3 mL) and was purified by flash column chromatography (1% MeOH in CH_2Cl_2) to furnish tetrahydroisoquinoline **5aa** (71 mg, 95%) as a colourless oil.

HRMS (ESI): Exact mass calculated for $C_{26}H_{36}N_3O_5S_2$ $[M+H]^+$: 534.2091, found: 534.2090.

^{1}H NMR (400 MHz, $CDCl_3$) δ 7.59 (d, *J* = 8.3 Hz, 2H, 2 x C^{Ar}**H**), 7.37 (dd, *J* = 7.5, 1.7 Hz, 1H, C^{Ar}**H**), 7.24 (d, *J* = 7.9 Hz, 2H, 2 x C^{Ar}**H**), 7.20 – 7.10 (m, 2H, 2 x C^{Ar}**H**), 3.92 (d, *J* = 15.6 Hz, 1H, C^1**H₂**), 3.51 – 3.28 (m, 9H, C^{16}**H₂** + C^{17}**H₂** + C^{18}**H₂** + C^{20}**H₂** + C^4**H**), 3.19 (d, *J* = 15.6 Hz, 1H, C^1**H₂**), 2.79 (dt, *J* = 11.7, 1.9 Hz, 1H, C^3**H₂**), 2.55 – 2.44 (m, 1H, C^{13}**H₂**), 2.45 – 2.35 (m, 1H, C^{13}**H₂**), 2.35 (s, 3H, C^{27}**H₃**), 2.30 (s, 3H, C^{11}**H₃**), 2.25 (dd, *J* = 11.4, 3.2 Hz, 1H, C^3**H₂**), 2.13 – 1.99 (m, 5H, C^{15}**H₃** + C^{12}**H₂**), 1.94 (td, *J* = 6.3, 2.1 Hz, 2H, C^{19}**H₂**)

^{13}C NMR (101 MHz, $CDCl_3$) δ 210.1 (C^{14}), 143.6 (C^{Ar}), 138.6 (C^{Ar}), 137.7 (C^{Ar}), 137.6 (C^{Ar}), 136.2 (C^{Ar}), 131.3 ($C^{Ar}H$), 129.9 (2 x $C^{Ar}H$), 126.9 (2 x $C^{Ar}H$), 126.2 ($C^{Ar}H$), 126.1 ($C^{Ar}H$), 57.6 (C^1H_2), 54.3 (C^3H_2), 51.8 ($C^{16/17/18/20}H_2$), 51.6 ($C^{16/17/18/20}H_2$), 47.7 ($C^{16/17/18/20}H_2$), 47.7 ($C^{16/17/18/20}H_2$), 46.0 ($C^{11}H_3$), 43.0 ($C^{13}H_2$), 36.3 (C^4H), 30.2 ($C^{12}H_2$), 29.7 ($C^{15}H_3$), 29.6 ($C^{19}H_2$), 21.6 ($C^{27}H_3$)

IR (neat) (cm^{-1}): 2947, 2784, 1709, 1321, 1290, 1228, 1090, 1057, 983, 906.

Derivatisations of tetrahydroisoquinolines

3-(4-Methyl-1,2,3,4-tetrahydroisoquinolin-4-yl)-1-(2,3,4,5,6-pentamethylphenyl)propan-1-one (6a)

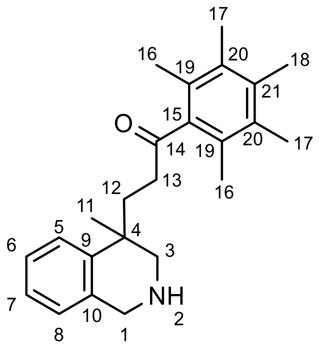

In a microwave vial tetrahydroisoquinoline **5d** (60 mg, 0.13 mmol) was charged and dissolved in MeOH (5 mL). To the solution was added $Pd(OH)_2/C$ (20% wt, 7.7 mg, 11 µmol, 8 mol%) and ammonium formate (86.0 mg, 1.36 mmol). The reaction mixture was then heated to 65 °C and stirred for 16 h. After cooling to room temperature, the reaction was poured onto 1M NaOH and extracted with CH_2Cl_2 (3 × 10 mL). After drying over $MgSO_4$ and evaporating the solvent the crude product was purified by silica gel chromatography (2% MeOH in CH_2Cl_2) to give amine **6a** (37 mg, 78%) as a colourless oil.

HRMS (ESI): Exact mass calculated for $C_{24}H_{32}NO$ $[M+H]^+$: 350.2478, found: 350.2479.

^{1}H NMR (400 MHz, $CDCl_3$) δ 7.28 (dd, *J* = 7.8, 1.4 Hz, 1H, C^{5}**H**), 7.21 – 7.14 (m, 1H, C^{6}**H**), 7.09 (td, *J* = 7.4, 1.4 Hz, 1H, C^{7}**H**), 6.97 (dd, *J* = 7.6, 1.4 Hz, 1H, C^{8}**H**), 3.97 (s, 2H, C^{1}**H$_2$**), 2.97 (d, *J* = 13.0 Hz, 1H, C^{3}**H$_a$**), 2.85 (d, *J* = 13.0 Hz, 1H, C^{3}**H$_b$**), 2.75 – 2.59 (m, 1H, C^{13}**H$_a$**), 2.54 – 2.40 (m, 1H, C^{13}**H$_b$**), 2.30 (ddd, *J* = 14.4, 11.6, 4.1 Hz, 1H, C^{12}**H$_a$**), 2.21 (s, 3H, C^{18}**H**), 2.16 (s, 6H, C^{16}**H**), 2.12 (s, 1H, N^{2}**H**), 2.01 (s, 6H, C^{17}**H**), 1.97 (dd, *J* = 10.4, 4.1 Hz, 1H, C^{12}**H$_b$**), 1.33 (s, 3H, C^{11}**H**).

^{13}C NMR (151 MHz, $CDCl_3$) δ 211.9 (C^{14}), 141.9 (C^{9}), 140.9 (C^{15}), 135.6 (C^{10}), 135.5 (C^{21}), 133.1 (2 x C^{19}), 127.3 (2 x C^{20}), 126.7 (C^{7}), 126.6 (C^{6}), 126.2 (C^{8}H), 125.8 (C^{7}H), 55.0 (C^{3}), 49.3 (C^{1}), 41.4 (C^{13}), 35.6 (C^{4}), 33.9 (C^{12}), 28.7 (C^{11}), 17.2 (2 x C^{17}), 16.7 (C^{18}), 16.0 (2 x C^{16}).

IR (neat) (cm^{-1}): 2923, 2865, 1698, 1489, 1446, 1407, 1381, 1311, 1118, 930.

3-(2-Benzyl-4-methyl-1,2,3,4-tetrahydroisoquinolin-4-yl)propanoic acid (6b)

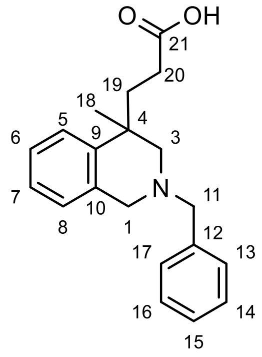

In a microwave vial tetrahydroisoquinoline **5d** (140 mg, 0.32 mmol) was charged and dissolved in HFIP (1.7 mL). To the solution was added concentrated hydrochloric acid (37%, 0.42 mL, 5.1 mmol) and the reaction mixture was heated to 65 °C and stirred for 19 hours. The reaction was allowed to cool to room temperature and diluted with water (10 mL) and CH_2Cl_2 (20 mL). The phases were separated, the aqueous phase was extracted once more with 20 mL CH_2Cl_2 and the combined organic phases dried over $MgSO_4$. After evaporation of the solvent the crude product was purified by silica gel chromatography (3% MeOH in CH_2Cl_2) to give carboxylic acid **6b** (85 mg, 86%) as a colourless oil.

HRMS (ESI): Exact mass calculated for $C_{20}H_{24}NO_2$ $[M+H]^+$: 310.1802, found: 310.1802.

^{1}**H NMR** (400 MHz, $CDCl_3$) δ 8.82 (br s, 1H, C^{21}**OH**), 7.42 – 7.38 (m, 2H, C^{Bn}**H**), 7.34 (ddd, *J* = 7.9, 6.9, 1.0 Hz, 2H, C^{Bn}**H**), 7.31 – 7.27 (m, 1H, C^{Bn}**H**), 7.26 – 7.23 (m, 1H, C^{5}**H**), 7.19 (td, *J* = 7.5, 1.5 Hz, 1H, C^{7}**H**), 7.11 (td, *J* = 7.4, 1.5 Hz, 1H, C^{6}**H**), 6.98 (dd, *J* = 7.6, 1.3 Hz, 1H, C^{8}**H**), 3.74 (d, *J* = 14.7 Hz, 1H, C^{1}$\mathbf{H_a}$), 3.72 – 3.61 (m, 2H, C^{11}**H**), 3.54 (d, *J* = 14.7 Hz, 1H, C^{1}$\mathbf{H_b}$), 2.68 (dd, *J* = 11.6, 1.3 Hz, 1H, C^{3}$\mathbf{H_a}$), 2.42 – 2.30 (m, 2H, C^{3}$\mathbf{H_b}$ + C^{19}$\mathbf{H_a}$), 2.14 (ddd, *J* = 13.1, 10.2, 5.4 Hz, 1H, C^{20}$\mathbf{H_a}$), 2.05 (ddd, *J* = 15.6, 10.1, 5.4 Hz, 1H, C^{19}$\mathbf{H_b}$), 1.89 (ddd, *J* = 13.1, 10.1, 5.4 Hz, 1H, C^{20}$\mathbf{H_b}$), 1.27 (s, 3H, C^{18}**H**)

^{13}**C NMR** (101 MHz, $CDCl_3$) δ 179.5 (C^{21}), 141.5 (C^{9}), 137.7 (C^{10}), 134.4 (C^{12}), 129.4 (C^{13} + C^{17}), 128.5 (C^{14} + C^{16}), 127.5 (C^{15}), 126.9 (C^{8}), 126.6 (C^{7}), 126.1 (C^{6}), 126.0 (C^{5}), 62.9 (C^{11}), 61.8 (C^{1}), 57.0 (C^{3}), 37.8 (C^{4}), 37.4 (C^{20}), 30.4 (C^{19}), 27.8 (C^{18})

IR (neat) (cm^{-1}): 2924, 2799, 1708, 1558, 1493, 1395, 1304, 1211, 1074, 1028, 912.

Alkylation of isoquinolinium salts

General procedure G: Alkylation of isoquinolinium salts with aldehydes

Isoquinolinium salt (0.125 mmol, 1.0 equiv.) and aldehyde (1.0 to 4.0 equiv.) are dissolved in MeCN (0.10 mL ≙ 1.25 M). Upon addition of HCO_2H:NEt_3 5:2 complex (42 µL, 4.0 equiv.) the reaction mixture is heated to 80 ˚C for 18 hours. The reaction was diluted with CH_2Cl_2 (10 mL) and quenched with an aqueous solution of K_2CO_3 (10 mL, 0.1 M). The solution was separated, and the aqueous layer was extracted with CH_2Cl_2 (3 x 10 mL). The organic layers were combined, dried ($MgSO_4$), filtered, and concentrated *in vacuo*. The crude material was purified by flash column chromatography to furnish the respective amines.

(*N*-Benzyl-4-methyl-1,2,3,4-tetrahydroisoquinolin-4-yl)methanol (7a)

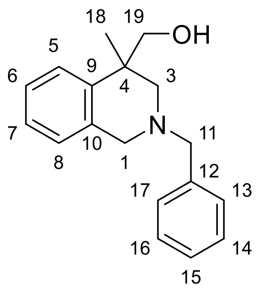

The title compound was prepared according to General Procedure **G** using isoquinolinium salt **4a** (45 mg, 0.125 mmol), formaldehyde (37 wt. % in H_2O**,** 19 µL, 0.25 mmol), 5:2 HCO_2H:Et_3N (42 µL, 0.50 mmol) in MeCN (0.1 mL) and was purified by flash column chromatography (20% EtOAc in pentane) to furnish tetrahydroisoquinoline **7a** (25 mg, 75%) as a colourless oil.

HRMS (ESI): Exact mass calculated for $C_{19}H_{22}NO[M+H]^+$: 268.1696, found: 268.1696.

^{1}H NMR (400 MHz, $CDCl_3$) δ 7.40 – 7.36 (m, 4H, 4 x C^{Ar}**H**), 7.36 – 7.29 (m, 2H, 2 x C^{Ar}**H**), 7.25 (t, *J* = 7.4 Hz, 1H, C^{Ar}**H**), 7.16 (td, *J* = 7.4, 1.3 Hz, 1H, C^{Ar}**H**), 7.01 (dd, *J* = 7.5, 1.4 Hz, 1H, C^{Ar}**H**), 3.91 (dd, *J* = 14.9, 1.8 Hz, 1H, C^1**H$_2$**), 3.78 (d, *J* = 10.0 Hz, 1H, C^{19}**H$_2$**), 3.73 – 3.61 (m, 3H, 2 x C^{11}**H$_2$** + C^{19}**H$_2$**), 3.39 (d, *J* = 14.8 Hz, 1H, C^1**H$_2$**), 3.06 (dd, *J* = 11.4, 1.9 Hz, 1H, C^3**H$_2$**), 2.48 (dd, *J* = 11.4, 2.6 Hz, 1H, C^3**H$_2$**), 1.16 (s, 3H, C^{18}**H$_3$**);

^{13}C NMR (101 MHz, $CDCl_3$) δ 139.6 (C^{Ar}), 137.0 (C^{Ar}), 135.0 (C^{Ar}), 129.3 (2 x $C^{Ar}H$), 128.7 (2 x $C^{Ar}H$), 127.7 ($C^{Ar}H$), 127.2 ($C^{Ar}H$), 126.4 ($C^{Ar}H$), 126.3 ($C^{Ar}H$), 125.7 ($C^{Ar}H$), 76.2 ($C^{19}H_2$), 63.7 (C^3H_2), 63.1 ($C^{11}H_2$), 56.9 (C^1H_2), 39.1 (C^4), 22.5 ($C^{18}H_3$).

IR (neat) (cm^{-1}): 3295, 3029, 2833, 1494, 1452, 1092, 1073, 1050, 1028, 762.

(*N*-Benzyl-4-butyl-1,2,3,4-tetrahydroisoquinolin-4-yl)methanol (7b)

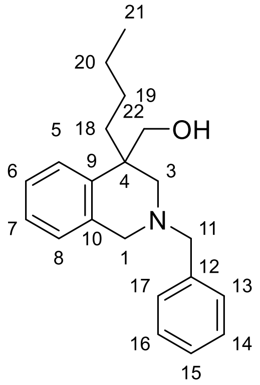

The title compound was prepared according to General Procedure **G** using isoquinolinium salt **4b** (43 mg, 0.125 mmol), formaldehyde (37 wt. % in H_2O**,** 19 μL, 0.25 mmol), 5:2 HCO_2H:Et_3N (42 μL, 0.50 mmol) in MeCN (0.1 mL) and was purified by flash column chromatography (12-20% EtOAc in pentane) to furnish tetrahydroisoquinoline **7b** (28 mg, 72%) as a colourless oil. Spectroscopic data are in accordance with those described in the literature.[4]

^{1}H NMR (400 MHz, $CDCl_3$) δ 7.37 (d, *J* = 4.4 Hz, 4H, 4 x C^{Ar}**H**), 7.34 – 7.22 (m, 3H, 3 x C^{Ar}**H**), 7.14 (ddd, *J* = 8.5, 7.1, 1.6 Hz, 1H, C$^{6/7}$**H**), 6.99 (d, *J* = 7.6 Hz, 1H, C$^{5/8}$**H**), 3.85 (dd, *J* = 14.7, 1.9 Hz, 1H, C^1**H$_2$**), 3.79 (d, *J* = 9.8 Hz, 1H, C^{22}**H$_2$**), 3.71 (d, *J* = 12.8 Hz, 1H, C^{11}**H$_2$**), 3.63 (d, *J* = 7.3 Hz, 1H, C^{11}**H$_2$**), 3.60 (dd, *J* = 7.1 Hz, 2.7 Hz, 1H, C^{22}**H$_2$**) 3.32 (d, *J* = 14.6 Hz, 1H, C^1**H$_2$**), 2.99 (dd, *J* = 11.4, 1.9 Hz, 1H, C^3**H$_2$**), 2.65 (dd, *J* = 11.4, 2.7 Hz, 1H, C^3**H$_2$**), 1.68 (ddd, *J* = 13.9, 12.2, 4.6 Hz, 1H, C^{18}**H$_2$**), 1.40 (ddd, *J* = 13.9, 12.0, 4.7 Hz, 1H, C^{18}**H$_2$**), 1.31 – 1.20 (m, 2H, C^{20}**H$_2$**), 1.17 – 0.97 (m, 2H, C^{19}**H$_2$**), 0.85 (t, *J* = 7.3 Hz, 3H, C^{21}**H$_3$**).

^{13}C NMR (101 MHz, $CDCl_3$) δ 138.2 (C^{Ar}), 137.0 (C^{Ar}), 135.7 (C^{Ar}), 129.3 (2 x C^{Ar}H), 128.7 (2 x C^{Ar}H), 127.7 (C^{Ar}H), 127.2 (C^{Ar}H), 126.3 (C^{Ar}H), 126.1 (C^{Ar}H), 125.8 (C^{Ar}H), 76.3 (C^{22}H$_2$), 63.3 (C^{11}H$_2$), 61.0 (C^{3}H$_2$), 56.9 (C^{1}H$_2$), 42.3 (C^4), 35.1 (C^{18}H$_2$), 26.2 (C^{19}H$_2$), 23.6 (C^{20}H$_2$), 14.0 (C^{21}H$_3$).

(*N*-Benzyl-4-methyl-1,2,3,4-tetrahydroisoquinolin-4-yl)methanol (7a)

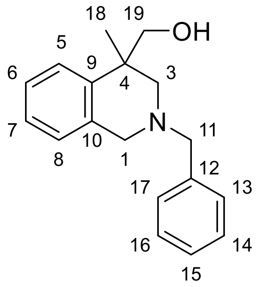

The title compound was prepared according to General Procedure **G** using isoquinolinium salt **4k** (43 mg, 0.125 mmol), formaldehyde (37 wt. % in H_2O**,** 29 μL, 0.375 mmol), 5:2 HCO_2H:Et_3N (63 μL, 0.75 mmol) in MeCN (0.1 mL) and was purified by flash column chromatography (20% EtOAc in pentane) to furnish tetrahydroisoquinoline **7a** (23 mg, 69%) as a colourless oil. For full characterisation data see above.

2,4-Dibenzyl-1,2,3,4-tetrahydroisoquinoline (7c)

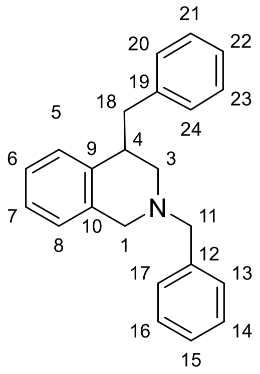

The title compound was prepared according to General Procedure **G** using isoquinolinium salt **4k** (43 mg, 0.125 mmol), benzaldehyde (13 mg, 0.125 mmol), 5:2 HCO_2H:Et_3N (42 μL, 0.5 mmol) in MeCN (0.1 mL) and was purified by column chromatography (2.5% EtOAc in pentane) to furnish

tetrahydroisoquinoline **7c** (28 mg, 72%) as a colourless oil. Spectroscopic data are in accordance with those described in the literature.[12]

^{1}H NMR (400 MHz, $CDCl_3$) δ 7.48 – 7.42 (m, 2H, 2 x C^{Ar}**H**), 7.42 – 7.31 (m, 3H, 3 x C^{Ar}**H**), 7.25 – 7.12 (m, 6H, 6 x C^{Ar}**H**), 7.07 – 7.00 (m, 3H, 3 x C^{Ar}**H**), 3.90 (d, *J* = 14.8 Hz, 1H, C^{1}**H$_2$**), 3.74 (d, *J* = 12.9 Hz, 1H, C^{11}**H$_2$**), 3.56 (d, *J* = 12.8 Hz, 1H, C^{11}**H$_2$**), 3.46 (d, *J* = 14.8 Hz, 1H, C^{1}**H$_2$**), 3.07 – 2.97 (m, 3H, C^{18}**H$_2$** + C^{4}**H**), 2.79 (d, *J* = 11.5 Hz, 1H, C^{3}**H$_2$**), 2.39 (d, *J* = 11.6 Hz, 1H, C^{3}**H$_2$**);

^{13}C NMR (101 MHz, $CDCl_3$) δ 140.9 (C^{Ar}), 138.7 (C^{Ar}), 138.7 (C^{Ar}), 135.2 (C^{Ar}), 129.6 (2 x C^{Ar}H), 129.5 (2 x C^{Ar}H), 128.8 (C^{Ar}H), 128.5 (2 x C^{Ar}H), 128.4 (2 x C^{Ar}H), 127.3 (C^{Ar}H), 126.6 (C^{Ar}H), 126.3 (C^{Ar}H), 126.0 (2 x C^{Ar}H), 63.1 (C^{11}H$_2$), 56.8 (C^{1}H$_2$), 53.1 (C^{3}H$_2$), 42.9 (C^{18}H$_2$), 41.5 (C^{4}H).

4-((2-Benzyl-1,2,3,4-tetrahydroisoquinolin-4-yl)methyl)-2-methoxyphenol (7d)

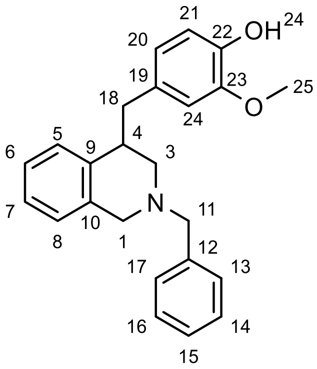

The title compound was prepared according to General Procedure **G** using isoquinolinium salt **4k** (40 mg, 0.115 mmol), vanillin (26 mg, 0.17 mmol) and 5:2 HCO_2H:Et_3N (39 μL, 0.46 mmol) in MeCN (0.1 mL) and was purified by column chromatography (10% EtOAc in pentane) to furnish phenol **7d** (30 mg, 72%) as a colourless oil.

HRMS (ESI): Exact mass calculated for $C_{24}H_{26}NO_2$ $[M+H]^+$: 360.1958 found: 360.1957.

^{1}H NMR (400 MHz, $CDCl_3$) δ 7.47 – 7.42 (m, 2H, C^{13}**H** + C^{17}**H**), 7.40 – 7.34 (m, 2H, C^{14}**H** + C^{16}**H**), 7.34 – 7.28 (m, 1H, C^{15}**H**), 7.21 – 7.11 (m, 3H, C^{6}**H** – C^{8}**H**), 7.06 – 6.99 (m, 1H, C^{5}**H**), 6.77 (d, *J* = 8.0 Hz, 1H, C^{21}**H**), 6.63 (d, *J* = 1.9 Hz, 1H, C^{24}**H**), 6.51 (dd, *J* = 8.0, 1.9 Hz, 1H, C^{20}**H**), 5.52 (br s, 1H, O^{24}**H**), 3.86 (d, *J* = 14.9 Hz, 1H, C^{1}**H$_a$**), 3.80 (s, 3H, C^{25}**H$_3$**), 3.72 (d, *J* = 13.1 Hz, 1H, C^{11}**H$_a$**), 3.59 (d, *J* = 13.0 Hz, 1H, C^{11}**H$_b$**), 3.45 (d, *J* = 14.9 Hz, 1H, C^{1}**H$_b$**), 2.96 (d, *J* = 5.2 Hz, 3H, C^{4}**H** + C^{18}**H$_2$**), 2.80 (ddd, *J* = 11.1, 1.8 Hz, 1H, C^{3}**H$_a$**), 2.45 (dd, *J* = 11.5, 3.3 Hz, 1H, C^{3}**H$_b$**).

^{13}C NMR (101 MHz, $CDCl_3$) δ 146.4 (C^{22}), 143.9 (C^{23}), 138.8 (C^{9}), 138.6 (C^{19}), 135.1 (C^{12}), 133.1 (C^{10}), 129.3 (C^{13}H + C^{17}H), 128.8 (C^{8}H), 128.5 (C^{14}H + C^{16}H), 127.3 (C^{15}H), 126.6 (C^{5}H), 126.2 (C^{7}H), 125.9 (C^{6}H), 122.2 (C^{20}H), 114.3 (C^{21}H), 111.9 (C^{24}H), 63.1 (C^{11}H$_2$), 56.8 (C^{1}H$_2$), 56.0 (C^{25}H$_3$), 53.6 (C^{3}H$_2$), 42.6 (C^{18}H$_2$), 41.6 (C^{4}H).

IR (neat) (cm^{-1}): 3531, 3025, 2921, 2798, 1512, 1429, 1268, 1206, 1120, 1033, 856, 821.

[12]B. Sundararaju, M. Achard, G. V. M. Sharma, C. Bruneau, *J. Am. Chem. Soc*. **2011**, *133*, 10340–10343.

4-((2-Benzyl-1,2,3,4-tetrahydroisoquinolin-4-yl)methyl)benzonitrile (7e)

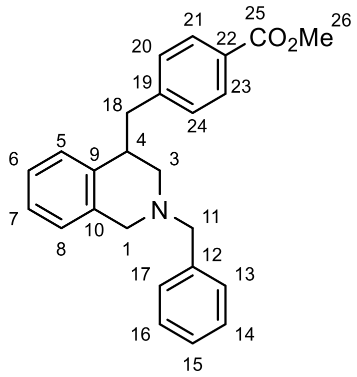

The title compound was prepared according to General Procedure **G** using isoquinolinium salt **4k** (40 mg, 0.12 mmol), methyl 4-formylbenzoate (19 mg, 0.12 mmol) and 5:2 HCO_2H:Et_3N (39 μL, 0.46 mmol) in MeCN (0.1 mL) and was purified by column chromatography (5% Et_2O in pentane) to furnish methyl ester **7e** (27 mg, 63%) as a colourless oil.

HRMS (ESI): Exact mass calculated for $C_{25}H_{26}NO_2$ $[M+H]^+$: 372.1958 found: 372.1958.

^{1}H NMR (400 MHz, $CDCl_3$) δ 7.87 (d, *J* = 8.2 Hz, 2H, C^{21}**H** + C^{23}**H**), 7.46 – 7.33 (m, 5H, C^{Bn}**H**), 7.21 – 7.12 (m, 3H, C^5**H** – C^7**H**), 7.04 (dd, *J* = 7.8, 5.8 Hz, 3H, C^{20}**H** + C^{24}**H** + C^8**H**), 3.92 (s, 3H, C^{26}**H₃**), 3.88 – 3.95 (m, 1H, C^1**H$_a$**), 3.75 (d, *J* = 12.7 Hz, 1H, C^{11}**H$_a$**), 3.48 (m, 2H, C^1**H$_b$** + C^{11}**H$_b$**), 3.14 – 2.95 (m, 3H, C^{18}**H$_2$** + C^4**H**), 2.73 (ddd, *J* = 11.8, 1.9, 1.9 Hz, 1H, C^3**H$_a$**), 2.35 (dd, *J* = 11.6, 3.7 Hz, 1H, C^3**H$_b$**).

^{13}C NMR (101 MHz, $CDCl_3$) δ 167.2 (C^{25}O), 146.6 (C^{19}), 138.6 (C^9), 138.2 (C^{12}), 135.1 (C^{22}), 129.6 (C^{21}H + C^{23}H), 129.5 (C^{13}H + C^{17}H), 129.4 (C^{20}H + C^{24}H), 128.7 (C^6H), 128.4 (C^{14}H + C^{16}H), 127.9 (C^{10}), 127.3 (C^{15}H), 126.6 (C^8H), 126.2 (C^7H), 126.0 (C^5H), 62.9 ($C^{11}H_2$), 56.9 (C^1H_2), 52.7 (C^3H_2), 52.0 ($C^{26}H_3$), 42.8 ($C^{18}H_2$), 41.3 (C^4H).

IR (neat) (cm^{-1}): 3027, 2921, 2799, 1718, 1434, 1275, 1177, 1101, 757, 740, 723.

4-((2-Benzyl-1,2,3,4-tetrahydroisoquinolin-4-yl)methyl)benzonitrile (7f)

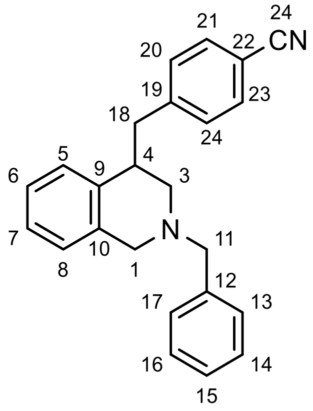

The title compound was prepared according to General Procedure **G** using isoquinolinium salt **4k** (40 mg, 0.115 mmol), 4-cyanobenzaldehyde (23 mg, 0.17 mmol) and 5:2 HCO_2H:Et_3N (39 μL, 0.46 mmol) in MeCN (0.1 mL) and was purified by column chromatography (10% EtOAc in pentane) to furnish nitrile **7f** (23 mg, 58%) as a colourless solid.

m.p.: 113 °C ($CHCl_3$)

HRMS (ESI): Exact mass calculated for $C_{24}H_{23}N_2$ $[M+H]^+$: 339.1856, found: 339.1855.

^{1}H NMR (600 MHz, $CDCl_3$) δ 7.43 (d, *J* = 8.2 Hz, 2H, C^{21}**H** + C^{23}**H**), 7.40 – 7.33 (m, 5H, C^{Bn}**H**), 7.22 – 7.15 (m, 2H, C^6**H** + C^7**H**), 7.13 (dd, *J* = 6.6, 2.6 Hz, 1H, C^5**H**), 7.07 – 7.04 (m, 1H, C^8**H**), 6.98 (d, *J* = 7.8 Hz, 2H, C^{20}**H** + C^{24}**H**), 3.95 (d, *J* = 14.9 Hz, 1H, C^1**H$_a$**), 3.78 (d, *J* = 12.7 Hz, 1H, C^{11}**H$_a$**), 3.49 (d, *J* = 14.8 Hz, 1H, C^1**H$_b$**), 3.44 (d, *J* = 12.7 Hz, 1H, C^{11}**H$_b$**), 3.19 – 3.01 (m, 1H, C^{18}**H$_a$**), 3.00 – 2.90 (m, 2H, C^{18}**H$_b$** + C^4**H**), 2.68 (d, *J* = 11.6 Hz, 1H, C^3**H$_a$**), 2.42 – 2.26 (m, 1H, C^3**H$_b$**).

^{13}C NMR (151 MHz, $CDCl_3$) δ 146.6 (C^{19}), 138.5 (C^{12}), 137.8 (C^{9}), 135.1 (C^{10}), 132.0 (C^{20}H + C^{24}H), 130.1 (C^{21}H + C^{23}H), 129.7 (C^{13}H + C^{17}H), 128.7 (C^{5}H), 128.4 (C^{14}H + C^{16}H), 127.3 (C^{15}H), 126.6 (C^{8}H), 126.3 (C^{7}H), 126.2 (C^{6}H), 119.1 (C^{22}), 109.7 (C^{24}N), 62.8 ($C^{11}H_2$), 57.1 ($C^{1}H_2$), 52.1 ($C^{3}H_2$), 42.8 ($C^{18}H_2$), 41.2 (C^{4}H).

IR (neat) (cm^{-1}): 3028, 2922, 2800, 2755, 2226, 1606, 1091, 851, 749, 700.

***N*-Benzyl-4-(furan-2-ylmethyl)-1,2,3,4-tetrahydroisoquinoline (7g)**

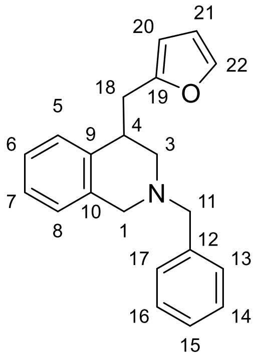

The title compound was prepared according to General Procedure **G** using isoquinolinium salt **4k** (43 mg, 0.125 mmol), furfural (13 mg, 0.125 mmol), 5:2 HCO_2H:Et_3N (42 μL, 0.5 mmol) in MeCN (0.1 mL) and was purified by column chromatography (2% EtOAc in pentane) to furnish tetrahydroisoquinoline **7g** (33 mg, 87%) as a colourless oil.

HRMS (ESI): Exact mass calculated for $C_{21}H_{22}NO$ $[M+H]^+$: 304.1696, found: 304.1696.

^{1}H NMR (400 MHz, $CDCl_3$) δ 7.44 – 7.27 (m, 6H, 6 x C^{Ar}**H**), 7.18 – 7.10 (m, 3H, 3 x C^{Ar}**H**), 7.05 – 7.00 (m, 1H,), 622 (dd, *J* = 3.2, 1.8 Hz, 1H, $C^{20/21}$**H**), 5.70 (d, *J* = 3.0 Hz, 1H, $C^{20/21}$**H**), 3.86 (d, *J* = 14.5 Hz, 1H, C^{1}**H**), 3.74 (d, *J* = 13.0 Hz, 1H, C^{11}**H₂**), 3.57 (d, *J* = 13.0 Hz, 1H, C^{11}**H₂**), 3.46 (d, *J* = 14.8 Hz, 1H, C^{1}**H₂**), 3.16 – 3.07 (m, 2H, C^{4}**H** + C^{18}**H₂**), 3.02 – 2.90 (m, 1H, C^{18}**H₂**), 2.83 – 2.48 (m, 1H, C^{3}**H₂**), 2.48 (dd, *J* = 11.4, 3.3 Hz, 1H, C^{3}**H₂**);

^{13}C NMR (101 MHz, $CDCl_3$) δ 154.8 (C^{19}H), 141.1 (C^{Ar}H), 138.9 (C^{Ar}), 138.1 (C^{Ar}), 135.3 (C^{Ar}), 129.3 (2 x C^{Ar}H), 128.8 (C^{Ar}H), 128.4 (2 x C^{Ar}H), 127.2 (C^{Ar}H), 126.6 (C^{Ar}H), 126.3 (C^{Ar}H), 126.1 (C^{Ar}H), 110.2 ($C^{20/21}$H), 106.6 ($C^{20/21}$H), 63.0 ($C^{11}H_2$), 56.8 ($C^{1}H_2$), 53.8 ($C^{3}H_2$), 38.6 (C^{4}H), 35.1 ($C^{18}H_2$);

IR (neat) (cm^{-1}): 3026, 2911, 2799, 2756, 1598, 1368, 1145, 1095, 1029, 1010, 922.

***N*-Benzyl-4-(pyridin-2-ylmethyl)-1,2,3,4-tetrahydroisoquinoline (7h)**

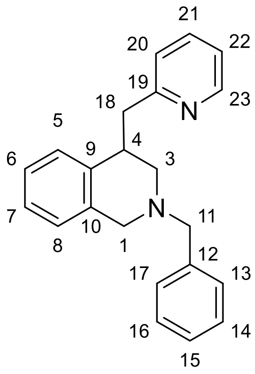

The title compound was prepared according to General Procedure **G** using isoquinolinium salt **4k** (43 mg, 0.125 mmol), picolinaldehyde (13 mg, 0.125 mmol), 5:2 HCO_2H:Et_3N (42 μL, 0.50 mmol) in MeCN (0.1 mL) and was purified by flash column chromatography (0.5% MeOH in CH_2Cl_2) to furnish tetrahydroisoquinoline **7h** (20 mg, 51%) as a colourless oil.

HRMS (ESI): Exact mass calculated for $C_{22}H_{23}N_2$ $[M+H]^+$: 315.1856, found: 315.1857.

^{1}H NMR (400 MHz, $CDCl_3$) δ 8.56 (ddd, *J* = 4.9, 1.9, 0.9 Hz, 1H, C^{23}**H**), 7.44 – 7.27 (m, 6H, 6 x C^{Ar}**H**), 7.23 – 7.17 (m, 1H, C^{Ar}**H**), 7.16 – 7.11 (m, 2H, 2 x C^{Ar}**H**), 7.09 – 7.00 (m, 2H, 2 x C^{Ar}**H**), 6.67 (dt, *J* = 7.8, 1.1 Hz,

1H, C^{Ar}**H**), 3.91 (dd, *J* = 14.8, 1.3 Hz, 1H, C^{1}**H_2**), 3.73 (d, *J* = 12.9 Hz, 1H, C^{11}**H_2**), 3.52 – 3.43 (m, 2H, C^{1}**H_2** + C^{11}**H_2**), 3.41 – 3.31 (m, 1H, C^{4}**H**), 3.21 – 3.09 (m, 2H, C^{18}**H_2**), 2.70 (ddd, *J* = 11.6, 2.8, 1.4 Hz, 1H, C^{3}**H_2**), 2.40 (dd, *J* = 11.6, 4.0 Hz, 1H, C^{3}**H_2**);

^{13}C NMR (101 MHz, $CDCl_3$) δ 160.7 (C^{19}), 149.6 (C^{23}H), 138.8 (C^{Ar}), 138.6 (C^{Ar}), 136.1 (C^{Ar}H), 135.1 (C^{Ar}), 129.5 (2 x C^{Ar}H), 128.9 (C^{Ar}H), 128.4 (2 x C^{Ar}H), 127.2 (C^{Ar}H), 126.6 (C^{Ar}H), 126.3 (C^{Ar}H), 126.0 (C^{Ar}H), 124.4 (C^{Ar}H), 121.1 (C^{Ar}H), 63.0 ($C^{11}H_2$), 57.0 ($C^{1}H_2$), 53.5 ($C^{3}H_2$), 45.2 ($C^{18}H_2$), 39.6 (C^{4}H);

IR (neat) (cm^{-1}): 3026, 2798, 2756, 1590, 1568, 1470, 1438, 1145, 1094, 1029, 756, 700.

N-Benzyl-4-hexyl-1,2,3,4-tetrahydroisoquinoline (7i)

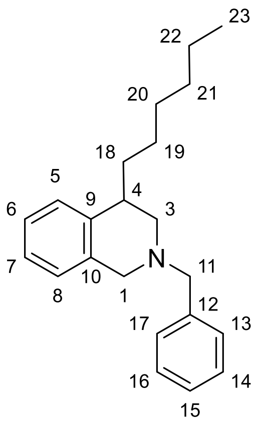

The title compound was prepared according to General Procedure **G** using isoquinolinium salt **4k** (43 mg, 0.125 mmol), hexanal (13 mg, 0.125 mmol), 5:2 $HCO_2H{:}Et_3N$ (42 µL, 0.500 mmol) in MeCN (0.1 mL) and was purified by column chromatography (1.5% EtOAc in pentane) to furnish tetrahydroisoquinoline **7i** (27 mg, 70%) as a colourless oil. Spectroscopic data are in accordance with those described in the literature.[12]

^{1}H NMR (400 MHz, $CDCl_3$) δ 7.44 – 7.39 (m, 2H, 2 x C^{Ar}**H**), 7.37 – 7.31 (m, 2H, 2 x C^{Ar}**H**), 7.30 – 7.25 (m, 1H, C^{Ar}**H**), 7.22 – 7.13 (m, 2H, 2 x C^{Ar}**H**), 7.10 (td, *J* = 7.2, 1.9 Hz, 1H, C^{Ar}**H**), 6.99 (dd, *J* = 7.5, 1.4 Hz, 1H, C^{Ar}**H**), 3.80 – 3.73 (m, 2H, C^{1}**H_2** + C^{11}**H_2**), 3.58 (d, *J* = 13.2 Hz, 1H, C^{11}**H_2**), 3.51 (d, *J* = 14.8 Hz, 1H, C^{1}**H_2**), 2.83 – 2.73 (m, 1H, C^{4}**H**), 2.74 – 2.68 (m, 1H, C^{3}**H_2**), 2.65 – 2.57 (m, 1H, C^{3}**H_2**), 1.84 – 1.73 (m, 1H, C^{18}**H_2**), 1.72 – 1.62 (m, 1H, C^{18}**H_2**), 1.33 – 1.20 (m, 8H, C^{19}**H_2** + C^{20}**H_2**+ C^{21}**H_2**+ C^{22}**H_2**), 0.94 – 0.86 (m, 3H, C^{23}**H_3**);

^{13}C NMR (101 MHz, $CDCl_3$) δ 139.7 (C^{Ar}), 138.9 (C^{Ar}), 135.0 (C^{Ar}), 129.1 (2 x C^{Ar}H), 128.4, (C^{Ar}H), 128.4 (2 x C^{Ar}H), 127.2 (C^{Ar}H), 126.5 (C^{Ar}H), 126.2 (C^{Ar}H), 125.6 (C^{Ar}H), 63.0 ($C^{11}H_2$), 56.9 ($C^{1}H_2$), 54.4 ($C^{3}H_2$), 38.9 (C^{4}H), 36.2 ($C^{18}H_2$), 32.0 ($C^{19}H_2$), 29.7 ($C^{20}H_2$), 27.5 ($C^{21}H_2$), 22.8 ($C^{22}H_2$), 14.3 ($C^{23}H_3$).

N-Benzyl-4-(2,2,2-trifluoroethyl)-1,2,3,4-tetrahydroisoquinoline (7j)

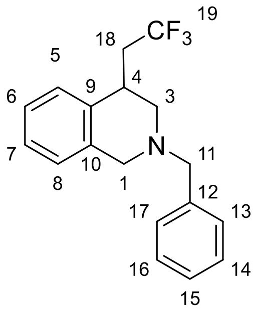

The title compound was prepared according to General Procedure **G** using isoquinolinium salt **4k** (43 mg, 0.125 mmol), 2,2,2-trifluoroacetaldehyde monohydrate (72% in H_2O) (20 mg, 0.125 mmol), 5:2 $HCO_2H{:}Et_3N$ (42 µL, 0.50 mmol) in MeCN (0.1 mL) and was purified by column chromatography (1% EtOAc in pentane) to furnish tetrahydroisoquinoline **7j** (8 mg, 21%) as a colourless oil.

HRMS (ESI): Exact mass calculated for $C_{18}H_{19}F_3N$+ $[M+H]^+$: 306.1464, found: 306.1465.

^{1}**H NMR** (400 MHz, $CDCl_3$) δ 7.40 – 7.33 (m, 4H, 4 x C^{Ar}**H**), 7.31 – 7.26 (m, 1H, C^{Ar}**H**), 7.21 – 7.12 (m, 3H, 3 x C^{Ar}**H**), 6.99 (d, *J* = 7.4 Hz, 1H, C^{Ar}**H**), 3.82 (d, *J* = 15.0 Hz, 1H, C^{1}**H_2**), 3.69 (d, *J* = 3.8 Hz, 2H, C^{11}**H_2**), 3.40 (d, *J* = 15.0 Hz, 1H, C^{1}**H_2**), 3.22 – 3.14 (m, 1H, C^{4}**H_2**), 2.98 (d, *J* = 11.7 Hz, 1H, C^{3}**H_2**), 2.81 (dt, *J* = 15.3, 10.7 Hz, 1H, C^{18}**H_2**), 2.62 (d, *J* = 11.8 Hz, 1H, C^{3}**H_2**), 2.33 (ddd, *J* = 15.2, 11.9, 3.3 Hz, 1H, C^{18}**H_2**);
^{13}**C NMR** (101 MHz, $CDCl_3$) δ 138.3 (C^{Ar}), 137.0 (C^{Ar}), 135.3 (C^{Ar}), 129.2 (2 x C^{Ar}H), 128.7 (C^{Ar}H), 128.5 (2 x C^{Ar}H), 127.4 (C^{Ar}H), 127.1 (q, *J* = 277.4 Hz, $C^{19}F_3$), 126.8 (C^{Ar}H), 126.8 (C^{Ar}H), 126.6 (C^{Ar}H), 63.0 ($C^{11}H_2$), 56.0 ($C^{1}H_2$), 54.3 ($C^{3}H_2$), 40.1 (q, *J* = 26.9 Hz, $C^{18}H_2$), 33.9 (C^{4}H);
IR (neat) (cm^{-1}):2806, 2762, 2360, 2341, 1440, 1371, 1129, 1108, 747, 725, 700.

***N*-Benzyl-4-(pent-4-en-1-yl)-1,2,3,4-tetrahydroisoquinoline (7k)**

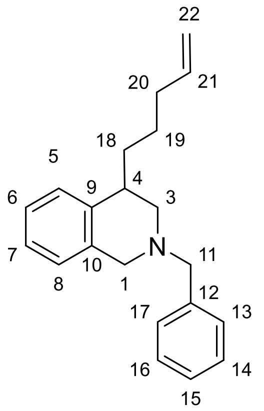

The title compound was prepared according to General Procedure **G** using isoquinolinium salt **4k** (43 mg, 0.125 mmol), pent-4-enal (22 mg, 0.25 mmol), 5:2 $HCO_2H:Et_3N$ (53 µL, 0.625 mmol) in MeCN (0.1 mL) and was purified by column chromatography (CH_2Cl_2) to furnish tetrahydroisoquinoline **7k** (15 mg, 41%) as a colourless oil.

HRMS (ESI): Exact mass calculated for $C_{21}H_{26}N$ $[M+H]^+$: 292.2060, found: 292.2060.

^{1}**H NMR** (400 MHz, $CDCl_3$) δ 7.35 – 7.29 (m, 2H, 2 x C^{Ar}**H**), 7.29 – 7.22 (m, 2H, 2 x C^{Ar}**H**), 7.22 – 7.16 (m, 1H, 1 x C^{Ar}**H**), 7.12 – 6.99 (m, 3H, 3 x C^{Ar}**H**), 6.94 – 6.88 (m, 1H, C^{Ar}**H**), 5.70 (ddt, *J* = 16.9, 10.2, 6.7 Hz, 1H, C^{21}**H**), 4.96 – 4.82 (m, 2H, C^{22}**H_2**), 3.73 – 3.64 (m, 2H, C^{11}**H_2**+ C^{1}**H_2**), 3.49 (d, *J* = 13.1 Hz, 1H, C^{11}**H_2**), 3.42 (d, *J* = 14.8 Hz, 1H, C^{1}**H_2**), 2.75 – 2.65 (m, 1H, C^{4}**H**), 2.63 (ddd, *J* = 11.3, 4.3, 1.1 Hz, 1H, C^{3}**H_2**), 2.51 (dd, *J* = 11.4, 4.4 Hz, 1H, C^{3}**H_2**), 2.04 – 1.89 (m, 2H, C^{20}**H_2**), 1.79 – 1.67 (m, 1H, C^{18}**H_2**), 1.65 – 1.54 (m, 1H, C^{18}**H_2**), 1.33 – 1.21 (m, 2H, C^{19}**H_2**);
^{13}**C NMR** (101 MHz, $CDCl_3$) δ 139.5 (C^{Ar}), 139.0 (C^{21}H), 139.0 (C^{Ar}) 135.0 (C^{Ar}), 129.1 (2 x C^{Ar}H), 128.4 (C^{Ar}), 128.4 (2 x C^{Ar}H), 127.2 (C^{Ar}H), 126.5 (C^{Ar}H), 126.2 (C^{Ar}H), 125.7 (C^{Ar}H), 114.6 ($C^{22}H_2$), 63.0 ($C^{11}H_2$), 56.9 ($C^{1}H_2$), 54.3 ($C^{3}H_2$), 38.7 (C^{4}H), 35.7 ($C^{18}H_2$), 34.1 ($C^{20}H_2$), 26.9 ($C^{19}H_2$);
IR (neat) (cm^{-1}): 3063, 3026, 2925, 2855, 2795, 1640, 1494, 1096, 1029, 742.

***N*-Benzyl-4-(cyclopropylmethyl)-1,2,3,4-tetrahydroisoquinoline (7l)**

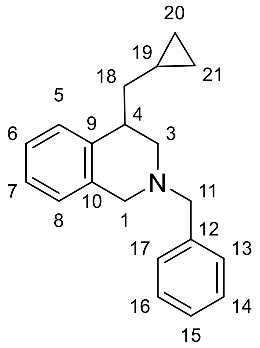

The title compound was prepared according to General Procedure **G** using isoquinolinium salt **4k** (43 mg, 0.125 mmol, 1.0 eq.), cyclopropanecarbaldehyde (9 mg, 0.125 mmol, 1.0 eq.), 5:2 $HCO_2H:Et_3N$ (42 µL, 0.500 mmol, 4.0 eq.) in MeCN (0.1 mL) and was purified by column chromatography (2% EtOAc in pentane) to furnish tetrahydroisoquinoline **7l** (27 mg, 78%) as a colourless oil.

HRMS (ESI): Exact mass calculated for $C_{20}H_{24}N$ $[M+H]^+$: 278.1903, found: 278.1904.

^{1}H NMR (400 MHz, $CDCl_3$) δ 7.42 – 7.37 (m, 2H, 2 x C^{Ar}**H**), 7.35 – 7.30 (m, 2H, 2 x C^{Ar}**H**), 7.28 – 7.23 (m, 1H, C^{Ar}**H**), 7.19 – 7.04 (m, 3H, 3 x C^{Ar}**H**), 6.99 – 6.93 (m, 1H, C^{Ar}**H**), 3.78 – 3.68 (m, 2H, C^1**H$_2$** + C^{11}**H$_2$**), 3.61 (d, *J* = 13.1 Hz, 1H, C^{11}**H$_2$**), 3.45 (d, *J* = 14.8 Hz, 1H, C^1**H$_2$**), 2.95 – 2.86 (m, 2H, C^4**H** + C^3**H$_2$**), 2.72 – 2.64 (m, 1H, C^3**H$_2$**), 1.71 (ddd, *J* = 13.8, 9.4, 7.0 Hz, 1H, C^{18}**H$_2$**), 1.55 (ddd, *J* = 13.8, 6.9, 4.2 Hz, 1H, C^{18}**H$_2$**), 0.69 – 0.60 (m, 1H, C^{19}**H**), 0.39 (ddt, *J* = 7.8, 4.8, 1.3 Hz, 2H, C$^{20/21}$**H$_2$**), 0.02 (ddd, *J* = 5.0, 3.2, 1.8 Hz, 2H, C$^{20/21}$**H$_2$**);

^{13}C NMR (101 MHz, $CDCl_3$) δ 139.3 (C^{Ar}), 138.9 (C^{Ar}), 135.0 (C^{Ar}), 129.2 (2 x C^{Ar}H), 128.5 (C^{Ar}H), 128.4 (2 x C^{Ar}H), 127.2 (C^{Ar}H), 126.5 (C^{Ar}H), 126.1 (C^{Ar}H), 125.6 (C^{Ar}H), 63.2 (C^{11}H$_2$), 56.8 (C^{1}H$_2$), 54.8 (C^{3}H$_2$), 41.3 (C^{18}H$_2$), 39.8 (C^{4}H), 9.6 (C^{19}H), 5.5 (C$^{20/21}$H$_2$), 4.3 (C$^{20/21}$H$_2$);

IR (neat) (cm^{-1}): 3071, 3025, 2912, 2753, 1493, 1454, 1367, 1144, 1095, 1028, 823, 741, 699.

***N*-Benzyl-4-(cyclobutylmethyl)-1,2,3,4-tetrahydroisoquinoline (7m)**

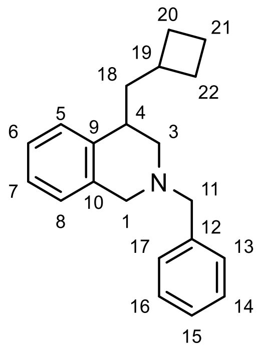

The title compound was prepared according to General Procedure **G** using isoquinolinium salt **4k** (40 mg, 0.115 mmol), formylcyclobutane (19 mg, 0.23 mmol) and 5:2 $HCO_2H:Et_3N$ (39 µL, 0.46 mmol) in MeCN (0.1 mL) and was purified by column chromatography (4% EtOAc in pentane) to furnish tetrahydroisoquinoline **7m** (27 mg, 80%) as a colourless oil.

HRMS (ESI): Exact mass calculated for $C_{21}H_{26}N$ $[M+H]^+$: 292.2060, found: 292.2062.

^{1}H NMR (400 MHz, $CDCl_3$) δ 7.45 – 7.39 (m, 2H, C^{13}**H** + C^{17}**H**), 7.35 (tt, *J* = 6.4, 1.0 Hz, 2H, C^{14}**H** + C^{16}**H**), 7.32 – 7.27 (m, 1H, C^{15}**H**), 7.19 – 7.15 (m, 2H, C^7**H** + C^8**H**), 7.14 – 7.05 (m, 1H, C^6**H**), 6.98 (dt, *J* = 7.6, 1.1 Hz, 1H, C^5**H**), 3.76 (d, *J* = 12.3 Hz, 1H, C^1**H$_a$**), 3.72 (d, *J* = 10.6 Hz, 1H, C^{11}**H$_a$**), 3.62 (d, *J* = 13.1 Hz, 1H, C^{11}**H$_b$**), 3.48 (d, *J* = 14.8 Hz, 1H, C^1**H$_b$**), 2.80 – 2.71 (m, 1H, C^4**H**), 2.67 (ddd, *J* = 11.3, 4.3, 1.0 Hz, 1H, C^3**H$_a$**), 2.60 (dd, *J* = 11.4, 4.5 Hz, 1H, C^3**H$_b$**), 2.40 – 2.24 (m, 1H, C^{19}**H**), 2.08 – 1.97 (m, 2H, C^{20}**H$_a$** + C^{22}**H$_a$**), 1.95 – 1.86 (m, 1H, C^{18}**H$_a$**), 1.86 – 1.75 (m, 3H, C^{18}**H$_b$** + C^{21}**H$_2$**), 1.72 – 1.55 (m, 2H, C^{20}**H$_b$** + C^{22}**H$_b$**).

^{13}C NMR (101 MHz, $CDCl_3$) δ 139.5 (C^{9}), 138.8 (C^{12}), 135.0 (C^{10}), 129.2 (C^{13} + C^{17}), 128.42 (C^{8}), 128.36 (C^{14} + C^{16}), 127.2 (C^{15}), 126.5 (C^{5}), 126.2 (C^{7}), 125.6 (C^{6}), 63.0 (C^{11}), 56.7 (C^{1}), 54.8 (C^{3}), 43.8 (C^{18}), 37.2 (C^{4}), 34.4 (C^{19}), 29.2 (C^{20}), 28.6 (C^{22}), 18.6 (C^{21}).

IR (neat) (cm^{-1}): 2925, 2863, 2794, 1367, 1143, 1095, 1071, 1028, 914, 739, 698.

***N*-Benzyl-4-cyclohexyl-1,2,3,4-tetrahydroisoquinoline (7n)**

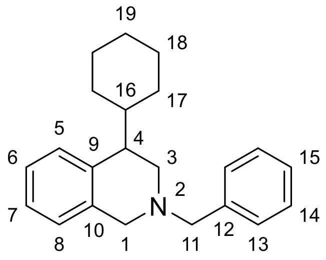

The title compound was prepared according to modified General Procedure **G** using isoquinolinium salt **4k** (40 mg, 0.125 mmol), cyclohexanone (25 mg, 0.25 mmol), 5:2 HCO_2H:Et_3N (42 µL, 0.50 mmol) and $[RhCp^*Cl_2]_2$ (0.01 mol%) in MeCN (0.1 mL) and was purified by column chromatography (4% EtOAc in pentane) to furnish tetrahydroisoquinoline **7n** (10 mg, 26%) as a colourless oil.

The title compound could not be prepared in the absence of a rhodium catalyst.

HRMS (ESI): Exact mass calculated for $C_{22}H_{27}N$ $[M+H]^+$: 306.2216, found: 306.2218.

^{1}H NMR (400 MHz, $CDCl_3$) δ 7.44 – 7.23 (m, 5H, 5 x C^{Ar}**H**), 7.21 – 7.05 (m, 3H, 3 x C^{Ar}**H**), 7.02 – 6.92 (m, 1H, C^{Ar}**H**), 3.77 – 3.66 (m, 2H, C^{1}**H$_2$** + C^{11}**H$_2$**), 3.58 (d, *J* = 13.0 Hz, 1H, C^{1}**H$_2$**/C^{11}**H$_2$**), 3.46 (d, *J* = 14.9 Hz, 1H, C^{1}**H$_2$**/C^{11}**H$_2$**), 2.80 (dd, *J* = 11.3, 4.8 Hz, 1H, C^{3}**H$_2$**), 2.67 (td, *J* = 5.15, 5.14 Hz, 1H, C^{4}**H**), 2.57 (dd, *J* = 11.3, 4.7 Hz, 1H, C^{3}**H$_2$**), 1.91 – 1.78 (m, 1H, C^{16}**H**), 1.78 – 1.69 (m, 1H, C^{17}**H$_2$**/C^{18}**H$_2$**/C^{19}**H$_2$**), 1.69 – 1.57 (m, 3H, C^{17}**H$_2$**/C^{18}**H$_2$**/C^{19}**H$_2$**), 1.51 – 1.39 (m, 1H, C^{17}**H$_2$**/C^{18}**H$_2$**/C^{19}**H$_2$**), 1.33 – 1.17 (m, 1H, C^{17}**H$_2$**/C^{18}**H$_2$**/C^{19}**H$_2$**), 1.17 – 0.96 (m, 4H, C^{17}**H$_2$**/C^{18}**H$_2$**/C^{19}**H$_2$**).

^{13}C NMR (101 MHz, $CDCl_3$) δ 137.7 (C^{12}), 129.3 (2 x C^{13}H/C^{14}H), 128.9 (C^{15}H), 128.3 (2 x C^{13}H/C^{14}H), 127.2 (C^{5}H/C^{6}H/C^{7}H/C^{8}H), 126.5 (C^{5}H/C^{6}H/C^{7}H/C^{8}H), 125.7 (C^{5}H/C^{6}H/C^{7}H/C^{8}H), 125.6 (C^{5}H/C^{6}H/C^{7}H/C^{8}H), 63.2 (C^{1}H$_2$/C^{11}H$_2$), 56.8 (C^{1}H$_2$/C^{11}H$_2$), 52.5 (C^{3}H), 44.0 (C^{4}H), 42.4 (C^{16}H), 32.0 (C^{Cy}H$_2$), 29.7 (C^{Cy}H$_2$), 27.2 (C^{Cy}H$_2$), 27.1 (C^{Cy}H$_2$), 26.8 (C^{Cy}H$_2$), C^{9} and C^{10} not observed.

IR (neat) (cm^{-1}) 2980, 2920, 2850, 2795, 2754, 1494, 1451, 1368, 1256, 1146.

Annulation reactions

N-Benzyl-3-methyl-1,2,3,4,4a,5,6,10b-octahydrophenanthridine (8a)

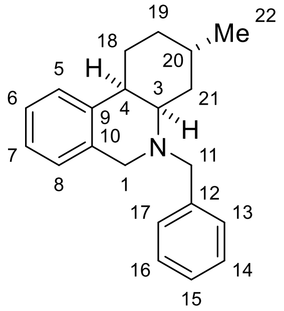

The title compound was prepared according to General Procedure **F** using isoquinolinium salt **4f** (45 mg, 0.125 mmol), methyl vinyl ketone (10 µL, 0.125 mmol), 5:2 HCO_2H:Et_3N (42 µL, 0.50 mmol), $[RhCp^*Cl]_2$ (7.7 µg, 0.0125 µmol, 0.01 mol%) in MeCN (0.1 mL) and was purified by flash column chromatography (1-2% EtOAc in pentane) to furnish the annulated tetrahydroisoquinoline **8a** (20 mg, 55%) as a single diastereomer as a colourless oil. A repetition on a 0.625 mmol scale furnished the product **8a** in 53% isolated yield.

The title compound could not be prepared according to General procedure **E**.

HRMS (ESI): Exact mass calculated for $C_{21}H_{26}N$ $[M+H]^+$: 292.2060, found: 292.2054.

^{1}H NMR (400 MHz, $CDCl_3$) 7.52 – 7.46 (m, 2H, 2 x C^{Ar}**H**), 7.43 – 7.35 (m, 2H, 2 x C^{Ar}**H**), 7.35 – 7.29 (m, 1H, C^{Ar}**H**), 7.21 – 7.07 (m, 3H, 3 x C^{Ar}**H**), 6.92 (d, J = 7.5 Hz, 1H, C^{Ar}**H**), 4.39 (d, J = 13.0 Hz, 1H, C^1**H₂**), 3.86 (d, J = 15.4 Hz, 1H, C^{11}**H₂**), 3.29 (d, J = 14.7 Hz, 1H, C^{11}**H₂**), 3.12 (d, J = 13.0 Hz, 1H, C^1**H₂**), 2.89 (d, J = 3.2 Hz, 1H, C^3**H**), 2.73 (dt, J = 11.9, 3.5 Hz, 1H, C^4**H**), 2.36 – 2.26 (m, 1H, C^{21}**H₂**), 2.26 – 2.15 (m, 1H, C^{18}**H₂**), 1.98 – 1.85 (m, 2H, C^{19}**H** + C^{20}**H₂**), 1.80 (dq, J = 13.3, 3.6 Hz, 1H, C^{18}**H₂**), 1.35 (ddd, J = 14.8, 11.9, 3.1 Hz, 1H, C^{21}**H₂**), 1.31 – 1.16 (m, 1H, C^{19}**H₂**), 0.97 (d, J = 6.5 Hz, 3H, C^{22}**H₃**);

^{13}C NMR (101 MHz, $CDCl_3$) δ 140.8 (C^{Ar}), 140.4 (C^{Ar}), 134.6 (C^{Ar}), 128.7 (2 x $C^{Ar}H$), 128.4 (2 x $C^{Ar}H$), 128.1 ($C^{Ar}H$), 126.9 ($C^{Ar}H$), 126.1 ($C^{Ar}H$), 126.0 ($C^{Ar}H$), 125.6 ($C^{Ar}H$), 58.3 (C^3H), 56.8 (C^1H_2), 56.0 ($C^{11}H_2$), 42.5 (C^4H), 37.5 ($C^{21}H_2$), 35.6 ($C^{19}H_2$), 32.4 ($C^{18}H_2$), 26.0 ($C^{20}H$), 22.7 ($C^{22}H$);

IR (neat) (cm^{-1}):2946, 2922, 2786, 1379, 1369, 1155, 1069, 966, 744, 699.

N-Benzyl-3-methyl-1,2,3,4,4a,5,6,10b-octahydrophenanthridin-5-ium chloride (8a·HCl)

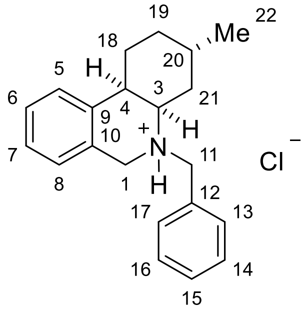

The hydrochloride of **8a** was formed by dissolving **8a** (94 mg, 0.32 mmol) in degassed Et_2O (3 mL) and adding 2M HCl in diethyl ether (0.18 mL, 0.35 mmol) dropwise at 0 °C. After stirring at 0° C for 20 min the reaction mixture was filtered to give **8a·HCl** (100 mg, 85%) as a colourless solid.

m.p. (CH_2Cl_2): 176 °C.

HRMS (ESI): Exact mass calculated for $C_{21}H_{27}N$ $[M]^+$: 292.2060, found: 292.2061.

^{1}H NMR (400 MHz, $CDCl_3$, mixture of diastereomers 60:40) δ 12.82 (s, 1H), 12.21 (s, 1H), 7.93 – 7.86 (m, 2H), 7.69 – 7.64 (m, 3H), 7.43 (m, 7H), 7.34 – 7.27 (m, 2H), 7.25 – 7.14 (m, 4H), 7.04 (d, J = 7.0 Hz, 1H), 6.99 (dd, J = 7.8, 1.3 Hz, 1H), 4.59 (dd, J = 16.0, 4.2 Hz, 2H), 4.48 (dd, J = 13.6, 3.9 Hz, 2H), 4.46 – 4.38 (m, 2H), 4.30 (dd, J = 13.5, 5.7 Hz, 1H), 4.13 (dd, J = 12.7, 7.0 Hz, 1H), 3.97 (dd, J = 15.8, 5.2 Hz,

4H), 3.07 (dt, *J* = 9.1, 4.5 Hz, 1H), 2.40 (dtd, *J* = 14.2, 9.0, 3.4 Hz, 1H), 2.28 – 2.01 (m, 3H), 1.91 – 1.70 (m, 5H), 1.65 (ddd, *J* = 12.4, 4.8 Hz, 1H), 1.42 (dddd, *J* = 13.8, 4.1 Hz, 1H), 1.24 – 1.11 (m, 3H), 0.94 (d, *J* = 6.8 Hz, 4H), 0.86 (d, *J* = 7.3 Hz, 3H).

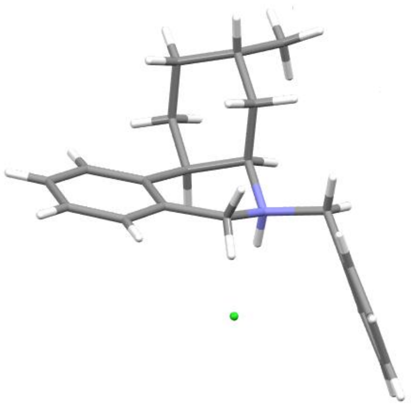

Crystal data $C_{21}H_{26}ClN$, M = 327.90, orthorombic, a = 11.29240(10), b = 13.6629(2), c = 23.2273(2) Å, β = 90°, Z = 8, T = 150 K, space group *Pna2₁*, 43420 reflections measured, 6973 unique (R_{int} = 0.027), which were used in all calculations.

N-Benzyl-3-phenyl-1,2,3,4,4a,5,6,10b-octahydrophenanthridine (8b)

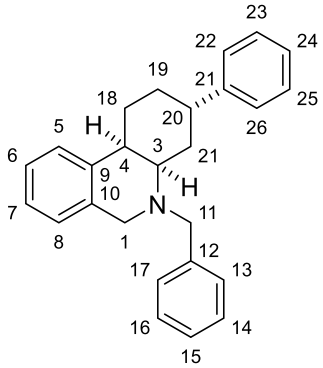

The title compound was prepared according to General Procedure **F** using isoquinolinium salt **4f** (45 mg, 0.125 mmol), phenyl vinyl ketone (17 mg, 0.125 mmol), 5:2 $HCO_2H:Et_3N$ (42 μL, 0.50 mmol), $[RhCp^*Cl]_2$ (7.7 μg, 0.0125 μmol, 0.01 mol%) in MeCN (0.1 mL) and was purified by column chromatography (0.75-1% EtOAc in pentane) to furnish the annulated tetrahydroisoquinoline **8b** (14 mg, 32%, d.r. > 25:1) as a colourless solid. The title compound could not be prepared according to General procedure **E**.

m.p. (CH_2Cl_2): 119 °C.

HRMS (ESI): Exact mass calculated for $C_{26}H_{28}N$+ $[M+H]^+$: 354.2216, found: 354.2217.

^{1}H NMR (400 MHz, $CDCl_3$) δ 7.51 – 7.45 (m, 2H, 2 x C^{Ar}**H**), 7.40 – 7.27 (m, 5H, 5 x C^{Ar}**H**), 7.25 – 7.06 (m, 6H, 6 x C^{Ar}**H**), 6.94 – 6.87 (m, 1H, C^{Ar}**H**), 4.43 (d, *J* = 13.0 Hz, 1H, C^{1}**H₂**), 3.86 (d, *J* = 15.4 Hz, 1H, C^{11}**H₂**), 3.28 (d, *J* = 15.4 Hz, 1H, C^{11}**H₂**), 3.10 (d, *J* = 13.0 Hz, 1H, C^{1}**H₂**), 3.04 (dt, *J* = 12.5, 3.2 Hz, 1H, C^{20}**H**), 2.97 (q, *J* = 3.1 Hz, 1H, C^{3}**H**), 2.82 (dt, *J* = 12.1, 3.5 Hz, 1H, C^{4}**H**), 2.51 (dq, *J* = 14.6, 2.8 Hz, 1H, C^{21}**H₂**), 2.38 – 2.24 (m, 1H, C^{18}**H₂**), 2.12 – 2.01 (m, 1H, C^{19}**H₂**), 1.94 – 1.70 (m, 2H, C^{21}**H₂** + C^{18}**H₂**), 1.81 – 1.70 (m, 1H, C^{19}**H₂**);

^{13}C NMR (101 MHz, $CDCl_3$) δ 147.2 (C^{Ar}), 140.5 (C^{Ar}), 140.2 (C^{Ar}), 134.5 (C^{Ar}), 128.8 (2 x C^{Ar}H), 128.6 (4x C^{Ar}H), 128.2 (C^{Ar}H), 127.0 (C^{Ar}H), 127.0 (2 x C^{Ar}H), 126.2 (C^{Ar}H), 126.2 (C^{Ar}H), 126.1 (C^{Ar}H), 125.8 (C^{Ar}H), 58.6 (C^{3}H), 56.9 ($C^{1}H_2$), 56.2 ($C^{11}H_2$), 42.5 (C^{4}H), 37.4 (C^{20}H), 36.7 ($C^{21}H_2$), 34.8 ($C^{19}H_2$), 33.0 ($C^{18}H_2$);

IR (neat) (cm^{-1}): 2926, 2786, 1138, 1111, 1071, 1028, 957, 745, 698.

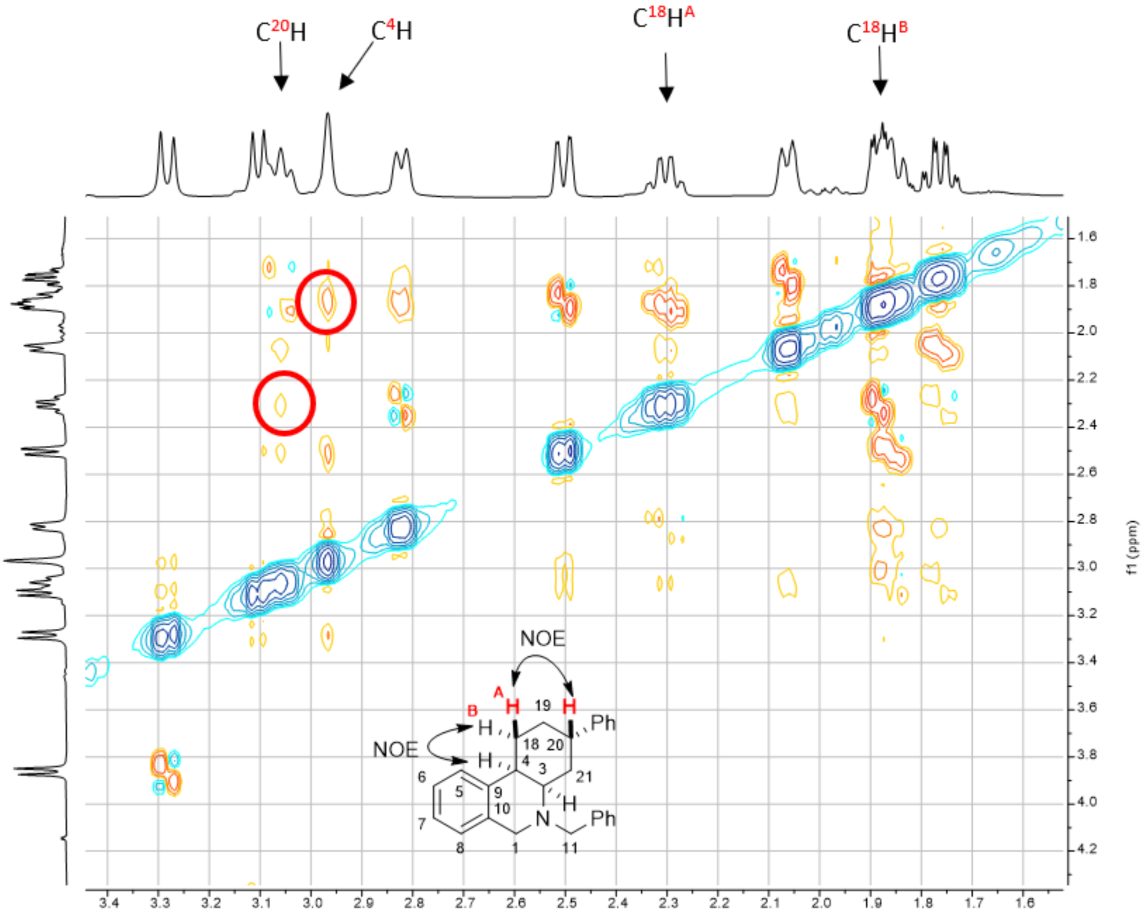

2-(*N*-Benzyl-1,2,3,4,4a,5,6,10b-octahydrophenanthridin-3-yl)-1-phenylethan-1-one (8c)

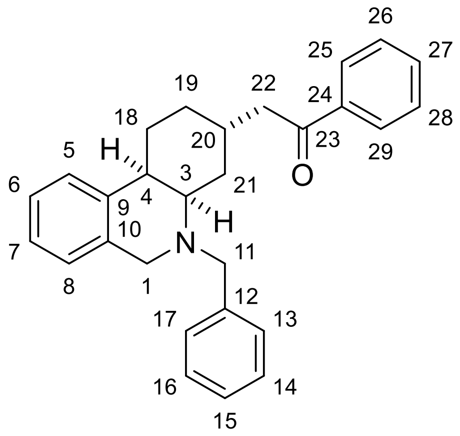

The title compound was prepared according to General Procedure **E** using isoquinolinium salt **4f** (45 mg, 0.125 mmol), (*E*)-1-phenylhexa-3,5-dien-1-one[13] (20 mg, 0.125 mmol), 5:2 HCO_2H:Et_3N (42 µL, 0.50 mmol) in MeCN (0.1 mL) and was purified by column chromatography (2-3% EtOAc in pentane) to furnish the annulated tetrahydroisoquinoline **8c** (11 mg, 22%, d.r. > 25:1) as a colourless solid.

m.p. (CH_2Cl_2): 121 °C;

HRMS (ESI): Exact mass calculated for $C_{28}H_{30}NO$ $[M+H]^+$: 396.2322, found: 396.2321.

^{1}H NMR (600 MHz, $CDCl_3$) δ 7.94 – 7.91 (m, 2H, C^{25}**H** + C^{29}**H**), 7.49 (t, *J* = 7.4 Hz, 1H, C^{27}**H**), 7.43 – 7.35 (m, 4H, 4 x C^{Ar}**H**), 7.29 (t, *J* = 7.5 Hz, 2H, 2 x C^{Ar}**H**), 7.24 (t, *J* = 7.3 Hz, 1H, C^{Ar}**H**), 7.13 – 7.02 (m, 3H, 3 x C^{Ar}**H**), 6.86 (d, *J* = 7.6 Hz, 1H, C^{Ar}**H**), 4.44 (d, *J* = 12.7 Hz, 1H, C^1**H$_2$**), 3.78 (d, *J* = 15.4 Hz, 1H C^{12}**H$_2$**), 3.21

[13] L. Bernardi, J. López-Cantarero, B. Niess, K. A. Jørgensen, *J. Am. Chem. Soc.* **2007**, *129*, 5772–5778.

(d, J = 15.4 Hz, 1H, $C^{12}\mathbf{H_2}$), 3.01 (d, J = 12.7 Hz, 1H, $C^{1}\mathbf{H_2}$), 2.94 (dd, J = 16.2, 5.5 Hz, 1H, $C^{22}\mathbf{H_2}$), 2.87 – 2.82 (m, 2H, $C^{22}\mathbf{H_2}$ + $C^{3}\mathbf{H}$), 2.74 – 2.67 (m, 1H, $C^{4}\mathbf{H}$), 2.59 – 2.49 (m, 1H, $C^{20}\mathbf{H}$), 2.43 (d, J = 13.5 Hz, 1H, $C^{21}\mathbf{H_2}$), 2.19 (qd, J = 13.0, 3.8 Hz, 1H, $C^{18}\mathbf{H_2}$), 1.96 – 1.89 (m, 1H, $C^{19}\mathbf{H_2}$), 1.80 – 1.73 (m, 1H, $C^{18}\mathbf{H_2}$), 1.40 – 1.31 (m, 2H, $C^{21}\mathbf{H_2}$ + $C^{19}\mathbf{H_2}$).;

^{13}C NMR (151 MHz, $CDCl_3$) δ 200.0 (C^{23}), 140.4 (C^{Ar}), 140.2 (C^{Ar}), 137.4 (C^{Ar}), 134.6 (C^{Ar}), 133.0 (C^{27}), 129.0 (2 x C^{Ar}H), 128.7 (2 x C^{Ar}H), 128.3 (2 x C^{Ar}H), 128.1 (2 x C^{Ar}H), 128.0 (C^{Ar}H), 126.8 (C^{Ar}H), 126.2 (C^{Ar}H), 126.0 (C^{Ar}H), 125.7 (C^{Ar}H), 58.0 (C^{3}H), 56.8 ($C^{1}H_2$), 55.9 ($C^{11}H_2$), 46.0 ($C^{22}H_2$), 42.6 (C^{4}H), 35.3 ($C^{21}H_2$), 33.7 ($C^{19}H_2$), 32.2 ($C^{18}H_2$), 27.9 (C^{20}H);

IR (neat) (cm^{-1}): 2926, 2787, 1683, 1597, 1448, 1367, 1316, 1068, 1001, 910, 748, 699.

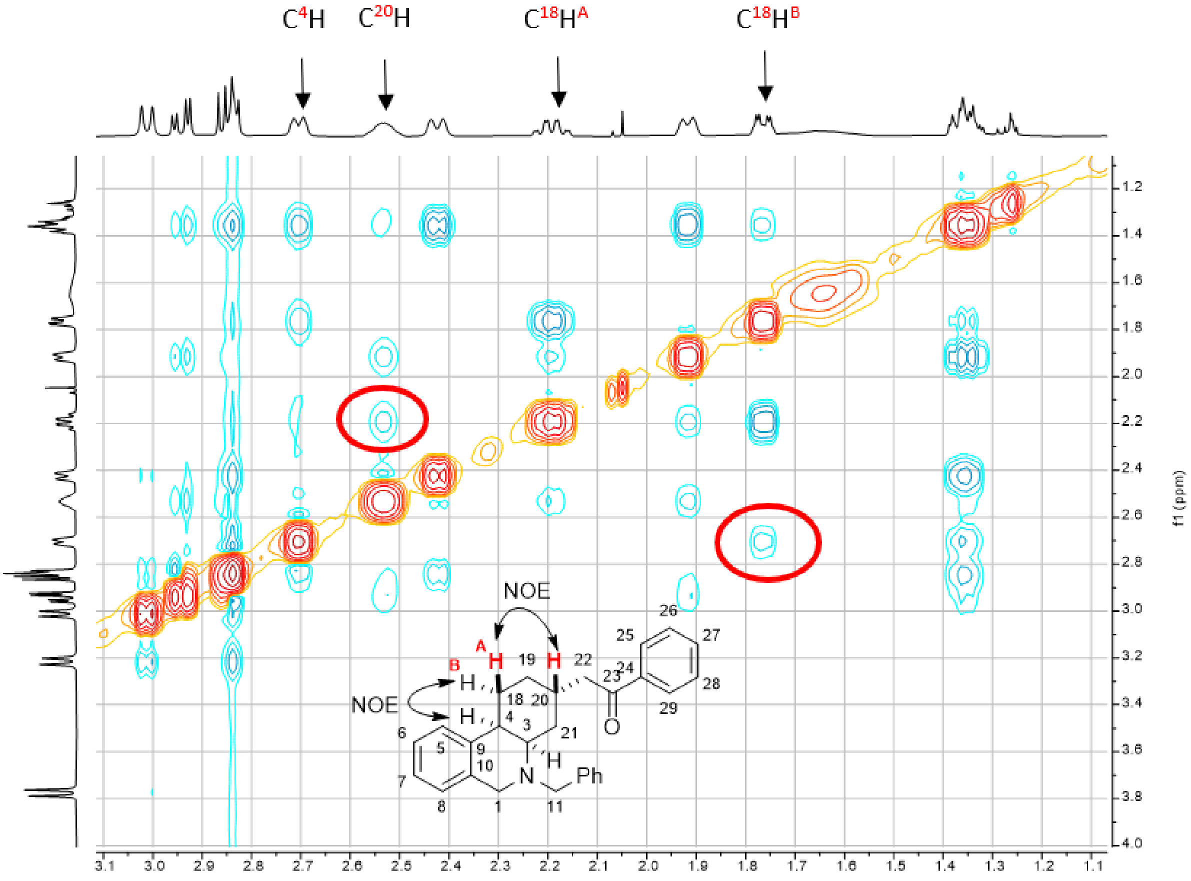

(*N*-Benzyl-10b-methyl-1,2,4a,5,6,10b-hexahydrophenanthridin-4-yl)(phenyl)methanone (8d)

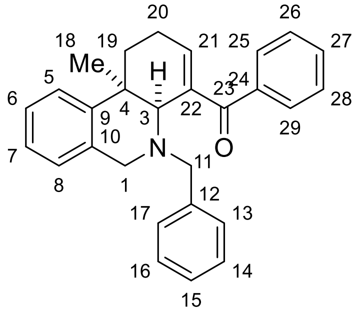

The title compound was prepared according to General Procedure **E** using isoquinolinium salt **4a** (45 mg, 0.125 mmol), (*E*)-1-phenylhexa-3,5-dien-1-one[13] (40 mg, 0.250 mmol), 5:2 HCO_2H:Et_3N (42 µL, 0.500 mmol) in MeCN (0.1 mL) and was purified by flash column chromatography (1-2% EtOAc in pentane) to furnish the annulated tetrahydroisoquinoline **8d** (27 mg, 55%, d.r. > 25:1) as a colourless oil. The product was found to be the *cis*-diastereomer (confirmed by NOESY).

HRMS (ESI): Exact mass calculated for $C_{28}H_{28}NO$ $[M+H]^+$: 394.2165, found: 394.2165.

^{1}H NMR (500 MHz, $CDCl_3$) δ 7.85 – 7.76 (m, 2H, 2 x C^{Ar}**H**), 7.61 – 7.53 (m, 1H, C^{Ar}**H**), 7.45 (t, *J* = 7.7 Hz, 2H, 2 x C^{Ar}**H**), 7.39 (dd, *J* = 7.9, 1.3 Hz, 1H, 2 x C^{Ar}**H**), 7.34 – 7.20 (m, 6H, 6 x C^{Ar}**H**), 7.13 (td, *J* = 7.5, 1.3 Hz, 1H, C^{Ar}**H**), 6.93 (dd, *J* = 7.6, 1.4 Hz, 1H, C^{Ar}**H**), 6.70 (t, *J* = 3.8 Hz, 1H C2^1**H**), 4.09 (s, 1H, C^3**H**), 3.90 (d, *J* = 13.4 Hz, 1H, C^1**H₂**), 3.77 (d, *J* = 15.6 Hz, 1H, C^{11}**H₂**), 3.67 – 3.58 (m, 2H, C^1**H₂** + C^{11}**H₂**), 2.49 (ddd, *J* = 13.7, 8.7, 5.8 Hz, 1H, C^{19}**H₂**), 2.44 – 2.26 (m, 2H, C^{20}**H₂**), 1.82 – 1.75 (m, 1H, C^{19}**H₂**), 1.37 (s, 3H, C^{18}**H₃**);

^{13}C NMR (126 MHz, $CDCl_3$) δ 197.2 (C^{23}), 145.0 (C^{21}H), 143.5 (C^{Ar}), 140.6 (C^{Ar}), 139.4 (C^{Ar}), 137.7 (C^{Ar}), 134.4 (C^{Ar}), 132.3 (C^{Ar}H), 129.8 (2 x C^{Ar}H), 128.6 (2 x C^{Ar}H), 128.4 (2 x C^{Ar}H), 128.3 (2 x C^{Ar}H), 126.9 (C^{Ar}H), 126.6 (C^{Ar}H), 126.6 (C^{Ar}H), 126.4 (C^{Ar}H), 125.6 (C^{Ar}H), 62.4 (C^3H), 58.5 (C^1H_2), 53.7 ($C^{11}H_2$), 37.7 (C^4), 32.9 ($C^{19}H_2$), 25.6 (br s, $C^{18}H_3$), 24.5 ($C^{20}H_2$);

IR (neat) (cm^{-1}): 2926, 1649, 1597, 1446, 1355, 1365, 1310, 1268, 909, 758.

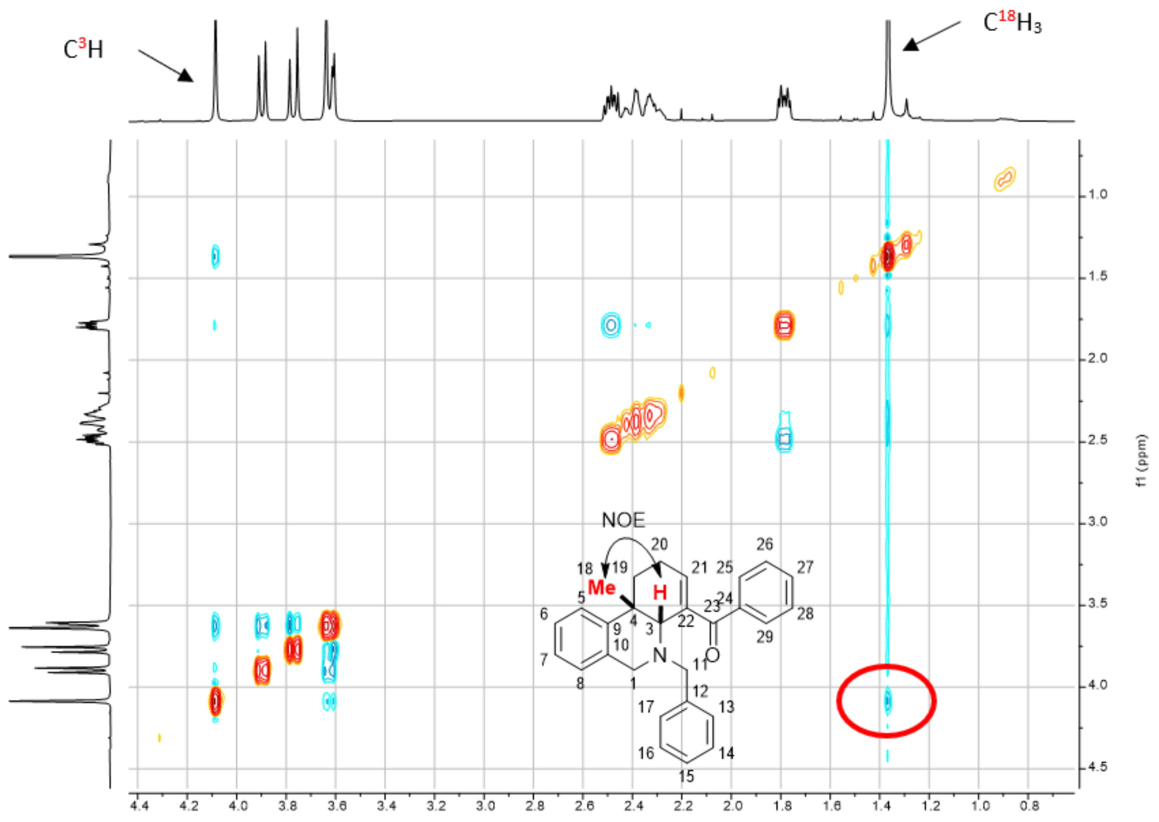

_N_-benzyl-2,2-dimethyl-1,2,3,3a,4,5-hexahydro-5,9b-(epiminomethano)cyclopenta[a]naphthalene (8e)

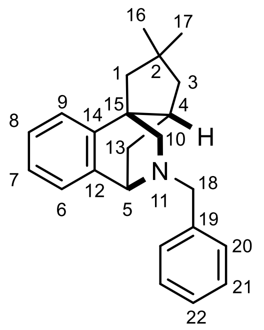

The title compound was prepared according to General Procedure **E** using isoquinolinium salt **4k** (80 mg, 0.23 mmol), 2,2-dimethylpent-4-enal (90%, 57 mg, 0.46 mmol) and 5:2 HCO_2H:Et_3N (72 μL, 0.92 mmol) in MeCN (0.2 mL) and was purified by column chromatography (1% acetone in CH_2Cl_2) to furnish tetracycle **8e** (33 mg, 45%, d.r. > 25:1) as an off-white solid. A single-crystal for X-ray analysis was grown by dissolving in $CHCl_3$/MTBE and slow evaporation. The single-crystal corresponded to the HI salt of **8e** and was obtained directly without the need for salt formation in an extra step.

m.p. (MTBE/$CHCl_3$): 86 – 87 °C.

HRMS (ESI): Exact mass calculated for $C_{23}H_{38}N$ $[M+H]^+$: 318.2216, found: 318.2216.

^{1}H NMR (400 MHz, $CDCl_3$) δ 7.40 (dd, J = 7.5, 1.3 Hz, 1H, C^9**H**), 7.38 – 7.27 (m, 5H, C^{Bn}**H** + C^8**H**), 7.28 – 7.21 (m, 2H, C^{Bn}**H** + C^7**H**), 7.13 (dd, J = 7.1, 1.5 Hz, 1H, C^6**H**), 3.69 (t, J = 2.7 Hz, 1H, C^5**H**), 3.39 (d, J = 13.3 Hz, 1H, C^{18}**H$_a$**), 3.23 (d, J = 13.3 Hz, 1H, C^{18}**H$_b$**), 3.07 (d, J = 9.0 Hz, 1H, C^{10}**H$_a$**), 2.45 – 2.37 (m, 1H, C^{13}**H$_a$**), 2.34 (d, J = 13.8 Hz, 1H, C^1**H$_a$**), 2.31 – 2.20 (m, 1H, C^4**H**), 1.90 (d, J = 9.0 Hz, 1H, C^{10}**H$_b$**), 1.44 (dd, J = 12.1, 5.8 Hz, 1H, C^3**H$_a$**), 1.35 (d, J = 13.9 Hz, 1H, C^1**H$_b$**), 1.18 (s, 3H, C^{16}**H$_3$**), 1.11 (s, 3H, C^{17}**H$_3$**), 1.00 (ddd, J = 12.0, 6.0, 1.7 Hz, 1H, C^{13}**H$_b$**), 0.62 (app t, J = 12.5 Hz, 1H, C^3**H$_b$**).

^{13}C NMR (101 MHz, $CDCl_3$) δ 141.9 (C^{14}), 139.6 (C^{12} + C^{19}), 129.0 (2 x $C^{20}H$), 128.3 (2 x $C^{21}H$), 127.0 ($C^{22}H$), 126.6 ($C^{8}H$), 125.8 ($C^{7}H$), 123.8 ($C^{6}H$), 123.2 ($C^{9}H$), 64.1 ($C^{10}H_2$), 61.5 ($C^{18}H_2$), 57.0 ($C^{5}H$), 48.8 (C^{15}), 47.9 ($C^{3}H_2$), 43.1 ($C^{1}H_2$), 40.2 ($C^{4}H$), 39.1 (C^{2}), 33.6 ($C^{13}H_2$), 33.1 ($C^{16}H_3$), 31.4 ($C^{17}H_3$).

IR (neat) (cm^{-1}): 3025, 2946, 2864, 1452, 1120, 1026, 909, 750, 728, 698.

Crystal data $C_{23}H_{28}NI$, M = 445.39, monoclinic, a = 9.2483(3), b = 14.3133(4), c = 16.1073(5) Å, β = 106.151(4)°, Z = 4, T = 150 K, space group $P2_1/n$, 14033 reflections measured, 4251 unique (R_{int} = 0.032), which were used in all calculations.

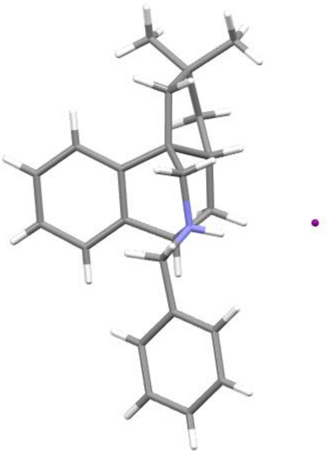

Relative stereochemistry could also be assigned from the following observed NOESY correlations:

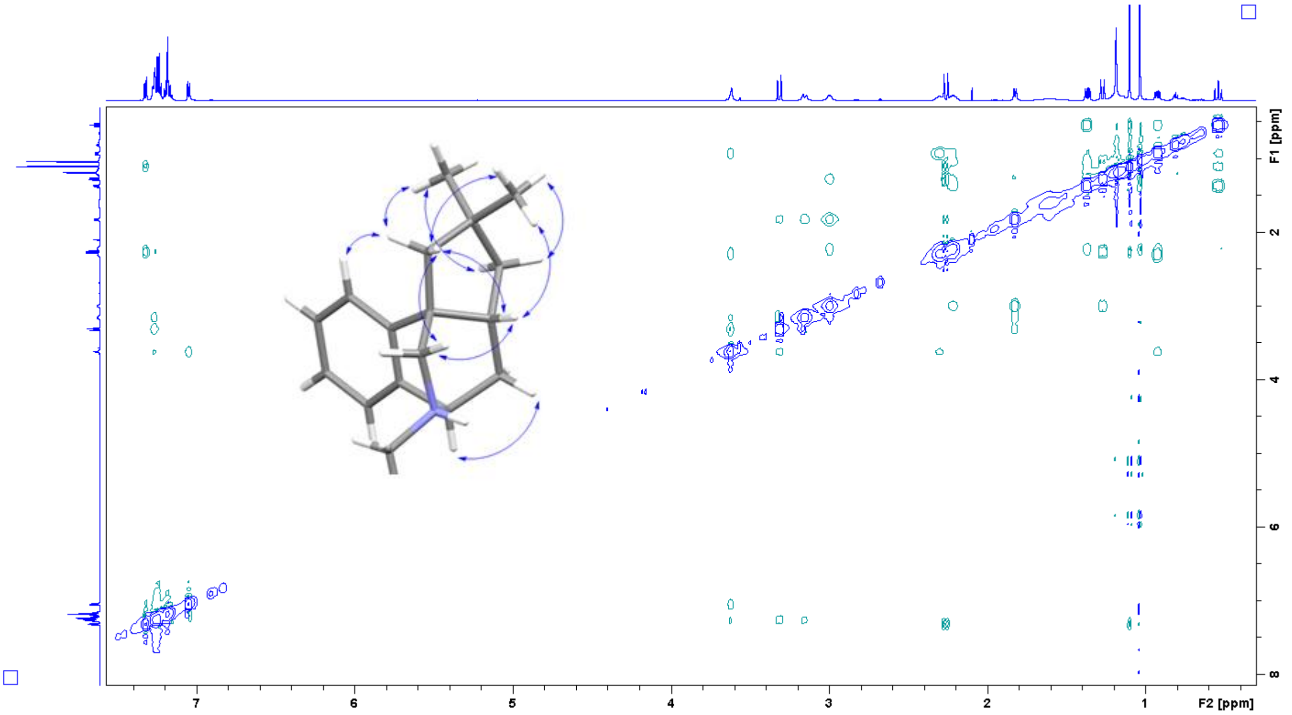

Investigation into reaction kinetics

General

Reactions were carried out in GC vials using 20 mg of *N*-Benzyl-3-methylquinolinium iodide (**1a**), and a "Mastermix", containing aliquots of 8.9 µL methyl vinyl ketone, 18.7 µL 5:2 formic acid-triethylamine complex and $[RhCp^*Cl_2]_2$ in acetonitrile (45 µL). The new vials (without magnetic stirring) were placed in an 80 °C oil bath and heated for the time indicated before quenching by dilution in CH_2Cl_2/0.1 M aqueous K_2CO_3. Crude mixtures obtained this way were analysed by quantitative ^{1}H-NMR using trimethoxybenzene (3.1 mg per $CDCl_3$ aliquot) as an internal standard. Methoxy peaks of TMB (6.09 ppm) and methyl peaks of the product **3a** (0.97 ppm) and reduced side-product **2a** (1.05 ppm) were chosen as diagnostic signals for quantification.

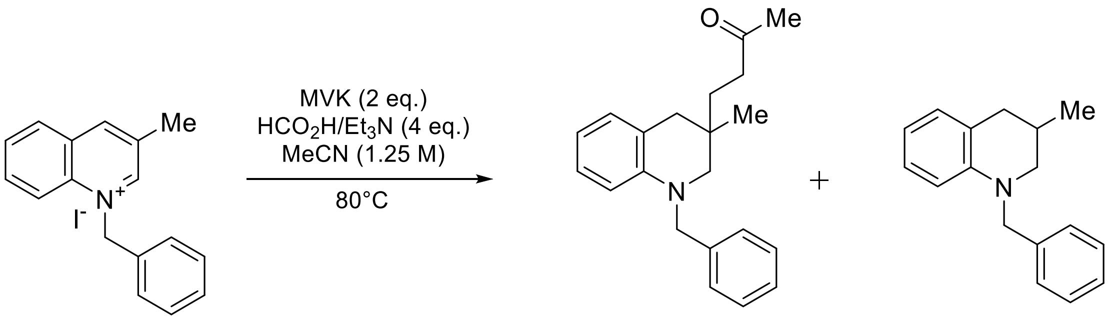

1a **2a** **3a**

Analysis of kinetic data

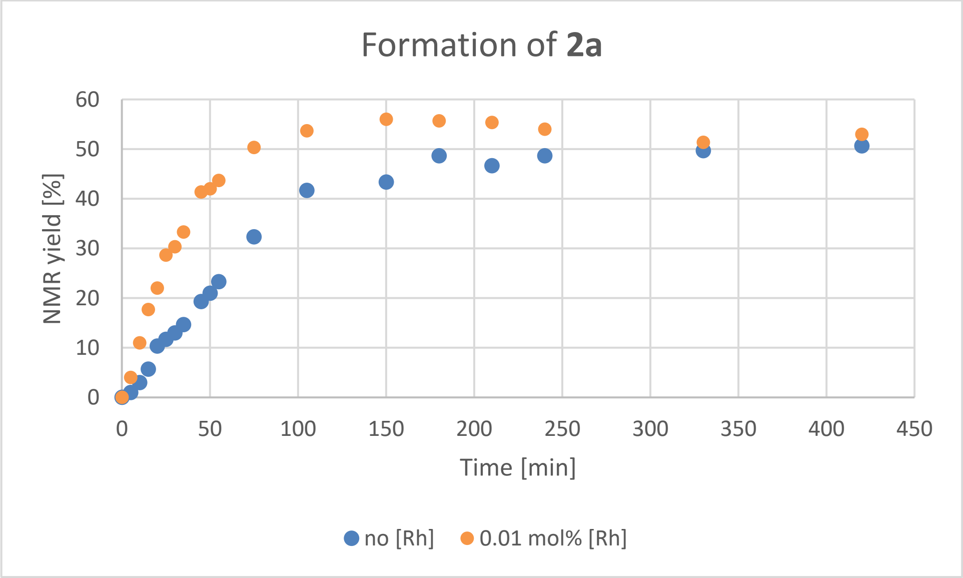

Figure 3: Difference in formation of **3a** in the presence of metal-catalyst

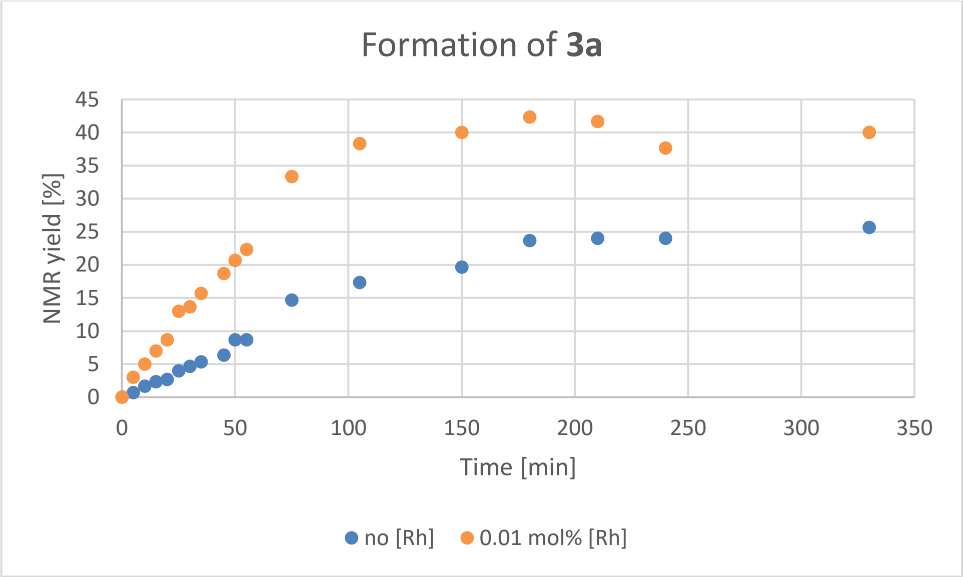

Figure 4: Difference in formation of **2a** in the presence of metal-catalyst

Deuterium labelling experiments

Formic-d acid, formic acid-d and formic acid-d_2 were purchased from Sigma Aldrich and used without further purification. All reactions were performed following General Procedure B or E, using deuterated reagents where specified in the reaction scheme.

Reaction with formic-d acid (DCO_2H):

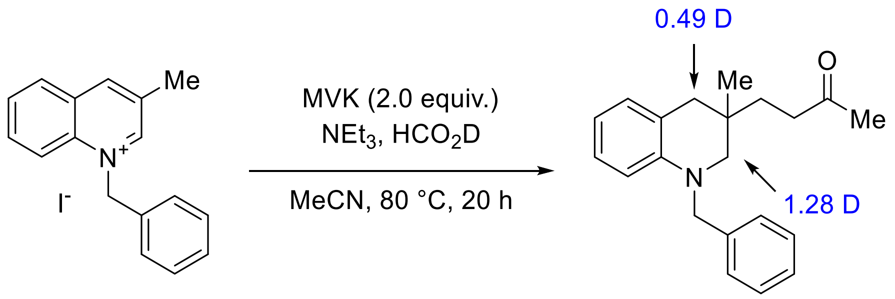

^{1}H NMR

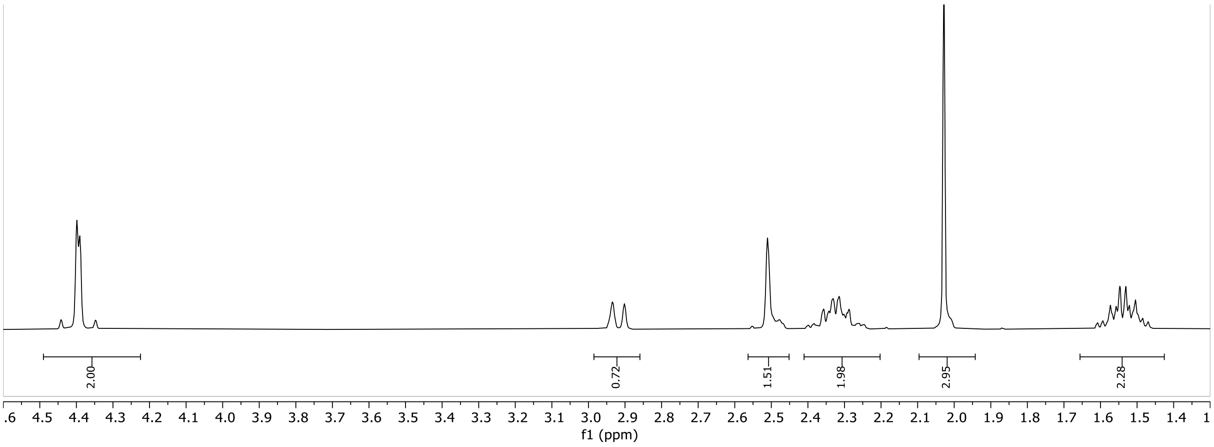

^{2}H NMR

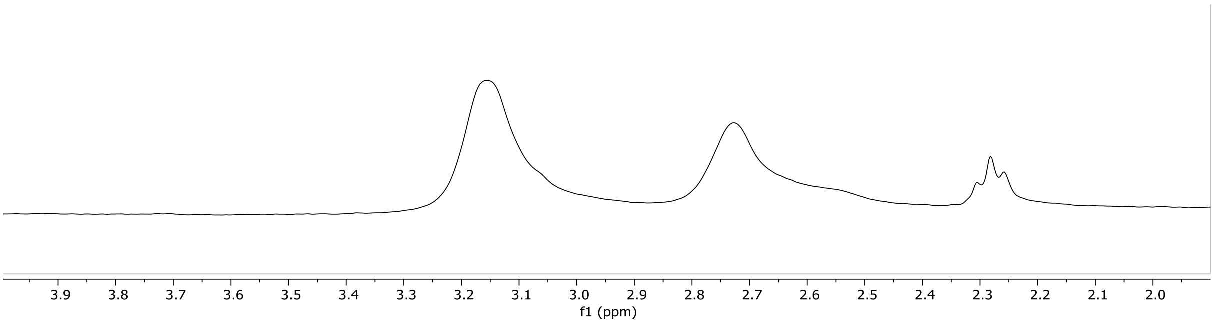

Reaction with formic acid-d (HCO_2D):

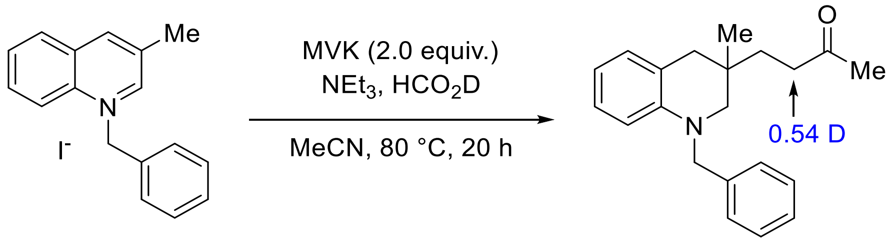

^{1}H NMR

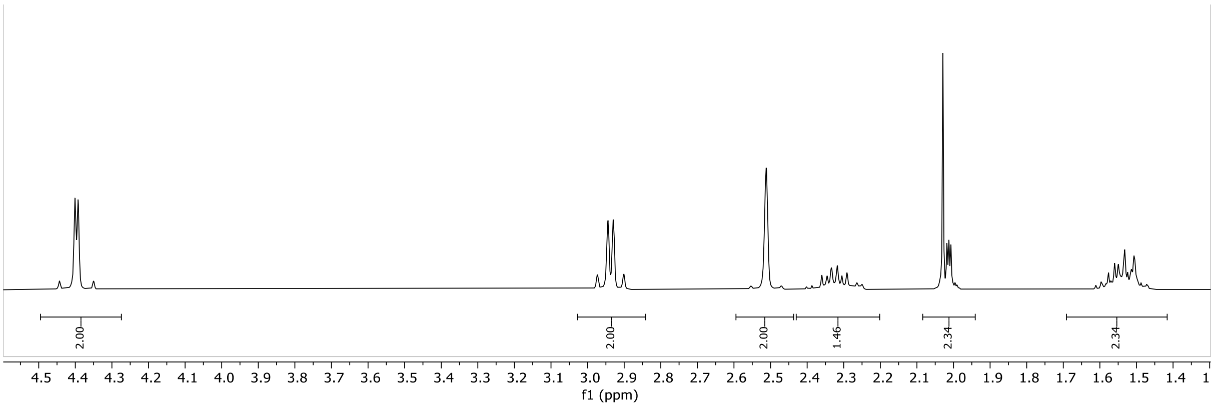

^{2}H NMR

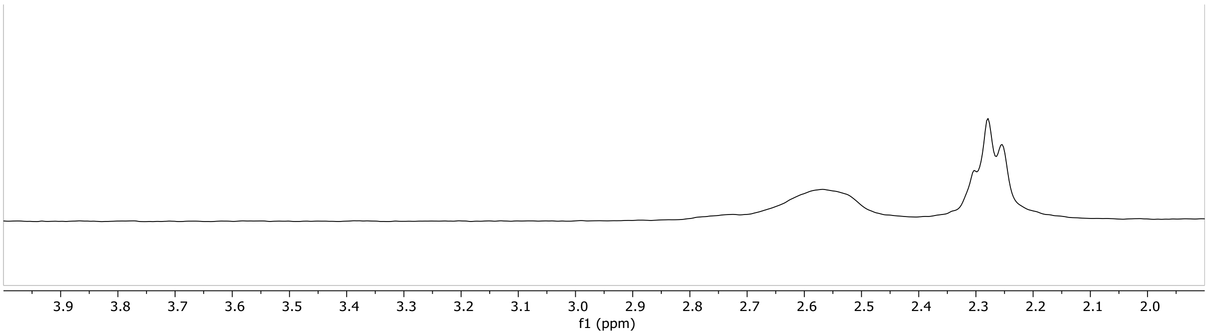

Reaction with formic acid-d$_2$ (DCO_2D):

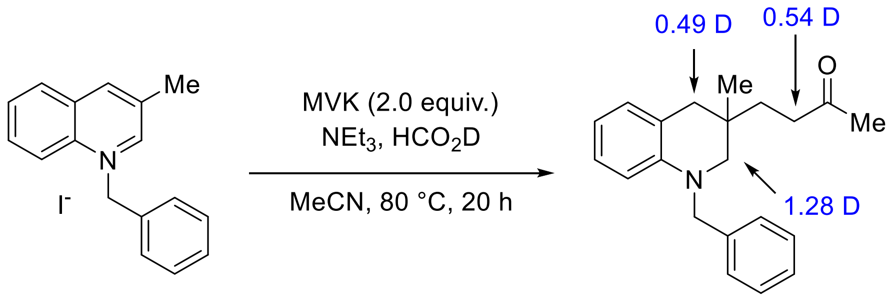

^{1}H NMR

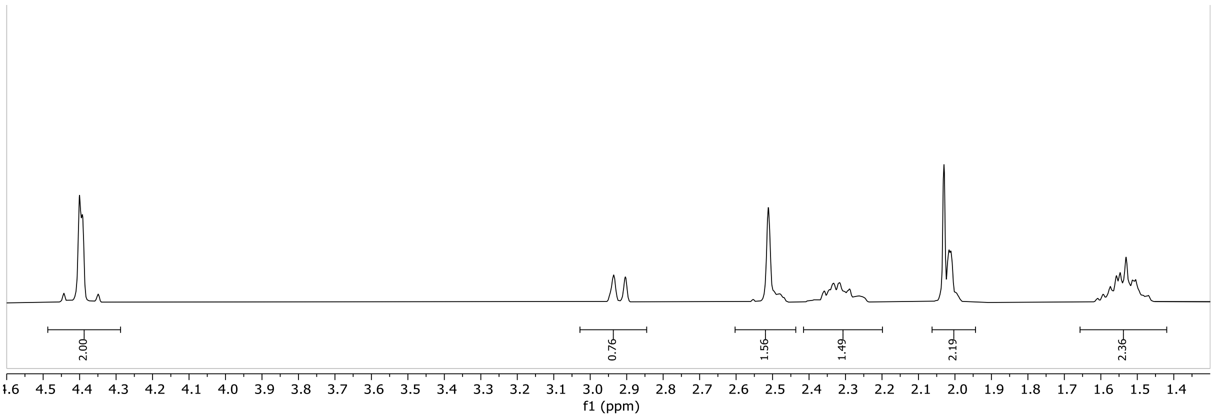

Reaction with formic-d acid (DCO_2H):

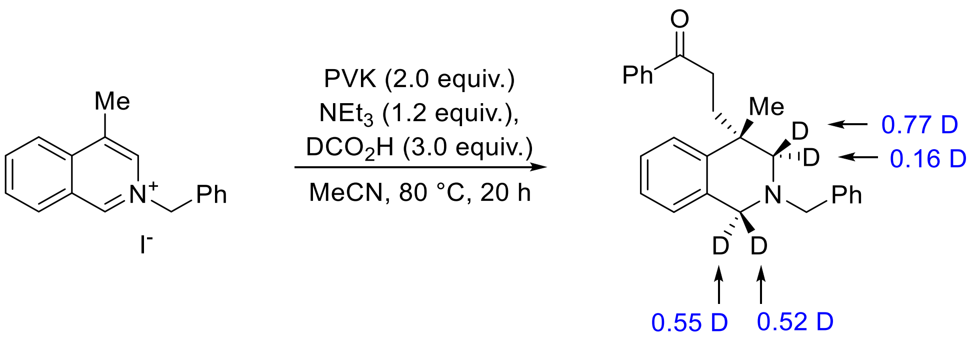

^{1}H NMR

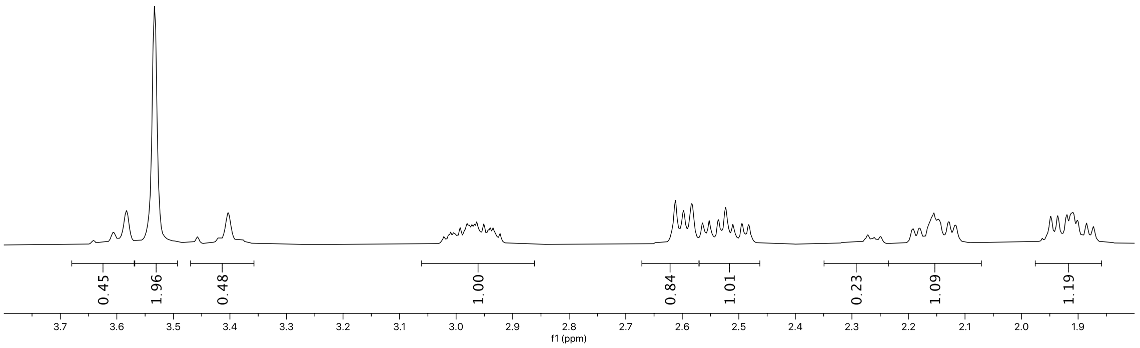

^2H NMR

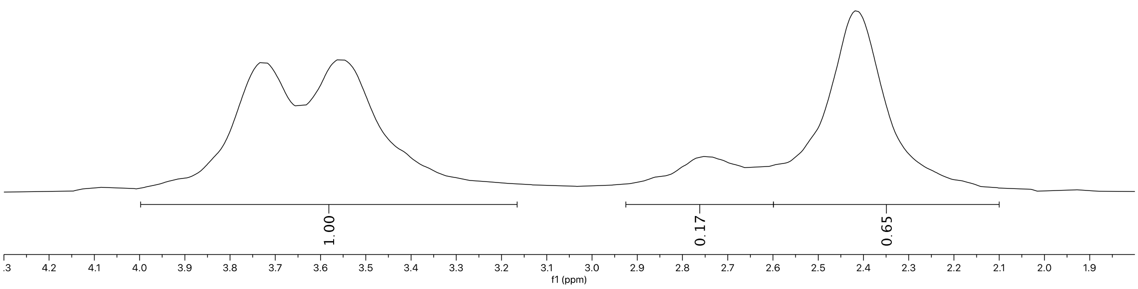

Reaction with formic acid-d (HCO_2D):

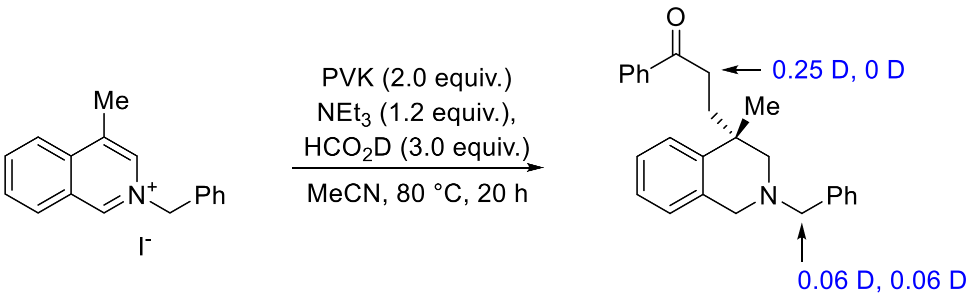

^1H NMR

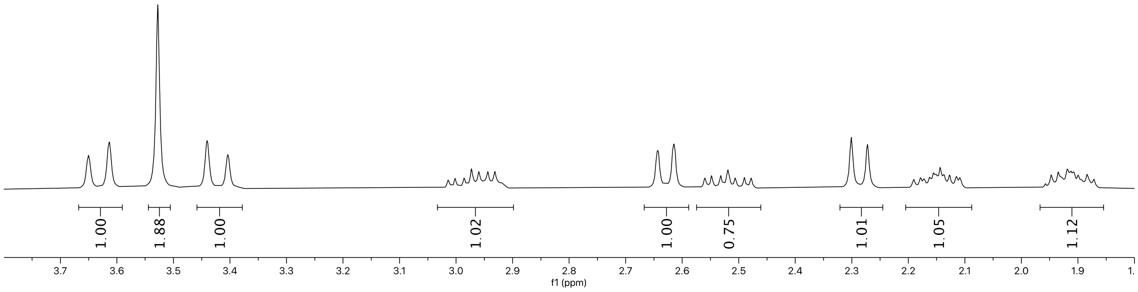

^2H NMR

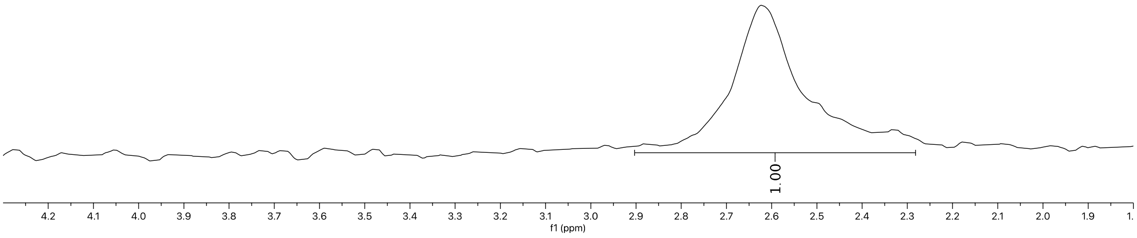

Reaction with formic acid-d (HCO_2D) and quench with 0.1 K_2CO_3 in D_2O:

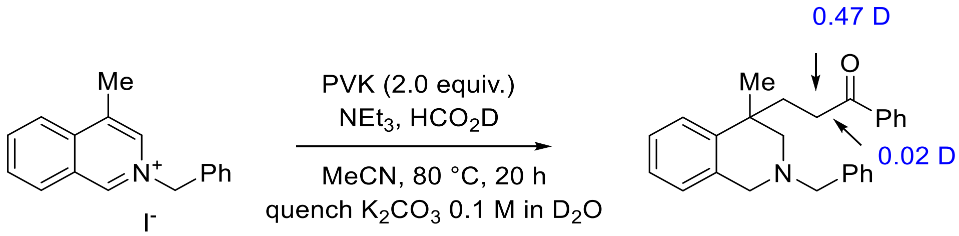

^{1}H NMR

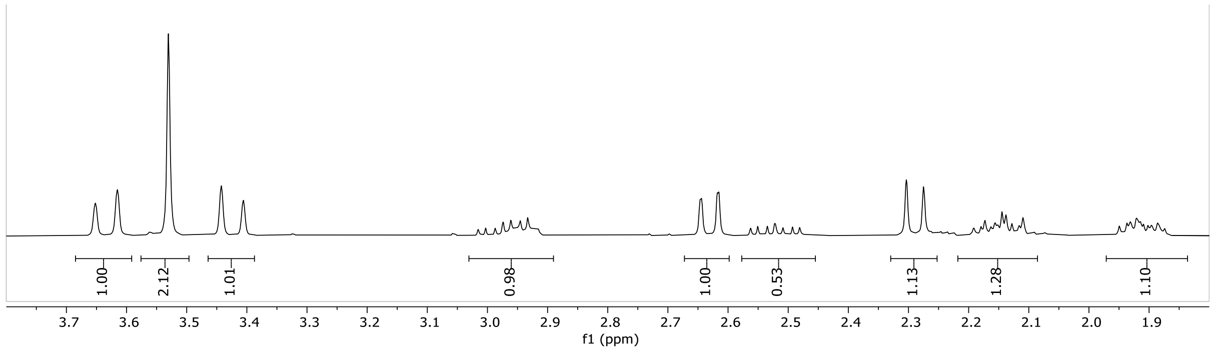

^{2}H NMR

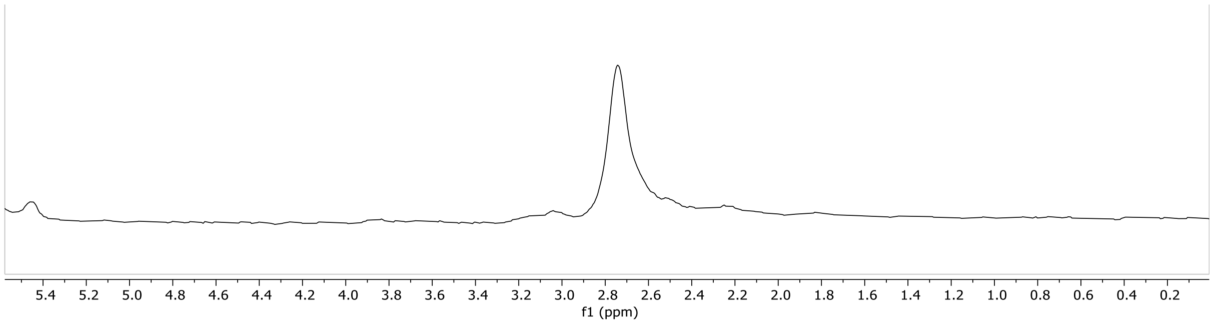

Reaction with formic acid-d_2 (DCO_2D):

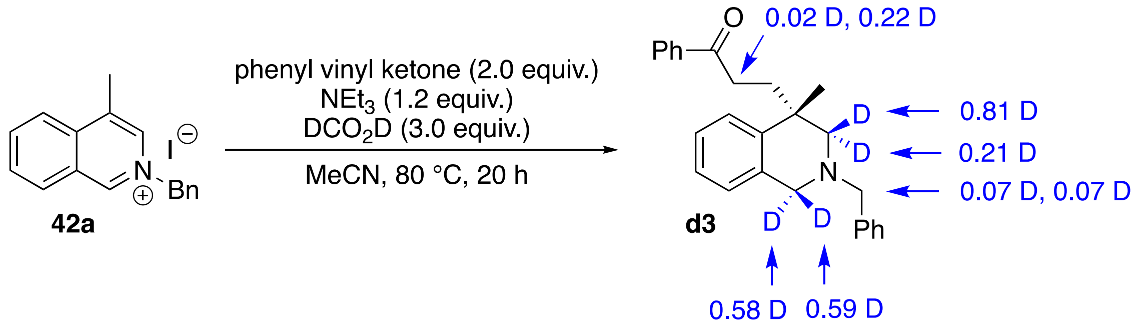

^{1}H NMR

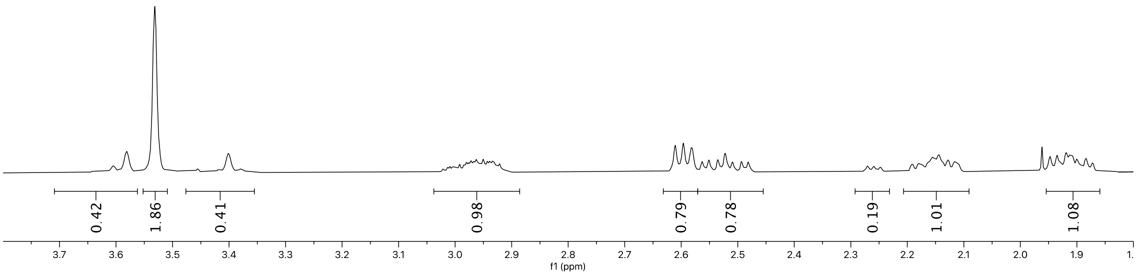

^{2}H NMR

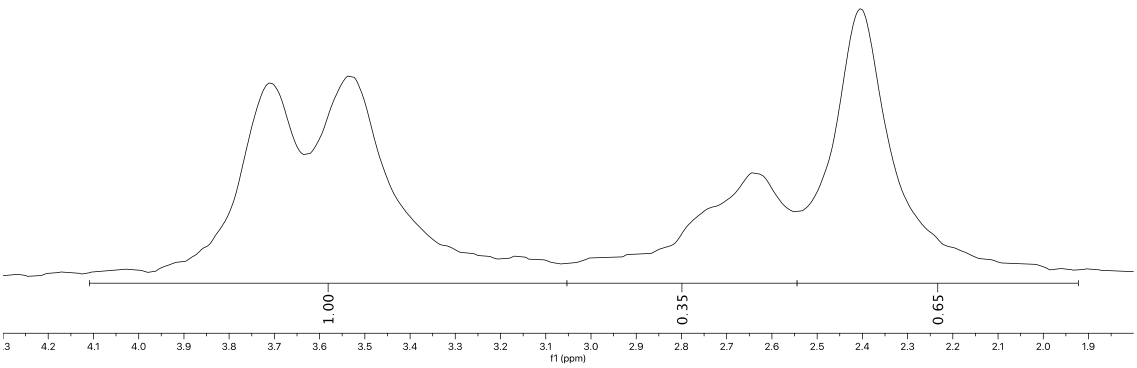

Proposed mechanism for quinolinium and isoquinolinium salts

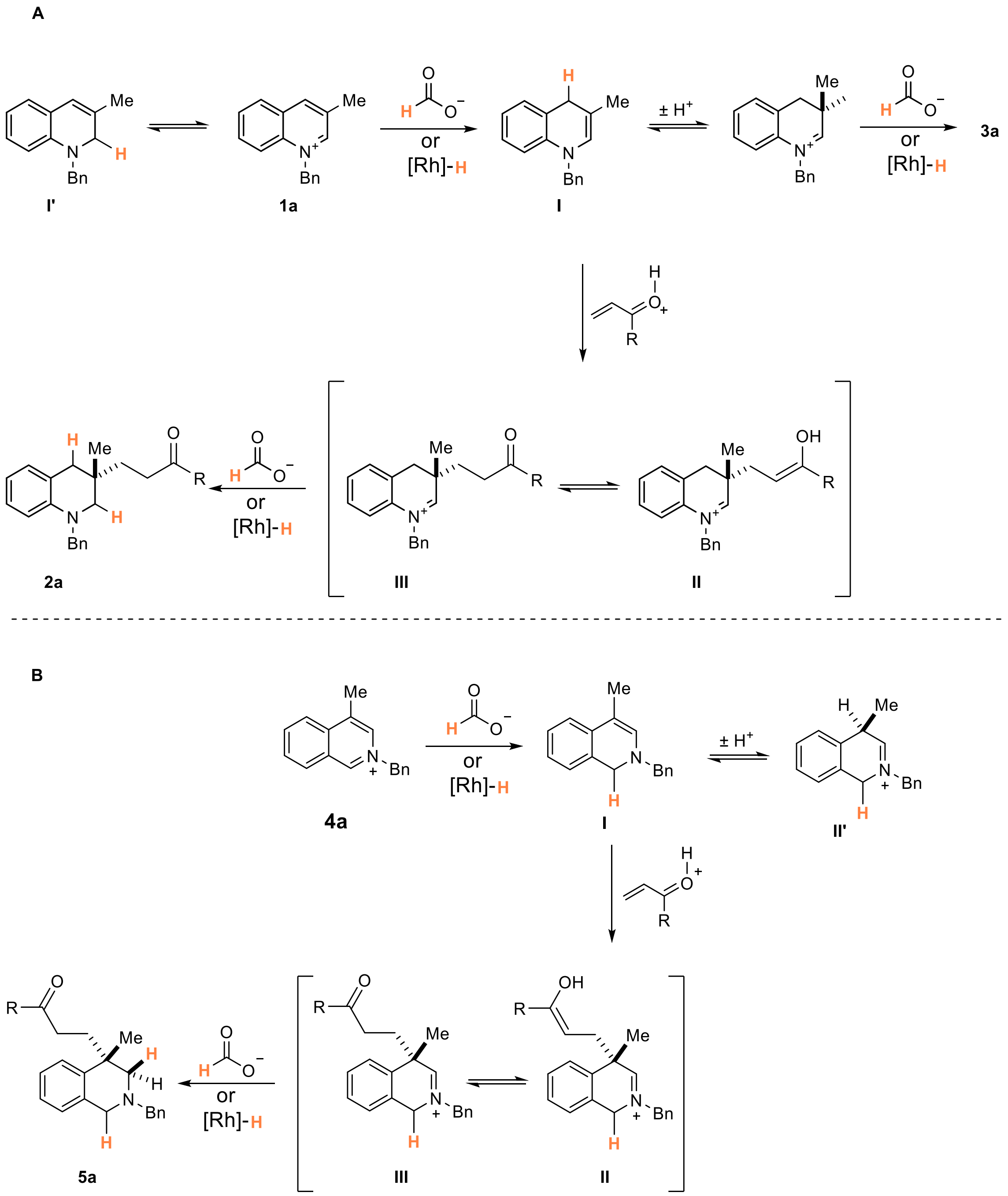

Figure 5: Mechanism of the reaction with activated quinoline (**A**) and isoquinolines (**B**)

Crystallographic data

Table 1. Crystal data and structure refinement for **2m**.

Empirical formula	C31 H38 Cl N O	
Formula weight	476.10	
Temperature	300 K	
Wavelength	1.54184 Å	
Crystal system	Triclinic	
Space group	P -1	
Unit cell dimensions	a = 9.2443(4) Å	α= 90.582(3)°.
	b = 16.6064(5) Å	β= 97.470(3)°.
	c = 18.4379(7) Å	γ = 104.885(3)°.
Volume	2709.49(18) $Å^3$	
Z	4	
Density (calculated)	1.167 Mg/m^3	
Absorption coefficient	1.407 mm^{-1}	
F(000)	1023.991	
Crystal size	0.11 x 0.08 x 0.05 mm^3	
Theta range for data collection	3.585 to 76.936°.	
Index ranges	-11<=h<=11, -15<=k<=20, -23<=l<=22	
Reflections collected	28854	
Independent reflections	11191 [R(int) = 0.049]	
Completeness to theta = 73.347°	99.8 %	
Absorption correction	Semi-empirical from equivalents	
Max. and min. transmission	0.93 and 0.73	
Refinement method	Full-matrix least-squares on F^2	
Data / restraints / parameters	11190 / 0 / 613	
Goodness-of-fit on F^2	1.0231	
Final R indices [I>2sigma(I)]	R1 = 0.0752, wR2 = 0.1996	
R indices (all data)	R1 = 0.1019, wR2 = 0.2419	
Largest diff. peak and hole	0.80 and -0.47 e.$Å^{-3}$	

Table 2. Crystal data and structure refinement for **5o.Hcl**.

Empirical formula	C31 H38 Cl N O	
Formula weight	476.10	
Temperature	35 K	
Wavelength	0.68890 Å	
Crystal system	Monoclinic	
Space group	P c	
Unit cell dimensions	a = 20.6290(8) Å	α= 90°.
	b = 7.2107(3) Å	β= 91.754(3)°.
	c = 17.2803(6) Å	γ = 90°.
Volume	2569.23(17) $Å^3$	
Z	4	
Density (calculated)	1.231 Mg/m^3	
Absorption coefficient	0.158 mm^{-1}	
F(000)	1024.001	
Crystal size	0.15 x 0.05 x 0.01 mm^3	
Theta range for data collection	1.915 to 23.684°.	
Index ranges	-29<=h<=26, -10<=k<=10, -24<=l<=24	
Reflections collected	24211	
Independent reflections	11639 [R(int) = 0.120]	
Completeness to theta = 25.058°	96.8 %	
Absorption correction	Semi-empirical from equivalents	
Max. and min. transmission	1.00 and 0.75	
Refinement method	Full-matrix least-squares on F^2	
Data / restraints / parameters	7483 / 355 / 666	
Goodness-of-fit on F^2	1.0081	
Final R indices [I>2sigma(I)]	R1 = 0.1266, wR2 = 0.3183	
R indices (all data)	R1 = 0.1357, wR2 = 0.3322	
Absolute structure parameter	-0.11(8)	
Largest diff. peak and hole	1.18 and -0.77 e.$Å^{-3}$	

Table 3. Crystal data and structure refinement for **8a**.

Empirical formula	C21 H26 Cl N	
Formula weight	327.90	
Temperature	150 K	
Wavelength	1.54180 Å	
Crystal system	Orthorhombic	
Space group	P n a 2_1	
Unit cell dimensions	a = 11.29240(10) Å	α= 90°.
	b = 13.6629(2) Å	β= 90°.
	c = 23.2273(2) Å	γ = 90°.
Volume	3583.67(7) $Å^3$	
Z	8	
Density (calculated)	1.215 Mg/m^3	
Absorption coefficient	1.857 mm^{-1}	
F(000)	1408	
Crystal size	0.20 x 0.19 x 0.18 mm^3	
Theta range for data collection	3.753 to 76.284°.	
Index ranges	-13<=h<=14, -16<=k<=17, -27<=l<=29	
Reflections collected	43420	
Independent reflections	6973 [R(int) = 0.027]	
Completeness to theta = 76.284°	99.7 %	
Absorption correction	Semi-empirical from equivalents	
Max. and min. transmission	0.72 and 0.56	
Refinement method	Full-matrix least-squares on F^2	
Data / restraints / parameters	6973 / 1 / 416	
Goodness-of-fit on F^2	1.0023	
Final R indices [I>2sigma(I)]	R1 = 0.0243, wR2 = 0.0666	
R indices (all data)	R1 = 0.0247, wR2 = 0.0671	
Absolute structure parameter	0.014(4)	
Largest diff. peak and hole	0.06 and -0.07 e.$Å^{-3}$	

Table 4. Crystal data and structure refinement for **8e**.

Empirical formula	C23 H28 I N	
Formula weight	445.39	
Temperature	150 K	
Wavelength	1.54184 Å	
Crystal system	Monoclinic	
Space group	P 2_1/n	
Unit cell dimensions	a = 9.2483(3) Å	α= 90°.
	b = 14.3133(4) Å	β= 106.151(4)°.
	c = 16.1073(5) Å	γ = 90°.
Volume	2048.03(12) $Å^3$	
Z	4	
Density (calculated)	1.444 Mg/m^3	
Absorption coefficient	12.297 mm^{-1}	
F(000)	904	
Crystal size	0.07 x 0.05 x 0.05 mm^3	
Theta range for data collection	4.208 to 76.604°.	
Index ranges	-11<=h<=11, -17<=k<=12, -19<=l<=20	
Reflections collected	14033	
Independent reflections	4251 [R(int) = 0.032]	
Completeness to theta = 74.306°	99.6 %	
Absorption correction	Semi-empirical from equivalents	
Max. and min. transmission	0.54 and 0.05	
Refinement method	Full-matrix least-squares on F^2	
Data / restraints / parameters	4244 / 0 / 226	
Goodness-of-fit on F^2	1.0544	
Final R indices [I>2sigma(I)]	R1 = 0.0767, wR2 = 0.1713	
R indices (all data)	R1 = 0.0819, wR2 = 0.1815	
Largest diff. peak and hole	2.84 and -0.50 e.$Å^{-3}$	

Kirsten to add data here for

Spectra

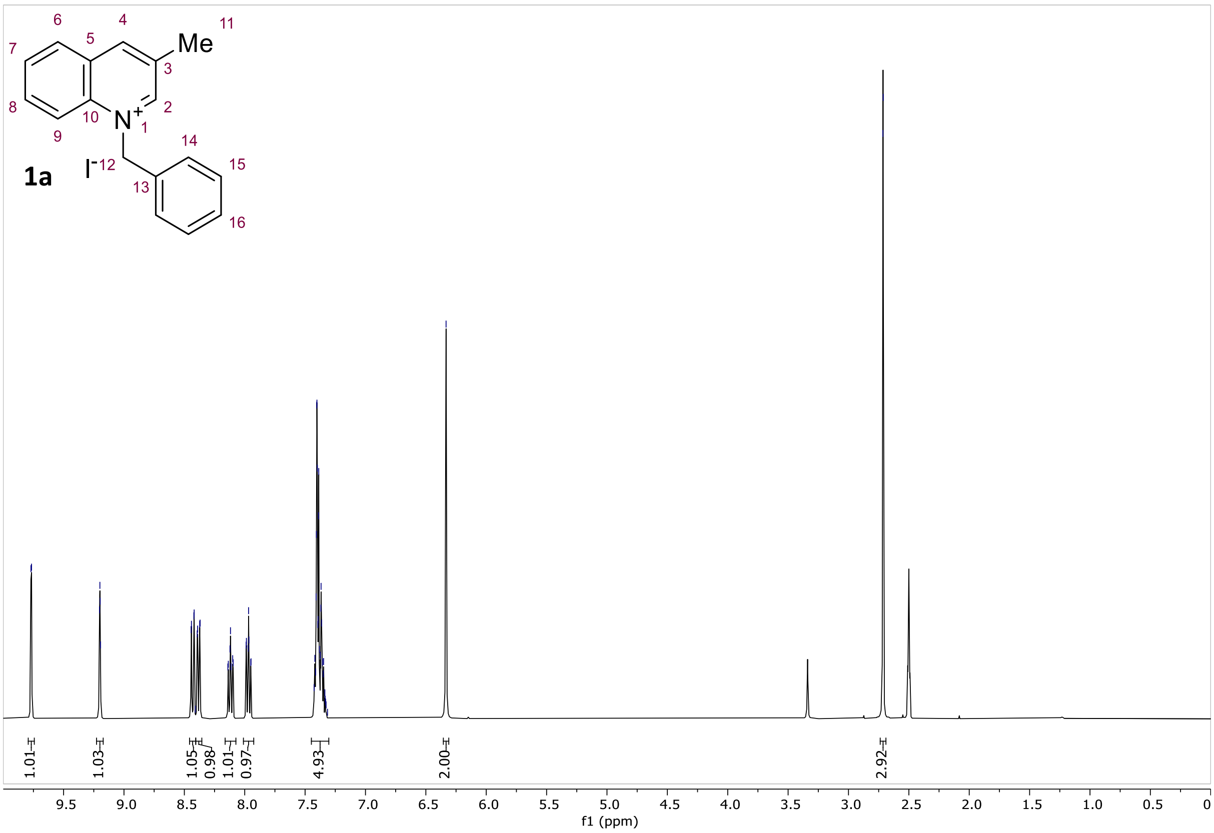

1a
Me
I-
1.01
1.03
1.05
0.98
1.01
0.97
4.93
2.00
2.92
f1 (ppm)

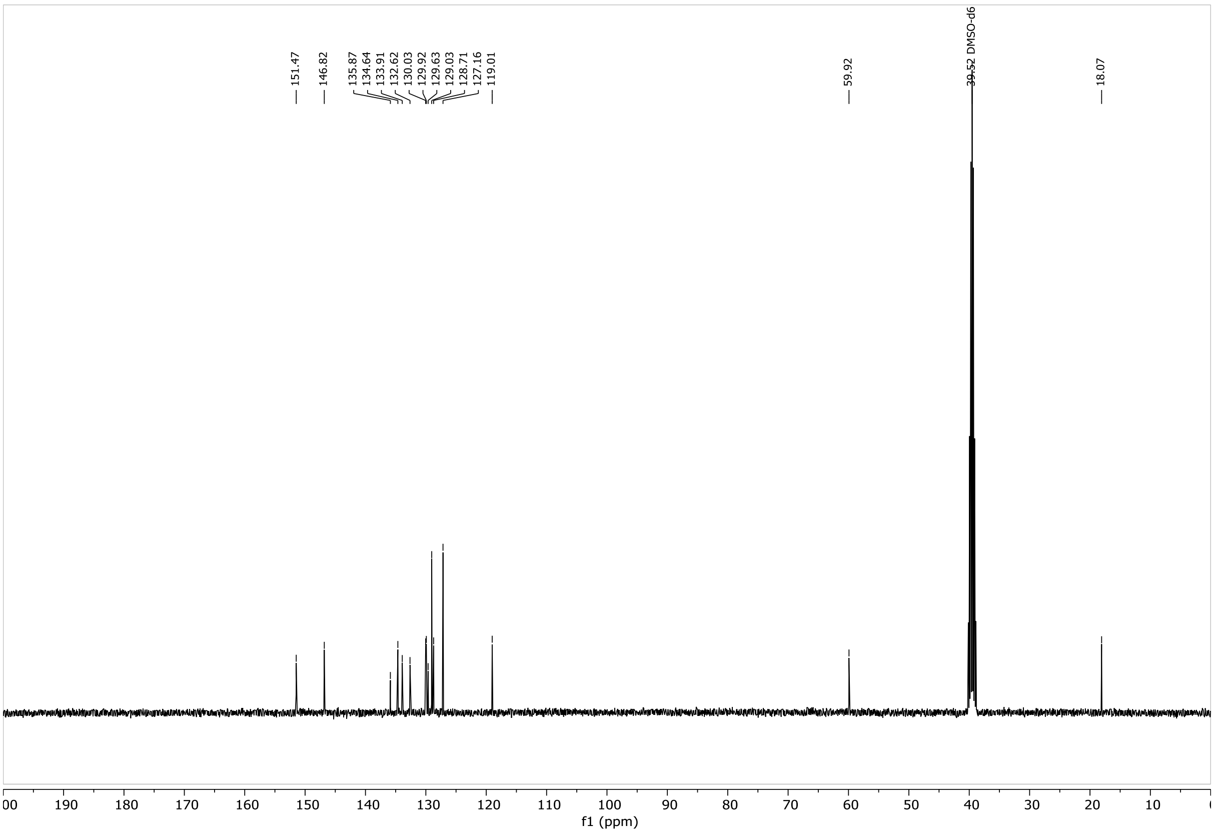

151.47
146.82
135.87
134.64
133.91
132.62
130.03
129.92
129.63
129.03
128.71
127.16
119.01
59.92
39.52 DMSO-d6
18.07
f1 (ppm)

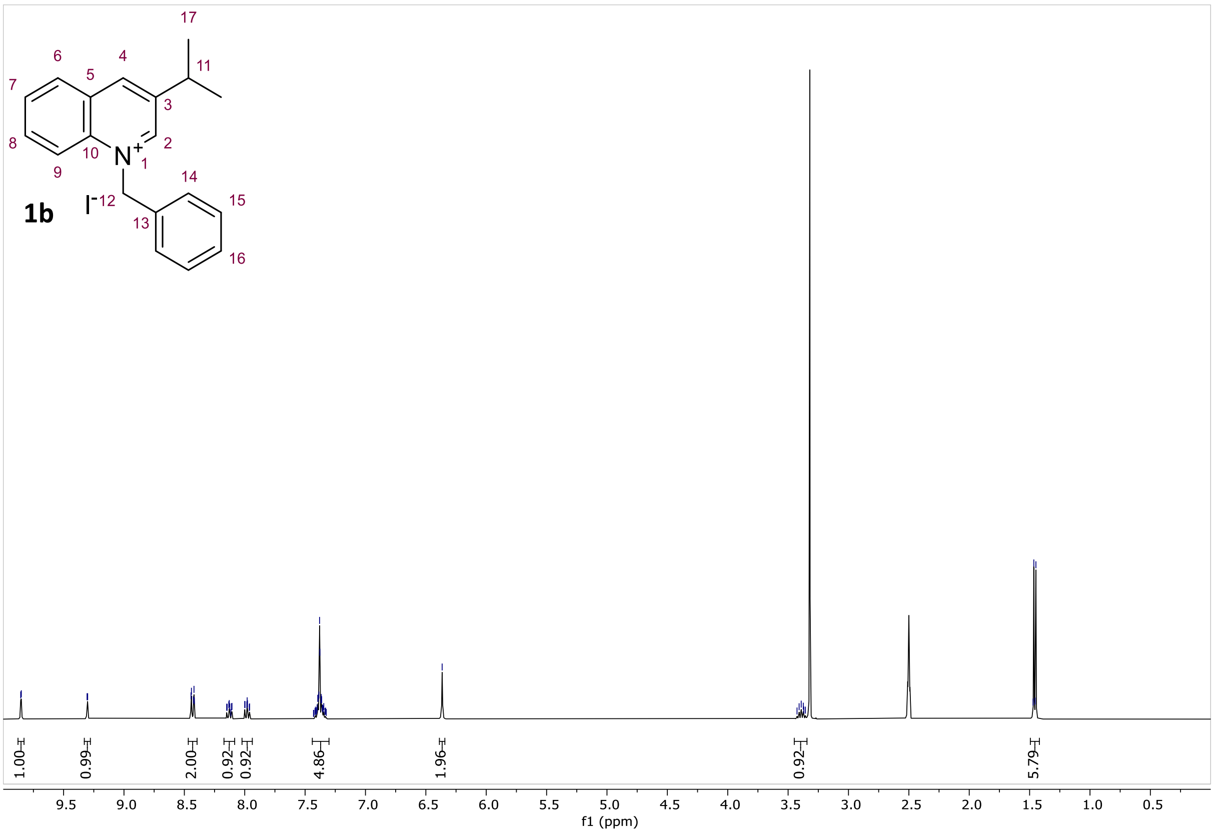
1b
I⁻
N+
1.00
0.99
2.00
0.92
0.92
4.86
1.96
0.92
5.79
9.5 9.0 8.5 8.0 7.5 7.0 6.5 6.0 5.5 5.0 4.5 4.0 3.5 3.0 2.5 2.0 1.5 1.0 0.5
f1 (ppm)

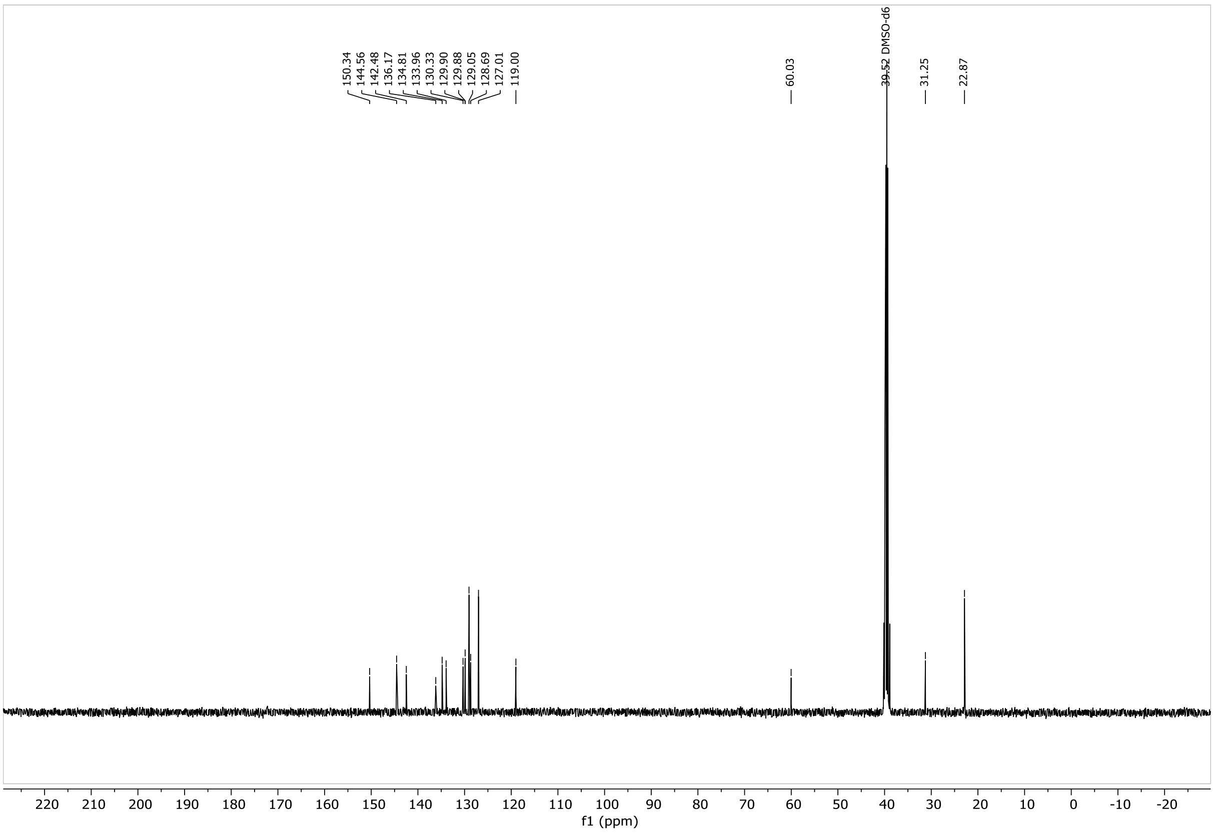
150.34
144.56
142.48
136.17
134.81
133.96
130.33
129.90
129.88
129.05
128.69
127.01
119.00
60.03
39.52 DMSO-d6
31.25
22.87
220 210 200 190 180 170 160 150 140 130 120 110 100 90 80 70 60 50 40 30 20 10 0 -10 -20
f1 (ppm)

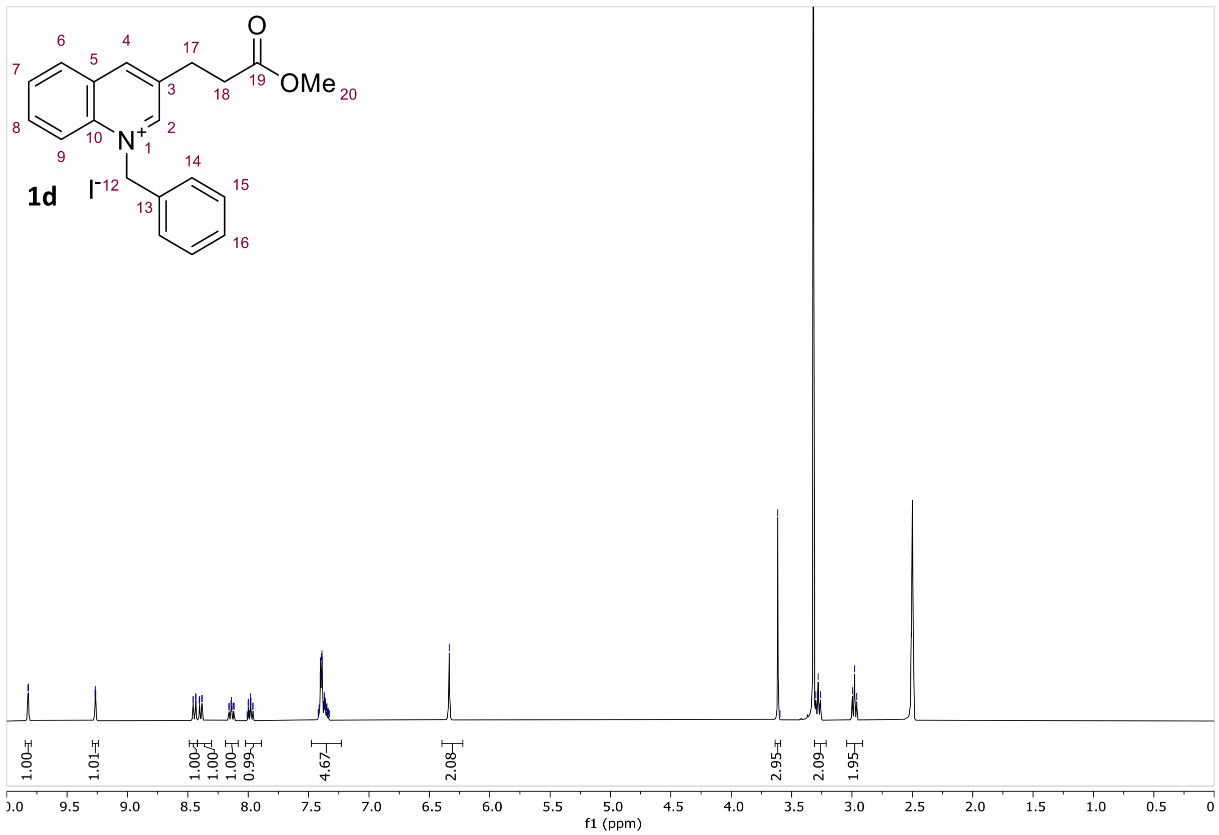
1d
OMe
I⁻
1.00
1.01
1.00
1.00
1.00
0.99
4.67
2.08
2.95
2.09
1.95
f1 (ppm)

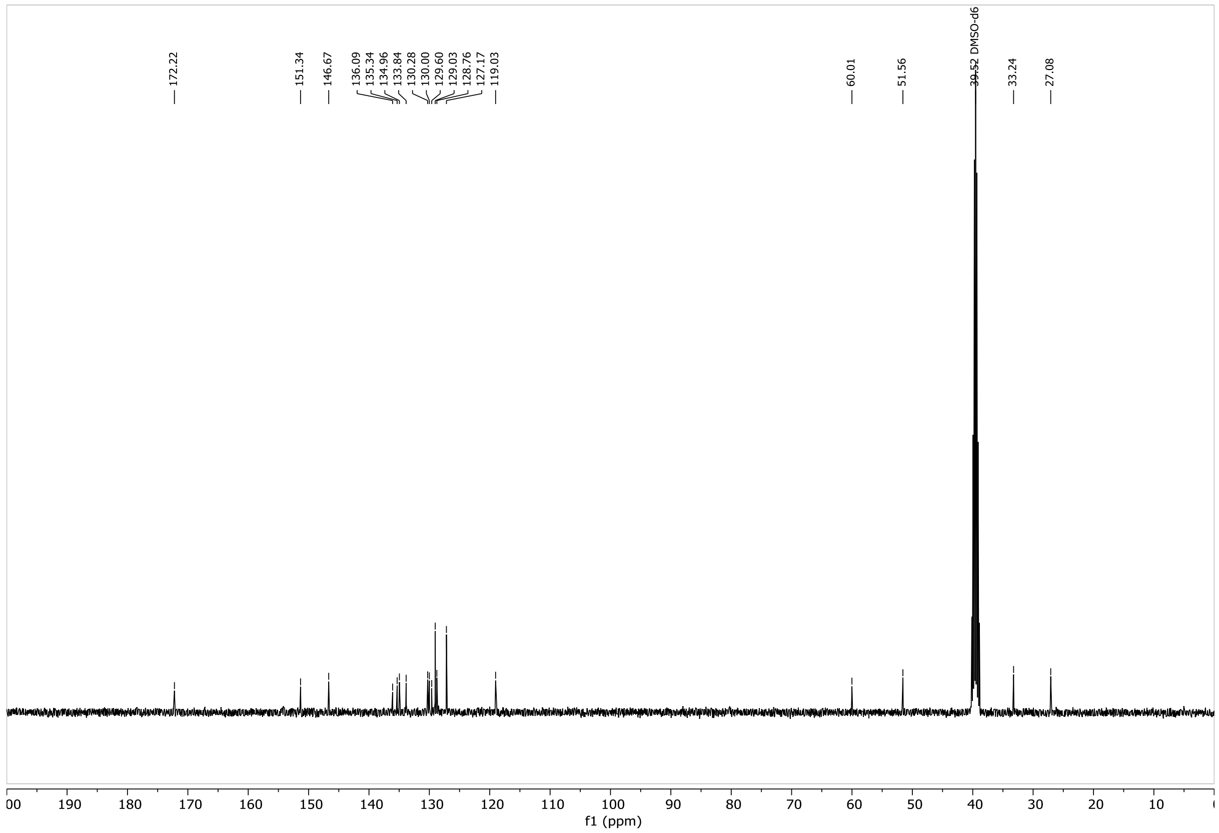
172.22
151.34
146.67
136.09
135.34
134.96
133.84
130.28
130.00
129.60
129.03
128.76
127.17
119.03
60.01
51.56
39.52 DMSO-d6
33.24
27.08
f1 (ppm)

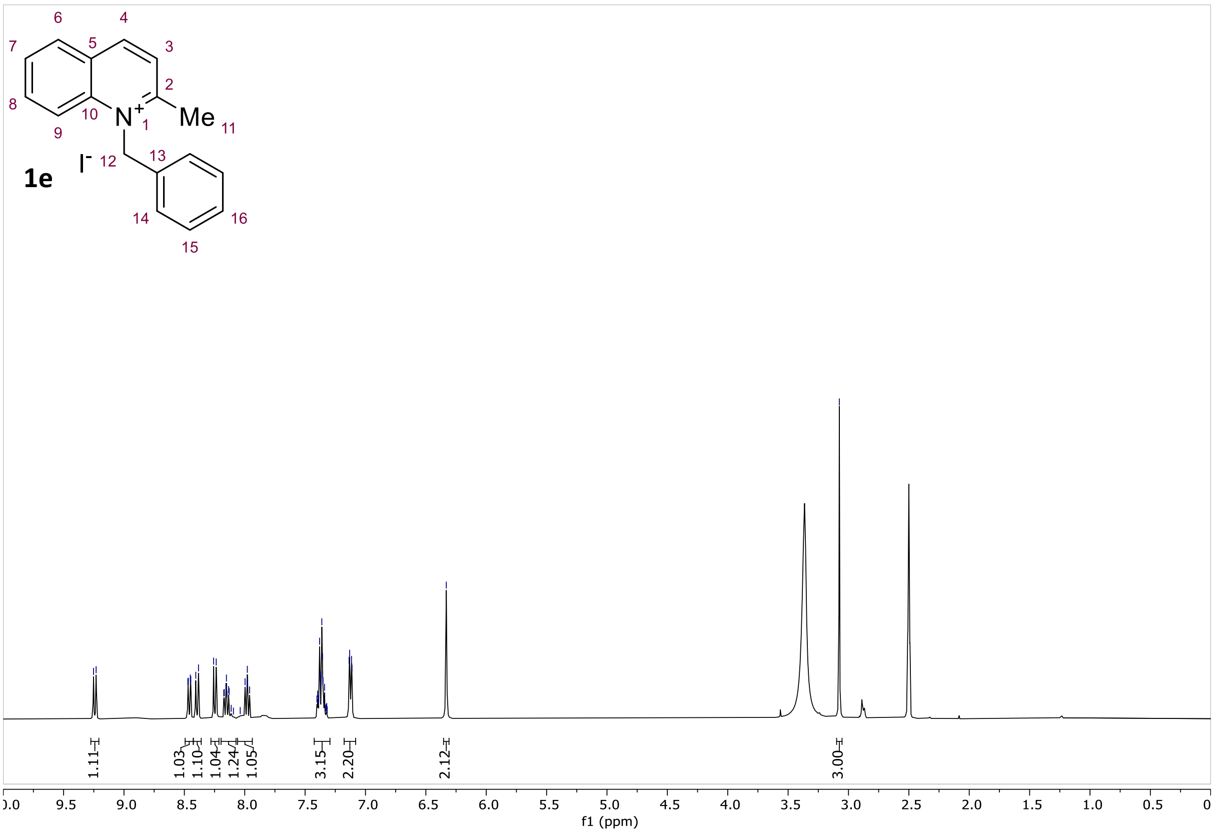
1e
Me
I-
1.11
1.03
1.10
1.04
1.24
1.05
3.15
2.20
2.12
3.00
f1 (ppm)

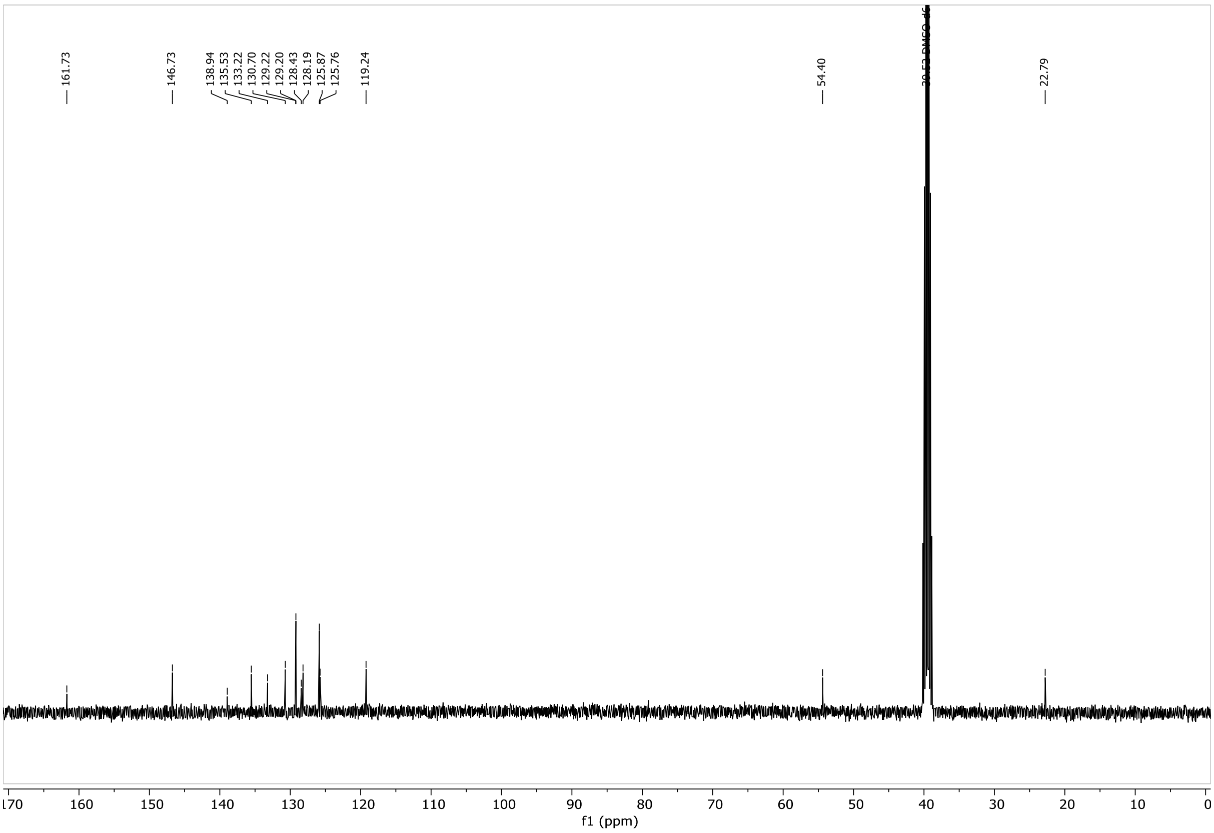
161.73
146.73
138.94
135.53
133.22
130.70
129.22
129.20
128.43
128.19
125.87
125.76
119.24
54.40
22.79
f1 (ppm)

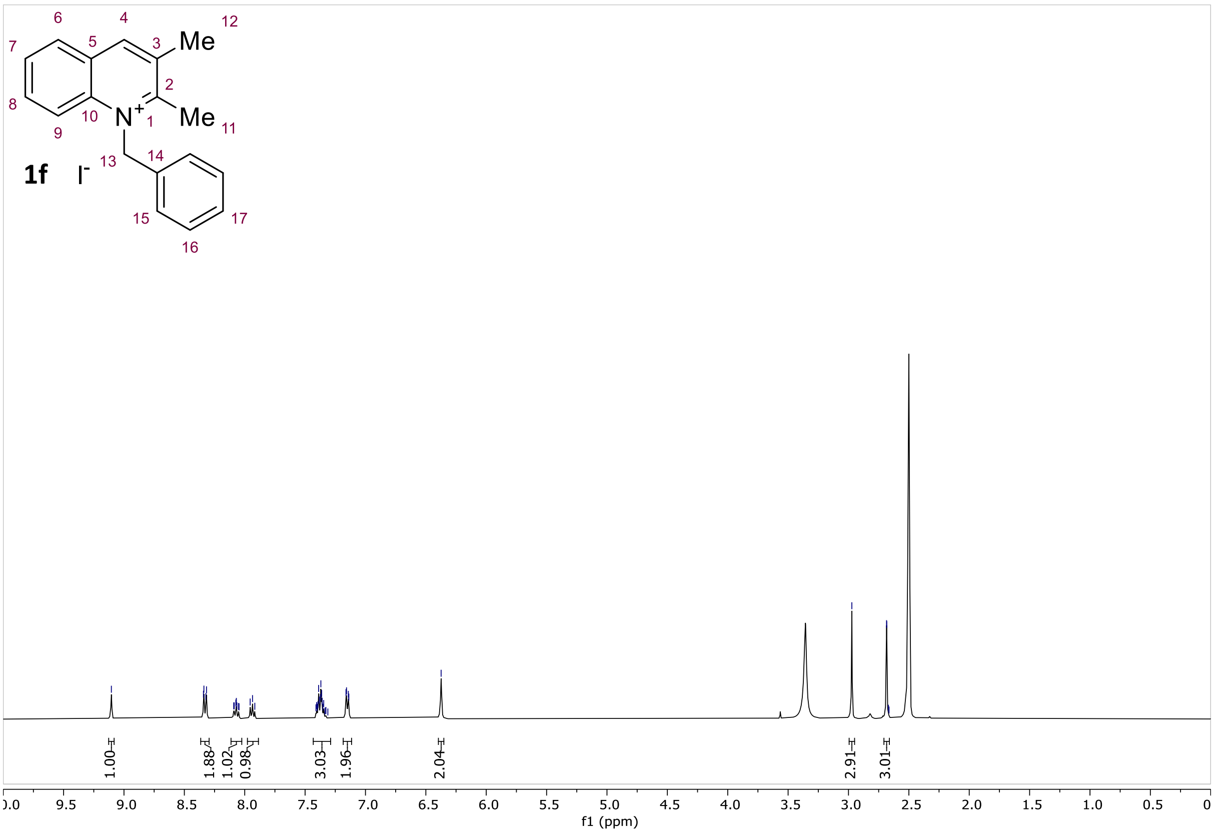

Me 12
Me 11
N+ 1
1f I-
1.00
1.88
1.02
0.98
3.03
1.96
2.04
2.91
3.01
f1 (ppm)

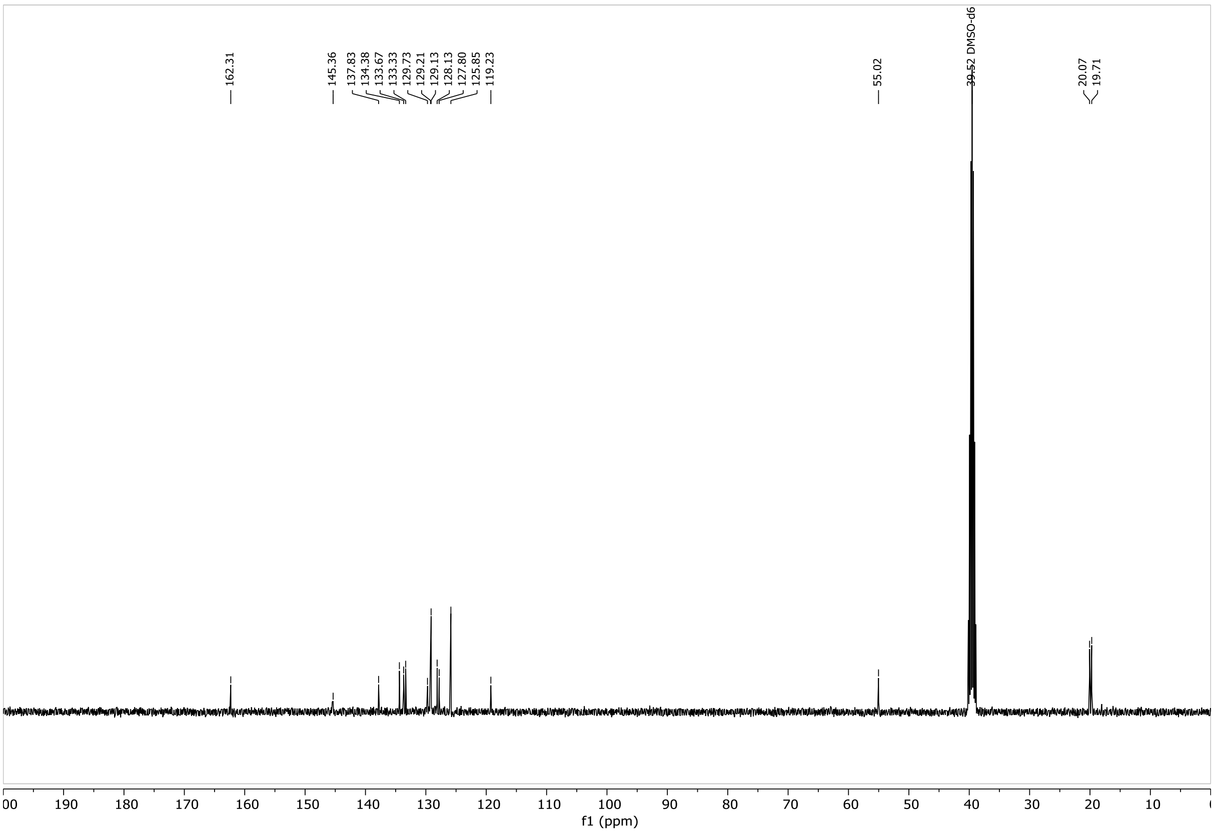

162.31
145.36
137.83
134.38
133.67
133.33
129.73
129.21
129.13
128.13
127.80
125.85
119.23
55.02
39.52 DMSO-d6
20.07
19.71
f1 (ppm)

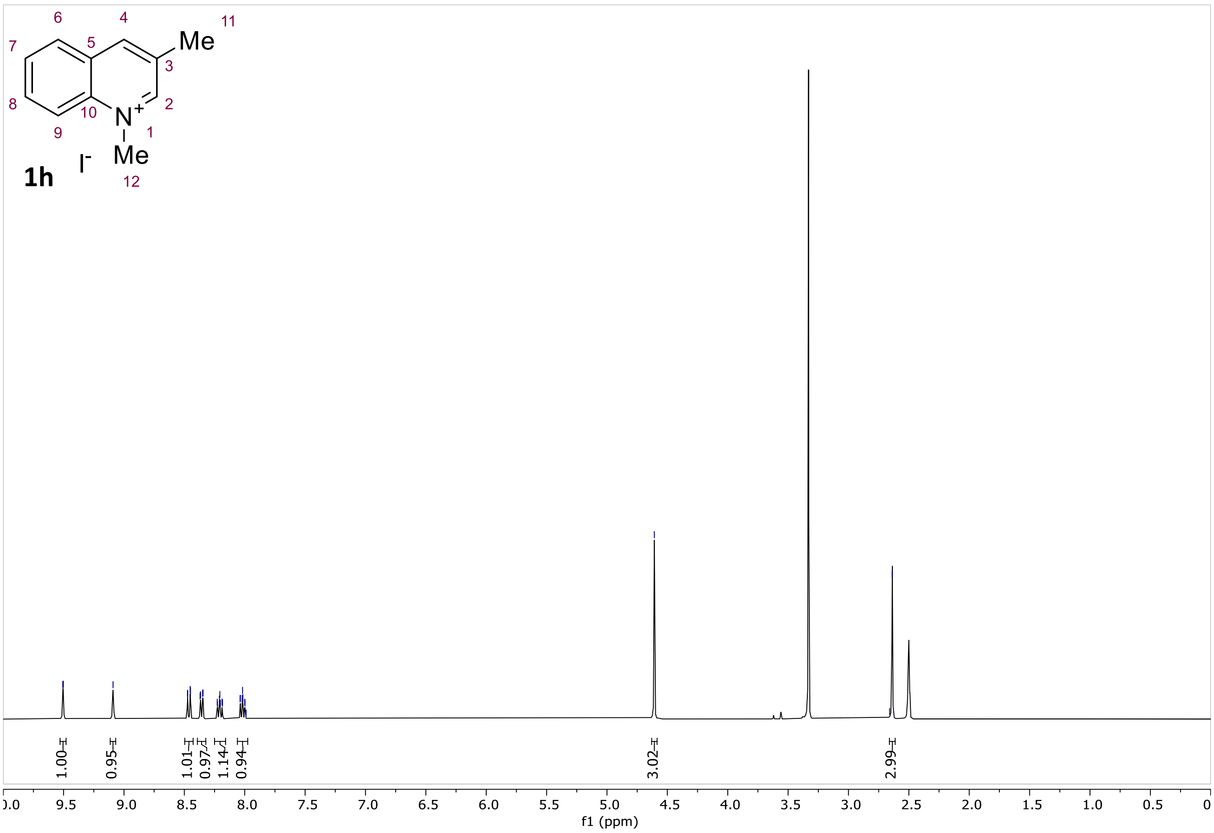

1h
Me
Me
I-
1.00
0.95
1.01
0.97
1.14
0.94
3.02
2.99
f1 (ppm)

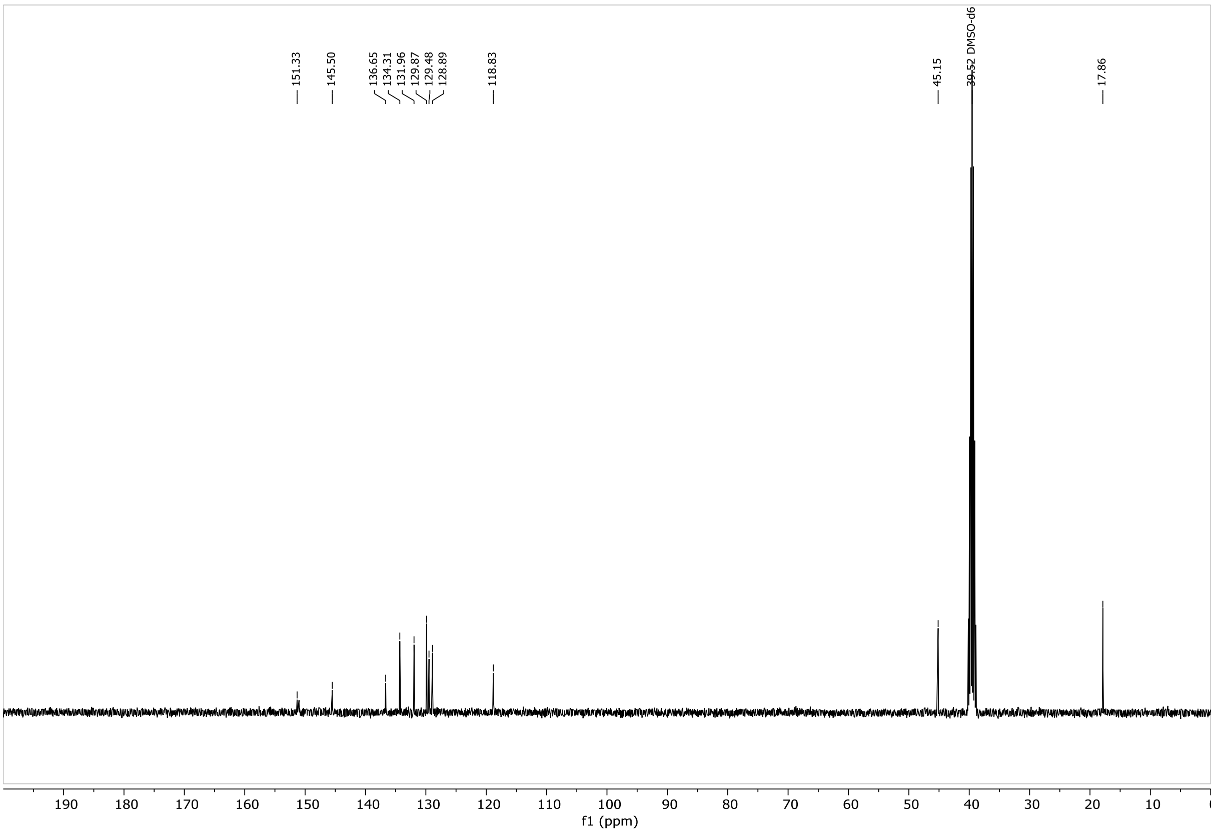

151.33
145.50
136.65
134.31
131.96
129.87
129.48
128.89
118.83
45.15
39.52 DMSO-d6
17.86
f1 (ppm)

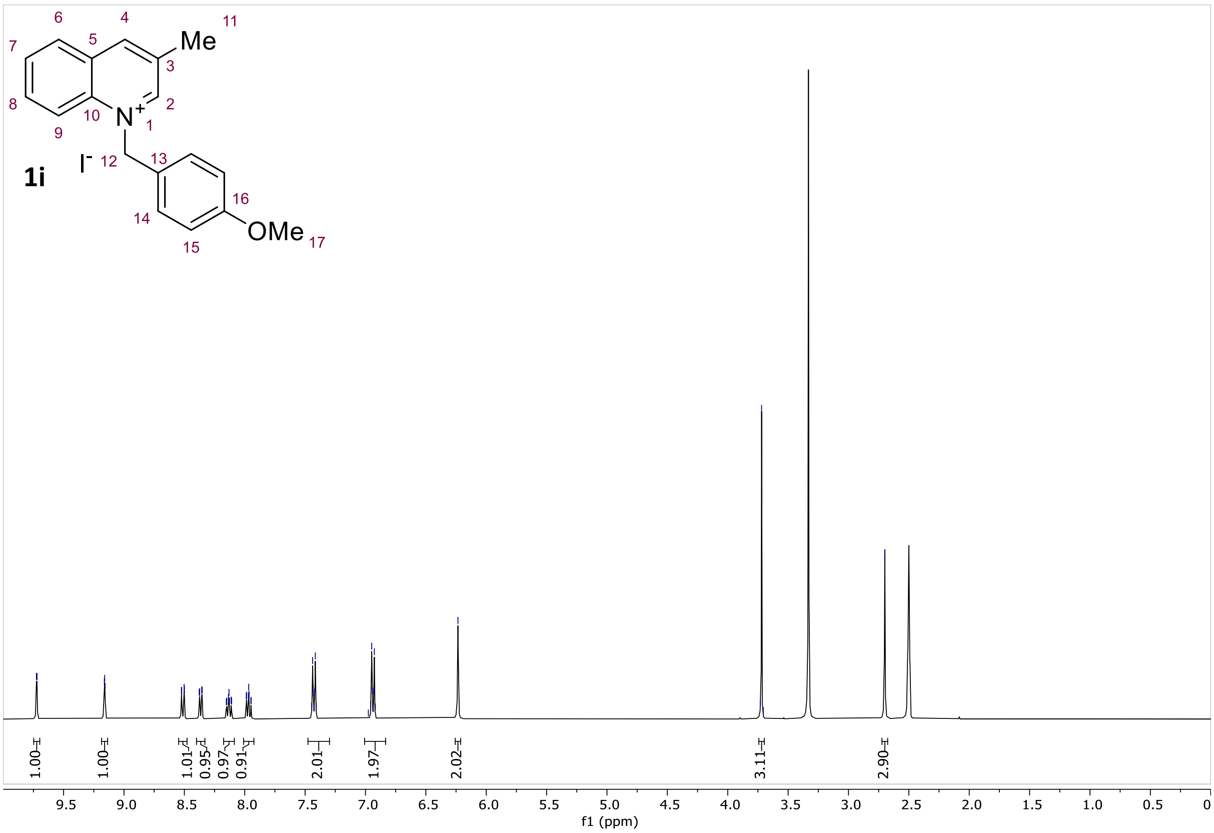
1i
Me
OMe
1.00
1.00
1.01
0.95
0.97
0.91
2.01
1.97
2.02
3.11
2.90
f1 (ppm)

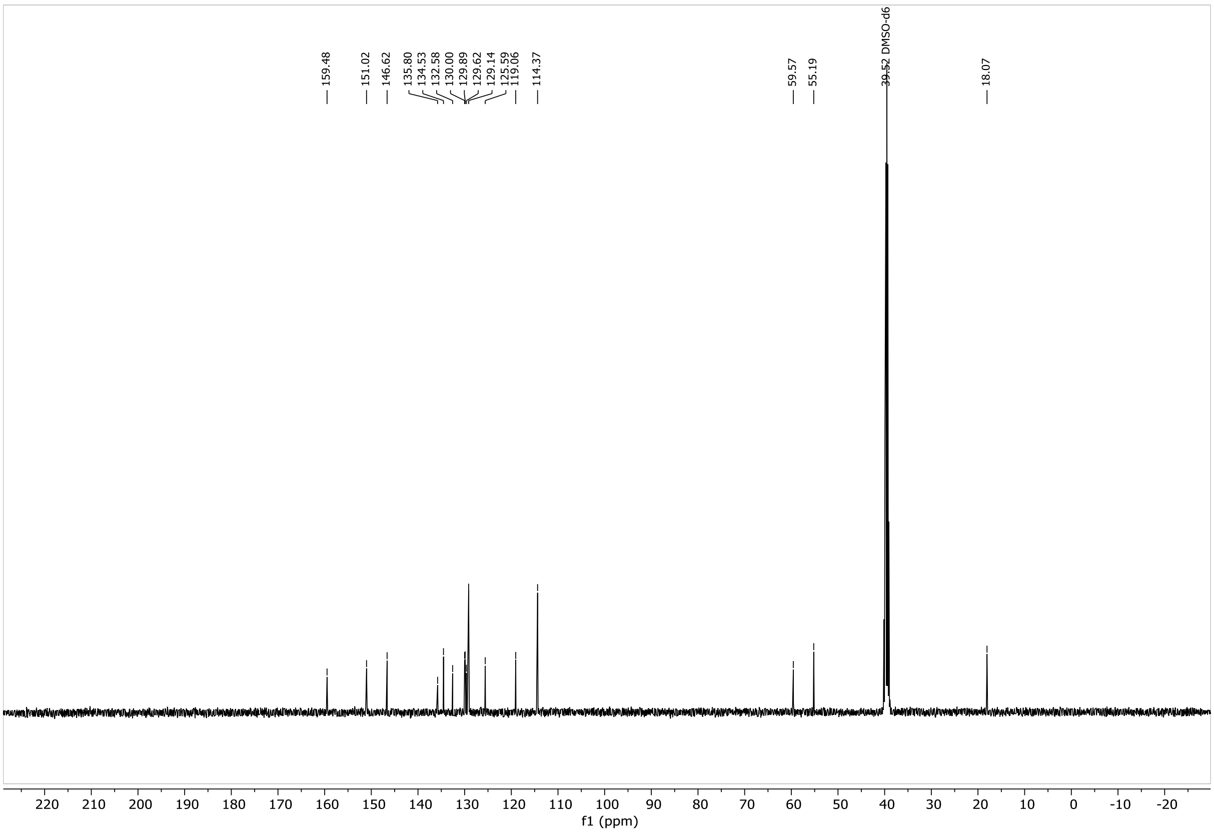
159.48
151.02
146.62
135.80
134.53
132.58
130.00
129.89
129.62
129.14
125.59
119.06
114.37
59.57
55.19
39.52 DMSO-d6
18.07
f1 (ppm)

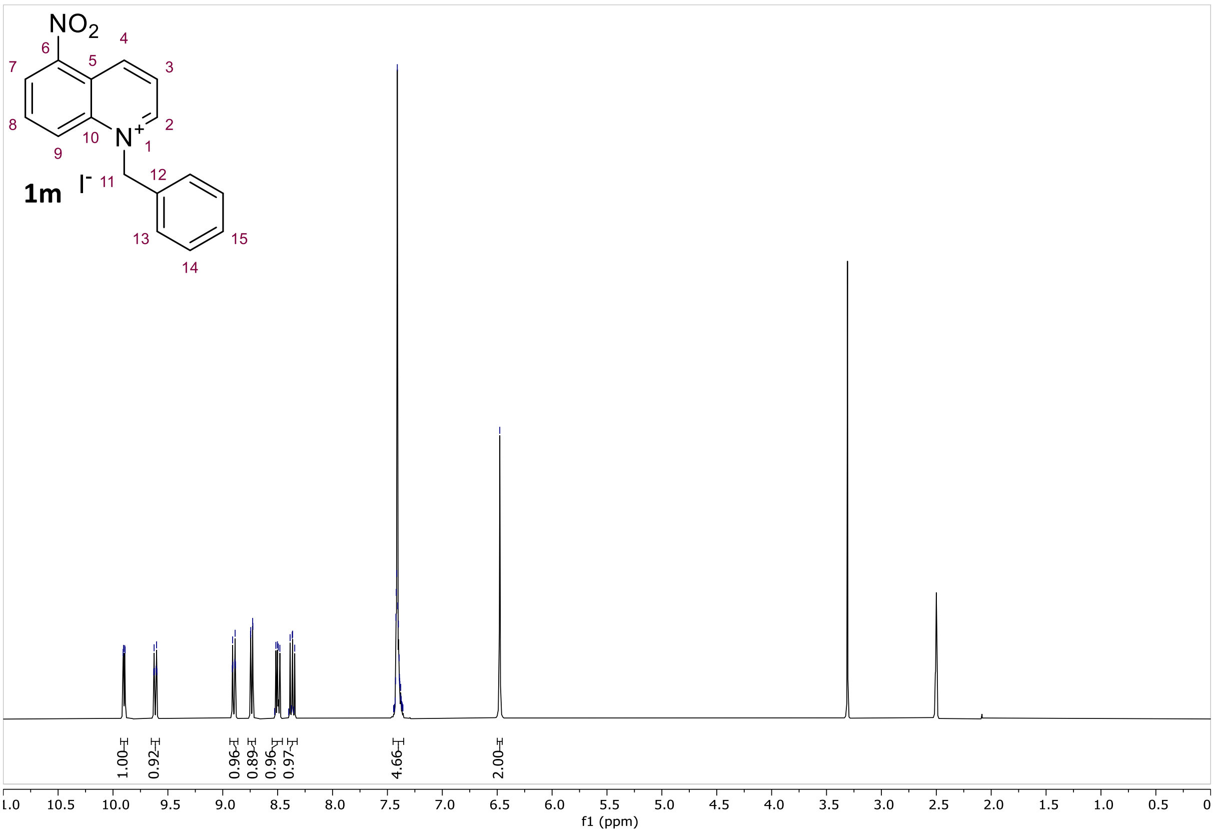

NO2
1m I-
1.00
0.92
0.96
0.89
0.96
0.97
4.66
2.00
f1 (ppm)

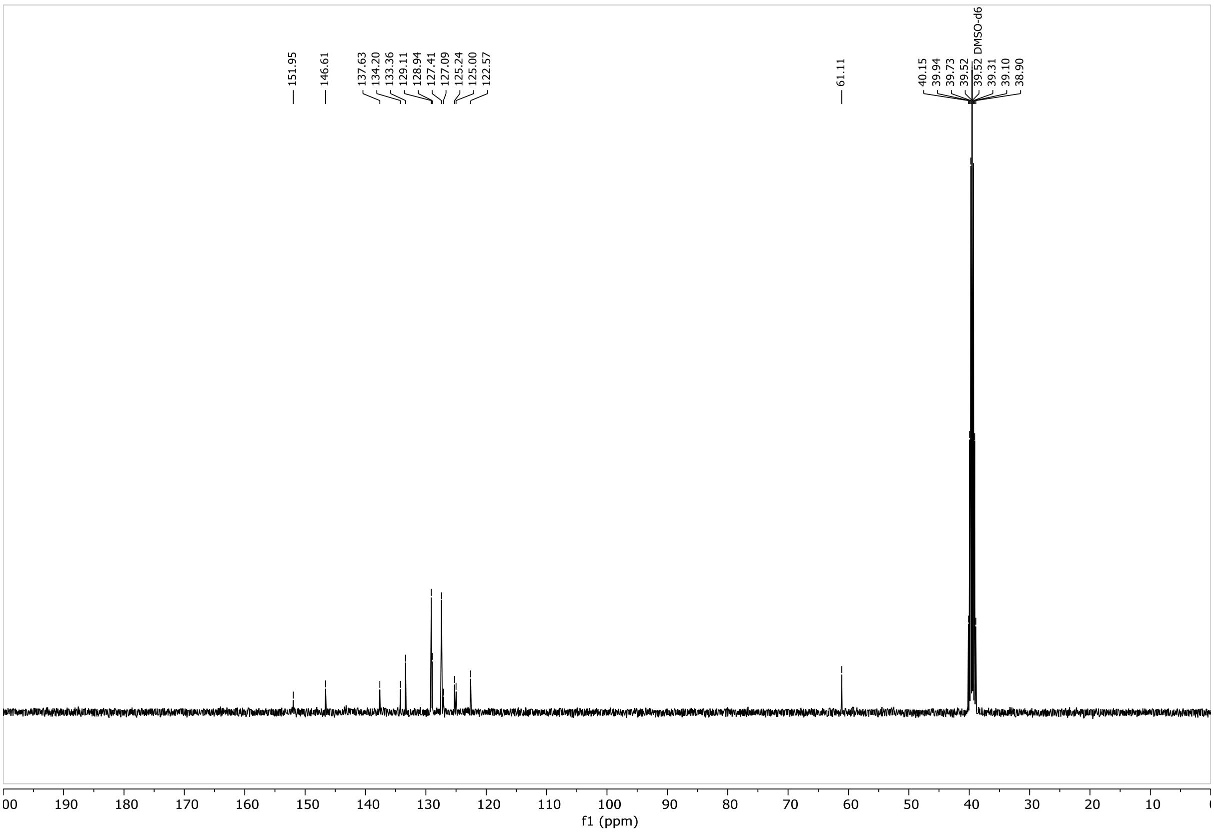

151.95
146.61
137.63
134.20
133.36
129.11
128.94
127.41
127.09
125.24
125.00
122.57
61.11
40.15
39.94
39.73
39.52
39.52 DMSO-d6
39.31
39.10
38.90
f1 (ppm)

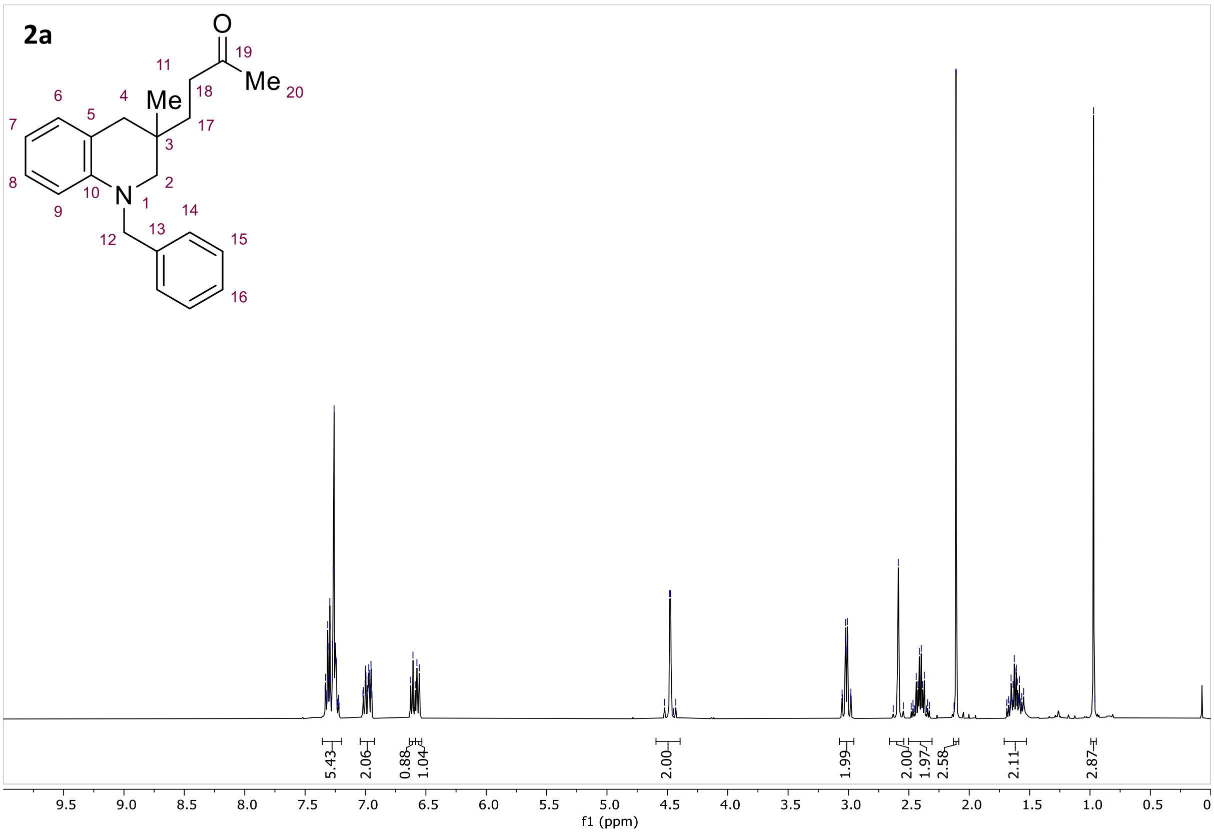
2a
O
19
Me 20
11
18
Me
4
6
5
17
7
3
2
8
10
N
1
14
9
12
13
15
16
5.43
2.06
0.88
1.04
2.00
1.99
2.00
1.97
2.58
2.11
2.87
9.5 9.0 8.5 8.0 7.5 7.0 6.5 6.0 5.5 5.0 4.5 4.0 3.5 3.0 2.5 2.0 1.5 1.0 0.5 0
f1 (ppm)

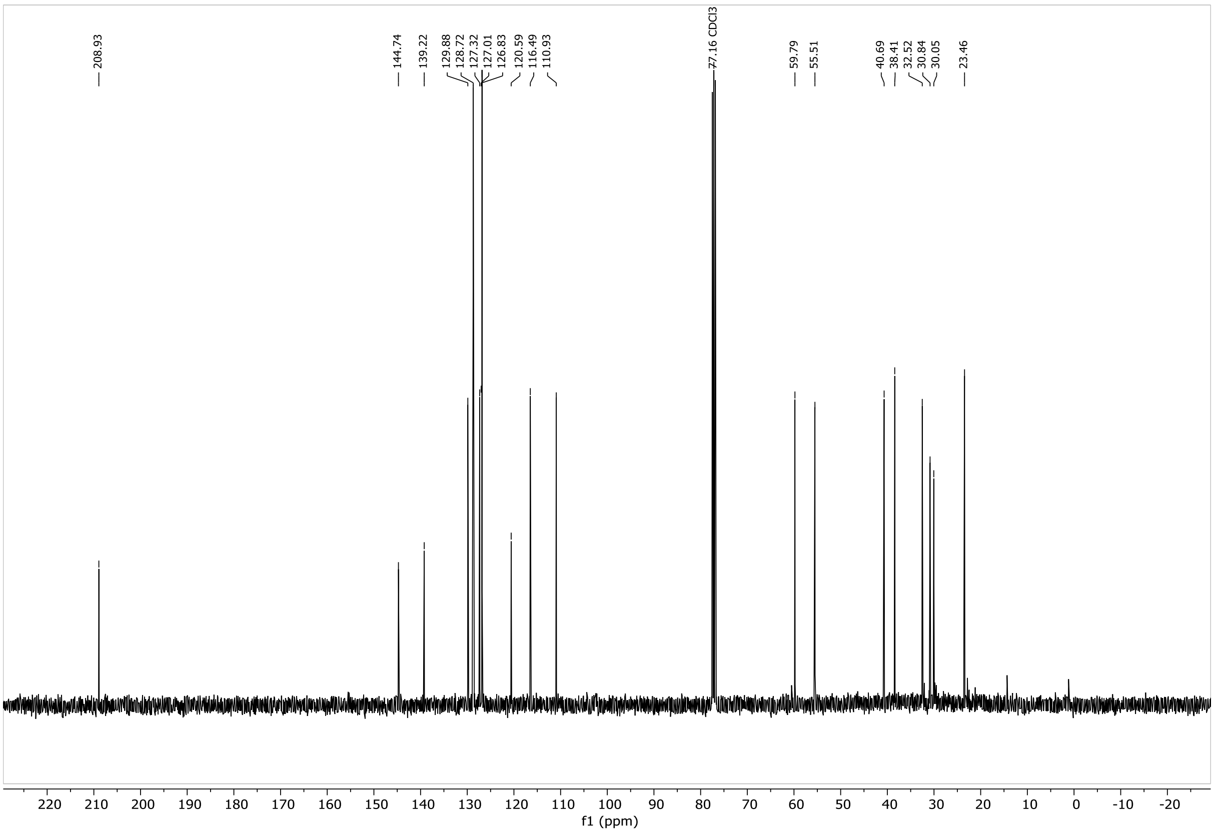
208.93
144.74
139.22
129.88
128.72
127.32
127.01
126.83
120.59
116.49
110.93
77.16 CDCl3
59.79
55.51
40.69
38.41
32.52
30.84
30.05
23.46
220 210 200 190 180 170 160 150 140 130 120 110 100 90 80 70 60 50 40 30 20 10 0 -10 -20
f1 (ppm)

2b

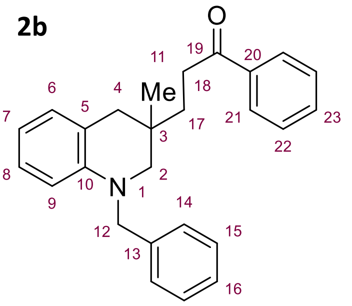
O
Me
N
1
2
3
4
5
6
7
8
9
10
11
12
13
14
15
16
17
18
19
20
21
22
23

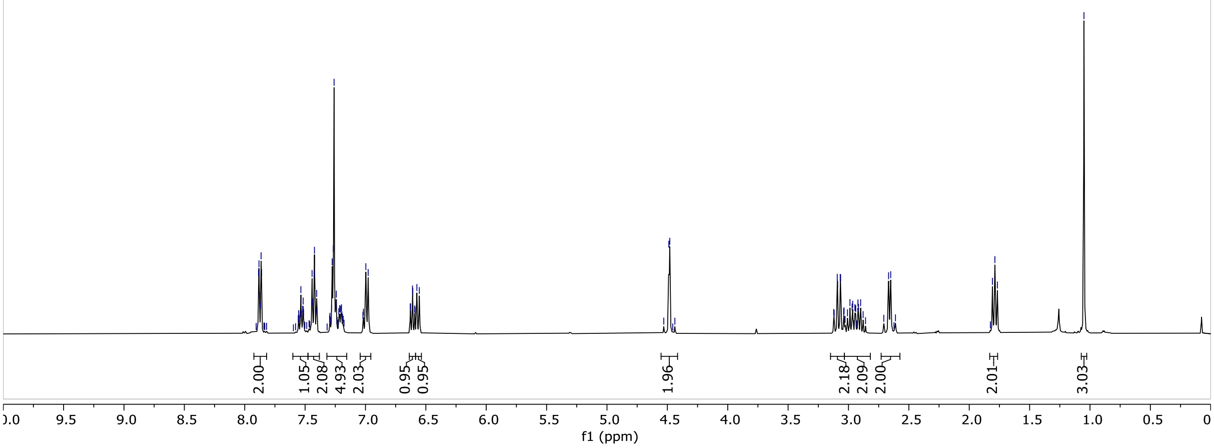
2.00
1.05
2.08
4.93
2.03
0.95
0.95
1.96
2.18
2.09
2.00
2.01
3.03
f1 (ppm)

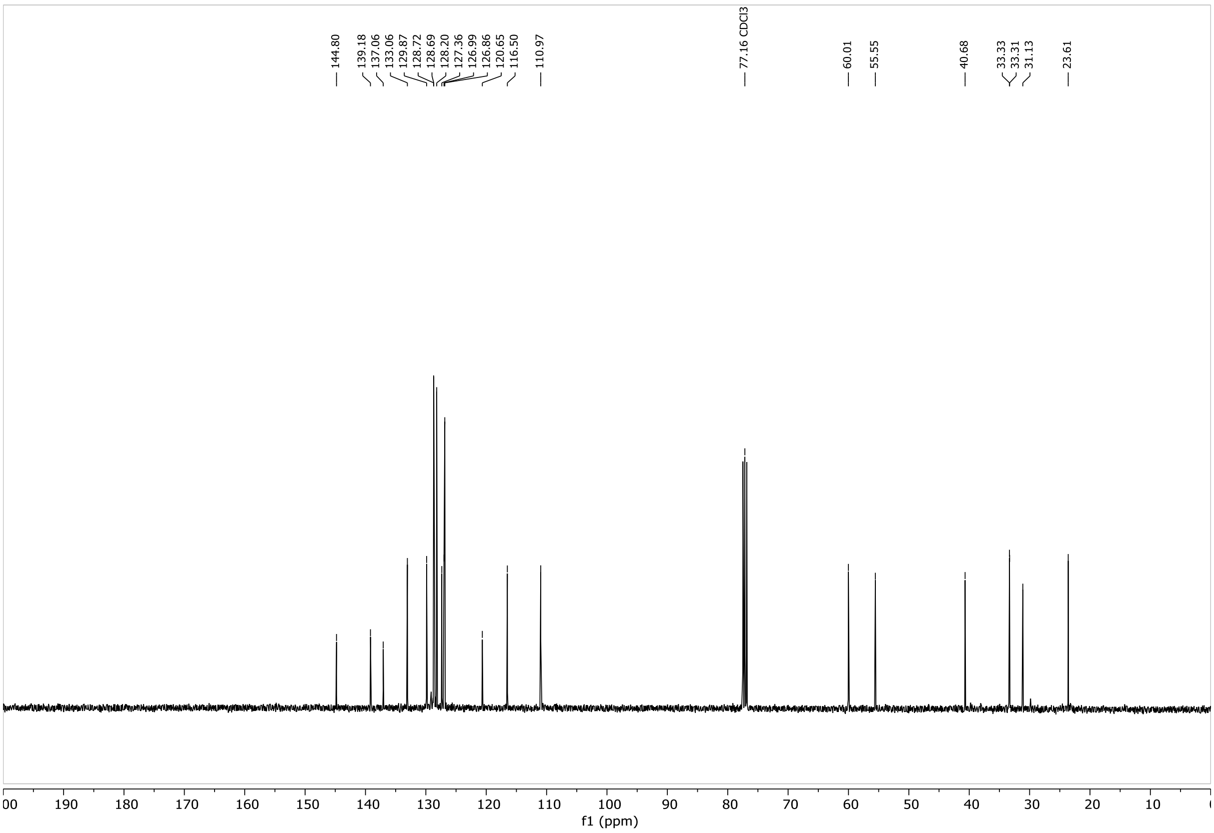
144.80
139.18
137.06
133.06
129.87
128.72
128.69
128.20
127.36
126.99
126.86
120.65
116.50
110.97
77.16 CDCl3
60.01
55.55
40.68
33.33
33.31
31.13
23.61
f1 (ppm)

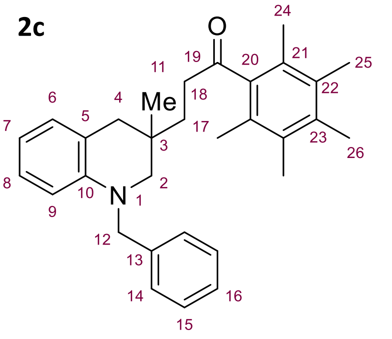

2c
Me
O
N

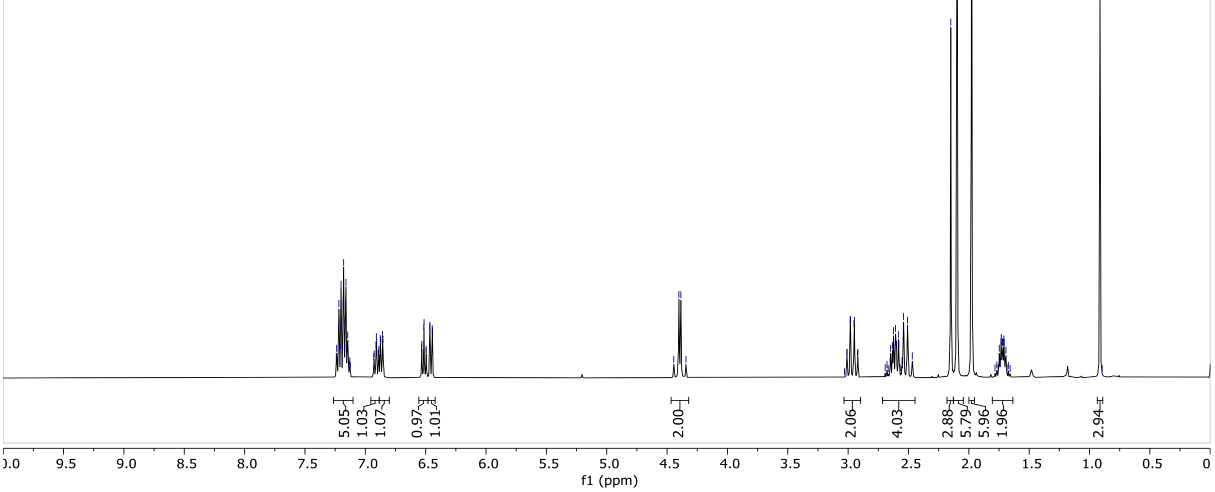

5.05
1.03
1.07
0.97
1.01
2.00
2.06
4.03
2.88
5.79
5.96
1.96
2.94
f1 (ppm)

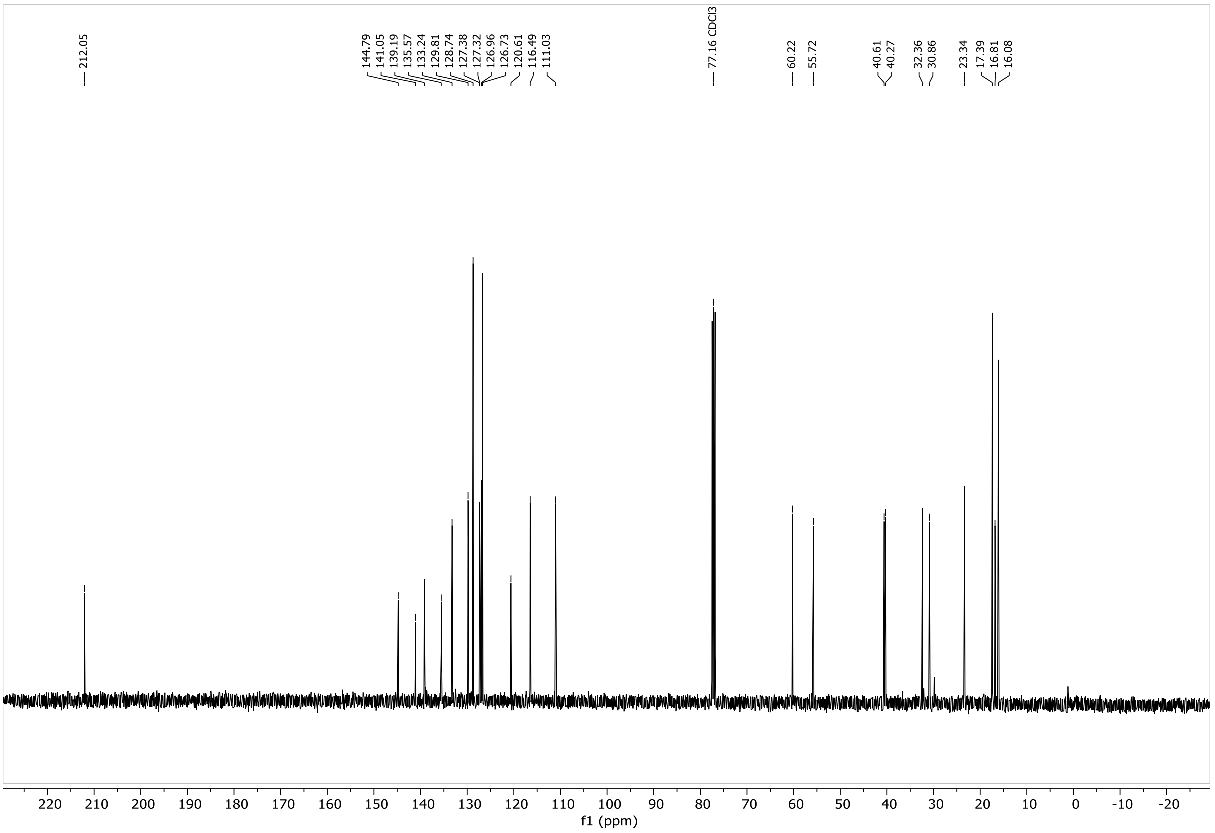

212.05
144.79
141.05
139.19
135.57
133.24
129.81
128.74
127.38
127.32
126.96
126.73
120.61
116.49
111.03
77.16 CDCl3
60.22
55.72
40.61
40.27
32.36
30.86
23.34
17.39
16.81
16.08
f1 (ppm)

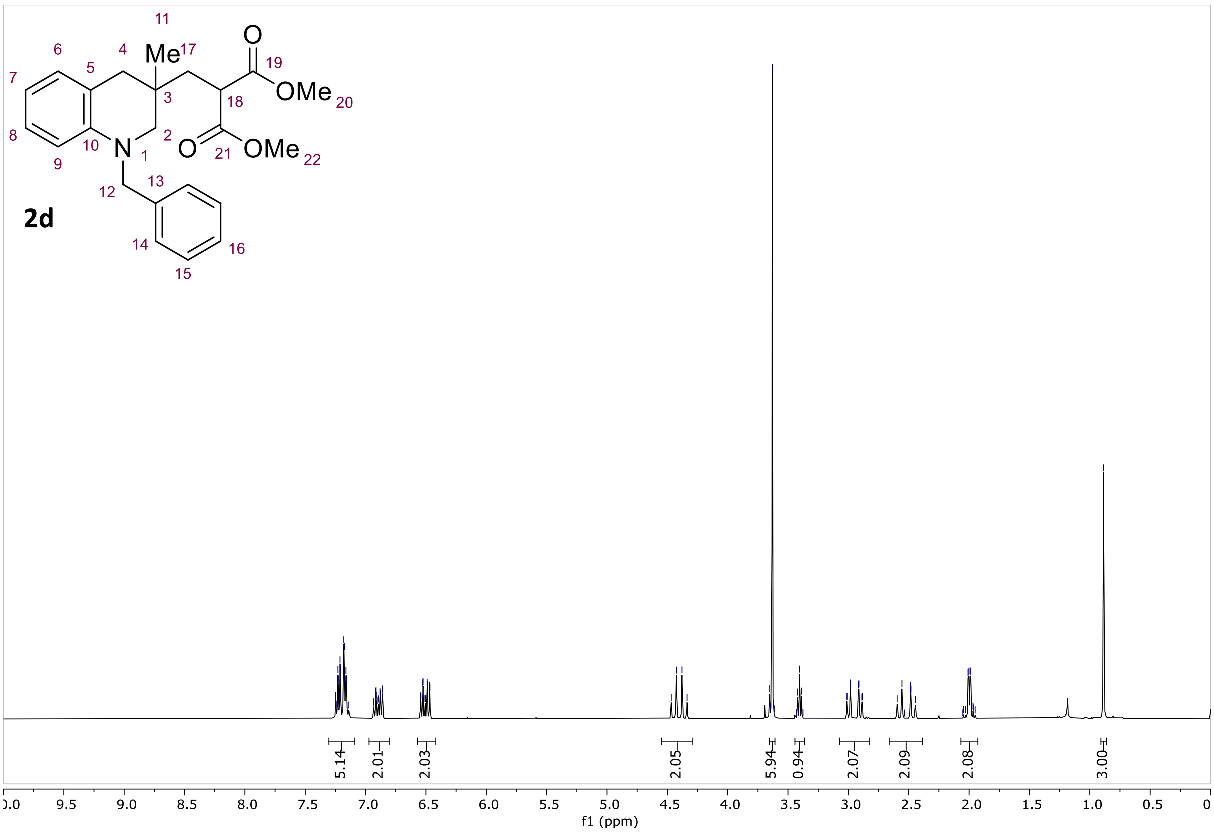

2d
Me
OMe
OMe
O
O
N
5.14
2.01
2.03
2.05
5.94
0.94
2.07
2.09
2.08
3.00
f1 (ppm)

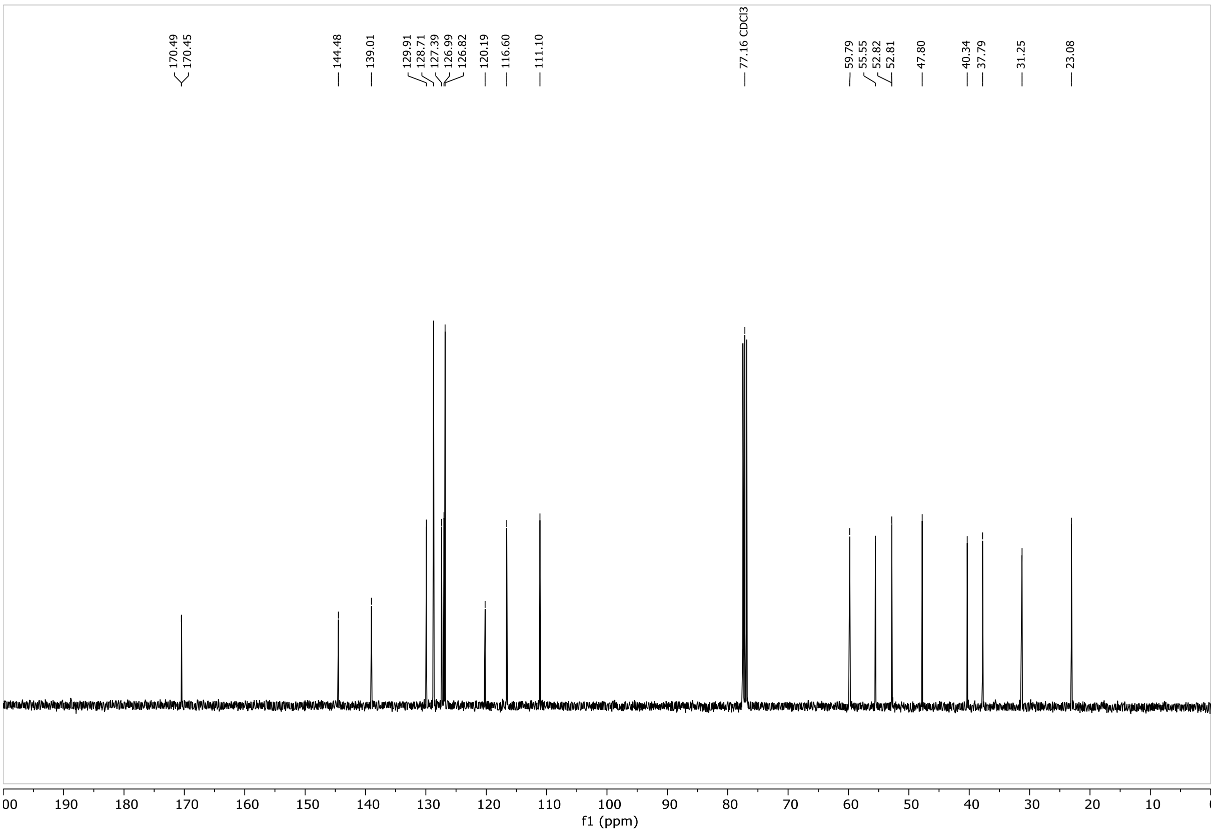

170.49
170.45
144.48
139.01
129.91
128.71
127.39
126.99
126.82
120.19
116.60
111.10
77.16 CDCl3
59.79
55.55
52.82
52.81
47.80
40.34
37.79
31.25
23.08
f1 (ppm)

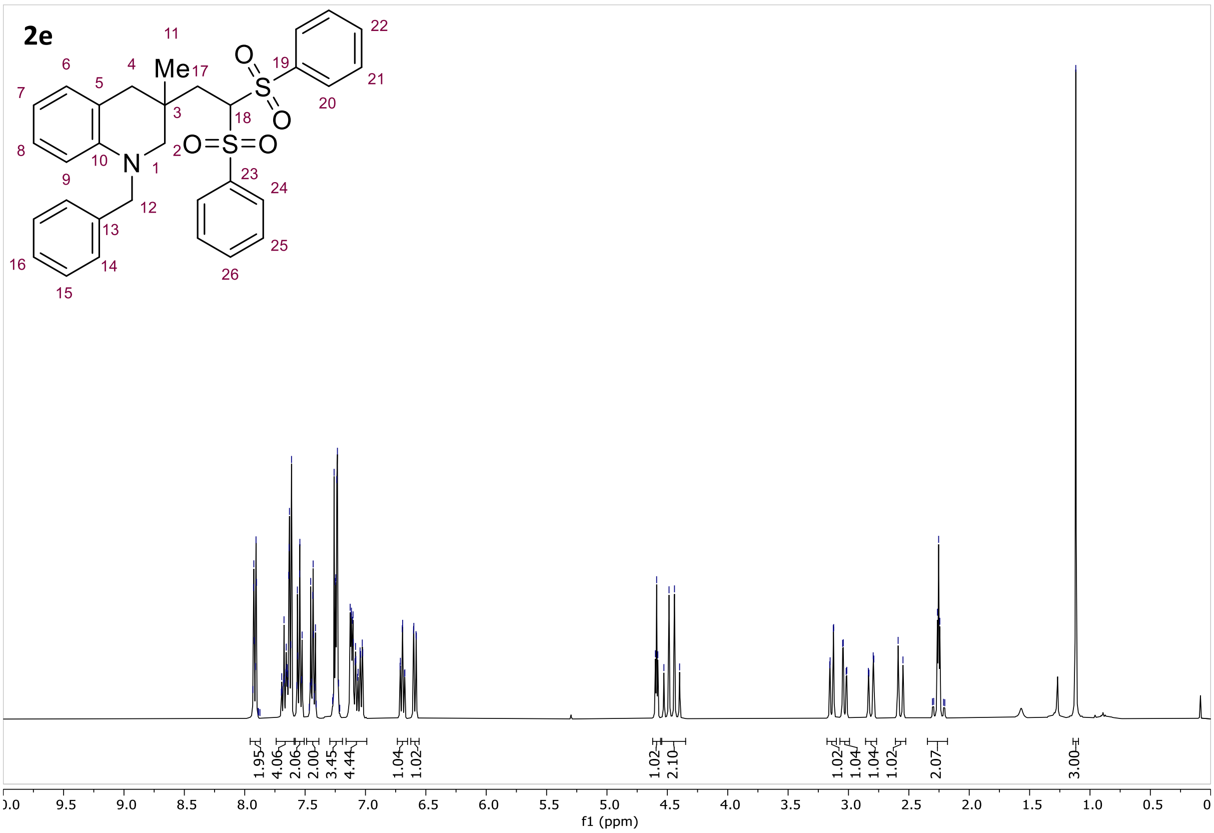
2e
1.95
4.06
2.06
2.00
3.45
4.44
1.04
1.02
1.02
2.10
1.02
1.04
1.04
1.02
2.07
3.00
f1 (ppm)

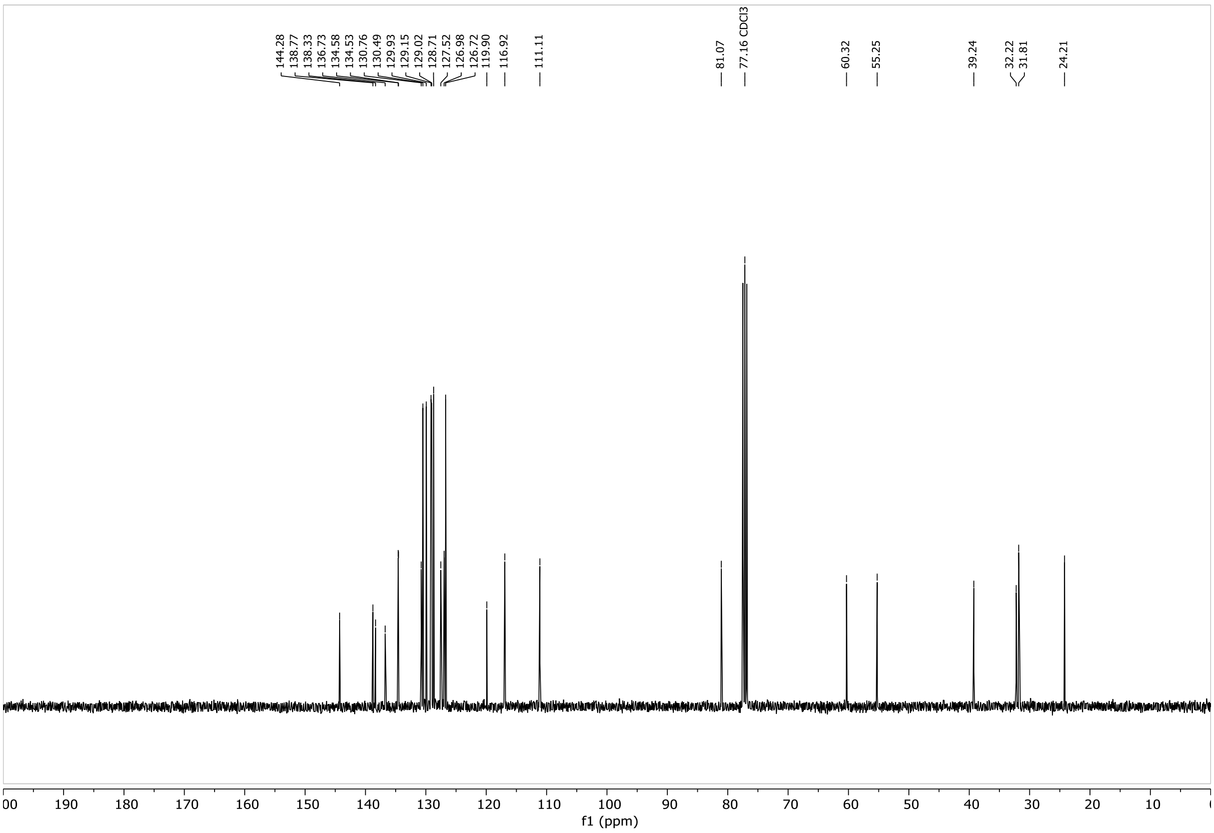
144.28
138.77
138.33
136.73
134.58
134.53
130.76
130.49
129.93
129.15
129.02
128.71
127.52
126.98
126.72
119.90
116.92
111.11
81.07
77.16 CDCl3
60.32
55.25
39.24
32.22
31.81
24.21
f1 (ppm)

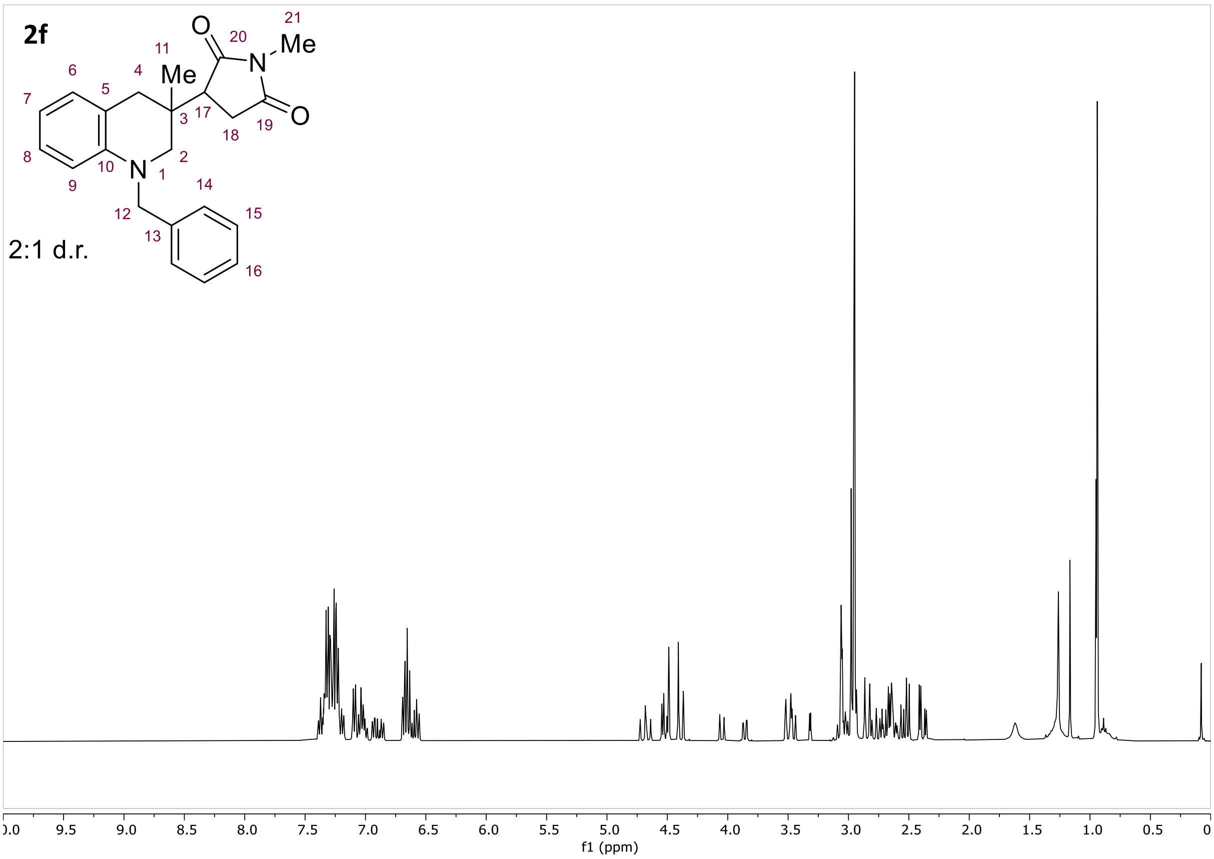
2f
2:1 d.r.
f1 (ppm)

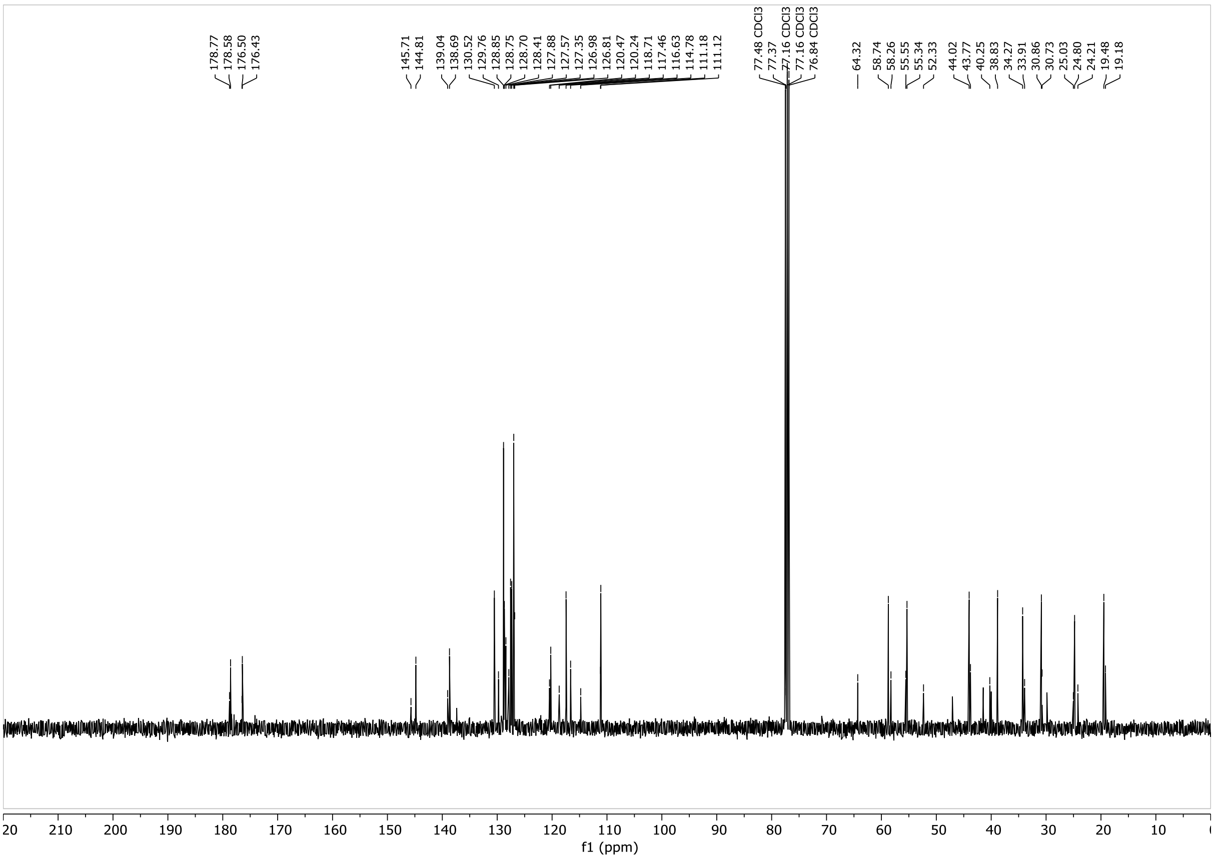
178.77
178.58
176.50
176.43
145.71
144.81
139.04
138.69
130.52
129.76
128.85
128.75
128.70
128.41
127.88
127.57
127.35
126.98
126.81
120.47
120.24
118.71
117.46
116.63
114.78
111.18
111.12
77.48 CDCl3
77.37
77.16 CDCl3
77.16 CDCl3
76.84 CDCl3
64.32
58.74
58.26
55.55
55.34
52.33
44.02
43.77
40.25
38.83
34.27
33.91
30.86
30.73
25.03
24.80
24.21
19.48
19.18
f1 (ppm)

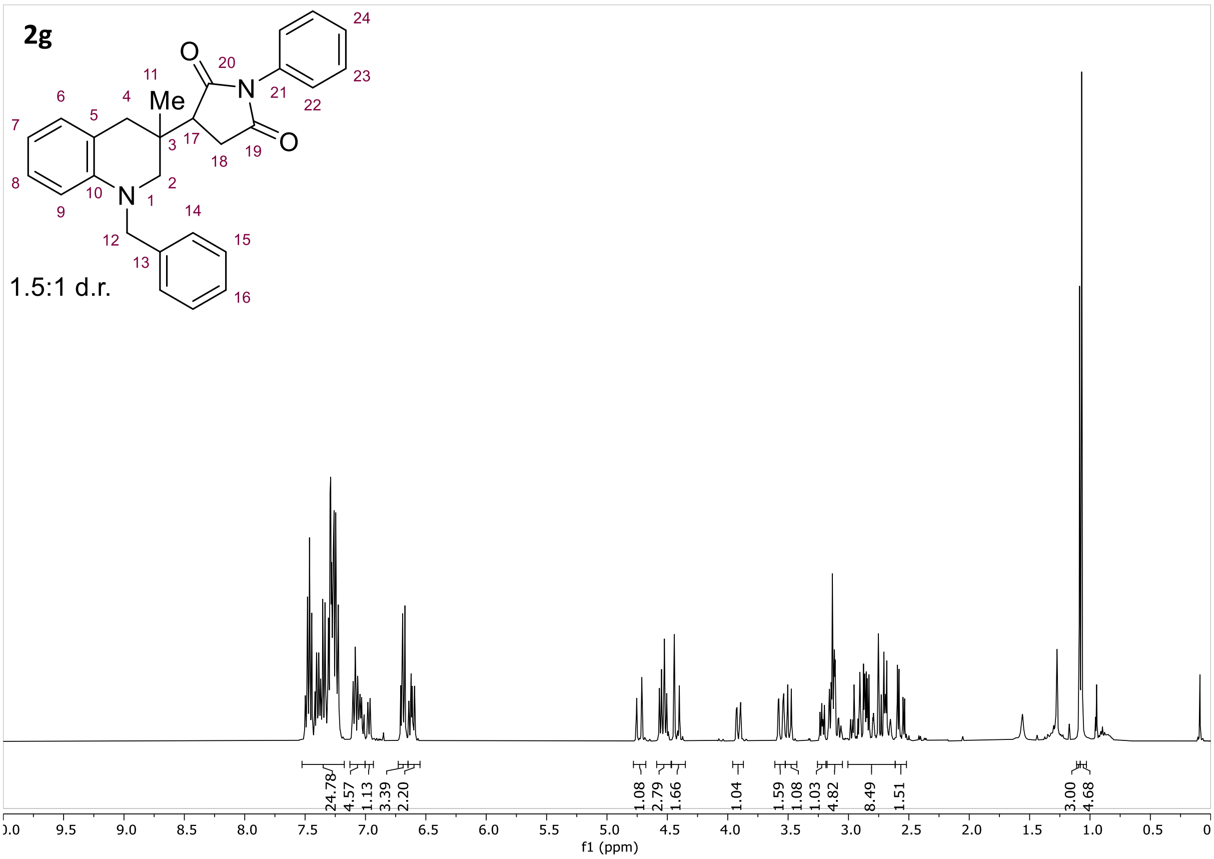
2g
Me
O
N
1.5:1 d.r.
24.78
4.57
1.13
3.39
2.20
1.08
2.79
1.66
1.04
1.59
1.08
1.03
4.82
8.49
1.51
3.00
4.68
f1 (ppm)

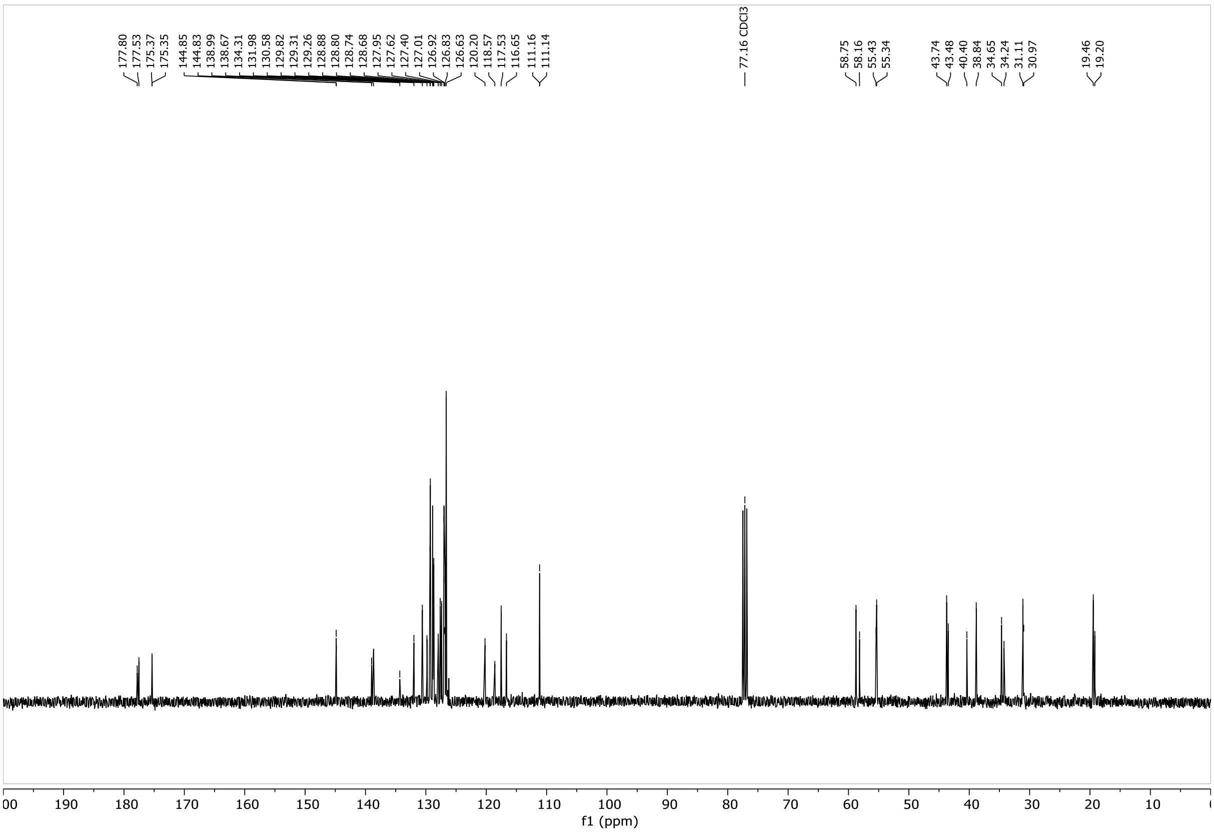
177.80
177.53
175.37
175.35
144.85
144.83
138.99
138.67
134.31
131.98
130.58
129.82
129.31
129.26
128.88
128.80
128.74
128.68
127.95
127.62
127.40
127.01
126.92
126.83
126.63
120.20
118.57
117.53
116.65
111.16
111.14
77.16 CDCl3
58.75
58.16
55.43
55.34
43.74
43.48
40.40
38.84
34.65
34.24
31.11
30.97
19.46
19.20
f1 (ppm)

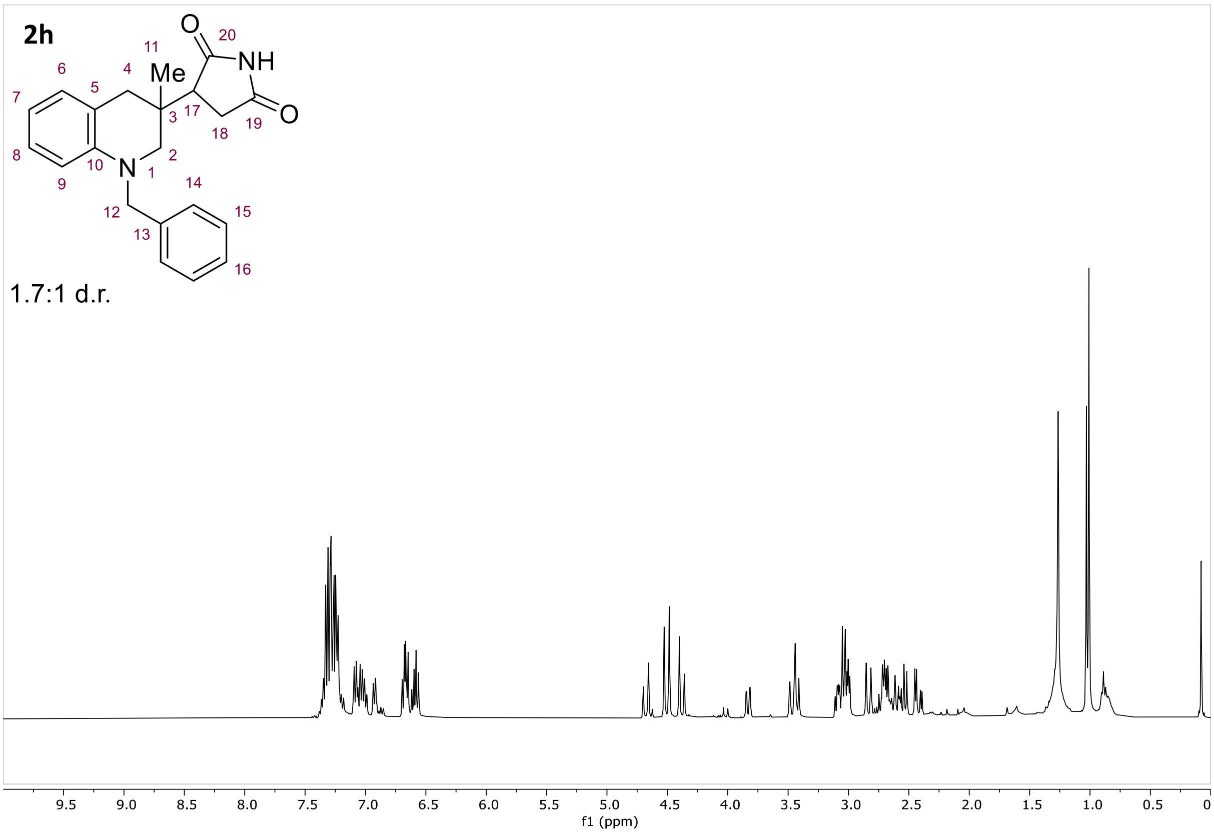
2h
1.7:1 d.r.
Me
NH
O
N
f1 (ppm)

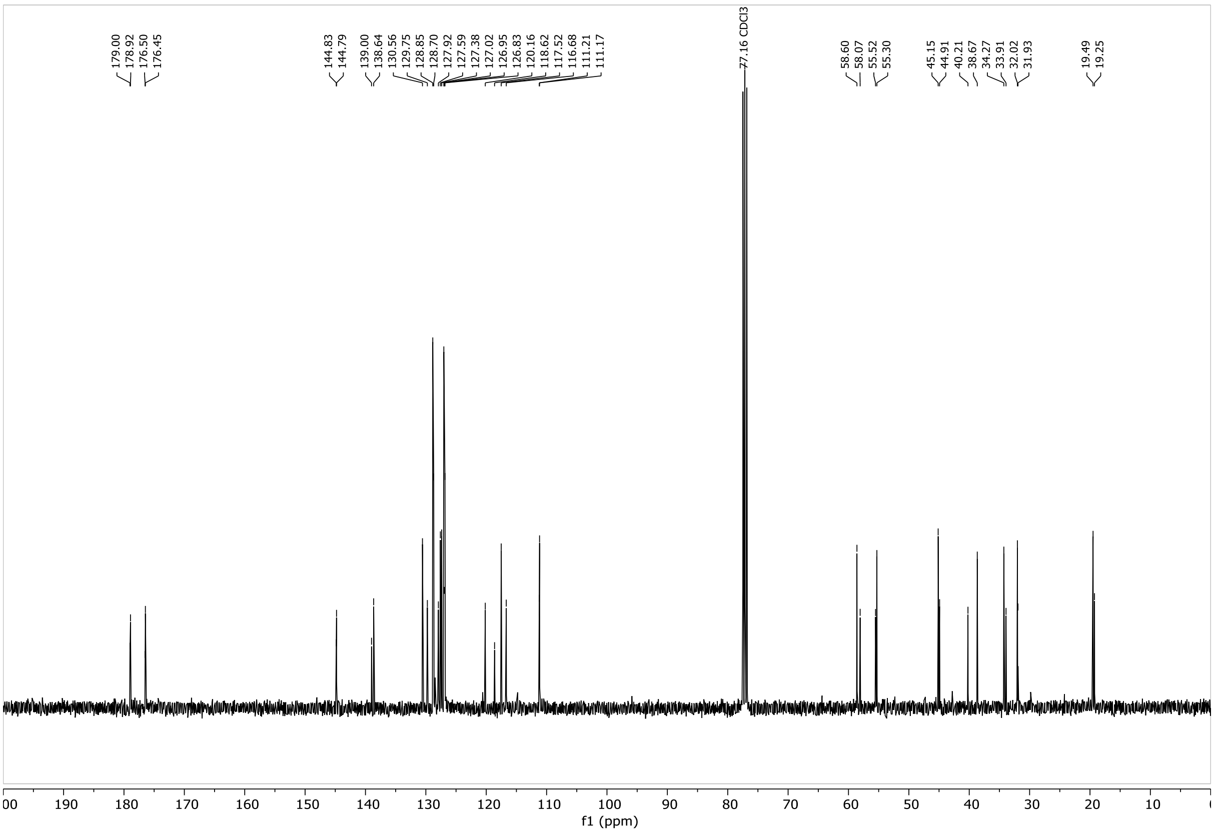
179.00
178.92
176.50
176.45
144.83
144.79
139.00
138.64
130.56
129.75
128.85
128.70
127.92
127.59
127.38
127.02
126.95
126.83
120.16
118.62
117.52
116.68
111.21
111.17
77.16 CDCl3
58.60
58.07
55.52
55.30
45.15
44.91
40.21
38.67
34.27
33.91
32.02
31.93
19.49
19.25
f1 (ppm)

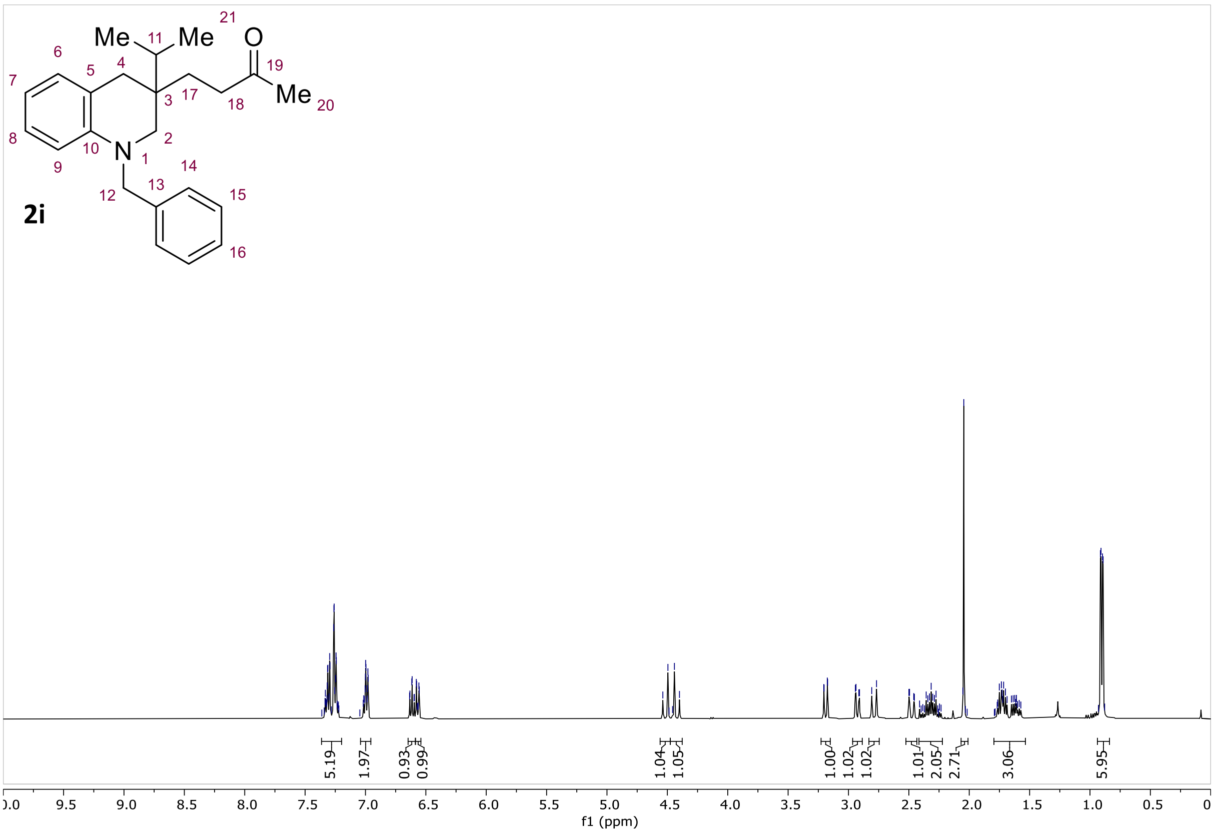

Me
Me
O
Me
N
2i
5.19
1.97
0.93
0.99
1.04
1.05
1.00
1.02
1.02
1.01
2.05
2.71
3.06
5.95
f1 (ppm)

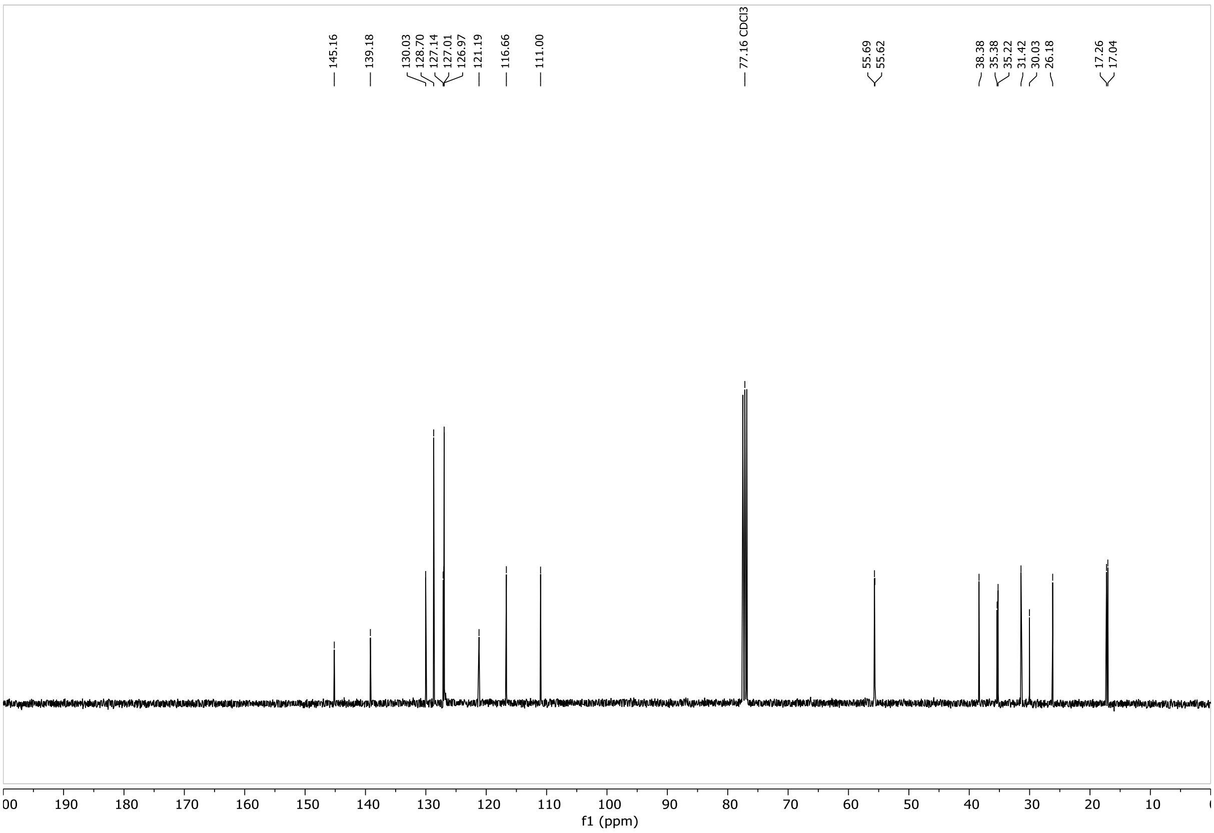

145.16
139.18
130.03
128.70
127.14
127.01
126.97
121.19
116.66
111.00
77.16 CDCl3
55.69
55.62
38.38
35.38
35.22
31.42
30.03
26.18
17.26
17.04
f1 (ppm)

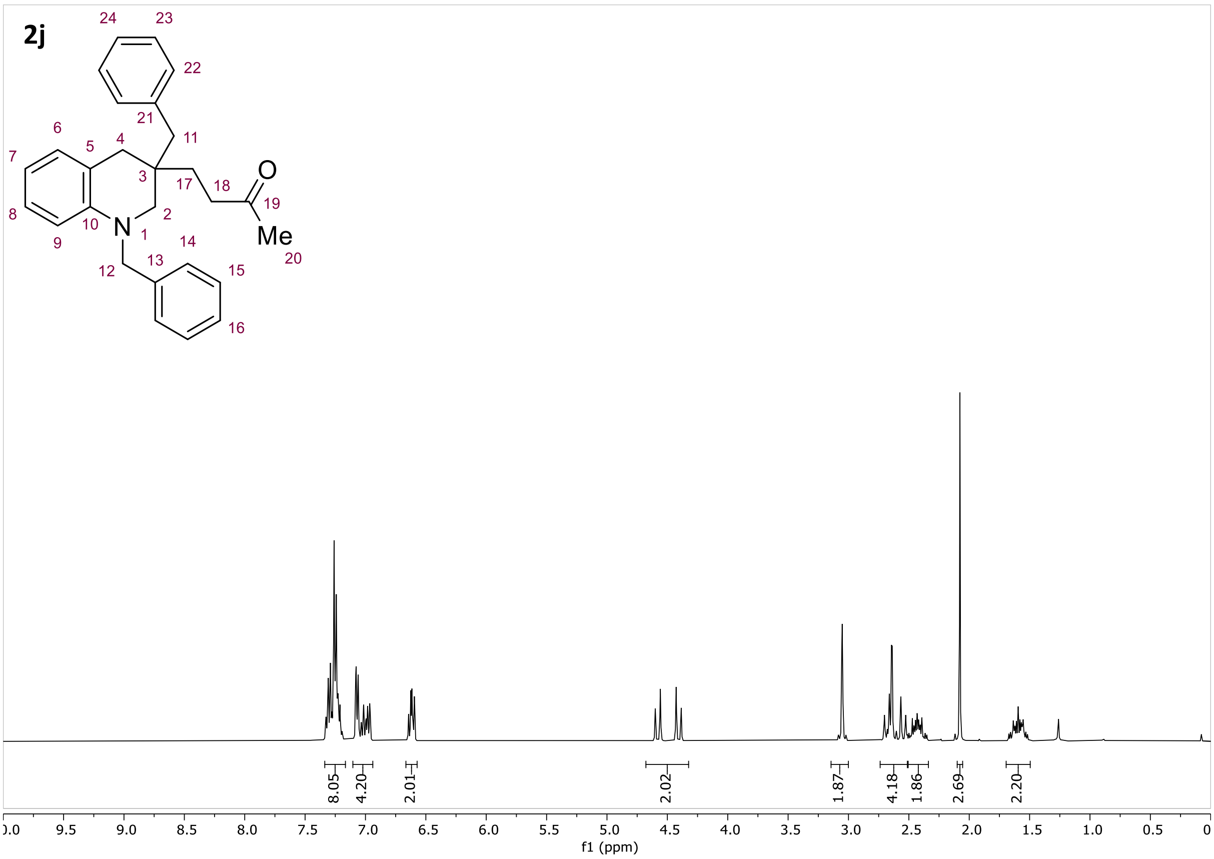
2j
24
23
22
21
11
6
7
5
4
3
17
18
O
19
8
10
2
Me
9
N
1
14
20
12
13
15
16
8.05
4.20
2.01
2.02
1.87
4.18
1.86
2.69
2.20
f1 (ppm)

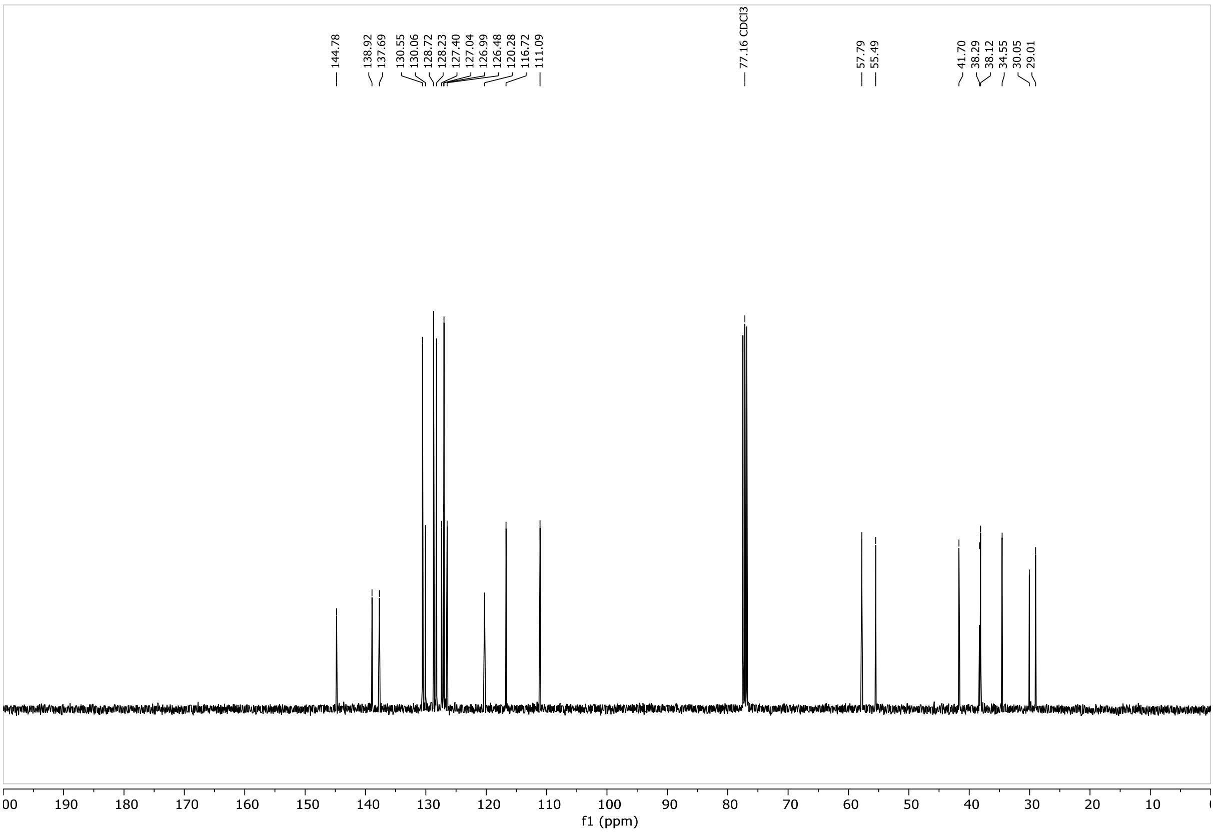
144.78
138.92
137.69
130.55
130.06
128.72
128.23
127.40
127.04
126.99
126.48
120.28
116.72
111.09
77.16 CDCl3
57.79
55.49
41.70
38.29
38.12
34.55
30.05
29.01
f1 (ppm)

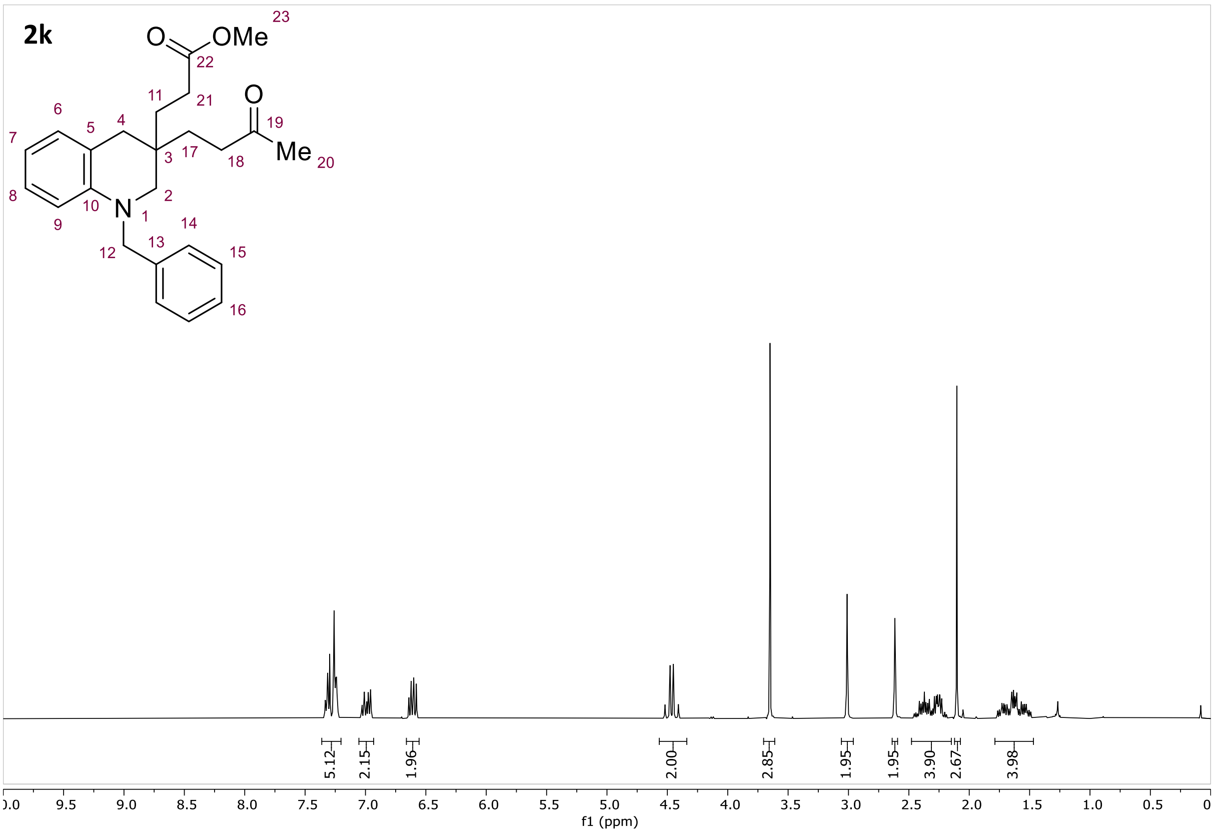
2k
OMe
Me
N
5.12
2.15
1.96
2.00
2.85
1.95
1.95
3.90
2.67
3.98
f1 (ppm)

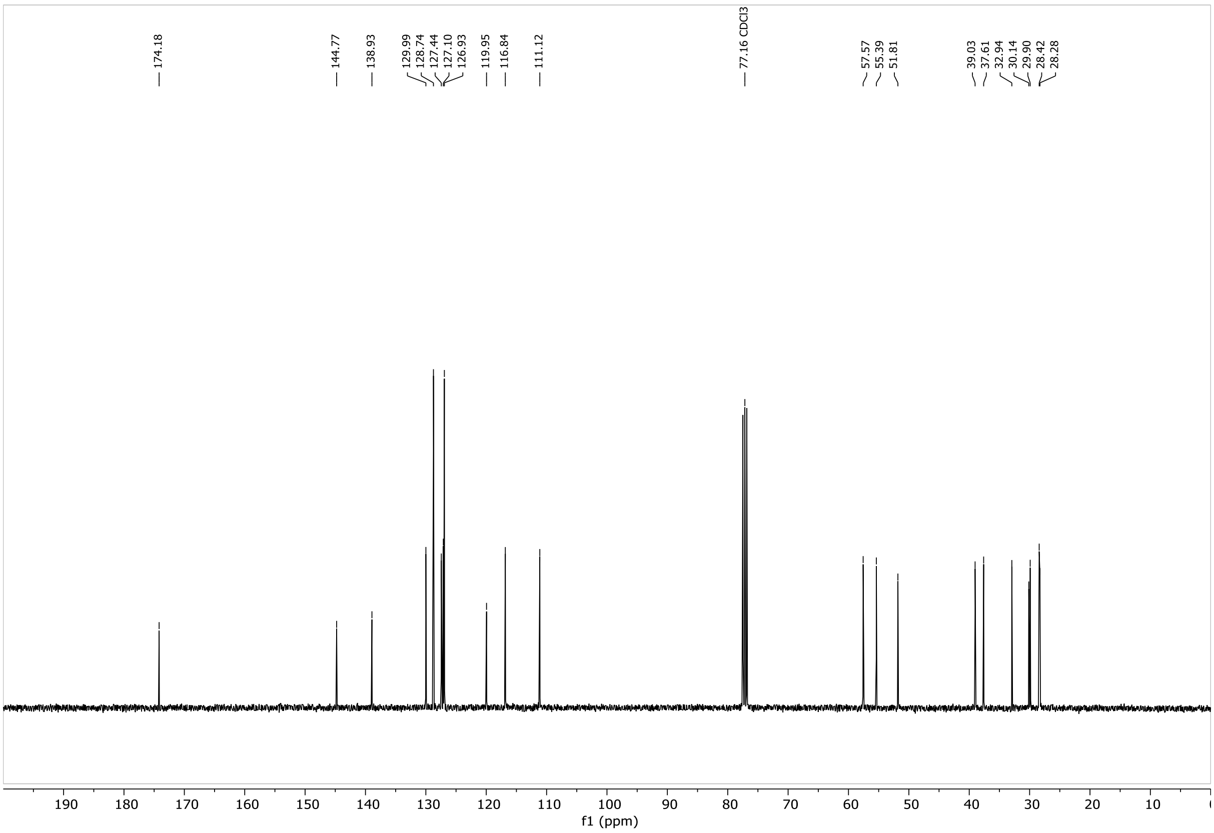
174.18
144.77
138.93
129.99
128.74
127.44
127.10
126.93
119.95
116.84
111.12
77.16 CDCl3
57.57
55.39
51.81
39.03
37.61
32.94
30.14
29.90
28.42
28.28
f1 (ppm)

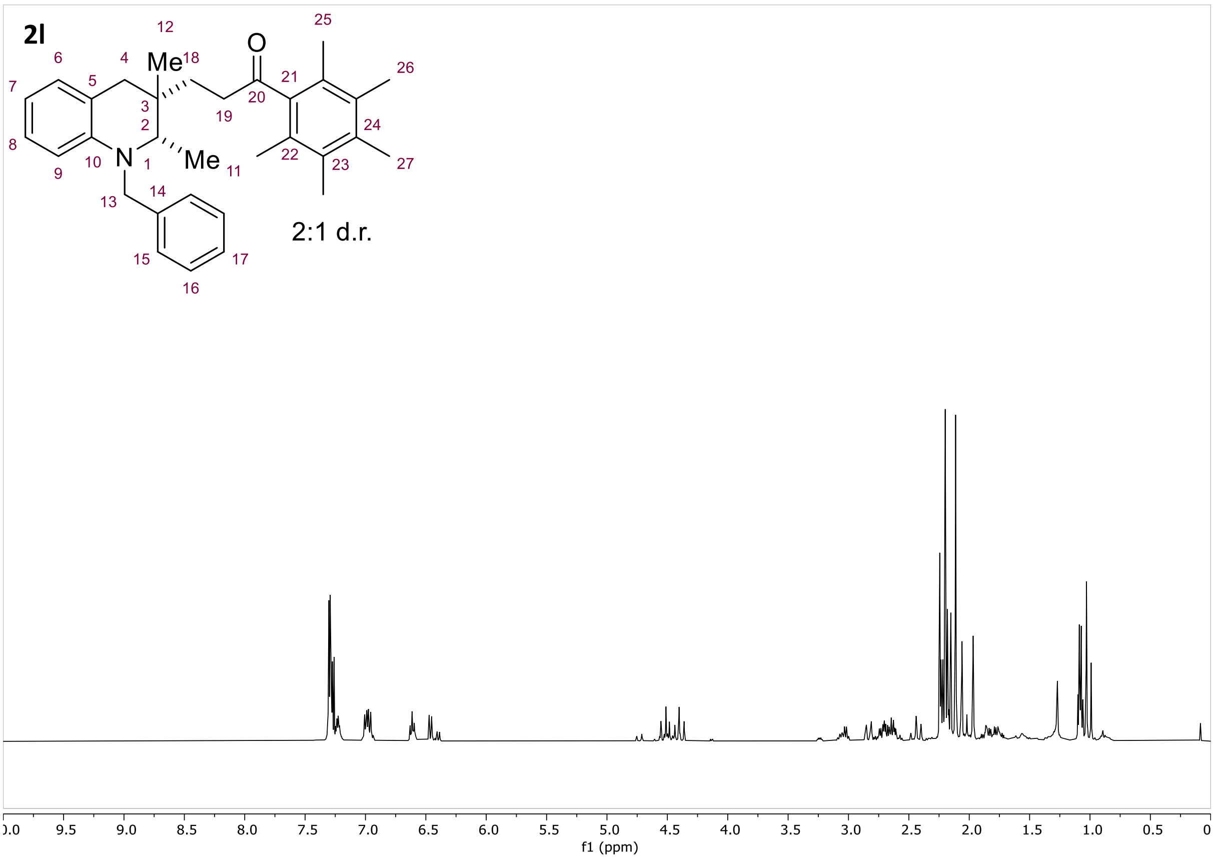
2l
2:1 d.r.
f1 (ppm)

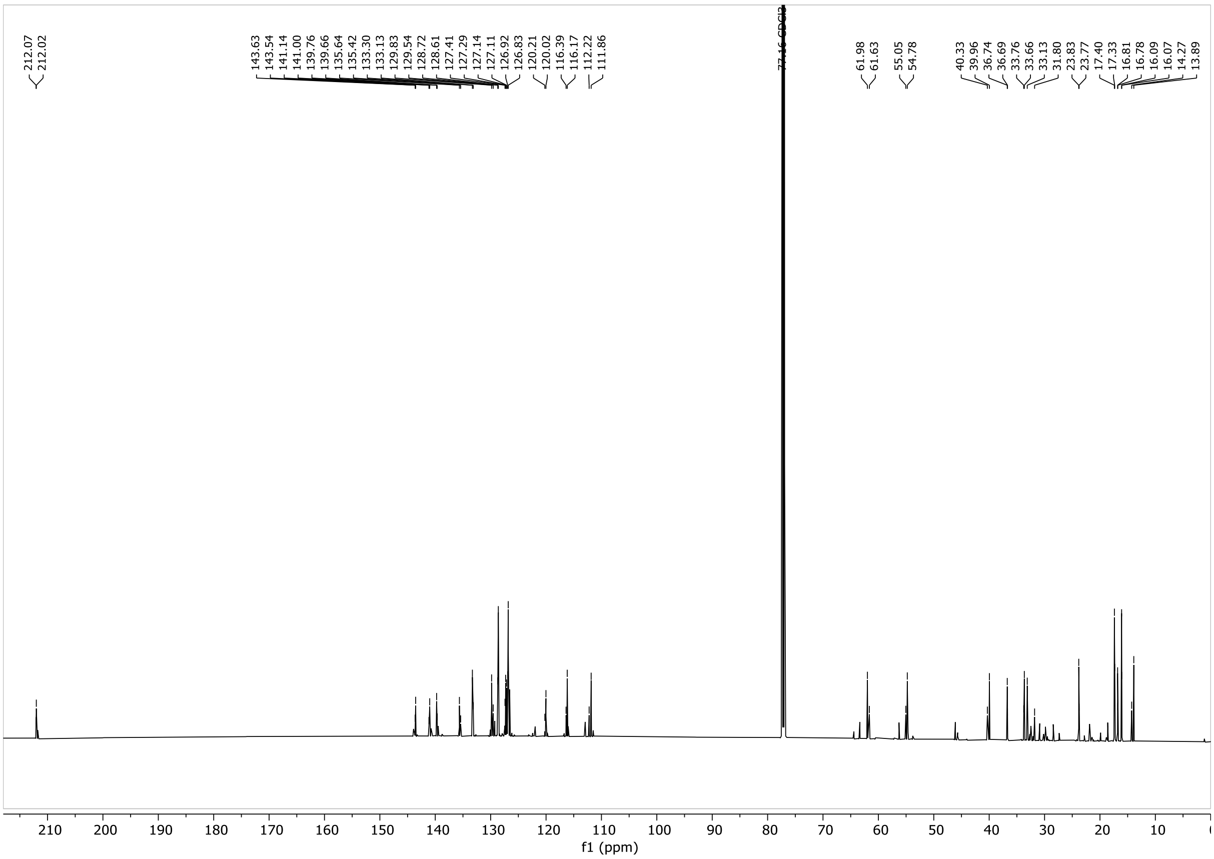
212.07
212.02
143.63
143.54
141.14
141.00
139.76
139.66
135.64
135.42
133.30
133.13
129.83
129.54
128.72
128.61
127.41
127.29
127.14
127.11
126.92
126.83
120.21
120.02
116.39
116.17
112.22
111.86
61.98
61.63
55.05
54.78
40.33
39.96
36.74
36.69
33.76
33.66
33.13
31.80
23.83
23.77
17.40
17.33
16.81
16.78
16.09
16.07
14.27
13.89
f1 (ppm)

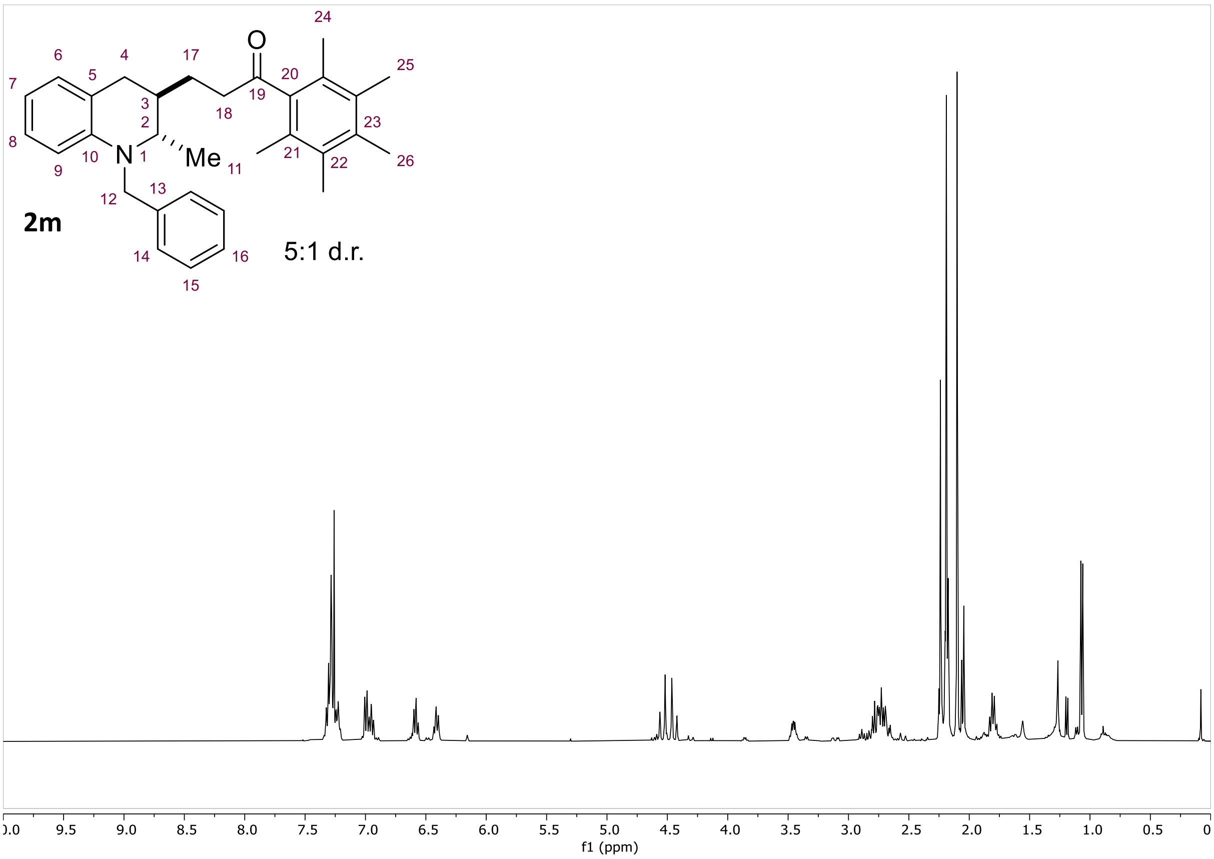
2m
5:1 d.r.
f1 (ppm)

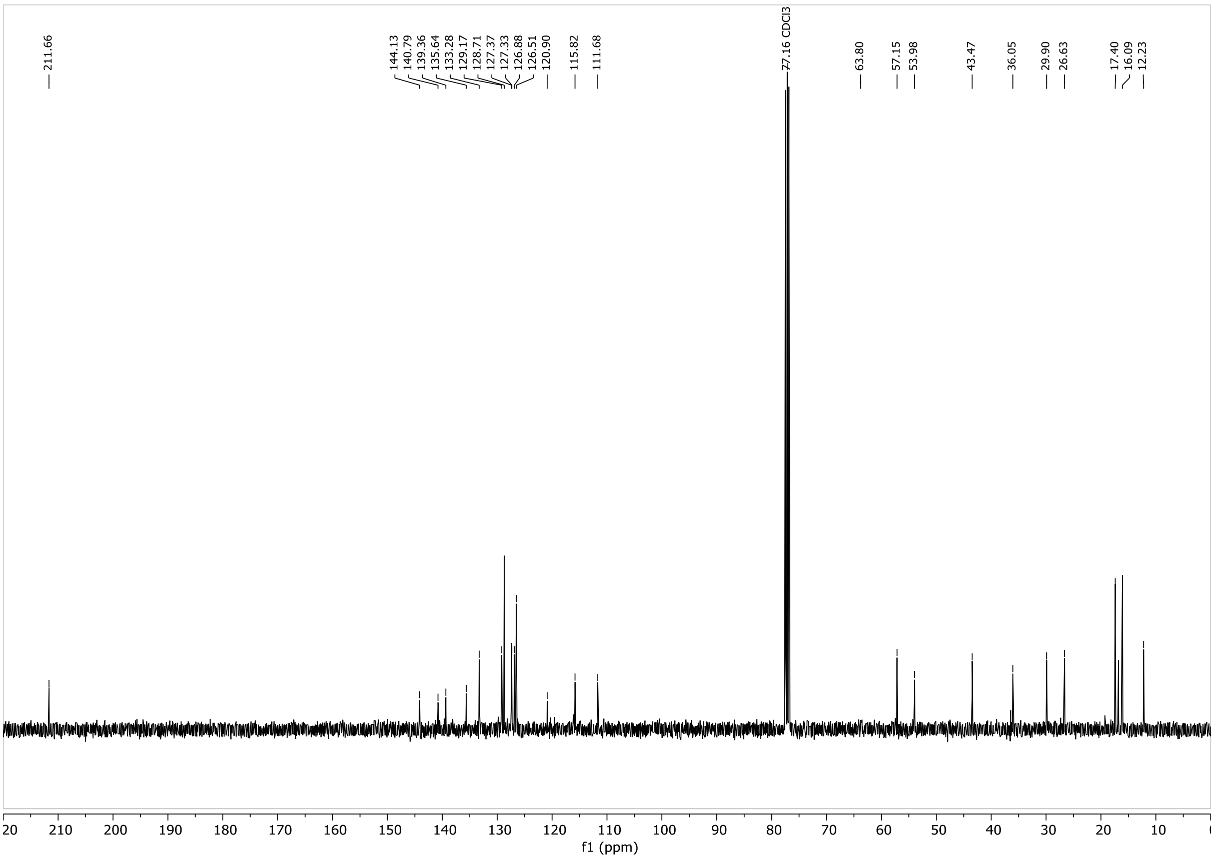
211.66
144.13
140.79
139.36
135.64
133.28
129.17
128.71
127.37
127.33
126.88
126.51
120.90
115.82
111.68
77.16 CDCl3
63.80
57.15
53.98
43.47
36.05
29.90
26.63
17.40
16.09
12.23
f1 (ppm)

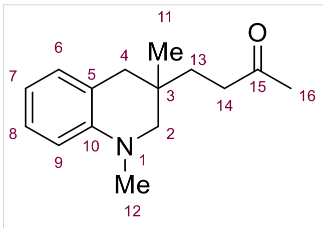
Me
O
Me
N
1
2
3
4
5
6
7
8
9
10
11
12
13
14
15
16

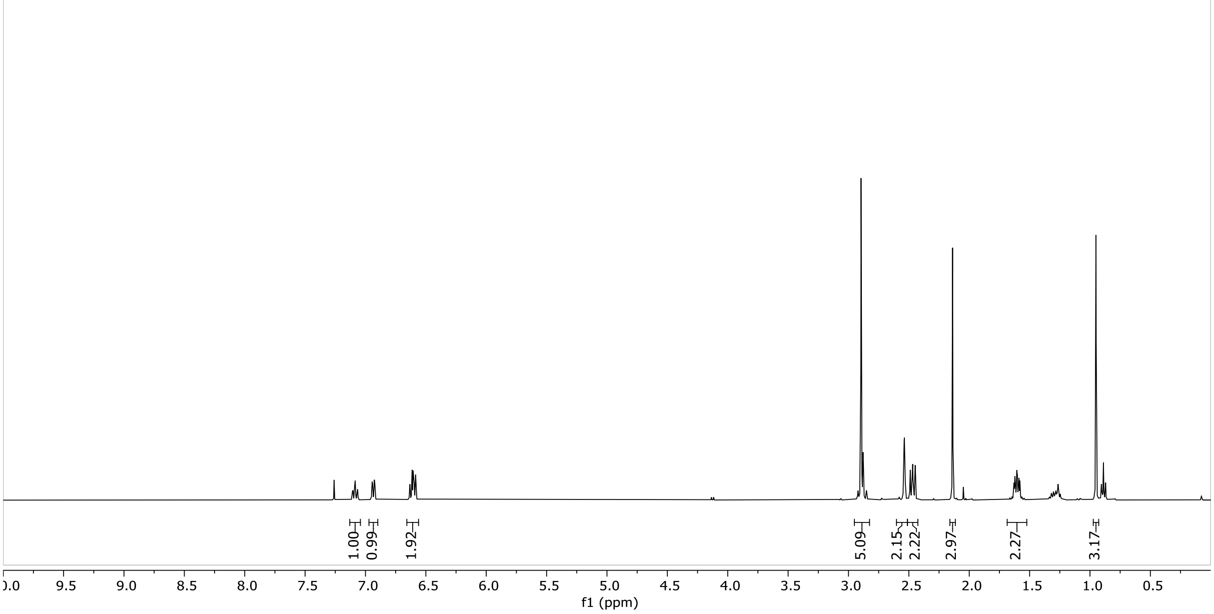
1.00
0.99
1.92
5.09
2.15
2.22
2.97
2.27
3.17
f1 (ppm)

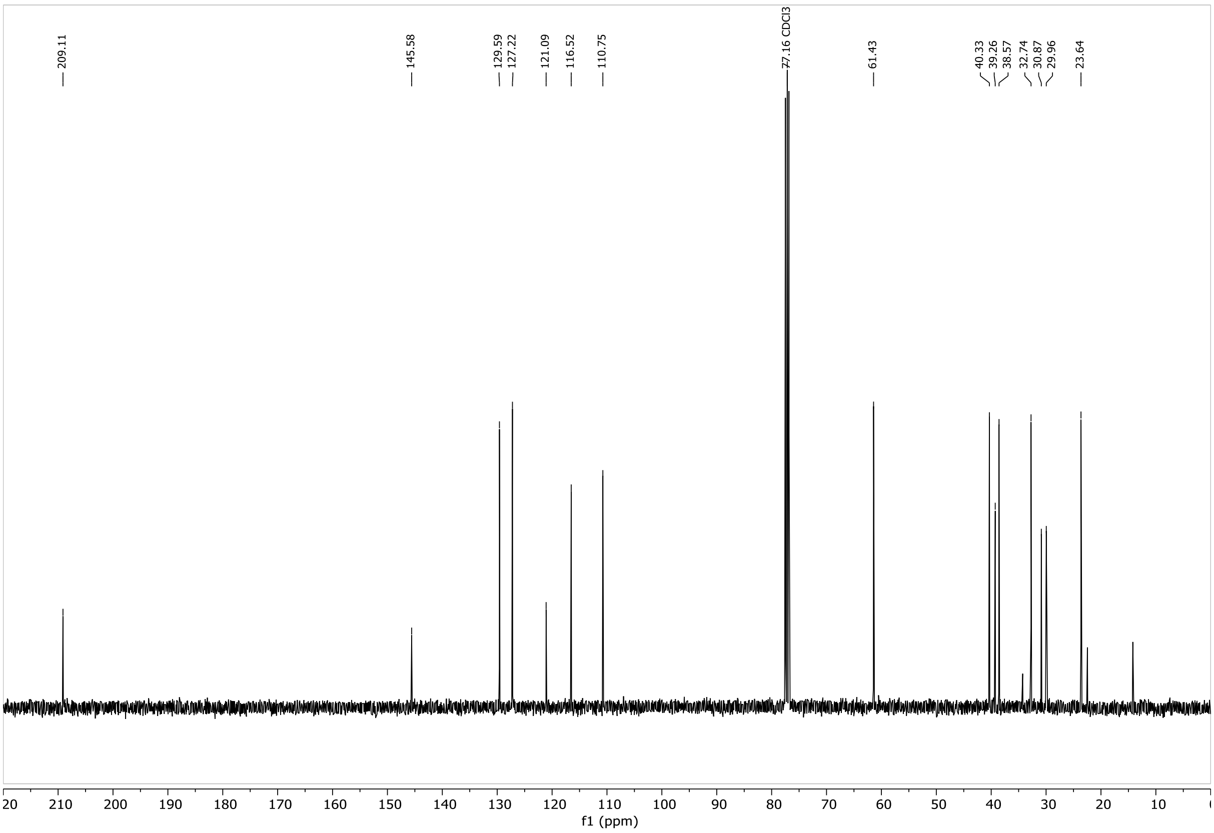
209.11
145.58
129.59
127.22
121.09
116.52
110.75
77.16 CDCl3
61.43
40.33
39.26
38.57
32.74
30.87
29.96
23.64
f1 (ppm)

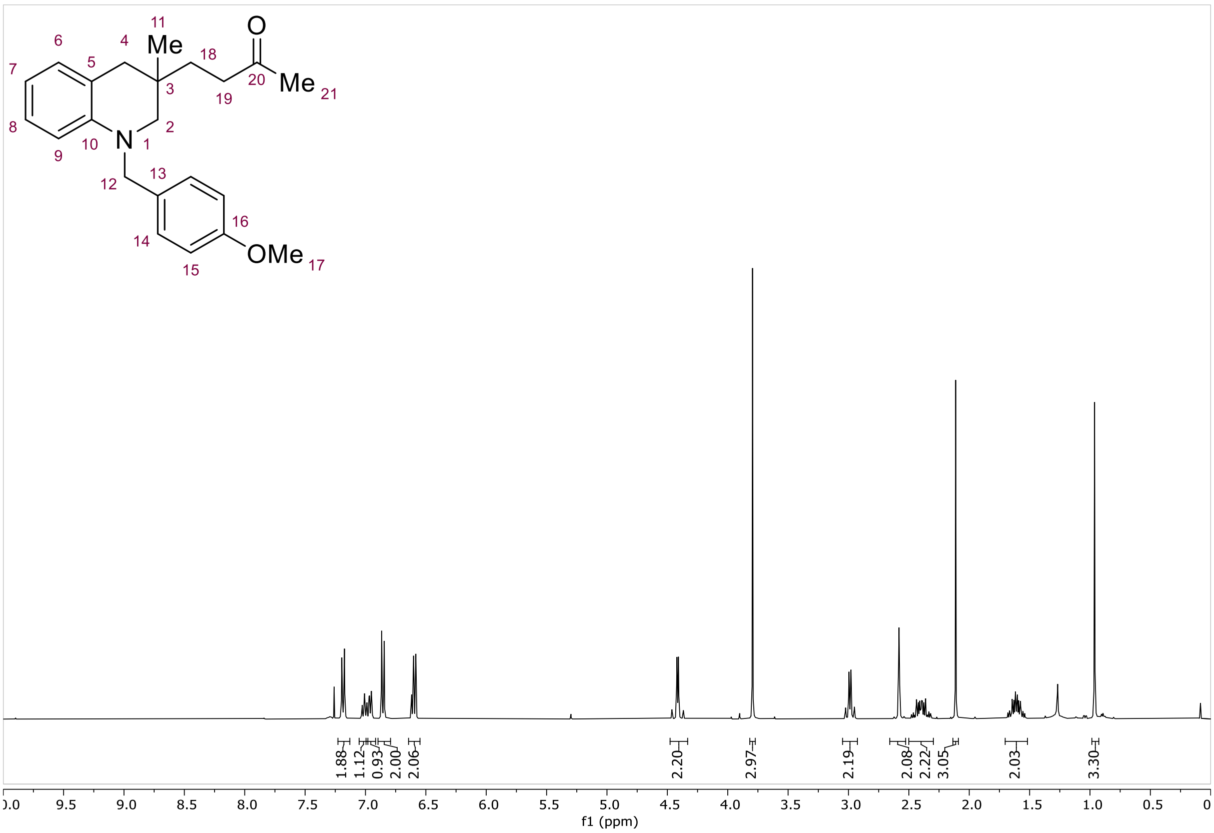
Me
O
Me
N
OMe
1.88
1.12
0.93
2.00
2.06
2.20
2.97
2.19
2.08
2.22
3.05
2.03
3.30
f1 (ppm)

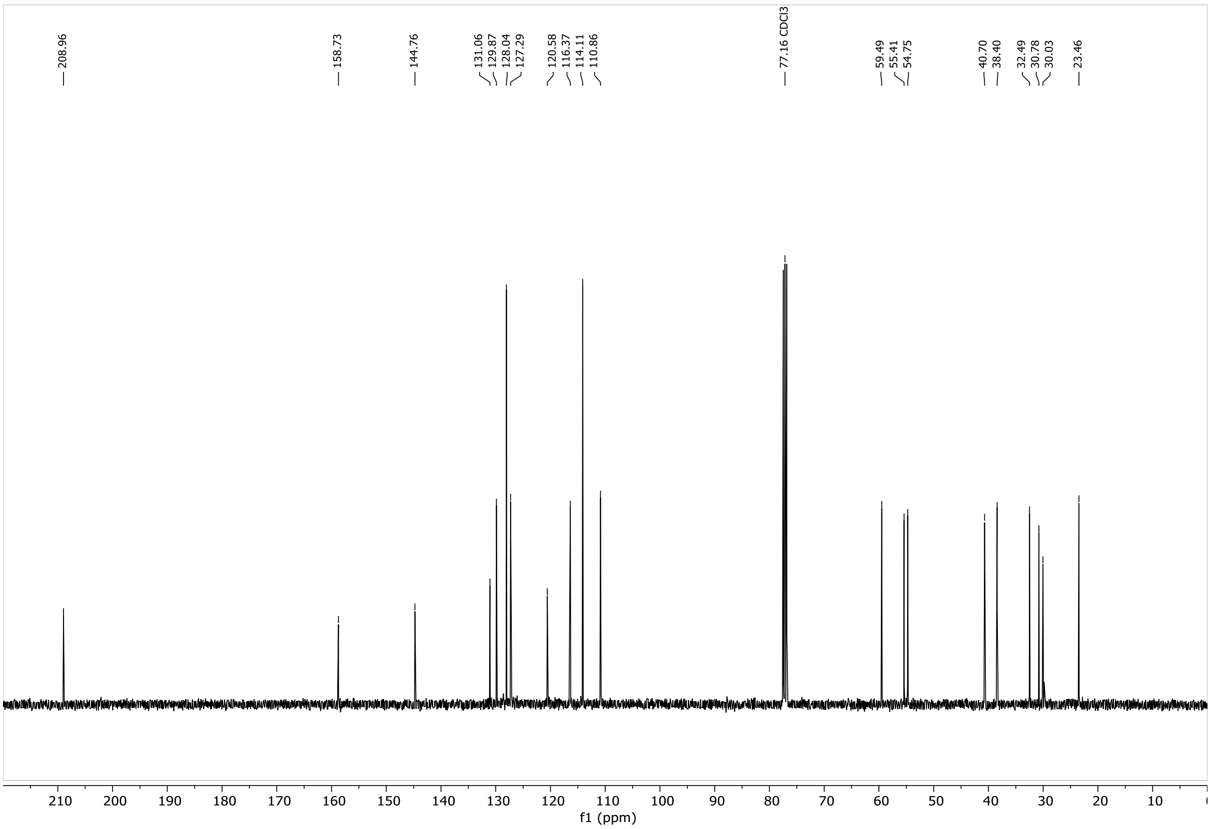
208.96
158.73
144.76
131.06
129.87
128.04
127.29
120.58
116.37
114.11
110.86
77.16 CDCl3
59.49
55.41
54.75
40.70
38.40
32.49
30.78
30.03
23.46
f1 (ppm)

2p

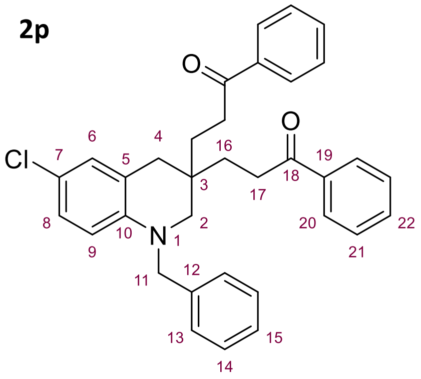

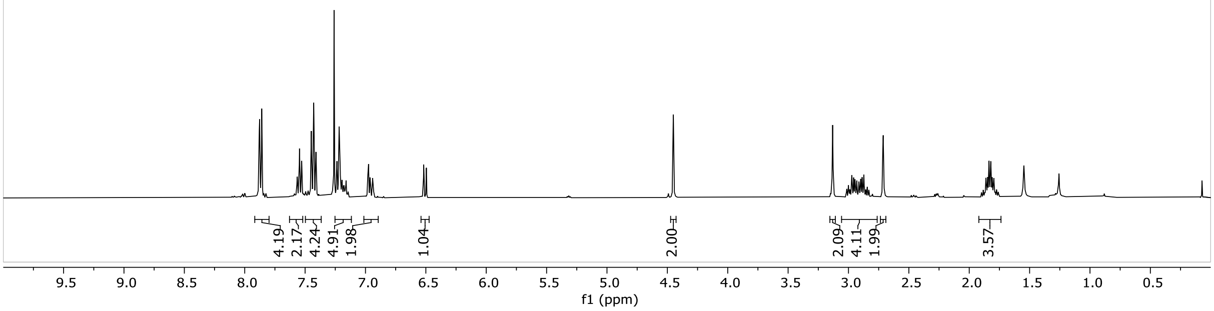
4.19
2.17
4.24
4.91
1.98
1.04
2.00
2.09
4.11
1.99
3.57
9.5 9.0 8.5 8.0 7.5 7.0 6.5 6.0 5.5 5.0 4.5 4.0 3.5 3.0 2.5 2.0 1.5 1.0 0.5
f1 (ppm)

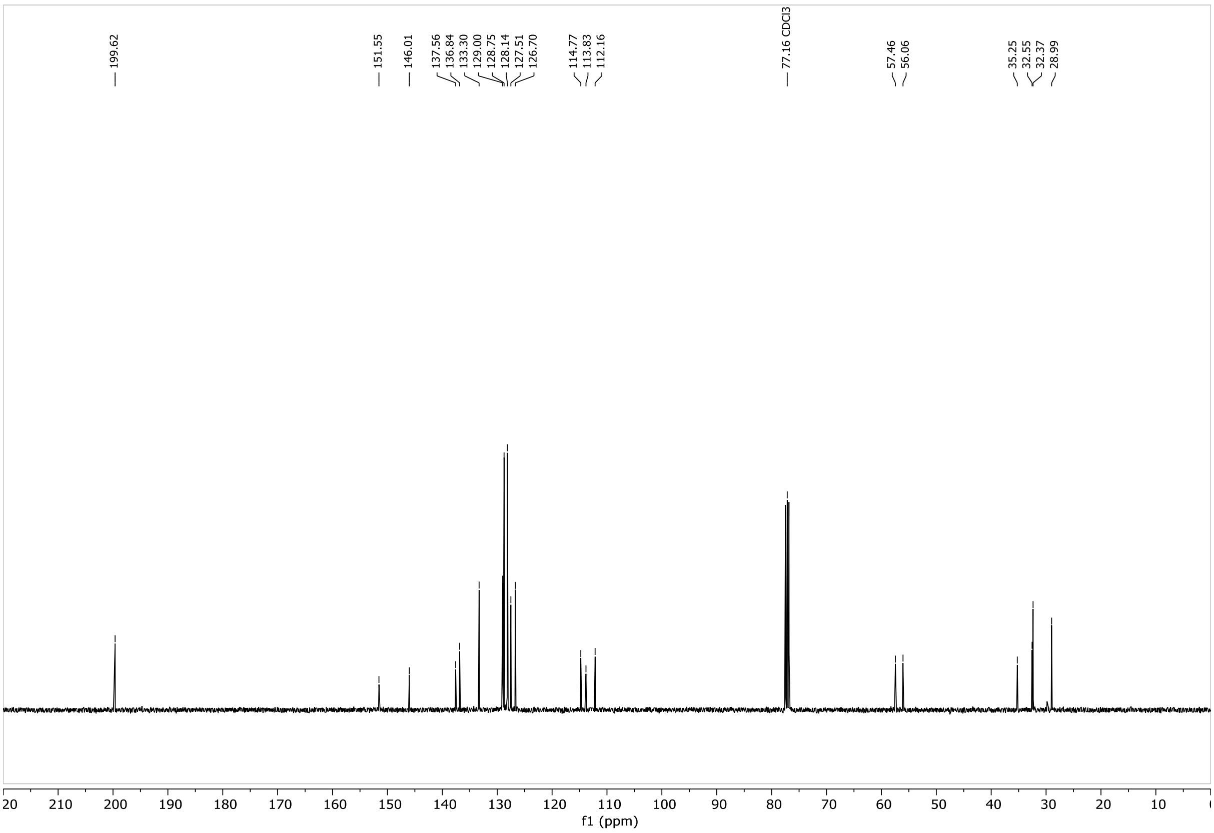
199.62
151.55
146.01
137.56
136.84
133.30
129.00
128.75
128.14
127.51
126.70
114.77
113.83
112.16
77.16 CDCl3
57.46
56.06
35.25
32.55
32.37
28.99
210 200 190 180 170 160 150 140 130 120 110 100 90 80 70 60 50 40 30 20 10
f1 (ppm)

2q

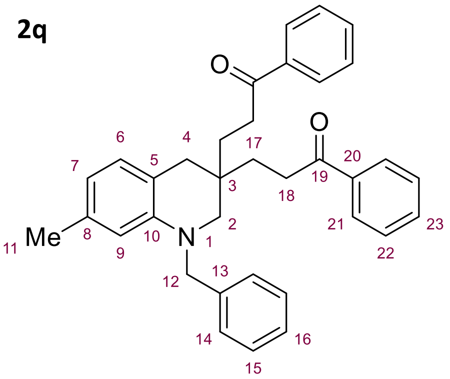

O
O
Me
N
1
2
3
4
5
6
7
8
9
10
11
12
13
14
15
16
17
18
19
20
21
22
23

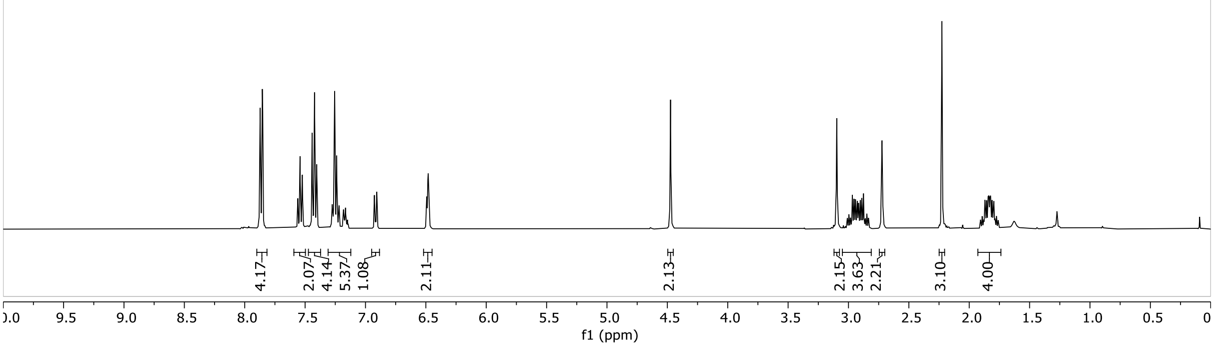

4.17
2.07
4.14
5.37
1.08
2.11
2.13
2.15
3.63
2.21
3.10
4.00
f1 (ppm)

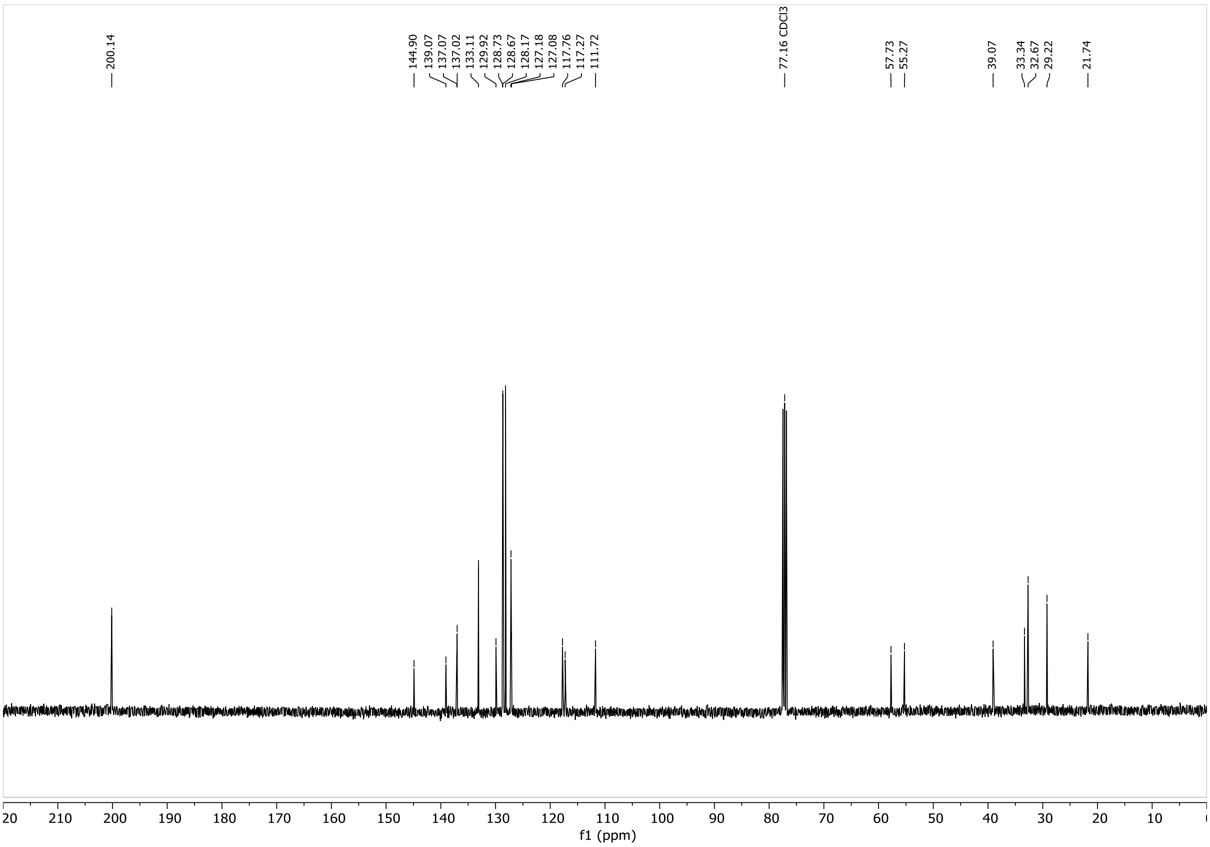

200.14
144.90
139.07
137.07
137.02
133.11
129.92
128.73
128.67
128.17
127.18
127.08
117.76
117.27
111.72
77.16 CDCl3
57.73
55.27
39.07
33.34
32.67
29.22
21.74
f1 (ppm)

2r

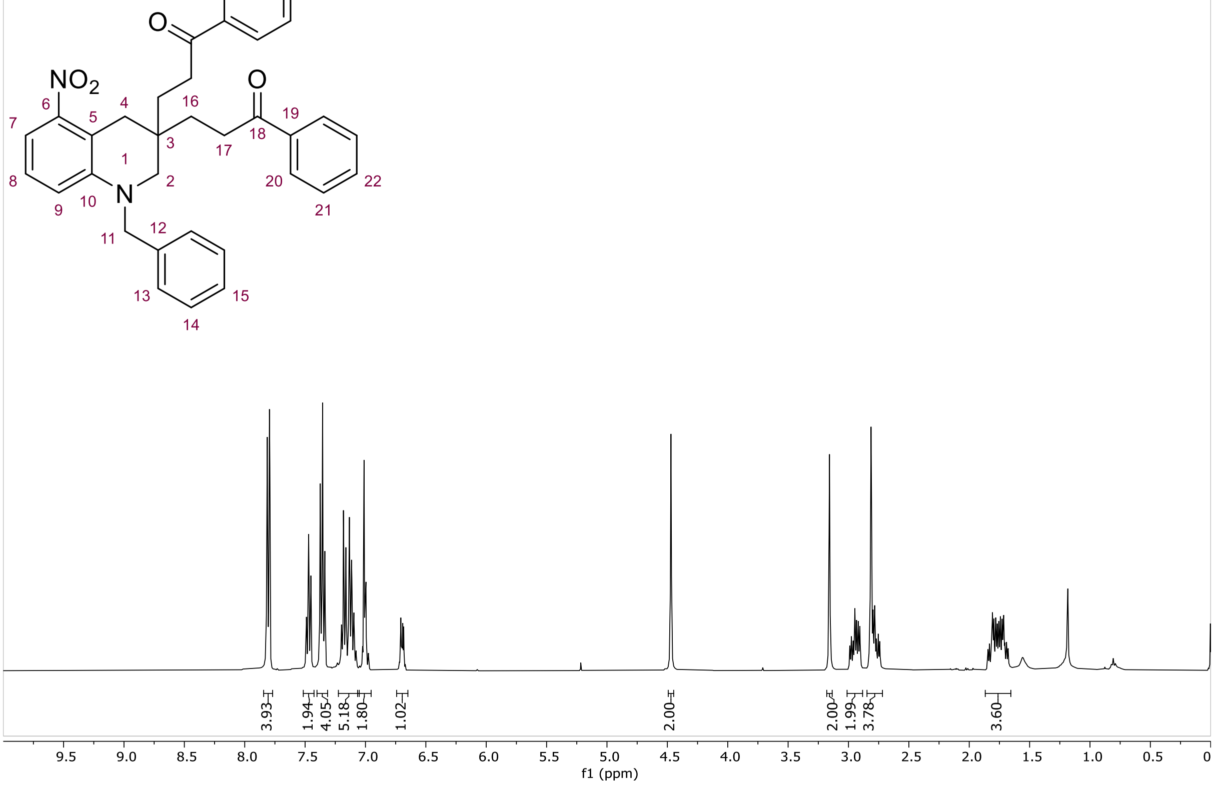
NO2
O
O
1 2 3 4 5 6 7 8 9 10 11 12 13 14 15 16 17 18 19 20 21 22
N
3.93
1.94
4.05
5.18
1.80
1.02
2.00
2.00
1.99
3.78
3.60
9.5 9.0 8.5 8.0 7.5 7.0 6.5 6.0 5.5 5.0 4.5 4.0 3.5 3.0 2.5 2.0 1.5 1.0 0.5 0
f1 (ppm)

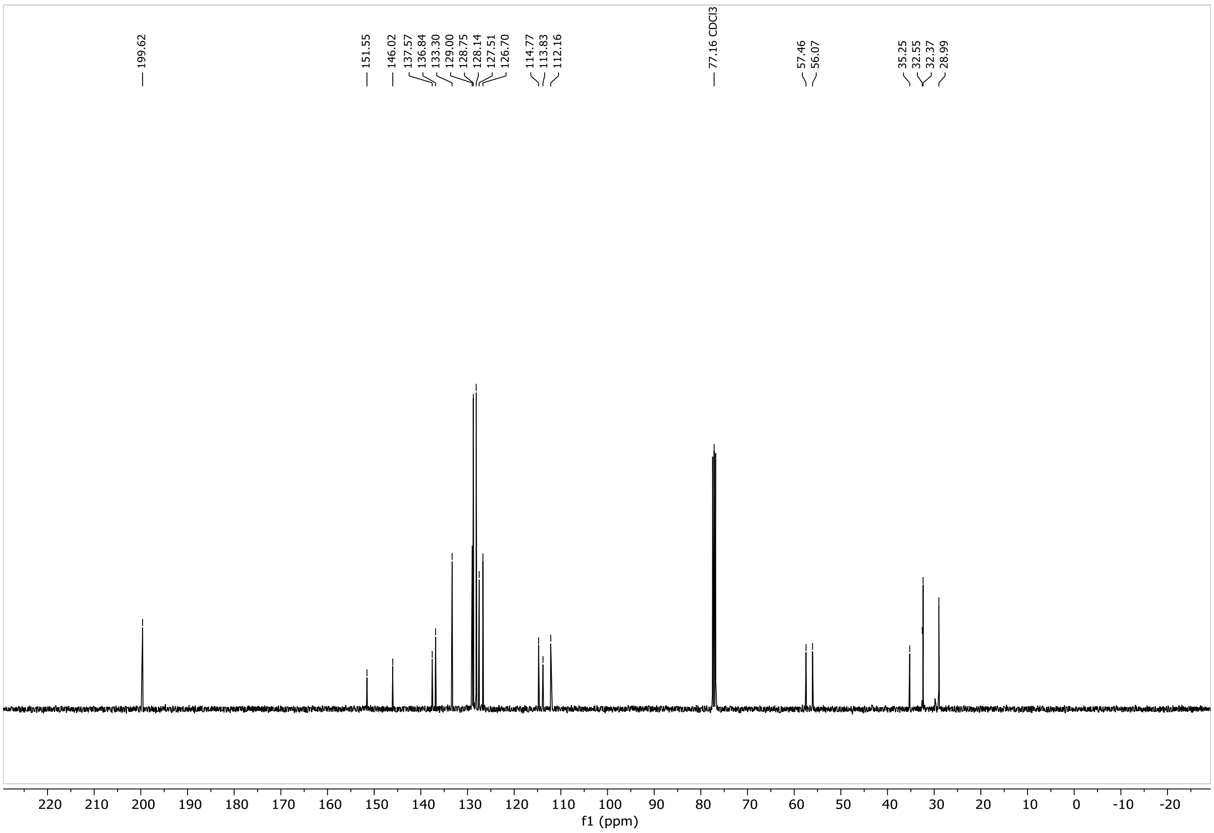
199.62
151.55
146.02
137.57
136.84
133.30
129.00
128.75
128.14
127.51
126.70
114.77
113.83
112.16
77.16 CDCl3
57.46
56.07
35.25
32.55
32.37
28.99
220 210 200 190 180 170 160 150 140 130 120 110 100 90 80 70 60 50 40 30 20 10 0 -10 -20
f1 (ppm)

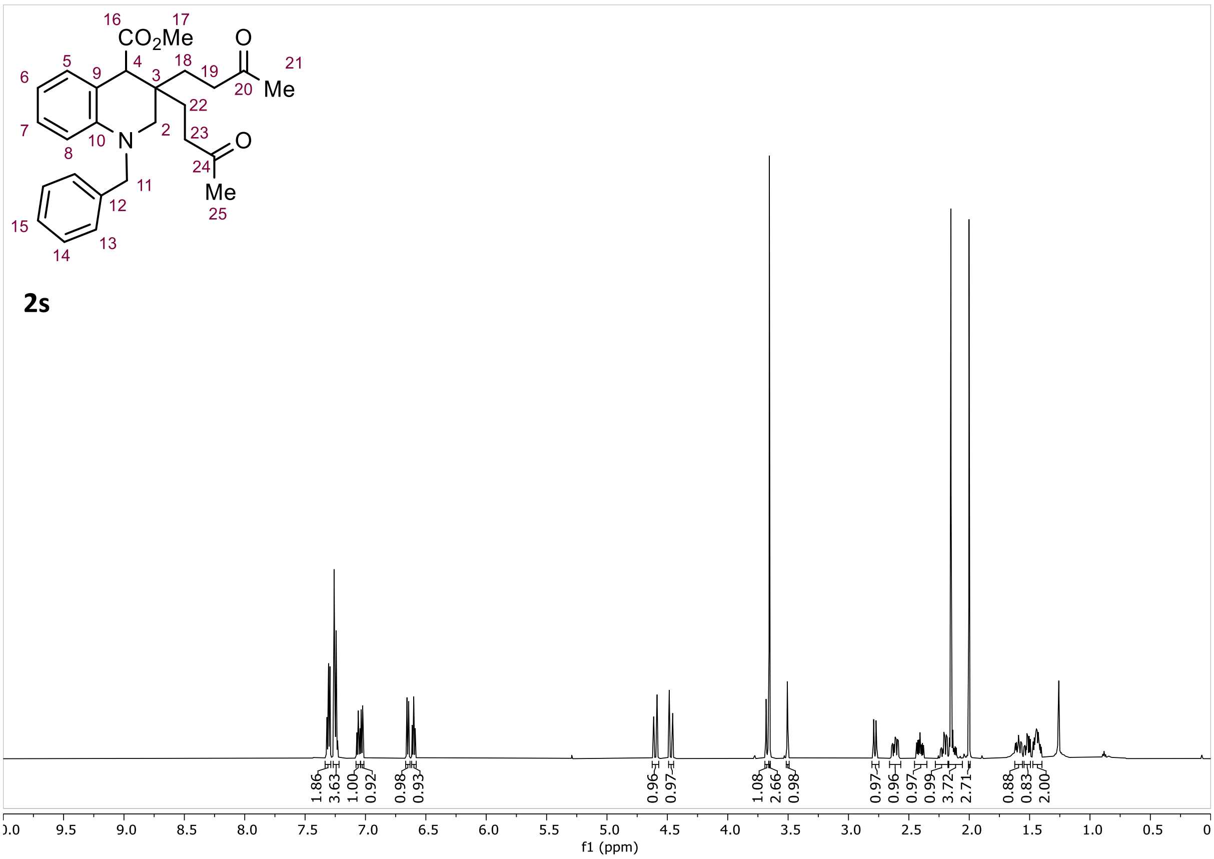

2s
1.86
3.63
1.00
0.92
0.98
0.93
0.96
0.97
1.08
2.66
0.98
0.97
0.96
0.97
0.99
3.72
2.71
0.88
0.83
2.00
f1 (ppm)

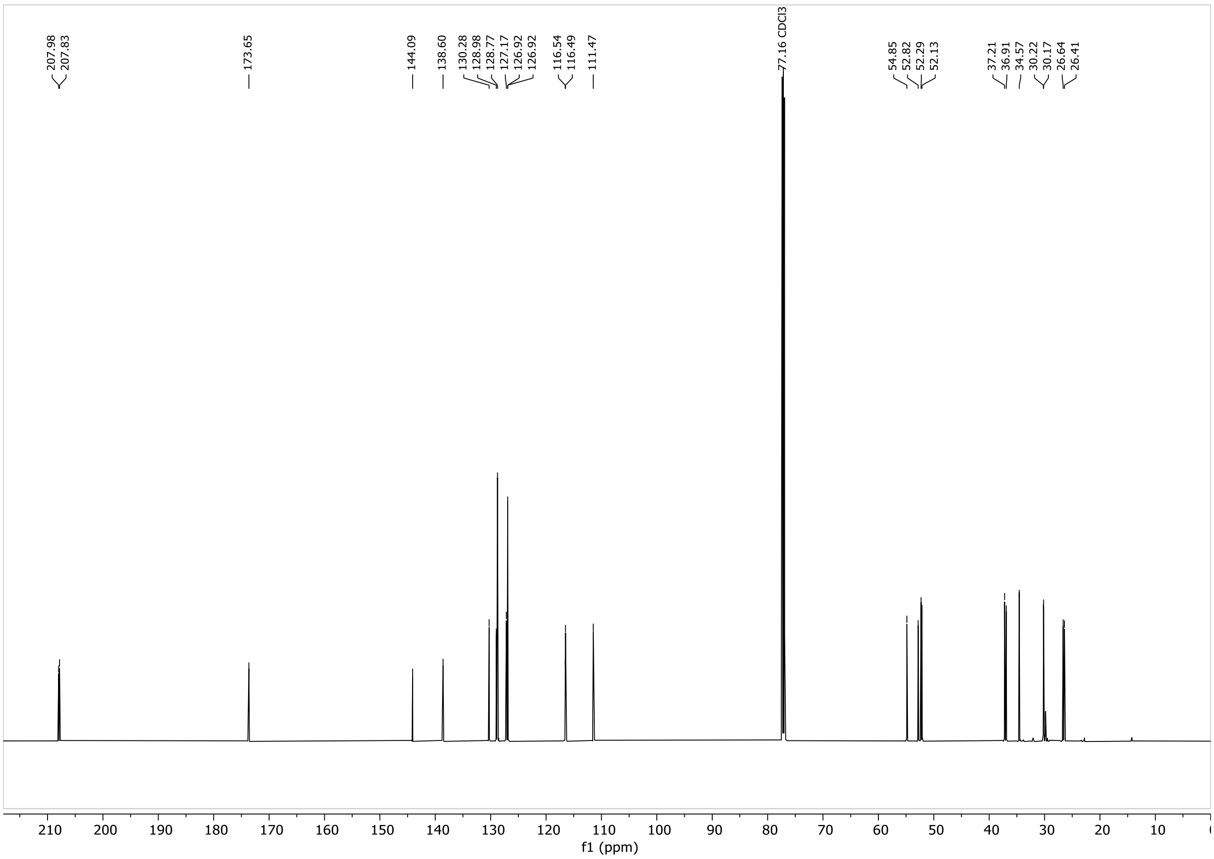

207.98
207.83
173.65
144.09
138.60
130.28
128.98
128.77
127.17
126.92
126.92
116.54
116.49
111.47
77.16 CDCl3
54.85
52.82
52.29
52.13
37.21
36.91
34.57
30.22
30.17
26.64
26.41
f1 (ppm)

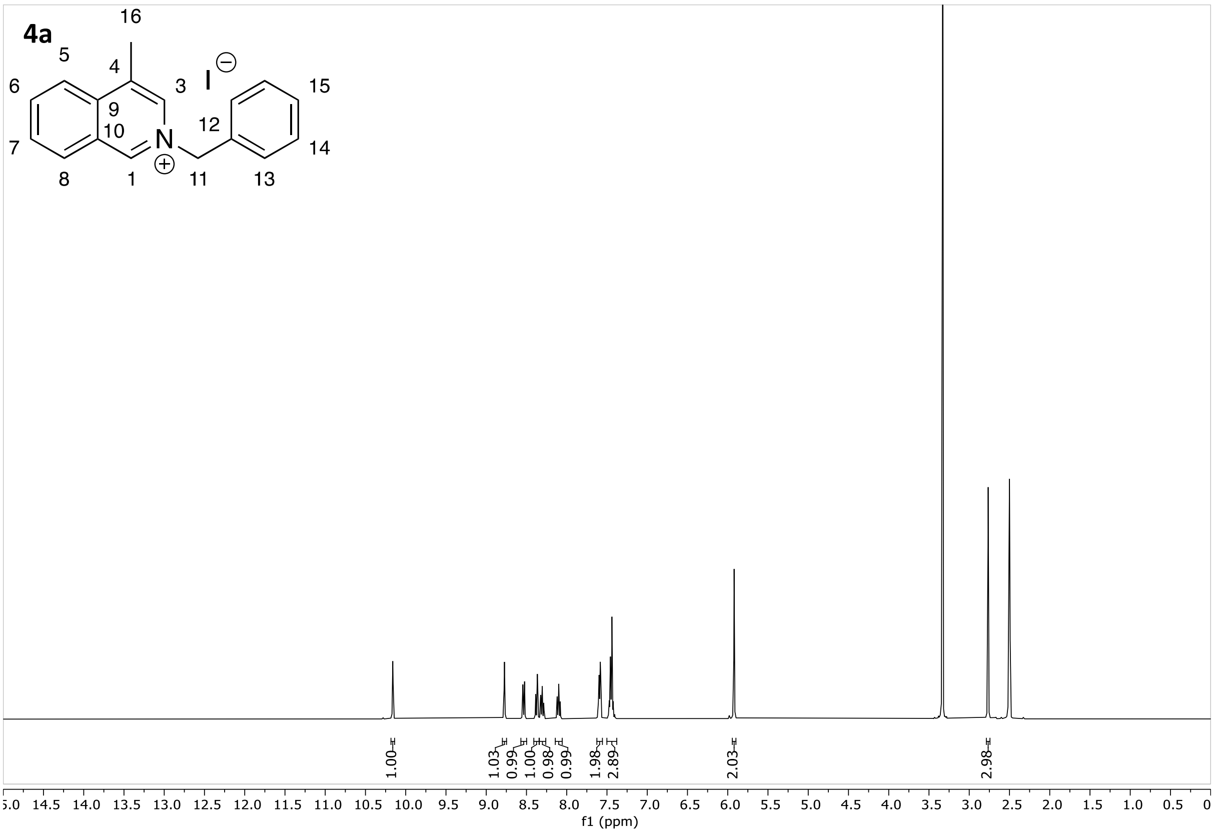
4a
16
5
4
6
3
9
10
N
12
15
7
14
8
1
11
13
I
1.00
1.03
0.99
1.00
0.98
0.99
1.98
2.89
2.03
2.98
f1 (ppm)

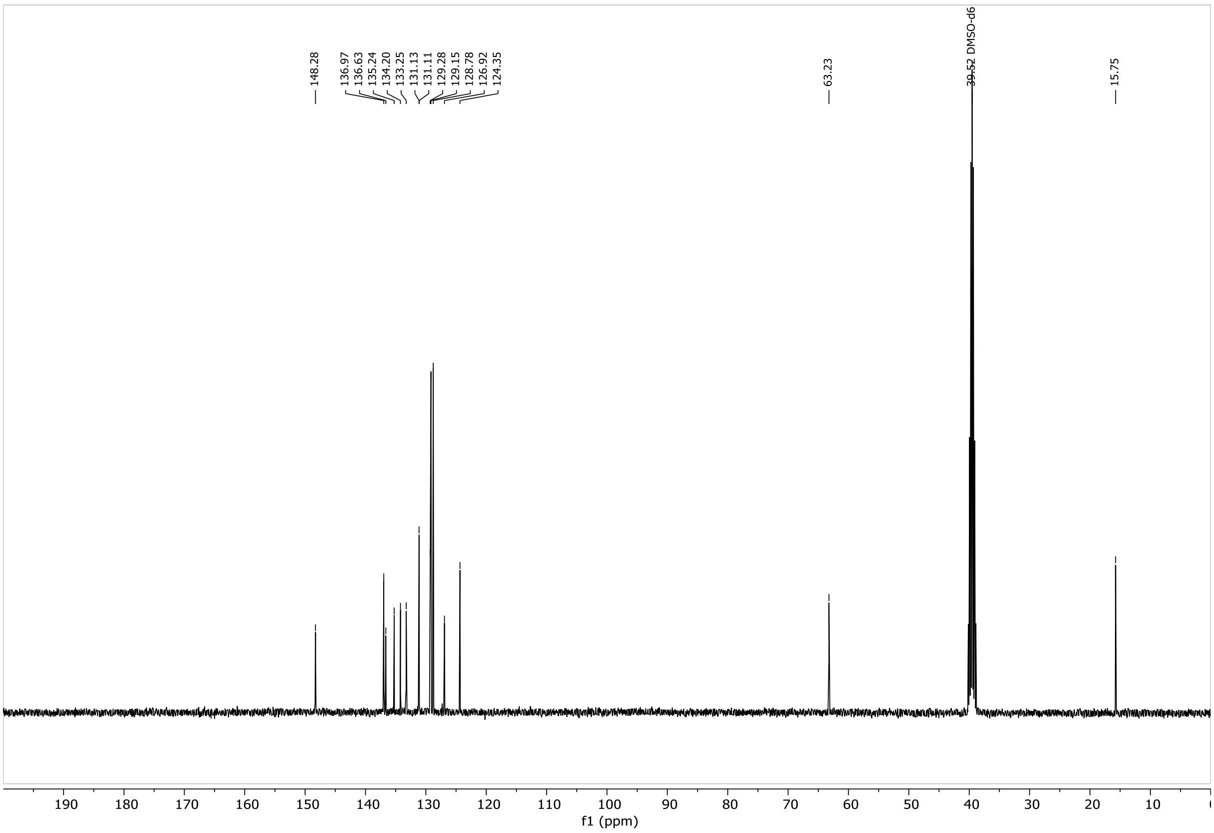
148.28
136.97
136.63
135.24
134.20
133.25
131.13
131.11
129.28
129.15
128.78
126.92
124.35
63.23
39.52 DMSO-d6
15.75
f1 (ppm)

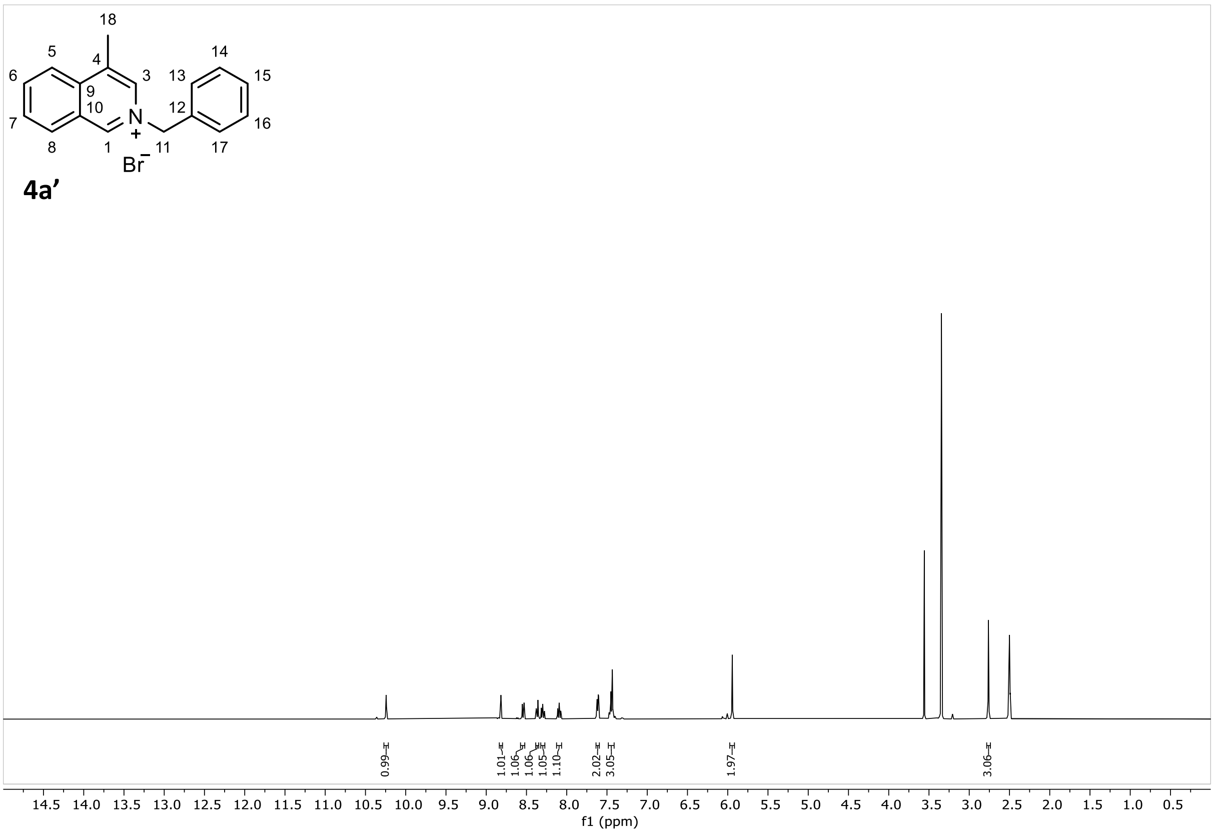
4a’
Br⁻
0.99
1.01
1.06
1.06
1.05
1.10
2.02
3.05
1.97
3.06
14.5 14.0 13.5 13.0 12.5 12.0 11.5 11.0 10.5 10.0 9.5 9.0 8.5 8.0 7.5 7.0 6.5 6.0 5.5 5.0 4.5 4.0 3.5 3.0 2.5 2.0 1.5 1.0 0.5
f1 (ppm)

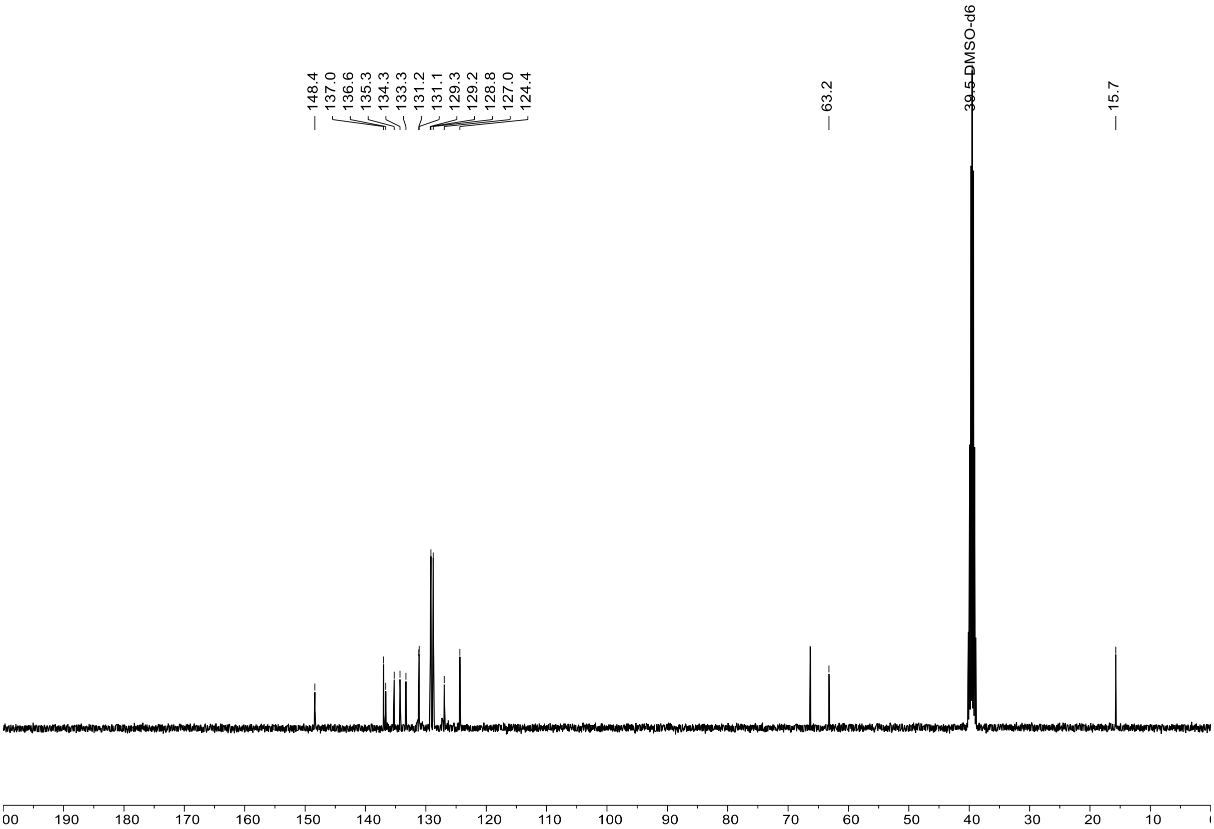
148.4
137.0
136.6
135.3
134.3
133.3
131.2
131.1
129.3
129.2
128.8
127.0
124.4
63.2
39.5 DMSO-d6
15.7
00 190 180 170 160 150 140 130 120 110 100 90 80 70 60 50 40 30 20 10

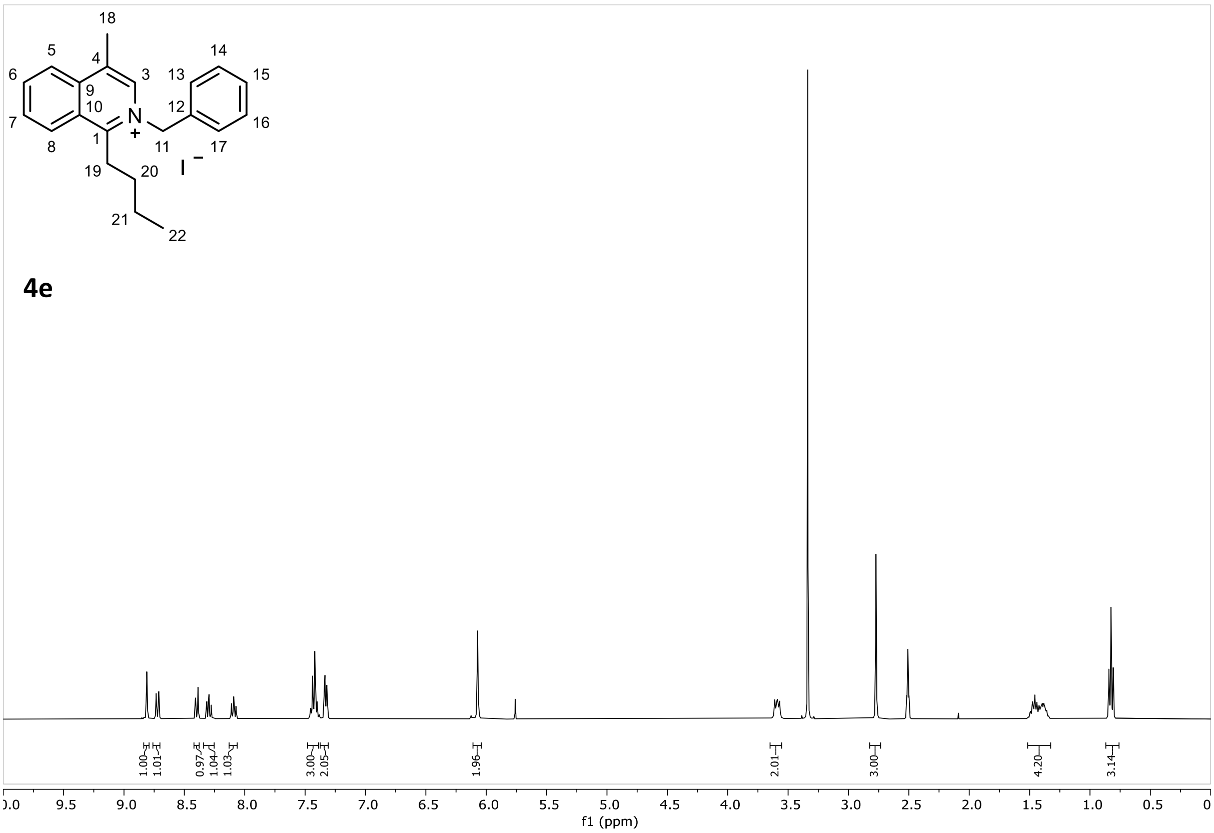

4e
I⁻
1.00
1.01
0.97
1.04
1.03
3.00
2.05
1.96
2.01
3.00
4.20
3.14
f1 (ppm)

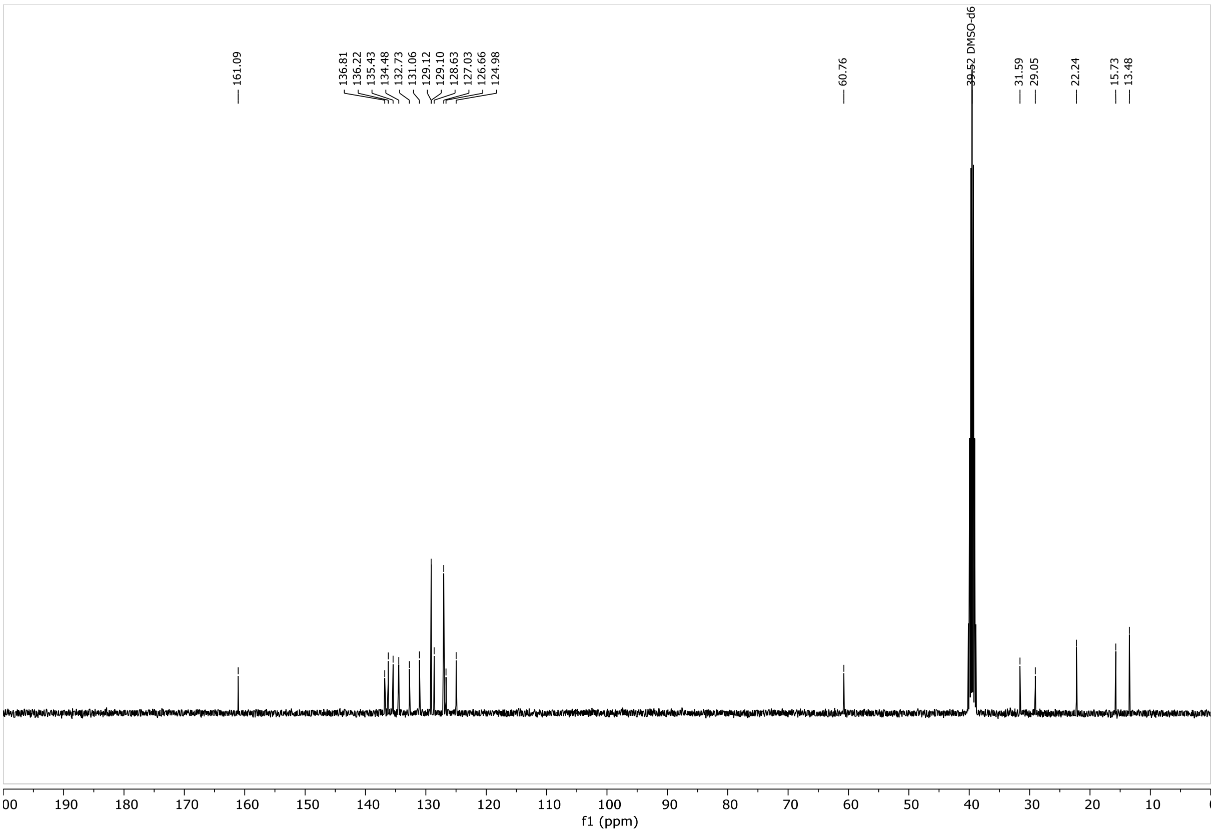

161.09
136.81
136.22
135.43
134.48
132.73
131.06
129.12
129.10
128.63
127.03
126.66
124.98
60.76
39.52 DMSO-d6
31.59
29.05
22.24
15.73
13.48
f1 (ppm)

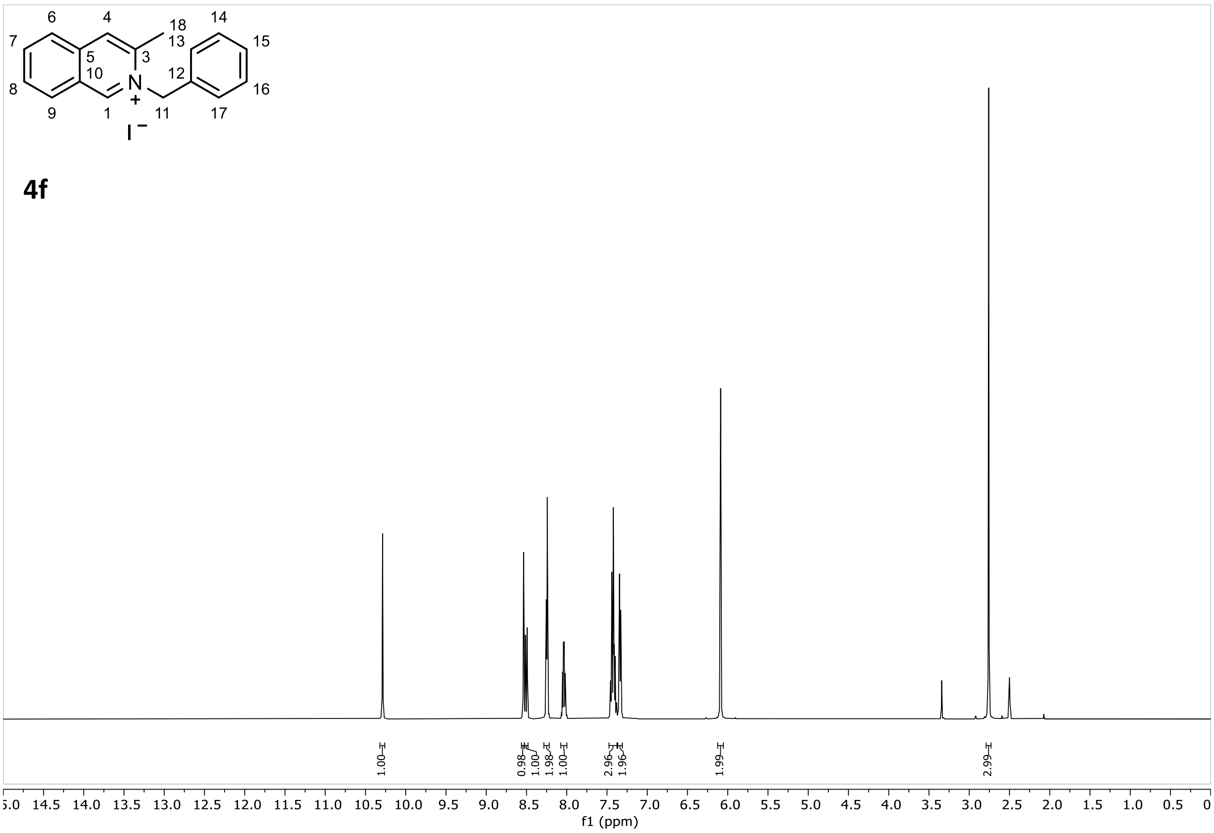
4f
1.00
0.98
1.00
1.98
1.00
2.96
1.96
1.99
2.99
f1 (ppm)

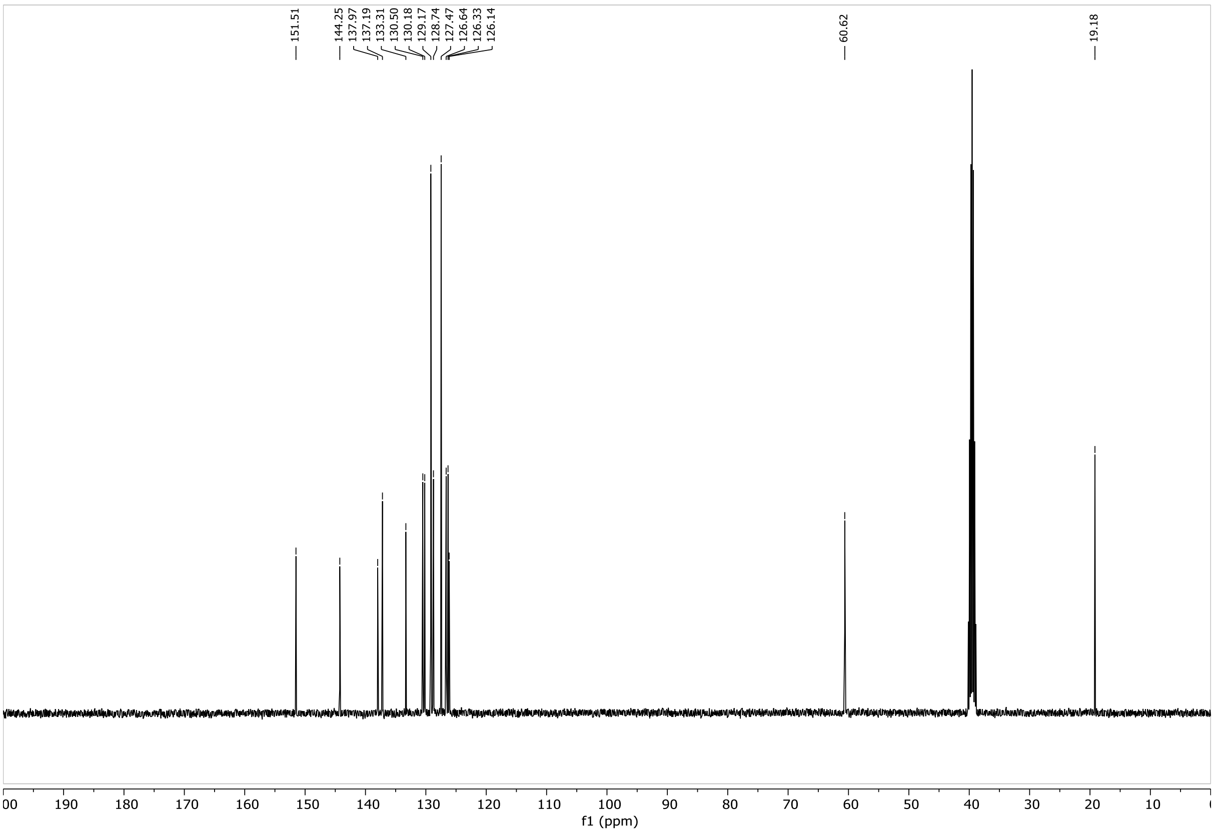
151.51
144.25
137.97
137.19
133.31
130.50
130.18
129.17
128.74
127.47
126.64
126.33
126.14
60.62
19.18
f1 (ppm)

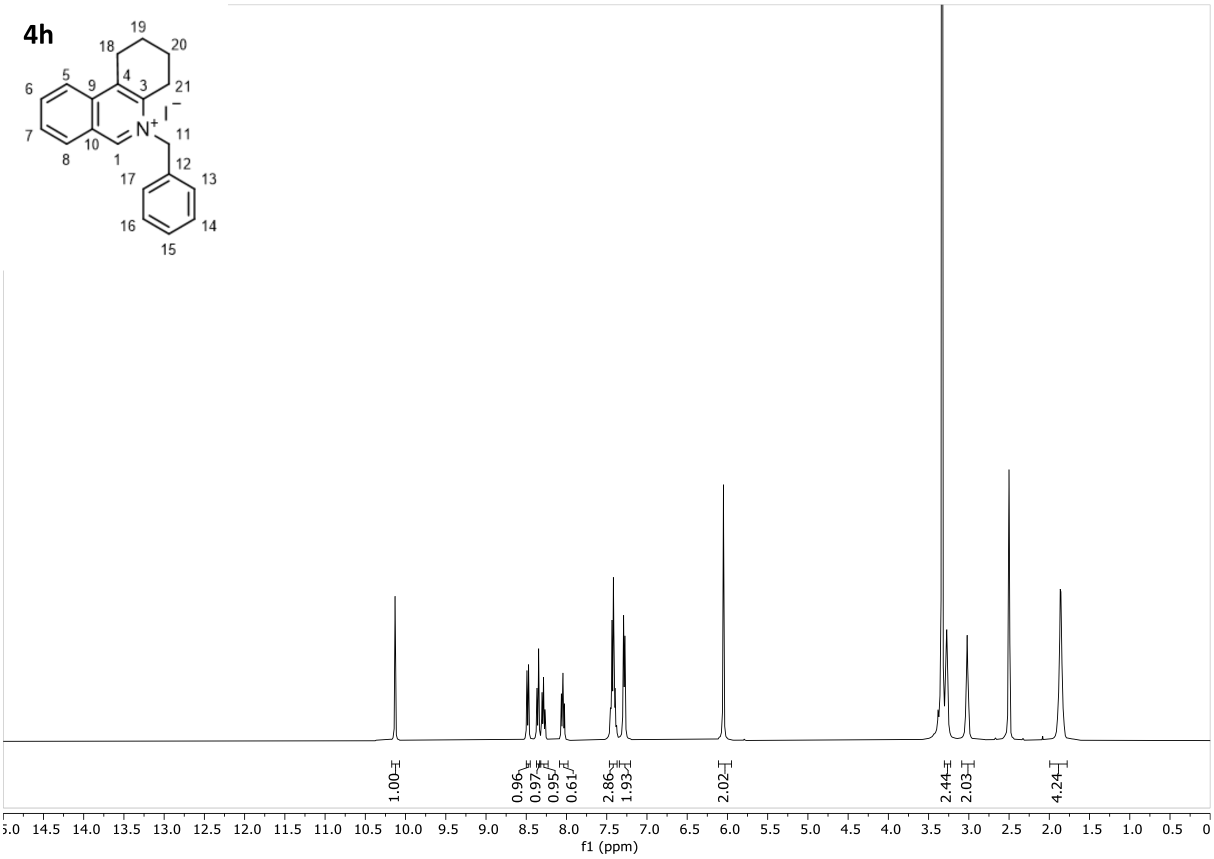
4h
1.00
0.96
0.97
0.95
0.61
2.86
1.93
2.02
2.44
2.03
4.24
f1 (ppm)

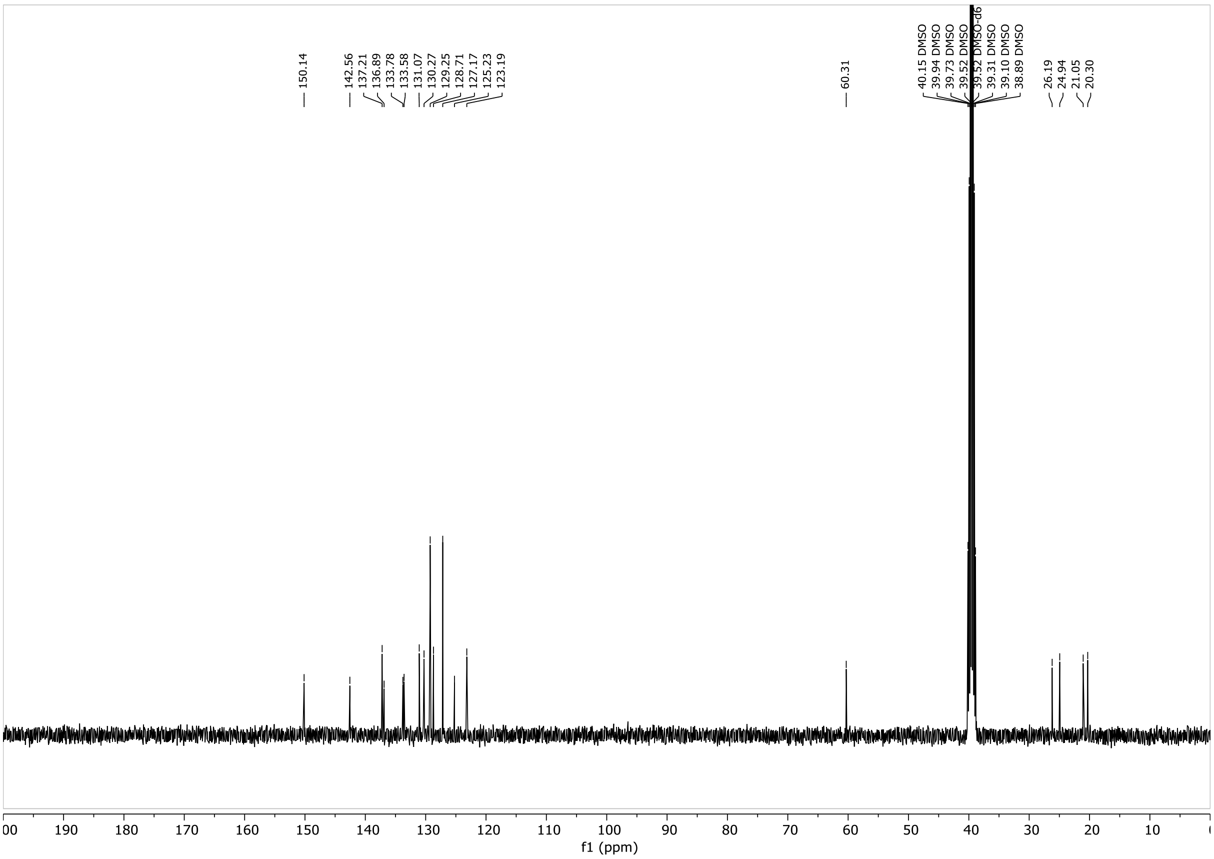
150.14
142.56
137.21
136.89
133.78
133.58
131.07
130.27
129.25
128.71
127.17
125.23
123.19
60.31
40.15 DMSO
39.94 DMSO
39.73 DMSO
39.52 DMSO
39.52 DMSO-d6
39.31 DMSO
39.10 DMSO
38.89 DMSO
26.19
24.94
21.05
20.30
f1 (ppm)

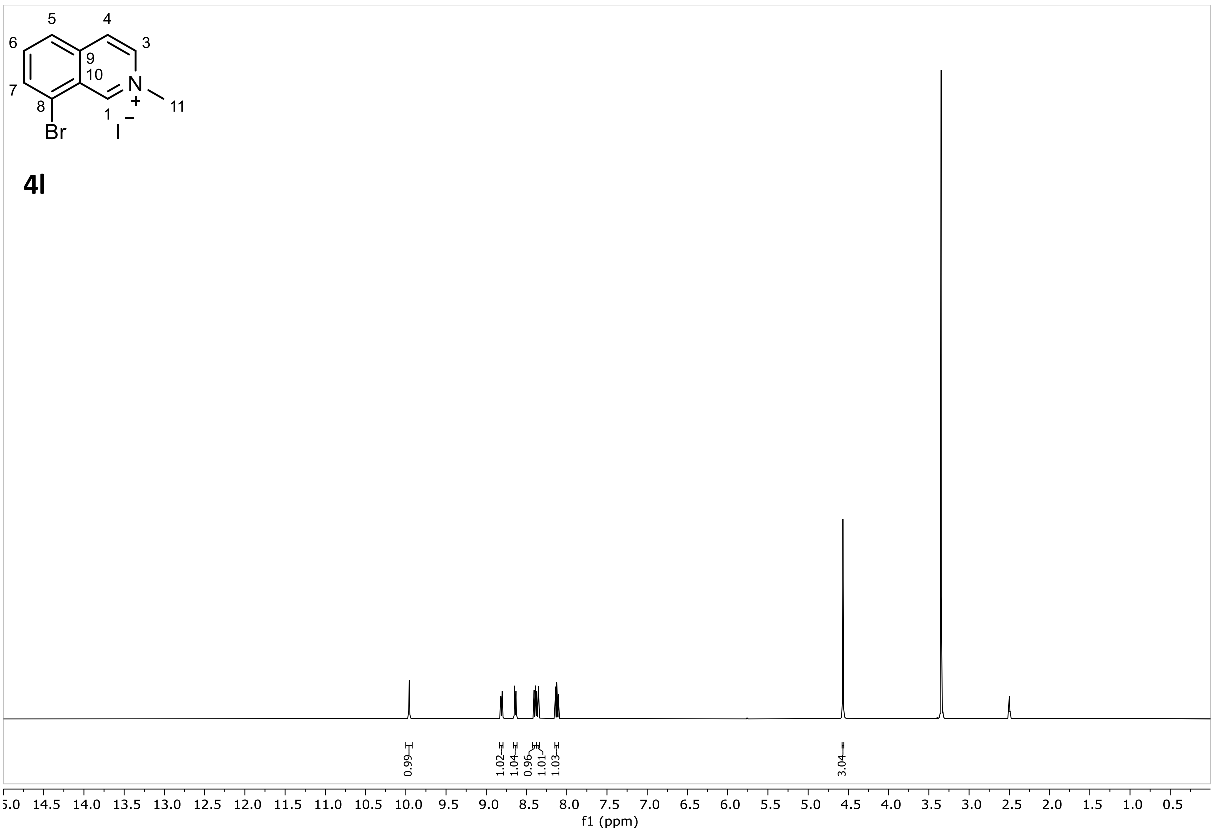
4l
0.99
1.02
1.04
0.96
1.01
1.03
3.04
f1 (ppm)

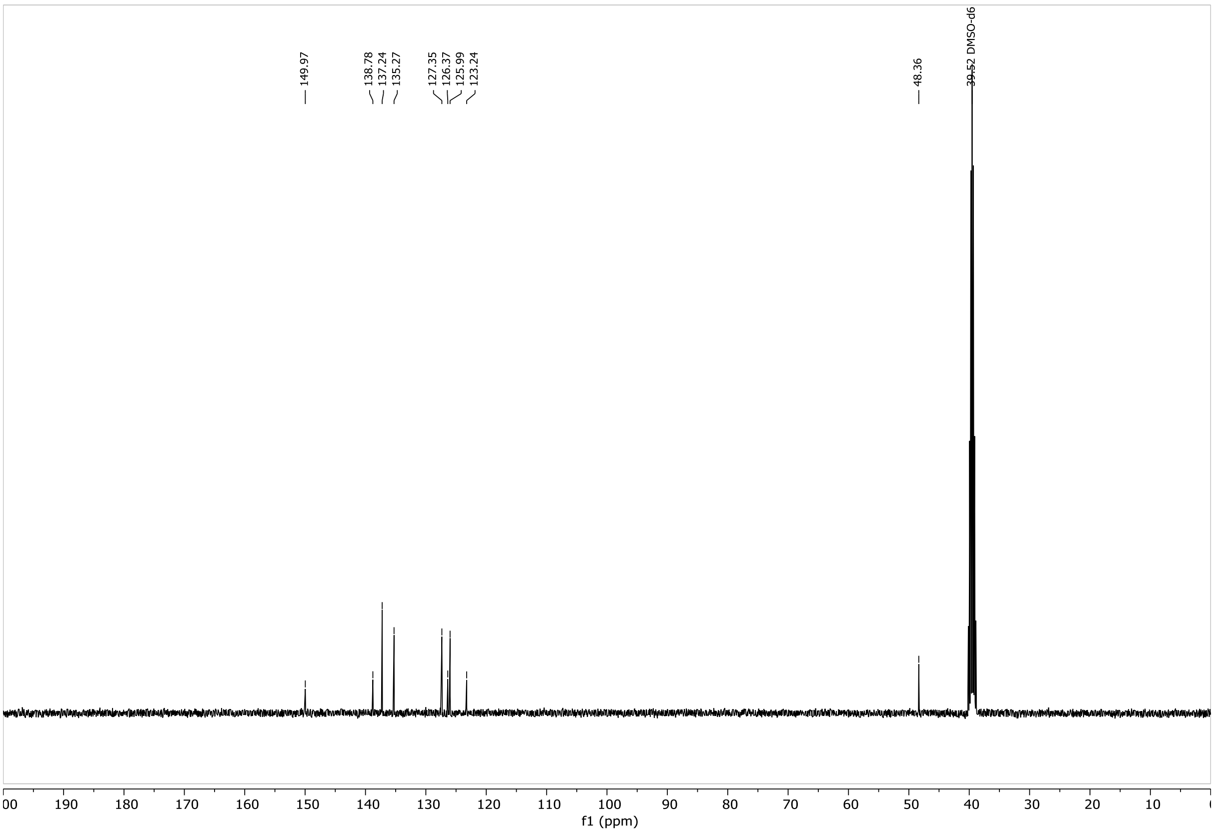
149.97
138.78
137.24
135.27
127.35
126.37
125.99
123.24
48.36
39.52 DMSO-d6
f1 (ppm)

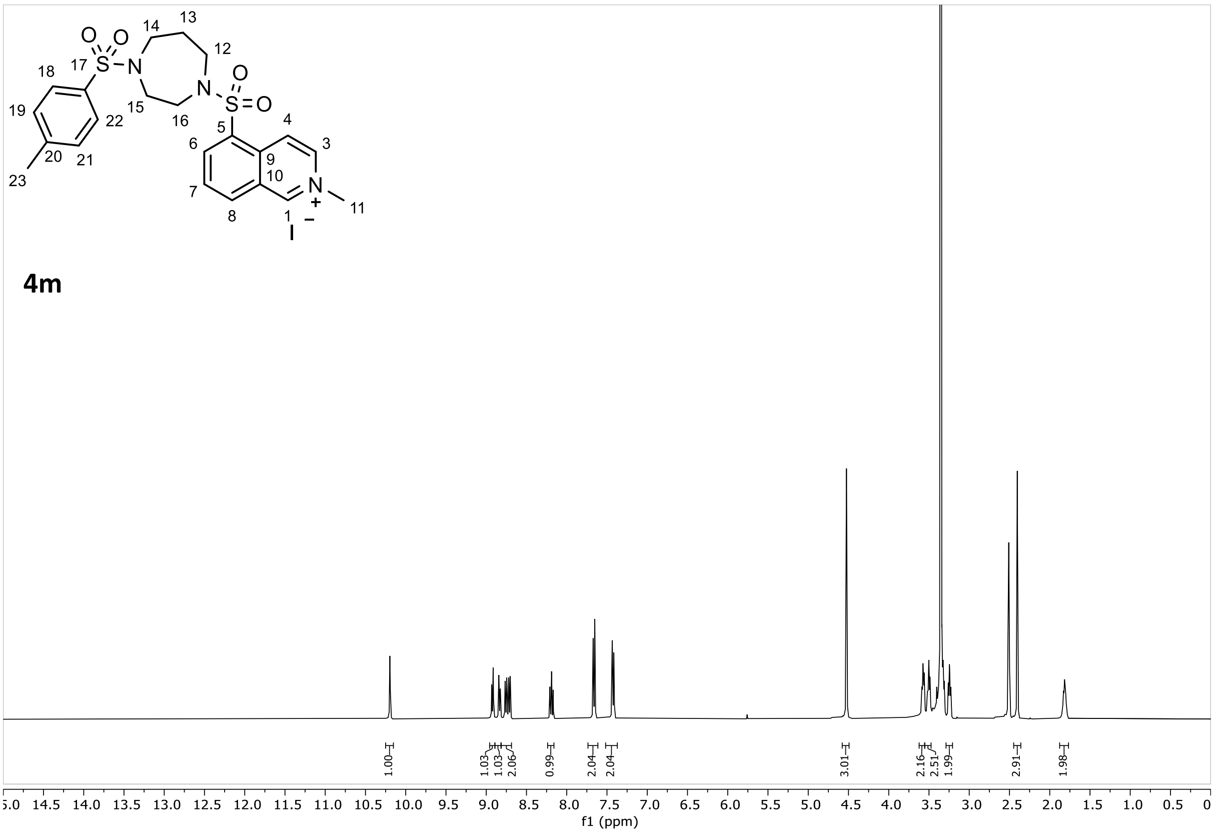
4m
f1 (ppm)

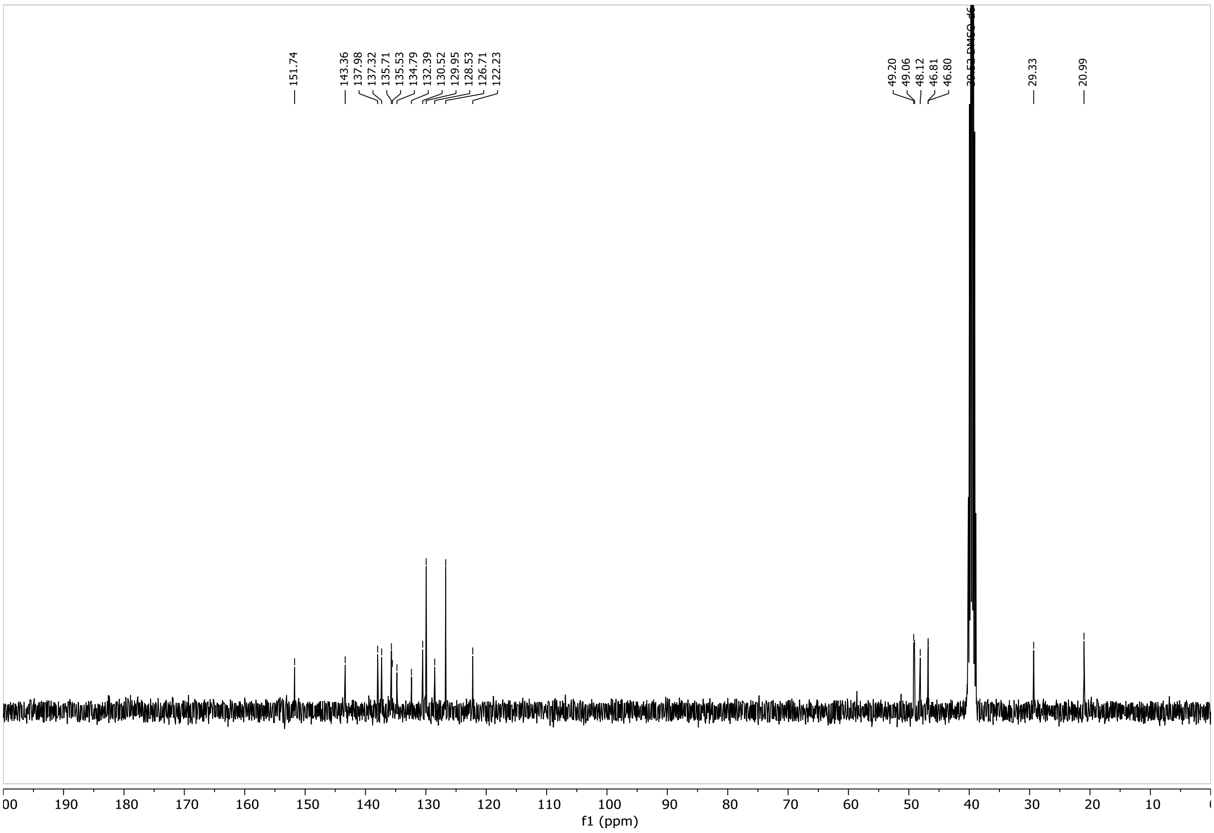
151.74
143.36
137.98
137.32
135.71
135.53
134.79
132.39
130.52
129.95
128.53
126.71
122.23
49.20
49.06
48.12
46.81
46.80
29.33
20.99
f1 (ppm)

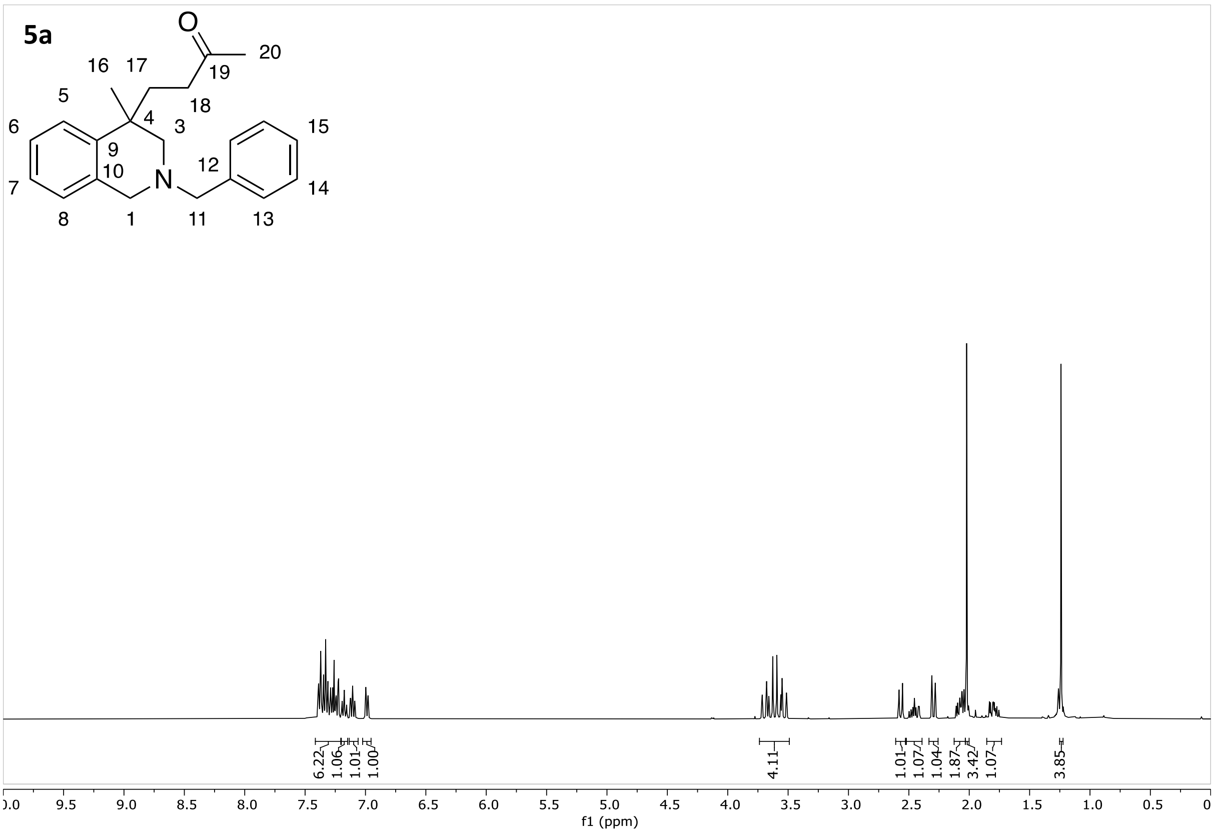
5a
O
20
19
16
17
18
5
4
3
6
9
10
12
15
N
7
14
8
1
11
13
6.22
1.06
1.01
1.00
4.11
1.01
1.07
1.04
1.87
3.42
1.07
3.85
f1 (ppm)

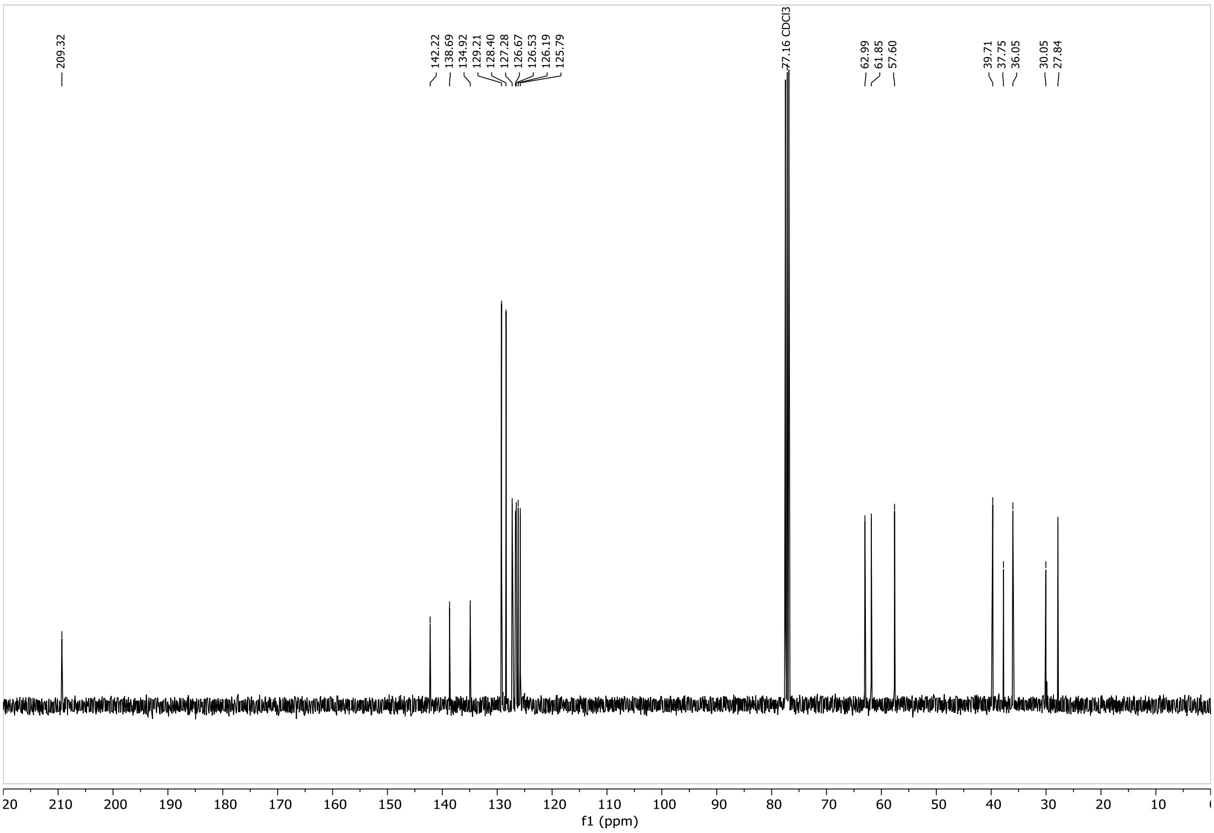
209.32
142.22
138.69
134.92
129.21
128.40
127.28
126.67
126.53
126.19
125.79
77.16 CDCl3
62.99
61.85
57.60
39.71
37.75
36.05
30.05
27.84
f1 (ppm)

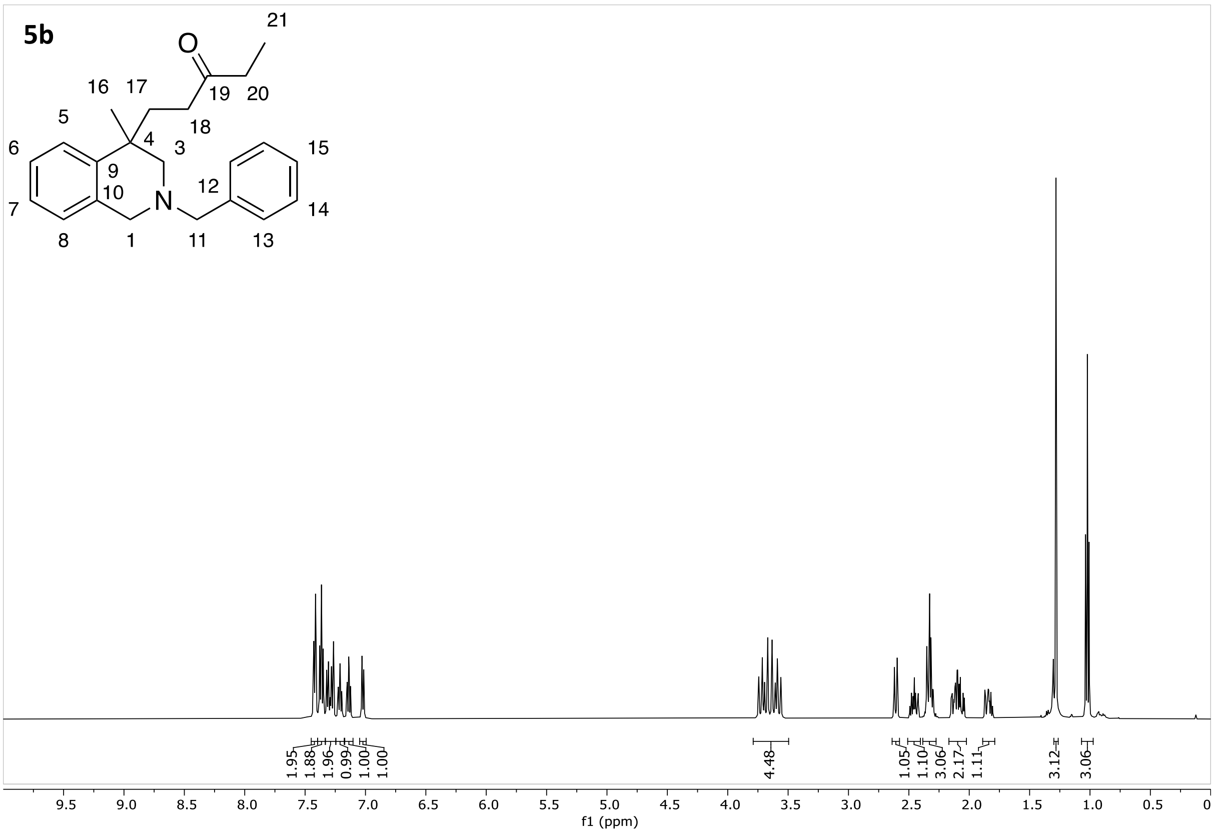
5b
1.95
1.88
1.96
0.99
1.00
1.00
4.48
1.05
1.10
3.06
2.17
1.11
3.12
3.06
f1 (ppm)

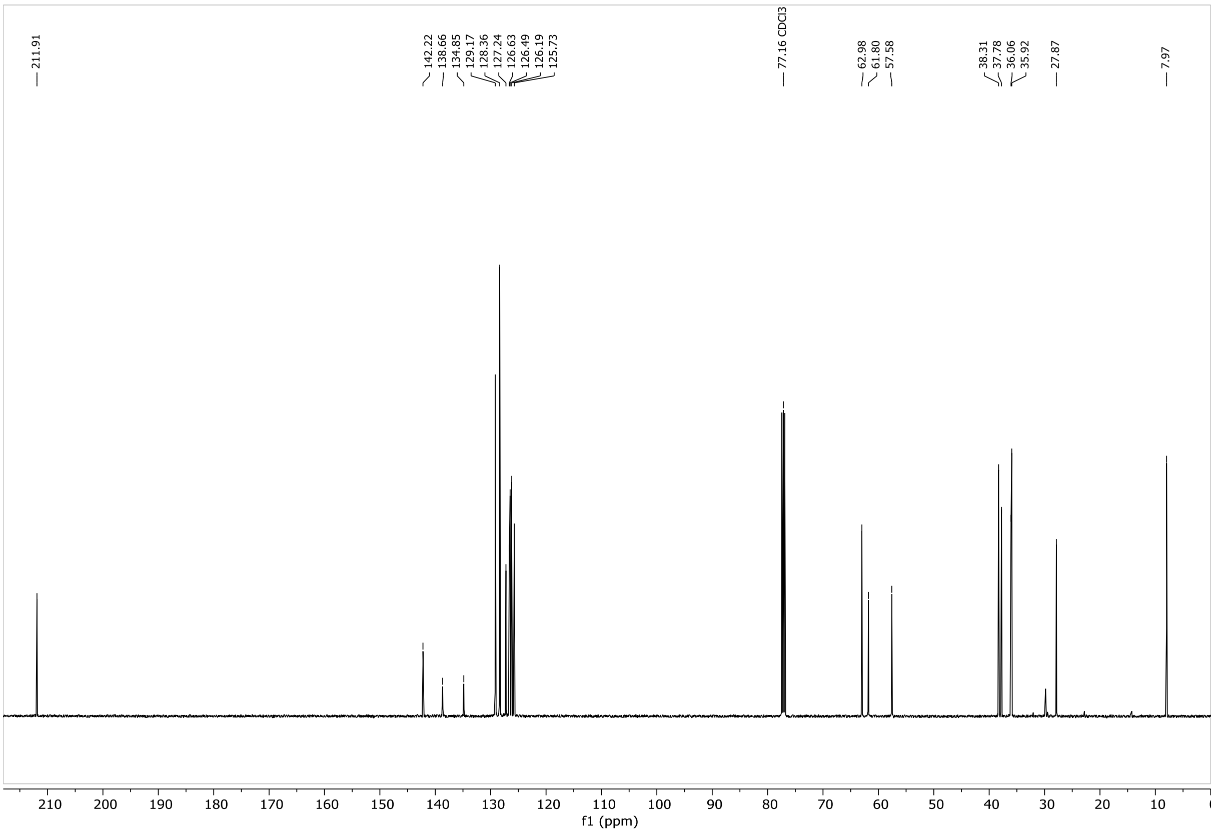
211.91
142.22
138.66
134.85
129.17
128.36
127.24
126.63
126.49
126.19
125.73
77.16 CDCl3
62.98
61.80
57.58
38.31
37.78
36.06
35.92
27.87
7.97
f1 (ppm)

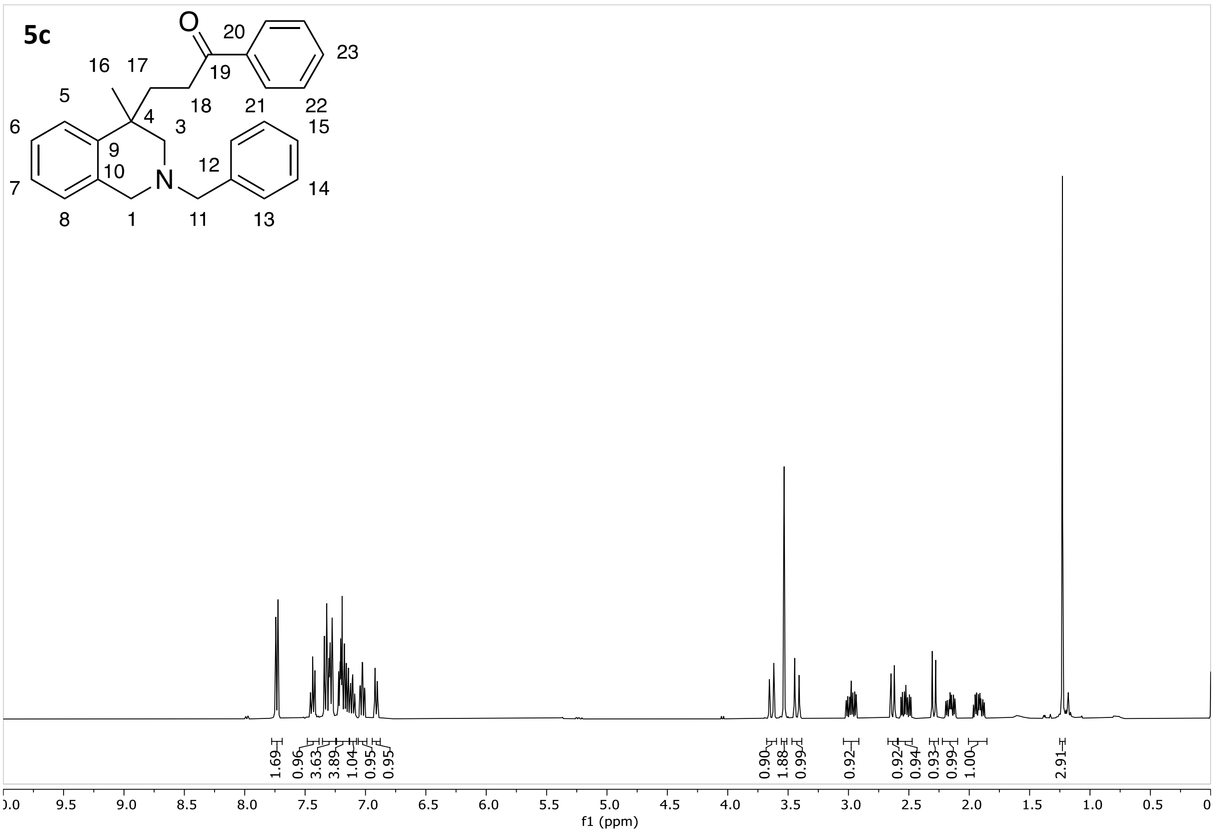
5c
1.69
0.96
3.63
3.89
1.04
0.95
0.95
0.90
1.88
0.99
0.92
0.92
0.94
0.93
0.99
1.00
2.91
f1 (ppm)

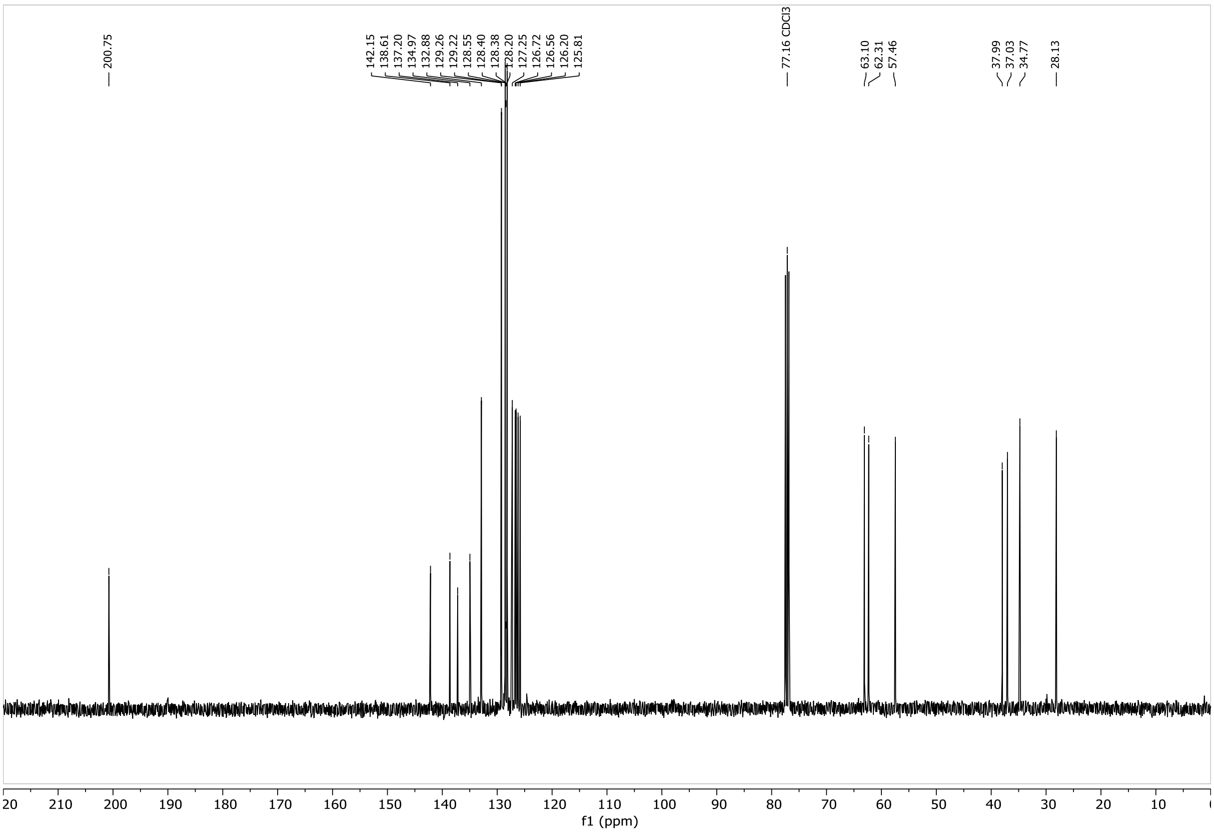
200.75
142.15
138.61
137.20
134.97
132.88
129.26
129.22
128.55
128.40
128.38
128.20
127.25
126.72
126.56
126.20
125.81
77.16 CDCl3
63.10
62.31
57.46
37.99
37.03
34.77
28.13
f1 (ppm)

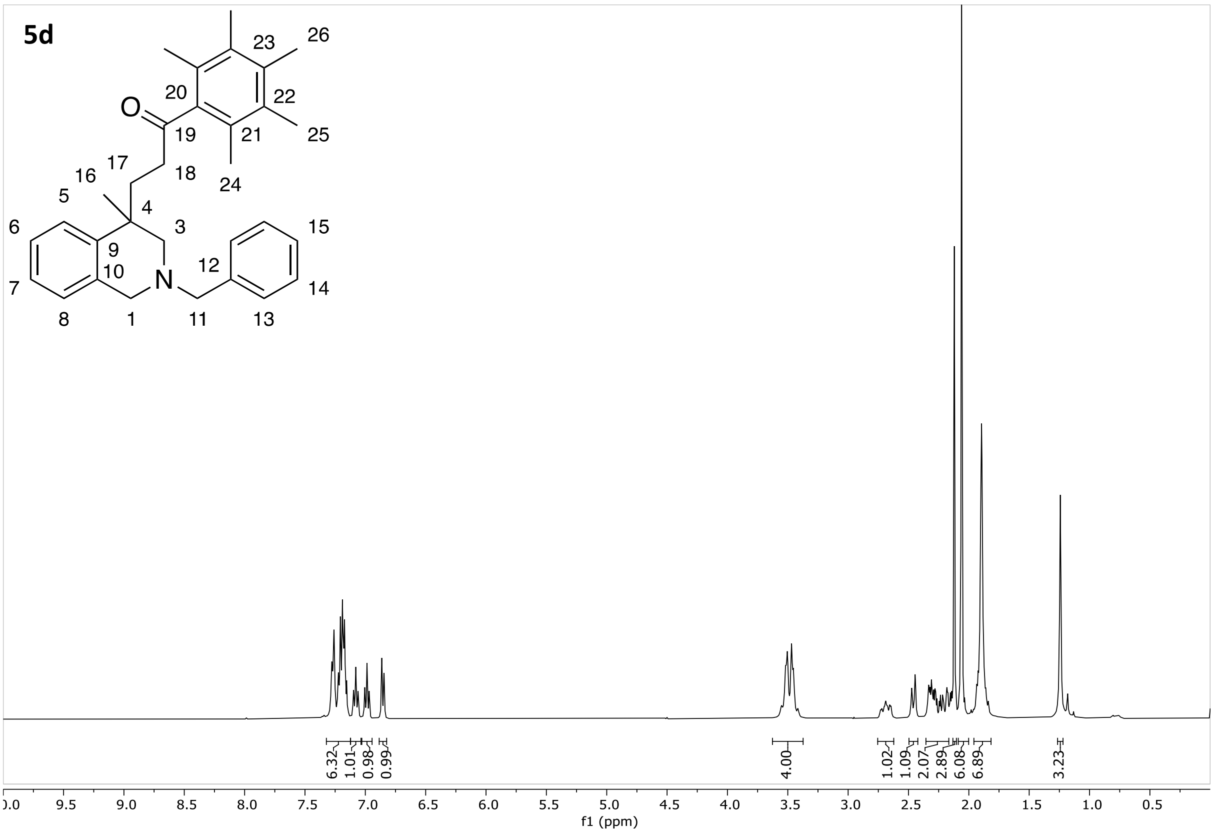
5d
O
N
1
3
4
5
6
7
8
9
10
11
12
13
14
15
16
17
18
19
20
21
22
23
24
25
26
6.32
1.01
0.98
0.99
4.00
1.02
1.09
2.07
2.89
6.08
6.89
3.23
f1 (ppm)

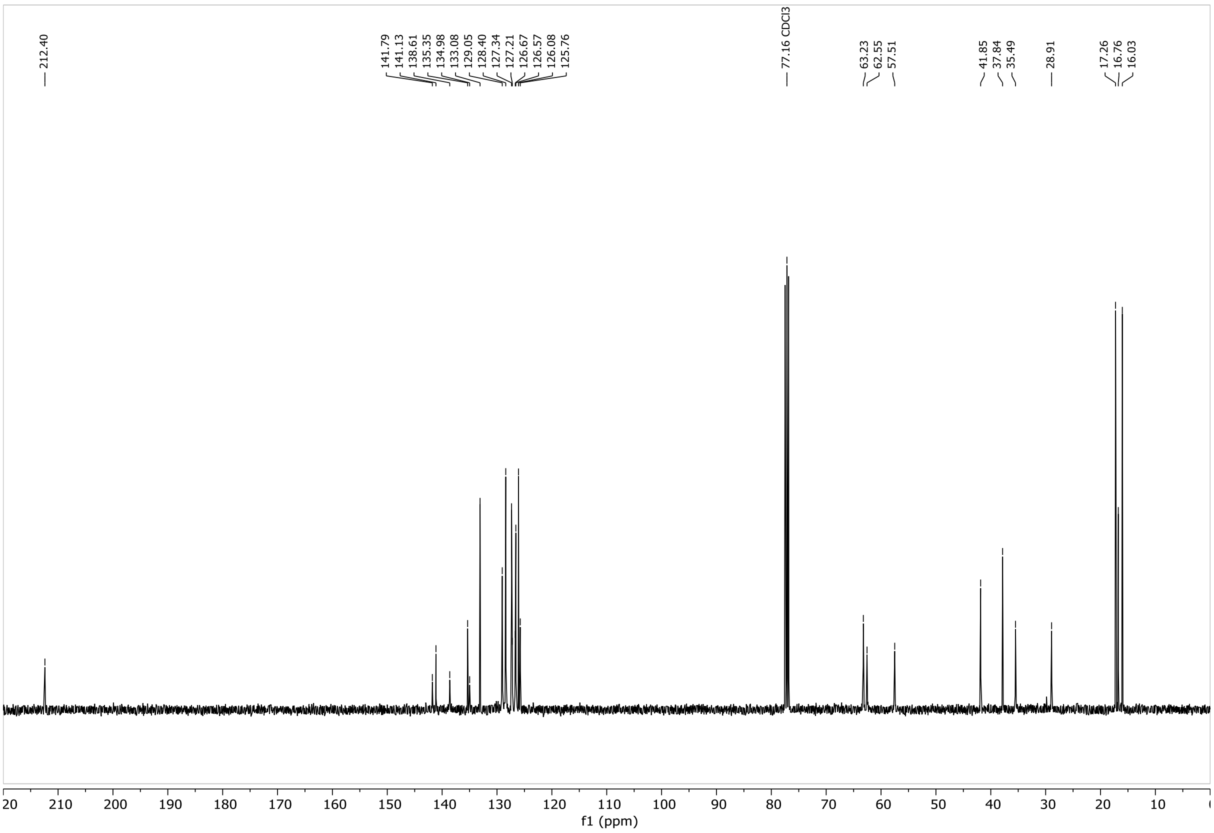
212.40
141.79
141.13
138.61
135.35
134.98
133.08
129.05
128.40
127.34
127.21
126.67
126.57
126.08
125.76
77.16 CDCl3
63.23
62.55
57.51
41.85
37.84
35.49
28.91
17.26
16.76
16.03
f1 (ppm)

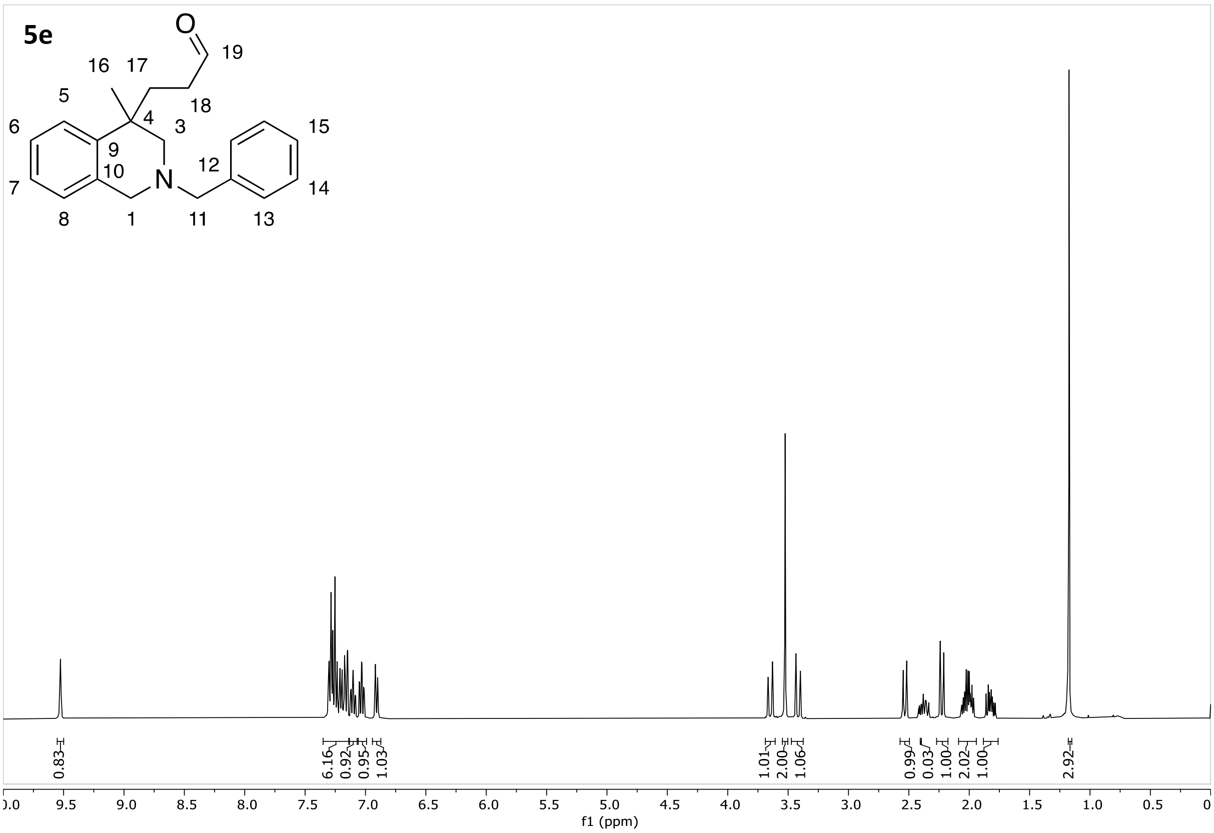
5e
O
19
16
17
18
5
4
3
6
9
10
12
15
7
N
14
8
1
11
13
0.83
6.16
0.92
0.95
1.03
1.01
2.00
1.06
0.99
0.03
1.00
2.02
1.00
2.92
f1 (ppm)

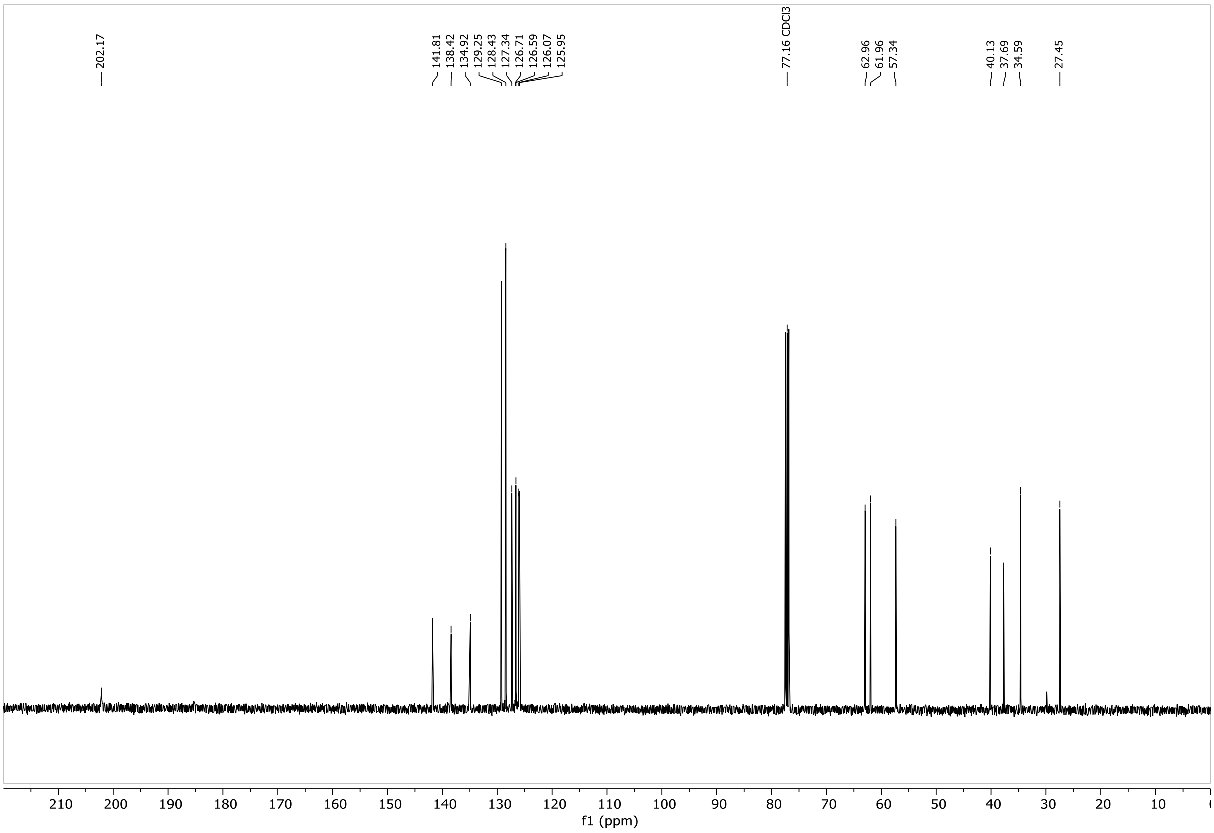
202.17
141.81
138.42
134.92
129.25
128.43
127.34
126.71
126.59
126.07
125.95
77.16 CDCl3
62.96
61.96
57.34
40.13
37.69
34.59
27.45
f1 (ppm)

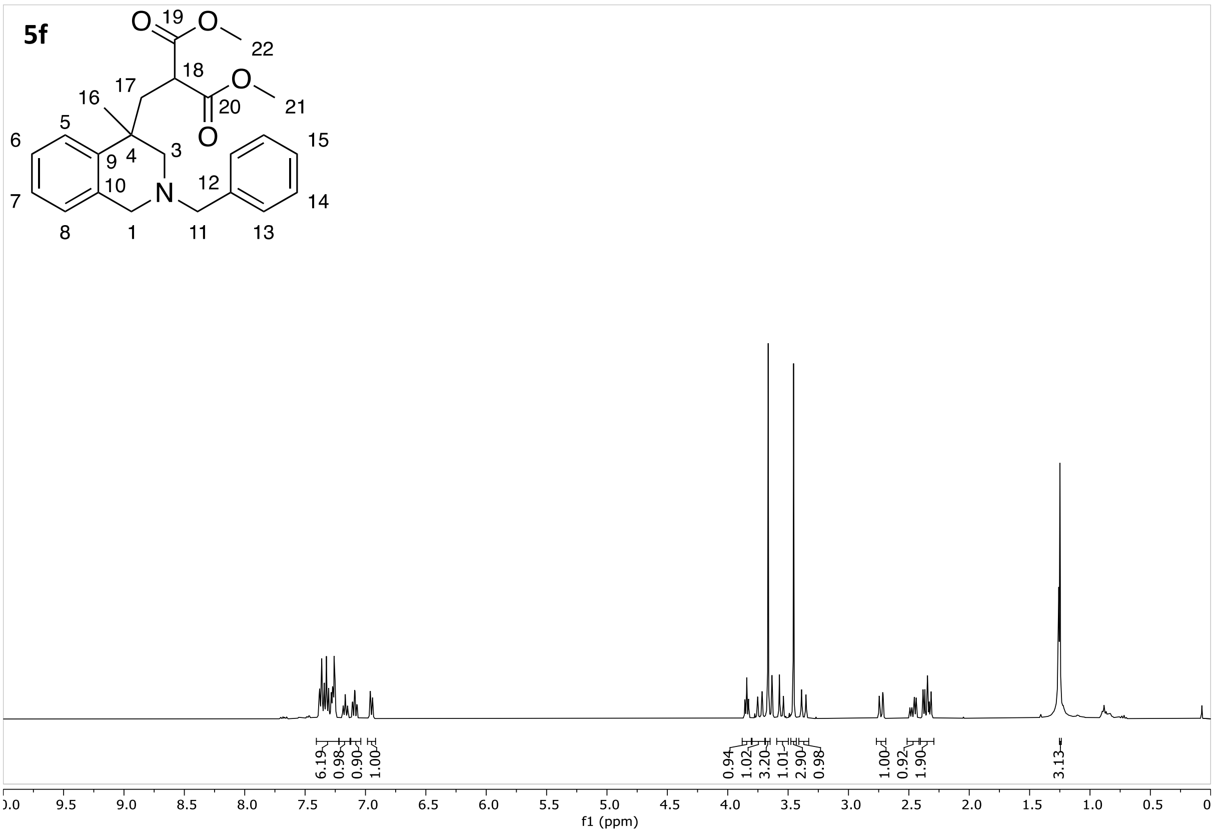
5f
6.19
0.98
0.90
1.00
0.94
1.02
3.20
1.01
2.90
0.98
1.00
0.92
1.90
3.13
f1 (ppm)

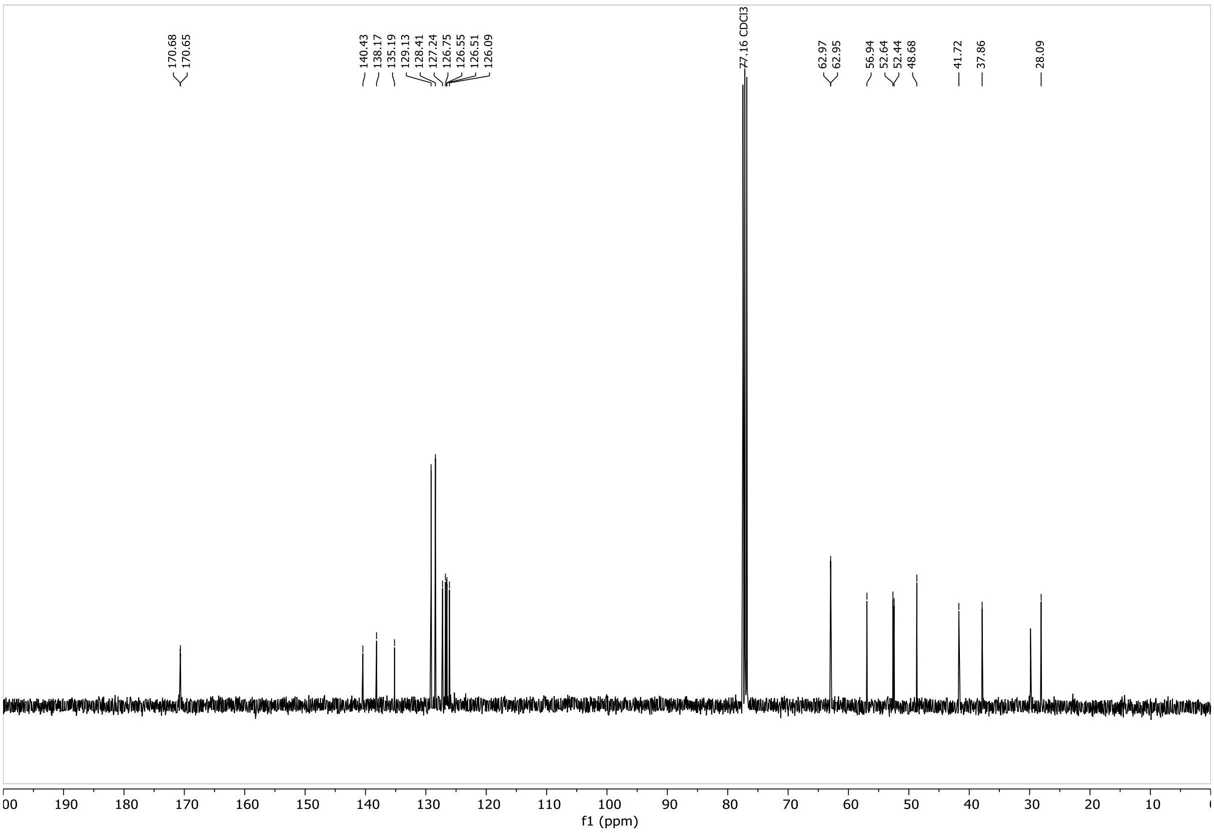
170.68
170.65
140.43
138.17
135.19
129.13
128.41
127.24
126.75
126.55
126.51
126.09
77.16 CDCl3
62.97
62.95
56.94
52.64
52.44
48.68
41.72
37.86
28.09
f1 (ppm)

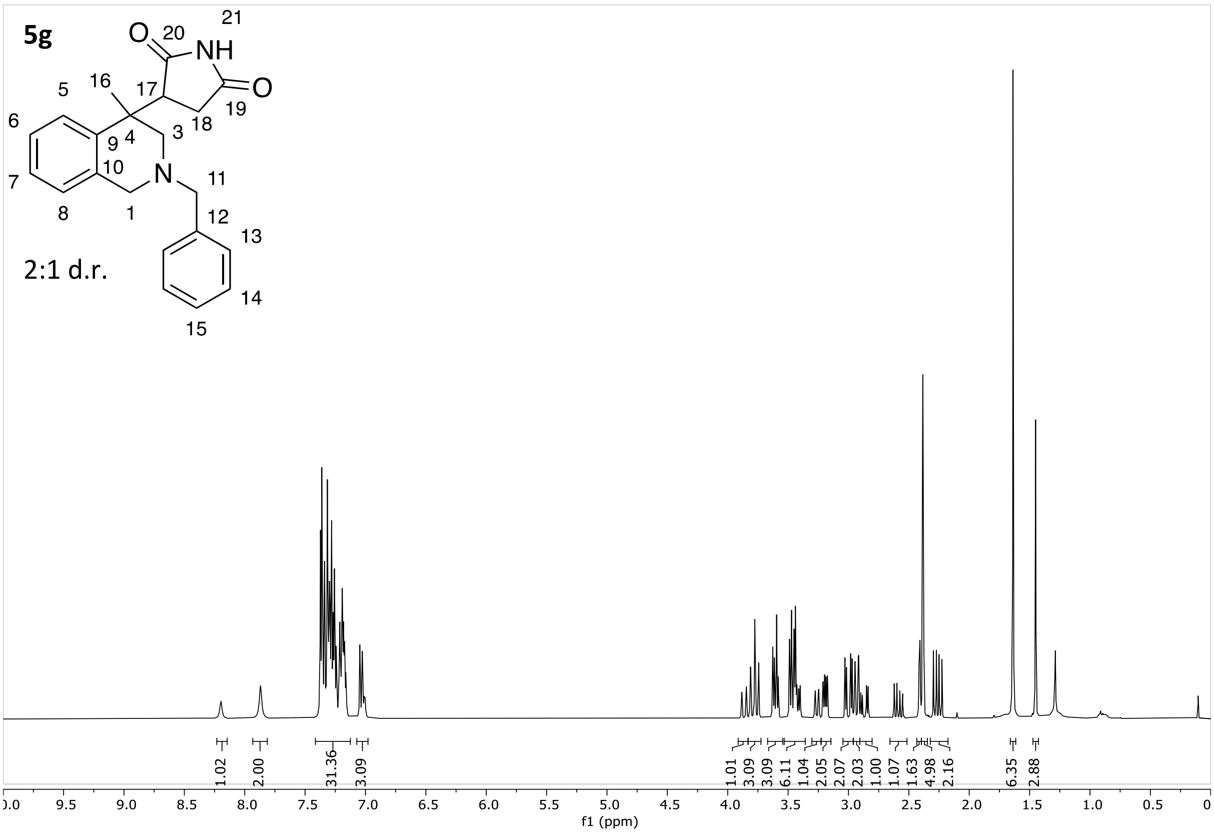
5g
2:1 d.r.
1.02
2.00
31.36
3.09
1.01
3.09
3.09
6.11
1.04
2.05
2.07
2.03
1.00
1.07
1.63
4.98
2.16
6.35
2.88
f1 (ppm)

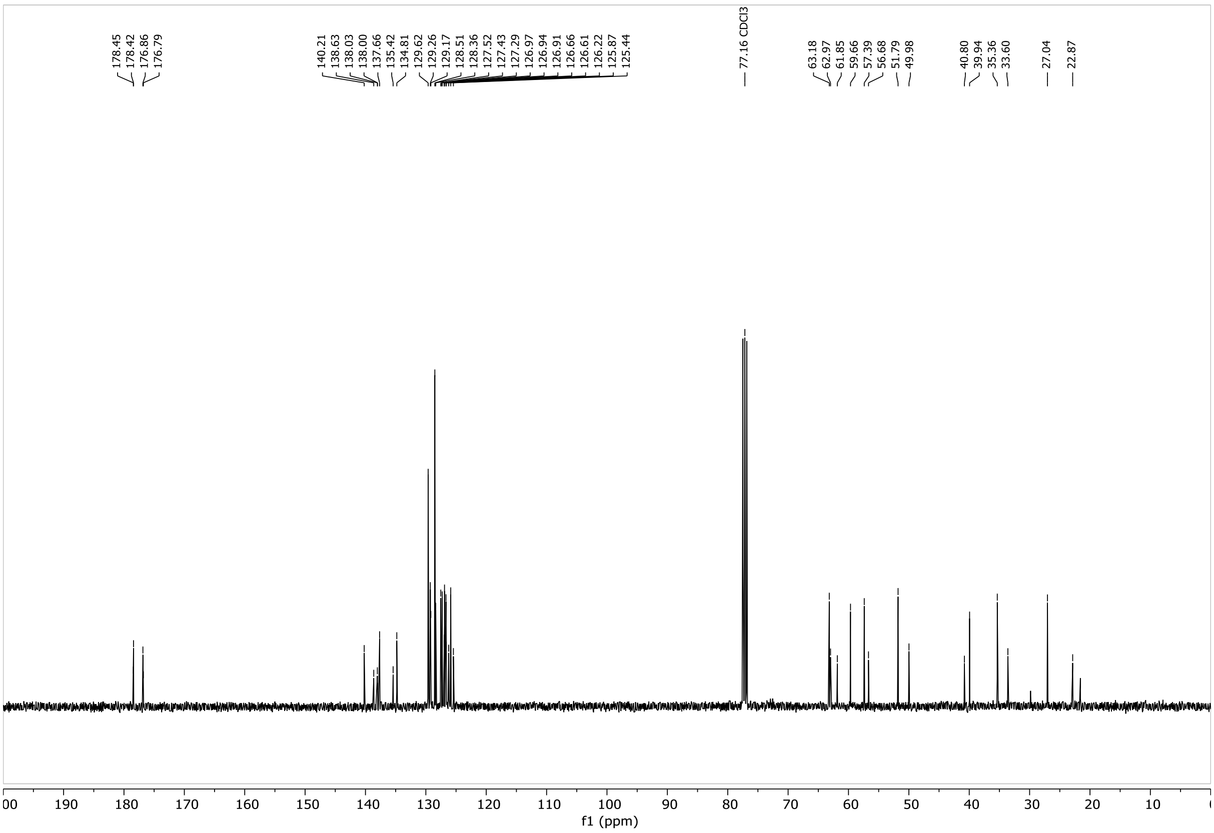
178.45
178.42
176.86
176.79
140.21
138.63
138.03
138.00
137.66
135.42
134.81
129.62
129.26
129.17
128.51
128.36
127.52
127.43
127.29
126.97
126.94
126.91
126.66
126.61
126.22
125.87
125.44
77.16 CDCl3
63.18
62.97
61.85
59.66
57.39
56.68
51.79
49.98
40.80
39.94
35.36
33.60
27.04
22.87
f1 (ppm)

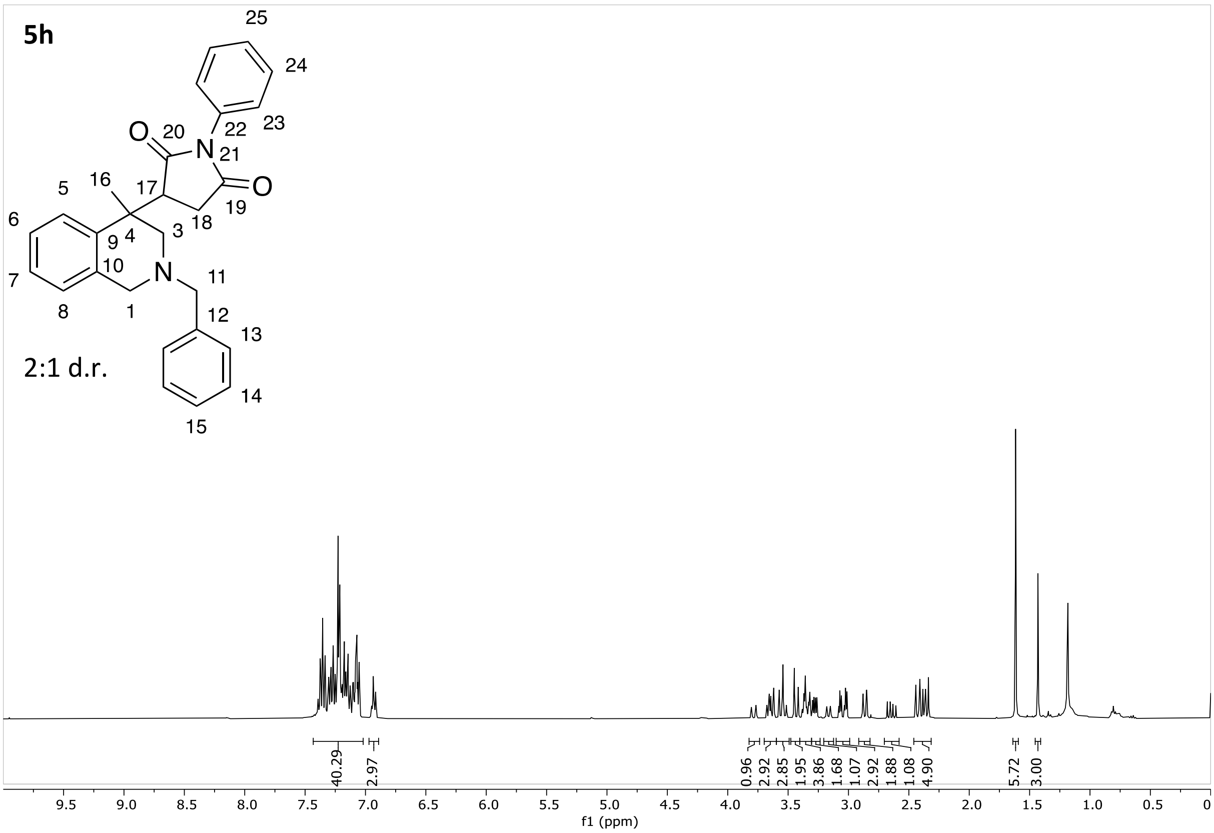
5h
2:1 d.r.
0.96
2.92
2.85
1.95
3.86
1.68
1.07
2.92
1.88
1.08
4.90
40.29
2.97
5.72
3.00
f1 (ppm)

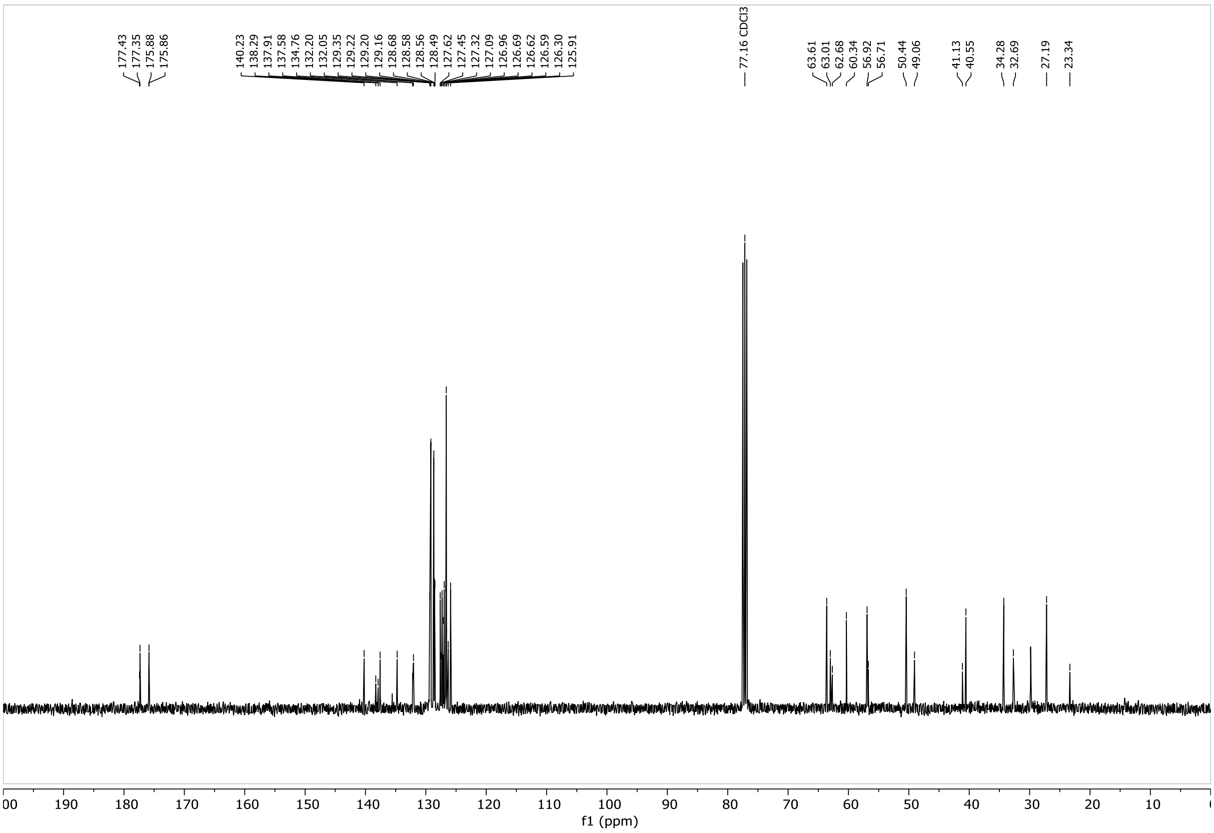
177.43
177.35
175.88
175.86
140.23
138.29
137.91
137.58
134.76
132.20
132.05
129.35
129.22
129.20
129.16
128.68
128.58
128.56
128.49
127.62
127.45
127.32
127.09
126.96
126.69
126.62
126.59
126.30
125.91
77.16 CDCl3
63.61
63.01
62.68
60.34
56.92
56.71
50.44
49.06
41.13
40.55
34.28
32.69
27.19
23.34
f1 (ppm)

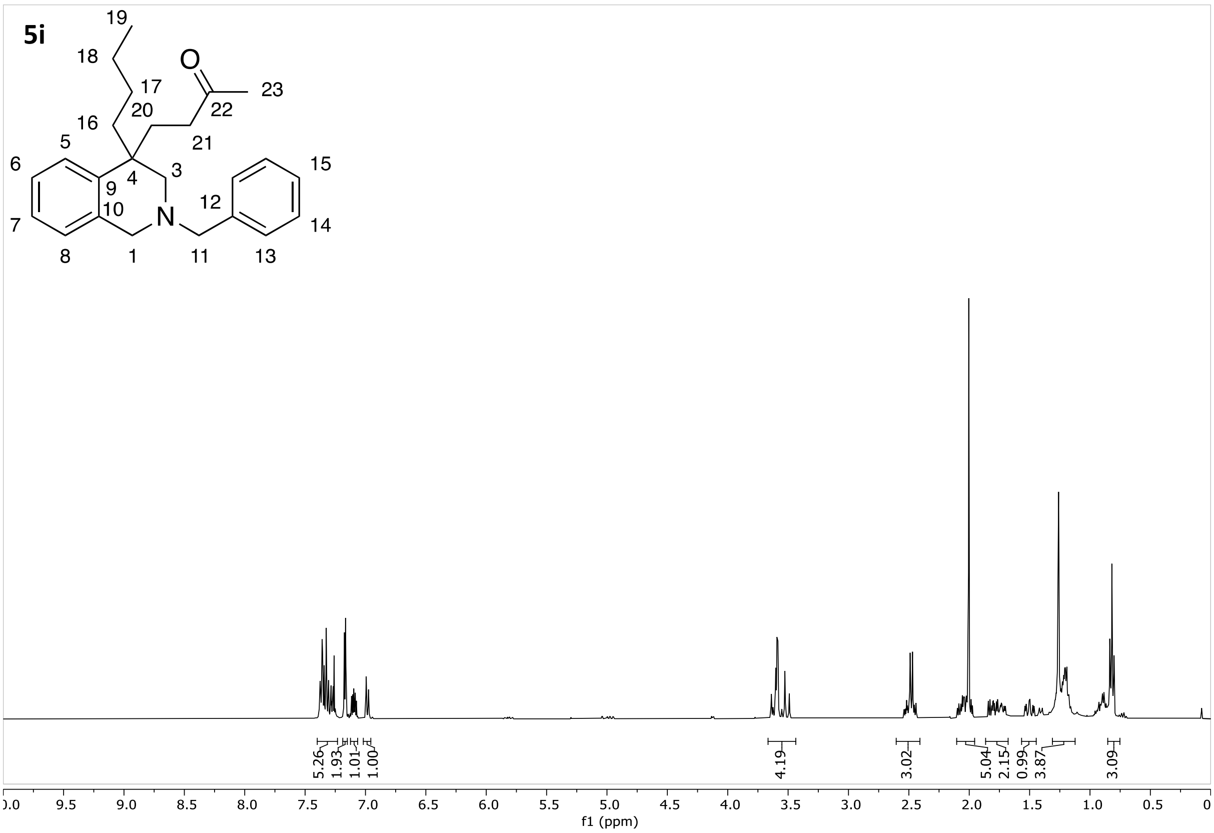
5i
5.26
1.93
1.01
1.00
4.19
3.02
5.04
2.15
0.99
3.87
3.09
f1 (ppm)

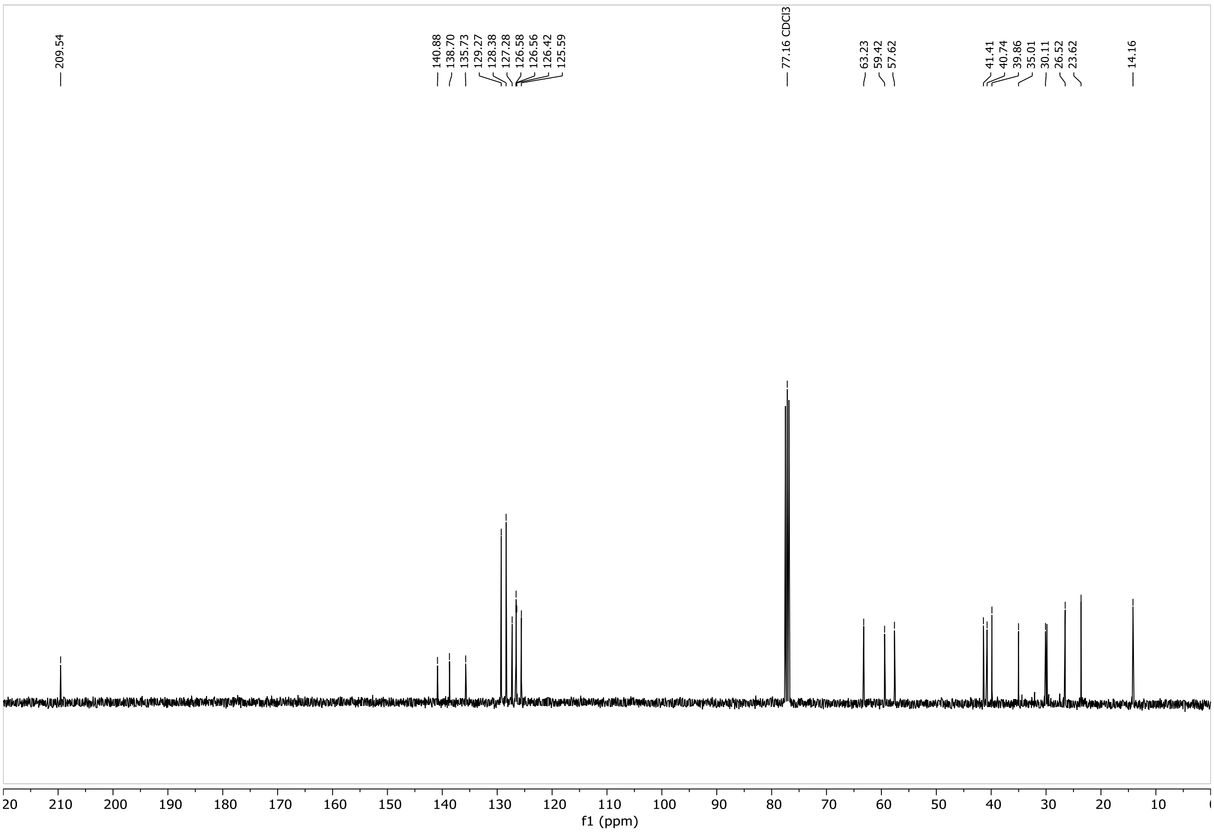
209.54
140.88
138.70
135.73
129.27
128.38
127.28
126.58
126.56
126.42
125.59
77.16 CDCl3
63.23
59.42
57.62
41.41
40.74
39.86
35.01
30.11
26.52
23.62
14.16
f1 (ppm)

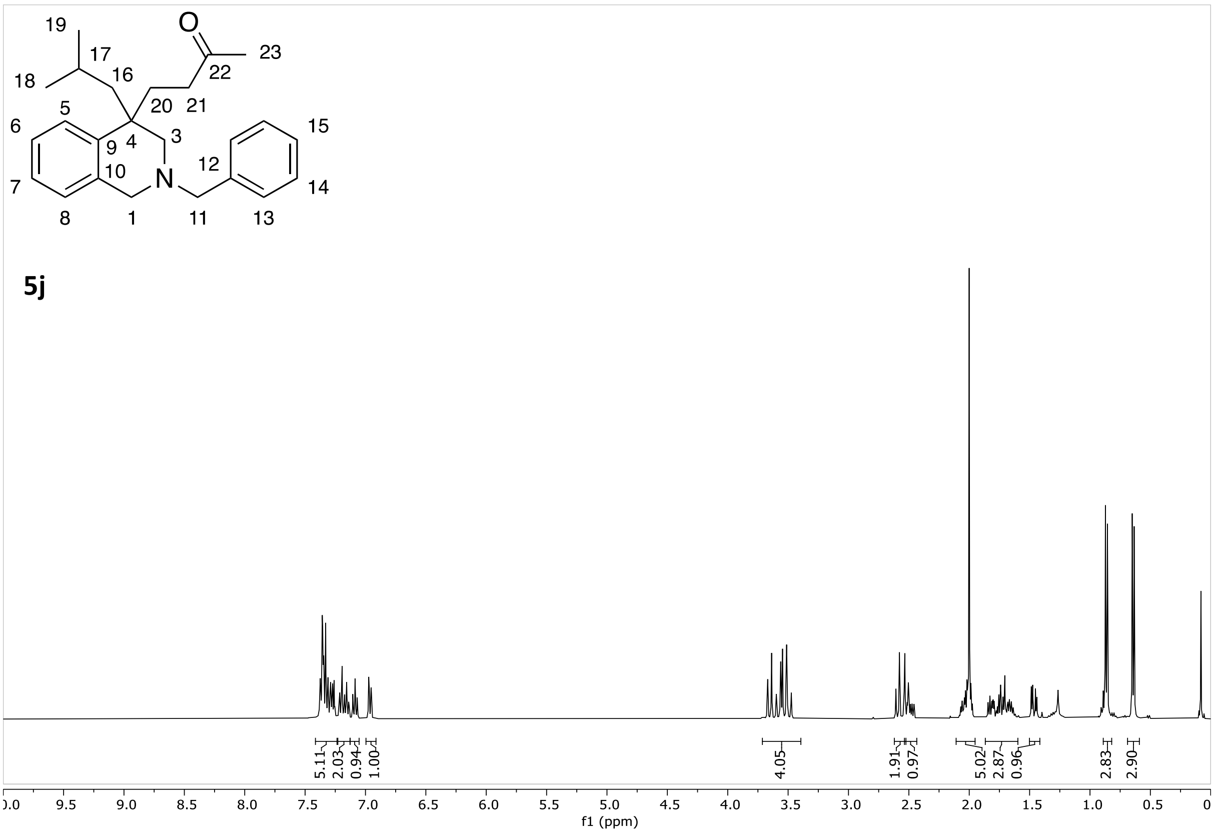
5j
5.11
2.03
0.94
1.00
4.05
1.91
0.97
5.02
2.87
0.96
2.83
2.90
f1 (ppm)

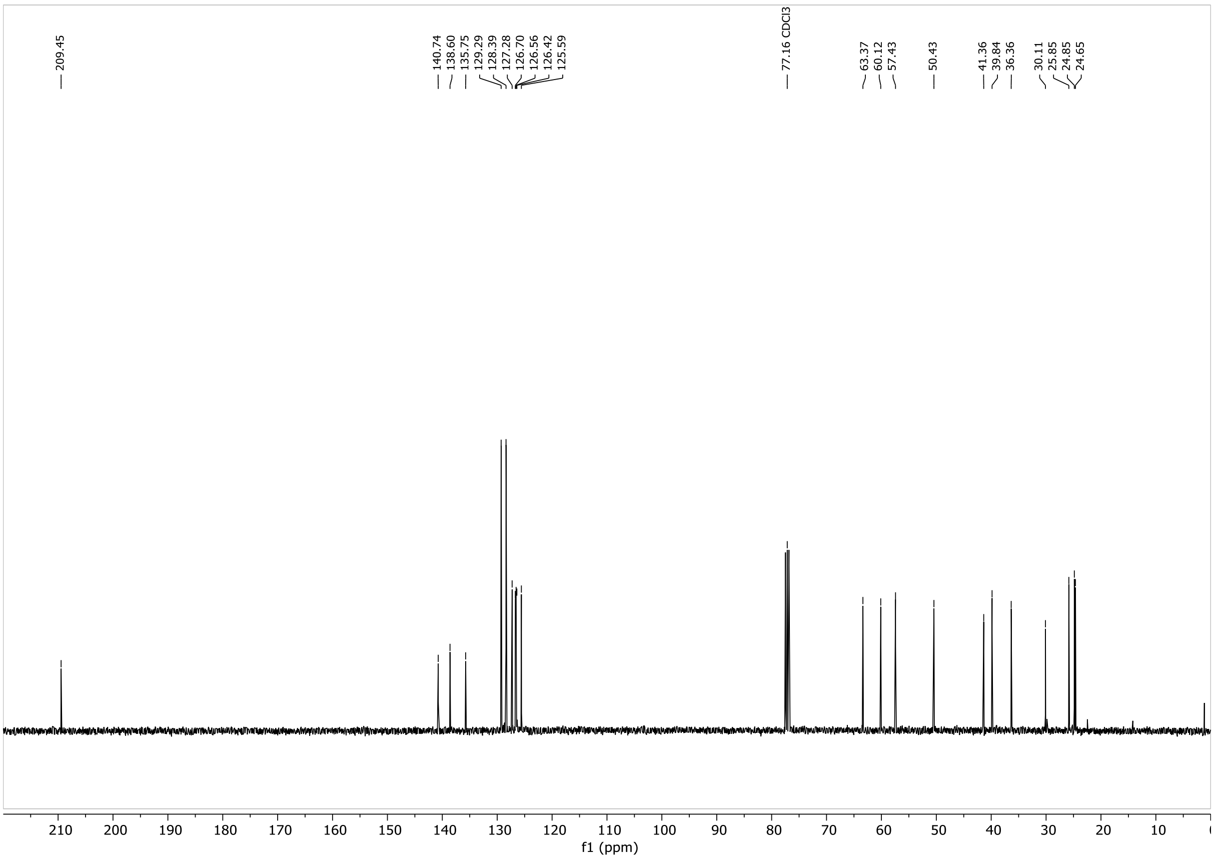
209.45
140.74
138.60
135.75
129.29
128.39
127.28
126.70
126.56
126.42
125.59
77.16 CDCl3
63.37
60.12
57.43
50.43
41.36
39.84
36.36
30.11
25.85
24.85
24.65
f1 (ppm)

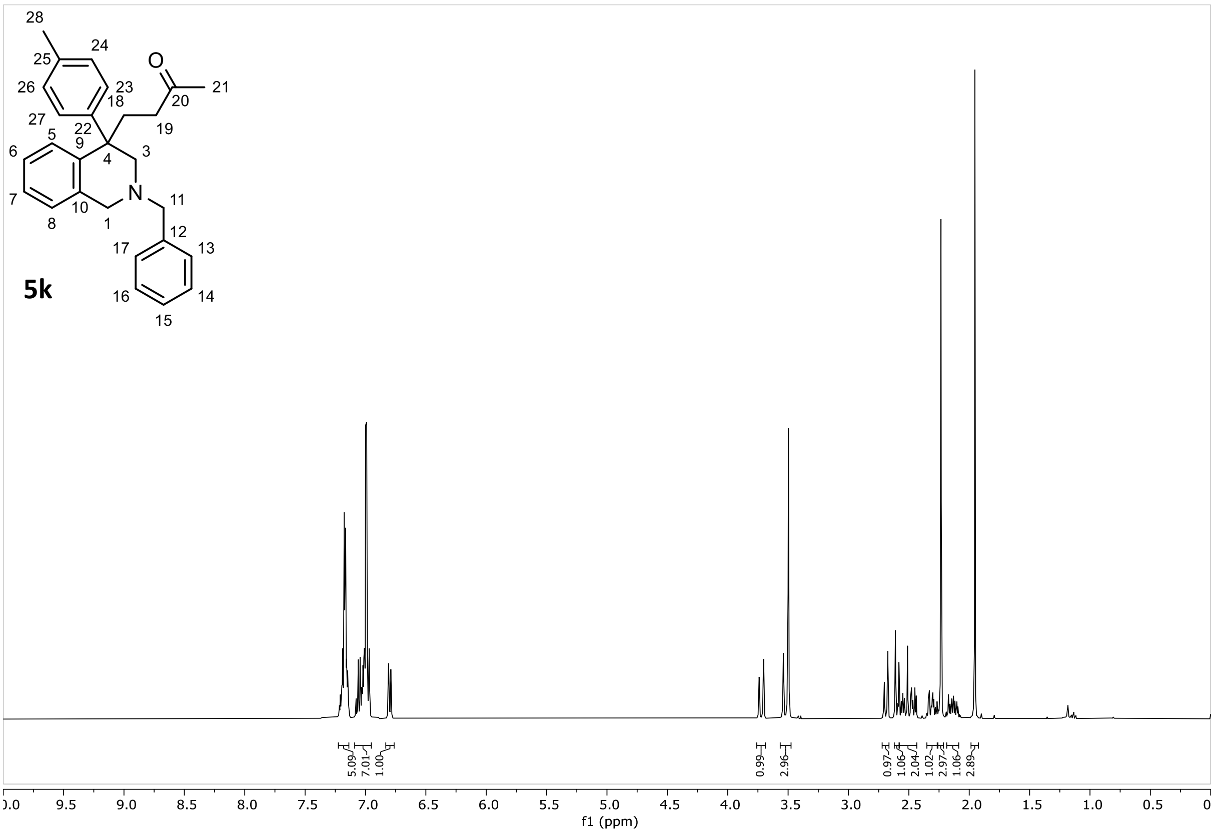

5k
5.09
7.01
1.00
0.99
2.96
0.97
1.06
2.04
1.02
2.97
1.06
2.89
f1 (ppm)

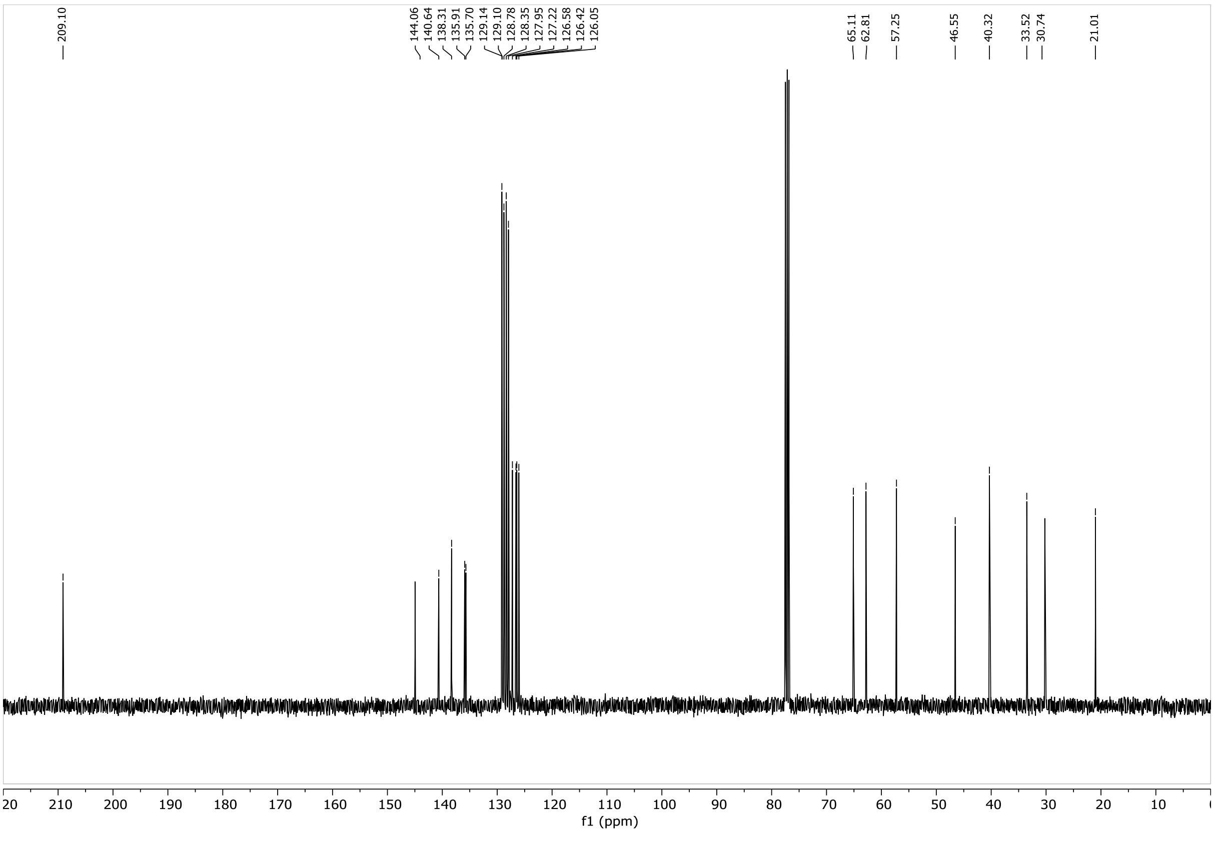

209.10
144.06
140.64
138.31
135.91
135.70
129.14
129.10
128.78
128.35
127.95
127.22
126.58
126.42
126.05
65.11
62.81
57.25
46.55
40.32
33.52
30.74
21.01
f1 (ppm)

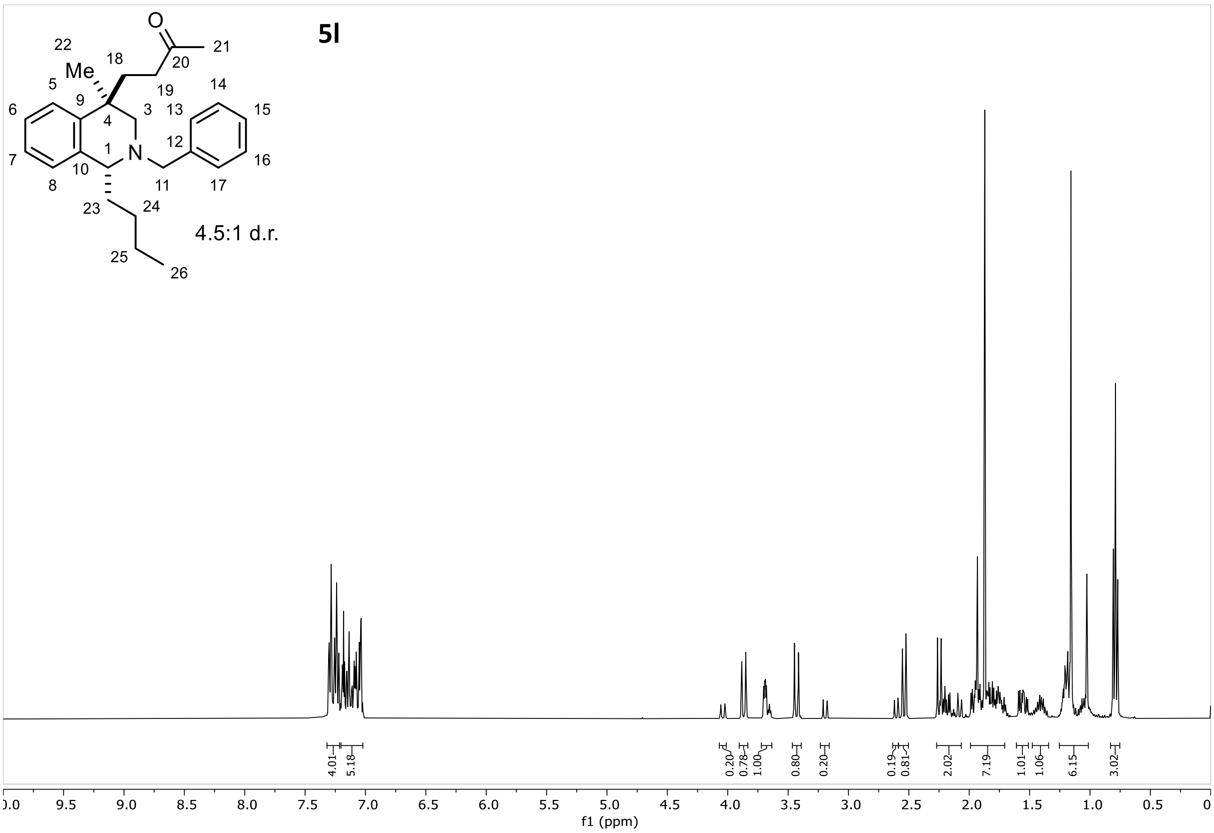
5l
4.5:1 d.r.
Me
O
N
f1 (ppm)
4.01
5.18
0.20
0.78
1.00
0.80
0.20
0.19
0.81
2.02
7.19
1.01
1.06
6.15
3.02

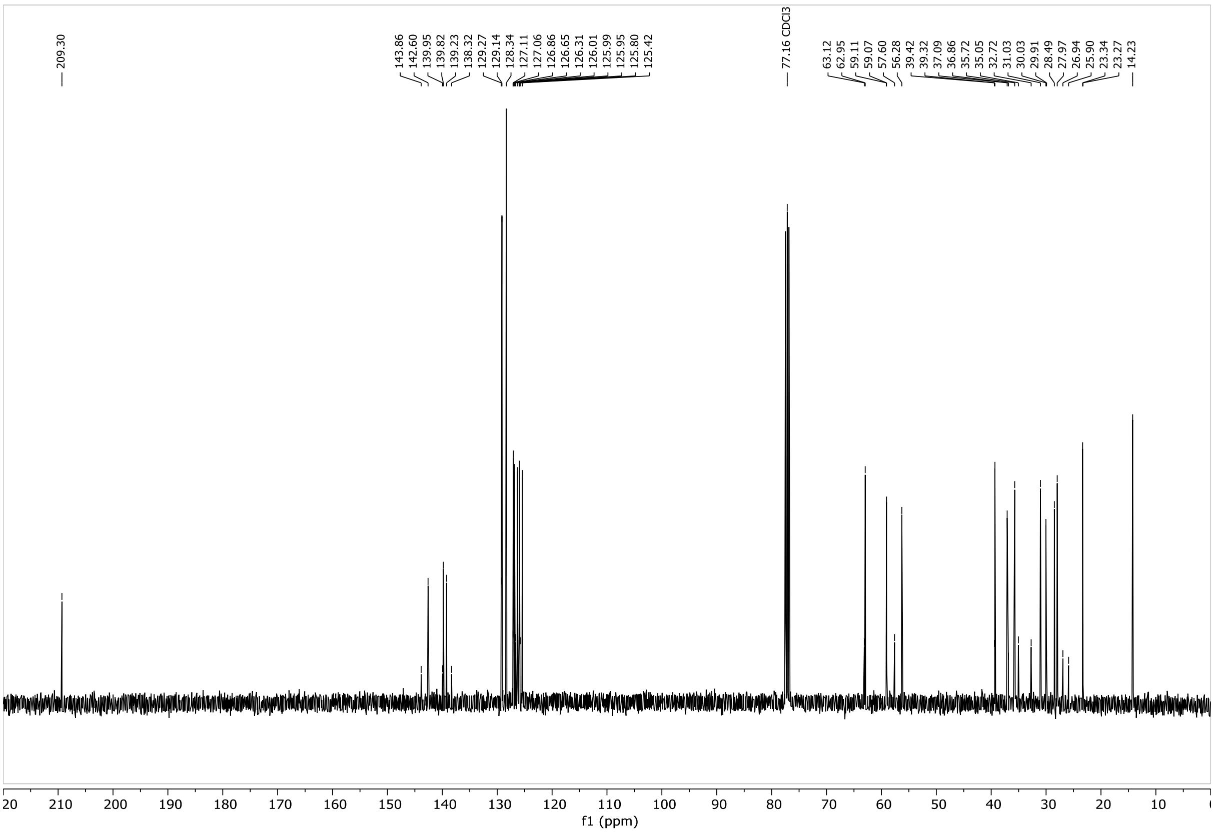
209.30
143.86
142.60
139.95
139.82
139.23
138.32
129.27
129.14
128.34
127.11
127.06
126.86
126.65
126.31
126.01
125.99
125.95
125.80
125.42
77.16 CDCl3
63.12
62.95
59.11
59.07
57.60
56.28
39.42
39.32
37.09
36.86
35.72
35.05
32.72
31.03
30.03
29.91
28.49
27.97
26.94
25.90
23.34
23.27
14.23
f1 (ppm)

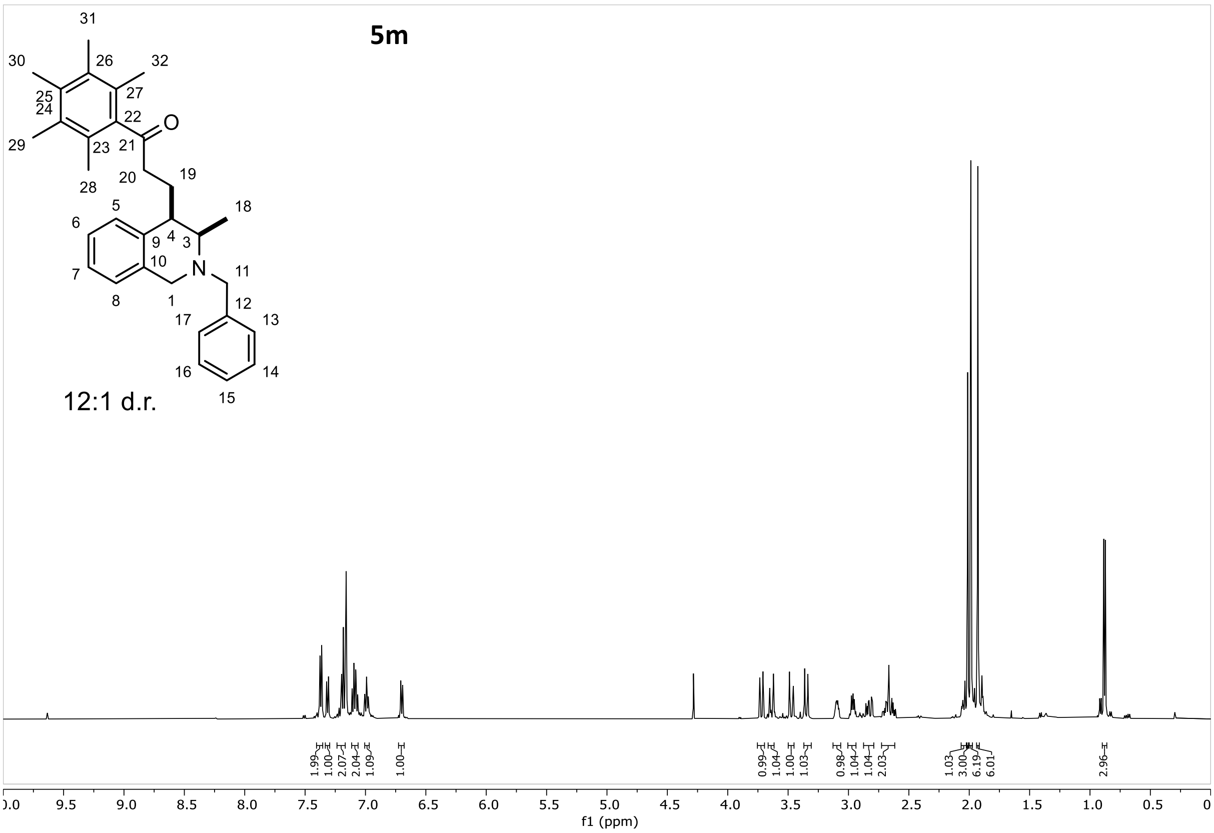
5m
12:1 d.r.
f1 (ppm)

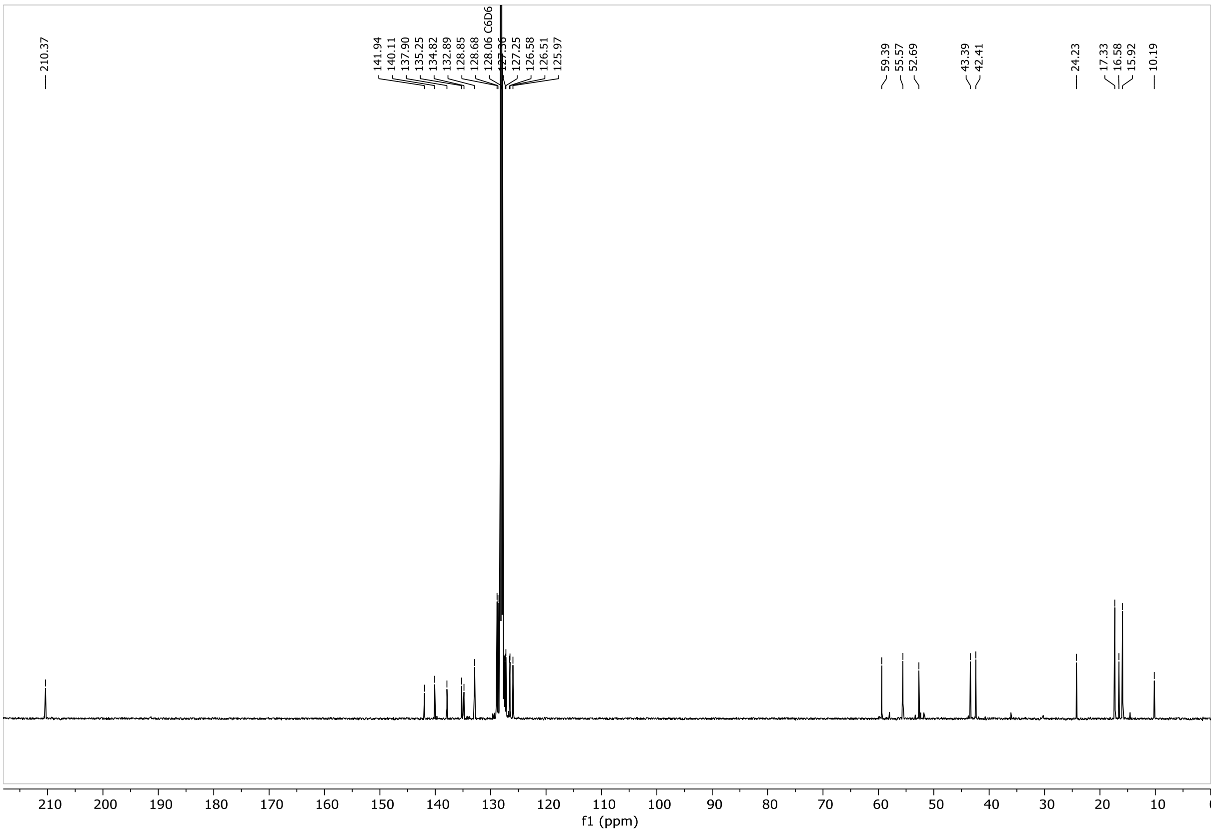
210.37
141.94
140.11
137.90
135.25
134.82
132.89
128.85
128.68
128.06 C6D6
127.25
126.58
126.51
125.97
59.39
55.57
52.69
43.39
42.41
24.23
17.33
16.58
15.92
10.19
f1 (ppm)

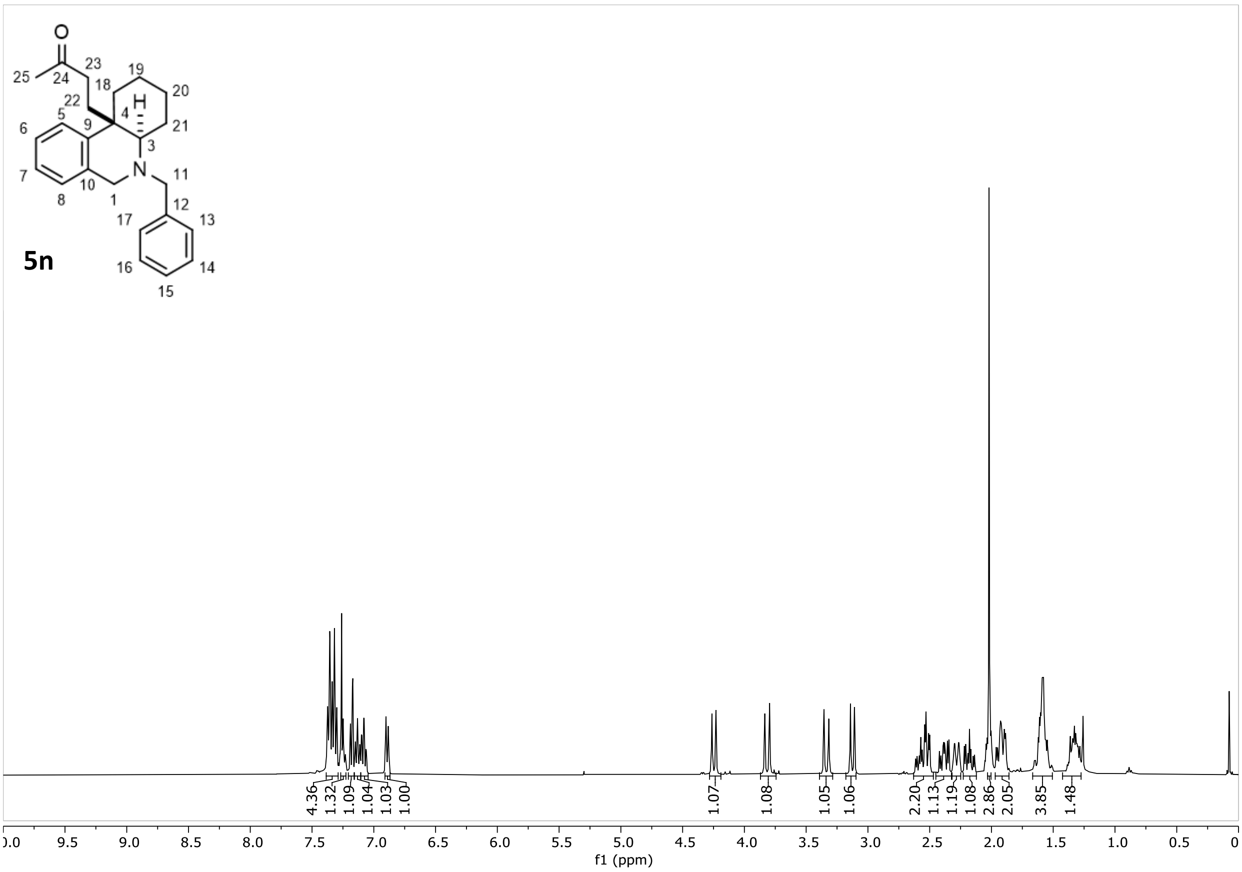
5n
4.36
1.32
1.09
1.04
1.03
1.00
1.07
1.08
1.05
1.06
2.20
1.13
1.19
1.08
2.86
2.05
3.85
1.48
f1 (ppm)

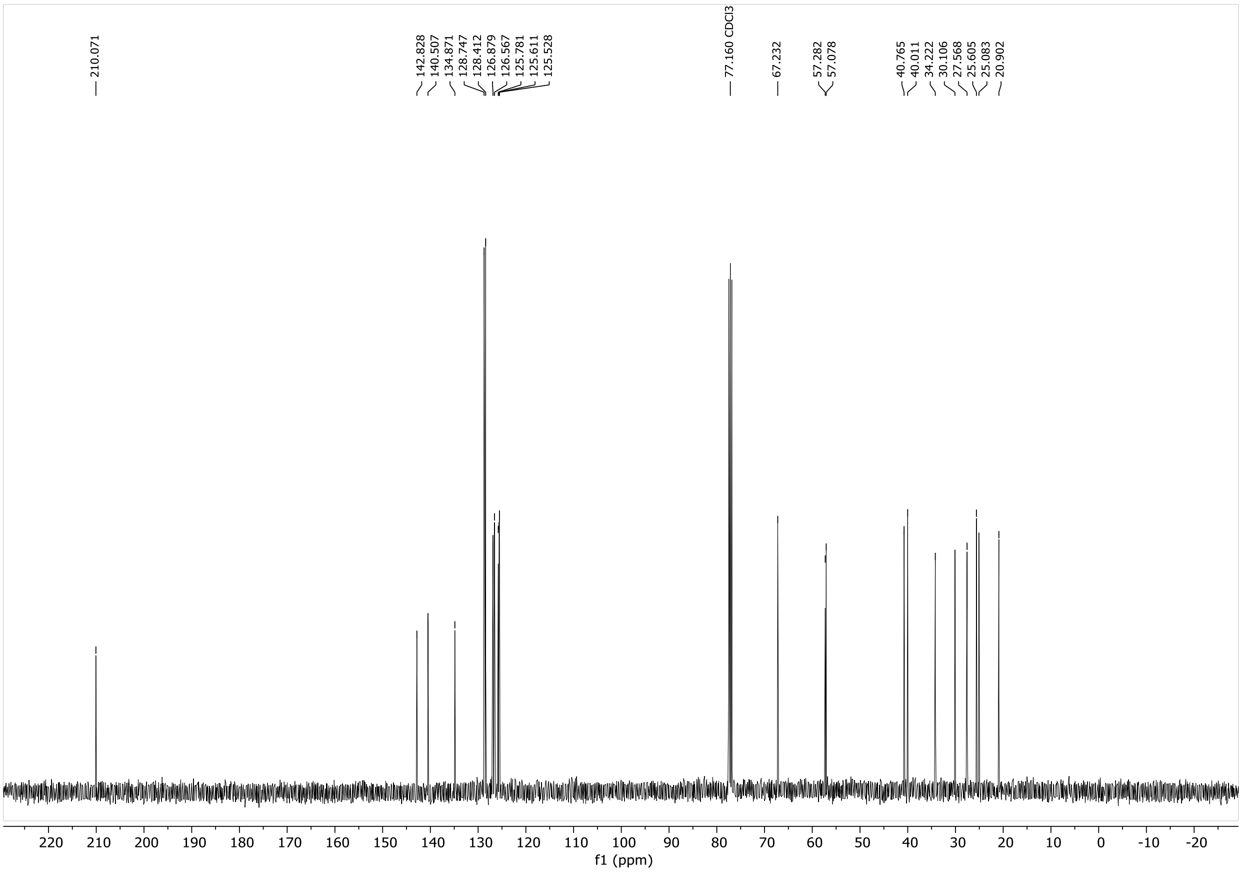
210.071
142.828
140.507
134.871
128.747
128.412
126.879
126.567
125.781
125.611
125.528
77.160 CDCl3
67.232
57.282
57.078
40.765
40.011
34.222
30.106
27.568
25.605
25.083
20.902
f1 (ppm)

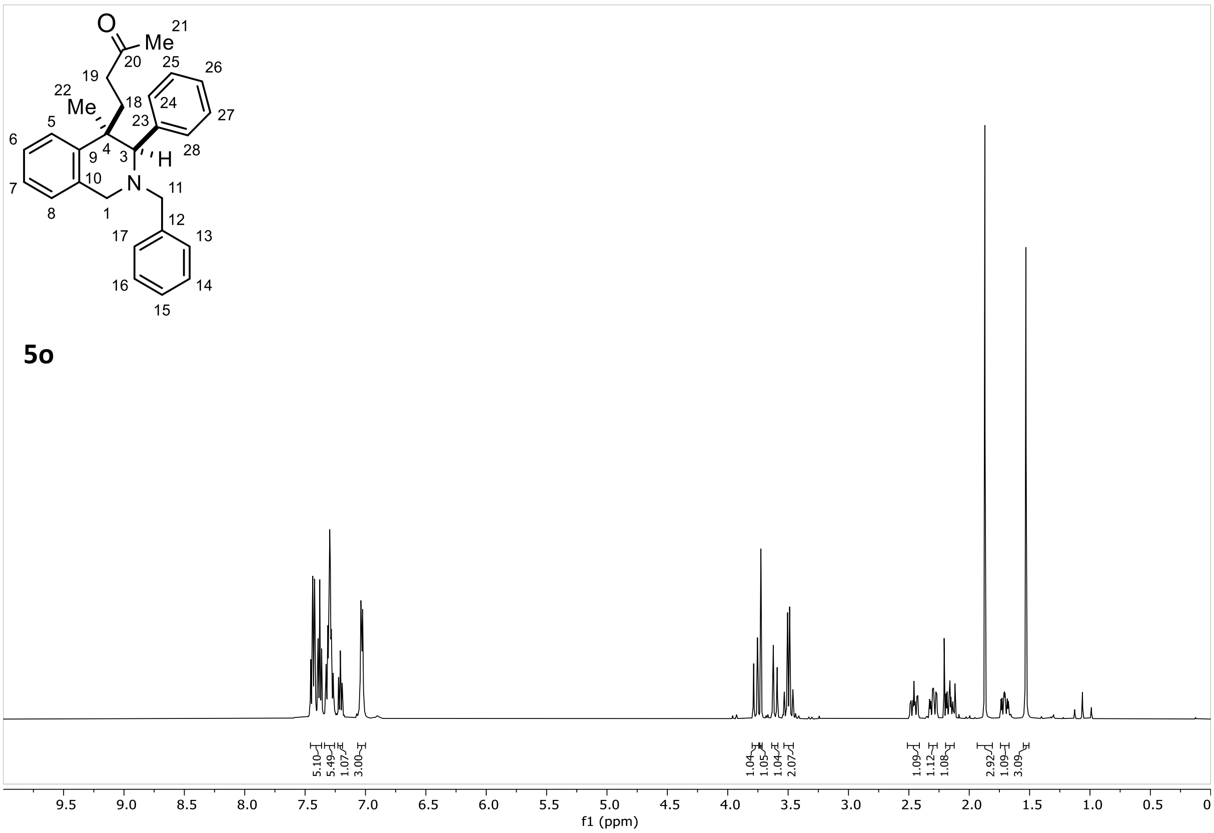

5o
1.04
1.05
1.04
2.07
5.10
5.49
1.07
3.00
1.09
1.12
1.08
2.92
1.09
3.09
f1 (ppm)

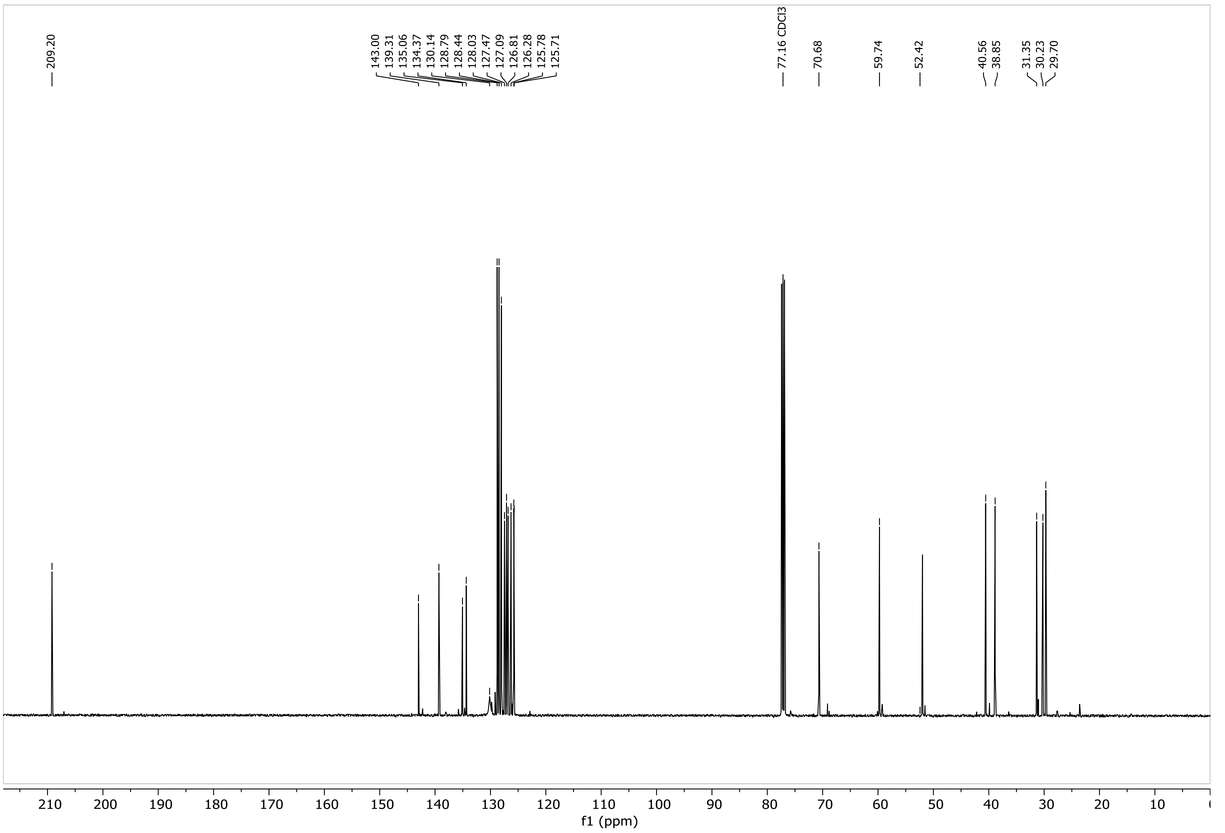

209.20
143.00
139.31
135.06
134.37
130.14
128.79
128.44
128.03
127.47
127.09
126.81
126.28
125.78
125.71
77.16 CDCl3
70.68
59.74
52.42
40.56
38.85
31.35
30.23
29.70
f1 (ppm)

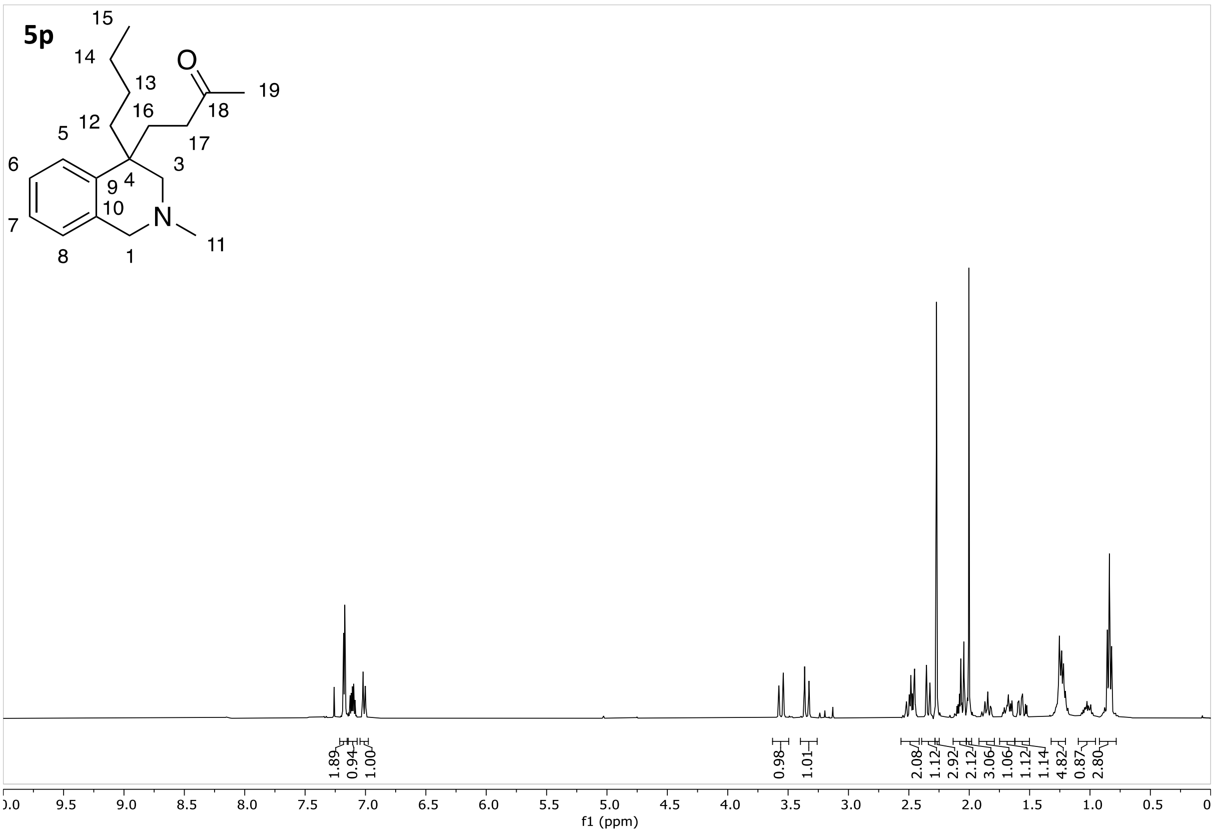
5p
15
14
13
16
19
18
12
5
17
6
3
9
4
10
7
N
11
8
1
O
1.89
0.94
1.00
0.98
1.01
2.08
1.12
2.92
2.12
3.06
1.06
1.12
1.14
4.82
0.87
2.80
f1 (ppm)

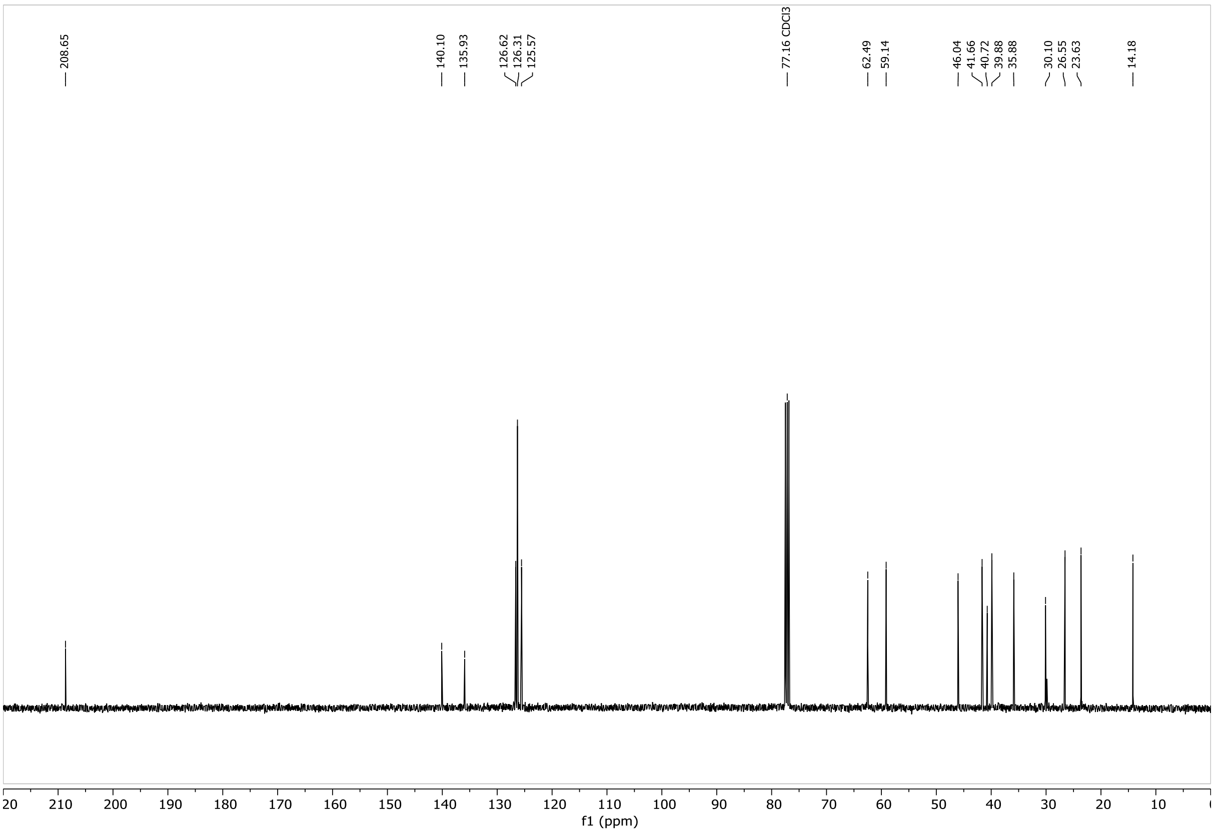
208.65
140.10
135.93
126.62
126.31
125.57
77.16 CDCl3
62.49
59.14
46.04
41.66
40.72
39.88
35.88
30.10
26.55
23.63
14.18
f1 (ppm)

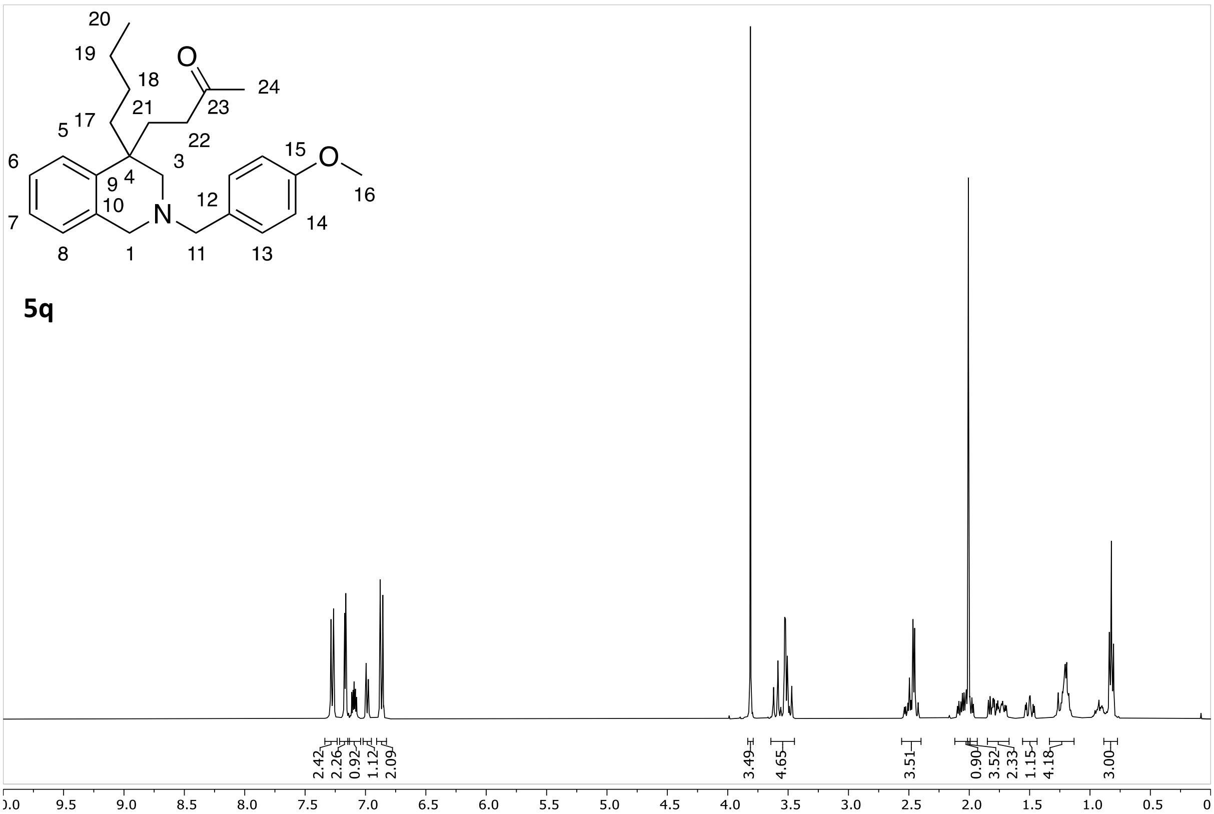

5q
2.42
2.26
0.92
1.12
2.09
3.49
4.65
3.51
0.90
3.52
2.33
1.15
4.18
3.00
f1 (ppm)

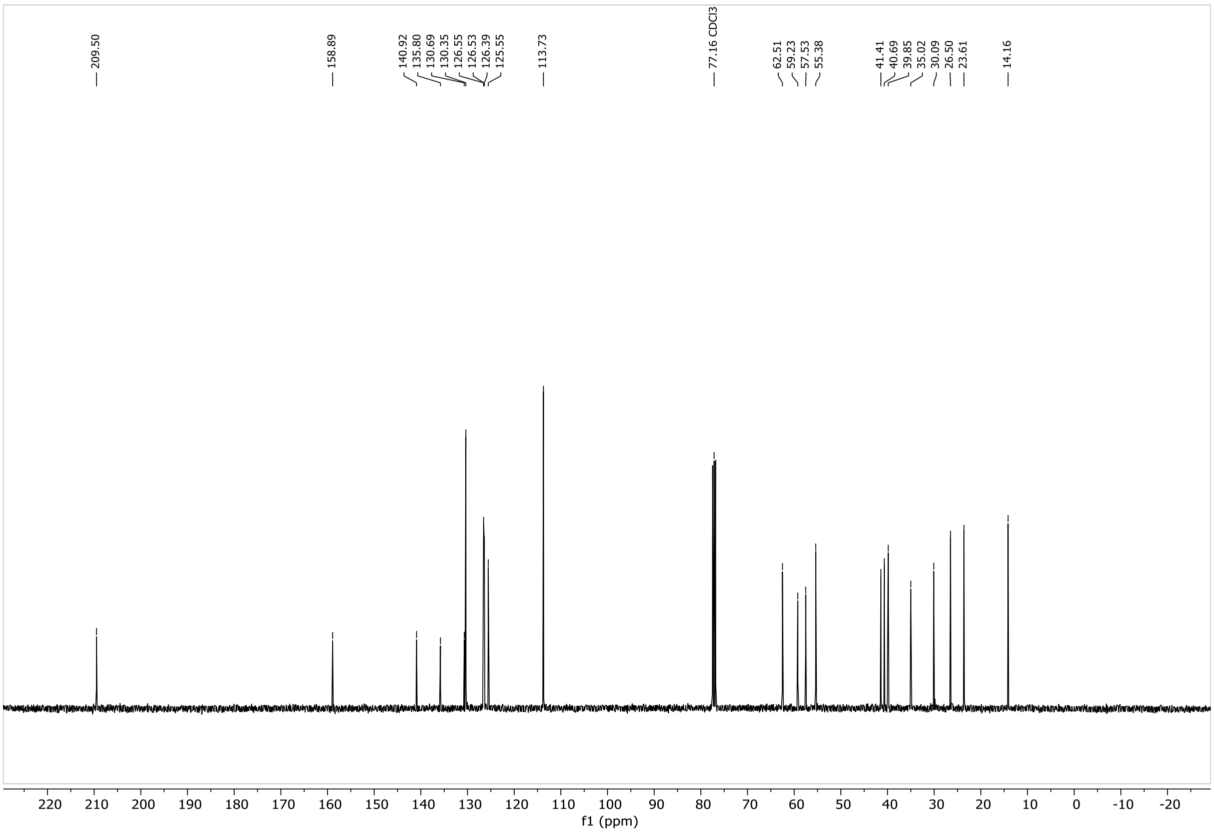

209.50
158.89
140.92
135.80
130.69
130.35
126.55
126.53
126.39
125.55
113.73
77.16 CDCl3
62.51
59.23
57.53
55.38
41.41
40.69
39.85
35.02
30.09
26.50
23.61
14.16
f1 (ppm)

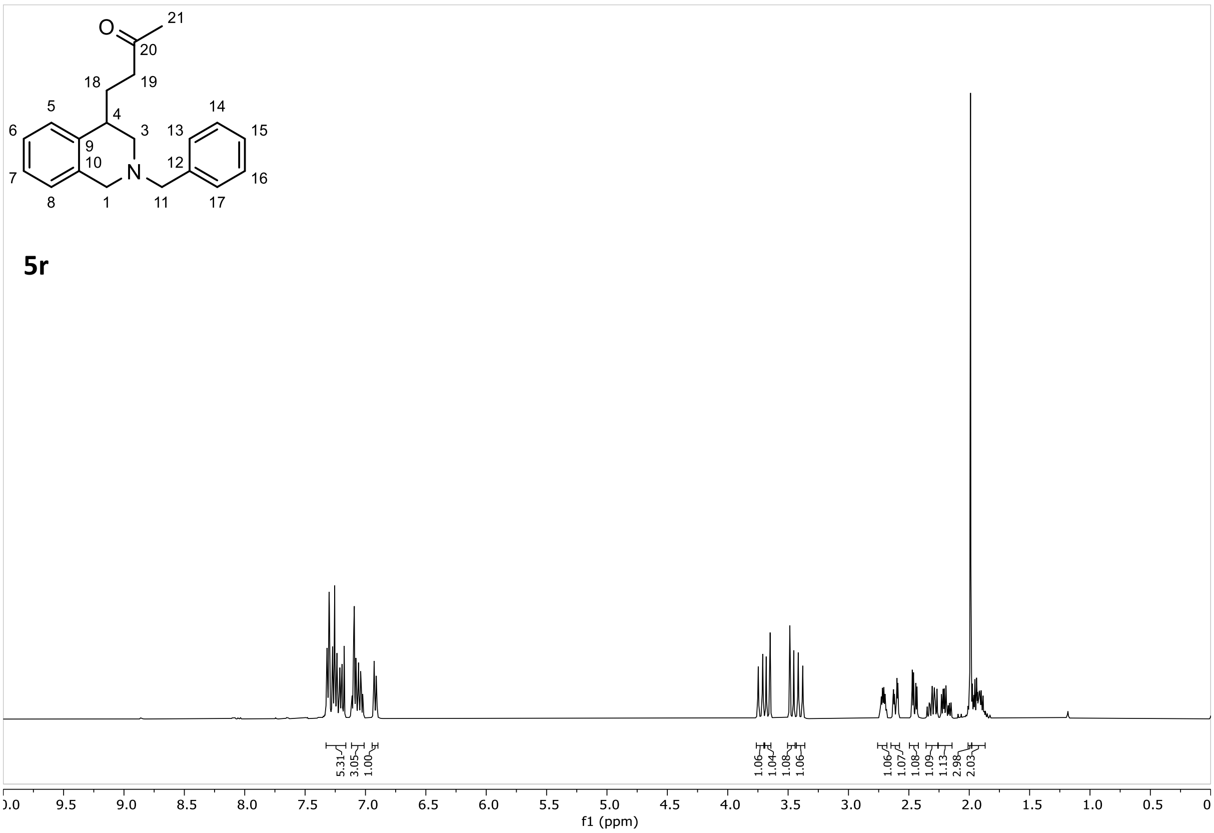
5r
O
N
21
20
19
18
5
4
6
3
13
14
15
9
10
12
7
16
8
1
11
17
5.31
3.05
1.00
1.06
1.04
1.08
1.06
1.06
1.07
1.08
1.09
1.13
2.98
2.03
f1 (ppm)

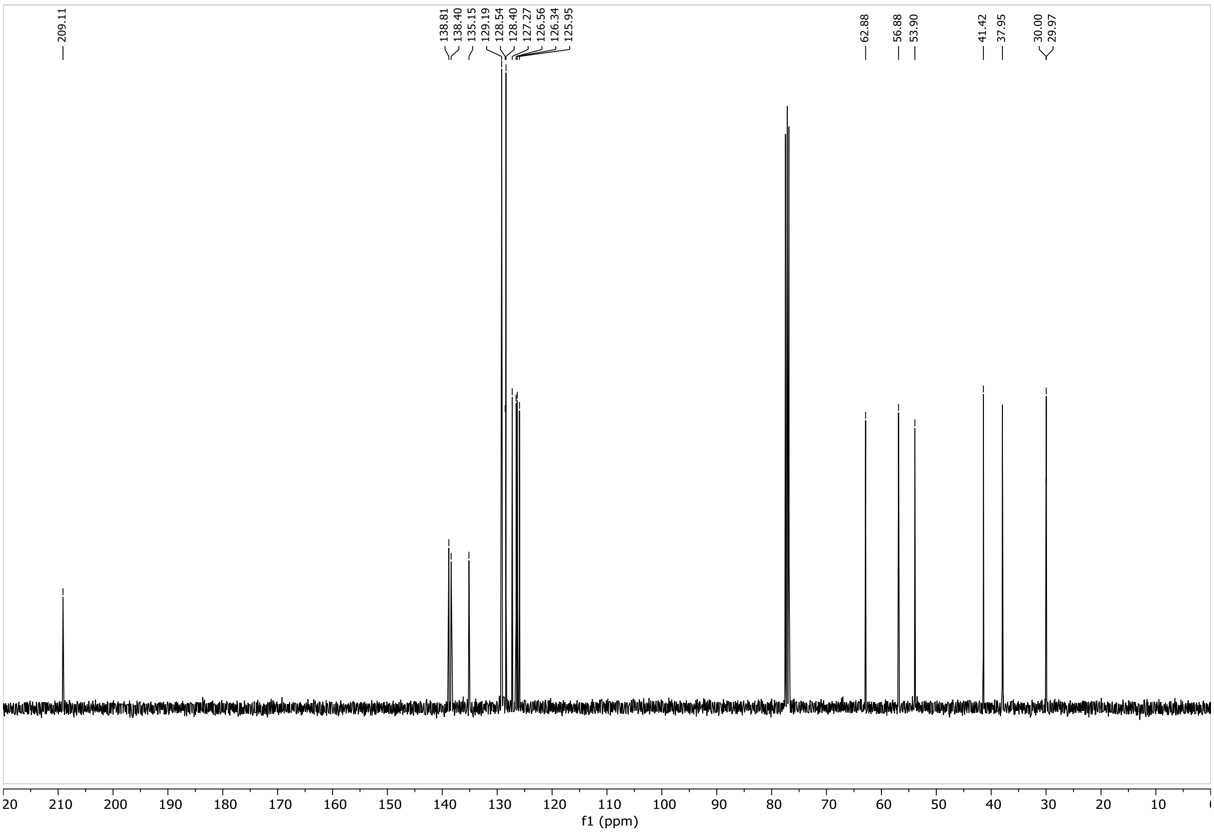
209.11
138.81
138.40
135.15
129.19
128.54
128.40
127.27
126.56
126.34
125.95
62.88
56.88
53.90
41.42
37.95
30.00
29.97
f1 (ppm)

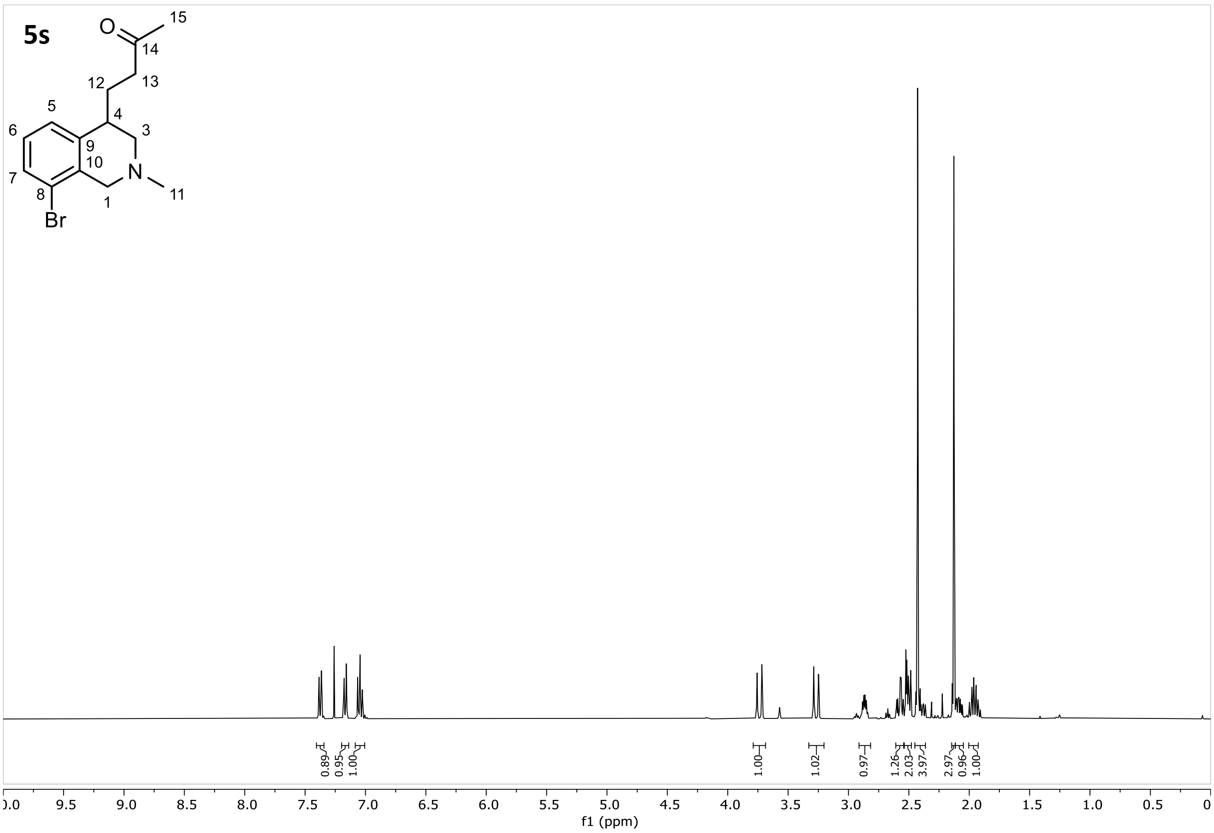
5s
O
15
14
12
13
5
4
6
3
9
10
N
7
8
11
1
Br
0.89
0.95
1.00
1.00
1.02
0.97
1.26
2.03
3.97
2.97
0.96
1.00
f1 (ppm)

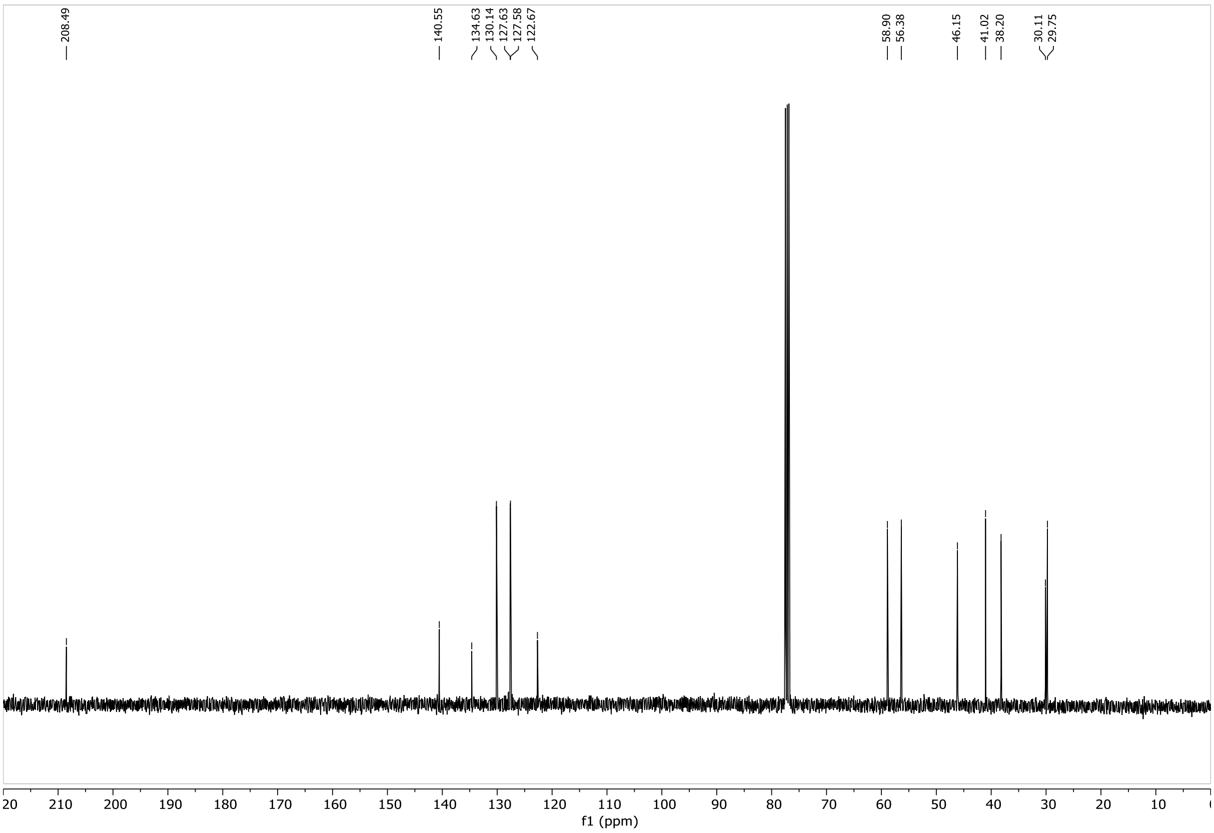
208.49
140.55
134.63
130.14
127.63
127.58
122.67
58.90
56.38
46.15
41.02
38.20
30.11
29.75
f1 (ppm)

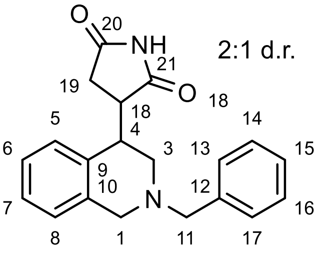
2:1 d.r.
O
NH
O
20
21
19
18
4
3
5
6
7
8
9
10
1
N
11
12
13
14
15
16
17

5t

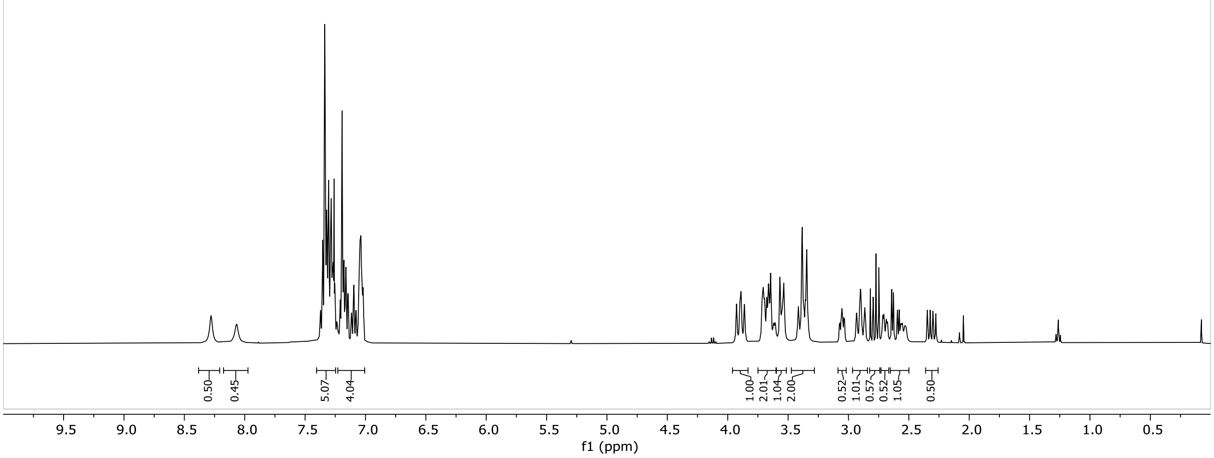
0.50
0.45
5.07
4.04
1.00
2.01
1.04
2.00
0.52
1.01
0.57
0.52
1.05
0.50
9.5 9.0 8.5 8.0 7.5 7.0 6.5 6.0 5.5 5.0 4.5 4.0 3.5 3.0 2.5 2.0 1.5 1.0 0.5
f1 (ppm)

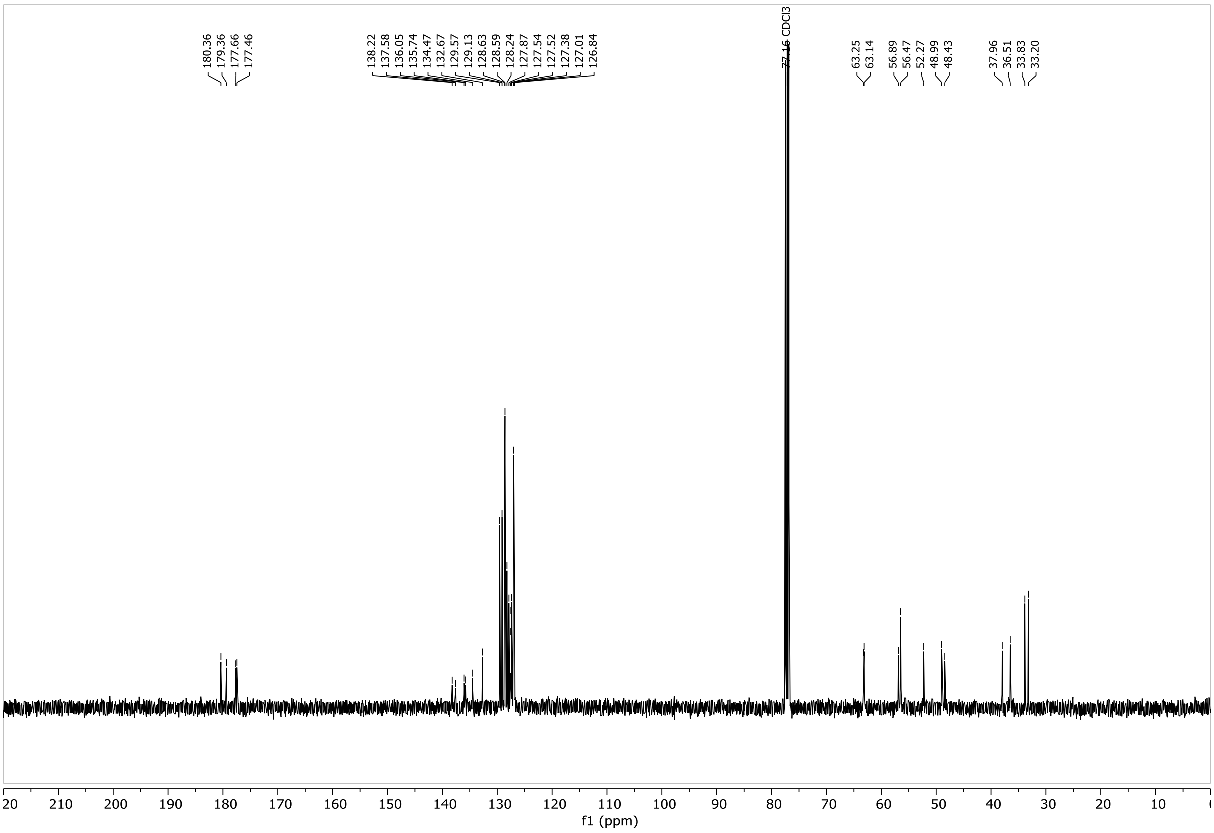
180.36
179.36
177.66
177.46
138.22
137.58
136.05
135.74
134.47
132.67
129.57
129.13
128.63
128.59
128.24
127.87
127.54
127.52
127.38
127.01
126.84
77.16 CDCl3
63.25
63.14
56.89
56.47
52.27
48.99
48.43
37.96
36.51
33.83
33.20
20 210 200 190 180 170 160 150 140 130 120 110 100 90 80 70 60 50 40 30 20 10
f1 (ppm)

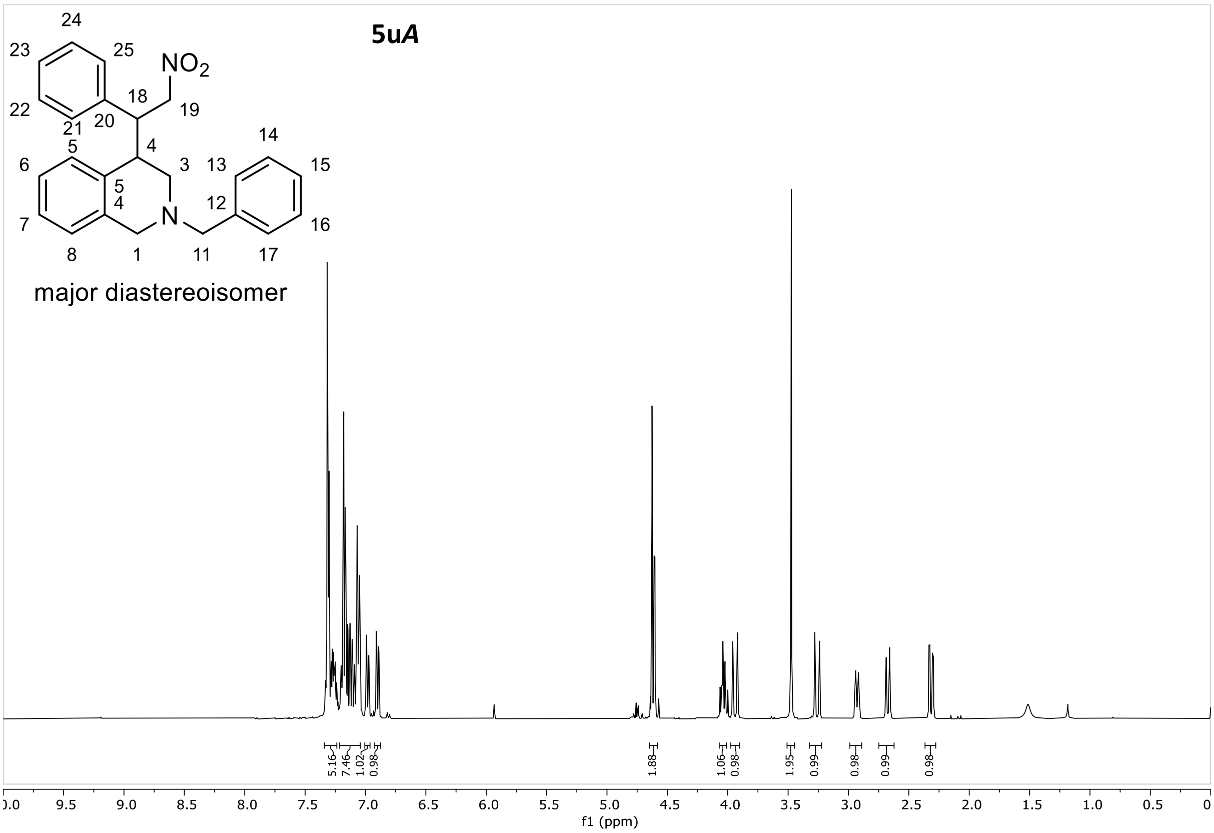
5u*A*
major diastereoisomer
f1 (ppm)

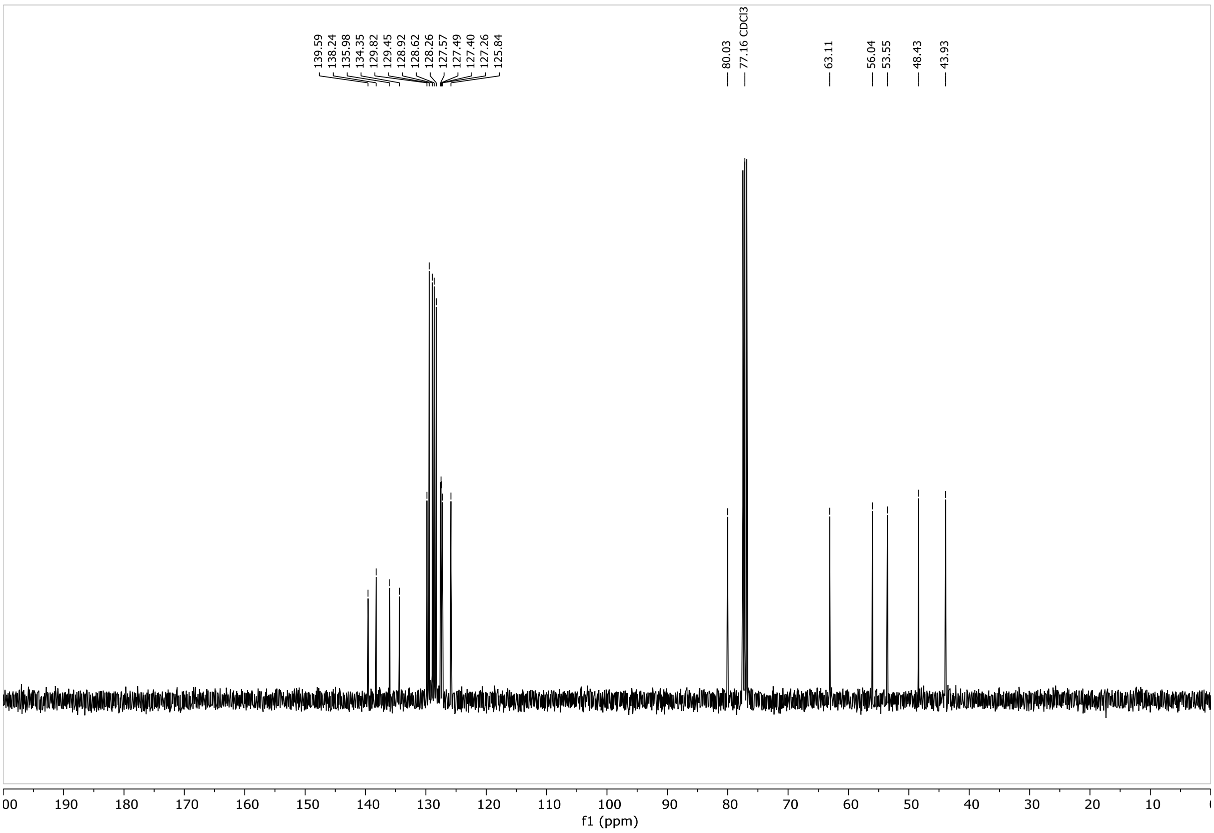
139.59
138.24
135.98
134.35
129.82
129.45
128.92
128.62
128.26
127.57
127.49
127.40
127.26
125.84
80.03
77.16 CDCl3
63.11
56.04
53.55
48.43
43.93
f1 (ppm)

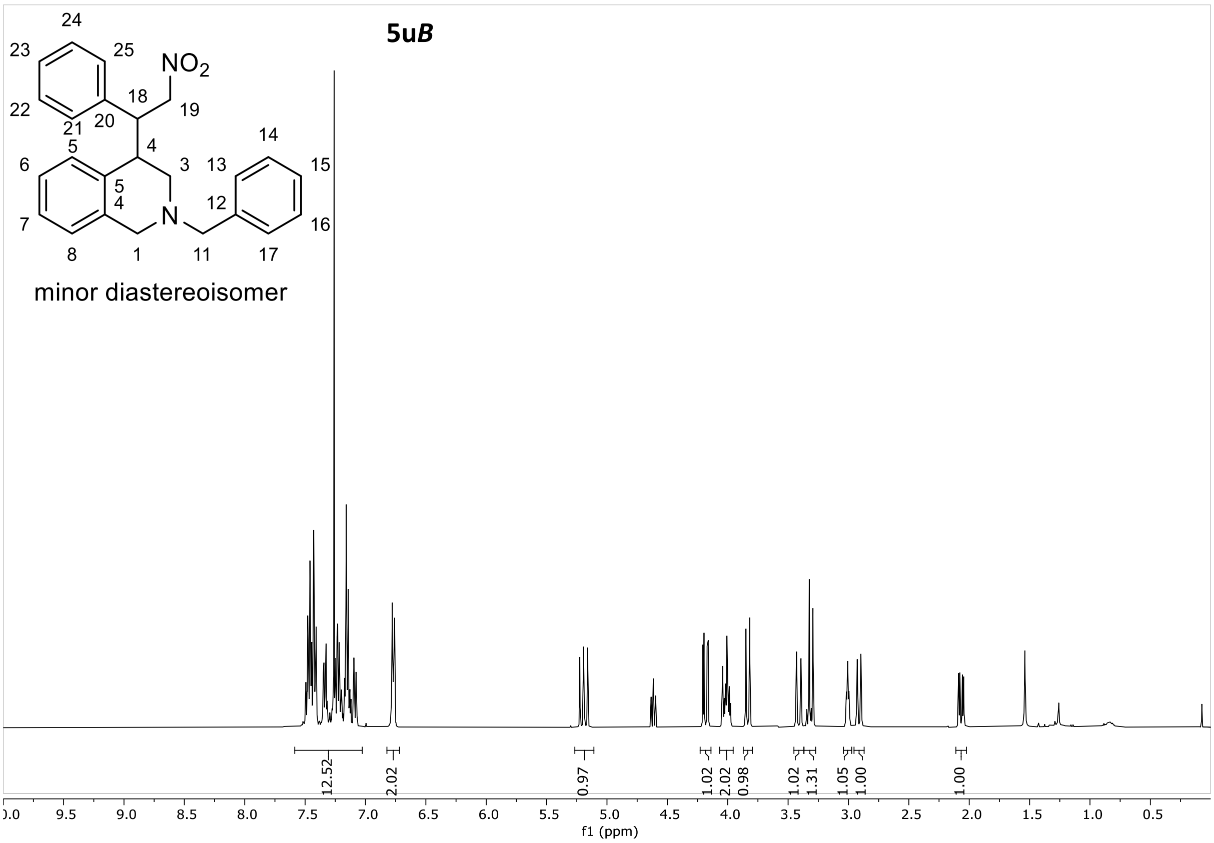
5u*B*
NO2
minor diastereoisomer
12.52
2.02
0.97
1.02
2.02
0.98
1.02
1.31
1.05
1.00
1.00
f1 (ppm)

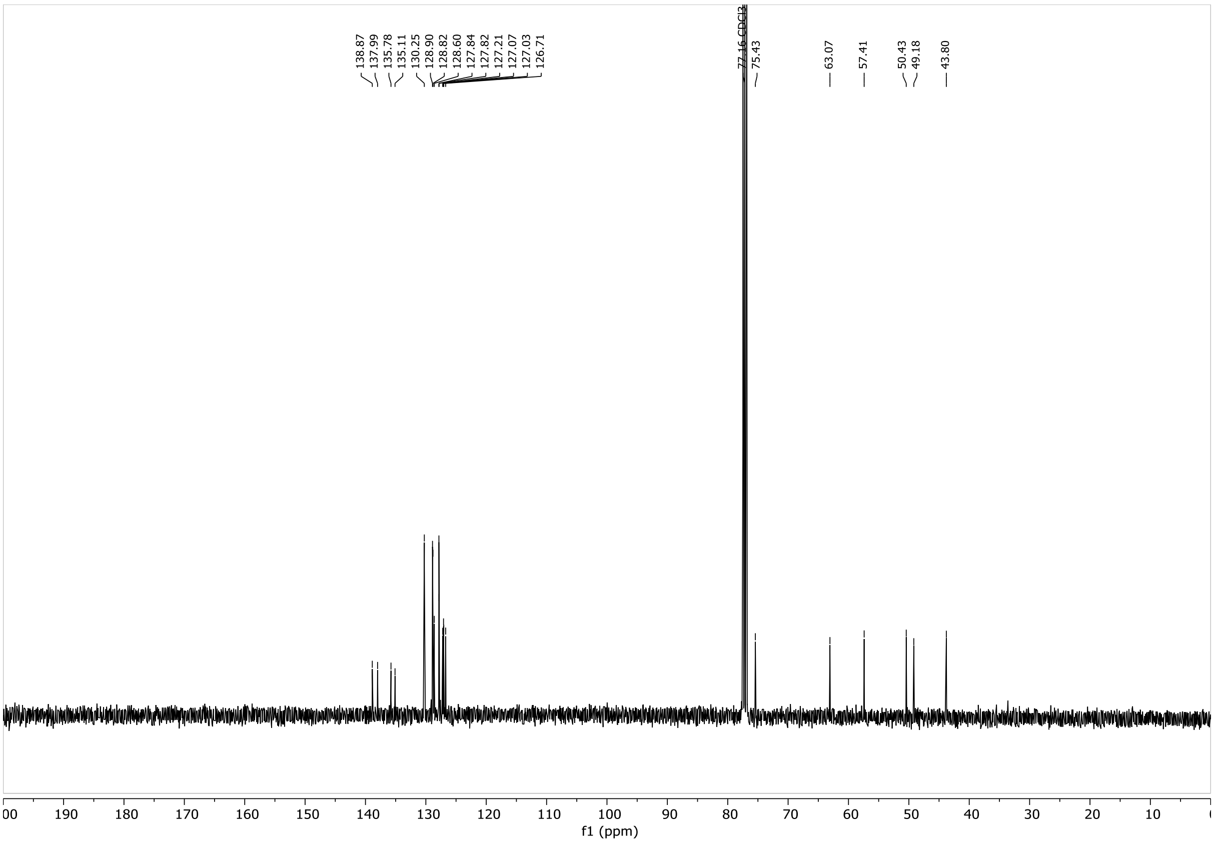
138.87
137.99
135.78
135.11
130.25
128.90
128.82
128.60
127.84
127.82
127.21
127.07
127.03
126.71
77.16 CDCl3
75.43
63.07
57.41
50.43
49.18
43.80
f1 (ppm)

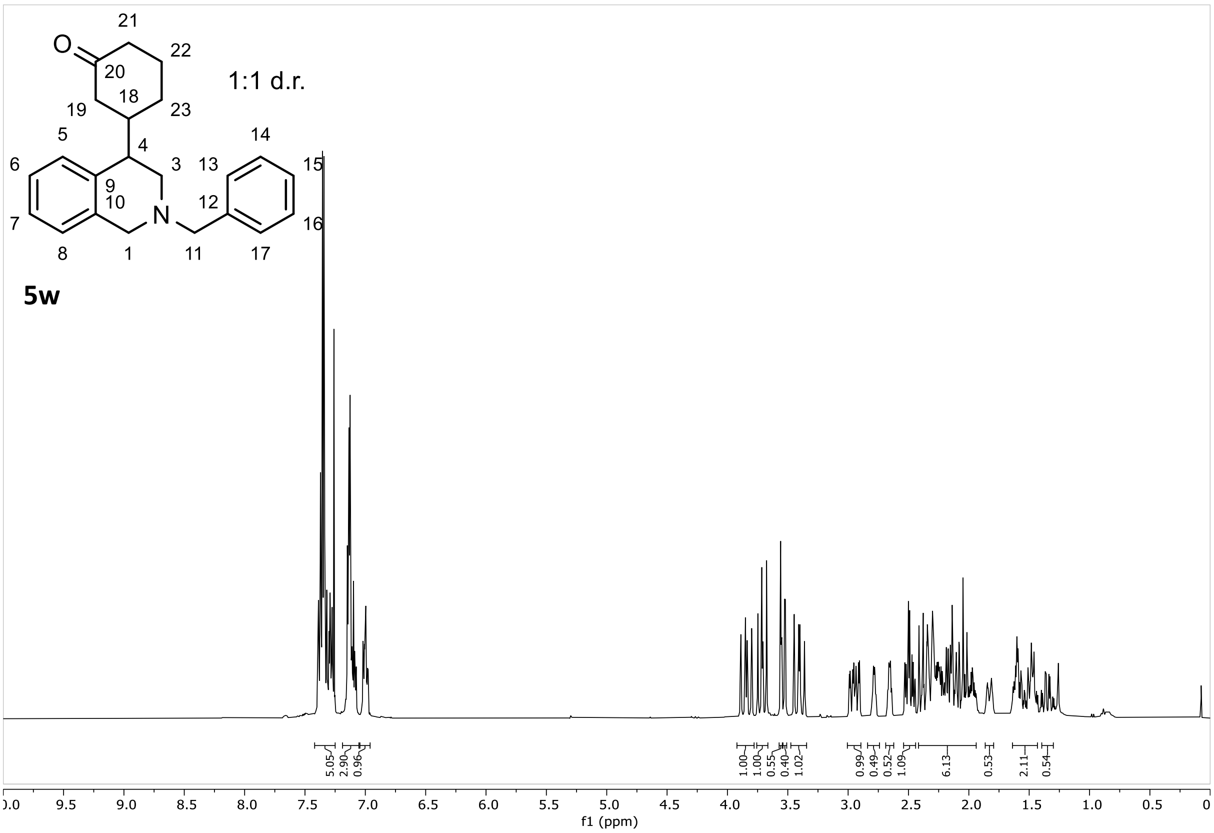
5w
1:1 d.r.
5.05
2.90
0.96
1.00
1.00
0.55
0.40
1.02
0.99
0.49
0.52
1.09
6.13
0.53
2.11
0.54
f1 (ppm)

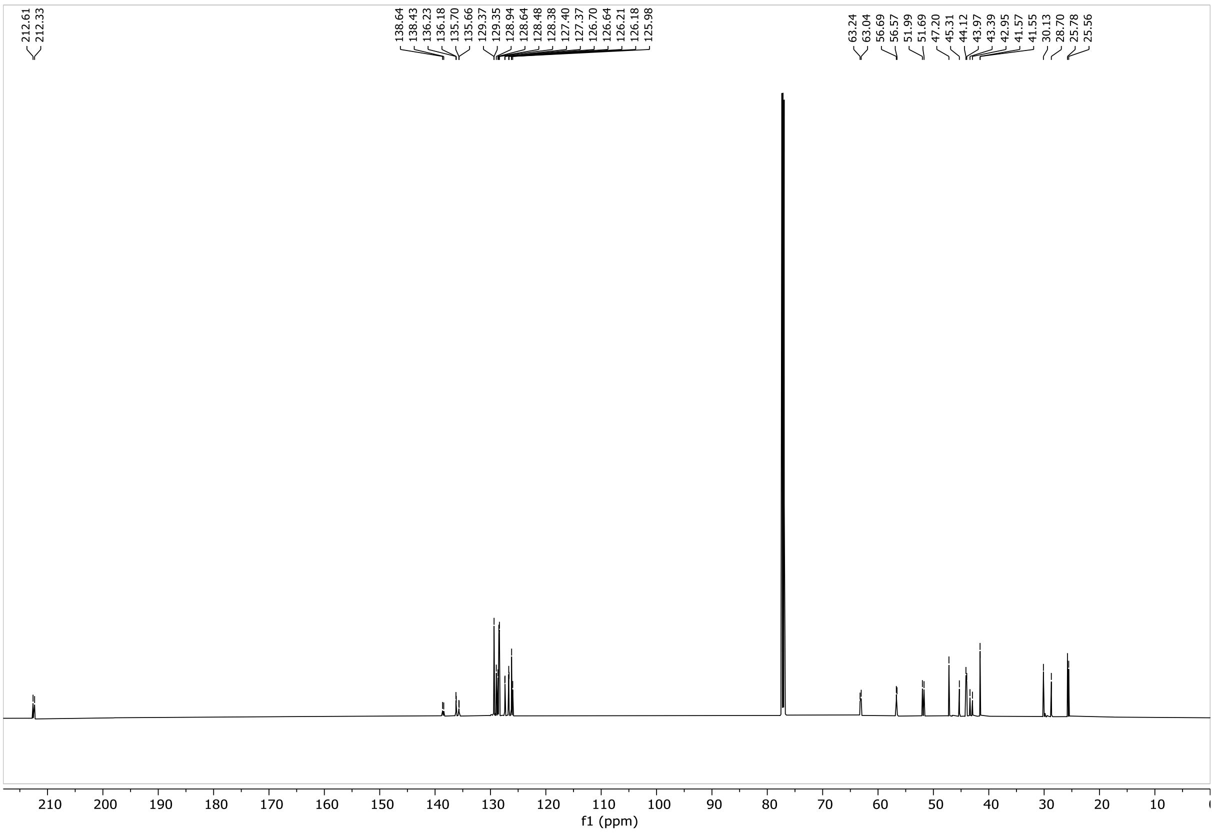
212.61
212.33
138.64
138.43
136.23
136.18
135.70
135.66
129.37
129.35
128.94
128.64
128.48
128.38
127.40
127.37
126.70
126.64
126.21
126.18
125.98
63.24
63.04
56.69
56.57
51.99
51.69
47.20
45.31
44.12
43.97
43.39
42.95
41.57
41.55
30.13
28.70
25.78
25.56
f1 (ppm)

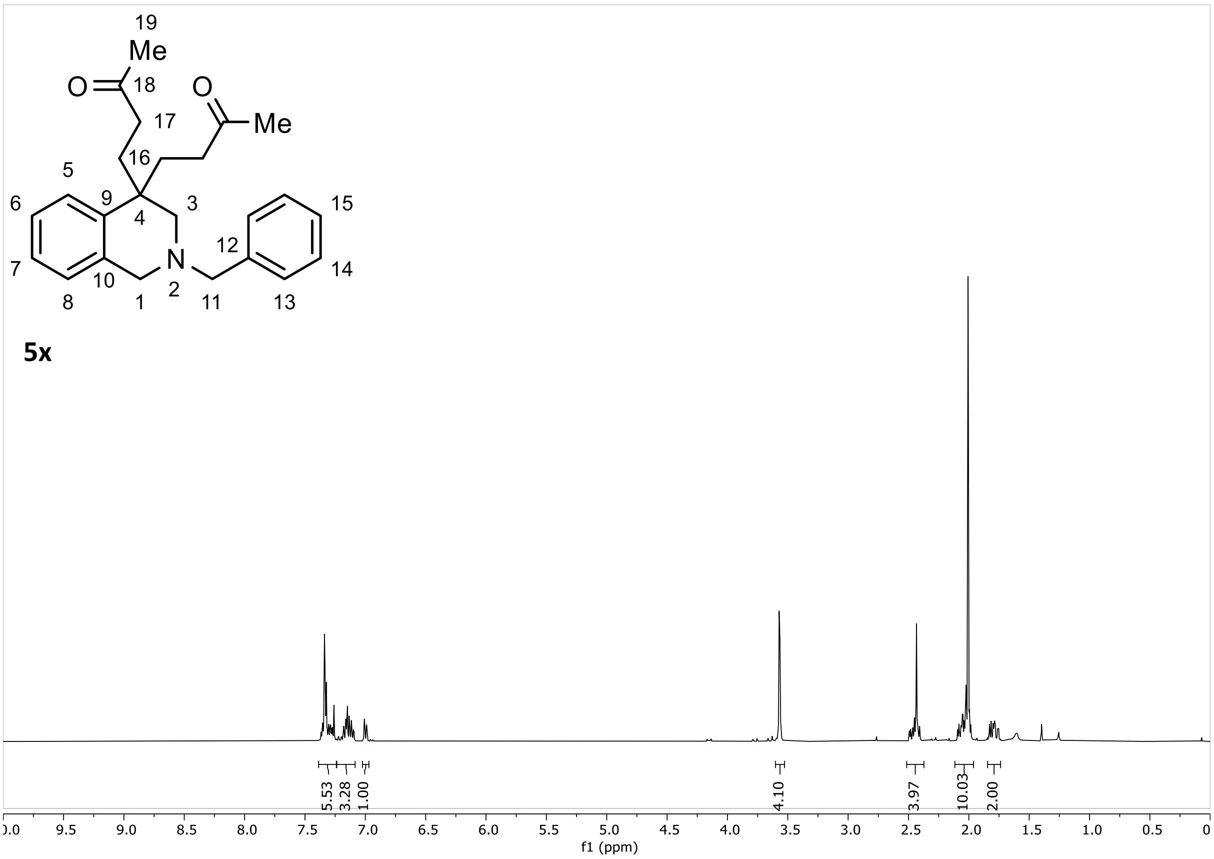
5x
Me
O
Me
O
5.53
3.28
1.00
4.10
3.97
10.03
2.00
f1 (ppm)

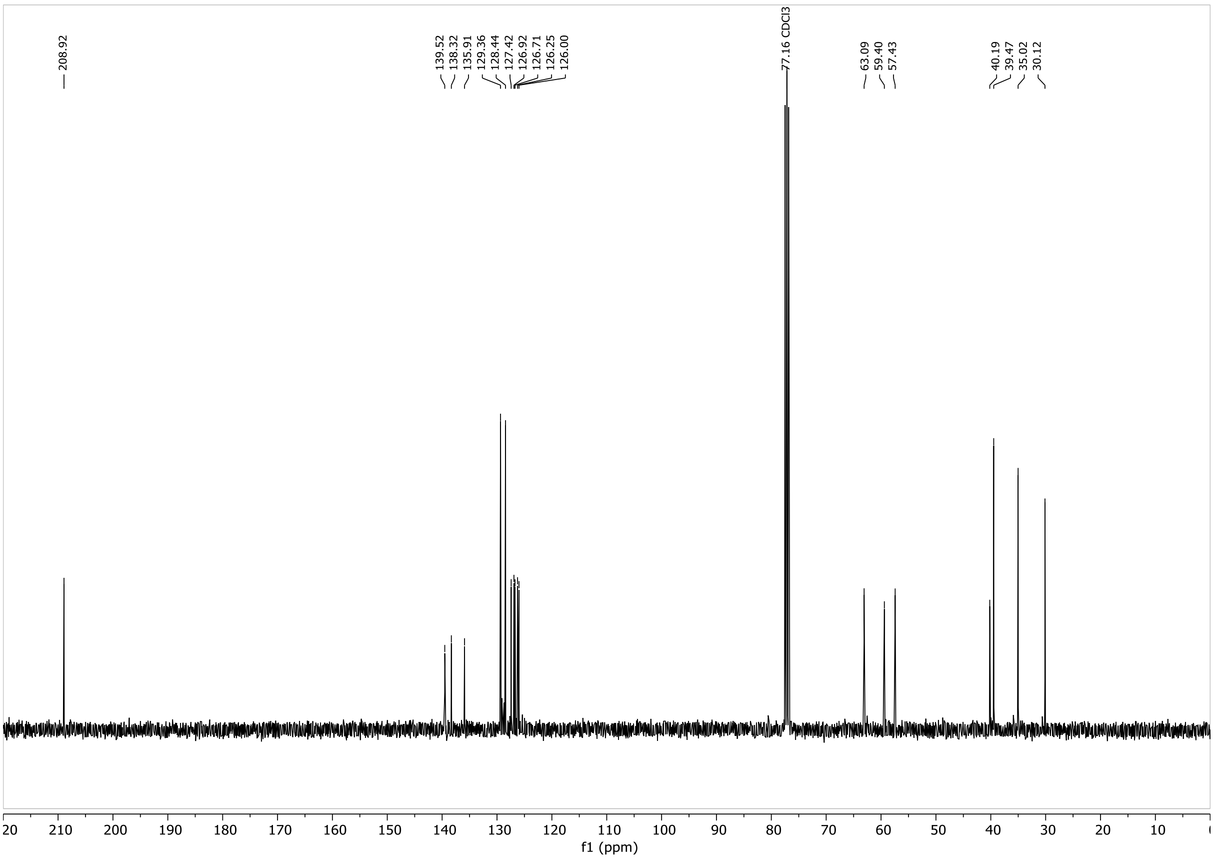
208.92
139.52
138.32
135.91
129.36
128.44
127.42
126.92
126.71
126.25
126.00
77.16 CDCl3
63.09
59.40
57.43
40.19
39.47
35.02
30.12
f1 (ppm)

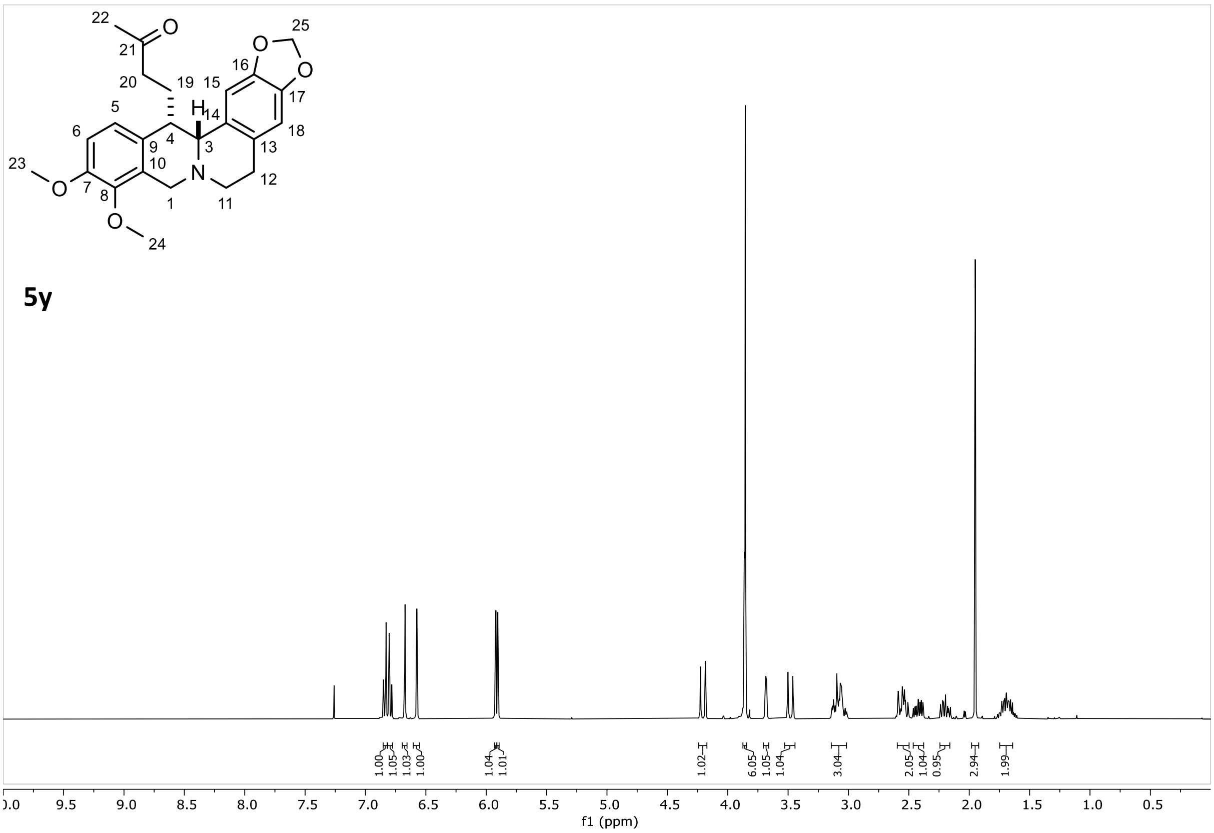
5y
1.00
1.05
1.03
1.00
1.04
1.01
1.02
6.05
1.05
1.04
3.04
2.05
1.04
0.95
2.94
1.99
f1 (ppm)

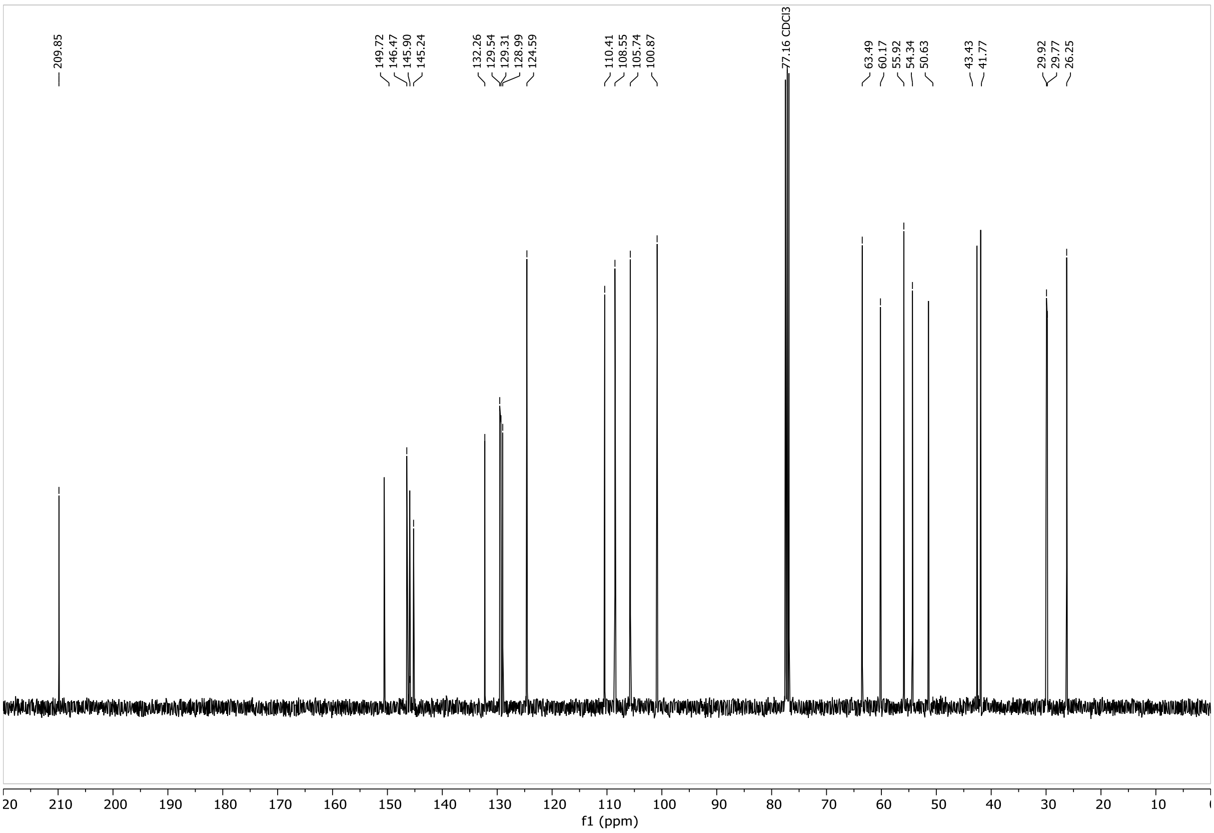
209.85
149.72
146.47
145.90
145.24
132.26
129.54
129.31
128.99
124.59
110.41
108.55
105.74
100.87
77.16 CDCl3
63.49
60.17
55.92
54.34
50.63
43.43
41.77
29.92
29.77
26.25
f1 (ppm)

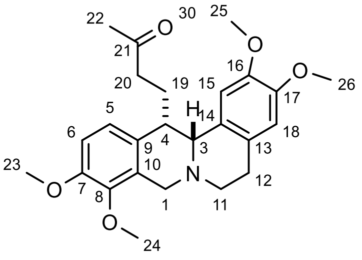
22
30
25
21
O
20
19
16
15
O
17
26
5
H
14
6
4
3
18
13
9
23
10
N
O
7
12
8
1
11
O
24

5z

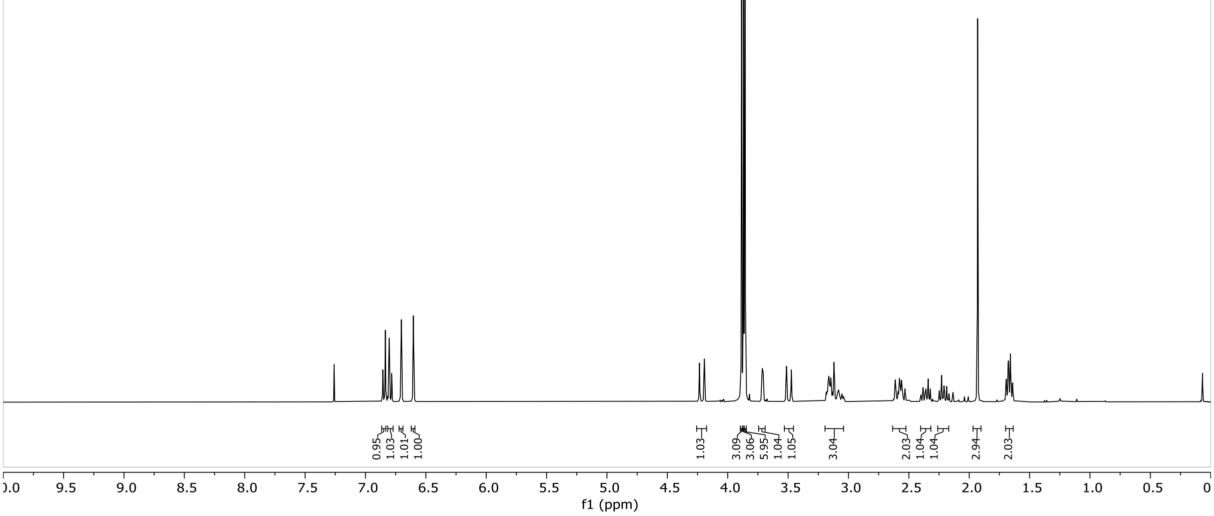
0.95
1.03
1.01
1.00
1.03
3.09
3.06
5.95
1.04
1.05
3.04
2.03
1.04
1.04
2.94
2.03
f1 (ppm)

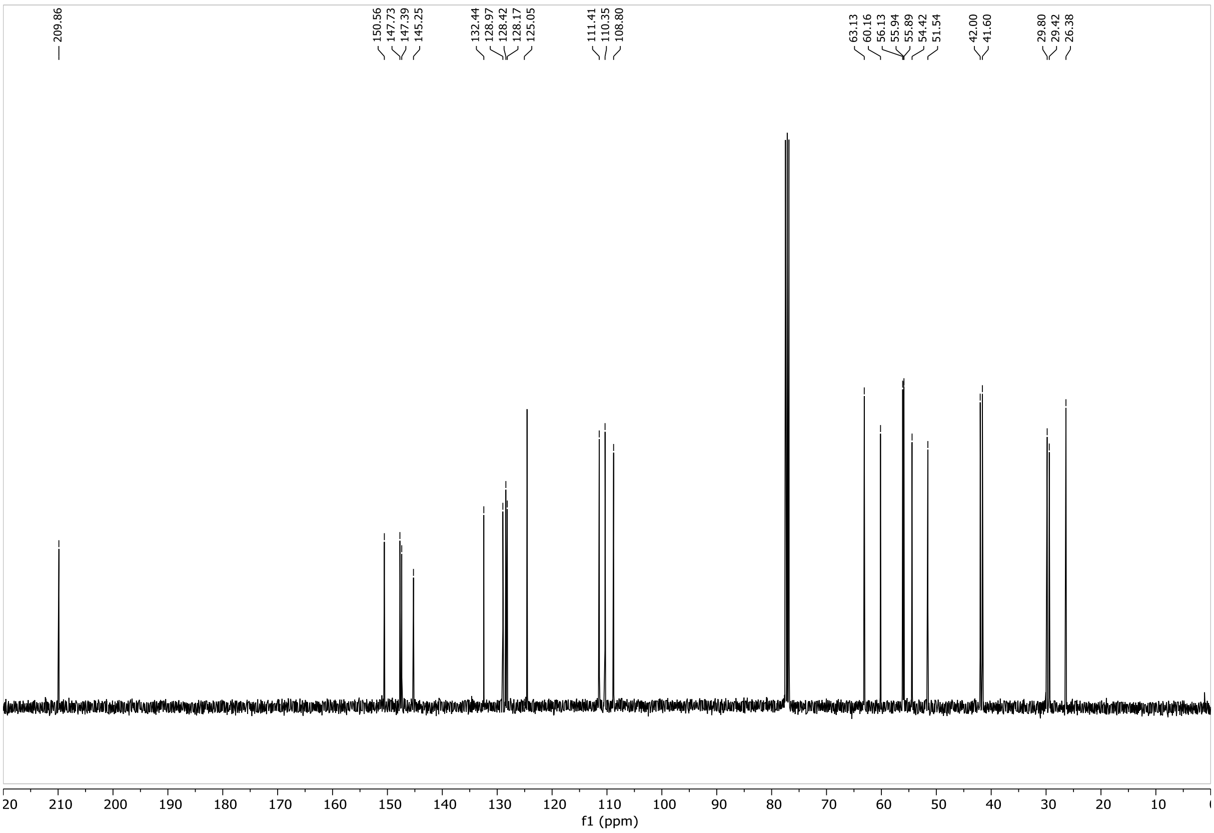
209.86
150.56
147.73
147.39
145.25
132.44
128.97
128.42
128.17
125.05
111.41
110.35
108.80
63.13
60.16
56.13
55.94
55.89
54.42
51.54
42.00
41.60
29.80
29.42
26.38
f1 (ppm)

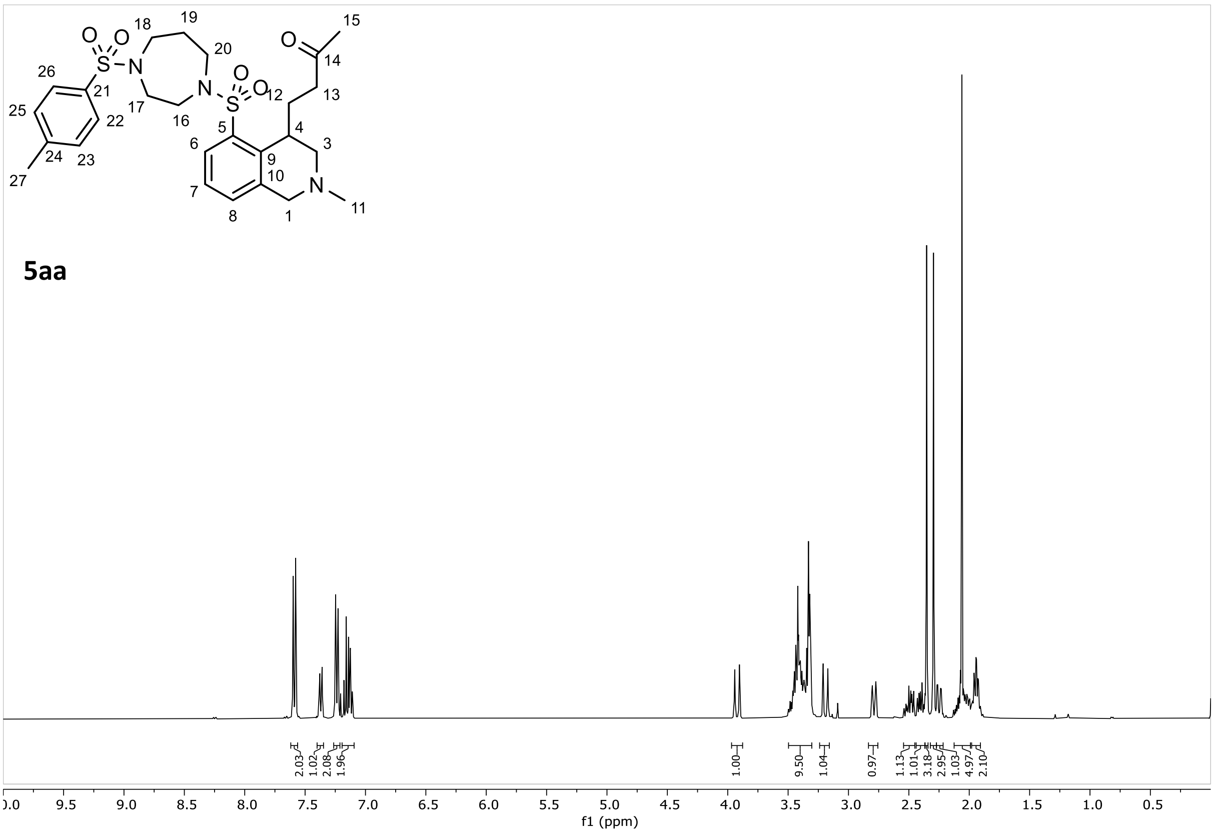
5aa
2.03
1.02
2.08
1.96
1.00
9.50
1.04
0.97
1.13
1.01
3.18
2.95
1.03
4.97
2.10
f1 (ppm)

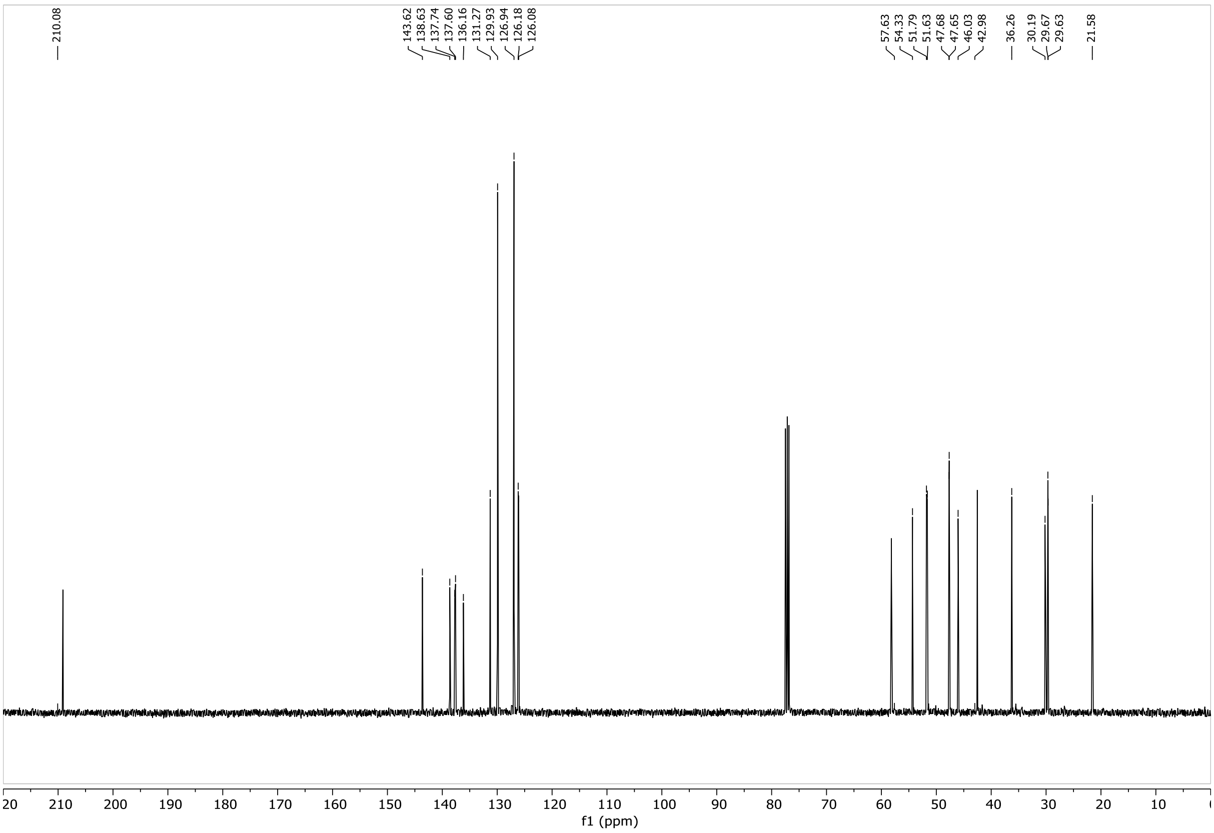
210.08
143.62
138.63
137.74
137.60
136.16
131.27
129.93
126.94
126.18
126.08
57.63
54.33
51.79
51.63
47.68
47.65
46.03
42.98
36.26
30.19
29.67
29.63
21.58
f1 (ppm)

6a

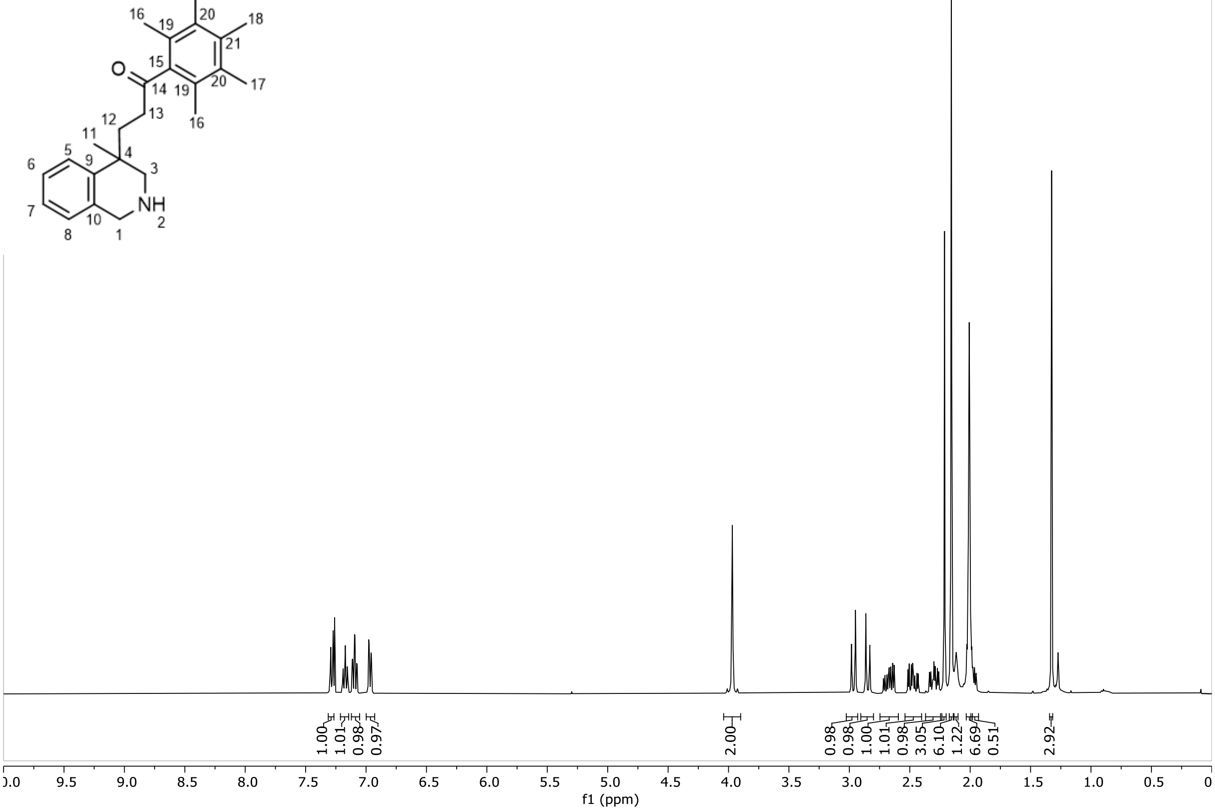
1.00
1.01
0.98
0.97
2.00
0.98
0.98
1.00
1.01
0.98
3.05
6.10
1.22
6.69
0.51
2.92
f1 (ppm)

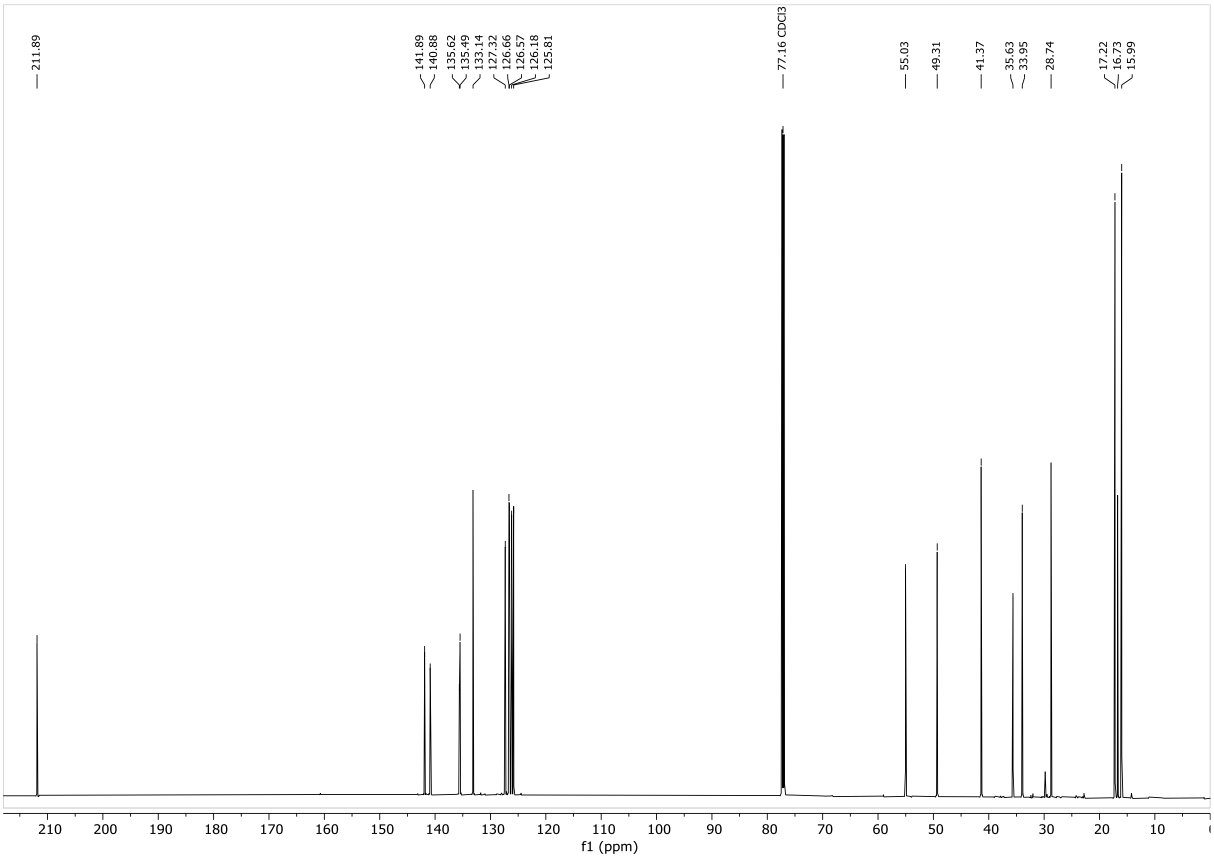
211.89
141.89
140.88
135.62
135.49
133.14
127.32
126.66
126.57
126.18
125.81
77.16 CDCl3
55.03
49.31
41.37
35.63
33.95
28.74
17.22
16.73
15.99
f1 (ppm)

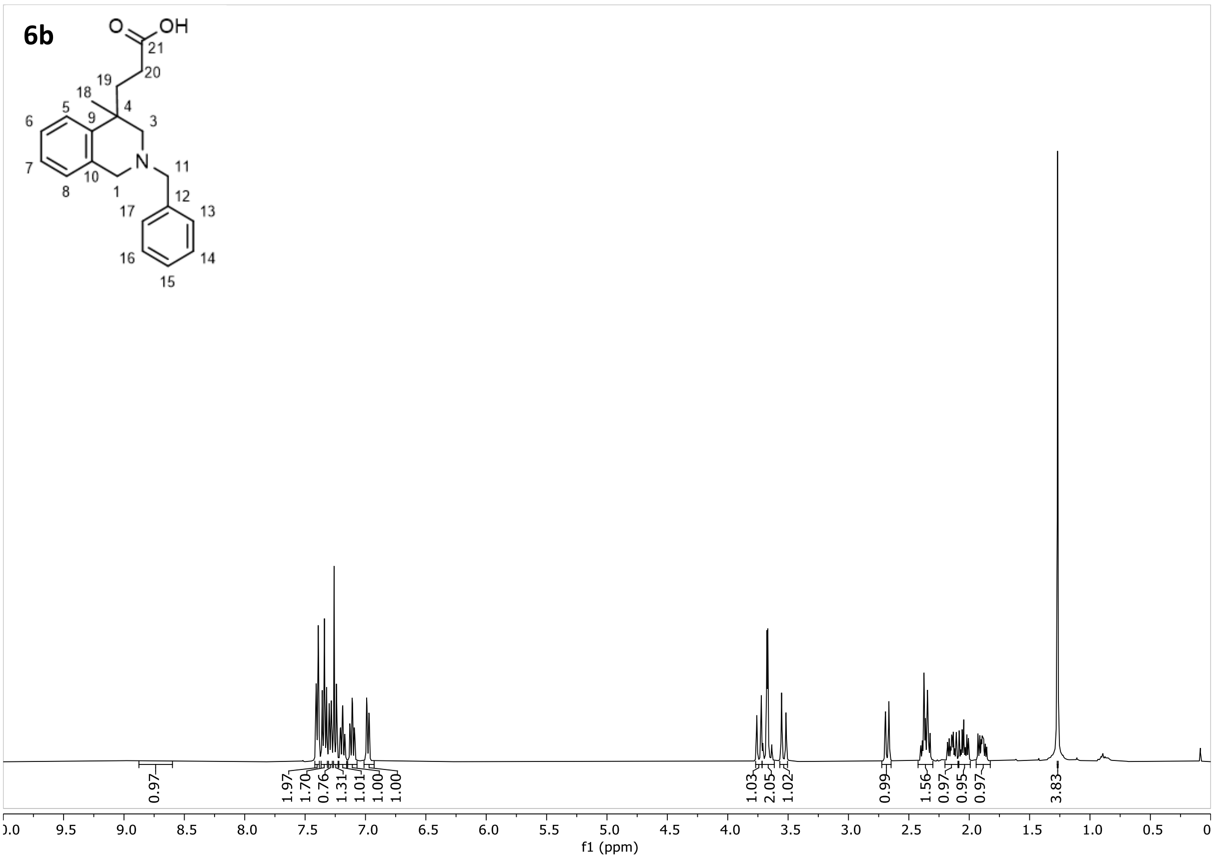
6b
OH
O
N
0.97
1.97
1.70
0.76
1.31
1.01
1.00
1.00
1.03
2.05
1.02
0.99
1.56
0.97
0.95
0.97
3.83
f1 (ppm)

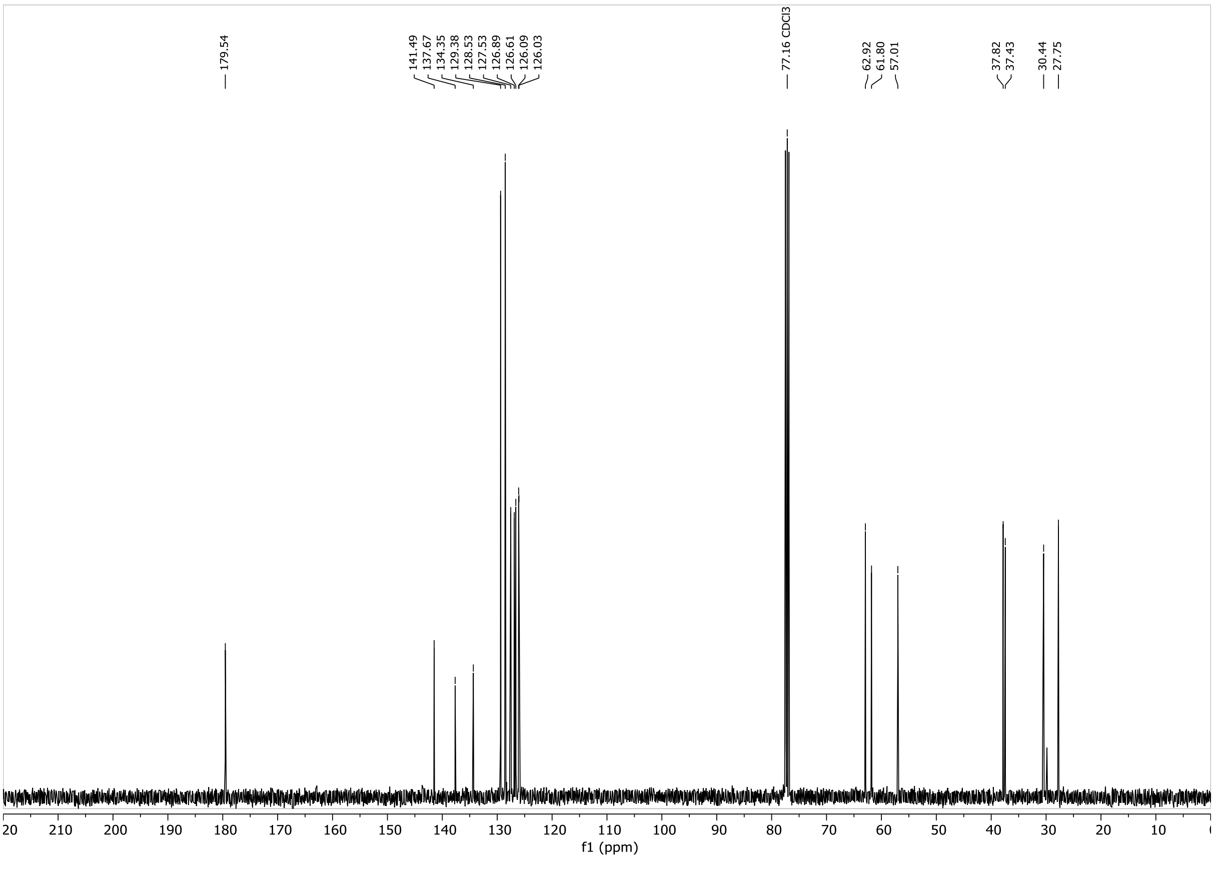
179.54
141.49
137.67
134.35
129.38
128.53
127.53
126.89
126.61
126.09
126.03
77.16 CDCl3
62.92
61.80
57.01
37.82
37.43
30.44
27.75
f1 (ppm)

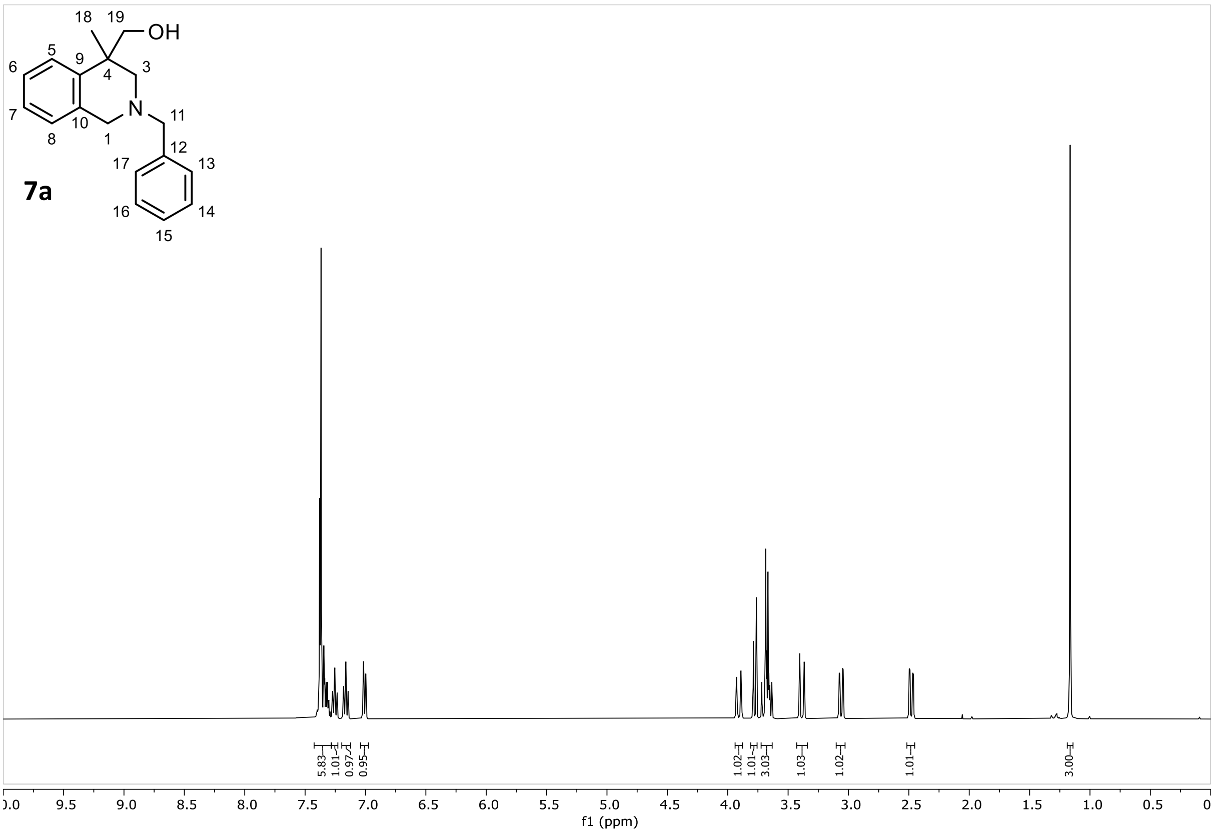
18
19
OH
5
9
6
4
3
7
10
N
11
8
1
17
12
13
7a
16
14
15
5.83
1.01
0.97
0.95
1.02
1.01
3.03
1.03
1.02
1.01
3.00
f1 (ppm)

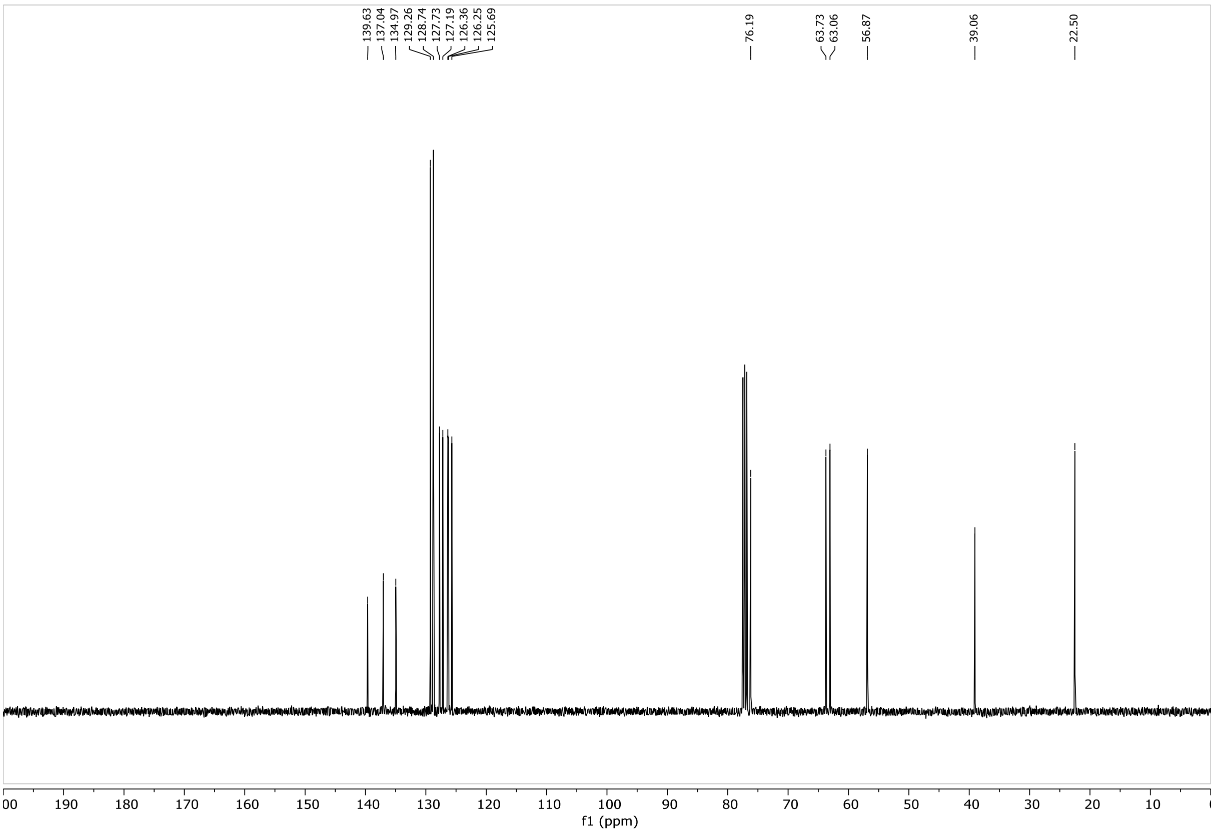
139.63
137.04
134.97
129.26
128.74
127.73
127.19
126.36
126.25
125.69
76.19
63.73
63.06
56.87
39.06
22.50
f1 (ppm)

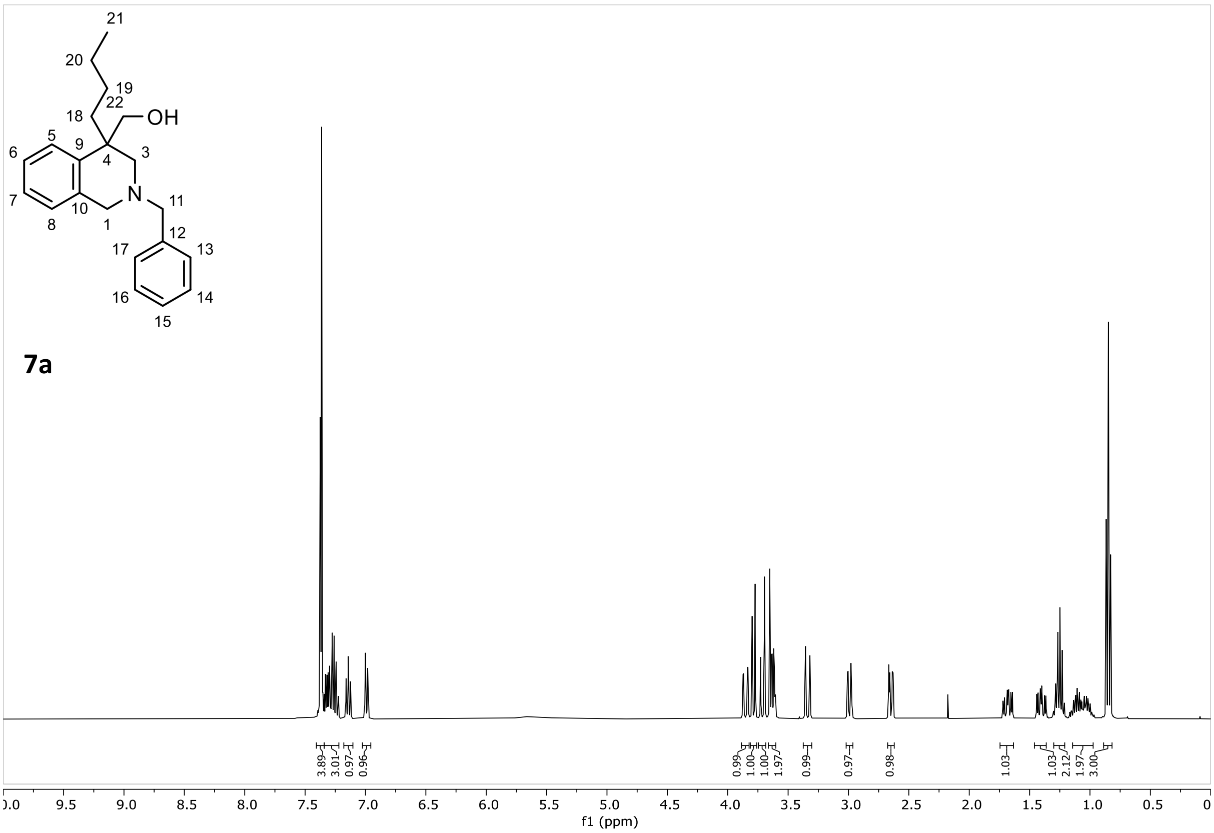
7a
3.89
3.01
0.97
0.96
0.99
1.00
1.00
1.97
0.99
0.97
0.98
1.03
1.03
2.12
1.97
3.00
f1 (ppm)

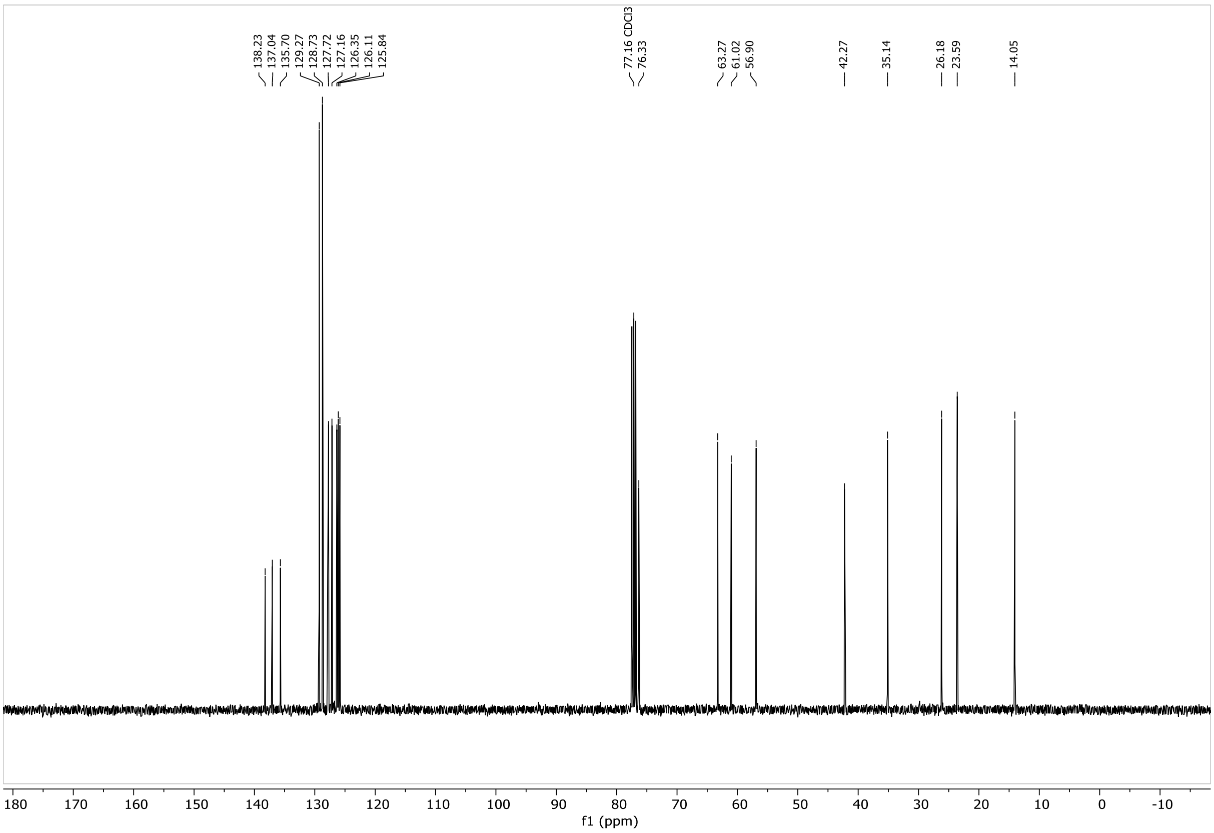
138.23
137.04
135.70
129.27
128.73
127.72
127.16
126.35
126.11
125.84
77.16 CDCl3
76.33
63.27
61.02
56.90
42.27
35.14
26.18
23.59
14.05
f1 (ppm)

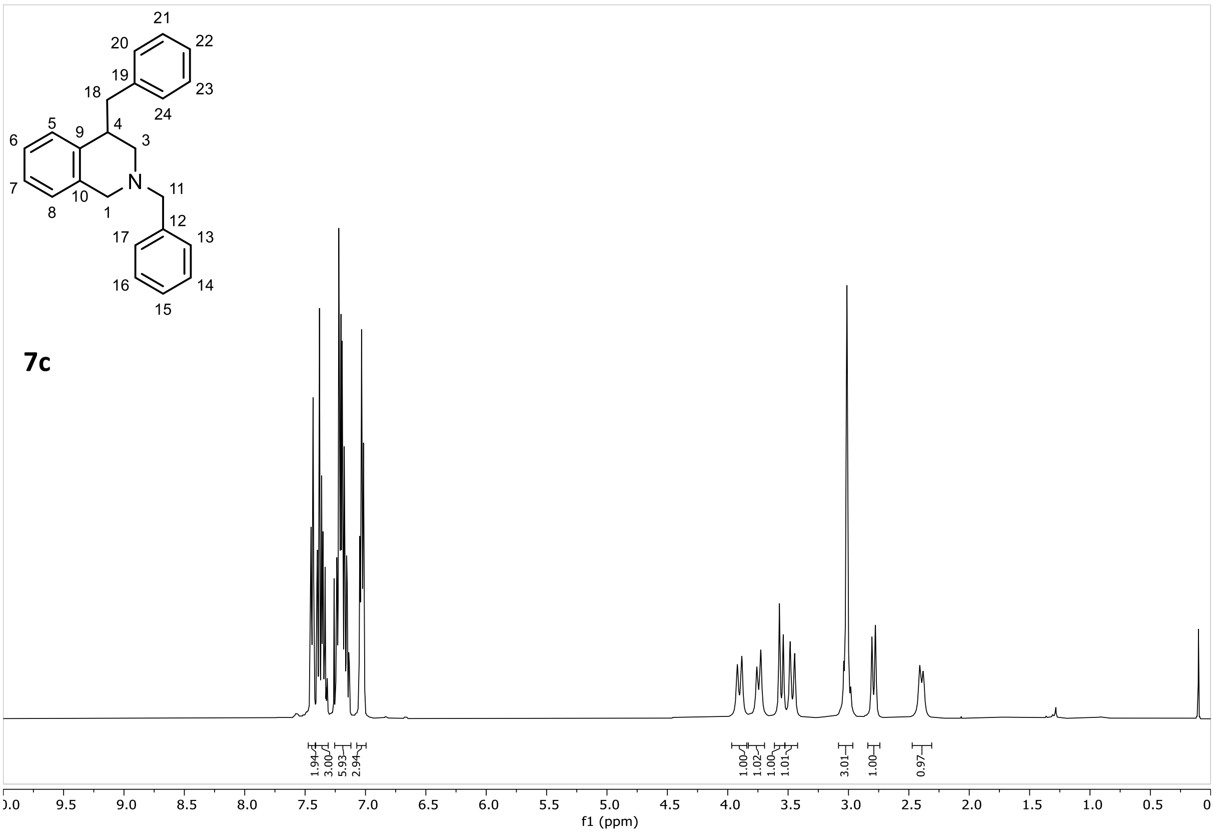
7c
1.94
3.00
5.93
2.94
1.00
1.02
1.00
1.01
3.01
1.00
0.97
f1 (ppm)

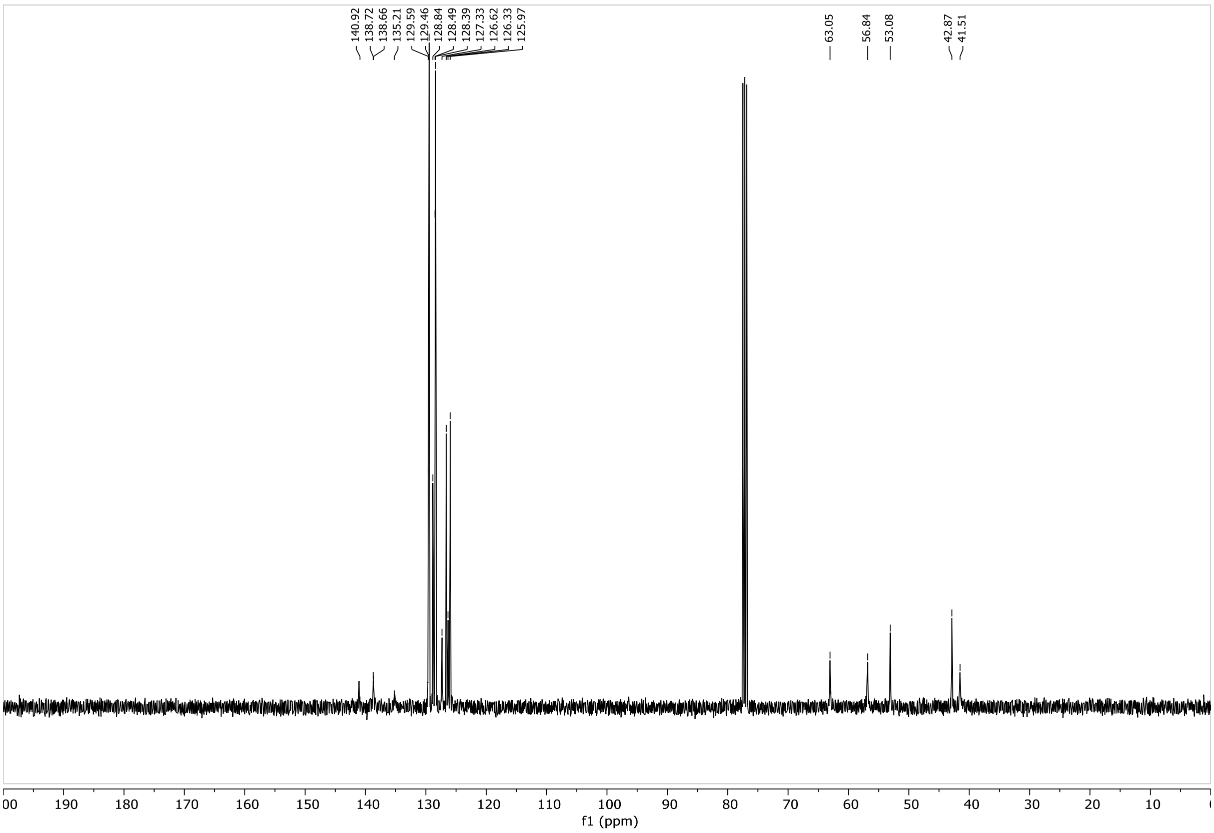
140.92
138.72
138.66
135.21
129.59
129.46
128.84
128.49
128.39
127.33
126.62
126.33
125.97
63.05
56.84
53.08
42.87
41.51
f1 (ppm)

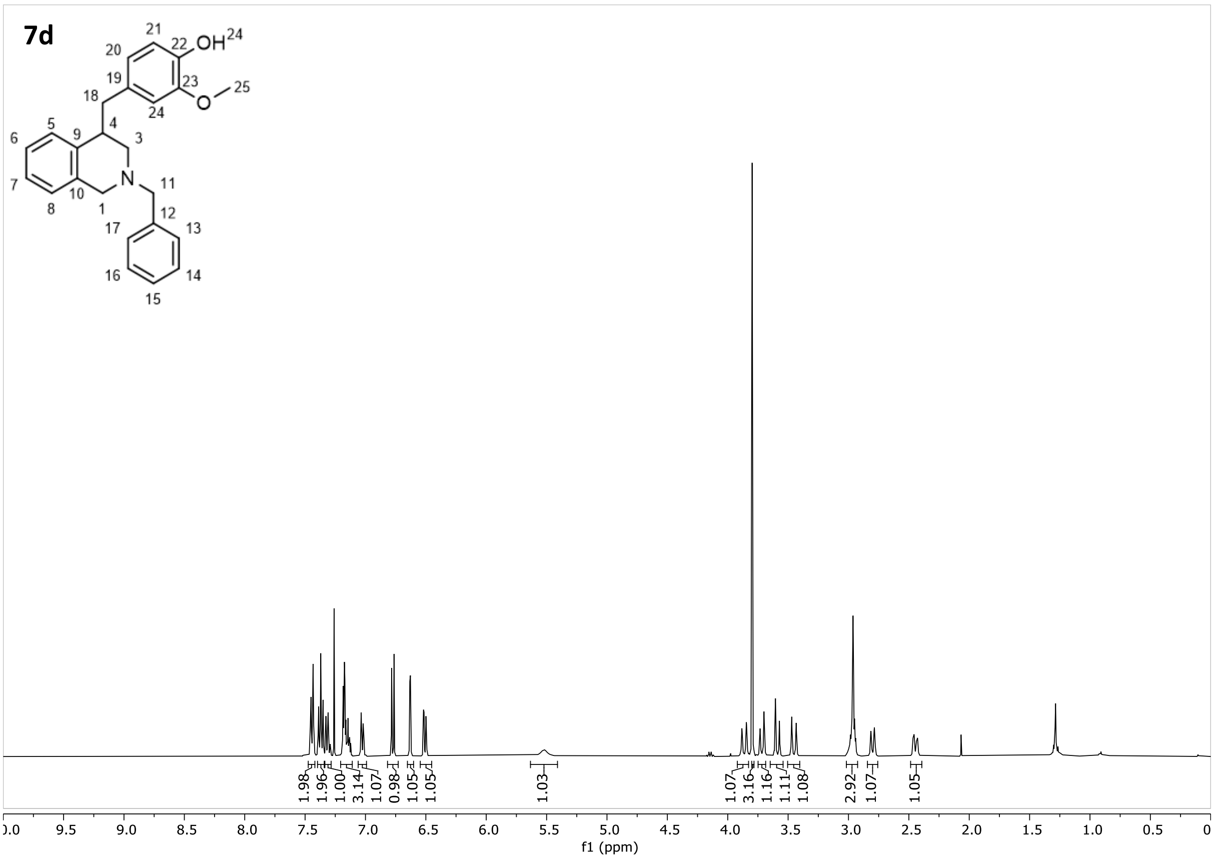
7d
OH
O
N
f1 (ppm)

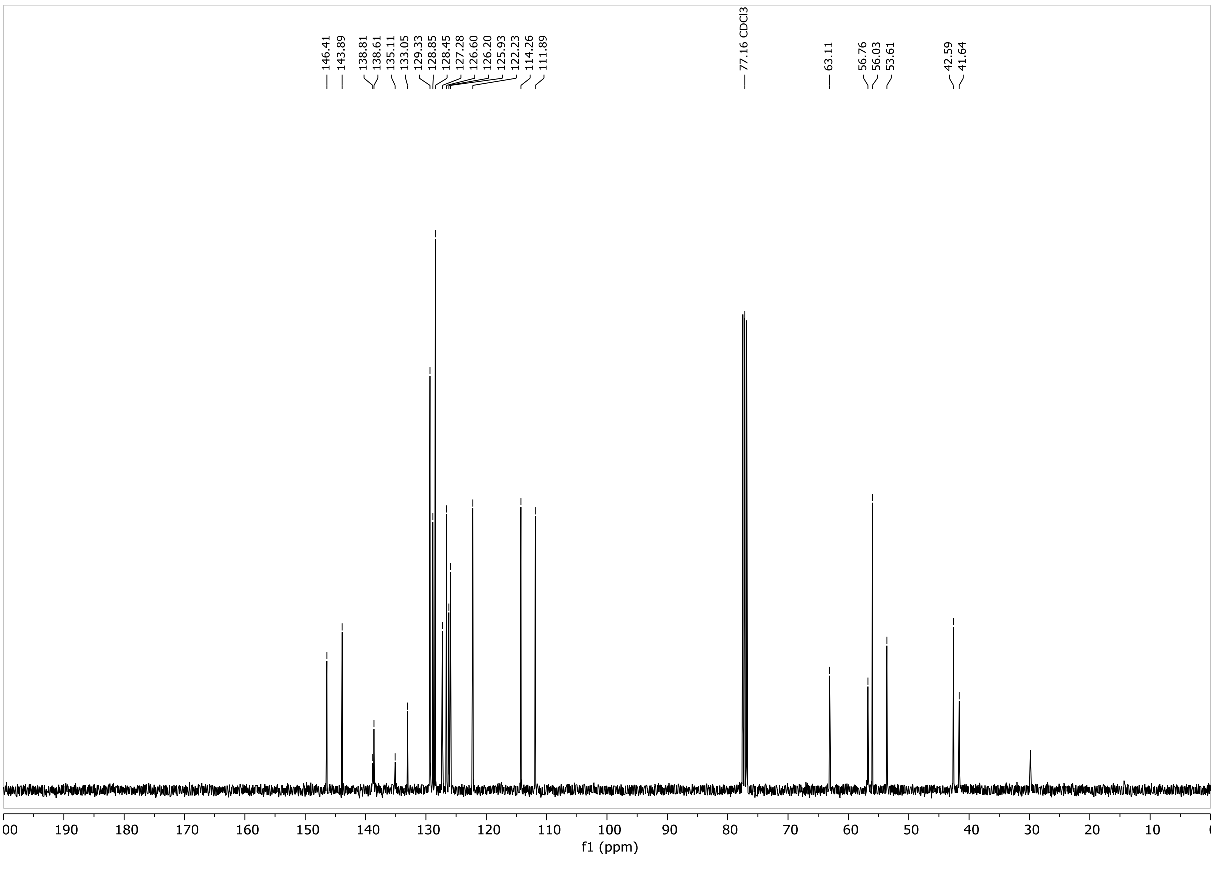
77.16 CDCl3
f1 (ppm)

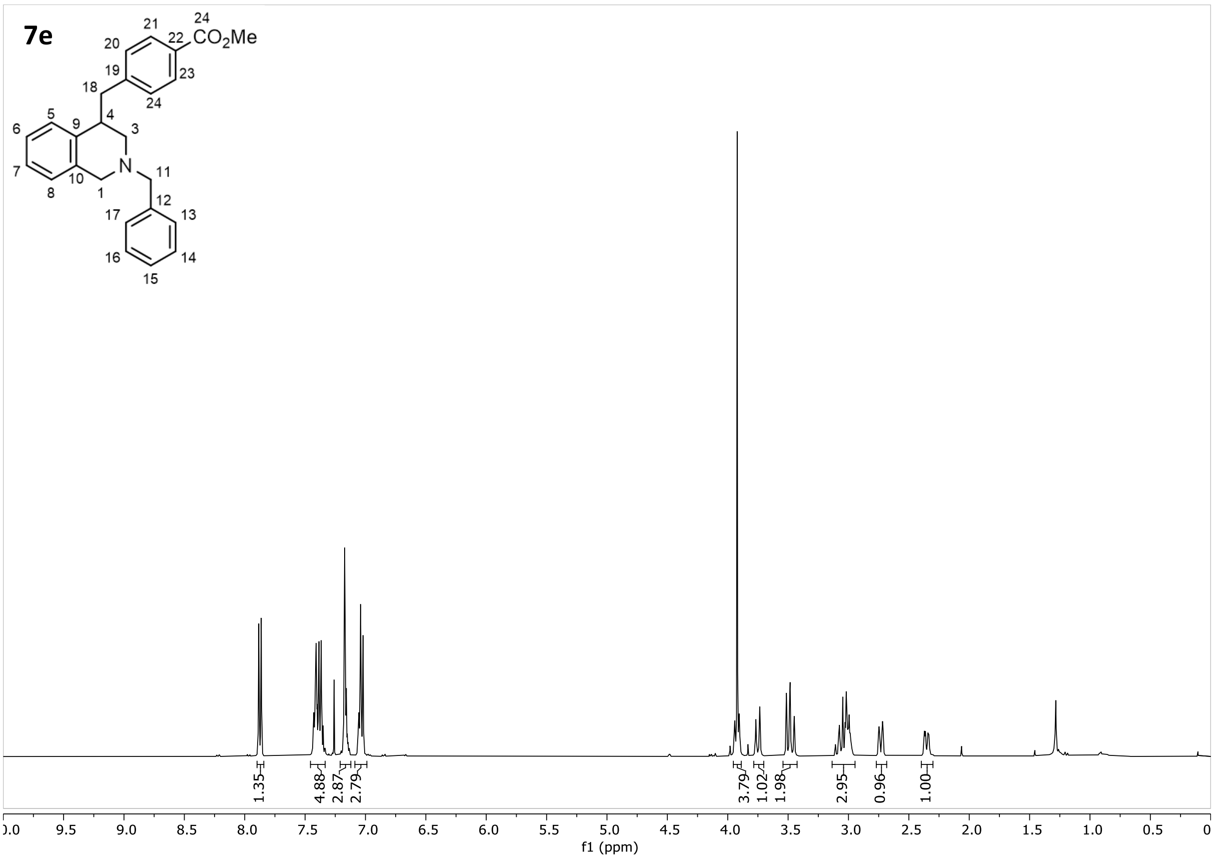
7e
CO2Me
1.35
4.88
2.87
2.79
3.79
1.02
1.98
2.95
0.96
1.00
f1 (ppm)

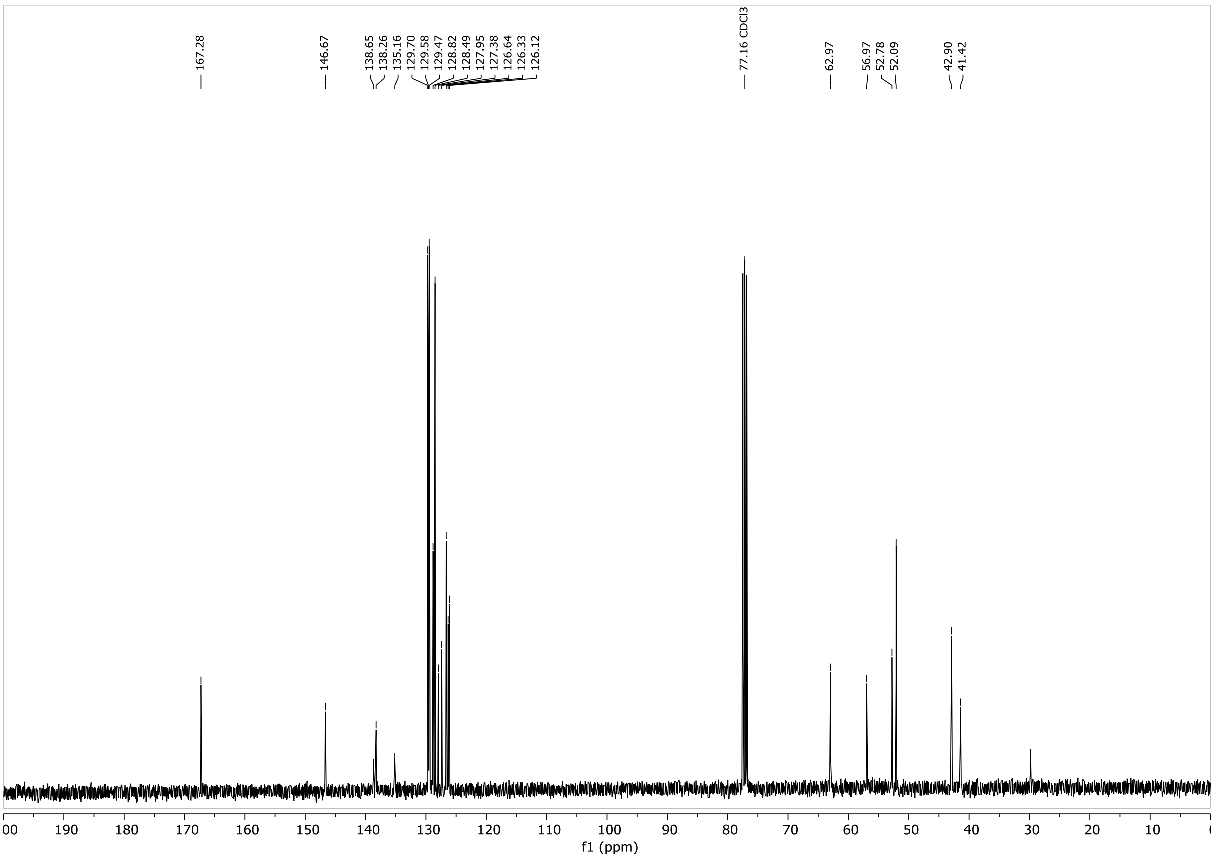
167.28
146.67
138.65
138.26
135.16
129.70
129.58
129.47
128.82
128.49
127.95
127.38
126.64
126.33
126.12
77.16 CDCl3
62.97
56.97
52.78
52.09
42.90
41.42
f1 (ppm)

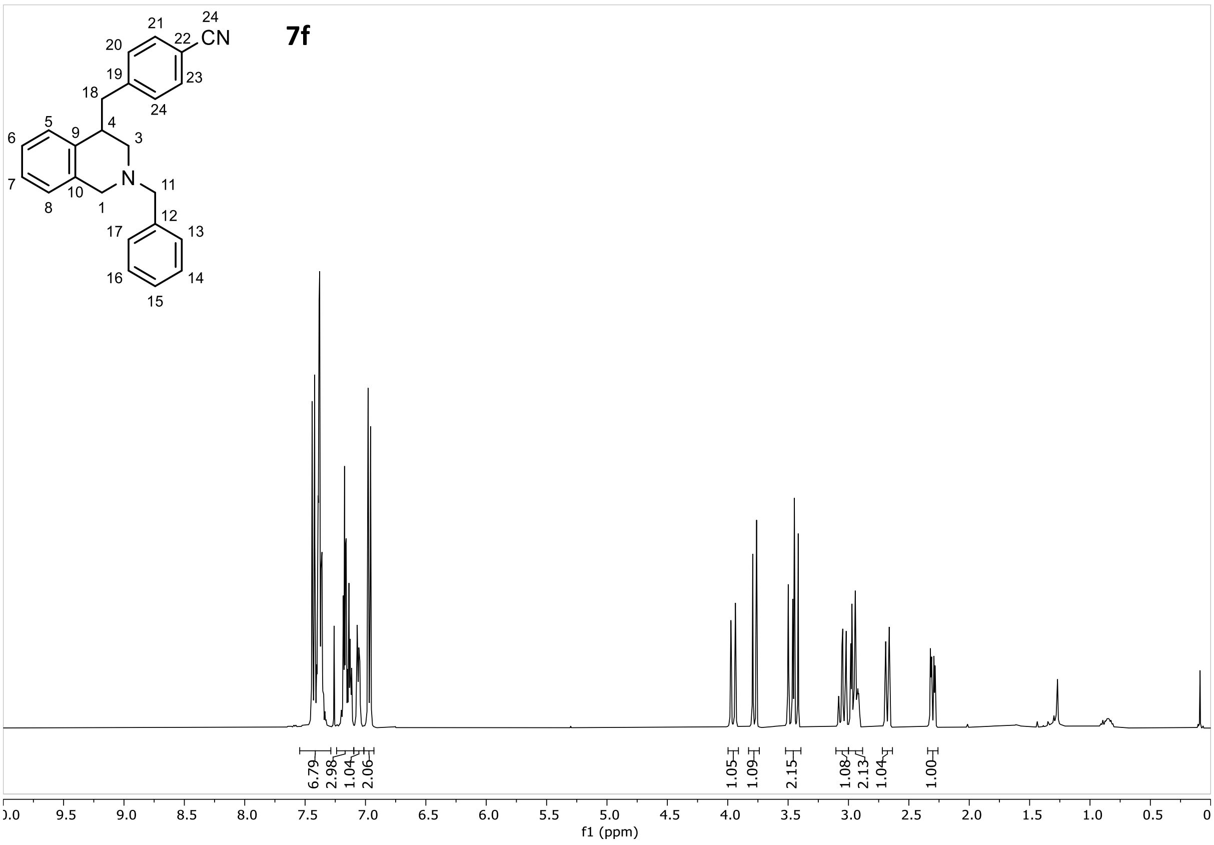
7f
CN
6.79
2.98
1.04
2.06
1.05
1.09
2.15
1.08
2.13
1.04
1.00
f1 (ppm)

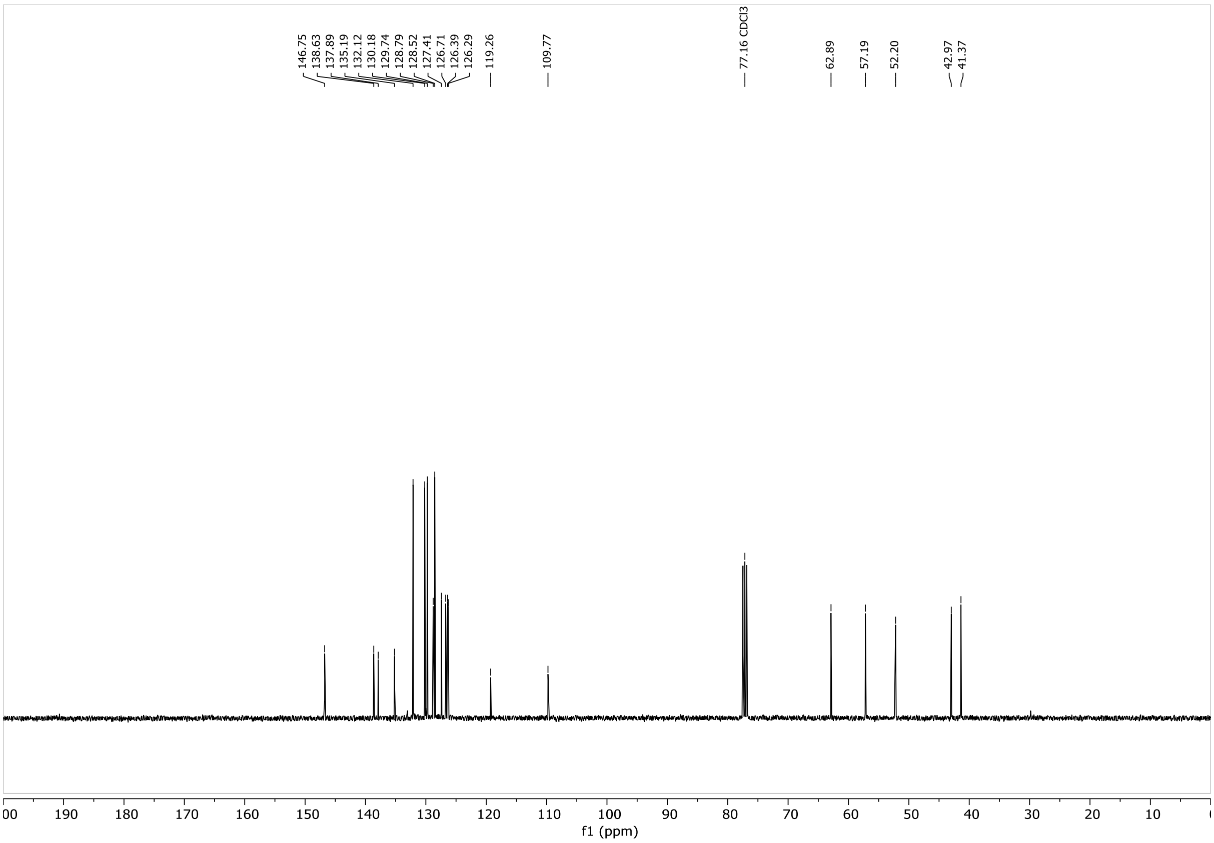
146.75
138.63
137.89
135.19
132.12
130.18
129.74
128.79
128.52
127.41
126.71
126.39
126.29
119.26
109.77
77.16 CDCl3
62.89
57.19
52.20
42.97
41.37
f1 (ppm)

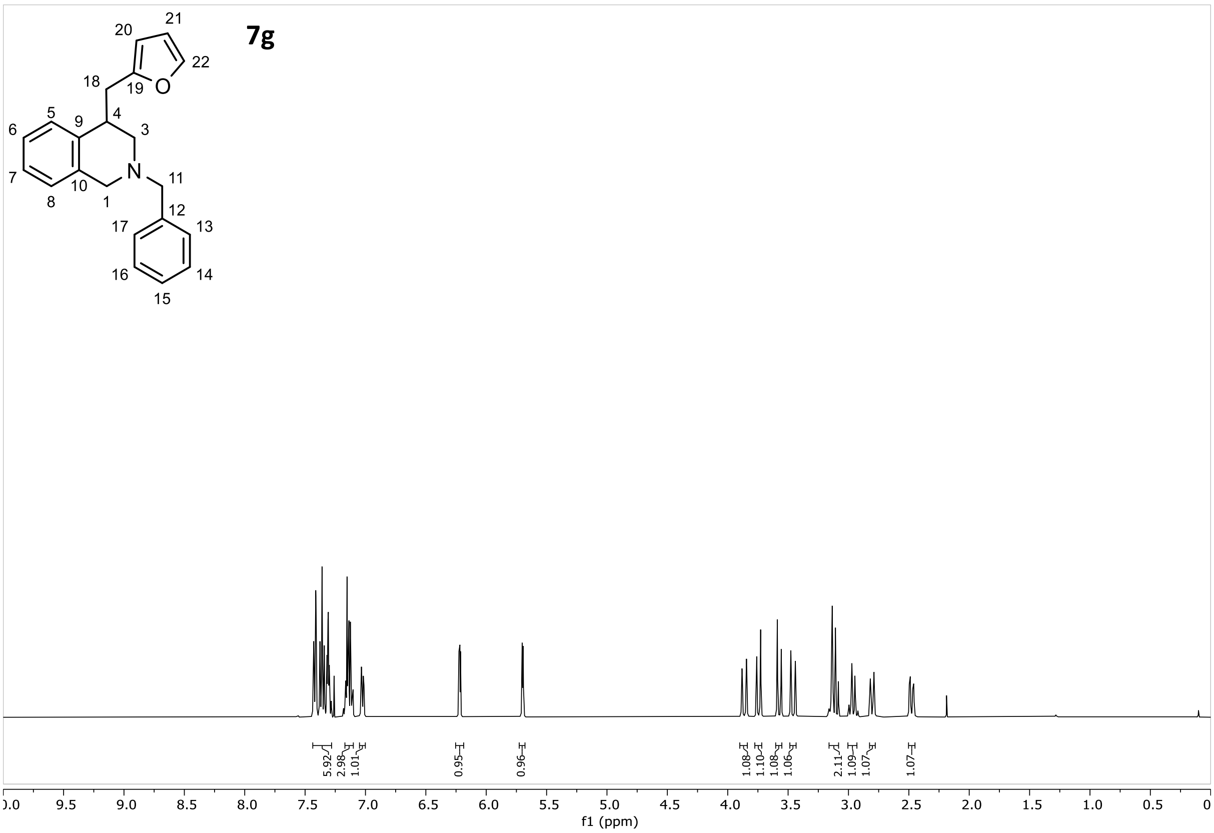
7g
20
21
22
18
19
O
5
9
4
3
6
7
10
N
11
8
1
12
17
13
16
14
15
5.92
2.98
1.01
0.95
0.96
1.08
1.10
1.08
1.06
2.11
1.09
1.07
1.07
f1 (ppm)

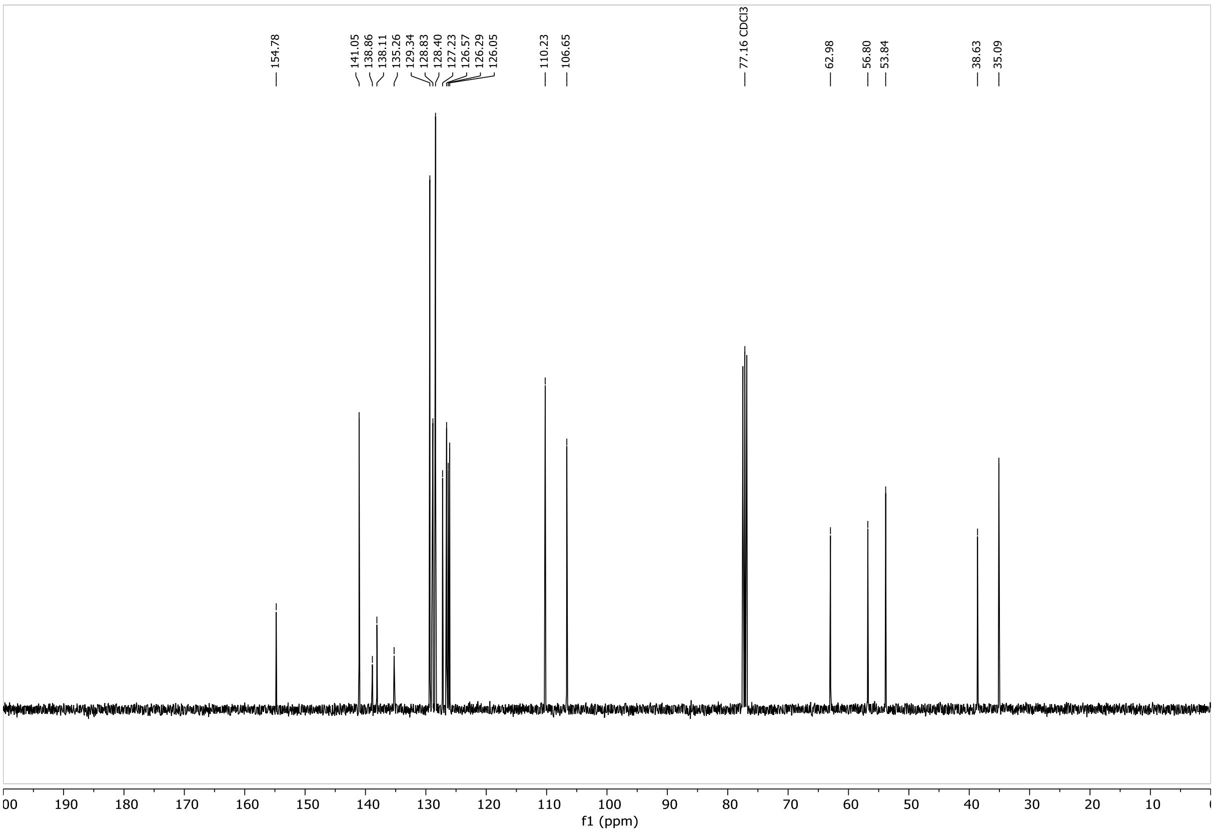
154.78
141.05
138.86
138.11
135.26
129.34
128.83
128.40
127.23
126.57
126.29
126.05
110.23
106.65
77.16 CDCl3
62.98
56.80
53.84
38.63
35.09
f1 (ppm)

7h

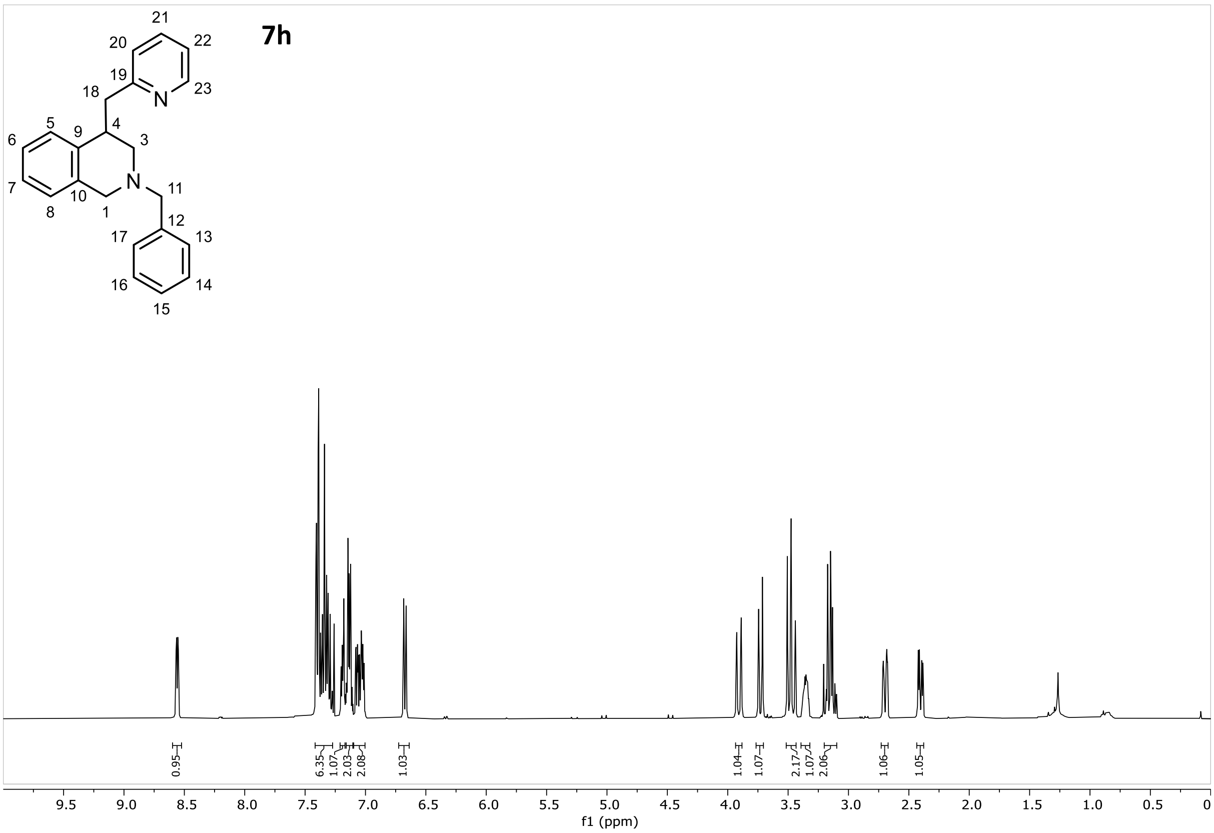

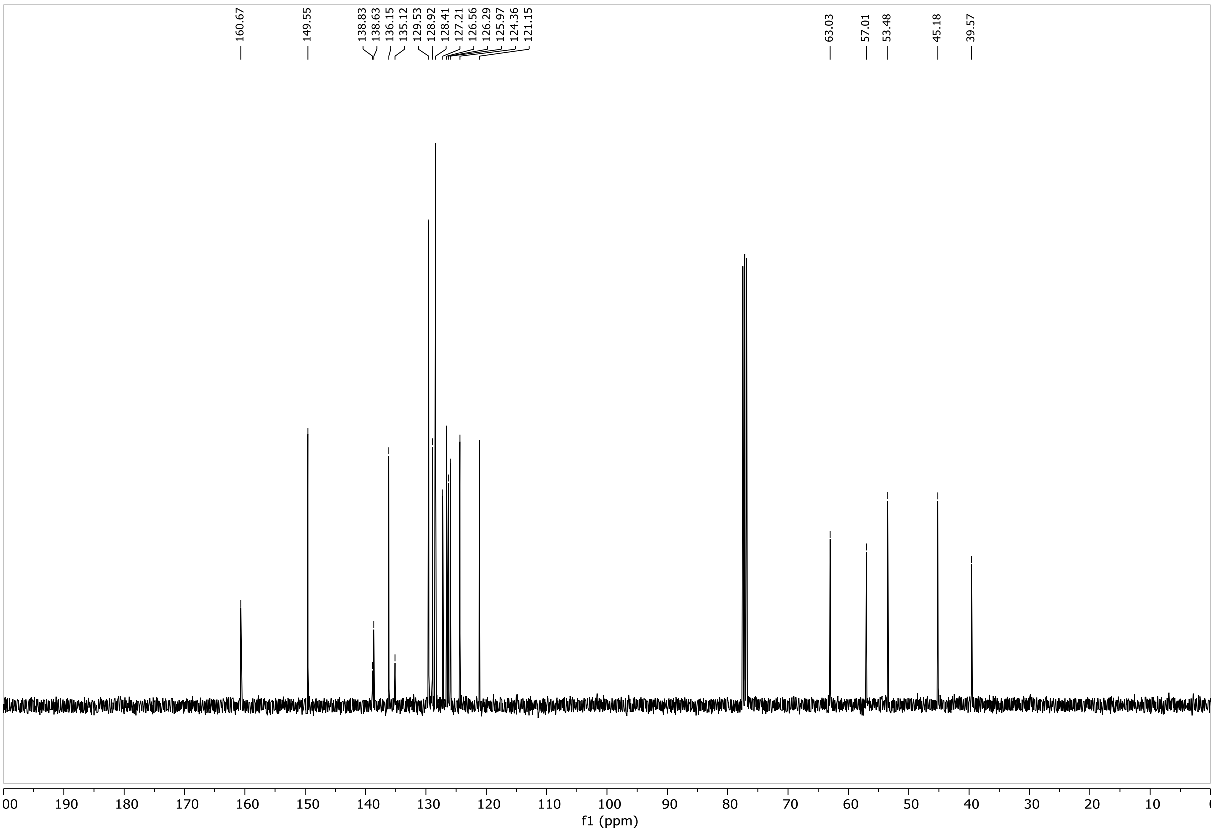

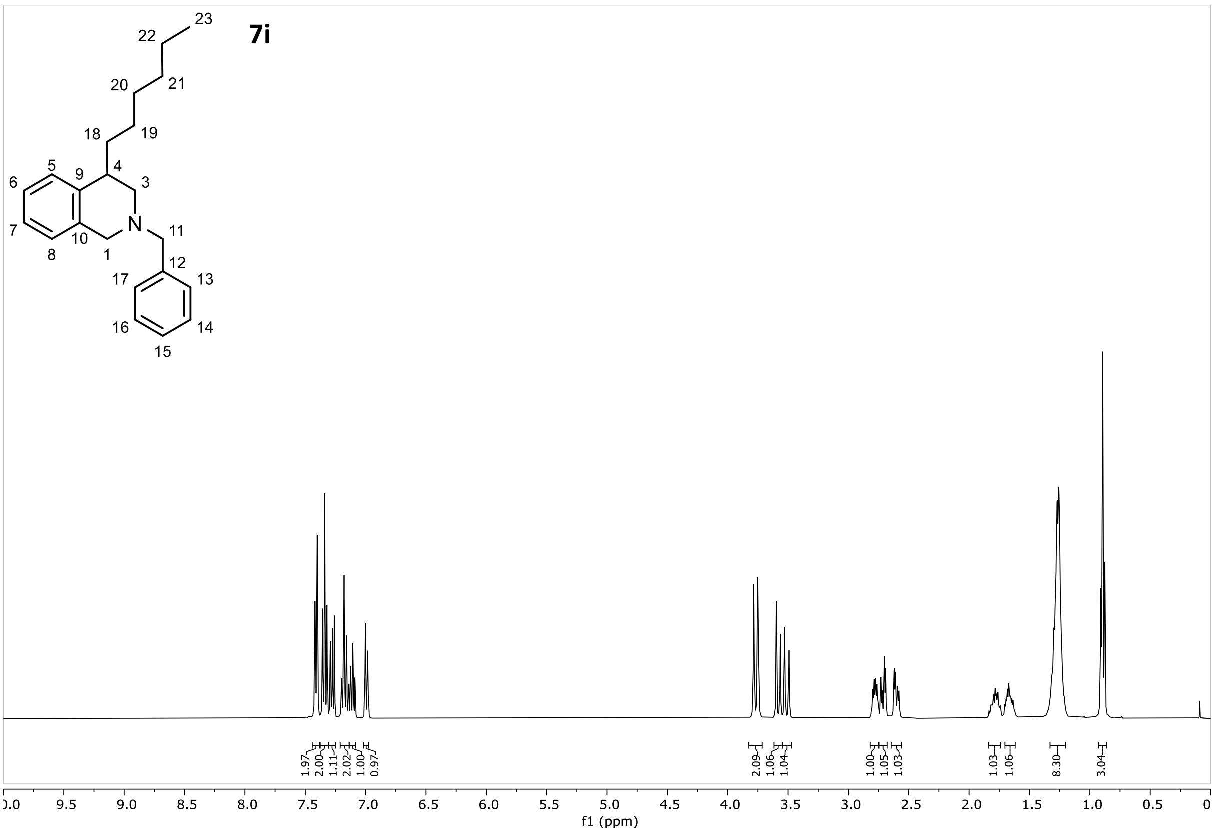
7i
1.97
2.00
1.11
2.02
1.00
0.97
2.09
1.06
1.04
1.00
1.05
1.03
1.03
1.06
8.30
3.04
f1 (ppm)

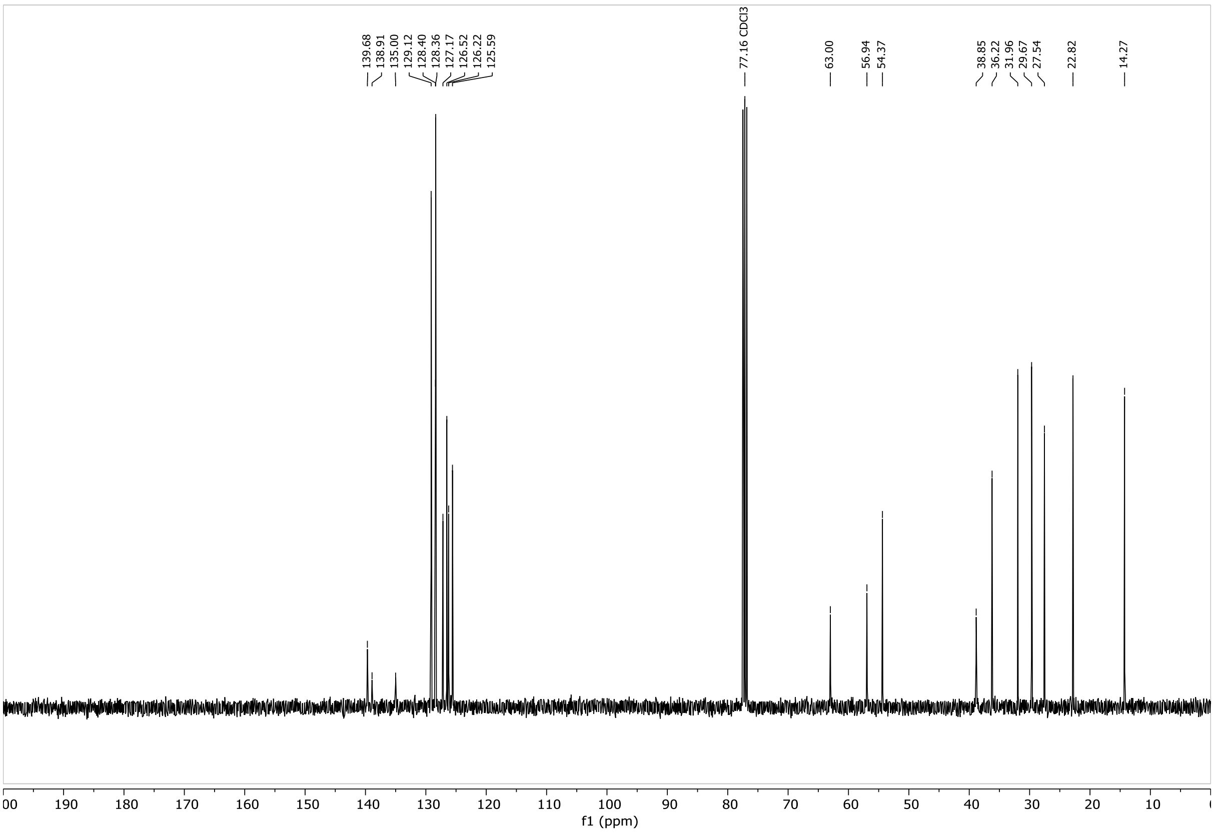
139.68
138.91
135.00
129.12
128.40
128.36
127.17
126.52
126.22
125.59
77.16 CDCl3
63.00
56.94
54.37
38.85
36.22
31.96
29.67
27.54
22.82
14.27
f1 (ppm)

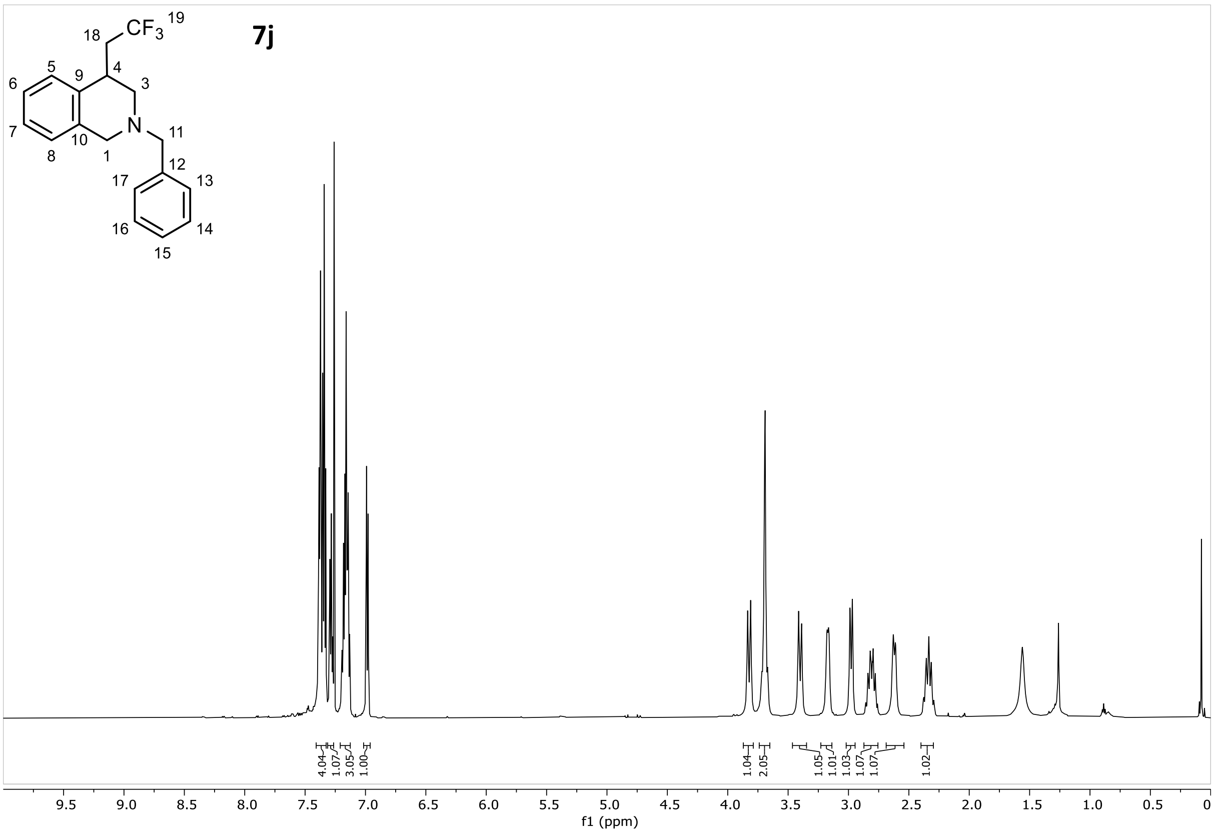
7j
CF3
4.04
1.07
3.05
1.00
1.04
2.05
1.05
1.01
1.03
1.07
1.07
1.02
f1 (ppm)

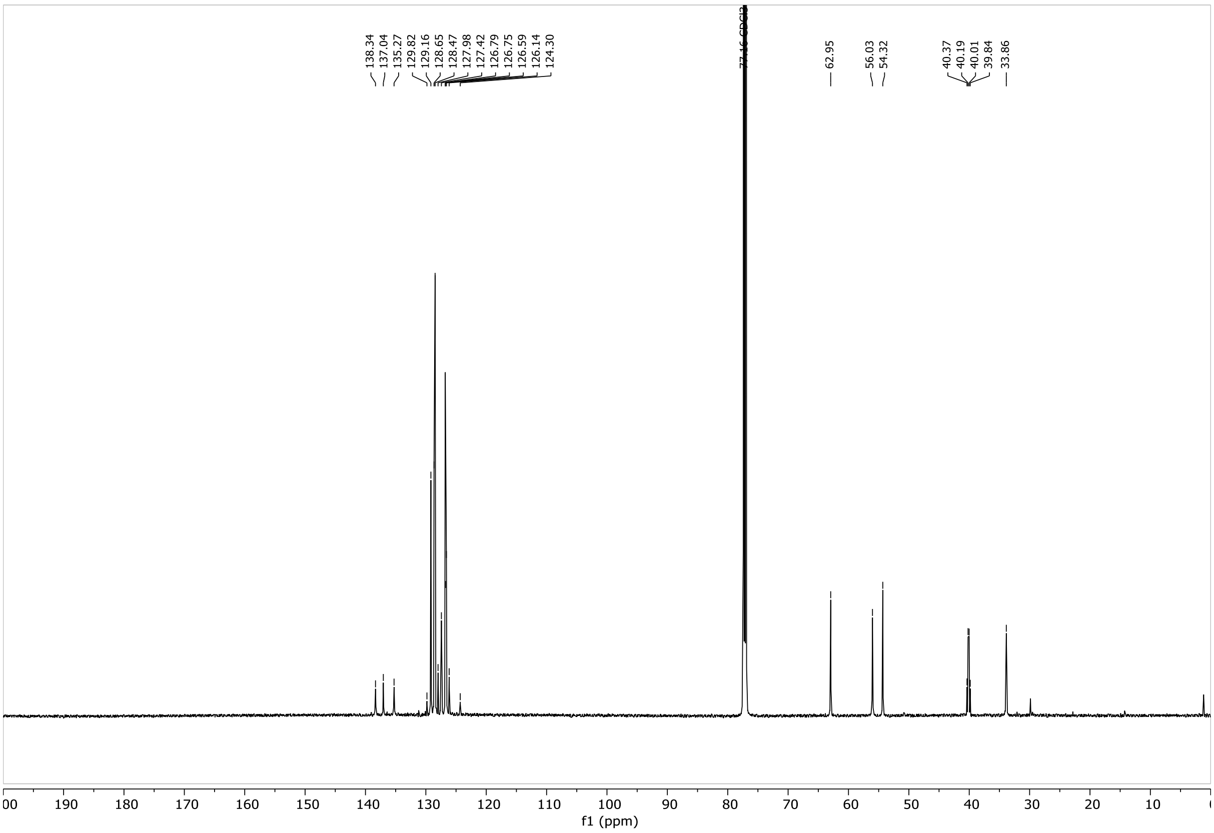
138.34
137.04
135.27
129.82
129.16
128.65
128.47
127.98
127.42
126.79
126.75
126.59
126.14
124.30
77.16 CDCl3
62.95
56.03
54.32
40.37
40.19
40.01
39.84
33.86
f1 (ppm)

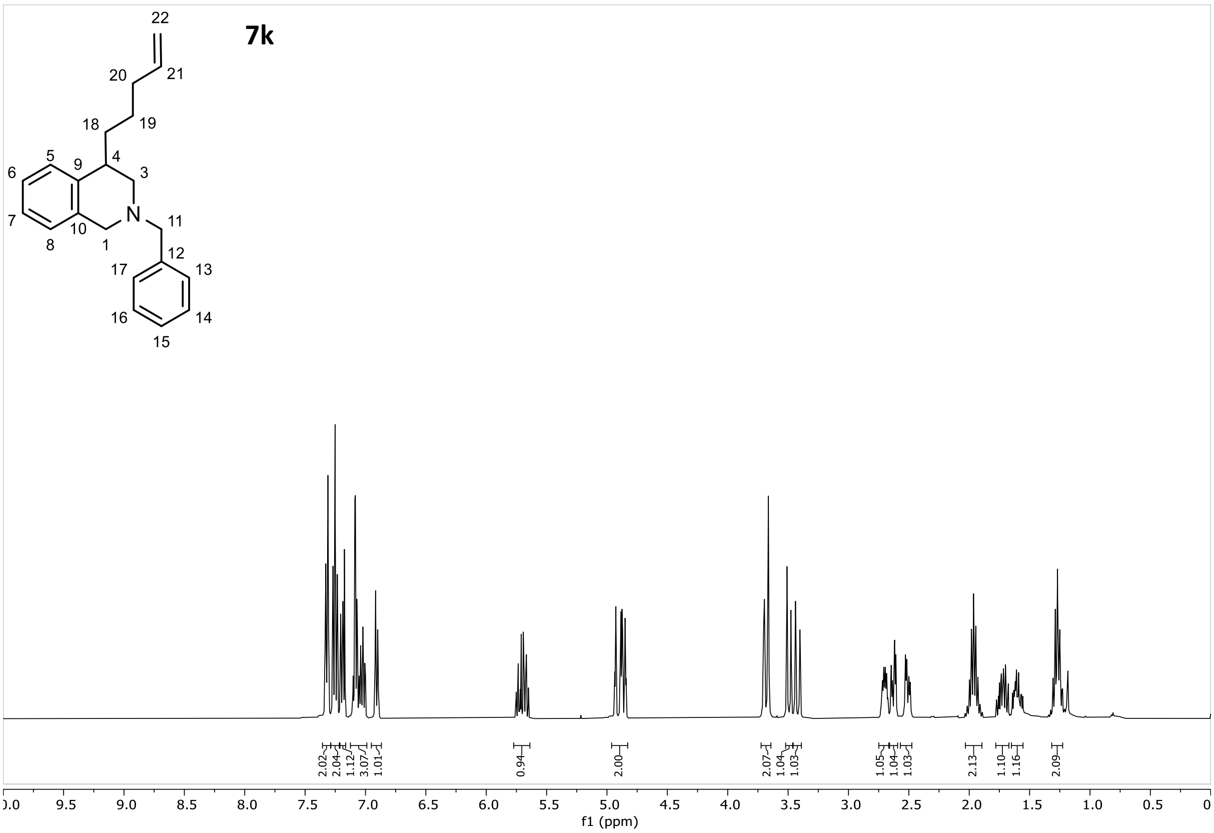
7k
22
21
20
19
18
4
3
5
9
6
7
10
N
11
8
1
12
17
13
16
14
15
2.02
2.04
1.12
3.07
1.01
0.94
2.00
2.07
1.04
1.03
1.05
1.04
1.03
2.13
1.10
1.16
2.09
f1 (ppm)

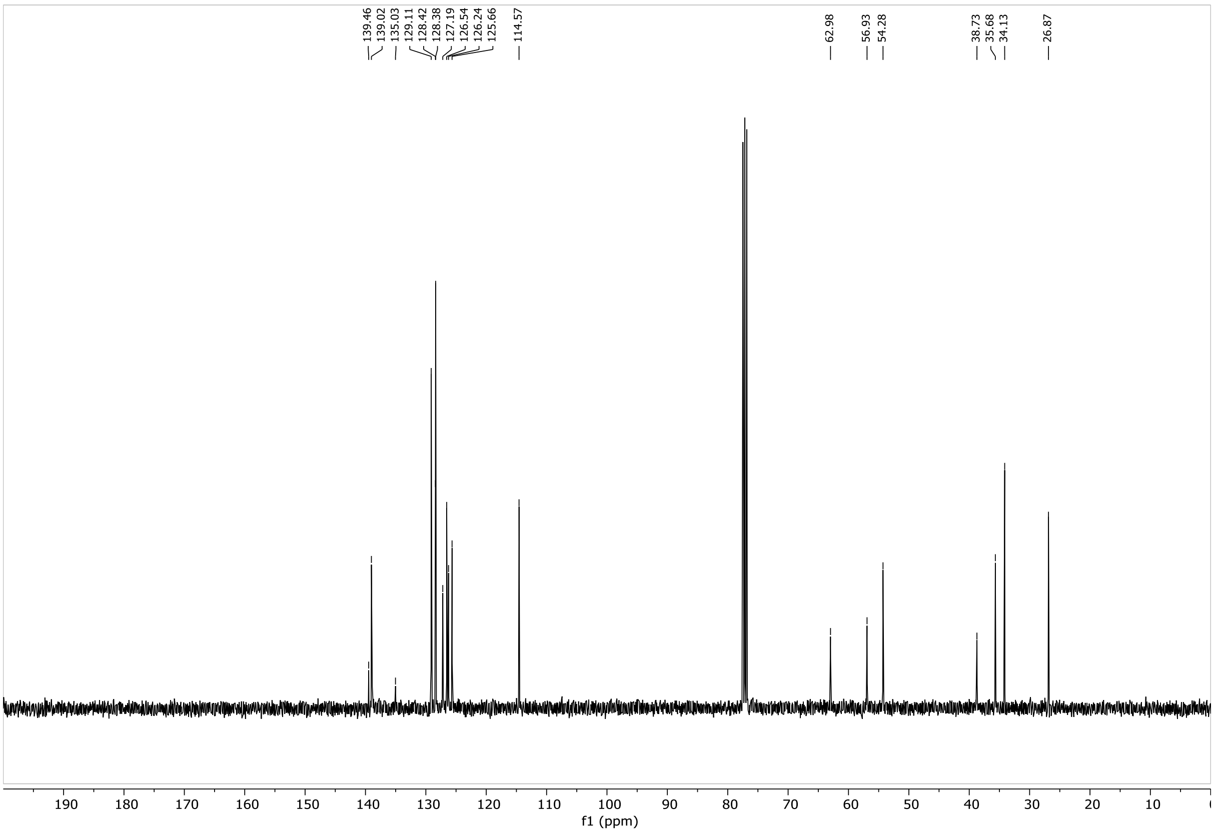
139.46
139.02
135.03
129.11
128.42
128.38
127.19
126.54
126.24
125.66
114.57
62.98
56.93
54.28
38.73
35.68
34.13
26.87
f1 (ppm)

7l

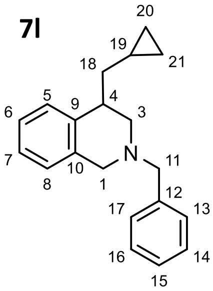
20
19
21
18
4
3
5
9
6
N
11
7
10
1
8
12
17
13
16
14
15

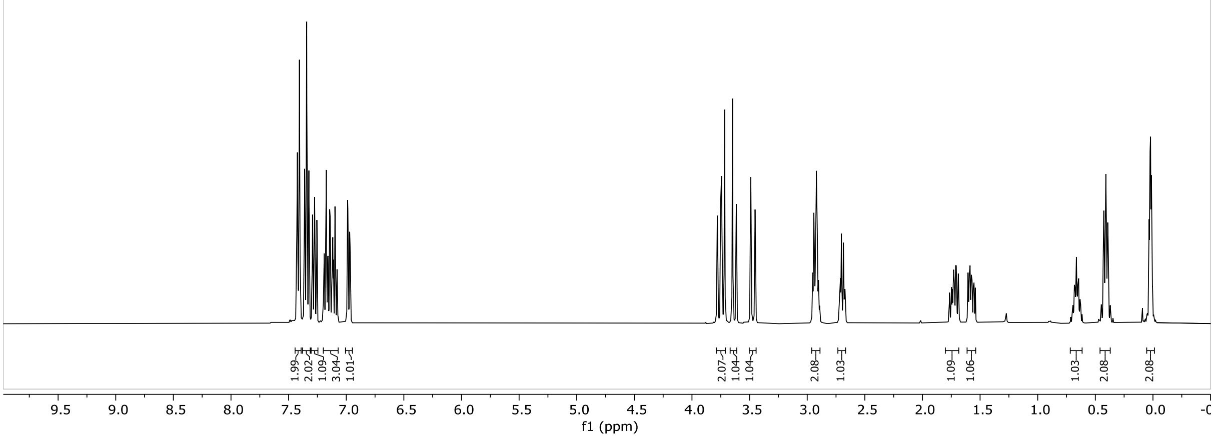
1.99
2.02
1.09
3.04
1.01
2.07
1.04
1.04
2.08
1.03
1.09
1.06
1.03
2.08
2.08
f1 (ppm)

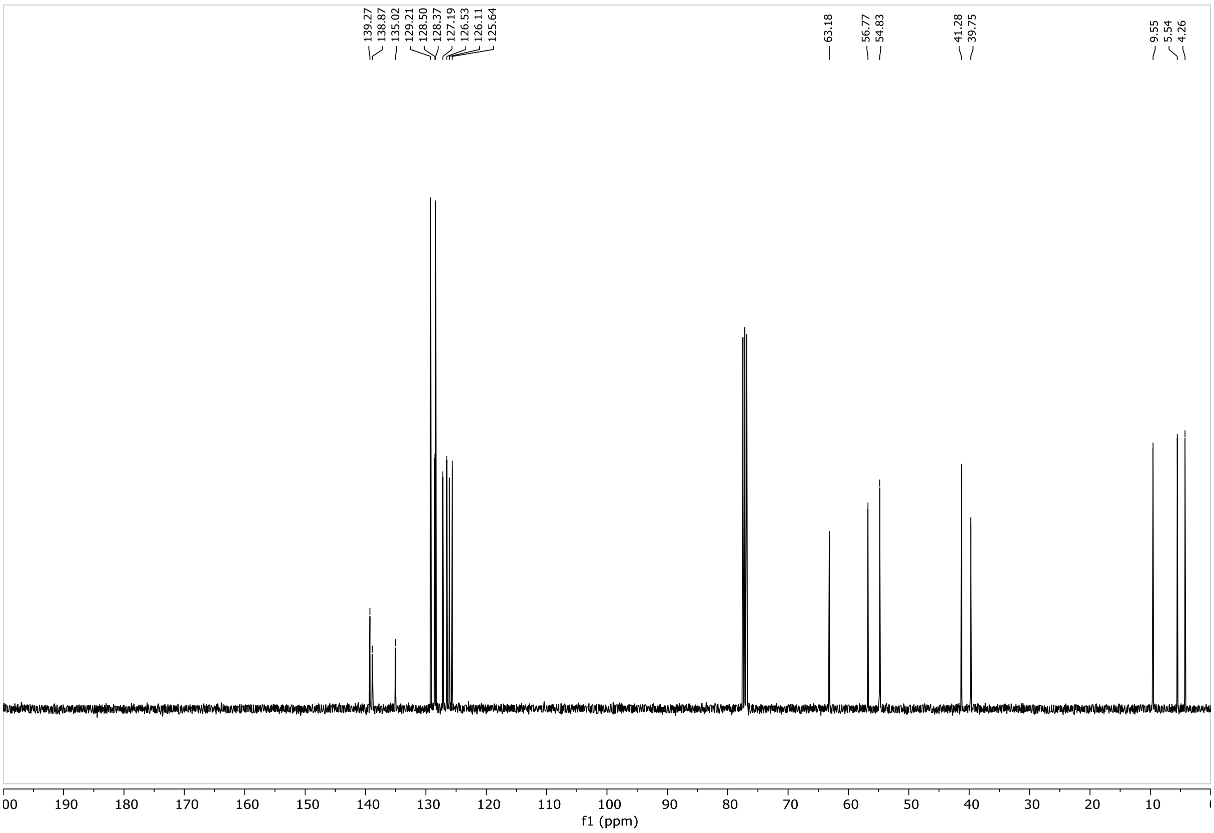
139.27
138.87
135.02
129.21
128.50
128.37
127.19
126.53
126.11
125.64
63.18
56.77
54.83
41.28
39.75
9.55
5.54
4.26
f1 (ppm)

7m

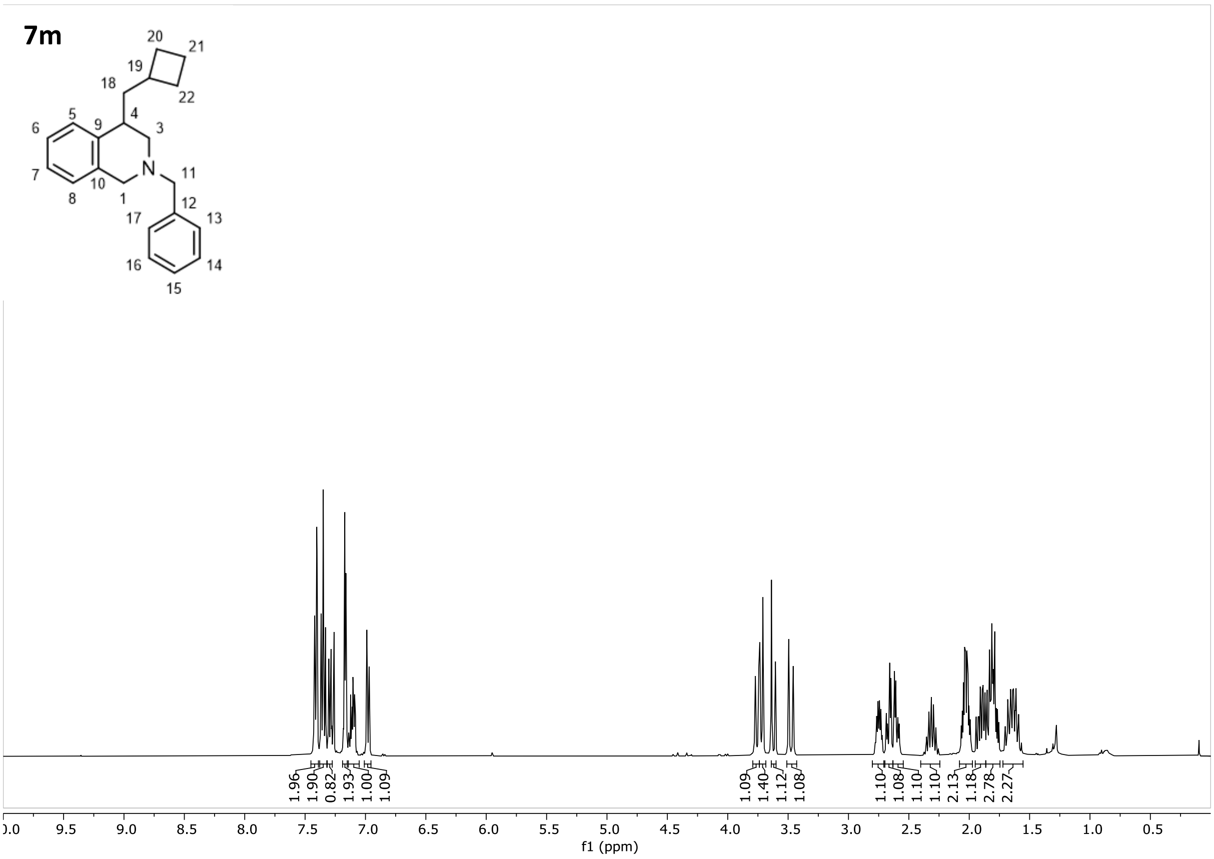

1.96
1.90
0.82
1.93
1.00
1.09
1.09
1.40
1.12
1.08
1.10
1.08
1.10
1.10
2.13
1.18
2.78
2.27
f1 (ppm)

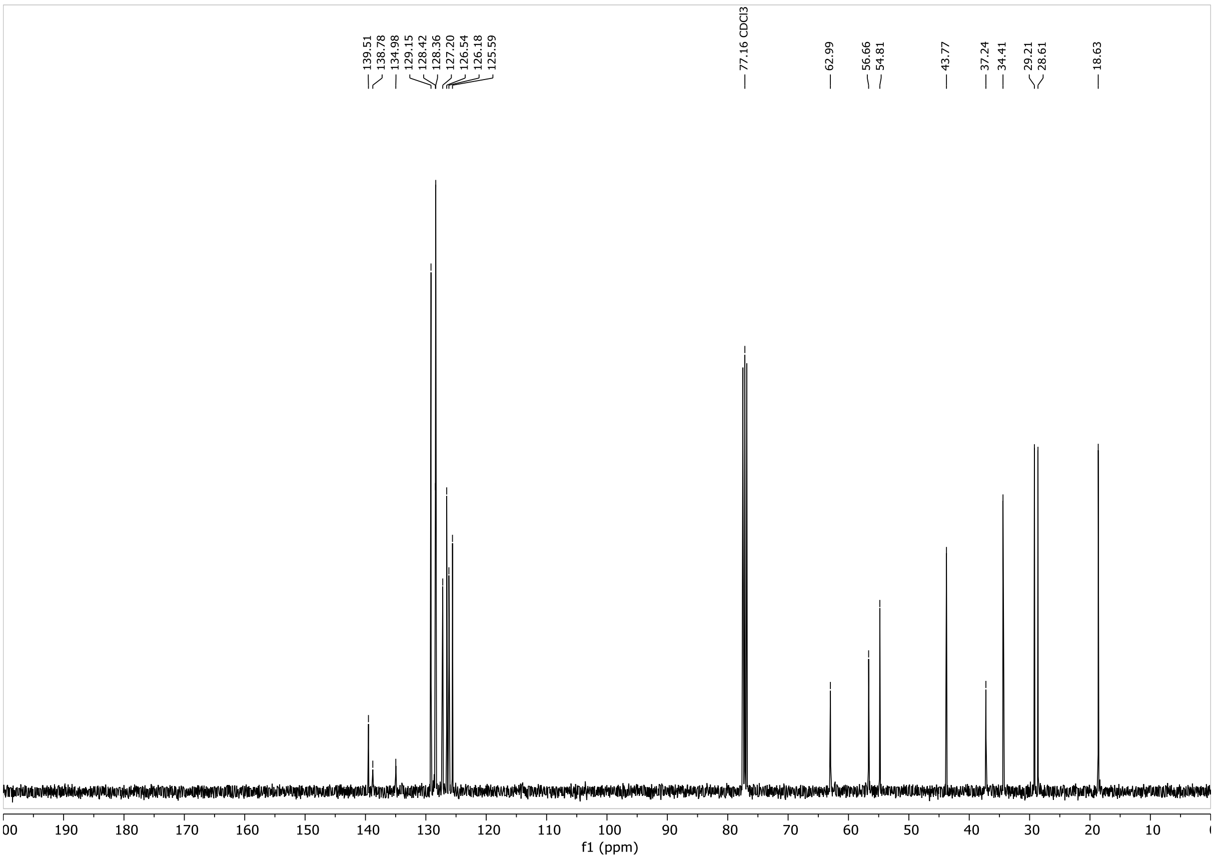

139.51
138.78
134.98
129.15
128.42
128.36
127.20
126.54
126.18
125.59
77.16 CDCl3
62.99
56.66
54.81
43.77
37.24
34.41
29.21
28.61
18.63
f1 (ppm)

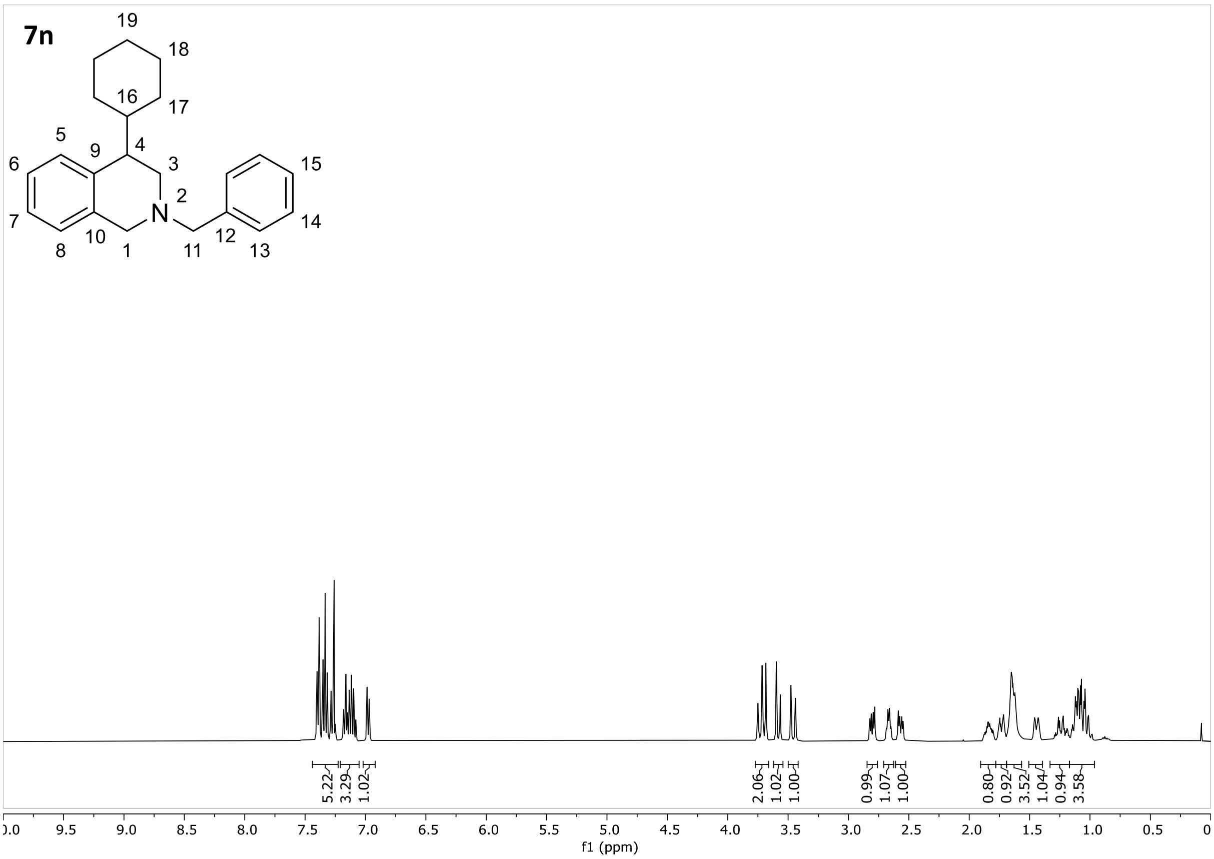
7n
19
18
16
17
5
9
4
3
6
2
N
15
7
10
12
14
8
1
11
13
5.22
3.29
1.02
2.06
1.02
1.00
0.99
1.07
1.00
0.80
0.92
3.52
1.04
0.94
3.58
f1 (ppm)

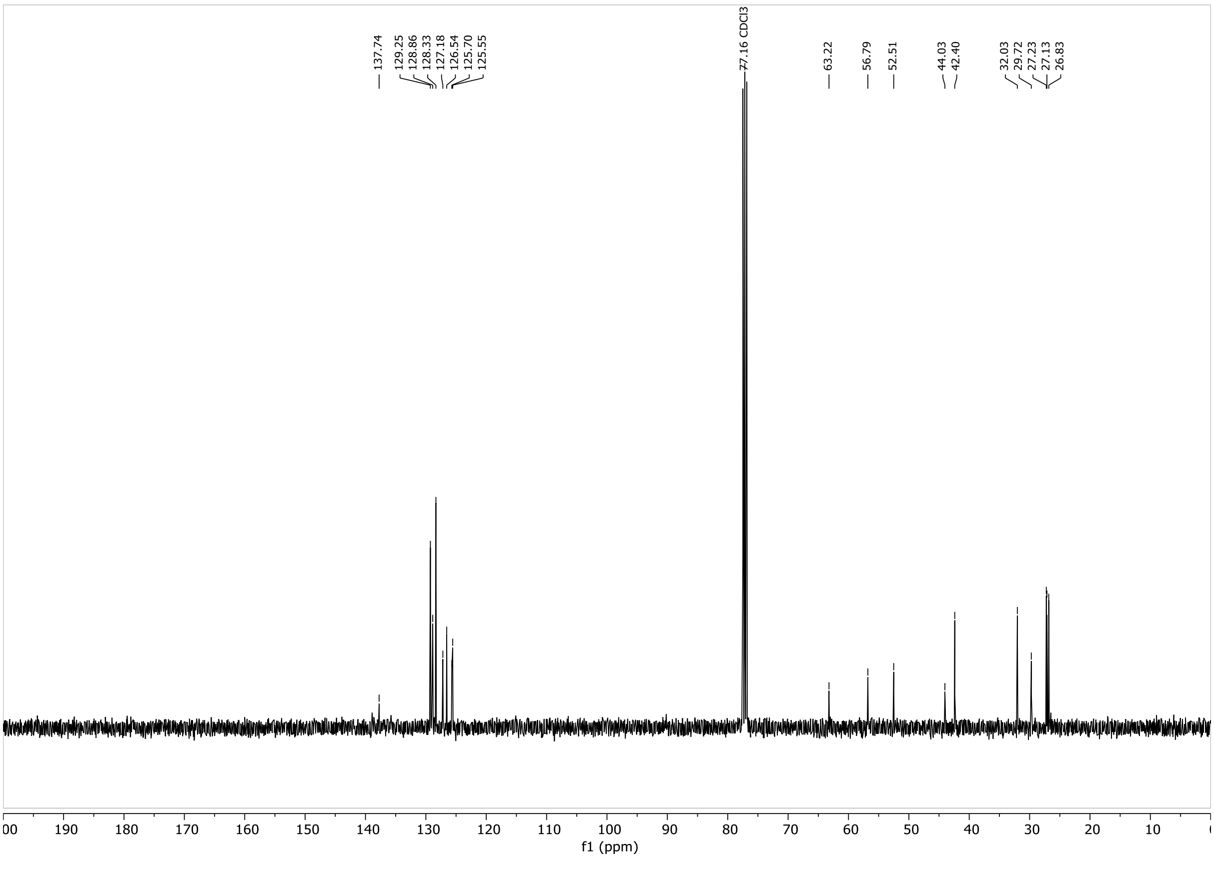
137.74
129.25
128.86
128.33
127.18
126.54
125.70
125.55
77.16 CDCl3
63.22
56.79
52.51
44.03
42.40
32.03
29.72
27.23
27.13
26.83
f1 (ppm)

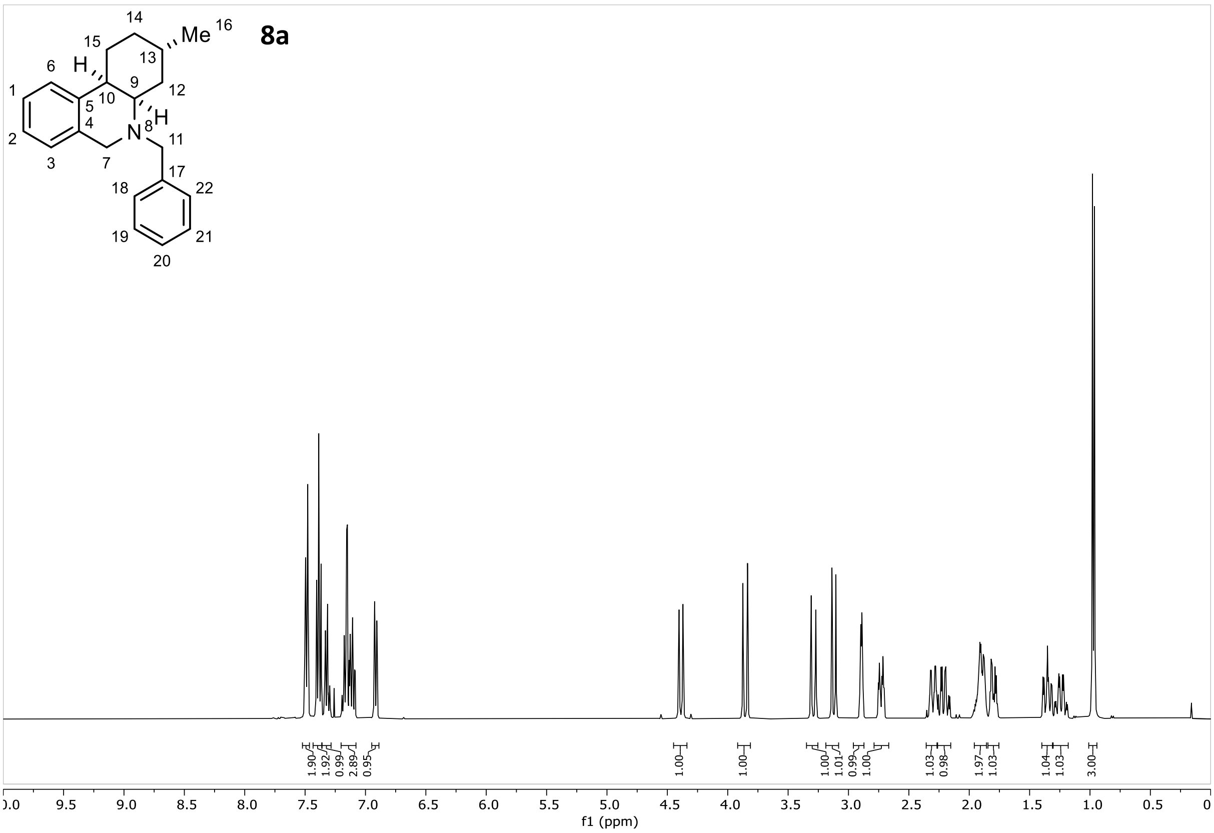
8a
Me 16
1.90
1.92
0.99
2.89
0.95
1.00
1.00
1.00
1.01
0.99
1.00
1.03
0.98
1.97
1.03
1.04
1.03
3.00
f1 (ppm)

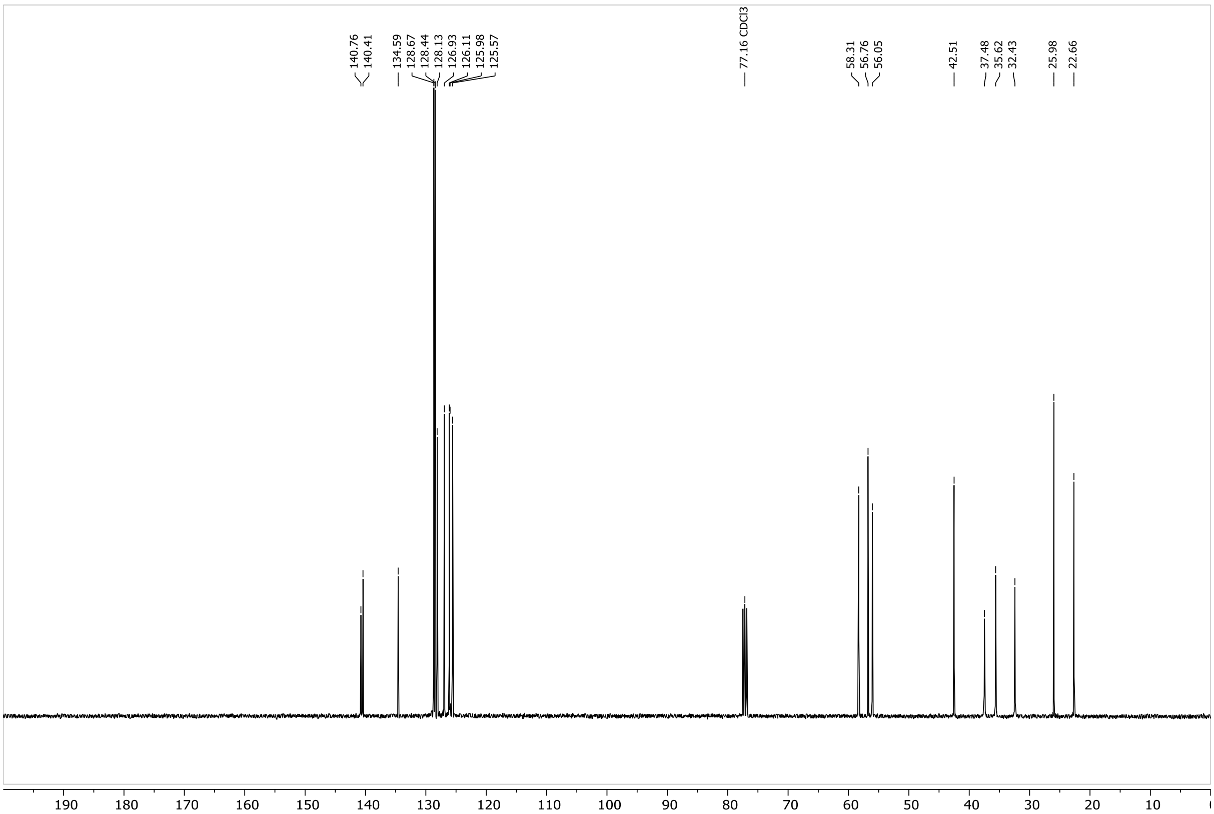
140.76
140.41
134.59
128.67
128.44
128.13
126.93
126.11
125.98
125.57
77.16 CDCl3
58.31
56.76
56.05
42.51
37.48
35.62
32.43
25.98
22.66
f1 (ppm)

8b

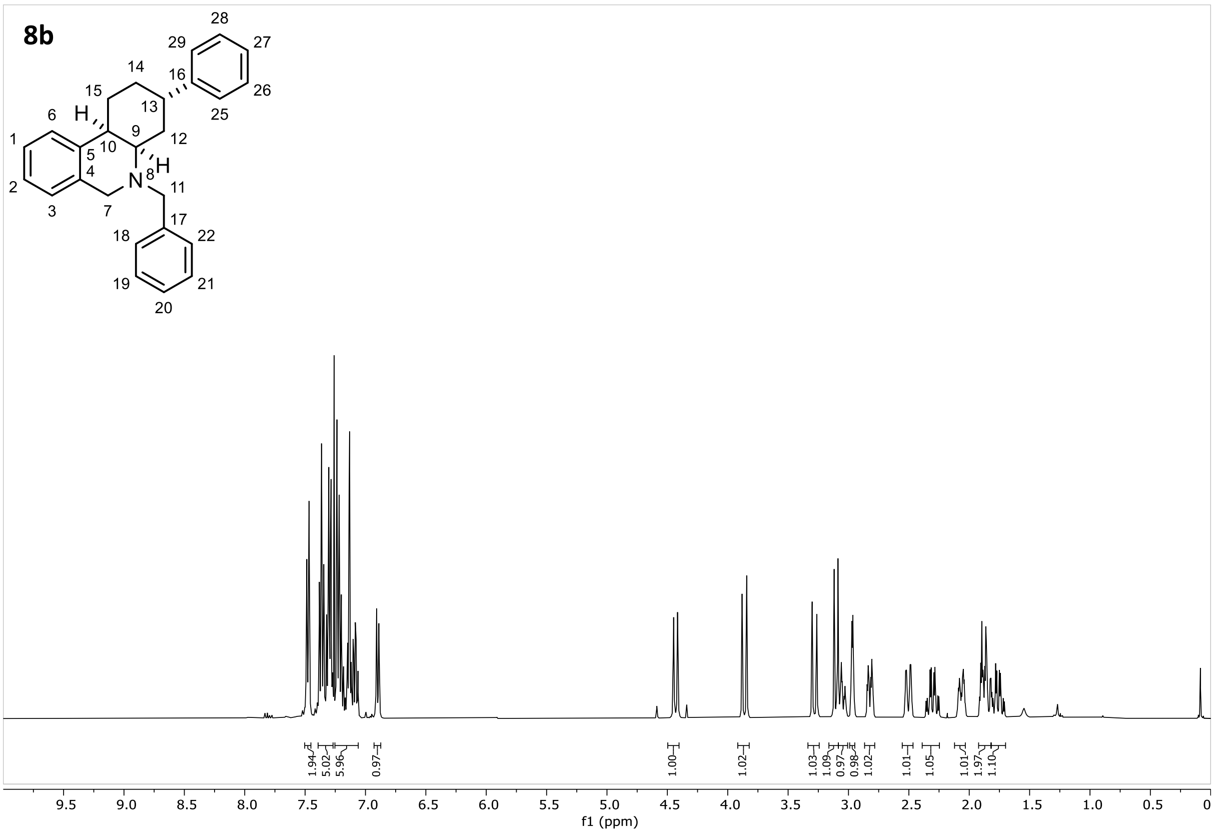
1.94
5.02
5.96
0.97
1.00
1.02
1.03
1.09
0.97
0.98
1.02
1.01
1.05
1.01
1.97
1.10
f1 (ppm)

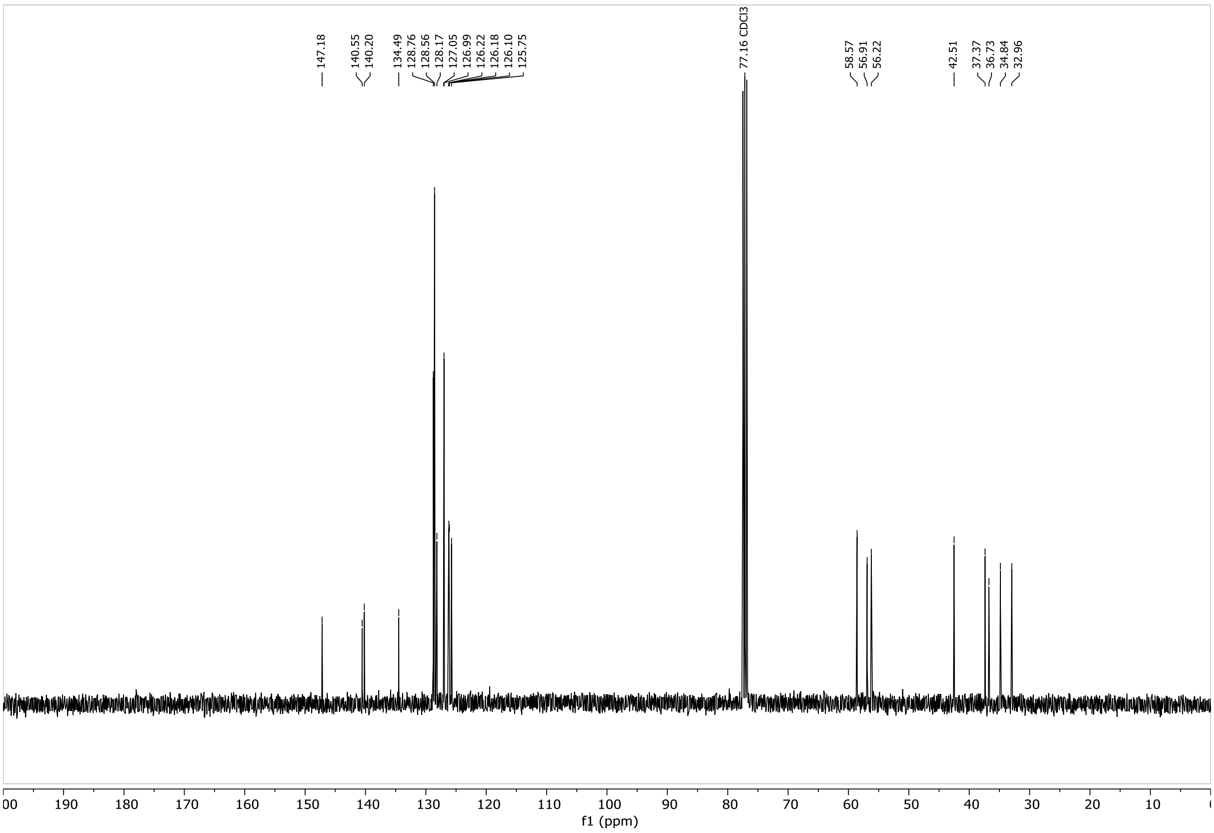
147.18
140.55
140.20
134.49
128.76
128.56
128.17
127.05
126.99
126.22
126.18
126.10
125.75
77.16 CDCl3
58.57
56.91
56.22
42.51
37.37
36.73
34.84
32.96
f1 (ppm)

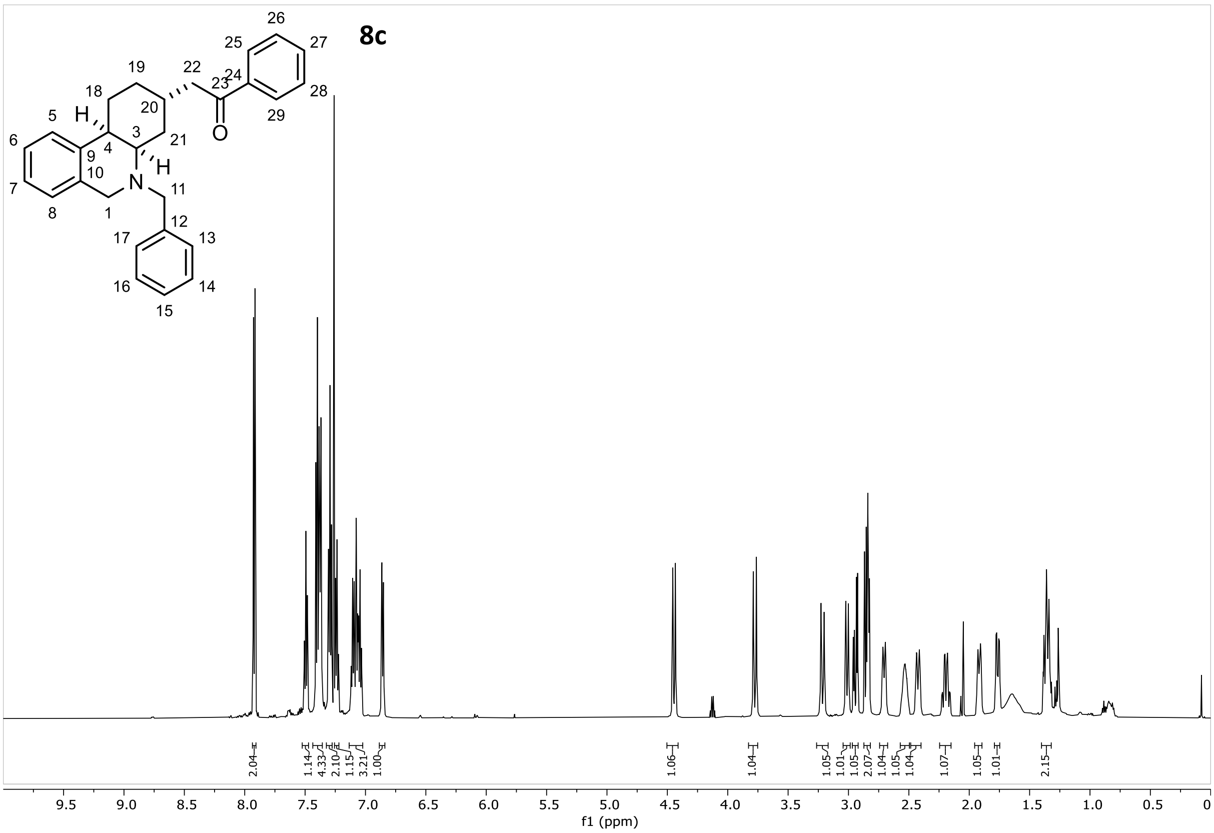
8c
26
25
27
24
23
22
19
18
20
28
29
5
H
6
4
3
21
9
H
10
7
N
11
8
1
12
17
13
16
14
15
O
2.04
1.14
4.33
2.10
1.15
3.21
1.00
1.06
1.04
1.05
1.01
1.05
2.07
1.04
1.05
1.04
1.07
1.05
1.01
2.15
9.5 9.0 8.5 8.0 7.5 7.0 6.5 6.0 5.5 5.0 4.5 4.0 3.5 3.0 2.5 2.0 1.5 1.0 0.5 0
f1 (ppm)
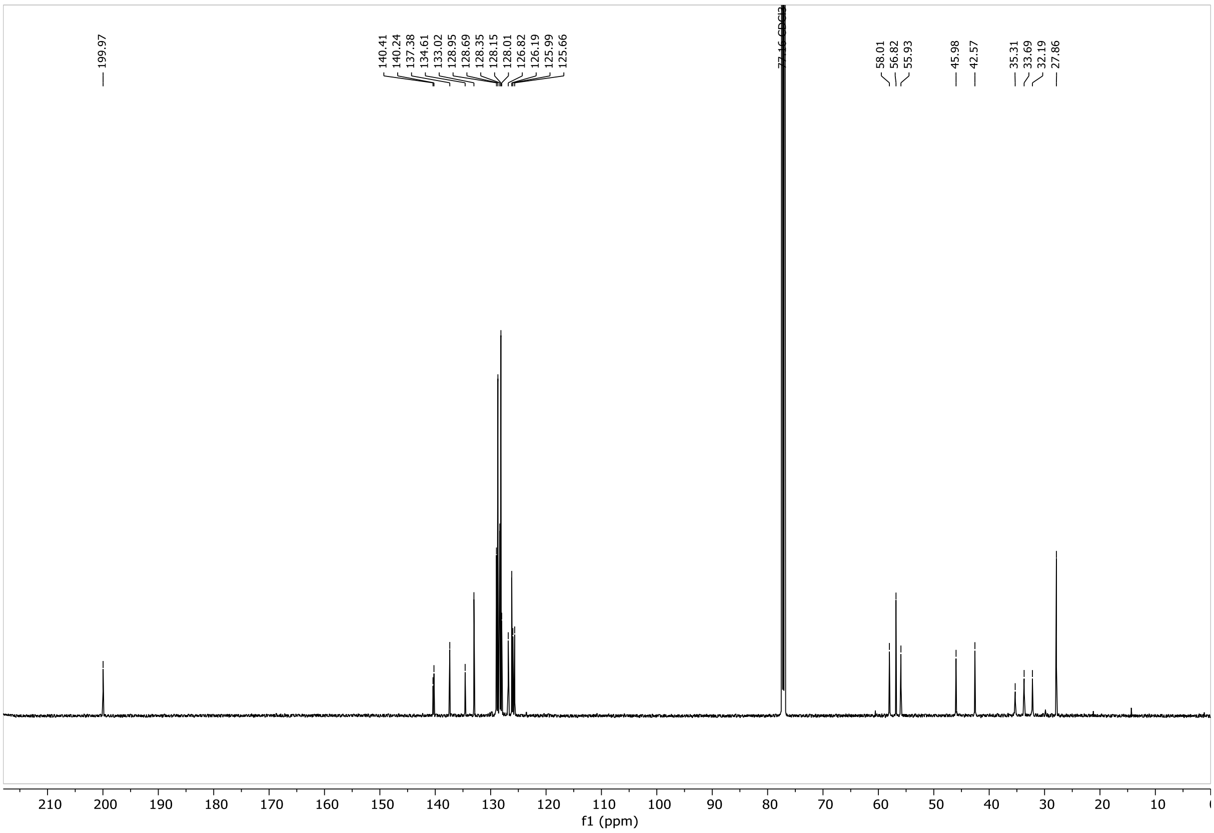
199.97
140.41
140.24
137.38
134.61
133.02
128.95
128.69
128.35
128.15
128.01
126.82
126.19
125.99
125.66
58.01
56.82
55.93
45.98
42.57
35.31
33.69
32.19
27.86
210 200 190 180 170 160 150 140 130 120 110 100 90 80 70 60 50 40 30 20 10
f1 (ppm)

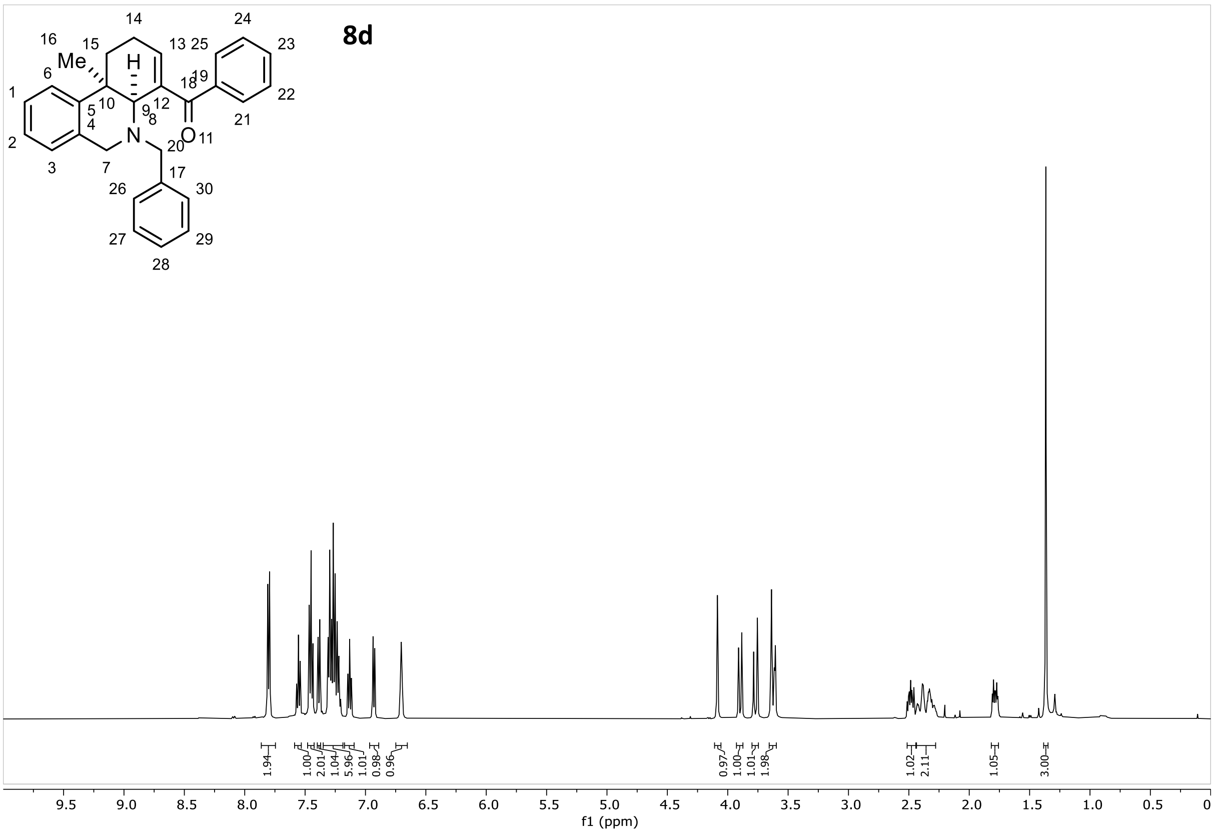
8d
Me
H
N
O
1.94
1.00
2.01
1.04
5.96
1.01
0.98
0.96
0.97
1.00
1.01
1.98
1.02
2.11
1.05
3.00
f1 (ppm)

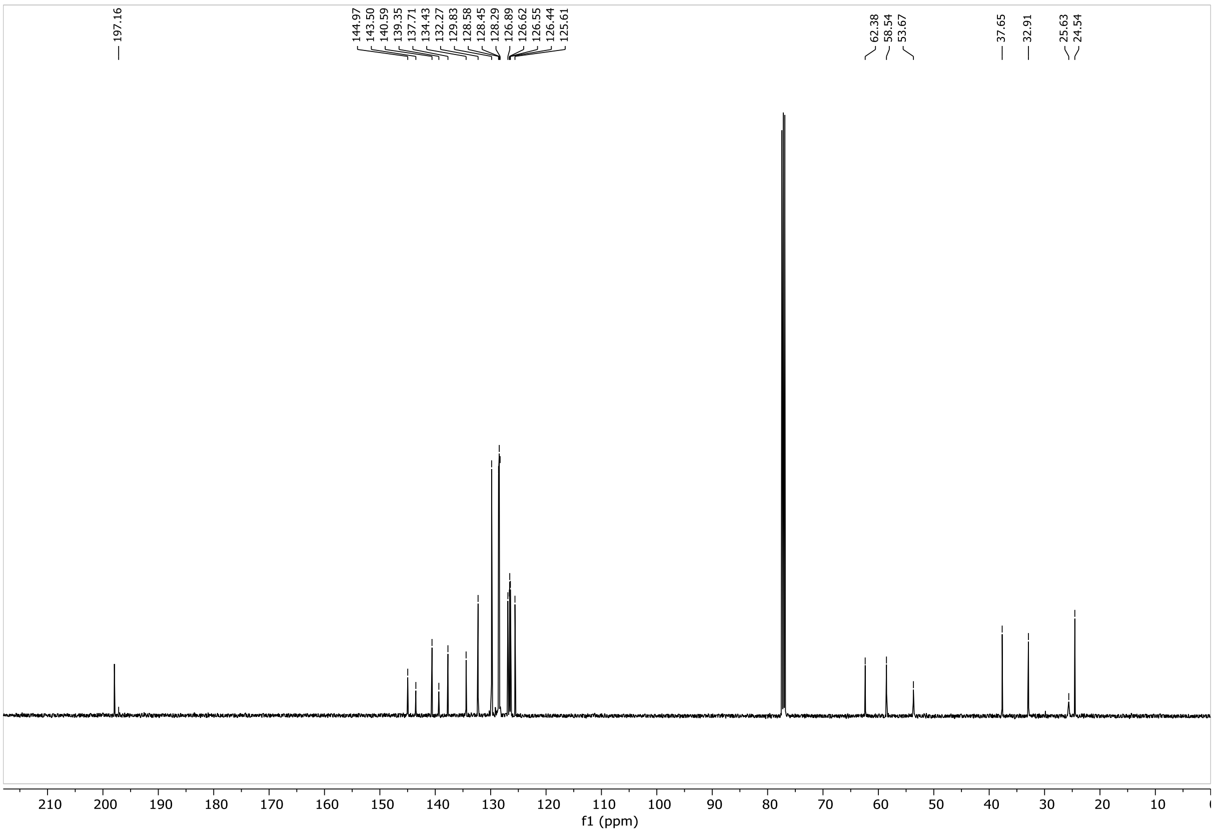
197.16
144.97
143.50
140.59
139.35
137.71
134.43
132.27
129.83
128.58
128.45
128.29
126.89
126.62
126.55
126.44
125.61
62.38
58.54
53.67
37.65
32.91
25.63
24.54
f1 (ppm)

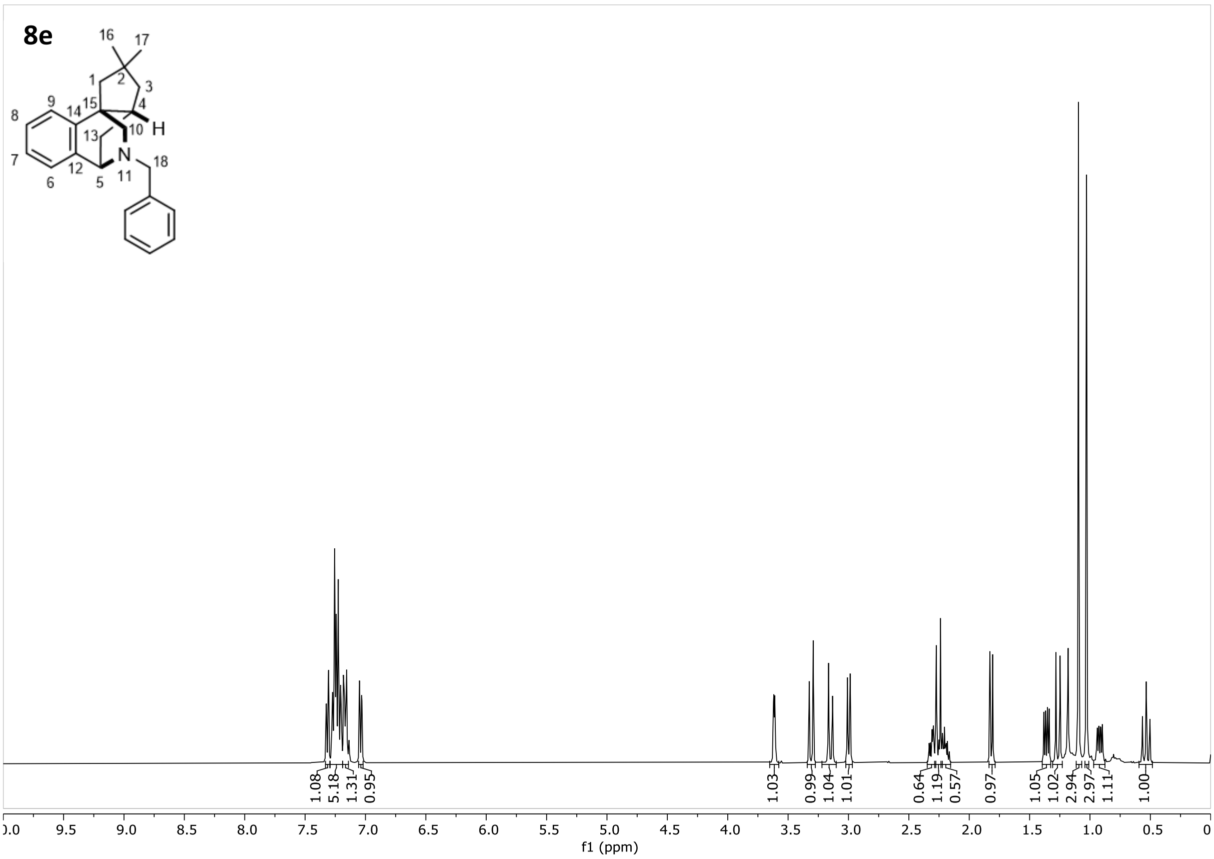
8e
16
17
1
2
3
4
8
9
14
15
13
10
H
7
N
18
6
12
5
11
1.08
5.18
1.31
0.95
1.03
0.99
1.04
1.01
0.64
1.19
0.57
0.97
1.05
1.02
2.94
2.97
1.11
1.00
f1 (ppm)

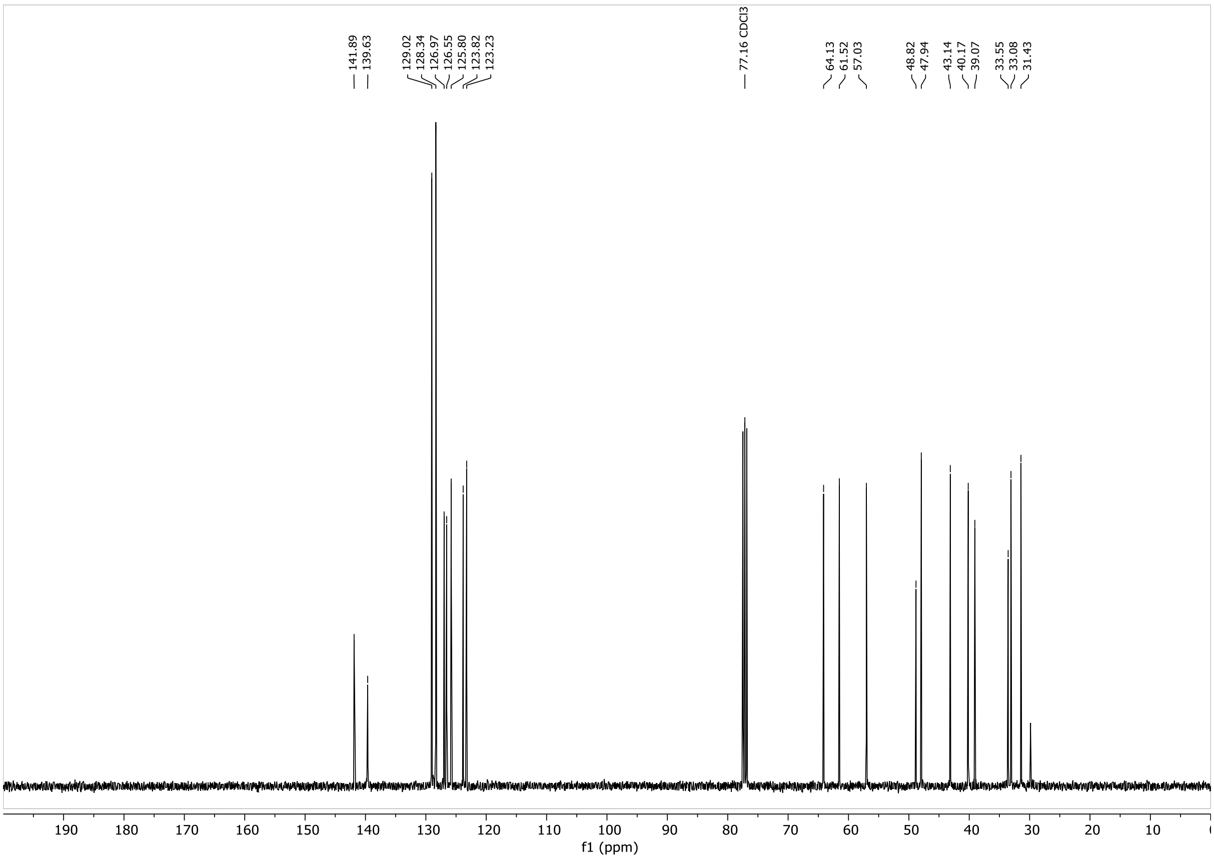
141.89
139.63
129.02
128.34
126.97
126.55
125.80
123.82
123.23
77.16 CDCl3
64.13
61.52
57.03
48.82
47.94
43.14
40.17
39.07
33.55
33.08
31.43
f1 (ppm)
